# Overexpression of *PvWOX3a* in switchgrass promotes stem development and increases plant height

**DOI:** 10.1038/s41438-021-00678-w

**Published:** 2021-12-01

**Authors:** Ruijuan Yang, Zhenying Wu, Chen Bai, Zhichao Sun, Mengqi Wang, Yuzhu Huo, Hailing Zhang, Yamei Wang, Huapeng Zhou, Shaojun Dai, Wenwen Liu, Chunxiang Fu

**Affiliations:** 1 Shandong Provincial Key Laboratory of Energy Genetics and CAS Key Laboratory of Biofuels, Qingdao Institute of Bioenergy and Bioprocess Technology, Chinese Academy of Sciences, 266101 Qingdao, Shandong China; 2 University of Chinese Academy of Sciences, 100049 Beijing, China; 3 Shanghai Normal University, 201418 Shanghai, China; 4 Grass and Science Institute of Heilongjiang Academy of Agricultural Sciences, Harbin, Heilongjiang China; 5 Key Laboratory of Bio-resource and Eco-environment of Ministry of Education, College of Life Sciences, Sichuan University, 610064 Chengdu, China

**Keywords:** Plant morphogenesis, Molecular engineering in plants

## Abstract

Switchgrass (*Panicum virgatum* L.) is an important perennial, noninvasive, tall ornamental grass that adds color and texture to gardens and landscapes. Moreover, switchgrass has been considered a forage and bioenergy crop because of its vigorous growth, low-input requirements, and broad geography. Here, we identified *PvWOX3a* from switchgrass, which encodes a WUSCHEL-related homeobox transcription factor. Transgenic overexpression of *PvWOX3a* in switchgrass increased stem length, internode diameter, and leaf blade length and width, all of which contributed to a 95% average increase in dry weight biomass compared with control plants. Yeast one-hybrid and transient dual-luciferase assays showed that PvWOX3a can repress the expression of *gibberellin 2-oxidase* and *cytokinin oxidase/dehydrogenase* through apparently direct interaction with their promoter sequences. These results suggested that overexpression of *PvWOX3a* could increase gibberellin and cytokinin levels in transgenic switchgrass plants, which promotes cell division, elongation, and vascular bundle development. We also overexpressed *PvWOX3a* in a transgenic miR156-overexpressing switchgrass line that characteristically exhibited more tillers, thinner internodes, and narrower leaf blades. Double transgenic switchgrass plants displayed significant increases in internode length and diameter, leaf blade width, and plant height but retained a tiller number comparable to that of plants expressing miR156 alone. Ultimately, the double transgenic switchgrass plants produced 174% more dry-weight biomass and 162% more solubilized sugars on average than control plants. These findings indicated that *PvWOX3a* is a viable potential genetic target for engineering improved shoot architecture and biomass yield of horticulture, fodder, and biofuel crops.

## Introduction

In vascular plants, the shoot system usually grows aboveground; it determines the morphology of the plant and comprises the aerial biomass. The shoot system consists of the stem and the organs attached to the main stem, such as leaves, buds, flowers, and fruits. The shoot architecture is characterized by repeating units called phytomers. Each phytomer is made up of an internode, leaf, and axillary meristem. All aboveground organs are generated from the shoot apical meristem (SAM), and are organized, established, and maintained through a complex gene regulatory network^1^. The stem is composed of nodes and internodes that form the central axis of the plant shoot system. The main function of the stem is to provide support for the plant, and its vascular system transports water and nutrition throughout the plant. Recent studies have suggested that the vascular system of stems can transport miRNAs, phytohormones, and proteins long distances to their target tissues throughout the plant^2–4^. Previous studies have proposed that genetic manipulation of stem development can largely increase biomass yield^5–9^. Thus, the stem architecture phenotype critically affects other aspects of plant development and growth and consequently serves as a major contributing factor in determining plant morphology and aboveground biomass.

Phytohormones, including gibberellin (GA), cytokinin (CK), auxin, ethylene, and brassinosteroid (BR), have all been shown to participate in the regulation of stem development^10^. Among them, GA determines internode length and plant height by controlling cell division and elongation^11,12^. Interruption of GA biosynthesis, perception, and signaling can result in dwarf or semidwarf phenotypes in plants^13,14^. In the GA biosynthetic pathway, *ent*-kaurene is converted to GA_12_ by cytochrome P450, *ent*-kaurene oxidase (KO), and *ent*-kaurenoic acid oxidase (KAO). Then, GA_12_ is converted into bioactive GA_1_, which is regulated by three dioxygenases, GA 20-oxidase (GA20ox), GA 3-oxidase (GA3ox), and GA 2-oxidase (GA2ox)^11^. Among these enzymes, GA2ox irreversibly catalyzes the conversion of bioactive GA or its precursors via 2-β hydroxylation into inactive catabolites^11^. Silencing *GA2ox* can enhance plant growth and fiber production^15^, while ectopic overexpression of *GA2ox* causes dwarfism and impairs stem lignification in transgenic *Arabidopsis* and switchgrass^7,16^. Moreover, overexpression of *ZmGA20ox* in maize can lead to a higher content of bioactive GAs and improve biomass yield together with increased lignin contents^9^. Thus, regulating key genes in the gibberellin pathway could serve as an effective strategy to improve biomass production in monocot plants.

*WUSCHEL*-related homeobox (WOX) genes encode one family of homeodomain (HD)-containing transcription factors that are broadly conserved in regulating diverse developmental programs, including SAM formation and maintenance, lateral organ generation, and vascular formation^17,18^. In *Arabidopsis*, the WOX family is divided into three clades: the modern WUSCHEL (WUS) clade (AtWUS and WOX1-7), the intermediate clade (AtWOX8, 9, 11, and 12), and the ancient clade (AtWOX10, 13 and 14). Spatial and temporal expression patterns of WOX genes are important for their functions. Among them, members of the WOX3 subclade perform essential functions in the regulation of leaf and other lateral organ development in *Arabidopsis*, rice, and maize^19–21^. In rice, overexpression of *OsWOX3a* leads to a severe dwarf phenotype associated with wider leaves^20^. This dwarf phenotype in rice is related to the downregulation of GA biosynthesis. Investigation of its molecular interactions revealed that OsWOX3a can bind to the promoter of *KAO* and thereby repress its expression^22^. Rice *NARROW LEAF2* (*NAL2*) and *NARROW LEAF3* (*NAL3*) both encode OsWOX3a transcription factors. The *NAL2/3* double mutant, however, exhibits a narrower leaf blade, a thinner stem, and reduced vascular bundles with normal plant height^20,23^. Further study suggested that OsWOX3a potentially affects auxin transport^23^. In addition, WOX1 is functionally redundant with WOX3 in regulating lateral organ and margin development in dicot species^24^. However, monocot species only contain the WOX3 subclade but not the WOX1 subclade^19,24^. In *Medicago truncatula*, the AtWOX1 ortholog STENOFOLIA (STF) regulates leaf blade outgrowth and vascular patterning by modulating auxin and CK^25^. Heterologous overexpression of *STF* in rice, *Brachypodium*, and switchgrass can increase leaf width and stem diameter. Subsequent studies have shown that STF can directly bind to the promoters of cytokinin oxidase/dehydrogenase (*CKX*) genes and repress their expression, leading to elevated CK accumulation in leaf and stem tissue^8^. However, previous studies examining the role of WOX3a in stem development have focused on Arabidopsis, rice, and maize, whereas very few studies have investigated the mechanism by which WOX3 functions in stem development in perennial monocot grasses.

Switchgrass (*Panicum virgatum* L.) is a noninvasive, perennial, upright clumping ornamental grass that provides an attractive vertical element for gardening and landscaping. Because of its vigorous growth, effective use of nutrients and large native geographic range, switchgrass is also regarded as a multiple-purpose crop that can be used for both livestock fodder and biofuel production^5,26^. Our previous study produced numerous transgenic switchgrass lines with increased tillers through overexpressing miR156. The bushy morphology significantly improves the ornamental value of switchgrass. However, more tillers are always associated with thinner stems, which limits the utilization of these novel switchgrass germplasms in garden and landscape ornaments and forage and bioenergy production. Here, we found that overexpression of *PvWOX3a* in switchgrass increased plant height, internode length and diameter, and leaf blade length and width, and improved biomass yield. Moreover, our results revealed that PvWOX3a can directly interact with the promoters of *GA2ox* and *CKX4b*, repressing their expression. These findings suggest that PvWOX3a participates in regulating gibberellin and cytokinin catabolism in switchgrass based on the functions of the genes that it suppresses. Furthermore, in a miR156-overexpressing transgenic switchgrass line that exhibited a higher tiller number, thinner internodes, and a narrower leaf blade, the overexpression of *PvWOX3a* (i.e., in double transgenic switchgrass plants) led to significant increases in internode length and diameter, leaf blade width, and plant height while maintaining a comparable tiller number. Finally, different transgenic switchgrass lines that overexpressed *PvWOX3a*, either alone or with miR156, yielded a significantly higher dry-weight biomass than control plants. Our findings thus demonstrate that the engineering of *PvWOX3a* expression in switchgrass could serve as a viable avenue for horticulture, forage, and bioenergy crop breeding programs seeking to improve plant features and biomass yield.

## Materials and methods

### Plant materials and growth conditions

A lowland switchgrass cultivar Alamo was used in this study. The developmental stages of switchgrass were divided into three vegetative stages (V1, V2, and V3), five elongation stages (E1, E2, E3, E4, and E5), and three reproductive stages (R1, R2, and R3), in accordance with previously published studies^27^. All switchgrass and *Nicotiana benthamiana* plants (used for subcellular localization and transient dual-luciferase assays) were grown in a greenhouse under long-day conditions (16 h light/8 h dark). Supplemental lighting was provided to extend the photoperiod to 390 μEm^−2^S^−1^.

### Sequence alignment and phylogenic analysis

Amino acid sequences of WOXs retrieved from cDNA libraries of *Arabidopsis thaliana*, *Oryza sativa*, and *Medicago truncatula*^28,29^ were downloaded from PlantTFDB v5.0. Putative amino acid sequences of switchgrass WOXs were obtained by BLAST search of the switchgrass genome database from Phytozome (http://www.phytozome.net/) with a threshold e-value of 1e^−10^ and were then confirmed by the presence of the conserved homeobox domain (HD) of WOXs^30^. We performed multiple alignments of WOX sequences using MEGA X software^31^. After manual removal of poorly aligned sequences from the alignment, we reconstructed a neighbor-joining phylogeny of 72 WOX sequences (26 WOXs from switchgrass, 15 WOXs from *Arabidopsis thaliana*, 13 WOXs from *Oryza sativa*, and 18 WOXs from *Medicago truncatula*) using MEGA X with 1000 bootstrap replicates.

### Total RNA isolation and quantitative RT–PCR analysis

To analyze the relative expression patterns of *PvWOX3a* in switchgrass, total RNA was extracted from E4I2 (Internode 2 at the E4 stage), E4L2 (Leaf 2 at the E4 stage), E3I2, E3L2, inflorescence, and crown bud tissues using a TRIzol Kit (TransGen Biotech, Beijing, China), and reverse transcribed into cDNA with a PrimeScript^TM^ RT Kit (TransGen Biotech, Beijing, China) after treatment with gDNA Eraser (Takara, Dalian, China). SYBR Premix ExTaq^TM^ (Takara, Dalian, China) was used for qRT–PCR, and the cycle thresholds were determined using a Roche LightCycler^®^ 480 II sequence detection system (Roche, Shanghai, China). The data were normalized to the level of *PvUbq2* transcripts (GenBank accession NO: HM209468). In addition, the top two internodes of control and transgenic plants were used for total RNA extraction when control plants reached the E5 stage. qRT–PCR was used to validate the RNA sequencing results and for further analysis of differentially expressed genes among control plants and transgenic plants. The primers used for qRT–PCR are listed in Table S3.

### Subcellular localization and confocal microscopy

*PvWOX3a* was isolated from switchgrass tiller bud tissues using primers based on the transcript sequence of *Pavir.8KG017200* downloaded from Phytozome (Table S3). The *35S::PvWOX3a-cGFP* vector construct was transformed into *Agrobacterium* strain *EHA105* for transient expression in tobacco. Young leaves of 4-week-old tobacco plants were used for needleless syringe infiltration, as previously reported^32^. GFP fluorescence was visualized with an Olympus FV-1000 microscope (Olympus, Japan) following leaf infiltration.

### Gene constructs and transformation

The *PvWOX3a* coding sequence was cloned into the pENTR^TM^/D-TOPO vector, and the final binary vector of pANIC6B-PvWOX3a and pMDC32-PvWOX3a was constructed by LR recombination reactions (Invitrogen, Shanghai, China)^33,34^. The pANIC6B-PvWOX3a vector was then transferred into *Agrobacterium* strain *EHA105*. To generate *PvWOX3a*-overexpressing transgenic plants, a single genotype, high-quality, embryogenic callus line was employed for *Agrobacterium*-mediated transformation following the procedure described by Xi et al.^35^. In addition, a miR156-overexpressing transgenic line (miR156OE-27, with the selectable marker for bialaphos resistance), previously generated in our lab, was employed for cotransformation with the pMDC32-PvWOX3a construct. The PvWOX3aOE transgenic plants and miR156OE_WOX3aOE transgenic plants were screened by genomic PCR. The relevant primers are listed in Table S3.

### Development and growth analysis

The control plants used for morphological analysis were generated from transgenic plants transformed with an empty vector. Plant height, the diameter of I3, the length of L3, and the width of L3 were measured using the tillers of 3-month-old control and transgenic plants. After 4 months of growth in the greenhouse, control plants, and positive transgenic plants were harvested to evaluate the fresh biomass yield. The aboveground biomass dry-weight yield was evaluated after drying plants in an oven at 40 °C for 96 h.

### Histological analysis

The E3I2 tissues of control plants and transgenic plants were fixed and embedded as described. The tissues were then sliced into 3 μm cross and longitudinal sections with a Leica RM2016 microtome, affixed to microscope slides, and stained with Safranin O-Fast Green. Images were taken with a Nikon Eclipse E100 microscope. ImageJ software was used to measure the cell length.

### Yeast one-hybrid assays

The full-length cDNA of *PvWOX3a* was fused to the active domain of pGADT7 (AD). Approximately 2.5 kb sequences from each respective promoter region of *PvGA2ox3*, *PvGA2ox7*, and *PvCKX4* were individually fused to the pHIS2.1 vector. Each of the six construct groups (AD plus pHIS2.1-*ProGA2ox3*; AD-WOX3a plus pHIS2.1-*ProGA2ox3*; AD plus pHIS2.1-*ProGA2ox7*; AD-WOX3a plus pHIS2.1-*ProGA2ox7*; AD plus pHIS2.1-*ProCKX4b*; and AD-WOX3a plus pHIS2.1-*ProCKX4b*) was then transformed into the yeast strain Y187, and the empty AD plasmid was used as a negative control. All of the yeast strains were grown on SD selective medium (SD/-His-Leu) and observed for 7 days. The Y1H assay was performed according to the manufacturer’s instructions (Clontech). Primers used for Y1H are listed in Table S3.

### Dual-luciferase (LUC) analysis

The *35S::PvWOX3a* vector was transformed into *EHA105* to serve as an effector. Approximately 2.5 kb sequences from each promoter region of *PvGA2ox3*, *PvGA2ox7*, and *PvCKX4* were inserted into the pGreenII 0800-LUC vector^36^ and then cotransformed with the helper plasmid pSoup19 into *EHA105* to serve as the reporter. The negative control *35S::NosT* plasmid was transformed into *EHA105*. The experimental and control groups were infiltrated into opposite positions on the same *N. benthamiana* leaves. After 3 days of growth under long-day white light conditions, the leaves were collected, and a Dual-Luciferase Reporter Assay System (Promega) was used to determine the relative ratio of firefly luciferase to Renilla luciferase. Three plants served as biological replicates, and one leaf from each plant was measured for each construct pair.

### Cell wall composition analysis

All of the aboveground dry tissues were ground for the following analyses. Soluble extracts were removed by successive extraction procedures, as described by Chen and Dixon, to generate the cell wall residues (CWR)^37^. The Klason method was used to quantify the lignin content^38^, and the supernatant liquid was used to determine the cellulase and hemicellulose contents, as described previously^39^.

### Determination of enzymatic hydrolysis efficiency

To perform the saccharification assays of switchgrass, CWR was digested by pretreatment with diluted H_2_SO_4_ (15%) at 121 °C for 60 min. Samples were then exposed to a cellulase and cellobiase mixture for 72 h after washing with Milli-Q water, following the analytical procedures described by the National Renewable Energy Laboratory (LAP-009: Enzymatic Saccharification of Lignocellulosic Biomass). The solubilized sugars were detected by phenol-sulfuric acid assays^40^. The solubilized sugar yields released by enzymatic hydrolysis were calculated as follows: solubilized sugar yields (g/plant) = cell wall carbohydrate yield of switchgrass biomass (g/plant) × saccharification efficiency.

### Transcriptome analysis

Total purified RNAs from selected WOX3aOE transgenic plants and control plants were extracted as described above and reverse transcribed into a cDNA library using SuperScript^TM^ II Reverse Transcriptase (Invitrogen, Chicago, USA). Sequencing was performed using an Illumina NovaSeq^TM^ 6000 (LC sciences, Houston, USA). Transcripts were assembled using HISAT2 software and quantified using StringTie. gffcompare software was used to construct a comprehensive transcriptome. The differentially expressed genes were selected for GO enrichment analysis with the following parameters: FPKM > 1, Log_2_FC < 0.5 for downregulated DEGs, Log_2_FC > 1 for upregulated DEGs and *p* < 0.05.

### Statistical analysis

Switchgrass plants were propagated by transferring the same number of tillers into each pot. Three copies of each line were grown in a single one gallon pot. The mean values were used for statistical analysis. Data from each trait were subjected to Student’s *t* test or analysis of variance (ANOVA). The significance of treatments was determined at the *p* < 0.05 level. Standard errors are provided in all tables and figures, as appropriate. All statistical analyses were performed with GraphPad Prism 7.

## Results

### Identification of PvWOX3a in switchgrass

**Fig. 1:**
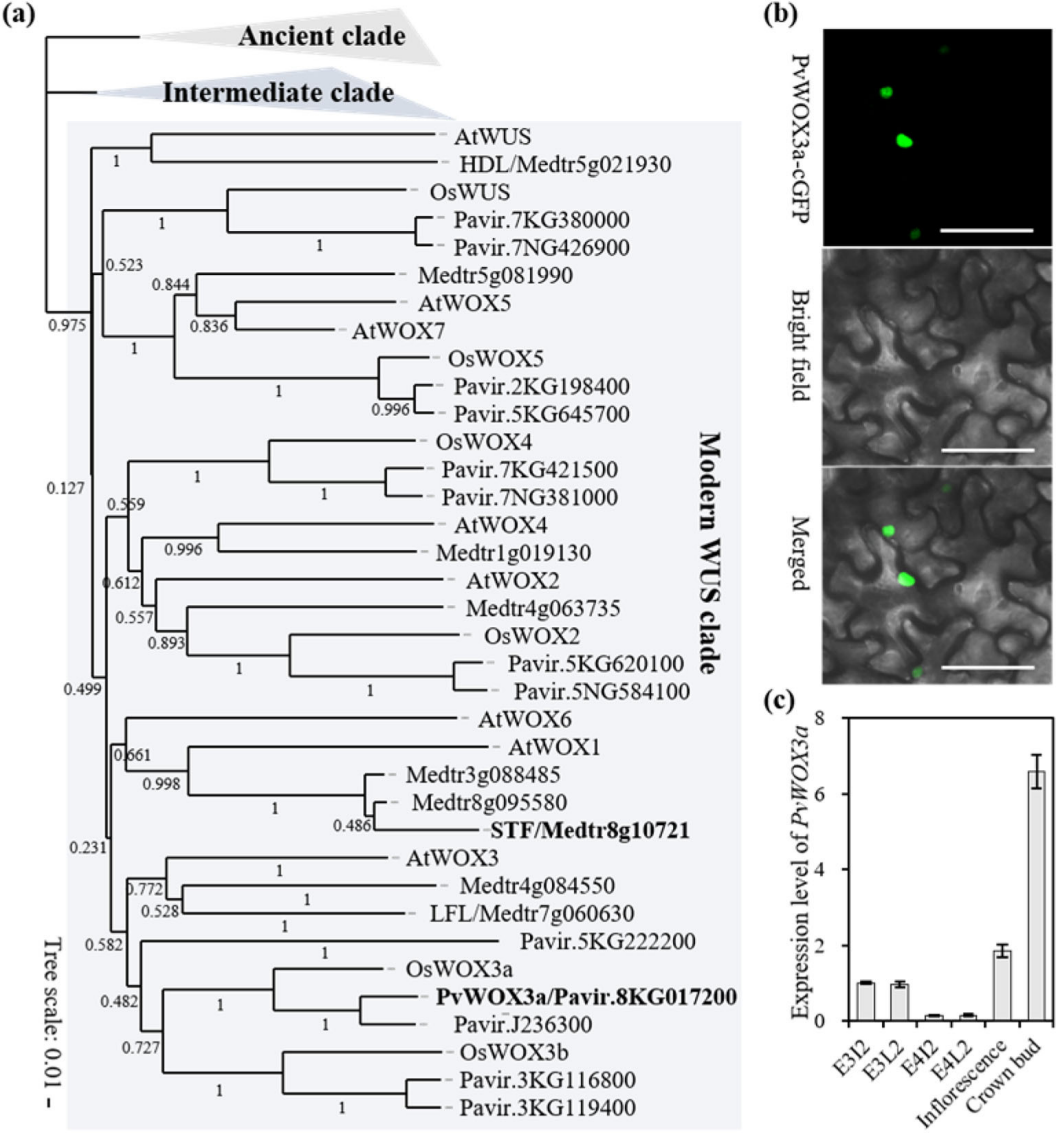
Molecular characterization of *PvWOX3a*. **a** Neighbor-joining phylogenetic tree of WOX3a-related proteins from *Panicum virgatum*, *Arabidopsis thaliana*, *Medicago truncatula*, and *Oryza sativa*. The tree was constructed from an alignment conducted using MEGA X with 1000 bootstrap replicates, and the bootstrap values of each node are shown on the tree. **b** Subcellular localization of the WOX3a-eGFP fusion reporter in *N. benthamiana* cells by confocal laser microscopy. GFP, bright field, and merged images are shown. Scale bar = 50 μm. **c** Expression patterns of *PvWOX3a* in switchgrass. E4I2 Internode 2 at the E4 stage, E4L2 Leaf 2 at the E4 stage, E3I2 Internode 2 at the E3 stage, E3L2 Leaf 2 at the E3 stage, Inflorescence; and Crown buds. qRT–PCR was normalized to the expression of switchgrass *PvUbq2*. Values are means ± SEs (*n* = 3)

To identify candidate PvWOXs potentially involved in switchgrass stem development, 26 WOXs in the Phytozome plant data portal of the JGI genome database were retrieved. The other WOXs that were retrieved for comparison belonged to *Arabidopsis thaliana*, *Medicago truncatula*, and *Oryza sativa*. Phylogenetic analysis showed that the PvWOXs were clustered into three distinct clades, namely, an ancient clade, an intermediate clade, and a modern WUS clade, which aligned with previously published phylogenies^41^ (Fig. 1a). No homolog of STF was retrieved in switchgrass due to the loss of WOX1 orthologs in monocot species (Fig. 1a). Given that WOX1 and WOX3 were found to perform redundant functions in dicots, we selected PvWOX3a (Pavir.8KG017200), a member of the WOX3 subclade, for further characterization.

*PvWOX3a* encodes a 247-amino acid protein that contains a conserved homeobox domain (HD) at its N-terminus, a putative acidic domain, and a WUS box motif (“TLXLFP”) at its C-terminus (Fig. S1). These domains have been shown to mediate WOX transcriptional regulatory activity, implying that PvWOX3a may also be a WOX transcription factor^23,28^. Furthermore, transient reporter fusion expression assays to observe the subcellular localization of PvWOX3a showed a strong green fluorescence signal in the nuclei of tobacco leaf cells (Fig. 1b). We also measured the expression levels of *PvWOX3a* in different organs/tissues of wild-type switchgrass using quantitative real-time PCR (qRT–PCR), which revealed that *PvWOX3a* was highly expressed in E3I2 (Internode 2 at the E3 stage) and E3L2 (Leaf 2 at the E3 stage), inflorescences, and crown buds (Fig. 1c). In contrast, *PvWOX3a* had lower expression levels in mature stems (E4I2, Internode 2 at the E4 stage) compared to organs with rapid cell division (Fig. 1c). Taken together, PvWOX3a may act as a WOX transcription factor and be expressed in rapidly growing organs.

### Overexpression of *PvWOX3a* in switchgrass promoted plant height and biomass yield

**Fig. 2:**
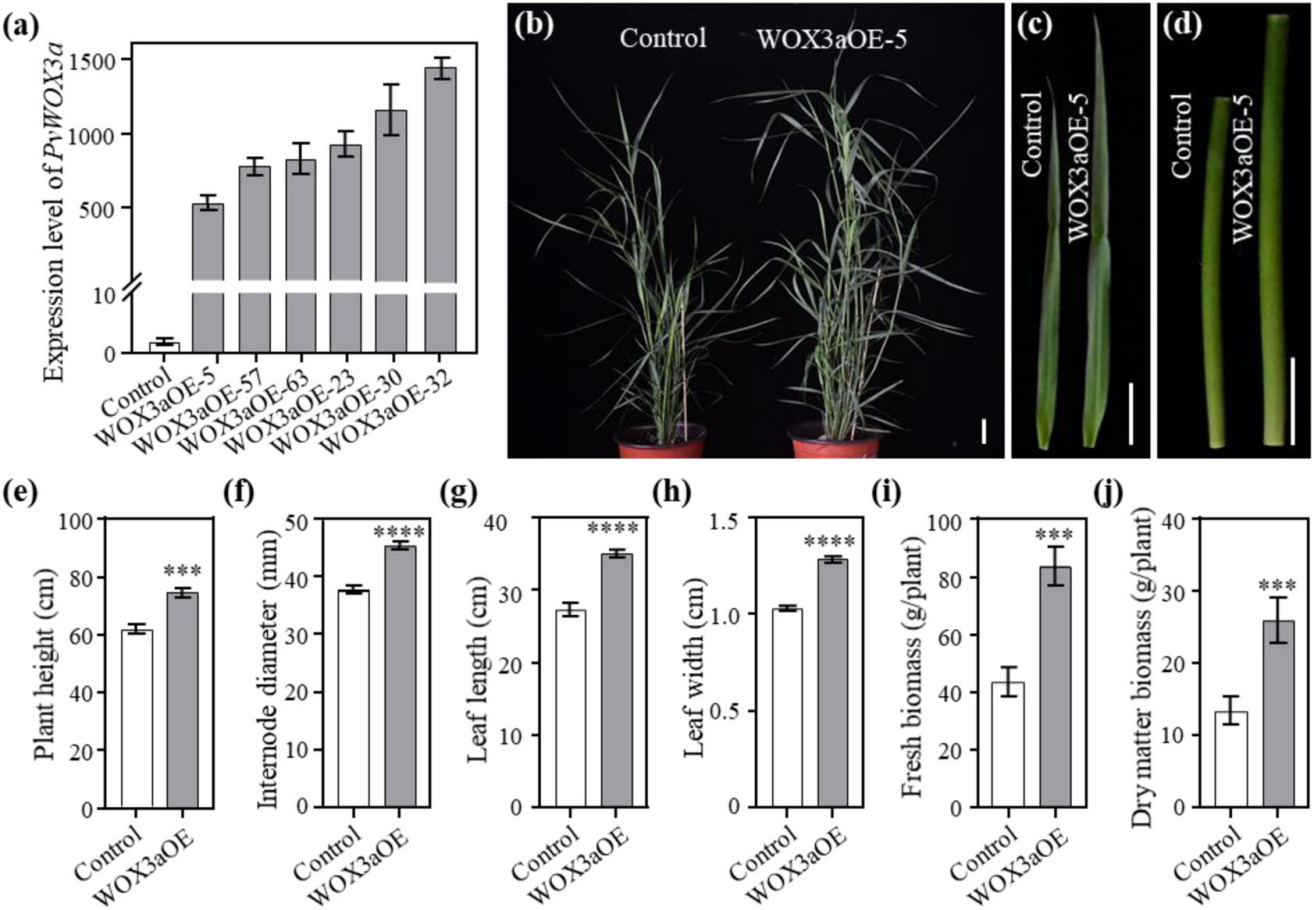
Morphological characterization of *PvWOX3a*-overexpressing transgenic plants. **a** The expression levels of *PvWOX3a* in transgenic lines revealed by qRT–PCR. Switchgrass *PvUbq2* was used for normalization. Values are means ± SEs (*n* = 3). **b** Gross phenotypic characterization of switchgrass plants overexpressing *PvWOX3a* (WOX3aOE). Control plants carried the pANIC6B empty vector. Scale bar = 5 cm. Leaf 3 at the E4 stage (**c**) and Internode 3 at the E4 stage (**d**) of control and WOX3aOE transgenic plants are shown. Scale bar = 5 cm. Three-month-old tillers were used to measure plant height (**e**), diameter of Internode 3 (**f**), length of Leaf 3 (**g**), and width of Leaf 3 (**h**). Three tillers from the same plant were measured for each replicate. Fresh weight biomass yield (**i**) and dry-weight biomass yield (**j**) of transgenic switchgrass plants. The control plants and WOX3aOE transgenic plants were harvested after 4 months of growth in the greenhouse. Values are means ± SEs (*n* = 6). Asterisks represent significant differences determined by Student’s *t* test. ****p < 0.0001; ***p < 0.0002

To elucidate the function of PvWOX3a in switchgrass stem development, we overexpressed *PvWOX3a* driven by the maize ubiquitin promoter in switchgrass plants. All transgenic lines were produced from a single genotypic embryogenic switchgrass callus line through *Agrobacterium*-mediated transformation, which excluded the potential influence of the genetic background of switchgrass on plant growth and development. Twenty-three independent positive transgenic switchgrass lines were identified by genomic PCR. The control plants were generated with the pANIC6B empty vector, which was used as the backbone for constructing the *PvWOX3a*-overexpressing vector. This process excluded the potential influence of the variations in switchgrass genetic background on plant growth and development. Twenty-three independent, positive, transgenic switchgrass lines were identified by genomic PCR. qRT–PCR analysis revealed no <15-fold upregulation of *PvWOX3a* in the transgenic switchgrass plants compared with the controls (Fig. 2a).

Six *PvWOX3a*-overexpressing transgenic lines (WOX3aOE), WOX3aOE-5, −23, −30, −32, −57, and −63, were randomly selected as representatives of the 23 transformant lines for further morphological analysis, which revealed obvious and consistent differences in morphological characteristics between the WOX3aOE lines and control plants (Fig. 2b). To quantify these trait differences, we measured the plant height, internode diameter, leaf blade length and width, tiller number, and flowering time of WOX3aOE lines and control plants. The results showed that the WOX3aOE transgenic plants displayed significantly greater plant height (by ~15 cm on average) than control plants, potentially due to *PvWOX3a-*mediated promotion of internode elongation (Fig. 2b, d, e). In addition, compared with the control plants, the WOX3aOE lines exhibited thicker internodes as well as longer and wider leaf blades (Fig. 2c, f, g, h). Moreover, there were no significant differences in tiller number or flowering time between control plants and WOX3aOE lines (Table S1). Finally, we compared biomass yield between the control and transgenic switchgrass plants and found that the WOX3aOE lines exhibited a 93% increase in fresh biomass yield (Fig. 2i) and a 95% increase in dry biomass yield (Fig. 2j) compared with the control plants. These results clearly indicate that PvWOX3a is a positive regulator of plant height and biomass yield in switchgrass.

### Effects of *PvWOX3a* overexpression on cell proliferation, vascular bundle formation, and cell wall composition

**Fig. 3:**
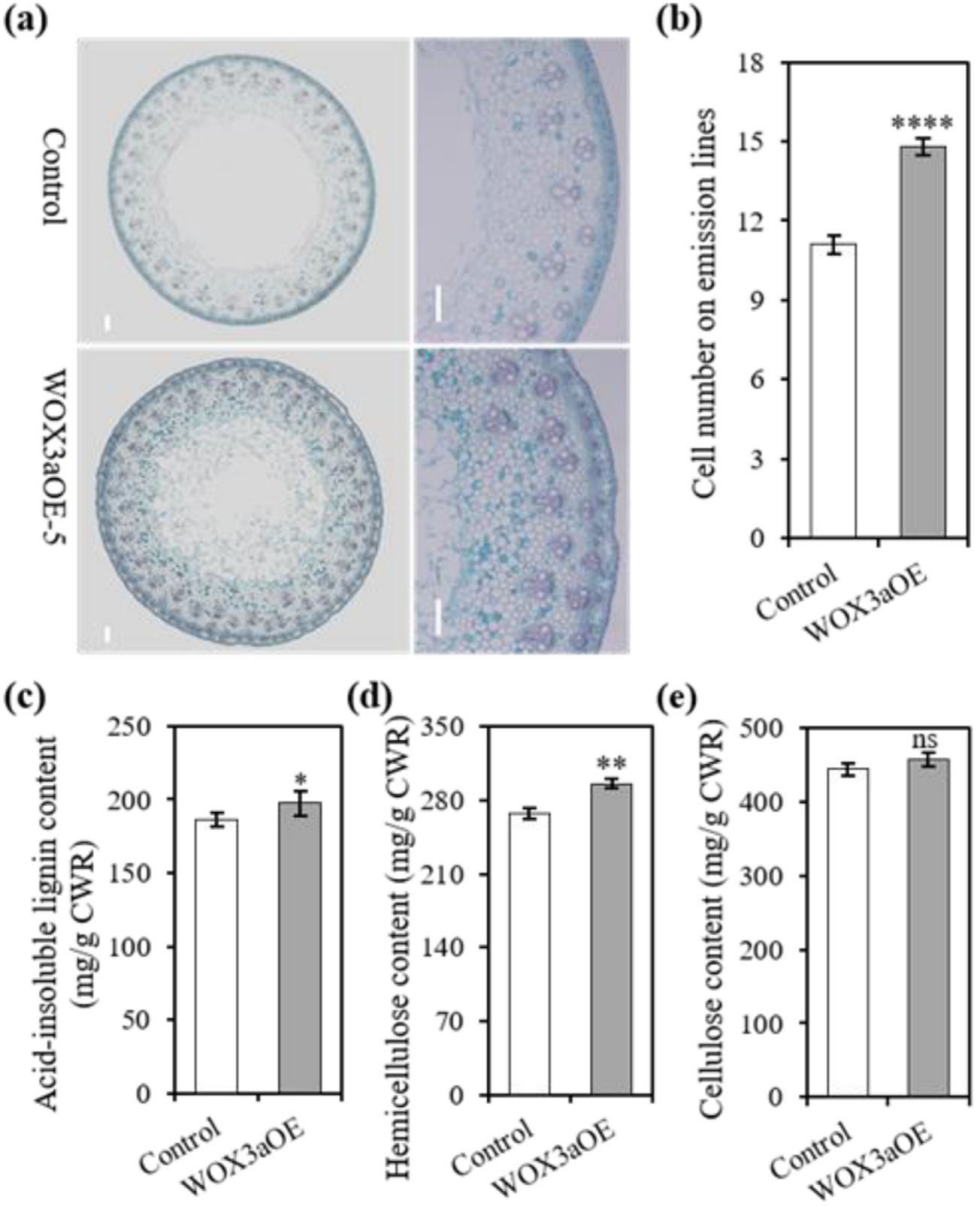
Overexpression of *PvWOX3a* in switchgrass promotes cell proliferation and vascular development and affects cell wall composition. **a** Cross sections of control and WOX3aOE internode bases of Internode 2 at the E4 stage. Scale bar = 200 μm. **b** Cell numbers were determined by counting along the radius at the 12 o’clock position from the outer edge of the pith to the epidermis in cross sections excised from the base of Internode 2 at the E4 stage of control and WOX3aOE transgenic plants. Values are means ± SEs (*n* = 9). **c** Acid-insoluble lignin content of three independent WOX3aOE lines and control plants. Hemicellulose content (**d**) and cellulose content (**e**) of three independent WOX3aOE lines and control plants. Values are means ± SEs (*n* = 3). Asterisks represent significant differences determined by Student’s *t* test. ****p < 0.0001; **p < 0.0021; *p < 0.0332; ns means no significance

Histological staining of internode cross sections was used to explore the reason for the increased internode diameter of WOX3aOE transgenic plants. The results showed that the WOX3aOE lines had a higher cell number and more vascular bundles than control plants (Fig. 3a, b). Consistent with previous observations in *STF-*overexpressing transgenic switchgrass plants, we observed that the expression levels of *cytokinin oxidase/dehydrogenase 4b* (*PvCKX4b*) were downregulated more than 4-fold in WOX3aOE transgenic plants compared to control plants (Fig. S2a). Moreover, promoter motif analysis and yeast one-hybrid and luciferase reporter assays each suggested that PvWOX3a can likely bind to the *PvCKX4b* promoter and repress its transcription (Figs. S2b, c and S3). These results together indicated that the downregulation of *PvCKX4b* is a potential contributing factor to the observed increase in cell number and vascular bundle formation in *PvWOX3a*-overexpressing transgenic plants.

In addition, we analyzed switchgrass cell wall deposition, including the contents of lignin, hemicellulose, and cellulose, since the vascular bundles were increased in WOX3aOE transgenic plants. We found that the acid-insoluble lignin contents of WOX3aOE transgenic lines were significantly higher than those of control plants (Fig. 3c). Moreover, the hemicellulose contents in WOX3aOE lines were also markedly higher than those in control plants (Fig. 3d), while the cellulose contents were comparable between control and WOX3aOE lines (Fig. 3e). These results thus suggested that increased vascular bundle formation resulting from *PvWOX3a* overexpression may have led to an increase in lignin and hemicellulose accumulation during stem development.

### Overexpression of *PvWOX3a* in switchgrass promoted cell elongation

**Fig. 4:**
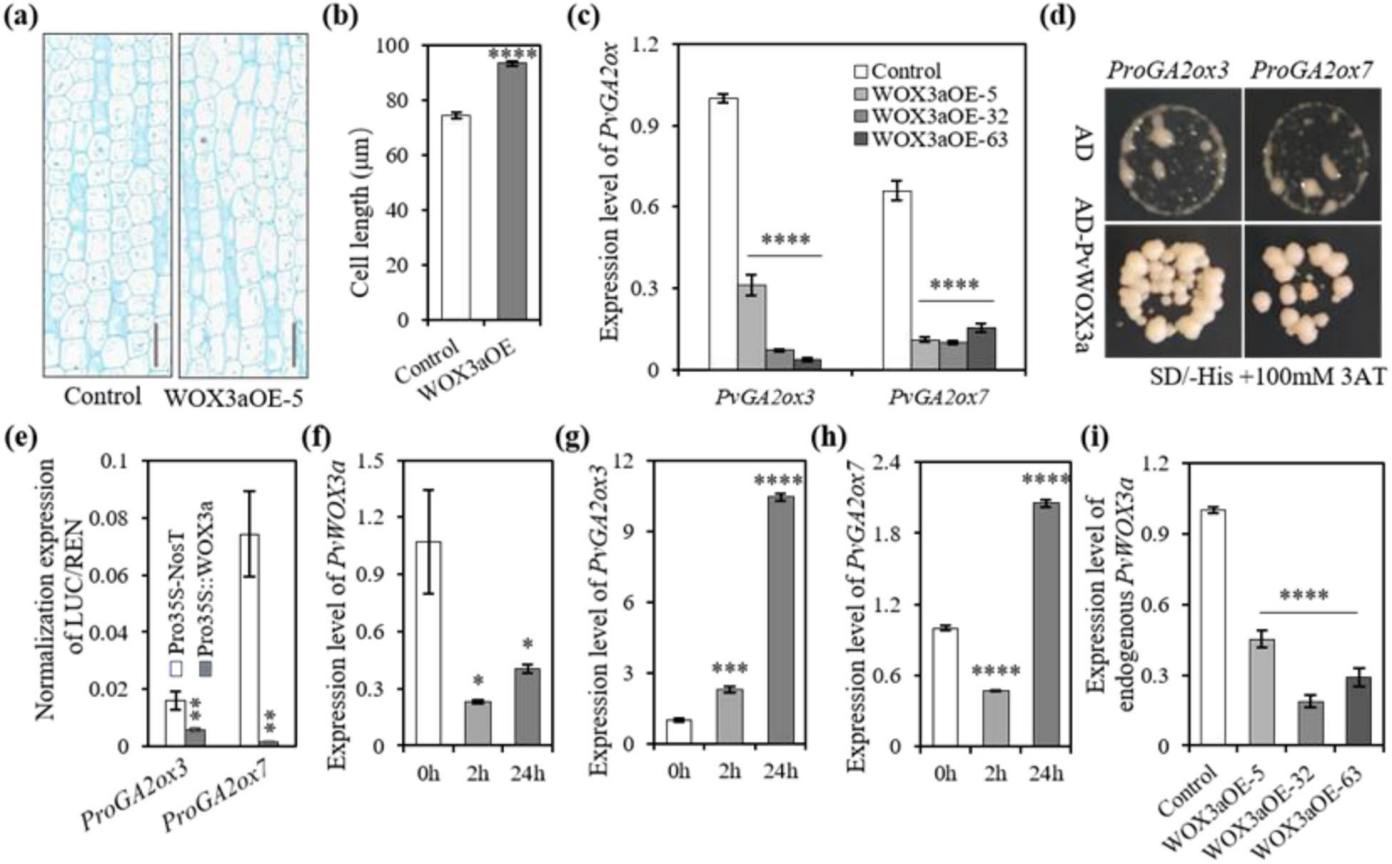
Overexpression of *PvWOX3a* downregulated *GA2ox* and promoted cell elongation in switchgrass. **a** Longitudinal section of Internode 2 at the E3 stage of control and WOX3aOE transgenic plants. Scale bar = 100 μm. **b** Cell lengths based on longitudinal sections of E3I2 of control and WOX3aOE transgenic plants. Values are means ± SEs (*n* = 18). **c** The expression levels of *PvGA2ox3* and *PvGA2ox7* in three WOX3aOE lines were revealed by qRT–PCR. Switchgrass *PvUbq2* was used for normalization. Values are means ± SEs (*n* = 3). **d** Growth of yeast cells on SD/-Trp-Leu-His supplemented with 100 mM 3-AT. pHIS2.1-*ProGA2ox3* plus pGADT7 and pHIS2.1-*ProGA2ox7* plus pGADT7 served as the negative controls. **e** Dual-luciferase assay showing the repression of *PvGA2ox3* and *PvGA2ox7* by the PvWOX3a effector construct compared to the control effector construct. Values are means ± SEs (*n* = 3). qRT–PCR analysis of *PvWOX3a* (**f**)*, PvGA2ox3* (**g**), and *PvGA2ox7* (**h**) expression levels in wild-type switchgrass plants treated with 200 μm GA_3_. Switchgrass *PvUbq2* was used for normalization. Values are means ± SEs (*n* = 3). **i** qRT–PCR analysis of endogenous *PvWOX3a* expression levels in control plants and WOX3aOE lines. Values are means ± SEs (*n* = 3). The asterisks represent significant differences in **b** and **e**, as determined by Student’s *t* test. ****p < 0.0001; **p < 0.0021. Asterisks represent significant differences in **c**, **f**, **g**, and **h** as determined by one-way ANOVA. *p < 0.0332; ***p < 0.0002; ****p < 0.0001

Internode elongation is mediated first by cell division followed by cell elongation. To further observe the cytological characteristics of the longer internodes observed in *PvWOX3a-*overexpressing switchgrass plants, we next examined cell number and cell size in longitudinal sections of I2 at the E3 stage. These observations showed that in WOX3aOE transgenic plants, the I2 cells were considerably longer than the same cells in control plants at this stage (~93 μm vs. ~74 μm average length, respectively) (Fig. 4a, b). These results together indicated that the observed increases in internode length and diameter in WOX3aOE plants were likely due to stimulated cell proliferation and cell elongation along the longitudinal axis.

### Overexpression of *PvWOX3a* in switchgrass reduced *GA2ox* expression levels

Gibberellin is known to perform essential hormone signaling functions in the regulation of longitudinal growth and cell elongation. To examine whether overexpression of *PvWOX3a* in switchgrass affected GA biosynthesis, catabolism, perception, and signaling, we analyzed the differentially expressed genes (DEGs) between the control and transgenic switchgrass plants by RNA-seq. In total, 4446 out of 85,522 (5.2%) genes exhibited significant differences (Log_2_FC < 0.5 for downregulated and Log_2_FC > 1 for upregulated DEGs) in their transcription levels between PvWOX3a-OE and control plants (Table S2). Among them, 2,136 genes were significantly upregulated, and 2,311 genes were downregulated. In addition, after filtering out genes with extremely low expression (FPKM < 1 across all samples), we conducted GO enrichment analysis on the resulting set of DEGs to identify the top 30 significantly enriched pathways (Fig. S4). Among these DEGs, the transcript abundances of *PvGA2ox3* and *PvGA2ox7* were significantly reduced in WOX3aOE transgenic plants (Table S2). Subsequent validation by qRT–PCR analysis confirmed the downregulation of *PvGA2ox3* and *PvGA2ox7* transcription in the WOX3aOE lines (Fig. 4c). In addition, the expression levels of genes encoding key enzymes in the GA pathway (including *CPS*, *KO1*, *KO2*, *KAO*, *GA20ox2*, and *GA3ox2*) were also validated by qRT–PCR. Consistent with RNA-seq analysis, none of these genes showed significant differences in expression between the control and WOX3aOE lines (Fig. S5).

### PvWOX3a interacted with the promoter sequences of *GA2ox*s and repressed their expression

To further test whether PvWOX3a can directly interact with putative downstream target genes in the GA catabolic pathway, such as *PvGA2ox3* and *PvGA2ox7*, we first examined 2.5 kb regions in the promoters and introns of potential target genes for the presence of well-established WOX recognition and binding motifs (i.e., TTAA motifs, CAAT motifs, CACGTG motifs, and TTAAT(G/C)^20–23^. This sequence analysis showed that the *PvGA2ox3* and *PvGA2ox7* promoter and intron regions contained at least 4 TTAA motifs and 12 CAAT motifs (Fig. S3). The TTAATCC motif was only found in the promoter of *PvGA2ox3* (Fig. S3). In addition, yeast one-hybrid assays further revealed that PvWOX3a apparently interacted with the promoter regions of both *PvGA2ox3* and *PvGA2ox7* (Fig. 4d). Moreover, the transient dual-luciferase assay showed that PvWOX3a significantly repressed luciferase activity driven by the *PvGA2ox3* and *PvGA2ox7* promoters (Fig. 4e). Taken together, our results suggested that PvWOX3a could potentially bind to the *PvGA2ox3* and *PvGA2ox7* promoter regions and repress their transcription.

To better understand the regulatory interactions between *PvWOX3*a and GA, we used exogenous applications of GA3 to wild-type plants to examine the effects of GA_3_ on *PvWOX3*a expression. The results showed that *PvWOX3a* transcription was rapidly but temporarily suppressed, and thus, the target genes *PvGA2ox3* and *PvGA2ox7* exhibited remarkable upregulation at 24 h after treatment (Fig. 4f, g, h). In agreement with our findings above, these results further suggested that PvWOX3a could indeed function as a transcriptional repressor of *PvGA2ox3* and *PvGA2ox7* expression. Moreover, we investigated whether signaling induced by exogenous GA affected native *PvWOX3a* expression using qRT–PCR-based measurement of endogenous *PvWOX3a* transcripts in WOX3aOE transgenic plants and control plants. Our results showed that the expression of endogenous *PvWOX3a* was suppressed in the WOX3aOE lines compared with that in control plants (Fig. 4i). Taken together, our results imply that *PvWOX3a* may regulate the homeostasis of GA concentration through a negative feedback loop in switchgrass.

### *PvWOX3a* overexpression in a miR156-overexpressing transgenic line led to increased biomass and soluble sugar yields

**Fig. 5:**
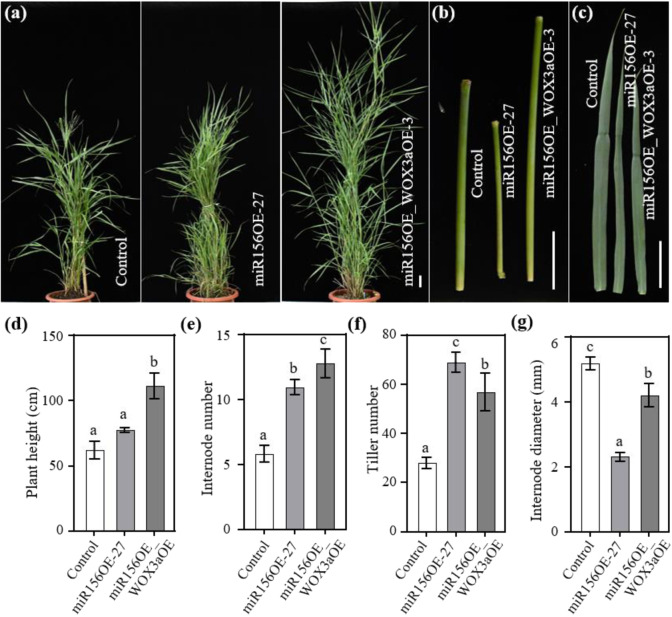
Overexpression of *PvWOX3a* in a miR156-overexpressing transgenic line. **a** Gross phenotypic characterization of control plants, miR156-overexpressing transgenic plants (miR156OE-27), and overexpression of *WOX3a* in miR156OE-27 transgenic plants (miR156OE_WOX3aOE). Scale bar = 5 cm. Internode 3 at the E4 stage (**b**) and Leaf 3 at the E4 stage (**c**) of control, transgenic miR156OE-27, and transgenic miR156OE_WOX3aOE plants are shown. Scale bar = 5 cm. Three-month-old tillers were used to measure plant height (**d**), internode number (**e**), tiller number (**f**), and internode diameter (**g**). Three tillers per plant were measured for each replicate. Values are means ± SEs (*n* = 3–7). The letters above the error bars indicate significant differences determined by one-way ANOVA (*p* < 0.05, Duncan’s multiple-range test)

Our previous studies showed that overexpression of miR156 in switchgrass can delay flowering time and lead to increased tiller number, resulting in improved biomass yield. However, the short and thin internode phenotype caused by miR156 overexpression can restrict further improvement to the biomass yield of switchgrass^26^. We therefore used a miR156-overexpressing transgenic line, miR156OE-27, which exhibited increased tiller and internode numbers but also had strikingly thin and short internodes, for our further characterization of the effects of PvWOX3a on the switchgrass phenotype. To this end, we overexpressed *PvWOX3a* in the miR156OE line to identify alterations to the thin and short internode phenotype exhibited by these plants. Double transgenic positive plants were screened by genomic PCR, and the relative expression levels of *PvWOX3a* and miR156 were then confirmed by qRT–PCR (Fig. S6a, b). Three double transgenic lines, miR156OE_WOX3aOE-3, −8, −12, which had considerably high levels of both miR156 and *PvWOX3a* transcripts (Fig. S6a, b), were selected for further morphological analysis. At the flowering initiation stage, we measured plant height, internode diameter, tiller number, and flowering time for the control plants, miR156OE-27 plants, and the double transgenic lines (Fig. 5a and Table S1). The results showed that the plant heights of the double transgenic lines were dramatically higher than those of both the control and the miR156OE27 lines (~50 cm) due to the substantially elongated internodes (Fig. 5a, b, d). Excitingly, we observed that the double transgenic lines retained the increased internode number displayed by the miR156OE line (Figs. 5e and S7). Moreover, the tiller number of the double transgenic lines was slightly lower than that of the miR156OE line but still significantly higher than that of the controls (Fig. 5f), potentially due to the reduced downregulation in miR156 in double transgenic lines compared with miR156OE-27. The stems of double transgenic plants were thinner than those of control plants but were significantly thicker than those of the miR156OE line (Fig. 5g). Furthermore, the leaf blade width of the double transgenic line was restored to that of the controls, although leaf blade length remained significantly shorter than that of control plants (Fig. 5c). In addition, the double transgenic plants exhibited a longer flowering time (by ~1 month) than the controls (Table S1).

**Fig. 6:**
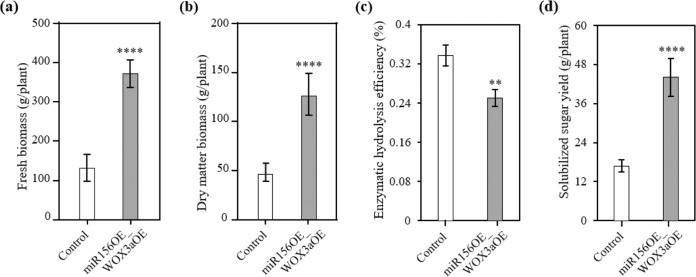
The double transgenic lines showed higher biomass and solubilized sugar yields than the control plants. Comparison of postharvest fresh (**a**) and dry (**b**) weights of total above-ground biomass of control and miR156OE_WOX3aOE lines after four months of growth in the greenhouse. Values are means ± SEs (*n* = 4–9). Enzymatic hydrolysis efficiency (**c**) and solubilized sugar yields (**d**) of three miR156OE_WOX3aOE independent lines compared to control plants. Values are means ± SEs (*n* = 3). The asterisks represent significant differences determined by Student’s *t* test. **p < 0.0021; ****p < 0.0001

Most notably, the double transgenic lines showed a 184% increase in fresh weight biomass and a 174% increase in average dry-weight biomass compared with the control plants (Fig. 6a, b). The total solubilized sugars were determined by combining the biomass yield and saccharification efficiency. Despite the increased lignin content and reduced saccharification efficiency (Fig. 6c), the double transgenic lines produced 162% more total sugar on average than the control plants due to the high biomass yield (Fig. 6d). Taken together, overexpression of *PvWOX3a* in the miR156-overexpressing transgenic line led to increased biomass and soluble sugar yields.

## Discussion

The size of ornamental grasses, as the first consideration for most gardeners, is usually determined by plant height, stem diameter, and tiller number. Switchgrass is an attractive medium to tall perennial ornamental bunchgrass that can be employed to divide a garden into distinct sections and accentuate the lines of the landscape. Stem development affects the shoot architecture, biomass, and lodging resistance of crop plants, and the manipulation of stem development has been proposed as an effective strategy to improve biomass production in switchgrass^7,25,26^. WOX3 has been established as a major regulator of lateral organ development^21,23,25,42^. However, the mechanism and function of WOX3 in modulating stem development have remained unclear. In this study, we investigated these potential mechanisms through overexpression of *PvWOX3a* in switchgrass. PvWOX3a can repress the transcription of *PvCKX4b*, and its transgenic overexpression also led to increased stem diameter, leaf width, and biomass yield. Overexpression of *PvWOX3a* increased internode length and diameter and plant height in switchgrass. Further analysis suggested that PvWOX3a can interact with the promoter region of *GA2ox* and repress its expression in transgenic switchgrass plants. We ultimately determined that the overexpression of *PvWOX3a* alone or with miR156 in a double transgenic overexpression switchgrass line yielded up to 135% and 223% more dry-weight biomass, respectively. Our data further indicated that *PvWOX3a* acts in the regulation of stem development and improves shoot architecture and biomass yield by coordinating GA and CK homeostasis in switchgrass.

Previous studies have shown that WOX1 and WOX3 act redundantly in regulating leaf blade morphology in dicot species^42^. However, in monocot species, WOX1 homologs were possibly lost, while WOX3 family members proliferated by duplication^19^. Overexpression of the *Medicago STF* gene in switchgrass, rice, and *Brachypodium* results in the repression of *CKX* gene expression, increased CK content in leaves and stems, and significantly higher plant biomass^8^. Interestingly, the overexpression of *PvWOX3a* repressed *GA2ox* transcription, which potentially increased the levels of bioactive GA, consequently stimulating cell division and elongation at the internodes. Previous studies exploring the overexpression of *OsWOX3a* in rice reported a severe dwarf phenotype with wider leaves than wild type, regardless of how much *OsWOX3a* transcripts increased, whereas the height of *nal2/3* mutants was similar to that of wild-type rice with narrower and thinner leaves and stems^21,22,24^.

Overexpression of *OsWOX3a* may directly repress the expression of *KAO*, which encodes a key enzyme in the biosynthetic pathway for bioactive GA, thus reducing the levels of endogenous GA intermediates and ultimately resulting in a dwarf architecture in rice^22^. However, we detected no alterations in the expression of *KAO* in *PvWOX3a*-overexpressing transgenic switchgrass plants, suggesting that the PvWOX3a-*KAO* regulation module appears to be absent from switchgrass. In addition, *PvWOX3a* transcription in internodes was repressed under treatments with exogenous GA_3_ in switchgrass, while *OsWOX3a* levels in rice seedlings increased under exposure to GA. These results suggest that WOX3 may have differing regulatory functions in internode development in different monocot species. However, future work exploring the nature of interactions between PvWOX3a and the *GA2ox* promoter region may reveal how GA signaling induces different transcriptional patterns between species.

In switchgrass, silencing *PvGA2ox* and overexpressing *ZmGA20ox* both led to increased bioactive GA content, as well as higher lignin content^7,9,43^. Interestingly, increased lignin and hemicellulose contents were also observed in our switchgrass WOX3aOE lines. However, lignin deposition did not significantly differ, and even showed a slight decrease, in *STF*-overexpressing transgenic switchgrass compared to wild-type switchgrass. We speculate that this difference between *PvWOX3a* and *STF* overexpression is also related to abnormal GA levels. These results are consistent with previous reports showing that GA accumulation can enhance lignin biosynthesis in plants^44^. Lignin has been described as a negative factor impacting the conversion efficiency of lignocellulosic biomass. However, simultaneous downregulation of lignin biosynthetic genes such as *PAL*, *CAD*, and *COMT* in the WOX3aOE background could further improve the quality of transgenic switchgrass plants.

The miR156-SPL module has been established as a master regulator of vegetative phase transition and regulates several developmental processes through coordination of several classes of phytohormones, such as GA, CK, auxin, jasmonate, and strigolactone^45–49^. For instance, GA and miR156 induce complex crosstalk among signals for floral transition and axillary bud formation through interactions between the DELLA and SPL transcription factors. The miR156-mediated flowering pathway has interplay with the GA-induced flowering pathway owing to DELLA protein-mediated inhibition of SPL expression or through protein–protein interactions^50^. In contrast, in the axillary bud formation interaction network, DELLA was shown to bind to SPL9 and attenuate the repression of *LATERAL SUPPRESSOR* (*LAS*), which promotes the initiation of axillary buds^51^. Previous studies have suggested that overexpression of miR156 in switchgrass can delay the juvenile-to-adult phase transition, prevent flowering, increase the tiller number, improve biomass yield, and enhance cell wall digestibility and starch content^26,52^. However, the transgenic switchgrass plants highly overexpressing miR156 also exhibit a reduced internode length and diameter, which impairs their ornamental characteristics in garden and landscape design and limits further improvement of biomass yield for forage and bioenergy production^26,52^.

Since overexpression of *PvWOX3a* drives an increase in stem length and diameter, we also overexpressed *PvWOX3a* in a miR156-overexpressing transgenic switchgrass line to test whether *PvWOX3a* can rescue its short internode phenotype. We observed a significant, surprising increase in internode length and diameter and leaf blade width in the double transgenic switchgrass plants, while there was no change in the tiller number compared with transgenic plants that overexpressed only miR156. Most interestingly, the double transgenic switchgrass plants also had a higher internode number than the miR156OE-27 transgenic line primarily due to elongation of the top stunted internode, which macroscopically revealed stunted nodes that were too small to be detected in the miR156-overexpressing plants. These results further indicated that WOX3a can promote internode elongation and that WOX3a, miR156, and GA comprise overlapping networks that may compete under different conditions to control internode architecture. Future metabolomics analysis will determine how hormone levels, such as those for GA and CK, are differently affected by the overexpression of *PvWOX3a* and miR156, either individually or together, in switchgrass.

Plant height, tiller number, internode/stem length, and thickness are the main effects of switchgrass morphology and biomass yield. Numerous factors affecting stem development have been applied to gain increased plant height, biomass, and vegetative yields of switchgrass in the active and innovative breeding programs of horticultural, forage, and bioenergy crops. In conclusion, our work suggests that PvWOX3a potentially affects the contents of both GA and CK via repression of *GA2ox* and *CKX4b* transcription and promotes cell elongation and division in the internodes/stems of switchgrass. Moreover, we found that overexpression of *PvWOX3a* leads to elongation of the internodes in a miR156-overexpression background in switchgrass. Most strikingly, the double transgenic switchgrass plants exhibited a bushy morphology and further improved plant height and biomass yield, which are excellent traits for garden and landscape ornamental plants and forage and bioenergy production. Taken together, our results uncover a previously undescribed mechanism and function of PvWOX3a in regulating switchgrass stem development and show that PvWOX3a is a viable target for improving the shoot architecture and biomass yield of horticulture, fodder, and biofuel crops.

## Supplementary information

Supporting figures S1-7

Supporting tables S1-3

## Data Availability

The data that support the findings of this study are available from the corresponding author upon reasonable request.
