# Supplementary material for: Overexpression of PvWOX3a in switchgrass promotes stem development and increases plant height
Source: Hortic Res. 2021 Dec 1;8:252. doi: 10.1038/s41438-021-00678-w (PMC8633294; doi:10.1038/s41438-021-00678-w)
Supplement: Supplementary file 1 — Supporting figures S1-7 [file 41438_2021_678_MOESM1_ESM.pdf]

Overexpression of *PvWOX3a* in switchgrass promotes stem development and improves biomass yield

Supporting Information (Fig. S1-7)

Fig. S1. Conserved amino acid domain analysis of AtWOX3, OsWOX3a, and PvWOX3a.

Fig. S2. PvWOX3a binds to the promoter of *PvCKX4b*. (a) Expression levels of *PvCKX4b* in three WOX3aOE transgenic plants were determined by qRT-PCR. Switchgrass *PvUbq2* was used for normalization. Values are means ± SE (n=3). Asterisks represent significant differences determined by one-way ANOVA. ****, $p < 0.0001$. (b) Growth of yeast cells on SD/-Trp-Leu-His supplemented with 100 mM 3-AT. pHIS2.1-*PvCKX4b* plus pGADT7 served as the negative control. (c) Dual luciferase assay showing the repression of *PvCKX4b* by the PvWOX3a effector construct compared to the control effector construct. Values are means ± SE (n=3). Asterisks represent significant differences determined by Student's t test. ***, $p < 0.0002$.

Fig. S3. The locations of the PvWOX3a binding motifs within the promoter regions and intron regions of *PvGA2ox3, PvGA2ox7*, and *PvCKX4b*.

Fig. S4. GO enrichment analysis of differentially expressed genes (DEGs) between control plants and WOX3aOE transgenic plants. The 30 most highly enriched GO pathways are listed.

Fig. S5. qRT-PCR analysis of GA pathways gene expression levels in control and transgenic WOX3aOE plants. Switchgrass *PvUbq2* was used for normalization. Values are means ± SE (n=3).

Fig. S6. qRT-PCR analysis of *PvWOX3a* (a) and mature miR156 (b) expression levels in control, transgenic miR156OE-27, and double transgene miR156OE_WOX3aOE plants. Switchgrass *PvUbq2* was used for normalization. Values are means ± SE (n=3). The letters above error bars indicate significant differences determined by one-way ANOVA ($p < 0.05$, Duncan's multiple-range test).

Fig. S7. Morphological characterization of internode number for control, miR156OE-27, and miR156OE_WOX3aOE plants.

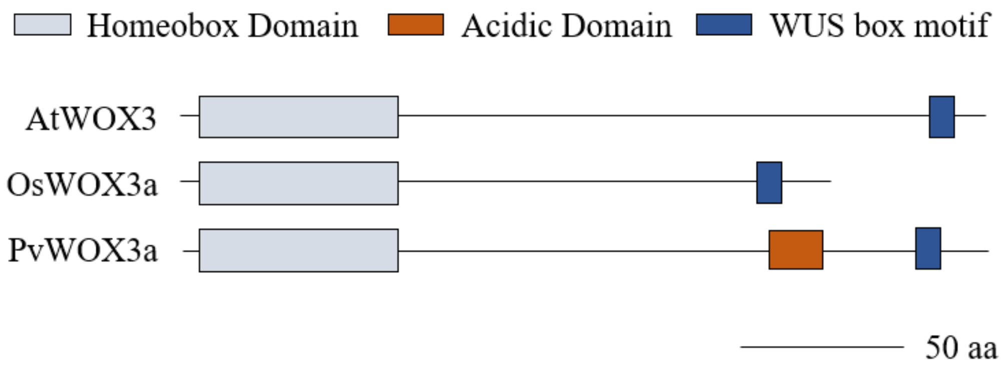

Fig. S1. Conserved amino acid domain analysis of AtWOX3, OsWOX3a, and PvWOX3a.

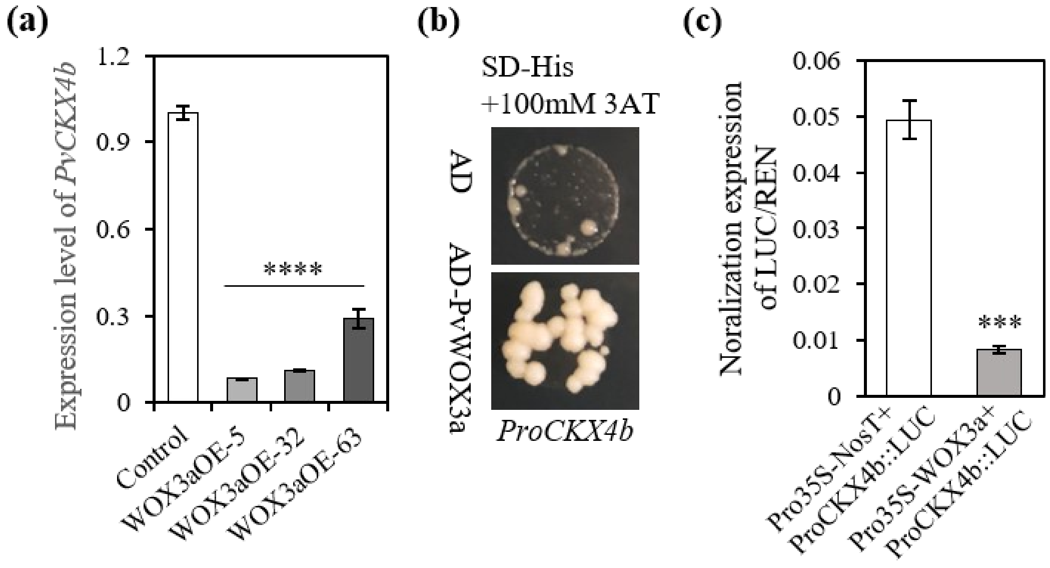

Fig. S2. PvWOX3a binds to the promoter of *PvCKX4b*. (a) Expression levels of *PvCKX4b* in three WOX3aOE transgenic plants were determined by qRT-PCR. Switchgrass *PvUbq2* was used for normalization. Values are means ± SE (n=3). Asterisks represent significant differences determined by one-way ANOVA. ****, $p < 0.0001$. (b) Growth of yeast cells on SD/-Trp-Leu-His supplemented with 100 mM 3-AT. pHIS2.1-*PvCKX4b* plus pGADT7 served as the negative control. (c) Dual luciferase assay showing the repression of *PvCKX4b* by the PvWOX3a effector construct compared to the control effector construct. Values are means ± SE (n=3). Asterisks represent significant differences determined by Student's t test. ***, $p < 0.0002$.

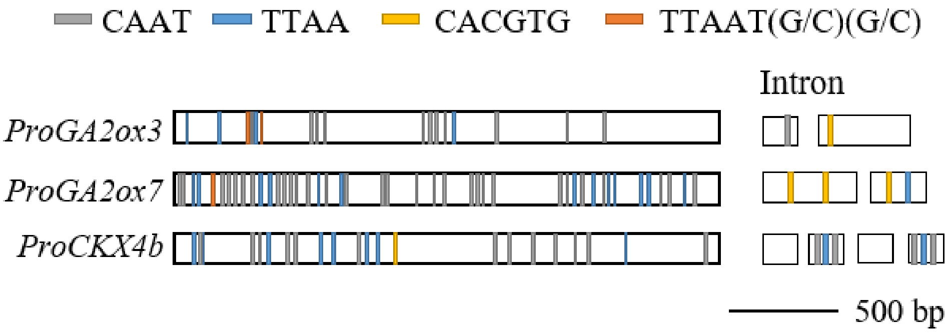

Fig. S3. he locations of the PvWOX3a binding motifs within the promoter regions and intron regions of *PvGA2ox3, PvGA2ox7*, and *PvCKX4b*.

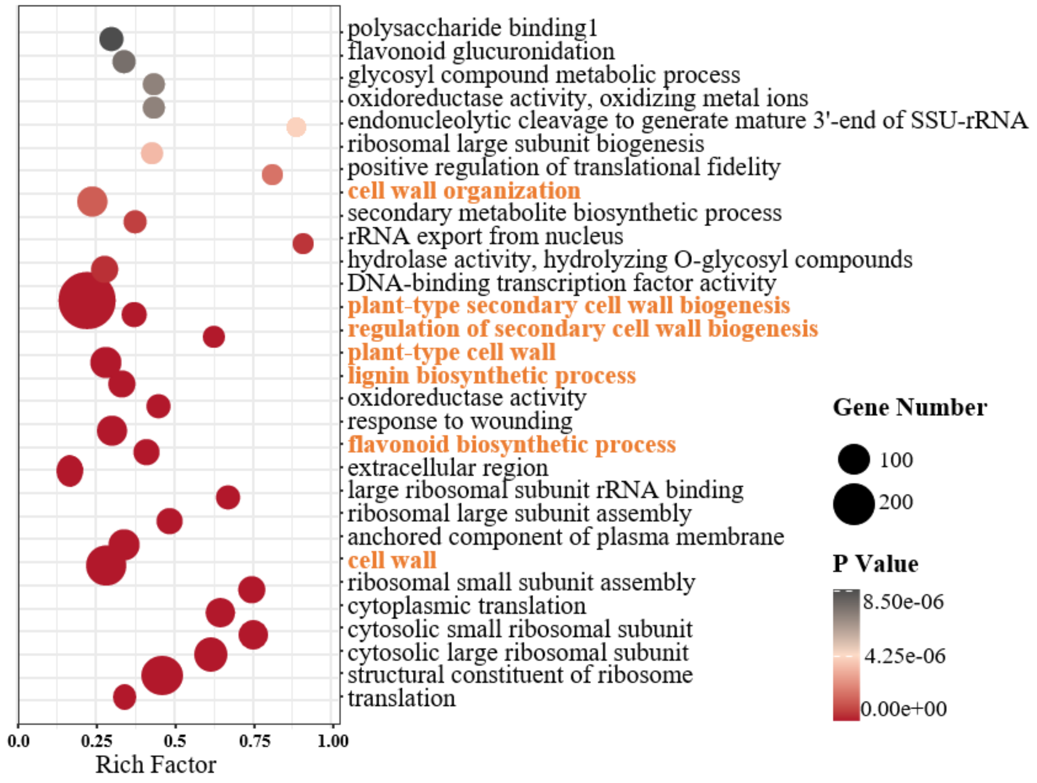

Fig. S4. GO enrichment analysis of differentially expressed genes (DEGs) between control plants and WOX3aOE transgenic plants. The 30 most highly enriched GO pathways are listed.

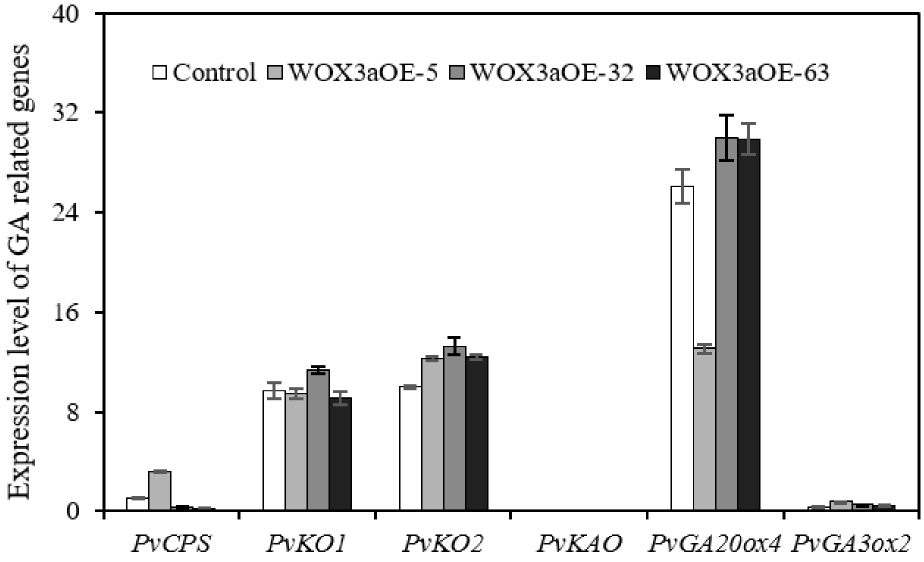

Fig. S5. qRT-PCR analysis of GA pathways gene expression levels in control and transgenic WOX3aOE plants. Switchgrass *PvUbq2* was used for normalization. Values are means ± SE (n=3).

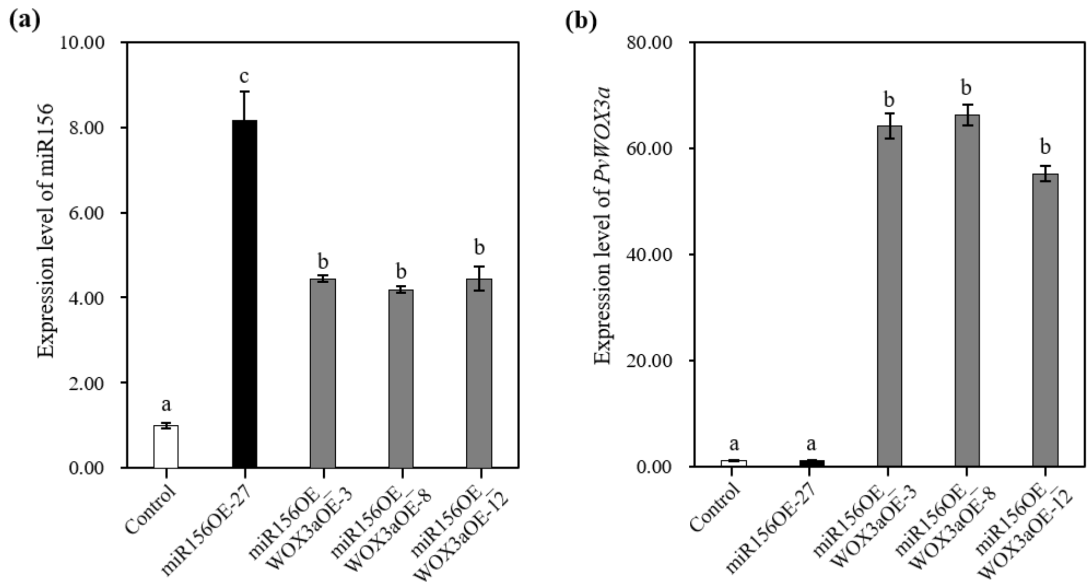

Fig. S6. qRT-PCR analysis of *PvWOX3a* (a) and mature miR156 (b) expression levels in control, transgenic miR156OE-27, and double transgene miR156OE_WOX3aOE plants. Switchgrass *PvUbq2* was used for normalization. Values are means ± SE (n=3). The letters above error bars indicate significant differences determined by one-way ANOVA (p < 0.05, Duncan's multiple-range test).

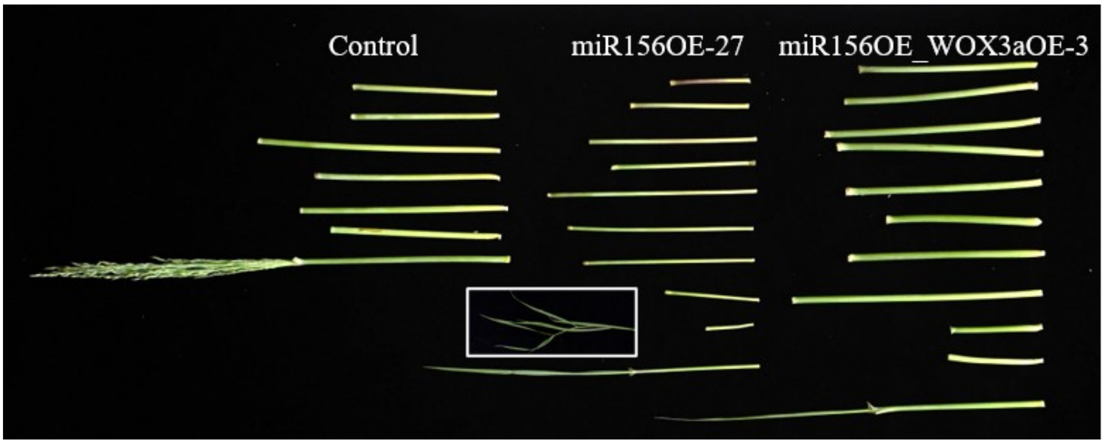

Fig. S7. Morphological characterization of internode number for control, miR156OE-27, and miR156OE_WOX3aOE plants.
