# Supplementary material for: Overexpression of PvWOX3a in switchgrass promotes stem development and increases plant height
Source: Hortic Res. 2021 Dec 1;8:252. doi: 10.1038/s41438-021-00678-w (PMC8633294; doi:10.1038/s41438-021-00678-w)
Supplement: Supplementary file 2 — Supporting tables S1-3 [file 41438_2021_678_MOESM2_ESM.pdf]

Table S1. Tiller number and flowering time in transgenic plants.

	Tiller number	Flowering time (day)
Control plants	27.67±1.45	92.44±1.39
WOX3aOE	27±0.58	92.56±1.45
miR156OE-27	68.67±2.33****	>130
WOX3aOE_miR156OE	57±3.51****	127.56±1.89****

The 3-month-old tillers were used to measure tiller number. Flowering time of switchgrass was measure before harvest.Values are mean ± SE (n=3). The aserisks represent significant differences determined by one-way ANOVA. ****, $p < 0.0001$.

Table S2. Genes differentially expressed in PvWOX3a overexpressing transgenic plants.

2311 down-regulated genes and 2136 up-regulated genes were shown in this table

gene_id	GO	KEGG	Description	FPKM.WO	FPKM.WO	FPKM.Ctrl	FPKM.Ctrl	fc	log2(fc)	pval	qval	regula	signific	swissprot_I	swissprot_name
Pavir.9KG417100.v	NA	NA	0	59.452957	98.525452	0.0001	0.0001	789892.05	19.591296	1.92611E-20	3.61848E-16	up	yes	NA	NA
Pavir.9KG597300.v	NA	NA	0	10.140471	42.575314	0.0001	0.0001	263578.93	18.007875	1.40772E-12	2.64461E-09	up	yes	NA	NA
Pavir.5NG411600.v	GO:000983	00514(Othe	galactosylga	26.245012	14.981599	0.0001	0.0001	206133.06	17.653216	7.44306E-22	2.79658E-17	up	yes	Probable be	IRX9L
Pavir.5KG083200.v	NA	NA	0	1.545063	27.976362	0.0001	0.0001	147607.13	17.171403	2.54974E-09	1.34931E-06	up	yes	NA	NA
Pavir.5KG222700.v	GO:000573	00073(Cuti	transferase f	1.03446	19.561344	0.0001	0.0001	102979.02	16.651991	1.02643E-10	8.76505E-08	up	yes	Acyl transfe	AT4
Pavir.7NG045600.v	NA	NA	0	11.994706	7.60467	0.0001	0.0001	97996.88	16.580448	2.65393E-16	1.26136E-12	up	yes	NA	NA
Pavir.5KG762100.v	GO:000436	00480(Glut	glutathione	2.292782	11.22545	0.0001	0.0001	67591.16	16.044547	1.31545E-09	7.84528E-07	up	yes	Glutathione	-
Pavir.6NG272000.v	NA	NA	0	7.282694	3.967573	0.0001	0.0001	56251.335	15.7796	9.82185E-09	3.92592E-06	up	yes	NA	NA
Pavir.8NG292400.v	NA	NA	0	7.488783	1.862189	0.0001	0.0001	46754.86	15.512829	1.47707E-08	5.33633E-06	up	yes	NA	NA
Pavir.9NG352500.v	NA	NA	0	4.061141	4.928474	0.0001	0.0001	44948.075	15.455972	1.33974E-08	4.93512E-06	up	yes	NA	NA
Pavir.5KG776400.v	GO:000417	NA	aspartyl ami	2.554522	5.004663	0.0001	0.0001	37795.925	15.205943	1.80937E-11	1.88843E-08	up	yes	Probable as	RCOM_1506700
Pavir.7KG000400.v	NA	NA	0	3.340431	3.750947	0.0001	0.0001	35456.89	15.113778	2.85634E-07	6.31301E-05	up	yes	NA	NA
Pavir.5KG773000.v	GO:000667	NA	ceramidase	3.089665	2.799566	0.0001	0.0001	29446.155	14.845792	5.48309E-09	2.49895E-06	up	yes	NA	NA
Pavir.4NG240700.v	GO:000588	NA	potassium tr	1.989411	3.880516	0.0001	0.0001	29349.635	14.841055	2.81002E-12	4.79913E-09	up	yes	Potassium t	HAK10
Pavir.9KG579900.v	GO:000815	00500(Starc	ppr repeat (	1.357056	1.886861	0.0001	0.0001	16219.585	13.985449	2.3908E-10	1.87144E-07	up	yes	Pentatricop	PCMP-H27
Pavir.5NG051800.v	GO:000400	03040(Splic	atp-depende	1.237915	1.709041	0.0001	0.0001	14734.78	13.846938	5.07337E-09	2.38277E-06	up	yes	DEAD-box	Os01g0197200
Pavir.4NG328100.v	GO:000367	04075(Plan	camp-respo	1.0350989	1.7713304	0.0001	0.0001	14032.146	13.776448	5.28727E-08	1.56424E-05	up	yes	Protein FD	FD;FD;FD
Pavir.1KG317200.v	NA	NA	0	1.12041	1.508599	0.0001	0.0001	13145.045	13.682231	2.07004E-08	7.00698E-06	up	yes	NA	NA
**Pavir.8KG017200.v**	**GO:00055**	**00190(Oxid**	**wuschel-rel**	**161.19339**	**373.26334**	**0.0001**	**0.130676**	**4086.8105**	**11.99676**	**2.36016E-13**	**5.91188E-10**	**up**	**yes**	**WUSCHEL**	**WOX3A**
Pavir.2KG535100.v	GO:000969	NA	dirigent pro	1.789788	41.016468	0.0001	0.030624	1393.2514	10.44424	7.75889E-09	3.23917E-06	up	yes	Dirigent pro	DIR5
Pavir.5KG768500.v	GO:000557	00514(Othe	glycosyltran	1.083748	8.191236	0.014661	0.0001	628.34388	9.2954105	4.56914E-08	1.40718E-05	up	yes	Xylan glyco	MUCI21
Pavir.5KG764800.v	GO:000563	03040(Splic	sulfotransfe	27.273813	7.520975	0.070328	0.029917	347.09749	8.4391971	7.78851E-13	1.5991E-09	up	yes	Cytosolic su	SOT5
Pavir.5KG688800.v	GO:000588	NA	probable lip	64.457573	3.241267	0.0001	0.232106	291.54647	8.1875821	1.97778E-06	0.00032881	up	yes	NA	NA
Pavir.4KG061000.v	GO:000032	NA	burp domain	2.401298	82.86747	0.079623	0.254937	254.86839	7.9936086	2.64971E-07	5.92604E-05	up	yes	BURP dom	BURP6;BURP6
Pavir.5KG045800.v	NA	NA	0	1.9885517	41.267857	0.0001	0.177125	244.07622	7.9311879	4.97299E-06	0.000744423	up	yes	Metallothio	MT3B;MT3A
Pavir.5KG761700.v	NA	NA	0	9.2551901	14.995722	0.122763	0.0001	197.38171	7.6248445	1.0241E-08	4.05035E-06	up	yes	NA	NA
Pavir.6KG292800.v	GO:000817	00945(Stilb	o-methyltran	2.911777	5.597317	0.053735	0.0001	158.05877	7.3043173	2.35741E-07	5.40092E-05	up	yes	Acetylserot	ASMT3
Pavir.6NG324800.v	GO:000563	NA	alcohol deh	1.701959	77.431297	0.037384	0.469956	155.97677	7.2851874	1.43741E-05	0.001836998	up	yes	2-alkenal re	DBR
Pavir.1NG049200.v	GO:000367	03013(RNA	cold shock	23.596362	19.334658	0.2493973	0.0334868	151.7619	7.2456658	3.11915E-11	3.0841E-08	up	yes	Glycine-rich	GRP-2;GRP-2;GRP-2
Pavir.7KG053700.v	GO:000393	NA	inosine-5-m	2.909015	18.879091	0.0001	0.182233	119.49623	6.9008213	7.9635E-06	0.00112486	up	yes	NA	NA
Pavir.2KG525700.v	GO:000727	NA	dextranase /	67.623684	3.185176	0.0001	0.6474138	109.35498	6.7728752	0.000139745	0.010945123	up	yes	NA	NA
Pavir.2NG625800.v	GO:000557	00860(Porp	zinc finger f	48.432617	1.437534	0.03069	0.429928	108.26792	6.758462	2.90261E-05	0.003278573	up	yes	GDSL ester	At2g23540
Pavir.7NG111800.v	GO:000588	NA	probable lip	59.012188	1.442786	0.056217	0.529691	103.18168	6.6890431	3.20828E-05	0.003546271	up	yes	NA	NA
Pavir.1KG133100.v	NA	NA	0	6.949437	1.925079	0.0001	0.087295	101.54489	6.6659739	2.46075E-05	0.002900104	up	yes	NA	NA
Pavir.1KG527200.v	GO:000097	00190(Oxid	myb-like dn	3.003386	2.122693	0.0001	0.051189	99.944998	6.6430625	3.45381E-06	0.000547552	up	yes	Transcriptio	MYB36
Pavir.2KG047800.v	NA	NA	0	91.258568	3.078885	0.0001	0.966223	97.625176	6.6091813	0.000345477	0.021526683	up	yes	NA	NA
Pavir.2KG518400.v	NA	NA	0	69.705048	2.828016	0.097834	0.648667	97.164055	6.6023508	8.23638E-06	0.001159047	up	yes	NA	NA
Pavir.5NG049000.v	NA	NA	0	1.76761	76.428452	0.0001	0.812811	96.192648	6.5878547	0.000464963	0.026835728	up	yes	Metallothio	MT3B
Pavir.8NG297000.v	NA	NA	dvl family (	11.840407	1.90717	0.0001	0.144063	95.361341	6.5753326	3.08999E-05	0.003445106	up	yes	NA	NA
Pavir.4NG241300.v	GO:000588	NA	potassium tr	2.094632	4.176663	0.0001	0.065948	94.950566	6.5691047	2.58978E-06	0.000421238	up	yes	Potassium t	HAK10
Pavir.9NG719300.v	GO:000551	04626(Plan	non-specific	1.164076	5.569713	0.0001	0.072618	92.601405	6.5329622	2.15453E-05	0.002611357	up	yes	Serine/threo	RIPK
Pavir.2KG249400.v	GO:000425	04144(Endo	proprotein c	9.723693	8.538114	0.07208	0.134451	88.421627	6.4663274	1.3128E-11	1.59116E-08	up	yes	CO(2)-resp	CRSP
Pavir.9KG158800.v	GO:000449	00073(Cuti	fatty acid or	29.891253	1.457183	0.0001	0.35714	87.751752	6.455356	0.00032072	0.020424429	up	yes	Cytochrome	CYP86B1
Pavir.7KG196700.v	NA	NA	0	3.321406	2.808231	0.0001	0.071078	86.117016	6.4282264	1.43686E-05	0.001836998	up	yes	NA	NA
Pavir.5NG569300.v	GO:000550	00053(Asco	laccase-12-r	30.89377	1.434576	0.0001	0.382208	84.560998	6.4019205	0.000379593	0.023003977	up	yes	Laccase-3 (	LAC3
Pavir.4NG160000.v	GO:000460	00940(Phen	peroxidase	40.886269	1.03397	0.053809	0.447239	83.665116	6.3865543	4.9663E-05	0.005112293	up	yes	Peroxidase	PER11
Pavir.5KG030900.v	NA	NA	0	1.992045	29.829693	0.061517	0.328275	81.63774	6.3511643	1.11324E-05	0.00148326	up	yes	NA	NA
Pavir.5KG610700.v	GO:000550	00053(Asco	laccase-12-r	25.011906	1.287344	0.0001	0.330304	79.597251	6.3146467	0.000369412	0.022532345	up	yes	Laccase-3 (	LAC3
Pavir.9KG637900.v	GO:004845	00750(Vita	homeobox c	2.168047	62.34845	0.0001	0.822607	78.419774	6.2931456	0.000817236	0.040349566	up	yes	Homeobox-	HOX12
Pavir.2KG572000.v	GO:000557	00860(Porp	zinc finger f	22.391449	1.513896	0.0001	0.308495	77.465108	6.2754747	0.00023552	0.016060248	up	yes	GDSL ester	At2g23540
Pavir.2NG578000.v	GO:000382	00941(Flav	nad depende	1.4778321	6.042019	0.0001	0.097688	76.899528	6.2649028	2.96478E-05	0.003325245	up	yes	Putative ant	-;-
Pavir.5KG171800.v	GO:000460	00940(Phen	peroxidase	3.059846	9.251431	0.0001	0.162517	75.707195	6.2423585	1.97412E-05	0.002428851	up	yes	Peroxidase	PER15
Pavir.7NG010200.v	NA	NA	0	3.164284	1.729207	0.032078	0.033977	74.082068	6.2110525	3.73687E-07	7.84389E-05	up	yes	NA	NA
Pavir.9NG172400.v	GO:000588	NA	probable lip	38.783264	3.258632	0.0001	0.569982	73.747103	6.2045145	0.00020873	0.014647483	up	yes	NA	NA
Pavir.5KG750300.v	GO:004316	NA	ring finger c	2.793091	2.559509	0.0001	0.078127	68.423946	6.0964294	2.14305E-05	0.002605852	up	yes	NA	NA
Pavir.9NG692600.v	GO:000588	02010(ABC	abc transpo	17.734163	1.491318	0.021966	0.267512	66.414308	6.0534222	1.87504E-05	0.002332812	up	yes	ABC transp	RCN1
Pavir.4KG214200.v	GO:000557	00500(Starc	zinc finger f	0.3420655	89.797678	0.0001	1.4152601	63.686789	5.9929222	0.008022287	0.162733919	up	yes	GDSL ester	At2g23540;At2g23540;At1g71250;At2g23540
Pavir.6NG190700.v	NA	NA	auxin-induc	11.464468	1.405127	0.0001	0.211077	60.942219	5.9293701	0.000225591	0.015495689	up	yes	NA	NA
Pavir.2KG047700.v	NA	NA	0	95.561554	1.518751	0.0001	1.604646	60.495745	5.9187618	0.003131368	0.096359443	up	yes	NA	NA
Pavir.1NG450700.v	GO:001678	NA	alpha/beta h	31.142332	1.015323	0.0001	0.560425	57.370599	5.8422397	0.001564357	0.062067139	up	yes	NA	NA
Pavir.4NG016500.v	GO:000078	NA	histone h1	10.58021	142.21582	0.88232	1.884125	55.231905	5.78743	5.41328E-06	0.000794505	up	yes	Histone H1	-;-
Pavir.4KG203000.v	GO:000557	00860(Porp	zinc finger f	23.447273	1.098136	0.0001	0.44667	54.939698	5.7797771	0.001109961	0.04941299	up	yes	GDSL ester	At1g74460
Pavir.4KG264600.v	GO:000990	04712(Circ	phosphatidy	1.62481	9.225426	0.0001	0.197992	54.773721	5.775412	0.000239614	0.016280287	up	yes	Protein RIC	RFT1
Pavir.2KG525900.v	NA	NA	0	63.642136	3.791563	0.047548	1.201806	53.974853	5.7542155	0.000182196	0.013190107	up	yes	NA	NA
Pavir.7NG215000.v	NA	NA	0	1.711566	20.283379	0.13341	0.274159	53.966187	5.7539839	1.93512E-05	0.002391717	up	yes	NA	NA
Pavir.8KG121300.v	GO:000573	00073(Cuti	acyltransfer	40.817322	4.55915	0.109708	0.798786	49.946914	5.6423236	9.88229E-06	0.0013651	up	yes	Omega-hyd	HHT1
Pavir.7NG302300.v	GO:000367	NA	protein spir	3.28783	74.761337	0.0001	1.635174	47.728495	5.5767789	0.002080208	0.073868681	up	yes	Protein SPI	Os04g0578300
Pavir.9KG055100.v	GO:000969	00270(Cyst	1-aminocycl	11.123075	65.031441	0.280445	1.33172	47.23742	5.5618583	1.82801E-06	0.000305261	up	yes	1-aminocyc	ACO1
Pavir.7NG302200.v	GO:000367	NA	uncharacteri	1.527429	4.423368	0.0001	0.127216	46.74037	5.5465973	0.000214858	0.014894566	up	yes	NA	NA
Pavir.9KG485000.v	GO:000588	02010(ABC	abc transpo	18.09083	1.0068	0.0001	0.41576	45.923219	5.5211519	0.001787376	0.067630484	up	yes	ABC transp	RCN1
Pavir.2NG206400.v	GO:000561	NA	germin-like	1.112112	1.684849	0.0001	0.06112	45.687047	5.5137133	0.000118169	0.00956891	up	yes	Germin-like	GER7
Pavir.9KG014600.v	GO:000367	NA	nucleotide-c	1.397115	10.538534	0.0001	0.261287	45.662749	5.5129458	0.000618156	0.032991467	up	yes	Uncharacter	At4g15970
Pavir.9KG417200.v	GO:000382	01200(Carb	enoyl-coa hy	37.637714	66.443802	0.737306	1.558567	45.334178	5.5025272	3.75738E-09	1.83346E-06	up	yes	Probable en	ECHIA
Pavir.6KG316000.v	NA	NA	0	120.95571	6.578284	0.330056	2.545616	44.349284	5.4708389	0.000100871	0.008665437	up	yes	NA	NA
Pavir.5KG335900.v	GO:000078	NA	histone h1	14.714299	160.45744	1.000925	3.073814	42.989684	5.4259186	7.22635E-06	0.001028468	up	yes	Histone H1	-
Pavir.9KG523800.v	GO:000940	04141(Prot	25.3 kda he	0.418601	90.35421	0.0001	2.17246	41.781498	5.3847923	0.011840959	0.19717125	up	yes	Small heat s	HSP21
Pavir.7NG350800.v	GO:000842	00500(Starc	glycosyl hyd	2.098781	53.759174	0.023595	1.342533	40.88779	5.3535982	0.001137755	0.050174711	up	yes	Beta-glucos	BGLU16
Pavir.6KG145400.v	NA	NA	auxin-induc	5.332971	2.852093	0.153476	0.047135	40.800674	5.3505211	1.02608E-05	0.001405436	up	yes	NA	NA
Pavir.4NG101200.v	GO:000557	00860(Porp	zinc finger f	11.167377	1.0151	0.0001	0.304655	39.974658	5.3210138	0.00144074	0.058840118	up	yes	GDSL ester	At1g74460

Pavir.6KG167700.v4	GO:000467	00520(Ami	glycerophos	2.244472	1.775903	0.032591	0.069589	39.346007	5.2981453	1.28282E-05	0.001673585	up	yes	PR5-like re	PR5K;PR5K	
Pavir.9NG798900.v4	GO:000166	00230(Puri	erebp-like fa	29.331608	68.126122	0.148723	2.35876	38.866756	5.2804648	2.28828E-05	0.002755685	up	yes	Ethylene-res	RAP2-2	
Pavir.3NG050300.v4	GO:000973	NA	nitrate, from	26.240524	3.91668	0.0001	0.782439	38.537637	5.2681962	0.00106678	0.048233626	up	yes	CASP-like p	-	
Pavir.9KG204700.v4	GO:000557	00561(Glyc	zinc finger f	2.660473	1.219573	0.0001	0.10192	38.032209	5.2491498	0.000323531	0.020499212	up	yes	GDSL ester	EXL3	
Pavir.7NG388300.v4	NA	NA	wound-indu	13.568178	86.637413	1.174469	1.496808	37.512243	5.2292896	4.90178E-07	9.59243E-05	up	yes	NA	NA	
Pavir.6KG065100.v4	NA	NA	0	4.244206	3.797057	0.0001	0.217892	36.887881	5.205075	0.000279302	0.018282632	up	yes	NA	NA	
Pavir.6NG284100.v4	NA	NA	0	150.88796	10.719016	0.727702	3.667656	36.767647	5.2003649	4.96137E-05	0.005112293	up	yes	NA	NA	
Pavir.5KG456400.v4	NA	NA	0	4.424713	2.687172	0.0001	0.195154	36.423761	5.186808	0.000401587	0.023837006	up	yes	NA	NA	
Pavir.5KG623300.v4	GO:000550	00053(Asco	multi-coppe	38.80014	14.824048	0.0001	1.48107	36.203939	5.1780748	0.001779453	0.067466606	up	yes	Putative lac	LAC5	
Pavir.2NG247500.v4	GO:001602	00941(Flav	cytochrome	2.952659	33.214252	0.0001	1.005619	35.961249	5.1683712	0.002730776	0.087741633	up	yes	Geraniol 8-h	CYP76B6	
Pavir.8NG252700.v4	GO:000578	NA	receptor exp	2.858931	57.719749	0.0001	1.694044	35.757692	5.1601817	0.003304046	0.099553272	up	yes	Protein HV.	HVA22	
Pavir.3KG434400.v4	GO:000367	04141(Prote	gem-like pro	2.252334	21.730099	0.0001	0.677199	35.40893	5.1460414	0.001831339	0.068671566	up	yes	GEM-like p	At5g13200	
Pavir.1KG148900.v4	NA	NA	0	49.193047	36.210133	0.11315	2.363064	34.489418	5.1080819	2.53264E-05	0.002964455	up	yes	NA	NA	
Pavir.3KG076600.v4	GO:000973	NA	nitrate, from	6.29066	1.812745	0.0001	0.237591	34.092183	5.0913691	0.000823491	0.040551819	up	yes	CASP-like p	Sb09g008190	
Pavir.1NG314600.v4	GO:000535	NA	predicted tra	1.010732	7.528188	0.0001	0.254409	33.550562	5.068265	0.001741656	0.066503286	up	yes	Sugar trans	MST1	
Pavir.9KG544300.v4	GO:000367	NA	homeobox p	2.399443	22.292564	0.0001	0.741696	33.286789	5.0568778	0.003588576	0.105668933	up	yes	Homeobox	BEL1	
Pavir.9KG418500.v4	GO:000382	01200(Carb	enoyl-coa hy	22.337406	38.846367	0.529532	1.311155	33.23964	5.0548328	2.93355E-08	9.50192E-06	up	yes	Probable en	ECHIA	
Pavir.5NG270200.v4	GO:001678	NA	alpha/beta h	68.569786	1.755977	0.335384	1.825088	32.551111	5.0246349	0.000369288	0.022532345	up	yes	NA	NA	
Pavir.9KG652000.v4	GO:000370	00230(Puri	ap2 domain	8.124622	19.078264	0.449119	0.38823	32.486915	5.0217869	1.08095E-08	4.09363E-06	up	yes	Ethylene-res	ERF073	
Pavir.9KG128600.v4	GO:000563	NA	protein of u	56.875557	1.824785	0.0001	1.817188	32.301067	5.0135099	0.006389796	0.145417214	up	yes	NA	NA	
Pavir.9KG455200.v4	GO:000552	NA	universal str	0.585017	208.32277	2.430103	4.084588	32.067183	5.0030257	0.002363401	0.079712811	up	yes	NA	NA	
Pavir.1KG417300.v4	GO:004665	NA	cupredoxin	53.782539	3.219656	0.223943	1.570373	31.768203	4.9895116	0.000179445	0.013117311	up	yes	Uclacyanin-	At2g44790	
Pavir.2NG061100.v4	GO:000367	NA	pddexk-like	5.014423	38.353564	0.287135	1.082294	31.668664	4.9849841	1.10316E-05	0.001475052	up	yes	NA	NA	
Pavir.1KG300300.v4	GO:000521	NA	protein nrt1.	3.041389	13.409681	0.092298	0.432404	31.353168	4.9705393	6.46503E-06	0.00092714	up	yes	Protein NR	NPF2.11	
Pavir.9NG655900.v4	GO:000455	NA	glycosyl hyd	3.6451558	17.716025	0.1064203	0.5907458	30.640015	4.9373451	1.01097E-05	0.001391404	up	yes	Endo-1,4-be	At4g33820;At4g33820;At4g33820	
Pavir.9NG337500.v4	NA	NA	0	33.382	6.627763	0.0001	1.323099	30.237147	4.9182501	0.001945441	0.07143226	up	yes	NA	NA	
Pavir.1NG390800.v4	NA	NA	0	6.281701	1.639149	0.020879	0.243933	29.91122	4.9026149	0.000128627	0.010239178	up	yes	FT-interacti	FTIP3	
Pavir.9KG582600.v4	GO:000449	00073(Cuti	alkane 1-mo	0.292819	56.133751	0.102792	1.796174	29.714366	4.8930887	0.004973279	0.127202873	up	yes	Noroxomar	Cyp96T1	
Pavir.9NG684400.v4	NA	NA	0	49.563501	6.5691188	0.4554312	1.4389005	29.631885	4.8890785	1.06291E-05	0.001436577	up	yes	Cytochrome	CYP86B1;CYP86B1;CYP86B1	
Pavir.2KG343700.v4	GO:000097	00190(Oxid	myb-like dn	3.191599	1.952863	0.0001	0.175766	29.252169	4.8704717	0.000908326	0.043475819	up	yes	Transcriptio	MYB36	
Pavir.5NG050000.v4	NA	NA	polyketide c	3.354577	42.948452	0.454937	1.133481	29.150406	4.8654441	4.03286E-05	0.004280415	up	yes	NA	NA	
Pavir.2KG227500.v4	NA	NA	0	49.457565	2.931886	0.0001	1.80556	29.014018	4.8586782	0.005475305	0.133847511	up	yes	NA	NA	
Pavir.6NG004700.v4	GO:000487	NA	late embryo	1.709359	49.355618	0.885615	0.905551	28.509349	4.8333632	0.000107	0.008976715	up	yes	NDR1/HIN	NHL1;NHL1	
Pavir.3NG269200.v4	NA	NA	0	37.723409	2.6764767	0.1057449	1.3229294	28.277884	4.8216023	0.000401274	0.023837006	up	yes	NA	NA	
Pavir.4KG076400.v4	GO:000980	00940(Phen	nad depende	2.518073	28.872969	0.0001	1.112984	28.201863	4.8177185	0.00474778	0.123521137	up	yes	Cinnamoyl-	CCR1	
Pavir.8NG276600.v4	GO:000467	00520(Ami	salt stress re	2.33835	5.808449	0.0001	0.29062	28.022836	4.8085311	0.001431679	0.058533688	up	yes	Cysteine-ric	CRK6	
Pavir.9NG241700.v4	GO:000980	00940(Phen	ferulate-5-h	100.30097	742.45685	0.440505	29.975172	27.708008	4.7922311	0.002071665	0.073816683	up	yes	Cytochrome	CYP84A1	
Pavir.5NG564700.v4	GO:004316	NA	ring-h2 fing	3.8856	13.940328	0.09484	0.549167	27.679712	4.790757	2.54598E-05	0.002970808	up	yes	RING-H2 f	ATL8	
Pavir.2NG056500.v4	NA	NA	0	1.89906	13.783286	0.142906	0.425468	27.591596	4.786157	9.60596E-05	0.008335448	up	yes	Protein LA	LATE	
Pavir.4NG323300.v4	GO:000367	NA	zinc-finger d	1.79072	15.555866	0.028059	0.602554	27.507498	4.781753	0.000583278	0.031624104	up	yes	FCS-Like Z	FLZ2;FLZ2	
Pavir.5KG395400.v4	GO:000573	03013(RNA	copper trans	21.063879	3.517807	0.0001	0.89669	27.410749	4.7766699	0.002873232	0.090734961	up	yes	Protein SOI	NAKR2	
Pavir.7NG407700.v4	GO:000573	00940(Phen	alcohol o-ac	5.903863	60.077843	0.108499	2.330496	27.052825	4.7577074	0.000610441	0.032626036	up	yes	Putrescine h	PHT1	
Pavir.9KG417300.v4	NA	NA	enoyl-coa hy	90.766884	158.61023	4.667873	4.573421	26.985086	4.7540904	1.43239E-07	3.61203E-05	up	yes	NA	NA	
Pavir.1KG271900.v4	NA	NA	0	5.109761	8.805597	0.0001	0.520187	26.745542	4.7412266	0.00142796	0.0584767	up	yes	NA	NA	
Pavir.2KG379900.v4	GO:000370	00190(Oxid	myb domain	22.868526	218.74663	0.076989	9.009859	26.589545	4.7327872	0.003652167	0.10662228	up	yes	Protein OD	ODO1	
Pavir.3KG149100.v4	GO:000436	00480(Glut	glutathione	7.489633	68.471634	0.940927	1.921203	26.540118	4.7301029	1.4226E-05	0.001836813	up	yes	Glutathione	GST4	
Pavir.1NG377600.v4	GO:004665	NA	cupredoxin	41.001072	2.166439	0.0001	1.630565	26.472336	4.7264136	0.007312072	0.156441785	up	yes	Uclacyanin-	At2g44790	
Pavir.9NG245600.v4	GO:000563	NA	protein of u	101.73969	2.569417	0.054108	4.029707	25.542075	4.6748038	0.005063887	0.128340138	up	yes	NA	NA	
Pavir.9NG811800.v4	GO:000013	00052(Gala	udp-glucuro	7.689352	53.431396	0.145598	2.273965	25.261069	4.6588438	0.000408643	0.024179452	up	yes	UDP-glucur	GUX2	
Pavir.2KG337500.v4	GO:000463	00564(Glyc	phospholipa	4.190718	40.103168	0.054266	1.704514	25.184438	4.6544606	0.001065692	0.048233626	up	yes	Phospholipa	PLD1	
Pavir.2KG526000.v4	NA	NA	0	28.279711	1.292597	0.0001	1.174333	25.180072	4.6542105	0.008517896	0.168001533	up	yes	NA	NA	
Pavir.2KG451600.v4	GO:000588	NA	eama-like tr	2.04835	10.948522	0.0001	0.523029	24.844488	4.6348539	0.003426529	0.102340991	up	yes	WAT1-rela	At5g64700	
Pavir.9KG171200.v4	NA	NA	0	3.332862	108.88115	0.0001	4.519894	24.826142	4.6337882	0.011593147	0.195331535	up	yes	NA	NA	
Pavir.8NG222600.v4	GO:000573	00073(Cuti	acyltransfera	27.784401	1.9593985	0.3420108	0.8590415	24.764783	4.6302181	7.87337E-05	0.007268452	up	yes	Omega-hyd	HHT1;HHT1	
Pavir.8KG106500.v4	NA	NA	0	2.6708885	2.7662138	0.080904	0.138651	24.764192	4.6301837	0.000123658	0.009991831	up	yes	Importin be	SAD2;SAD2	
Pavir.5NG447500.v4	GO:000416	00900(Terp	(2e,6e)-farn	1.9906885	34.446517	0.0001	1.4876496	24.491491	4.6142087	0.011872382	0.197303733	up	yes	Farnesyl py	FPS;FPS	
Pavir.8NG168900.v4	GO:000662	00561(Glyc	alpha/beta-h	1.367692	7.209556	0.0001	0.352924	24.296501	4.6026767	0.003650037	0.10662228	up	yes	NA	NA	
Pavir.9KG544000.v4	GO:000573	NA	alpha/beta h	2.035586	14.635787	0.291879	0.39718	24.194406	4.5966016	3.66112E-05	0.003964242	up	yes	NA	NA	
Pavir.9NG628000.v4	GO:000367	NA	protein of u	13.158592	65.224724	0.047679	3.209378	24.065688	4.5889057	0.001234584	0.05331844	up	yes	NA	NA	
Pavir.4NG022500.v4	GO:000078	NA	histone h1	2.477904	35.0182	1.05206	0.5201	23.850056	4.5759207	8.1112E-05	0.007415139	up	yes	Histone H1	-	
Pavir.7NG393100.v4	GO:000467	04075(Plan	non-specific	1.178304	2.15013	0.050604	0.089765	23.71203	4.5675473	6.4026E-05	0.006168331	up	yes	Probable re	At5g20050	
Pavir.9KG080600.v4	GO:000685	NA	multidrug re	1.7826527	5.923128	0.0313374	0.2949695	23.615133	4.5616397	0.000145843	0.0113688	up	yes	Protein DE	DTX29;DTX29	
Pavir.6KG128800.v4	NA	NA	0	1.22936	28.25642	0.059831	1.192708	23.540808	4.5570919	0.002469871	0.082191385	up	yes	Putative CE	CIPK13	
Pavir.9KG594000.v4	GO:000449	00906(Caro	fad/nad(p)-b	1.2974029	3.9574988	0.0001	0.2235198	23.499269	4.5545439	0.002740958	0.087741633	up	yes	Monooxyge	MO2;MO2	
Pavir.5KG443900.v4	GO:000484	NA	f-box domai	1.906757	23.869543	0.54327	0.559173	23.381073	4.5472692	3.35102E-05	0.00367078	up	yes	NA	NA	
Pavir.2KG366100.v4	NA	NA	0	1.27906	2.599318	0.119437	0.050345	22.843281	4.513698	0.0001697	0.012697896	up	yes	NA	NA	
Pavir.2KG350300.v4	GO:000971	03410(Base	member of '	1.313233	2.942187	0.049209	0.138183	22.708654	4.5051703	0.000336039	0.02107844	up	yes	2-hydroxyis	HIDM	
Pavir.2NG633200.v4	GO:000551	NA	ricin-type be	3.9932127	12.276427	0.050183	0.6685097	22.637825	4.5006634	0.000397893	0.023767948	up	yes	Ricin B-like	R40G3;R40G3	
Pavir.9NG390000.v4	GO:000817	00945(Stilb	o-methyltra	3.0461569	31.04417	0.0199566	1.5181425	22.163934	4.4701421	0.002667901	0.086340255	up	yes	5-pentadeca	OMT3;OMT3;OMT3;OMT3	
Pavir.6NG153900.v4	GO:000367	00190(Oxid	myb-like dn	8.626385	3.371275	0.053431	0.491794	22.00497	4.4597575	0.000193918	0.013825568	up	yes	Transcriptio	MYB93	
Pavir.3NG096200.v4	NA	NA	0	5.70179	24.273357	0.084805	1.288748	21.823073	4.4477823	0.000599054	0.032200677	up	yes	NA	NA	
Pavir.5KG509900.v4	GO:000550	00073(Cuti	fatty acid or	2.044053	2.995317	0.0001	0.231332	21.774733	4.4445831	0.002635609	0.085741949	up	yes	Cytochrome	CYP86A1	
Pavir.9NG691500.v4	GO:000436	00480(Glut	glutathione	13.427217	44.607536	0.213398	2.469421	21.632005	4.4350955	0.000117123	0.009559058	up	yes	Protein IN2	IN2-1	
Pavir.3KG320400.v4	GO:000563	NA	uncharacteri	1.8546081	3.6538285	0.0001	0.2549106	21.600813	4.4330137	0.002740184	0.087741633	up	yes	Probable cy	LOGL7;LOGL7	
Pavir.4NG330000.v4	GO:000557	00860(Porp	zinc finger f	1.712346	3.265225	0.0001	0.230364	21.598041	4.4328286	0.003186106	0.097484986	up	yes	GDSL ester	At5g55050	
Pavir.5NG034700.v4	GO:000368	NA	transcriptio	3.087612	11.364645	0.109801	0.56056	21.558917	4.4302128	0.000110181	0.009199635	up	yes	Transcriptio	KUA1	
Pavir.8KG344800.v4	GO:000578	NA	receptor exp	1.4081539	19.853427	0.0001	0.9940697	21.386268	4.4186129	0.010456937	0.185504479	up	yes	Protein HV.	HVA22;HVA22;HVA22;HVA22;HVA22;HVA22;HVA22	
Pavir.4KG064000.v4	GO:000367	04626(Plan	ethylene-res	2.69665	164.37592	2.456804	5.359642	21.374492	4.4178183	0.00107614	0.04849422	up	yes	Ethylene-res	RAP2-9	
Pavir.6KG041100.v4	GO:000486	NA	pathogenesi	4.258166	12.005829	0.249901	0.511647	21.356494	4.4166029	0.000114013	0.009394348	up	yes	NA	NA	
Pavir.2NG594300.v4	GO:000402	01200(Carb	alcohol deh	1.4847185	6.5324511	0.0001	0.3763946	21.294248	4.4123919	0.004667752	0.122881305	up	yes	Alcohol deh	At5g42250;At1g22440	
Pavir.7NG020100.v4	GO:001602	00903(Lim	cytochrome	1.044475	7.913251	0.0001	0.422782	21.182566	4.4048055	0.006764299	0.149943964	up	yes	Cytochrome	CYP78A7	
Pavir.2NG578500.v4	NA	NA	0	156.92464	2.345767	3.453778	4.09629	21.095228	4.3988448	0.000987975	0.045974104	up	yes	NA	NA	

Pavir.5NG451300.v	GO:001602	02010(ABC	atp-binding	2.956824	105.88317	1.284353	3.898728	20.999092	4.392255	0.001149799	0.050527955	up	yes	ABC transp	ABCB4
Pavir.7KG178200.v	GO:00041	NA	aspartyl pro	10.733094	21.663044	0.020639	1.528285	20.915253	4.3864836	0.001182198	0.051589701	up	yes	Aspartic pr	nep1
Pavir.9KG652100.v	GO:00016	00230(Puri	erebp-like f	69.353354	84.933829	0.2112882	7.2009352	20.815236	4.379568	0.000690642	0.03584186	up	yes	Ethylene-re	RAP2-2;RAP2-2
Pavir.9KG641100.v	GO:000727	NA	eukaryotic c	3.841426	19.248423	0.0001	1.114103	20.723198	4.3731747	0.006566347	0.147030604	up	yes	Cytochrome	At4g12980
Pavir.5KG380900.v	GO:00044	00905(Bras	cytochrome	5.4104446	37.484925	0.6064571	1.4698602	20.659352	4.3687231	2.55809E-05	0.002975702	up	yes	Cytochrome	CYP72A15;CYP72A15
Pavir.7KG405500.v	GO:000367	NA	wound-indu	15.483505	47.568188	2.19431	0.870933	20.569884	4.3624618	4.95432E-06	0.000744423	up	yes	NA	NA
Pavir.5KG203300.v	GO:000573	NA	copper trans	6.4694864	31.658826	0.0749305	1.7833893	20.517627	4.358792	0.000609143	0.032603027	up	yes	Protein PYI	PI21;PI21
Pavir.4KG341100.v	GO:001602	NA	transmembr	1.3338061	6.7306609	0.1691089	0.2246326	20.481631	4.3562587	4.70031E-05	0.004905691	up	yes	Transmemb	TMN8;TMN8
Pavir.9NG662000.v	GO:000727	NA	eukaryotic c	6.408141	28.407522	0.033287	1.672205	20.413853	4.3514766	0.001459243	0.059466546	up	yes	Cytochrome	At4g12980
Pavir.7KG405400.v	GO:000367	NA	wound-indu	1.3195996	8.6313952	0.3273448	0.1613241	20.36347	4.3479115	0.000343965	0.021468101	up	yes	NA	NA
Pavir.5NG190300.v	NA	NA	leucine-rich	1.434113	2.725137	0.0001	0.205017	20.277451	4.3418044	0.003118626	0.096046002	up	yes	NA	NA
Pavir.7NG077500.v	GO:00053	04075(Plan	manganese	1.431226	17.996666	0.0001	0.970987	20.006335	4.322385	0.010860462	0.188567538	up	yes	Metal trans	NRAMP4
Pavir.4KG163100.v	GO:00046	00940(Pher	peroxidase	29.693174	1.665971	0.709423	0.858196	20.004315	4.3222394	0.000181106	0.013136497	up	yes	Peroxidase	PER11
Pavir.7KG030500.v	GO:000662	NA	thioesterase	0.6017254	41.36657	0.0001	2.13941	19.615844	4.2939475	0.030100654	0.324339516	up	yes	Acyl-acyl c	ALT3;ALT3;ALT3
Pavir.7KG404700.v	NA	NA	wound-indu	1.025258	34.273396	1.200298	0.612573	19.471134	4.283265	0.00083282	0.040643524	up	yes	NA	NA
Pavir.3NG290800.v	GO:000467	04075(Plan	serine/threo	1.309139	17.239168	0.119215	0.837748	19.38247	4.2766806	0.001086207	0.048701743	up	yes	CBL-intera	CIPK4
Pavir.2NG294600.v	GO:00040	NA	arylformami	1.447557	6.400769	0.164335	0.243664	19.23614	4.2657474	0.000227	0.015535679	up	yes	Cyclase-lik	CYL4
Pavir.5NG108600.v	GO:00055	00073(Cuti	cytochrome	5.527586	89.981209	0.124023	4.859828	19.163654	4.2603007	0.004750434	0.123521137	up	yes	Cytochrome	CYP94C1
Pavir.3NG061000.v	GO:000563	NA	2-alkenal re	2.7925489	45.809737	0.0001	2.5474724	19.077882	4.2538291	0.01831346	0.247418968	up	yes	NADPH-de	AER;AER
Pavir.9NG622300.v	GO:00043	00480(Glut	glutathione	5.189883	54.683693	1.223108	1.926337	19.010834	4.2487499	0.000109527	0.009165352	up	yes	Glutathione	GST4
Pavir.1NG359100.v	NA	NA	0	2.10133	8.907719	0.0001	0.580221	18.97062	4.2456949	0.006879867	0.151522424	up	yes	NA	NA
Pavir.9NG565400.v	GO:00055	00073(Cuti	cytochrome	5.731231	96.596313	0.12413	5.319528	18.79757	4.2324743	0.005450813	0.133532772	up	yes	Cytochrome	CYP94C1
Pavir.7NG185800.v	GO:000941	00906(Carc	zeaxanthin	7.293602	5.7181516	0.2085203	0.4839712	18.789766	4.2318752	3.2446E-06	0.000518763	up	yes	Zeaxanthin	ZEP;ZEP;ZEP;ZEP
Pavir.2NG502800.v	GO:000981	00942(Antl	glucosyl/glu	2.465843	7.036049	0.0001	0.506467	18.757424	4.2293898	0.005715413	0.136102468	up	yes	UDP-glyco	UGT71K1
Pavir.9KG138400.v	GO:00098	00940(Pher	ferulate-5-h	27.391554	113.63692	0.299823	7.284341	18.595124	4.2168525	0.001317332	0.055676155	up	yes	Cytochrome	CYP84A1
Pavir.3NG328900.v	NA	04075(Plan	saur family	0.497108	24.167877	0.0001	1.34096	18.392156	4.2010187	0.023945261	0.285708255	up	yes	NA	NA
Pavir.2KG086900.v	GO:000367	NA	pddexk-like	6.885122	71.485229	1.544603	2.720949	18.372851	4.1995036	0.000149905	0.01151819	up	yes	NA	NA
Pavir.5NG326000.v	GO:00052	04145(Phag	actin//subfa	3.857422	26.316698	0.447577	1.196503	18.353194	4.1979593	6.66004E-05	0.006367367	up	yes	Actin-1 OS	ACT1
Pavir.9KG591200.v	GO:00431	NA	ring finger c	1.230317	14.335055	0.02357	0.833753	18.155785	4.1823574	0.003608575	0.106008587	up	yes	RING-H2 f	ATL47
Pavir.5KG126400.v	GO:000557	00514(Othe	glycosyltran	8.831876	2.482831	0.0001	0.627921	18.016447	4.1712426	0.007510636	0.157709059	up	yes	Alpha-1,3-	XAT3
Pavir.7KG272600.v	GO:000007	01200(Carb	cyclin	1.754382	16.315569	0.194815	0.810448	17.975347	4.1679477	0.000706592	0.036418057	up	yes	Cyclin-P4-1	CYCP4-1
Pavir.5KG768400.v	GO:000563	04075(Plan	armadillo/b	4.273392	15.060146	0.384224	0.70746	17.70983	4.1464785	4.08971E-05	0.00432853	up	yes	NA	NA
Pavir.1KG489100.v	GO:000367	04712(Circ	dof domain,	5.798935	52.364796	1.013899	2.27804	17.668532	4.1431103	9.54885E-05	0.008335023	up	yes	Dof zinc fin	DOF4
Pavir.9KG493900.v	GO:00055	00053(Asc	multi-coppe	31.437933	15.050863	0.0295045	2.619145	17.551887	4.1335542	0.002964538	0.092741035	up	yes	Laccase-10	LAC10;LAC10
Pavir.9KG409100.v	GO:000455	00500(Star	glucan endo	16.307549	9.544692	0.384633	1.088713	17.546619	4.1331212	3.34084E-06	0.000531887	up	yes	Glucan end	At5g56590
Pavir.1KG232500.v	GO:000573	NA	26s proteas	1.051416	28.961563	0.0001	1.711367	17.536405	4.1322811	0.027131425	0.306772055	up	yes	NA	NA
Pavir.7KG087900.v	NA	NA	0	10.059403	19.204079	0.0001	1.67553	17.464167	4.1263259	0.006315681	0.144254761	up	yes	NA	NA
Pavir.2KG589500.v	GO:00046	00940(Pher	peroxidase (	101.40479	41.599377	0.865505	7.334	17.440585	4.1243765	0.000200839	0.014237939	up	yes	Peroxidase	PER66
Pavir.7NG254300.v	NA	NA	plant plecks	6.7043358	10.678137	0.683852	0.3141526	17.417228	4.1224431	1.36177E-06	0.000232017	up	yes	NA	NA
Pavir.9KG154400.v	GO:000557	NA	peptidase of	1.551067	7.513738	0.0001	0.523777	17.303308	4.112976	0.008841953	0.172158188	up	yes	Basic secret	-
Pavir.9KG620900.v	GO:000685	NA	multidrug r	1.920641	14.007124	0.226112	0.697952	17.236647	4.1074073	0.000188648	0.013578658	up	yes	Protein DE	DTX42
Pavir.9NG825000.v	GO:00041	NA	aspartyl pro	15.520022	35.161224	0.0001	2.941836	17.227175	4.1066142	0.014722766	0.220917927	up	yes	Aspartic pr	nep1
Pavir.1KG171900.v	GO:000382	NA	n-carbamoy	399.30624	1225.0527	1.4139565	92.982521	17.207834	4.1049936	0.004017596	0.112540498	up	yes	Probable in	At3g16190;At3g16190
Pavir.2KG594100.v	GO:001507	NA	osmotic stre	4.406888	14.641305	0.339109	0.77409	17.11122	4.0968707	9.53438E-06	0.001321901	up	yes	Potassium t	HAK7
Pavir.9KG421700.v	GO:000573	00052(Gala	inositol 3-al	7.594262	48.41972	0.349137	2.941854	17.0204	4.089193	0.000418194	0.0245738	up	yes	Galactinol s	GOLS1
Pavir.8NG229000.v	NA	NA	0	36.825067	35.014229	0.4835469	3.7404796	17.007303	4.0880824	0.000102888	0.008746163	up	yes	Protein LA	LA1;LA1;LA1;LA1
Pavir.1KG427600.v	GO:00057	NA	protein tricl	13.812728	2.0593437	0.0001	0.9387306	16.906216	4.0794819	0.012471392	0.202939636	up	yes	Protein tricl	TBL34;TBL34
Pavir.3KG146000.v	GO:00043	00480(Glut	glutathione	40.709412	324.92545	8.59204	13.09621	16.858661	4.0754181	0.000154713	0.01183919	up	yes	Glutathione	GST4
Pavir.1NG270300.v	GO:000367	00500(Star	gb	3.054	1.079566	0.0001	0.245124	16.856286	4.0752148	0.007116588	0.154919788	up	yes	Transcriptic	OFP2
Pavir.9KG413500.v	GO:001602	00941(Flav	flavonoid 3'	94.527657	434.61639	0.385823	31.122522	16.793775	4.0698546	0.005839137	0.137393622	up	yes	Flavonoid 3	CYP75A5
Pavir.4KG024500.v	GO:000367	NA	early noduli	4.1042751	49.072482	1.909818	1.2696013	16.725305	4.0639606	0.00016999	0.012697896	up	yes	Early nodul	-;-
Pavir.2NG578400.v	NA	NA	0	132.49187	8.173565	1.576542	6.843286	16.70645	4.0623333	0.000711303	0.036560583	up	yes	NA	NA
Pavir.9KG004100.v	GO:000573	NA	gdp-fucose	2.162243	7.160454	0.044295	0.514558	16.681841	4.0602066	0.000717766	0.036792104	up	yes	O-fucosyltr	OFUT19
Pavir.2NG585300.v	GO:000872	NA	guanosine-3	2.605052	129.86101	1.366016	6.593091	16.643332	4.0568724	0.003586404	0.105668933	up	yes	Probable G	RSH3
Pavir.7NG191400.v	GO:00039	00592(alph	nadh oxidor	2.221806	2.457795	0.0001	0.28114	16.639173	4.0565118	0.004351029	0.118204918	up	yes	Putative 12	OPR11
Pavir.5KG074400.v	NA	NA	levansucras	14.507033	18.681303	0.106303	1.898816	16.551804	4.0489165	0.000773886	0.038821408	up	yes	NA	NA
Pavir.7NG289500.v	GO:000367	04626(Plan	f3o9.29 pro	1.839313	2.123746	0.0001	0.239594	16.533826	4.0473487	0.006106641	0.141632613	up	yes	Myb-relate	-
Pavir.1KG485800.v	GO:000333	NA	amino acid	12.543272	49.931814	0.9308474	2.8487784	16.529437	4.0469657	6.4704E-05	0.006217708	up	yes	Probable an	AAP7;AAP7;AAP7;AAP7;AAP7;AAP7;AAP7
Pavir.9KG189300.v	GO:000561	NA	ubiquitin//s	1.360076	17.800396	0.176654	0.990027	16.42306	4.0376511	0.001279391	0.054501785	up	yes	Polyubiquit	UBQ8
Pavir.9NG700300.v	GO:00055	00053(Asc	multi-coppe	144.38294	100.30784	0.242547	14.670012	16.408369	4.0363599	0.003158214	0.096836422	up	yes	Laccase-10	LAC10
Pavir.5NG584800.v	GO:00055	00053(Asc	multi-coppe	565.55341	12.424958	2.185498	33.145966	16.358744	4.0319901	0.008078229	0.163096878	up	yes	Laccase-12	LAC12
Pavir.9NG573200.v	GO:000552	NA	universal st	0.635376	114.32536	0.486896	6.592456	16.238878	4.02138	0.013236782	0.209056578	up	yes	NA	NA
Pavir.5NG642100.v	NA	NA	aba/wds ind	1.007289	63.058144	0.938814	3.0172	16.19444	4.0174267	0.003058767	0.094746131	up	yes	Abscisic str	ASR3
Pavir.1NG302700.v	GO:000104	04075(Plan	helix-loop-l	1.5712092	14.438545	0.218791	0.7702791	16.186673	4.0167346	0.000477728	0.027488014	up	yes	Transcriptic	LRL3;LRL3;RHL1
Pavir.7KG261200.v	GO:000467	00500(Star	protein kina	1.51318	5.055687	0.053041	0.355175	16.091645	4.0082399	0.00079358	0.039545343	up	yes	L-type lecti	SIT2
Pavir.2KG343000.v	GO:000367	NA	tetraspanin	11.086767	88.348167	0.371226	5.861819	15.952866	3.9957438	0.002013962	0.07262055	up	yes	Tetraspanin	TET8
Pavir.2KG557000.v	GO:000551	04626(Plan	non-specific	4.797478	42.990612	0.172212	2.829616	15.919663	3.9927379	0.001812731	0.068177911	up	yes	Serine/threc	RIPK
Pavir.1NG455300.v	GO:000467	00520(Ami	non-specific	1.676491	2.231181	0.0001	0.247763	15.765451	3.9786945	0.007159671	0.15531774	up	yes	G-type lecti	B120
Pavir.8KG024100.v	GO:000487	NA	root phototr	25.21664	70.302055	1.987634	4.086157	15.726372	3.975114	2.36477E-05	0.002820679	up	yes	Root photot	RPT2
Pavir.7NG387600.v	GO:000367	NA	wound-indu	9.269739	32.260143	1.939354	0.705151	15.704218	3.9730802	5.43306E-05	0.005472821	up	yes	NA	NA
Pavir.3KG366100.v	NA	NA	0	2.521135	1.449034	0.0001	0.253172	15.675515	3.9704409	0.006070844	0.141348194	up	yes	NA	NA
Pavir.3KG457700.v	GO:000527	NA	ureide perm	6.069145	8.638722	0.060544	0.883168	15.585122	3.9620976	0.001558799	0.061917697	up	yes	Ureide perm	UPS1
Pavir.7NG090700.v	GO:000367	NA	zinc-finger	2.123591	16.433874	0.248582	0.942183	15.58449	3.962039	0.000510494	0.028828877	up	yes	FCS-Like Z	FLZ8
Pavir.6NG273500.v	GO:00041	NA	aspartyl pro	7.016456	23.263502	0.2525	1.697085	15.531489	3.9571242	0.000188107	0.013565747	up	yes	Aspartyl pr	AP37
Pavir.2NG039900.v	NA	NA	0	1.282062	71.037758	0.0001	4.664989	15.502345	3.9544146	0.045802203	0.403216066	up	yes	NA	NA
Pavir.7NG387900.v	GO:000367	NA	wound-indu	11.388677	58.650436	2.118383	2.41065	15.464474	3.9508859	2.27137E-05	0.002744123	up	yes	NA	NA
Pavir.8NG022100.v	GO:00088	00770(Pant	4'-phospho	1.5869916	5.742245	0.211029	0.262973	15.462459	3.9506979	0.000316051	0.020161291	up	yes	NA	NA
Pavir.3KG199700.v	GO:000367	NA	protein of u	5.210557	57.949829	0.18836	3.907248	15.421492	3.9468704	0.003071032	0.095002339	up	yes	NA	NA
Pavir.7NG230300.v	GO:000573	NA	methyltrans	2.208324	7.5623795	0.171125	0.4629386	15.409659	3.945763	0.000164353	0.012375231	up	yes	Protein-L-is	PCM;PCM
Pavir.8KG050700.v	GO:000521	NA	predicted tr	1.167176	19.06353	0.423468	0.891349	15.386709	3.9436127	0.000927576	0.044172122	up	yes	NA	NA
Pavir.5KG191400.v	GO:000552	NA	universal st	7.745335	58.275833	1.617963	2.681439	15.355896	3.9407207	0.000160649	0.012155889	up	yes	NA	NA
Pavir.6KG072800.v	NA	NA	0	3.681715	116.0372	0.524137	7.395075	15.117529	3.9181504	0.00553983	0.134626641	up	yes	NA	NA

Pavir.1NG495600.v	GO:000367	00190(Oxic	myb-like dn	34.183216	10.120481	0.0001	2.934756	15.095697	3.9160655	0.015506176	0.228296849	up	yes	Transcriptic	MYB93
Pavir.2KG589600.v	GO:00046(	00940(Pher	peroxidase /	3.141882	1.098831	0.0001	0.281978	15.033831	3.9101408	0.010600789	0.18673391	up	yes	Peroxidase	PER66
Pavir.6KG331900.v	GO:000382	03410(Basc	3-methylade	109.87515	580.65536	0.271253	45.709942	15.017672	3.9085893	0.012427394	0.202479745	up	yes	NA	NA
Pavir.5NG585100.v	GO:00055(	00053(Asc	multi-coppe	50.009922	4.701518	0.036593	3.627313	14.932545	3.9003881	0.009666258	0.179708223	up	yes	Putative lac	LAC5
Pavir.4NG274900.v	GO:000367	NA	genomic dn	2.722865	2.271946	0.15681	0.177724	14.930653	3.9002053	0.000355683	0.021895072	up	yes	VQ motif-c	VQ22
Pavir.9KG621900.v	GO:000573	NA	microtubule	18.670885	171.88342	0.021393	12.76326	14.904926	3.8977173	0.024442234	0.288976738	up	yes	Microtubul	MAP70.4
Pavir.6KG103600.v	NA	NA	expressed p	3.773413	6.323872	0.120218	0.558346	14.880372	3.8953387	0.000869383	0.041878642	up	yes	NA	NA
Pavir.2NG429400.v	GO:001602	00903(Lim	flavonoid 3'	2.341244	31.277149	0.023223	2.2384	14.86472	3.8938204	0.010352057	0.184953805	up	yes	Cytochrome	CYP78A9
Pavir.5KG092700.v	NA	00564(Glyc	lysophosph	2.329751	52.525414	0.112982	3.591186	14.809038	3.888406	0.007367046	0.156828349	up	yes	Lecithin-ch	Os03g0232800
Pavir.9NG799000.v	GO:00037(	00230(Puri	ap2 domain	3.840175	2.919103	0.189305	0.270533	14.699259	3.8776716	7.46269E-05	0.006992411	up	yes	Ethylene-re	EREBP1
Pavir.3KG136900.v	GO:000573	00073(Cuti	transferase f	55.909851	218.22168	0.182899	18.528635	14.650404	3.8728685	0.008333486	0.165668823	up	yes	Acyl transf	AT4
Pavir.7KG317800.v	GO:000367	00190(Oxic	f3o9.29 pro	9.745284	13.553767	0.0001	1.593042	14.624592	3.8703244	0.011660795	0.195931866	up	yes	Myb-relatec	-
Pavir.7NG387700.v	GO:000367	NA	wound-indu	24.096296	137.27811	6.015575	5.036032	14.601895	3.8680837	3.17113E-05	0.003525116	up	yes	NA	NA
Pavir.8KG308700.v	GO:000981	03013(RN/	hydroquino	16.285889	160.90782	2.95096	9.327099	14.431736	3.851173	0.000751162	0.038084222	up	yes	Hydroquino	AS
Pavir.9NG255100.v	GO:000367	00190(Oxic	myb-like dn	1.538877	29.869617	0.932903	1.243515	14.431278	3.8511272	0.001397248	0.057754467	up	yes	Transcriptic	MYB87
Pavir.7NG201700.v	GO:000425	04144(End	proprotein c	3.755035	4.479716	0.044598	0.530942	14.307869	3.8387369	0.000812004	0.04019688	up	yes	Subtilisin-li	SBT1.5
Pavir.5KG603500.v	GO:004316	NA	ring-h2 fing	1.496186	4.805561	0.108322	0.336831	14.156362	3.8233787	0.000637622	0.033742764	up	yes	RING-H2 f	ATL74
Pavir.9KG560200.v	GO:000557	NA	probable lip	2.57771	32.9967	0.290873	2.229008	14.117496	3.8194123	0.001975503	0.07185437	up	yes	Non-specifi	LTP-2
Pavir.1KG484300.v	GO:000419	NA	aspartyl pro	8.305428	1.275583	0.060714	0.618451	14.107045	3.8183439	0.001946787	0.07143226	up	yes	Aspartyl pr	At5g10770
Pavir.9NG191100.v	GO:000685	NA	multidrug re	2.1752904	5.6769247	0.0001	0.5573108	14.086944	3.8162868	0.012658713	0.204359507	up	yes	Protein DE	DTX29;DTX29
Pavir.1NG465300.v	GO:000419	NA	aspartyl pro	1.555761	1.182545	0.0001	0.196343	13.939443	3.801101	0.009972651	0.181831401	up	yes	Aspartyl pr	At5g10770
Pavir.9NG558800.v	GO:001602	00941(Flav	flavonoid 3'	102.82064	294.32382	0.62209	27.977417	13.886409	3.7956017	0.004797615	0.124489486	up	yes	Flavonoid 3	CYP75A1
Pavir.7KG083600.v	GO:001602	00380(Tryp	cytochrome	89.415871	566.41443	0.093371	47.483208	13.78473	3.7849991	0.025171203	0.294261858	up	yes	Cytochrome	CYP93G1
Pavir.6KG397400.v	NA	NA	0	5.1889528	63.690635	0.1670288	4.8451773	13.742369	3.7805589	0.005779092	0.136909091	up	yes	Non-specifi	C4;C4
Pavir.7NG256600.v	GO:000551	00940(Pher	shikimate o	96.518326	607.82715	8.509414	43.174702	13.627891	3.7684904	0.000987482	0.045974104	up	yes	Hydroxycin	HCT1
Pavir.3KG176100.v	GO:00046(	00940(Pher	peroxidase (	0.769062	29.209017	1.098339	1.105317	13.603793	3.765937	0.002208557	0.076683618	up	yes	Peroxidase	PER1
Pavir.1NG314700.v	NA	NA	0	1.6781949	1.5505794	0.1884051	0.0496319	13.564174	3.7617293	0.000721627	0.036939613	up	yes	NA	NA
Pavir.6KG231800.v	GO:000419	NA	aspartyl pro	1.407542	11.360215	0.132401	0.810166	13.545729	3.7597661	0.001639239	0.063957557	up	yes	Aspartic pr	nep1
Pavir.3NG201000.v	GO:000367	04141(Prot	gem-like pr	1.623953	1.238586	0.0001	0.211274	13.542531	3.7594255	0.009960182	0.181831401	up	yes	GEM-like p	At5g13200
Pavir.5KG658300.v	GO:000563	01200(Carb	fructose-1,6	4.850207	39.618614	0.095817	3.189771	13.534509	3.7585707	0.004901489	0.126486019	up	yes	Fructose-1,(	CFBP
Pavir.5NG422000.v	GO:00037(	04626(Plan	wrky dna -b	1.953441	7.555786	0.030045	0.675241	13.482796	3.7530478	0.00259693	0.084995151	up	yes	WRKY tra	WRKY71
Pavir.9NG300700.v	GO:000484	NA	ubiquitinyl	2.071901	25.455977	0.289949	1.7541926	13.466718	3.7513264	0.001732102	0.066340751	up	yes	Ubiquitin c	UBP12;UBP12
Pavir.9NG558700.v	GO:000459	01200(Carb	oxoglutarat	13.419811	25.485134	0.052249	2.839427	13.454116	3.7499757	0.004550741	0.121438195	up	yes	Flavonoid 3	CYP75A6
Pavir.2NG389600.v	GO:000967	NA	auxin efflux	0.747271	21.419981	0.591529	1.069165	13.348186	3.7385718	0.002095578	0.073961786	up	yes	Protein PIN	PILS1
Pavir.7NG089800.v	GO:000561	04144(End	cupin doma	197.0594	372.30133	29.669695	13.015946	13.33846	3.7375202	3.47569E-05	0.003785274	up	yes	Germin-like	Os08g0231400
Pavir.7NG309200.v	GO:004316	NA	ring finger c	5.978803	51.294571	2.259287	2.044429	13.307889	3.7342098	0.000146847	0.011399742	up	yes	RING-H2 f	ATL8
Pavir.8KG163300.v	GO:000969	NA	dirigent pro	2.111548	6.723235	0.029476	0.639346	13.209468	3.7235005	0.005647364	0.135497056	up	yes	Dirigent pr	DIR7
Pavir.1NG239400.v	GO:000419	NA	aspartyl pro	1.378776	5.39184	0.0001	0.515116	13.141315	3.7160378	0.018159017	0.246440481	up	yes	Aspartic pr	At1g65240
Pavir.2NG276200.v	GO:000981	NA	sterol 3beta	1.1417409	4.1962273	0.1165405	0.2901627	13.124974	3.7142427	0.000565881	0.030936632	up	yes	Sterol 3-bet	UGT80A2;UGT80A2
Pavir.9KG101400.v	GO:005121	00904(Dite	oxidoreduct	47.772041	132.16156	1.764369	11.94505	13.124816	3.7142253	0.000535	0.029604647	up	yes	Naringenin,	F3H
Pavir.6NG268200.v	GO:000573	NA	oligopeptid	6.9819619	27.975326	0.2037211	2.4629247	13.109085	3.7124951	0.001061811	0.048182876	up	yes	Oligopeptid	OPT5;OPT5;OPT5
Pavir.9KG650100.v	GO:000013	00052(Gala	udp-glucuro	11.842687	145.64452	0.278429	11.749861	13.093067	3.7107311	0.010675106	0.187407705	up	yes	UDP-glucu	GUX2
Pavir.2NG376300.v	GO:00055(	NA	ef-hand calc	1.876683	1.57669	0.0001	0.263674	13.092166	3.7106319	0.012023415	0.198748695	up	yes	NA	NA
Pavir.5NG328900.v	GO:000551	00941(Flav	chalcone isc	35.172626	239.96704	0.389425	20.68321	13.056728	3.7067215	0.009139407	0.174757488	up	yes	Chalcone--f	CHI
Pavir.4NG311300.v	GO:00055(	00260(Glyc	choline mor	1.401667	5.060175	0.055133	0.441855	13.002008	3.7006626	0.00280626	0.089258594	up	yes	Choline mo	At4g29890
Pavir.5NG479200.v	NA	NA	0	3.631686	10.725848	0.0001	1.104271	13.000644	3.7005112	0.01673046	0.237661086	up	yes	Pathogen-re	-
Pavir.5NG619800.v	GO:005121	00941(Flav	oxidoreduct	1.4982149	12.246895	0.12957	0.928893	12.985915	3.6988758	0.0020205	0.072677271	up	yes	Protein SR(	SRG1;SRG1
Pavir.9NG313800.v	GO:001602	00941(Flav	flavonoid 3'	3.76112	17.057108	0.030691	1.576723	12.951379	3.6950338	0.008275342	0.165124501	up	yes	Trimethyltri	CYP92C6
Pavir.7KG254700.v	GO:000588	01200(Carb	opt oligope	3.690353	9.294475	0.340787	0.664199	12.920407	3.6915796	4.93235E-05	0.005105324	up	yes	Probable m	YSL12
Pavir.9NG824300.v	GO:000582	00040(Pent	aldo/keto re	1.287254	10.15816	0.274102	0.618679	12.819957	3.6803195	0.001044016	0.047490076	up	yes	Deoxymugi	DMAS1-A
Pavir.5KG586600.v	GO:000588	01200(Carb	eama-like tr	2.117355	3.723989	0.081874	0.374062	12.811763	3.6793971	0.000829294	0.040643524	up	yes	WAT1-rela	At5g07050
Pavir.4NG296200.v	NA	NA	0	1.244969	2.304013	0.0001	0.277778	12.771727	3.6748817	0.011625364	0.195737083	up	yes	NA	NA
Pavir.9NG129900.v	GO:001649	NA	cytochrome	7.53438	74.648361	1.482833	4.968509	12.73886	3.6711642	0.000806318	0.040073775	up	yes	Probable tra	CYB561C
Pavir.3NG123600.v	GO:000588	02010(ABC	abc transpo	2.971328	1.974405	0.016095	0.372472	12.728134	3.6699491	0.003229662	0.09822653	up	yes	ABC transp	ABCG23
Pavir.6KG329100.v	GO:000521	NA	oligopeptid	7.948882	20.357988	0.087927	2.143897	12.68329	3.6648571	0.002010915	0.07262055	up	yes	Protein NR	NPF5.10
Pavir.8KG062700.v	NA	NA	0	0.644686	24.822817	0.0001	2.010852	12.664401	3.662707	0.044602224	0.398414767	up	yes	NA	NA
Pavir.5NG343900.v	GO:000557	00514(Othe	glycosyltran	2.057528	2.527608	0.087817	0.275989	12.603245	3.6557233	0.000577324	0.031350638	up	yes	Xylan glyco	MUCI21
Pavir.5KG250600.v	NA	NA	expressed p	3.278872	4.551841	0.0001	0.623341	12.560472	3.6508187	0.013942761	0.214437719	up	yes	NA	NA
Pavir.2NG449200.v	GO:00037(	00190(Oxic	myb domair	28.950672	94.035683	0.168542	9.65982	12.513413	3.6454035	0.007103755	0.154909682	up	yes	Protein OD	ODO1
Pavir.9KG329500.v	GO:000557	NA	pollen aller	56.155972	56.629295	1.017772	8.02662	12.470188	3.6404113	0.000423895	0.024808423	up	yes	Expansin-B	EXPB3
Pavir.9NG400000.v	NA	04075(Plan	camp-respo	1.123459	11.919048	0.726564	0.327533	12.373156	3.6291417	0.001730515	0.066340751	up	yes	Protein FD	FD
Pavir.1NG104100.v	NA	NA	cysteine-ricl	3.143352	39.747627	0.0001	3.479691	12.325734	3.6236017	0.041161293	0.38167652	up	yes	NA	NA
Pavir.1KG496300.v	GO:000402	00010(Glyc	aldehyde de	5.5006831	9.815431	0.0001	1.24285	12.32239	3.6232102	0.019288579	0.254738055	up	yes	Aldehyde d	ALDH2B4;ALDH2B4;ALDH2B4;ALDH2B7
Pavir.9NG255200.v	NA	NA	0	3.2839815	66.611878	1.4141617	4.2733957	12.289258	3.6193259	0.001969203	0.071764192	up	yes	Protein NE(	NDX1;NDX1;NDX1
Pavir.7NG368000.v	GO:00095(	00941(Flav	anthocyanid	1.477882	3.136315	0.202859	0.173575	12.257652	3.6156108	0.000717727	0.036792104	up	yes	Anthocyani	ANR
Pavir.5NG482100.v	GO:000981	00942(Antl	glucosyl/glu	12.230111	1.408368	0.306588	0.808601	12.229747	3.6123226	0.001509903	0.060740453	up	yes	Anthocyani	RhGT1
Pavir.1NG409000.v	GO:000573	NA	nudix hydro	43.586842	58.630444	1.494736	6.869193	12.221204	3.6113146	0.000151451	0.011613229	up	yes	Nudix hydr	NUDT18
Pavir.3KG348300.v	GO:000367	NA	pddexk-like	1.5729271	27.880561	0.2633289	2.1554765	12.176874	3.6060719	0.004670229	0.122881305	up	yes	NA	NA
Pavir.7KG033000.v	GO:000432	00592(alph	amp-bindin	2.577336	4.695075	0.0001	0.597267	12.174109	3.6057443	0.01709507	0.240475103	up	yes	4-coumarat	4CLL7
Pavir.4NG067600.v	NA	NA	0	15.615005	33.632126	0.904151	3.151472	12.142926	3.6020442	6.21806E-05	0.006036984	up	yes	NA	NA
Pavir.3KG457600.v	GO:000527	NA	ureide perm	5.12643	13.129685	0.516779	0.986817	12.141636	3.6018909	0.000510474	0.028828877	up	yes	Ureide pern	UPS2
Pavir.9KG339000.v	GO:000972	NA	transcriptio	105.57587	695.2069	9.1363645	57.442877	12.027514	3.5882666	0.002016091	0.072627606	up	yes	Transcriptic	MYBS2;MYBS2
Pavir.3NG284100.v	NA	NA	0	11.095717	27.53728	1.2245975	1.9906724	12.015475	3.5868218	1.67926E-05	0.002110201	up	yes	Patatin-like	PLP3;PLP3;PLP3
Pavir.2NG005900.v	GO:001507	NA	osmotic stre	4.182225	11.121671	0.051134	1.224806	11.994213	3.5842666	0.002711356	0.087520426	up	yes	Potassium t	HAK22
Pavir.4NG131800.v	GO:000375	03040(Splic	peptidyl-prc	1.1161653	4.227684	0.3510345	0.0984405	11.889091	3.5715666	0.001029484	0.047056931	up	yes	Peptidyl-pr	CYP40;CYP40;CYP40;CYP40;CYP40;CYP40;CYP40
Pavir.5KG340000.v	GO:000467	04626(Plan	protein kina	5.792276	16.686224	0.110427	1.791063	11.821519	3.5633435	0.001925577	0.071098082	up	yes	Cysteine-ric	CRK41
Pavir.9NG774200.v	GO:000367	NA	homeobox p	1.485989	5.679171	0.0001	0.607721	11.788273	3.5592805	0.023069782	0.280699783	up	yes	Homeobox	BEL1
Pavir.9NG651400.v	NA	NA	0	184.10271	453.76852	1.759781	52.381226	11.781665	3.5584716	0.004950811	0.127009363	up	yes	NA	NA
Pavir.2NG353900.v	GO:000367	04075(Plan	camp-respo	6.013556	39.825657	0.062558	3.836695	11.755896	3.5553126	0.009951097	0.181831401	up	yes	ABSCISIC	ABF4
Pavir.7NG411800.v	GO:000367	NA	wound-indu	7.009391	34.90736	2.628915	0.947845	11.719196	3.5508017	0.000391279	0.023560148	up	yes	NA	NA
Pavir.9NG713900.v	NA	NA	0	13.029553	50.218584	0.149236	5.2927894	11.622169	3.5388074	0.005266384	0.130782442	up	yes	Protein CH	CHUP1;CHUP1
Pavir.3NG159100.v	GO:000509	04120(Ubic	rho gdp-dis	5.424191	8.369364	0.364925	0.823459	11.606985	3.5369213	0.000356151	0.021895072	up	yes	Rho GDP-d	GDI1

Pavir.7KG334800.v	GO:000573	00940(Pher	alcohol o-ac	58.208923	233.19247	0.170662	25.055914	11.551365	3.5299915	0.017614477	0.243207467	up	yes	Hydroxycin	HCT4
Pavir.1KG093600.v	GO:00098(	00940(Pher	cinnamyl al	248.66228	850.69244	12.784616	82.512589	11.536065	3.5280793	0.001299353	0.055164499	up	yes	Probable ci	CAD
Pavir.4NG171600.v	NA	NA	myosin heav	1.352978	17.351597	0.12263	1.50132	11.51795	3.5258121	0.005819713	0.137393622	up	yes	Protein POI	POLAR
Pavir.4KG339900.v	NA	NA	0	1.169269	3.401941	0.049657	0.349062	11.464741	3.5191318	0.000741256	0.037738765	up	yes	NA	NA
Pavir.5NG382000.v	GO:00039	01210(2-O:	acetylornith	2.1975	4.128352	0.334249	0.217948	11.455788	3.5180048	0.000292501	0.018948513	up	yes	Acetylornitl	AG118
Pavir.7NG351000.v	GO:000842	00500(Starc	beta-glucosi	5.0378402	27.624533	0.3545496	2.5067712	11.415139	3.5128765	0.001370169	0.057074691	up	yes	Beta-glucos	BGLU18;BGLU18
Pavir.6NG069800.v	GO:000382	00053(Asco	gdp-d-mann	9.544003	24.90069	0.447192	2.575199	11.396505	3.5105195	0.000385262	0.023235047	up	yes	GDP-mann	GME-1
Pavir.9NG447000.v	GO:000467	NA	5'-amp-activ	2.948597	106.3411	3.409758	6.218504	11.350926	3.5047381	0.004646664	0.122881305	up	yes	SNF1-relat	KINB1
Pavir.4NG293400.v	GO:000367	NA	crib domain	2.03021	9.255239	0.134694	0.863297	11.308167	3.4992932	0.002540244	0.083870448	up	yes	CRIB doma	RIC10
Pavir.2KG374200.v	GO:000662	NA	long-chain-a	0.918139	19.115259	0.475023	1.296773	11.306831	3.4991227	0.003638484	0.106425992	up	yes	Probable lo	AT4
Pavir.1NG407500.v	GO:000377	NA	dynein light	13.156465	5.491421	0.395141	1.255688	11.296074	3.4977495	0.000754414	0.038100161	up	yes	NA	NA
Pavir.6NG319400.v	GO:000563	04626(Plan	cleavage site	1.2579834	6.9299608	0.059969	0.6651792	11.291408	3.4971535	0.005579417	0.134904319	up	yes	RPM1-inter	RIN4;RIN4;RIN4
Pavir.9NG033400.v	NA	NA	0	2.656945	1.21493	0.0001	0.343586	11.265734	3.4938694	0.018240609	0.246847474	up	yes	NA	NA
Pavir.9KG387500.v	GO:000577	NA	cobra-like p	400.11014	766.70929	0.281371	103.41083	11.252721	3.492202	0.025781545	0.297966779	up	yes	COBRA-lik	BC1
Pavir.1KG315500.v	NA	NA	mitochondri	2.359383	69.814095	1.214299	5.214271	11.226988	3.488899	0.00526153	0.1307627	up	yes	NA	NA
Pavir.9NG366500.v	GO:000455	04075(Plan	xyloglucan:	3.628286	14.898005	0.321022	1.337412	11.170955	3.4816806	0.000650803	0.034295409	up	yes	Probable xy	XTH28
Pavir.3KG370000.v	GO:00046(	00940(Pher	peroxidase (	33.075413	38.32716	0.207724	6.199349	11.144336	3.4782387	0.004089654	0.114160909	up	yes	Peroxidase	PRX74
Pavir.9NG307500.v	GO:001602	00941(Flav	flavonoid 3'	10.908053	66.411577	0.0001	6.943174	11.135904	3.4771467	0.04369121	0.394807564	up	yes	Flavonoid 3	CYP75B4;CYP75B4
Pavir.7NG381500.v	NA	NA	0	41.535938	144.95203	3.632237	13.137167	11.120727	3.4751792	0.000275741	0.01820809	up	yes	NA	NA
Pavir.7KG402900.v	GO:000467	00520(Ami	non-specific	2.37025	6.818429	0.120483	0.707245	11.101085	3.4726288	0.002979934	0.093071544	up	yes	Cysteine-ric	CRK10
Pavir.8KG043800.v	GO:000467	03018(RNA	dirigent-like	3.047792	26.621645	0.451464	2.222572	11.095377	3.4718868	0.001557272	0.061917697	up	yes	Cysteine-ric	CRK7
Pavir.3KG108900.v	GO:000588	NA	eama-like tr	1.106116	12.506181	0.3530169	0.879563	11.043744	3.4651574	0.002083965	0.073868681	up	yes	WAT1-rela	At5g47470;At5g47470
Pavir.9KG476000.v	NA	NA	0	13.649211	71.77099	0.5953029	7.1451313	11.035583	3.4640909	0.003381215	0.101309708	up	yes	Protein CHI	CHUP1;CHUP1;CHUP1
Pavir.9NG548100.v	GO:000449	00905(Bras	cytochrome	82.377953	396.879	0.513467	43.037415	11.004529	3.4600255	0.016272526	0.234255793	up	yes	Cytochrom	CYP709B2
Pavir.2NG602600.v	GO:000455	00500(Starc	glucan endo	13.478168	33.134642	0.1451226	4.0931331	10.998111	3.4591839	0.003693233	0.107064137	up	yes	Glucan end	At3g13560;At3g13560
Pavir.9KG297400.v	GO:001602	00941(Flav	flavonoid 3'	29.401247	252.18509	0.085795	25.551624	10.983412	3.4572544	0.032841164	0.339554498	up	yes	Flavonoid 3	CYP75B4
Pavir.9KG125100.v	NA	NA	0	1.694149	1.443417	0.223483	0.062632	10.966101	3.4549788	0.000347096	0.021578359	up	yes	NA	NA
Pavir.1KG062200.v	GO:000013	NA	polysacchar	153.0089	290.54419	0.688429	39.80035	10.954963	3.4535127	0.009606361	0.179037603	up	yes	Protein IRR	IRX15
Pavir.9NG075300.v	NA	NA	0	3.38282	8.979431	0.580307	0.554061	10.897919	3.4459808	0.000226116	0.015503418	up	yes	NA	NA
Pavir.5NG346900.v	GO:000557	04144(End	zinc finger f	8.266578	30.002991	0.210791	3.30453	10.886508	3.4444693	0.00260469	0.085157899	up	yes	GDSL ester	At1g09390
Pavir.9KG293200.v	NA	NA	0	1.589346	2.812296	0.29812	0.110071	10.78329	3.4307255	0.002608701	0.085157899	up	yes	NA	NA
Pavir.1NG006300.v	GO:000573	04141(Prot	membrane a	70.74222	105.73783	1.9438942	14.441812	10.770366	3.4289953	0.001032447	0.047093577	up	yes	Protein disu	PDIL1-4;PDIL1-4
Pavir.4KG307200.v	GO:000419	NA	aspartyl pro	8.142811	49.970699	0.14715	5.256107	10.755274	3.4269724	0.008299172	0.165424299	up	yes	Aspartyl pr	At5g10770
Pavir.5KG587400.v	GO:000588	01200(Cart	eama-like tr	4.524676	6.434087	0.314391	0.705869	10.741147	3.4250762	0.000253856	0.017028678	up	yes	WAT1-rela	At5g07050
Pavir.7NG010800.v	GO:00095(	00941(Flav	anthocyanid	11.476215	22.452368	0.335044	2.824734	10.737648	3.4246061	0.000988664	0.045974104	up	yes	Anthocyani	ANR
Pavir.1KG385200.v	GO:000573	NA	glutaredoxi	1.083695	43.092205	1.86793	2.254785	10.715245	3.4215929	0.005065564	0.128340138	up	yes	Glutaredoxi	GRXC4
Pavir.9KG365200.v	GO:001678	NA	2,6-dioxo-6	10.329547	11.509062	0.567061	1.475802	10.690198	3.4182166	3.69432E-05	0.003977267	up	yes	Probable E3	XBOS33
Pavir.8KG230100.v	GO:00040(	NA	aluminum i	5.077308	69.429276	1.582885	5.399903	10.670034	3.4154928	0.002492159	0.08257308	up	yes	NA	NA
Pavir.3NG018100.v	NA	NA	0	1.83912	3.294826	0.230362	0.251323	10.658306	3.4139062	0.001260914	0.054020874	up	yes	NA	NA
Pavir.4NG154700.v	NA	NA	dnaj homol	20.208443	283.31906	3.780945	24.769627	10.631223	3.4102357	0.003959612	0.111692581	up	yes	NA	NA
Pavir.5KG166600.v	GO:000487	NA	rapid alkali	103.40731	211.23941	1.396405	28.262758	10.608753	3.4071831	0.003966911	0.111780454	up	yes	Rapid alkal	RALF
Pavir.2KG578800.v	GO:000374	03013(RNA	translation f	4.126029	11.820954	0.257134	1.247287	10.60008	3.4060033	0.000778823	0.038946672	up	yes	Elongation	REFA1
Pavir.5KG128600.v	GO:000557	00514(Oth	glycosyltran	122.61646	48.669868	1.635602	14.635263	10.52718	3.3960472	0.001974765	0.07185437	up	yes	Alpha-1,3-a	XAT3
Pavir.7KG405300.v	GO:000367	NA	wound-indu	3.420503	18.256676	0.89027	1.170683	10.518037	3.3947935	0.001166804	0.051215323	up	yes	NA	NA
Pavir.6NG266900.v	GO:000382	03410(Base	3-methylad	216.84084	767.73706	0.472119	93.139824	10.517653	3.3947408	0.024077295	0.286555651	up	yes	NA	NA
Pavir.8KG019500.v	GO:00046(	00940(Pher	peroxidase :	1.003276	1.370656	0.0001	0.22577	10.51017	3.393714	0.018138486	0.246301979	up	yes	Peroxidase	GSVIVT00023967001
Pavir.8KG167000.v	GO:00016(	00010(Glyc	alcohol deh	41.213203	134.84457	6.807368	9.954846	10.503253	3.3927643	0.000203614	0.014407479	up	yes	Alcohol del	ADH2
Pavir.8NG009700.v	GO:00046(	01230(Bios	arogenate d	2.04434	17.482298	0.246239	1.620673	10.459324	3.3867177	0.004682909	0.123006906	up	yes	Arogenate c	TYRAAT2
Pavir.5KG074300.v	GO:001678	NA	alpha/beta l	11.628859	17.948513	0.0001	2.83131	10.446164	3.3849013	0.025548473	0.296006694	up	yes	NA	NA
Pavir.8KG349800.v	GO:000462	NA	ca2+-indep	1.140327	38.141628	1.121531	2.639633	10.444095	3.3846156	0.005046667	0.128340138	up	yes	Patatin-like	PLP1
Pavir.3KG532600.v	GO:000955	NA	burp domai	4.612831	18.236008	0.420944	1.769324	10.431983	3.3829415	0.000941493	0.044608736	up	yes	BURP dom	BURP13
Pavir.7KG310200.v	NA	NA	0	30.846578	199.67873	0.063848	22.078027	10.411282	3.3800758	0.033438523	0.342863091	up	yes	Protein SPI	Os04g0578300;Os04g0578300
Pavir.2KG092800.v	GO:001526	NA	mitochondri	2.289935	10.982059	0.517288	0.760687	10.385175	3.3764536	0.001422481	0.05846665	up	yes	NA	NA
Pavir.2NG628900.v	GO:000563	03013(RNA	aaa atpase//	2.337654	3.376817	0.311032	0.239801	10.374235	3.3749331	0.0001017	0.008665437	up	yes	ATPase fan	FIGL1
Pavir.1NG468400.v	GO:004665	NA	plastocyani	25.383793	36.965584	1.118586	4.898634	10.361824	3.3732061	0.000159051	0.012072757	up	yes	Blue coppe	-
Pavir.9NG746200.v	GO:004316	NA	ring finger c	6.981178	32.055809	0.33775	3.430994	10.358089	3.372686	0.002350919	0.079434433	up	yes	RING-H2 f	ATL47
Pavir.2KG400300.v	NA	NA	0	1.5891604	7.5536745	0.056596	0.8262461	10.356138	3.3724141	0.006120882	0.141865055	up	yes	NA	NA
Pavir.3KG311300.v	GO:000367	NA	protein of u	17.7297	8.623156	0.084693	2.478287	10.282115	3.3620652	0.00545871	0.133615697	up	yes	NA	NA
Pavir.5NG134500.v	GO:000557	00514(Oth	glycosyltran	7.365853	6.548637	0.09673	1.258756	10.265314	3.3597059	0.001990946	0.072276139	up	yes	Beta-1,2-xy	XYXT1
Pavir.1KG048600.v	GO:000455	00500(Starc	endo-1,4-be	20.175737	16.723202	0.085351	3.510474	10.261606	3.3591846	0.005451768	0.133532772	up	yes	Endoglucan	Os02g0151300
Pavir.9NG499600.v	GO:001678	NA	2,6-dioxo-6	28.846926	66.663358	1.4521572	7.8623781	10.253897	3.3581004	0.000705311	0.036402001	up	yes	NA	NA
Pavir.8NG275300.v	NA	NA	protein of u	1.104942	1.491176	0.0001	0.253508	10.236735	3.3556838	0.02343722	0.281789387	up	yes	NA	NA
Pavir.1NG465700.v	GO:000552	04141(Prot	ubiquitin-co	1.250288	1.367818	0.0001	0.255805	10.230773	3.3548433	0.023785374	0.284704632	up	yes	Ubiquitin-c	UBC11
Pavir.5KG646700.v	GO:000367	04712(Circ	homeodoma	1.994801	1.087773	0.0001	0.301915	10.206692	3.3514434	0.025447903	0.295794776	up	yes	Transcripti	MYB98
Pavir.9NG472700.v	NA	NA	0	1.166596	3.801116	0.486664	0.0001	10.205586	3.3512872	0.022626125	0.277230089	up	yes	NA	NA
Pavir.7KG364600.v	GO:000538	NA	vacuolar iro	3.190261	8.762228	0.0001	1.172771	10.190796	3.3491948	0.030262593	0.324966107	up	yes	Vacuolar ir	Os04g0686800
Pavir.8KG336400.v	GO:000367	NA	protein of u	11.877721	6.331952	0.823964	0.96431	10.18282	3.3480653	6.98828E-05	0.006647362	up	yes	NA	NA
Pavir.1NG080500.v	NA	NA	0	6.403758	18.139257	0.310958	2.102267	10.170214	3.3462782	0.005085453	0.128459391	up	yes	NA	NA
Pavir.9NG099600.v	NA	NA	0	1.491492	5.432678	0.264757	0.419777	10.115159	3.338447	0.001903835	0.070545156	up	yes	NA	NA
Pavir.5KG709500.v	NA	NA	0	2.225182	23.168762	0.0001	2.510728	10.113773	3.3382494	0.046015262	0.40452303	up	yes	NA	NA
Pavir.5NG105200.v	NA	NA	0	2.225866	1.860777	0.104226	0.300105	10.107172	3.3373075	0.002862871	0.090664626	up	yes	NA	NA
Pavir.7KG228500.v	GO:000551	00940(Pher	shikimate o	45.963776	212.73328	6.542589	19.107729	10.08553	3.334215	0.000998103	0.046014367	up	yes	Hydroxycin	HCT1
Pavir.7KG201500.v	GO:000333	NA	amino acid	4.773582	10.963772	0.16607	1.396535	10.07123	3.332168	0.001660999	0.064442719	up	yes	Probable ar	AAP7
Pavir.4NG007500.v	GO:000588	01200(Cart	eama-like tr	13.441088	4.2833674	0.0001	1.7614362	10.061931	3.3308353	0.035045477	0.352186433	up	yes	WAT1-rela	At3g30340;At3g30340;At3g30340
Pavir.5KG604300.v	GO:000579	NA	gdsl/sgnh-li	16.184896	9.650999	0.303865	2.263956	10.061408	3.3307603	0.000664623	0.034828272	up	yes	Protein ESI	ESK1;ESK1
Pavir.8KG034700.v	GO:000578	00901(Indo	strictosidin	1.492755	5.143412	0.255845	0.404515	10.049317	3.3290256	0.000322941	0.020499212	up	yes	Protein STI	SSL10
Pavir.4KG373100.v	NA	NA	0	2.214084	31.087593	1.836557	1.489779	10.011519	3.323589	0.004105206	0.114255493	up	yes	NA	NA
Pavir.5KG339000.v	GO:000484	NA	fetal globin-	2.256879	11.702747	0.094806	1.306743	9.9601412	3.3161662	0.00556794	0.134904319	up	yes	Probable E3	XBOS36
Pavir.2KG274000.v	GO:000098	00940(Pher	nad depend	33.231815	84.260826	1.138606	10.664625	9.9542779	3.3153167	0.002116921	0.074474785	up	yes	Cinnamoyl-	CCR1
Pavir.5NG344500.v	GO:000447	01230(Bios	s-adenosyln	350.19385	2016.1258	43.882359	194.02105	9.9465563	3.3141971	0.00246014	0.081953792	up	yes	S-adenosyl	SAM2;SAM2
Pavir.4KG387000.v	GO:000467	04626(Plan	non-specific	3.128221	1.678659	0.0001	0.485957	9.8895397	3.3059034	0.029024375	0.31784694	up	yes	Probable re	At5g57670
Pavir.3KG104400.v	GO:000563	NA	act domain-	4.224978	52.292591	2.078326	3.638459	9.8862506	3.3054235	0.001455964	0.059397321	up	yes	ACT domai	ACR9

Pavir.5KG186500.v	NA	NA	0	3.6364061	9.831465	0.2769747	1.0853844	9.8856986	3.3053429	0.000309105	0.01985299	up	yes	NA	NA
Pavir.5KG118600.v	GO:000467	00520(Ami	non-specific	2.53266	2.340934	0.365	0.128582	9.8739298	3.3036244	0.001032792	0.047093577	up	yes	Cysteine-ric	CRK41
Pavir.8KG103600.v	GO:000013	00790(Fola	beta-1,4-xyl	30.109842	31.616066	0.054313	6.21305	9.8487846	3.2999457	0.013894126	0.214303781	up	yes	Probable gl	Os01g0926700
Pavir.1KG022400.v	GO:001014	00561(Glyc	glycerol-3-p	4.174564	4.222006	0.602031	0.250558	9.8483208	3.2998778	0.000341017	0.021329717	up	yes	Glycerol-3-	GPAT4
Pavir.7NG306700.v	NA	NA	0	1.686754	5.19838	0.0001	0.699406	9.842852	3.2990764	0.032656407	0.338729464	up	yes	NA	NA
Pavir.5NG135400.v	GO:000557	00514(Othe	glycosyltran	37.815496	32.804797	0.330879	6.8524183	9.8311806	3.2973647	0.004398584	0.118484849	up	yes	Alpha-1,3-a	XAT2;XAT2
Pavir.1NG479700.v	GO:000367	04075(Plan	saur family	2.396466	13.296172	0.286698	1.312369	9.8136213	3.2947856	0.004397854	0.118484849	up	yes	NA	NA
Pavir.5NG488800.v	GO:000577	NA	n-acetylated	19.236141	63.303719	0.029865	8.382021	9.8122894	3.2945898	0.029428969	0.320224347	up	yes	Probable gl	LAMP1
Pavir.5KG495400.v	GO:004316	NA	ring/u-box c	46.400293	45.607503	0.1922356	9.20378	9.7922141	3.2916351	0.008984717	0.173297114	up	yes	E3 ubiquiti	At1g63170;At1g63170
Pavir.9NG183600.v	GO:000551	03015(mRN	iq calmodul	2.1536356	11.354164	0.3642344	1.0187227	9.7673305	3.2879643	0.001203586	0.052340665	up	yes	Protein IQ-I	IQD1;IQD1
Pavir.5NG631800.v	GO:000561	04144(Endo	cupin doma	12.632991	8.732924	0.150841	2.03944	9.7548739	3.2861232	0.003528283	0.104962919	up	yes	Germin-like	Os01g0952100
Pavir.1NG117900.v	NA	NA	domain of u	31.693449	105.9174	3.673044	10.465912	9.7327445	3.2828467	0.000527531	0.029277606	up	yes	NA	NA
Pavir.2NG170500.v	GO:000419	04145(Phag	cysteine pro	2.602878	16.310455	0.853602	1.093328	9.7144391	3.2801307	0.000780591	0.038949746	up	yes	Senescence-	SAG12
Pavir.8NG077300.v	NA	NA	0	2.850216	9.594472	0.398939	0.882521	9.7113355	3.2796697	0.001708503	0.065637595	up	yes	NA	NA
Pavir.6KG397000.v	GO:000573	NA	bcl2-associa	246.15765	346.42725	20.938686	40.170956	9.6970769	3.2775499	0.000210559	0.01473245	up	yes	BAG family	BAG1;BAG1
Pavir.5NG534500.v	GO:000460	00940(Pher	peroxidase (	7.521287	11.872254	0.187604	1.812772	9.6949478	3.2772331	0.001688167	0.065122682	up	yes	Peroxidase	PER56
Pavir.7KG413800.v	GO:000950	00941(Flav	anthocyanid	14.687389	38.990692	0.0001	5.53904	9.6906886	3.2765992	0.045143894	0.400541715	up	yes	Anthocyani	ANR
Pavir.2NG044900.v	GO:000020	00053(Asco	e3 ubiquitin	1.1526829	5.4547034	0.1930573	0.4893939	9.6818442	3.2752819	0.001778928	0.067466606	up	yes	E3 ubiquiti	RHF2A;RHF2A
Pavir.9KG037100.v	GO:000035	03040(Spli	pre-mrna-sp	1.271581	6.829672	0.347928	0.489495	9.6740273	3.2741166	0.002460381	0.081953792	up	yes	NA	NA
Pavir.9NG531600.v	GO:000577	NA	cobra-like p	95.329971	105.99589	0.250912	20.621677	9.6454665	3.269851	0.015252435	0.226367538	up	yes	COBRA-lik	BC1
Pavir.1NG022300.v	GO:000455	00500(Star	endo-1,4-be	12.872665	9.589634	0.021432	2.310588	9.6321211	3.2678535	0.013341752	0.209368875	up	yes	Endoglucan	Os02g0151300
Pavir.4KG339800.v	NA	NA	0	1.109901	2.840646	0.195848	0.215933	9.5938059	3.2621033	0.002639034	0.085775469	up	yes	NA	NA
Pavir.1KG062100.v	GO:000367	03008(Ribo	single-stran	62.731236	94.274734	9.298369	7.094497	9.5777011	3.2596794	8.91395E-05	0.007955435	up	yes	Single-stran	WHY2
Pavir.8NG030700.v	GO:000981	00942(Anth	cyanidin 3-c	5.400362	28.452919	0.233745	3.305822	9.5642436	3.2576509	0.005257445	0.1307627	up	yes	Cyanidin 3-	5GT
Pavir.2KG409200.v	NA	NA	0	32.039902	33.831635	0.391529	6.499173	9.5594813	3.2569323	0.002553316	0.084080415	up	yes	NA	NA
Pavir.2KG016900.v	GO:001507	NA	osmotic stre	3.332236	9.044059	0.0001	1.294804	9.5576931	3.2566624	0.038284205	0.368928551	up	yes	Potassium t	HAK22
Pavir.9KG561000.v	NA	NA	0	5.585478	10.026771	0.034039	1.599481	9.5574275	3.2566224	0.010614138	0.186793906	up	yes	Salt toleran	OsI_16820
Pavir.2NG440400.v	NA	NA	methyltrans	22.295464	53.219418	0.450352	7.453928	9.5536699	3.256055	0.004669189	0.122881305	up	yes	NA	NA
Pavir.5KG744700.v	GO:000467	03022(Basa	cell division	9.9577153	13.752296	0.094951	2.3923214	9.532535	3.2528599	0.0044284	0.11901879	up	yes	Probable se	At1g54610;At1g54610;At1g54610;At1g54610
Pavir.6NG279000.v	GO:004665	NA	plastocyani	44.783466	16.50495	0.36006	6.097454	9.4910233	3.2465636	0.003695603	0.107064137	up	yes	Blue copper	-
Pavir.3NG174200.v	GO:000557	NA	cupin (cupi	1.5053201	16.444252	0.3404924	1.5508306	9.4904845	3.2464817	0.004396201	0.118484849	up	yes	Glutelin typ	GLUA2;GLUA2
Pavir.2KG557500.v	GO:000872	NA	gtp diphosp	2.114504	23.166626	0.136261	2.532682	9.4723379	3.2437206	0.010734322	0.187853135	up	yes	Probable G	RSH2
Pavir.6KG385600.v	GO:000367	NA	stress up-re	2.0446031	5.8812273	0.2800007	0.5580996	9.4569001	3.2413673	0.000499089	0.028412543	up	yes	NA	NA
Pavir.6KG400000.v	GO:000367	00190(Oxic	myb domair	3.170043	56.424751	3.083779	3.224843	9.4465628	3.2397895	0.002998774	0.093426977	up	yes	Protein OD	ODO1
Pavir.2KG339200.v	GO:000983	00500(Star	x-box trans	286.80924	703.93182	1.430397	103.51804	9.4402654	3.2388274	0.017655839	0.243207467	up	yes	Cellulose sy	CESA9
Pavir.1NG553800.v	GO:000467	04626(Plan	interleukin-	2.3917411	7.6999839	0.354452	0.7146873	9.4391106	3.2386509	0.00055091	0.030306501	up	yes	Calmodulin	CRCK2;CRCK2
Pavir.7KG235900.v	GO:000367	NA	senescence	7.19705	47.270958	1.480404	4.323428	9.3848354	3.2303314	0.001041799	0.047446677	up	yes	NA	NA
Pavir.6NG077300.v	NA	NA	0	5.168816	127.0564	0.213477	13.892342	9.3738061	3.228635	0.031710354	0.333684044	up	yes	NA	NA
Pavir.1NG151600.v	GO:000983	00500(Star	x-box trans	232.75205	648.5213	0.844646	93.228203	9.3679883	3.2277393	0.023194424	0.281068051	up	yes	Cellulose sy	CESA9
Pavir.9KG061200.v	GO:000484	NA	inhibitor of	6.350802	36.889782	0.768528	3.857989	9.3462499	3.2243876	0.002115671	0.074474785	up	yes	Probable B	BRG3
Pavir.2NG429900.v	NA	NA	0	15.725439	0.8830027	1.1719224	0.6063721	9.3395337	3.2233505	0.0030369	0.094224159	up	yes	NA	NA
Pavir.7KG261700.v	GO:000419	NA	legumain /	1.6754429	13.507857	1.005254	0.621628	9.3327604	3.2223039	0.001777265	0.067466606	up	yes	Vacuolar-p	OsI_16797;OsI_16797
Pavir.4KG029200.v	NA	NA	cysteine-ricl	14.196347	16.200881	1.0276814	2.2331155	9.3220244	3.2206433	5.59698E-05	0.005580645	up	yes	NA	NA
Pavir.2NG556800.v	NA	NA	0	39.323109	174.92241	0.456386	22.545361	9.314315	3.2194497	0.015713268	0.229994002	up	yes	NA	NA
Pavir.5KG772400.v	NA	NA	0	3.398196	2.216556	0.293146	0.309715	9.3135101	3.219325	0.002323178	0.078597368	up	yes	NA	NA
Pavir.8NG306600.v	NA	NA	metallothioi	114.83823	819.63525	8.900379	91.653145	9.2932943	3.2161901	0.007274384	0.156441785	up	yes	Metallothio	ERR26
Pavir.2KG548600.v	GO:000402	01200(Carb	alcohol deh	2.2319803	5.0888069	0.0001	0.7883924	9.2845373	3.21483	0.035056675	0.352186433	up	yes	Alcohol del	At5g42250;At5g42250
Pavir.8NG022800.v	GO:000029	03008(Ribo	5'-3' exoribo	19.439983	89.186612	2.5232946	9.178659	9.2827743	3.214556	0.001662432	0.064442719	up	yes	5'-3' exorib	XRN4;XRN4;XRN4;XRN4
Pavir.4NG191300.v	GO:000013	00510(N-G	beta-1,4-xyl	16.626734	27.257219	0.359184	4.372713	9.2740719	3.2132029	0.001999538	0.072473063	up	yes	Probable gl	Os01g0926700
Pavir.1NG318200.v	GO:000981	00908(Zeat	glucosyl/glu	1.415039	3.309594	0.0001	0.5093835	9.2733778	3.2130949	0.036429082	0.358687078	up	yes	7-deoxyloga	UGT85A24;UGT85A24
Pavir.4KG282000.v	GO:000551	NA	uncharacteri	8.844458	30.613087	0.616811	3.642468	9.2639024	3.2116201	0.001322734	0.055795265	up	yes	Pirin-like p	At1g50590
Pavir.9KG216400.v	GO:000588	NA	inorganic pl	3.948557	5.127707	0.353067	0.627184	9.2591224	3.2108755	0.000173032	0.012848477	up	yes	Probable in	PHT1-8
Pavir.5KG021400.v	NA	NA	0	1.530021	124.39722	1.582629	12.049945	9.2372317	3.2074606	0.025167803	0.294261858	up	yes	NA	NA
Pavir.6NG147700.v	GO:000980	00940(Pher	nad depende	131.00916	534.25165	24.246033	48.01659	9.2061535	3.2025985	0.000924919	0.044157543	up	yes	Cinnamoyl-	CCR1
Pavir.2NG630300.v	NA	NA	0	6.335499	26.637915	0.865158	2.72729	9.1785362	3.1982641	0.001400096	0.057808561	up	yes	NA	NA
Pavir.2NG417500.v	GO:000551	NA	uncharacteri	4.07009	4.839871	0.376046	0.594787	9.1776454	3.1981241	0.002088612	0.073943863	up	yes	NA	NA
Pavir.7KG007300.v	GO:000538	04075(Plan	manganese	2.5342449	9.3918248	0.1238841	1.1761701	9.1735172	3.197475	0.003882296	0.110347156	up	yes	Metal trans	NRAMP4;NRAMP4;NRAMP4;NRAMP4;NRAMP4;NRAMP4
Pavir.2NG169700.v	GO:000906	00430(Taur	cysteamine	3.1609728	19.948605	0.0937874	2.4460603	9.0988044	3.185677	0.011849272	0.19717125	up	yes	Plant cystei	PCO5;PCO5;PCO5;PCO5
Pavir.9KG635100.v	GO:000455	NA	glycosyl hyc	4.659528	8.009157	0.142645	1.25097	9.09052	3.1843628	0.002271986	0.077785179	up	yes	Endo-1,4-b	At4g33820
Pavir.5NG037700.v	GO:000370	NA	nac domain	14.216478	31.098701	0.810683	4.181499	9.077229	3.1822519	0.000697483	0.03604747	up	yes	NAC doma	NAC075
Pavir.9NG568900.v	NA	NA	0	7.251595	51.592365	2.598351	3.893145	9.0647764	3.1802714	0.000779493	0.038946672	up	yes	NA	NA
Pavir.1KG267600.v	GO:000367	NA	zinc-finger	4.353207	14.361124	0.104331	1.961479	9.0590766	3.179364	0.009357004	0.176596389	up	yes	FCS-Like Z	FLZ2
Pavir.6NG125100.v	GO:000443	00562(Inos	phosphatidy	3.868454	1.963903	0.142662	0.501451	9.0548661	3.1786933	0.002612874	0.085220073	up	yes	Type IV inc	IP5P9
Pavir.2NG335600.v	GO:000563	03410(Base	dna-3-methy	21.191257	46.706772	0.061422	7.465181	9.0210722	3.1732989	0.021149952	0.267564696	up	yes	NA	NA
Pavir.7KG405000.v	GO:000367	NA	wound-indu	4.626479	17.132462	1.293789	1.118689	9.0193324	3.1730207	0.000760258	0.038342492	up	yes	NA	NA
Pavir.5KG500300.v	GO:000456	00520(Ami	chitinase//su	1.253126	1.493163	0.063395	0.241226	9.015429	3.1723961	0.004710633	0.12336531	up	yes	Acidic endo	-
Pavir.8NG240800.v	GO:000367	00620(Pyru	plant protei	5.1031993	2.54328	0.0001	0.8495487	8.999578	3.1698573	0.038175342	0.368162761	up	yes	NA	NA
Pavir.9KG193600.v	GO:000983	00500(Star	cellulose sy	163.56302	291.6243	0.670787	50.047298	8.9748522	3.1658882	0.018835343	0.250779717	up	yes	Cellulose sy	CESA7
Pavir.4NG019200.v	GO:000484	NA	predicted e3	1.308779	6.163986	0.110059	0.724439	8.9548028	3.1626617	0.006514954	0.14647695	up	yes	Probable E	XBOS36
Pavir.5KG578100.v	GO:000367	NA	atp-depende	1.360225	15.451758	0.381194	1.49717	8.9503328	3.1619413	0.005105802	0.128751867	up	yes	Sulfite expo	At4g21250
Pavir.5NG034300.v	GO:000097	00190(Oxic	myb-like dn	1.239564	16.427973	0.598042	1.378489	8.9386592	3.1600584	0.005202398	0.130321762	up	yes	Transcriptic	MYB36
Pavir.5KG758700.v	GO:000399	NA	inactive pur	27.349926	27.265352	1.0474569	5.0638027	8.9368283	3.1597629	0.000396347	0.023764777	up	yes	Probable in	PAP16;PAP16
Pavir.5KG442400.v	GO:000557	00511(Othe	zinc finger f	1.376214	5.625024	0.022633	0.761626	8.9272013	3.158208	0.013872979	0.214288516	up	yes	GDSL ester	At1g28600
Pavir.9KG269300.v	NA	NA	0	4.563813	10.176355	0.451173	1.20332	8.9091752	3.1552919	0.001358007	0.056946866	up	yes	NA	NA
Pavir.1NG019800.v	NA	NA	0	107.32974	80.867752	0.294735	20.918581	8.8716679	3.1492054	0.016823991	0.238475181	up	yes	Protein IRR	IRX15
Pavir.3KG434800.v	GO:000484	NA	ring finger p	9.196245	13.855147	0.1520762	2.44864	8.8634784	3.147873	0.003455169	0.102950884	up	yes	Protein LA	LAX2;LAX2;LAX2;LAX2;LAX2;LAX2;LAX2
Pavir.3KG016900.v	GO:000578	00901(Indo	strictosidine	3.427387	4.544039	0.0522047	0.8480524	8.8546104	3.1464288	0.004466958	0.119744441	up	yes	Protein STF	SSL10;SSL10;SSL10;SSL10
Pavir.3NG063900.v	GO:000418	00940(Pher	serine prote	4.751464	19.93346	0.252371	2.53622	8.8521135	3.1460219	0.003803054	0.109139838	up	yes	Serine carbo	CBP1
Pavir.6KG256000.v	NA	NA	0	29.673723	53.251629	0.551423	8.816916	8.85166	3.145948	0.006591835	0.147204491	up	yes	NA	NA
Pavir.9NG438600.v	GO:000013	00510(N-G	beta-1,4-xyl	13.324278	23.23455	0.223242	3.90857	8.8481344	3.1453733	0.004354074	0.118204918	up	yes	Probable gl	Os01g0926700
Pavir.9NG229500.v	GO:000685	NA	mate efflux	1.8133323	6.8629447	0.088899	0.8933041	8.8334854	3.1429828	0.004685007	0.123006906	up	yes	Protein DE	DTX40;DTX40
Pavir.3KG247300.v	GO:000550	00260(Glyc	amine oxida	8.132066	26.09934	0.160833	3.720921	8.8185408	3.14054	0.00718113	0.15551388	up	yes	Primary am	At1g62810

Pavir.2NG591500.v4	GO:000467	04626(Plan	leucine rich	6.8936535	2.8986569	0.5243707	0.5868099	8.8125279	3.1395559	0.000215803	0.014932518	up	yes	Protein STF	SRF6;SRF6;SRF6
Pavir.5NG276400.v4	GO:000449	00905(Bras	cytochrome	1.041615	2.985159	0.0001	0.457359	8.8024807	3.1379102	0.041224891	0.3820085	up	yes	Secologanin	CYP72A1
Pavir.5NG433800.v4	GO:000367	NA	protein of u	227.10988	473.08432	5.700976	73.887276	8.797708	3.1371277	0.004289179	0.117219331	up	yes	NA	NA
Pavir.9NG512500.v4	GO:004665	NA	plastocyanin	17.740662	24.701509	0.883156	3.958875	8.7653654	3.1318142	0.000828329	0.040643524	up	yes	Blue copper	-
Pavir.5NG202700.v4	GO:000563	NA	uncharacteri	1.599915	1.691055	0.0001	0.375609	8.759359	3.1308253	0.033233197	0.341834488	up	yes	Probable cy	LOGL7
Pavir.7NG028600.v4	GO:000002	00900(Terp	mrna turnov	7.6923455	39.216468	0.5549621	4.8010189	8.7582112	3.1306362	0.003770217	0.108633725	up	yes	Probable ph	-;Os01g0832000;-
Pavir.5NG167800.v4	NA	NA	0	1.7014336	6.5048511	0.2821801	0.6558691	8.7482456	3.1289937	0.001017137	0.046701331	up	yes	Protein argo	AGO4A;NA;NA;NA;NA
Pavir.7KG421900.v4	NA	NA	0	3.6051914	6.8697183	0.256997	0.941362	8.7410448	3.1278057	0.00402262	0.112540498	up	yes	NA	NA
Pavir.3KG214700.v4	GO:000382	00941(Flav	nad depende	7.3032454	29.25232	0.7158523	3.4836471	8.7047435	3.1218018	0.00147808	0.059909295	up	yes	Cinnamoyl-	SNL6;SNL6
Pavir.6KG311300.v4	GO:000462	NA	ca2+-indepe	10.707491	0.782272	0.033257	1.287293	8.7007406	3.1211382	0.031160083	0.330075502	up	yes	Patatin-like	PLP2
Pavir.6NG224100.v4	GO:000552	00360(Pher	trans-ferulo	63.991365	168.89778	4.0129166	22.766447	8.6965897	3.1204498	0.002296246	0.078221538	up	yes	Probable 4-	4CL3;4CL3
Pavir.6KG154400.v4	GO:000552	00360(Pher	trans-ferulo	164.0493	478.34036	7.314808	66.619446	8.6886609	3.1191339	0.004661459	0.122881305	up	yes	Probable 4-	4CL3
Pavir.2NG585800.v4	GO:000551	04626(Plan	non-specific	16.305637	33.109188	0.111316	5.576408	8.6879787	3.1190206	0.011661224	0.195931866	up	yes	Serine/threo	RIPK
Pavir.2NG381000.v4	NA	NA	0	17.507378	48.48806	1.336335	6.268897	8.6776364	3.1173021	0.000811711	0.04019688	up	yes	NA	NA
Pavir.2KG450600.v4	GO:000367	NA	genomic dna	30.628014	17.23296	0.0001	5.516067	8.6764911	3.1171117	0.045923248	0.403903134	up	yes	Vascular-re	VUP1
Pavir.8KG077700.v4	GO:000588	00860(Porp	very-long-ch	6.381471	25.014912	0.0001	3.619685	8.6735491	3.1166224	0.047528891	0.410907277	up	yes	Short-chain	TIC32
Pavir.9NG475600.v4	GO:000557	NA	pollen allerg	11.812159	5.349772	0.0001	1.978649	8.6731218	3.1165514	0.041950447	0.385759888	up	yes	Expansin-B	EXPB3
Pavir.9NG501500.v4	GO:000436	00480(Glut	glutathione	8.781908	18.089891	0.511597	2.600076	8.6358043	3.1103305	0.001174249	0.051384114	up	yes	Probable gl	GSTU1
Pavir.3KG454400.v4	NA	NA	0	1.472216	10.525724	0.550741	0.838702	8.6350718	3.1102082	0.00465698	0.122881305	up	yes	Uncharacter	At4g22758
Pavir.1KG407400.v4	NA	NA	0	1.409679	1.910625	0.148187	0.237334	8.6125114	3.106434	0.002489999	0.08257308	up	yes	NA	NA
Pavir.5NG447800.v4	GO:004316	NA	ring/u-box c	37.107506	56.353194	0.0864977	10.771663	8.6074156	3.1055801	0.023864229	0.285459792	up	yes	E3 ubiquitin	At1g63170;NA;NA
Pavir.5NG446500.v4	GO:000864	NA	rag1-activat	1.5608896	4.8812397	0.0001	0.7493165	8.5961937	3.103698	0.044661137	0.398557495	up	yes	Bidirectiona	SWEET2B;SWEET2B
Pavir.4NG122600.v4	GO:000588	01200(Carb	eama-like tr	8.030488	12.049242	0.066133	2.270746	8.5925416	3.1030849	0.008555484	0.168565915	up	yes	WAT1-rela	At1g44800
Pavir.1NG555000.v4	GO:004316	00563(Glyc	ring finger c	7.170087	27.172173	1.33119	2.669635	8.5837946	3.1016156	0.000623456	0.033133136	up	yes	RING-H2 f	ATL1
Pavir.3NG200800.v4	NA	NA	0	20.581785	28.136478	0.732708	4.951275	8.5711486	3.0994886	0.005625911	0.135154959	up	yes	Protein LAX	LAX2
Pavir.7KG017200.v4	GO:000573	00750(Vita	aldo-keto re	7.2306893	44.273314	1.6701953	4.346829	8.5597134	3.0975625	0.001220332	0.052824329	up	yes	Probable ald	Os04g0338000;Os04g0338000
Pavir.8KG155300.v4	GO:000521	NA	oligopeptide	21.378471	64.035591	0.408437	9.586713	8.5455508	3.0951735	0.009872577	0.181389904	up	yes	Protein NRT	NPF4.5
Pavir.6KG319400.v4	GO:004665	NA	plastocyanin	5.057766	4.166871	0.0001	1.080778	8.5343924	3.0932884	0.039601933	0.375463894	up	yes	Blue copper	-
Pavir.5NG066500.v4	GO:000449	00905(Bras	cytochrome	12.550261	2.2114015	0.0460158	1.6843417	8.5309895	3.0927131	0.017044921	0.240475103	up	yes	Cytochrome	CYP734A6;CYP734A6;CYP734A6
Pavir.8NG089600.v4	GO:001602	00380(Tryp	angelicin sy	4.016467	15.167859	0.08122	2.172352	8.5128525	3.0896426	0.013126548	0.208366623	up	yes	Cytochrome	CYP71A6
Pavir.1NG374600.v4	GO:000551	00910(Nitro	cytochrome	2.777625	5.448792	0.0001	0.967042	8.505904	3.0884646	0.042095239	0.386620619	up	yes	Cytochrome	CYTB5-E
Pavir.3NG037300.v4	GO:000098	NA	b-box zinc f	1.701246	5.997147	0.22111	0.684931	8.496738	3.0869091	0.003072827	0.095002339	up	yes	B-box zinc	BBX22
Pavir.3NG063700.v4	GO:000436	00350(Tyro	maleylaceto	1.3332464	25.471046	1.1365378	2.0208229	8.4894615	3.0856731	0.005380766	0.132397857	up	yes	Glutathione	GSTZ1;GSTZ1;GSTZ1
Pavir.2NG555600.v4	NA	NA	0	44.764404	138.664	1.330666	20.28002	8.4878567	3.0854003	0.00795677	0.162325709	up	yes	NA	NA
Pavir.9KG059200.v4	GO:000557	03010(Ribo	leucine rich	8.093596	30.132071	0.033753	4.47085	8.4859125	3.0850698	0.025411177	0.295687253	up	yes	Leucine-rich	LRX6
Pavir.1KG557700.v4	NA	NA	camp-respo	5.361946	31.775171	1.484177	2.904039	8.4629191	3.0811554	0.00216798	0.075726759	up	yes	NA	NA
Pavir.3NG019800.v4	GO:000588	NA	amino acid t	5.6727251	1.8159719	0.0643333	0.8213621	8.4551615	3.0798323	0.006529926	0.14647695	up	yes	Cationic am	CAT6;CAT6;CAT6;CAT6
Pavir.2NG007400.v4	GO:000333	NA	proline trans	2.6264085	7.9688074	0.0356574	1.2179528	8.4517628	3.0792523	0.013097697	0.208163554	up	yes	Probable pr	LOC Os07g01090;LOC Os07g01090;LOC Os07g01090;LOC Os07g01090;LOC Os07g01090
Pavir.2KG106400.v4	GO:000552	00561(Glyc	bcs1 aaa-typ	0.965046	13.08731	0.162187	1.501568	8.4461693	3.0782972	0.013598093	0.211824682	up	yes	AAA-ATPa	At3g28610;At3g28600
Pavir.5NG057800.v4	GO:005121	00402(Benz	oxidoreduct	6.11837	20.867725	1.425077	1.77608	8.4301067	3.0755509	0.003034775	0.094224159	up	yes	1-aminocyc	At5g59530
Pavir.9KG547900.v4	GO:000573	NA	heavy-metal	54.329071	140.42339	2.919177	20.204378	8.4222541	3.0742064	0.003075764	0.095002339	up	yes	Heavy meta	HIPP39
Pavir.9KG436700.v4	GO:000598	00500(Starc	sucrose synt	35.609238	115.11749	3.785779	14.19176	8.3841689	3.0676678	0.001896686	0.070349639	up	yes	Sucrose syn	SUS4
Pavir.9KG631100.v4	NA	NA	0	171.77223	387.6684	4.010636	62.784672	8.375448	3.0661664	0.007323905	0.156441785	up	yes	NA	NA
Pavir.9NG444300.v4	GO:000467	04626(Plan	protein kina	12.660137	26.300808	0.0606398	4.5965376	8.3657848	3.0645009	0.020297022	0.261260708	up	yes	Probable LI	At1g06840;At1g06840;At1g06840;At1g06840;At1g06840;At1g06840;At1g06840;At1g06840
Pavir.5KG622200.v4	GO:000550	00053(Asco	multi-coppe	282.36731	40.236744	1.222381	37.403816	8.3519497	3.062113	0.020468972	0.262664163	up	yes	Laccase-4 C	LAC4
Pavir.4KG403300.v4	GO:000382	NA	phosphosulf	5.393231	14.628413	0.516798	1.888838	8.3228069	3.0570702	0.001484418	0.060090369	up	yes	Protein HEA	HSA32
Pavir.7NG331100.v4	GO:000666	00514(Othe	kdel lys-asp	18.166712	33.293419	0.049157	6.137521	8.3178939	3.0562183	0.022442309	0.276105064	up	yes	NA	NA
Pavir.8NG281300.v4	GO:000333	NA	amino acid t	15.010295	13.658653	0.312858	3.138668	8.306166	3.0541827	0.00222729	0.076904503	up	yes	Amino acid	AAP3
Pavir.9KG526900.v4	NA	NA	0	5.395859	38.408199	0.685158	4.591785	8.3010292	3.0532902	0.004734566	0.123521137	up	yes	NA	NA
Pavir.9KG547600.v4	GO:000563	NA	transcriptio	3.346467	4.770675	0.145856	0.834058	8.2835249	3.0502448	0.007464675	0.157697172	up	yes	Transcriptio	OFP5
Pavir.6KG279900.v4	GO:000980	00940(Pher	nad depende	77.494255	678.17499	45.68362	45.603336	8.2779542	3.0492743	0.002876143	0.090734961	up	yes	Cinnamoyl-	CCR1
Pavir.7NG359300.v4	GO:000367	NA	filament-like	0.893337	26.87029	0.052235	3.3066248	8.2657892	3.0471526	0.042664356	0.389038382	up	yes	Filament-lik	FPP7;FPP7
Pavir.5KG578400.v4	GO:000460	00940(Pher	peroxidase (	2.961457	2.894514	0.094837	0.614194	8.2591184	3.0459878	0.004589356	0.122035291	up	yes	Peroxidase	PER56
Pavir.7KG241000.v4	GO:000842	00500(Starc	glycosyl hyd	1.735544	6.413172	0.183996	0.803229	8.2541629	3.0451219	0.004198843	0.115831961	up	yes	Beta-glucos	BGLU16
Pavir.7NG365500.v4	GO:000367	NA	mkiaa1688	10.402033	20.837969	0.089224	3.708598	8.2257678	3.0401504	0.017296294	0.241498946	up	yes	CRIB doma	RIC9
Pavir.5KG145100.v4	NA	NA	levansucrase	375.55634	79.225288	1.112163	54.244595	8.2154671	3.0383426	0.023287686	0.281238111	up	yes	NA	NA
Pavir.9NG307200.v4	GO:000983	00500(Starc	cellulose sy	313.57364	534.17609	1.347595	101.84982	8.2148346	3.0382315	0.023391034	0.281789387	up	yes	Cellulose sy	CESA7
Pavir.4NG312700.v4	NA	NA	domain of u	6.200793	10.483459	0.533577	1.498323	8.211158	3.0375857	0.003394117	0.101534354	up	yes	NA	NA
Pavir.1KG481200.v4	NA	NA	0	499.26703	610.71082	5.401162	129.8647	8.2058978	3.0366612	0.010026703	0.181875162	up	yes	NA	NA
Pavir.5NG202500.v4	GO:000367	NA	cotton fibre	6.366982	10.043305	0.380313	1.625131	8.1828697	3.0326069	0.001085487	0.048701743	up	yes	Pathogen-as	A70
Pavir.7KG416500.v4	GO:005121	00270(Cyst	oxidoreduct	1.997159	5.7655	0.090941	0.858327	8.177521	3.0316636	0.013633634	0.212114502	up	yes	Flavanone 3	F3H-2
Pavir.7NG446000.v4	GO:000552	04146(Pero	oxalate--coa	3.461748	10.004627	0.286312	1.36156	8.1719788	3.0306855	0.001848815	0.069119935	up	yes	Oxalate--Co	AAE3
Pavir.2NG515900.v4	GO:000367	NA	genomic dna	8.273619	10.723886	0.295091	2.032248	8.162758	3.0290567	0.003857633	0.110003727	up	yes	Vascular-re	VUP1
Pavir.1NG551600.v4	GO:000577	00531(Glyc	aquaporin tr	4.904119	20.076571	1.77062	1.293572	8.1524559	3.0272347	0.000347454	0.021578359	up	yes	Aquaporin I	PIP1-5;PIP1-5;PIP1-5
Pavir.7NG339000.v4	GO:000467	00500(Starc	protein kina	4.711107	4.925364	0.215086	0.967796	8.1466038	3.0261987	0.001609237	0.063114666	up	yes	L-type lecti	SIT2
Pavir.7NG381700.v4	NA	NA	0	5.632724	7.699927	0.636091	1.001081	8.1437082	3.0256859	0.001695486	0.065321851	up	yes	NA	NA
Pavir.7KG302500.v4	GO:000425	00240(Pyrin	rhomboid-re	43.440121	113.22295	0.402624	18.840628	8.141195	3.0252406	0.017905529	0.244744653	up	yes	RHOMBOI	RBL1
Pavir.7NG214400.v4	GO:000367	01200(Carb	ntkl-binding	133.36958	188.37071	0.850071	38.707706	8.1334271	3.0238634	0.016418195	0.235419518	up	yes	NA	NA
Pavir.9KG239900.v4	GO:000370	00750(Vita	homeobox-l	11.381536	74.309326	0.554884	9.981217	8.1330714	3.0238003	0.013214566	0.208896413	up	yes	Homeobox-	HOX15
Pavir.9KG409200.v4	NA	NA	0	1.054179	1.153609	0.0001	0.271544	8.1275051	3.0228126	0.033743843	0.343943783	up	yes	NA	NA
Pavir.2KG336800.v4	GO:000563	03410(Base	dna-3-methy	37.763641	57.466564	0.277698	11.452073	8.1186755	3.0212444	0.014085698	0.215619109	up	yes	NA	NA
Pavir.1KG490500.v4	GO:000367	NA	bzip protein	6.798495	2.277006	0.157259	0.960934	8.1162205	3.0208081	0.013176185	0.208801681	up	yes	Basic leucin	BZIP43
Pavir.1KG500500.v4	GO:000573	NA	nudix hydro	21.516747	8.966037	1.142561	2.613415	8.115809	3.0207349	0.000534082	0.029597456	up	yes	Nudix hydro	NUDT18
Pavir.9NG461600.v4	GO:000368	04712(Circ	dnaj homolo	14.613854	65.117172	3.463839	6.37868	8.1006728	3.0180417	0.000955315	0.045036469	up	yes	Transcriptio	MYBS2
Pavir.3NG218800.v4	GO:000487	NA	root phototr	12.878283	64.651344	2.6016	6.973437	8.0970577	3.0173978	0.001956191	0.07156763	up	yes	Root photot	RPT2
Pavir.1NG359200.v4	NA	NA	mitochondri	5.244094	46.641773	2.013813	4.397647	8.0926758	3.0166168	0.002316237	0.078562572	up	yes	NA	NA
Pavir.3KG207300.v4	NA	NA	0	12.537251	34.249973	1.043469	4.738022	8.0925879	3.0166011	0.001081865	0.048681336	up	yes	Serine carbo	SCPL50
Pavir.8NG121500.v4	GO:000406	NA	asparagine s	23.182989	125.93968	4.7870128	13.64602	8.0899695	3.0161343	0.002607653	0.085157899	up	yes	NA	NA
Pavir.3KG187400.v4	NA	NA	cysteine-rich	9.449471	190.94475	5.251328	19.524263	8.0883729	3.0158495	0.010947438	0.189203355	up	yes	NA	NA
Pavir.2KG117500.v4	NA	NA	0	151.05426	396.4743	5.165521	62.808483	8.05497	3.0098792	0.007070133	0.154535264	up	yes	NA	NA
Pavir.2KG322500.v4	GO:000579	NA	beta-1,3-n-a	17.228968	11.591875	0.827285	2.754889	8.045629	3.0082052	0.000536349	0.02963563	up	yes	Hydroxypro	GALT3
Pavir.5NG036400.v4	GO:000368	NA	transcriptio	11.130759	38.378029	0.357272	5.796621	8.0451168	3.0081134	0.007110588	0.154919423	up	yes	Transcriptio	KUA1

Pavir.9NG590300.v	GO:000573	00250(Alar	aminotransf	18.282888	71.846451	0.0610297	11.143453	8.0440431	3.0079208	0.039866155	0.376733162	up	yes	Alanine--gl	At3g08860;At3g08860;At3g08860
Pavir.5NG456400.v	GO:000467	04626(Plan	protein tyro	10.167804	13.718436	0.4754362	2.4990493	8.0303774	3.0054678	0.001092697	0.048934324	up	yes	LEAF RUS	LRK10L-1.2;LRK10L-1.2;LRK10L-1.2
Pavir.9NG545900.v	GO:000455	00500(Starc	glucan endo	4.136755	2.871948	0.578987	0.294144	8.0270922	3.0048775	0.001224734	0.052953884	up	yes	Glucan endo	At3g13560
Pavir.5NG590400.v	GO:000367	NA	an1-type zir	12.506997	29.096512	1.251312	3.934627	8.0223676	3.0040281	0.001851085	0.069135988	up	yes	Zinc finger	SAP2
Pavir.9NG745800.v	GO:000588	NA	mfs transpo	1.182683	1.640654	0.020013	0.33203	8.0198641	3.0035778	0.015131534	0.225341714	up	yes	Inorganic pl	PHT1-1;PHT1-1
Pavir.9KG368100.v	GO:005121	00270(Cyst	oxidoreduct	1.245718	2.798233	0.1518	0.352579	8.0176831	3.0031854	0.005415102	0.132981468	up	yes	S-norcoclau	NCS1
Pavir.2KG532300.v	GO:000557	NA	cobra-like p	61.959549	9.035326	0.892553	7.971529	8.0092755	3.0016717	0.008645389	0.169658116	up	yes	COBRA-lik	BC1L7
Pavir.9NG696800.v	GO:000552	03040(Splic	heat shock 7	1.024263	9.266696	0.254287	1.030647	8.0089398	3.0016113	0.00697436	0.15306521	up	yes	Heat shock	HSP70
Pavir.7NG317500.v	NA	NA	0	67.703072	510.8764	33.151943	39.125534	8.0049761	3.0008971	0.002548536	0.084070376	up	yes	Late embryo	At5g17165
Pavir.9NG408600.v	GO:000550	04626(Plan	calcium bin	5.794487	19.955214	0.069473	3.152399	7.9921552	2.9985846	0.017514247	0.242738038	up	yes	Probable ca	CML8
Pavir.9KG367200.v	GO:000016	NA	rna recognit	2.22408	5.57304	0.204185	0.771628	7.9903834	2.9982647	0.002875442	0.090734961	up	yes	Zinc finger	Os08g0126700
Pavir.2NG165600.v	GO:004316	00510(N-G	ring finger c	1.791308	2.697858	0.0001	0.562867	7.9741193	2.9953252	0.045126457	0.400541715	up	yes	RING-H2 f	ATL70
Pavir.5NG567300.v	NA	NA	0	3.07147	6.955385	0.445242	0.812438	7.972501	2.9950324	0.00035634	0.021895072	up	yes	NA	NA
Pavir.2NG087700.v	NA	NA	0	106.67156	542.00311	2.067842	79.364449	7.9658163	2.9938222	0.01990467	0.258492149	up	yes	NA	NA
Pavir.1KG009100.v	GO:000467	04626(Plan	receptor-lik	21.222197	53.599949	3.496247	5.89794	7.9647282	2.9936251	0.000644689	0.034020928	up	yes	Receptor-lil	NCRK
Pavir.9NG703600.v	GO:000368	NA	domain of u	9.146119	19.39632	1.865566	1.720862	7.9584587	2.992489	9.38892E-05	0.008261587	up	yes	AT-hook m	AHL22
Pavir.5NG584700.v	GO:000550	00053(Asco	multi-coppe	325.08292	74.184356	1.495243	48.744028	7.9473143	2.9904674	0.019960131	0.258492149	up	yes	Laccase-4 C	LAC4;LAC4
Pavir.3NG151200.v	GO:000098	04626(Plan	wrky dna -b	2.921328	20.189577	1.375264	1.533481	7.9453183	2.990105	0.001651344	0.064363024	up	yes	Probable W	WRKY46
Pavir.5KG600800.v	GO:000467	04626(Plan	protein kina	4.0902454	8.2931469	0.5074183	1.0559455	7.920992	2.9856811	0.000278042	0.018274388	up	yes	Probable LI	At1g06840;At1g06840;At1g06840;At1g06840
Pavir.1NG216300.v	NA	NA	0	1.145367	4.313653	0.084174	0.605423	7.9162467	2.9848166	0.012697943	0.204594639	up	yes	NA	NA
Pavir.6KG299800.v	GO:000578	NA	hva22-like p	2.225137	52.244522	2.511048	4.371494	7.9141775	2.9844394	0.007398996	0.15733021	up	yes	Protein HV	HVA22
Pavir.7KG270400.v	GO:000588	01200(Carb	opt oligopep	23.073645	79.167534	3.3431235	9.5964781	7.9014163	2.9821113	0.00176685	0.067192175	up	yes	Probable me	YSL16;YSL16
Pavir.5KG259800.v	GO:000557	04144(Endo	zinc finger f	15.196318	58.18895	1.121505	8.173004	7.8955508	2.9810399	0.004741226	0.123521137	up	yes	GDSL ester	At1g09390
Pavir.2NG589200.v	GO:000467	04626(Plan	leucine rich	7.4354538	3.0874016	0.6150853	0.7227215	7.8657514	2.9755846	0.000399442	0.023822612	up	yes	Protein STR	SRF6;SRF6
Pavir.2KG198900.v	GO:000573	00965(Beta	4,5-dopa di	101.65023	150.94147	2.751452	29.374069	7.862649	2.9750155	0.005279611	0.130937827	up	yes	Extradiol ri	LIGB
Pavir.2KG315800.v	GO:000467	04626(Plan	interleukin-	44.336838	62.702354	0.174124	13.504445	7.8253209	2.9681499	0.023347354	0.281524432	up	yes	Probable se	PBL28
Pavir.1KG473300.v	GO:000467	00520(Ami	non-specific	15.023974	12.064485	1.494974	1.969808	7.818229	2.9668418	0.000100417	0.008653603	up	yes	G-type lecti	B120
Pavir.9NG839700.v	GO:000579	NA	gdsl/sgnh-li	60.659721	44.429451	0.039872	13.408788	7.8140998	2.9660797	0.042338372	0.388019839	up	yes	Protein AL7	AXY4
Pavir.2NG047600.v	GO:000563	04075(Plan	carboxyleste	1.020462	16.706905	0.154935	2.114134	7.812617	2.9658059	0.022548785	0.277052154	up	yes	Probable ca	CXE15
Pavir.3KG390300.v	GO:000550	00260(Glyc	amine oxida	12.211489	37.716801	0.46883	5.922757	7.8115639	2.9656114	0.007507186	0.157709059	up	yes	Primary am	At1g62810
Pavir.9KG184600.v	GO:000546	NA	solute carric	16.744556	5.023031	0.112902	2.675011	7.807843	2.964924	0.011772596	0.196771493	up	yes	UDP-xylose	UXT1
Pavir.5NG037000.v	GO:000367	00190(Oxic	myb-like dn	1.959001	23.346592	1.1504	2.098191	7.7897134	2.9615703	0.004372438	0.118361397	up	yes	Transcriptic	MYB87;MYB87
Pavir.4NG148200.v	GO:000557	00514(Othe	glycosyltran	15.822842	20.296459	0.107352	4.533666	7.7826246	2.9602568	0.012999059	0.20735682	up	yes	Beta-1,2-xy	XAX1
Pavir.9NG610900.v	GO:000573	00052(Gala	inositol 3-al	17.674105	106.16433	3.198543	12.716159	7.7813857	2.9600271	0.004299377	0.11731336	up	yes	Galactinol s	GOLS1
Pavir.5KG381100.v	GO:000449	00905(Bras	cytochrome	14.896773	30.565422	0.179843	5.6821768	7.7553806	2.9551976	0.011295852	0.192482559	up	yes	Cytochrome	CYP72A14;CYP72A14;CYP72A14
Pavir.7NG182600.v	GO:000535	NA	predicted tra	1.5681751	1.0292353	0.067776	0.267149	7.7552002	2.955164	0.007029995	0.153926566	up	yes	Sugar transp	MST1;MST1
Pavir.9KG328200.v	GO:005121	00941(Flav	oxidoreduct	1.0350112	3.1687086	0.1062448	0.4364389	7.7461705	2.9534832	0.007814494	0.160955646	up	yes	2-oxoglutar	2ODD11;2ODD11;2ODD11;2ODD11;2ODD11
Pavir.8KG320200.v	GO:000577	00909(Sesq	lanosterol sy	0.394198	42.90008	2.8827481	2.7077652	7.7442402	2.9531237	0.024084983	0.286556389	up	yes	Achilleol B	Os11g0285000;Os11g0285000
Pavir.5NG028600.v	GO:000551	04626(Plan	non-specific	2.588429	5.495788	0.056985	0.98837	7.7334657	2.9511151	0.013215521	0.208896413	up	yes	Serine/threc	RIPK
Pavir.7KG122200.v	GO:000367	01200(Carb	ntkl-binding	267.57901	453.79642	2.990399	90.316078	7.7312471	2.9507011	0.015031129	0.224380055	up	yes	NA	NA
Pavir.9NG037000.v	GO:000551	00941(Flav	chalcone isc	39.202442	288.79025	2.151172	40.325661	7.7216843	2.9489156	0.018865985	0.251024069	up	yes	Chalcone--f	CHI
Pavir.3KG073600.v	GO:000367	NA	emb	1.899934	5.154208	0.279126	0.636225	7.7064885	2.9460736	0.003596888	0.105830759	up	yes	NA	NA
Pavir.9KG426300.v	GO:000645	04141(Prot	dnaj homolc	1.101186	10.510229	0.61541	0.893702	7.6942036	2.943772	0.004929852	0.126834076	up	yes	Chaperone p	DJA6
Pavir.5KG777700.v	NA	NA	0	17.586279	26.972586	0.514443	5.284434	7.6840507	2.941867	0.003660383	0.106779169	up	yes	NA	NA
Pavir.3KG103800.v	NA	NA	0	4.569612	14.095197	0.487079	1.94329	7.6798252	2.9410735	0.006397102	0.145417262	up	yes	Zinc finger	ZFP7
Pavir.9NG562600.v	GO:000367	NA	alcohol acet	13.026041	19.874367	4.28721	0.0001	7.6739046	2.9399608	0.046486533	0.40582121	up	yes	NA	NA
Pavir.1NG007800.v	GO:000467	04626(Plan	receptor-lik	20.410929	52.646641	2.157771	7.387399	7.6538783	2.936191	0.001819865	0.068377798	up	yes	Receptor-lil	NCRK
Pavir.7KG265000.v	GO:000666	00514(Othe	kdel lys-asp	32.414494	29.824125	0.470361	7.67297	7.6428944	2.9341191	0.007561076	0.157976512	up	yes	NA	NA
Pavir.9NG606500.v	GO:000645	04141(Prot	dnaj homolc	2.214515	10.256608	0.326418	1.306087	7.6392556	2.9334321	0.003797799	0.109093798	up	yes	Chaperone p	ATJ2
Pavir.5KG608600.v	NA	NA	0	1.277677	2.351096	0.23605	0.240627	7.6126455	2.9283979	0.00176566	0.067192175	up	yes	NA	NA
Pavir.1KG122100.v	NA	NA	cysteine-ricl	10.133225	211.49884	1.008182	28.180012	7.5932093	2.9247098	0.035898571	0.356279575	up	yes	NA	NA
Pavir.1NG335500.v	NA	NA	protein of u	2.647399	16.881708	0.713497	1.858614	7.5926377	2.9246012	0.004876545	0.126102155	up	yes	NA	NA
Pavir.9KG330500.v	GO:000563	NA	plant plecks	12.658989	35.460278	1.855296	4.487651	7.5862635	2.9233895	0.000539366	0.029758575	up	yes	VAN3-bind	VAB
Pavir.1KG408700.v	GO:000484	04141(Prot	rnf5//subfar	10.118068	32.639538	0.903322	4.738186	7.5791093	2.9220283	0.002550907	0.084074773	up	yes	E3 ubiquiti	RMA1H1
Pavir.5NG509500.v	GO:000487	NA	rapid alkali	41.202042	98.318359	1.922215	16.503052	7.5722323	2.9207187	0.003844347	0.109926675	up	yes	Rapid alkal	RALF
Pavir.3NG295000.v	GO:000367	04075(Plan	camp-respo	5.06111	73.993011	3.046561	7.401421	7.5664488	2.9196164	0.006422891	0.145641094	up	yes	Light-induc	CPRF2
Pavir.1NG368100.v	GO:000484	04141(Prot	rnf5//subfar	1.357523	16.925396	0.769594	1.647404	7.5643087	2.9192082	0.006270354	0.144060155	up	yes	E3 ubiquiti	RMA1H1
Pavir.2KG536300.v	GO:000449	00905(Bras	cytochrome	21.351034	145.61853	5.202734	16.920713	7.5471767	2.9159371	0.005166826	0.130028897	up	yes	Cytochrome	CYP709B2
Pavir.6NG060500.v	GO:000582	00940(Phen	flavone 3'-o	1043.3783	1977.7953	57.637424	342.98297	7.5412376	2.9148013	0.003902025	0.110649653	up	yes	Caffeic acid	COMT
Pavir.1NG217500.v	NA	NA	0	2.149535	11.787933	1.301521	0.552778	7.5163002	2.9100227	0.0057618	0.136614798	up	yes	NA	NA
Pavir.9NG259300.v	GO:000467	04626(Plan	non-specific	12.625831	21.599613	0.081268	4.480046	7.5034177	2.9075479	0.017637996	0.243207467	up	yes	Leucine-ricl	PXC1
Pavir.2NG623300.v	NA	NA	0	4.69237	29.061943	0.67511	3.824069	7.5023272	2.9073382	0.007454397	0.157697172	up	yes	NA	NA
Pavir.6NG212600.v	NA	NA	0	2.035864	8.768811	0.788889	0.656185	7.4769008	2.9024404	0.004941121	0.126961436	up	yes	NA	NA
Pavir.4KG339000.v	GO:000370	NA	myosin heav	5.9994465	70.63343	2.5596857	7.7057651	7.4651253	2.9001665	0.007434481	0.157460964	up	yes	Protein POI	POLAR;POLAR;POLAR
Pavir.1KG072500.v	NA	NA	protein of u	1.3771733	2.4726811	0.0488491	0.4678451	7.4509334	2.8974212	0.011868573	0.197303733	up	yes	NA	NA
Pavir.2NG197100.v	GO:001602	02010(ABC	peptide expe	5.9183803	12.76118	0.3986072	2.110574	7.4444844	2.8961719	0.001603317	0.063092092	up	yes	Putative mu	Os02g0190300;Os02g0190300
Pavir.2KG266800.v	GO:000367	04144(Endo	c2 domain (	3.407327	3.667701	0.232107	0.719074	7.4381511	2.8949441	0.006904083	0.151966683	up	yes	NA	NA
Pavir.9NG071300.v	GO:000563	NA	duf761-assc	18.984764	31.221004	0.291837	6.461866	7.4338134	2.8941025	0.012183953	0.20016951	up	yes	NA	NA
Pavir.2KG537800.v	NA	NA	0	6.8875531	21.632808	1.8599991	1.9845611	7.4183677	2.8911018	0.00034118	0.021329717	up	yes	NA	NA
Pavir.2KG306900.v	GO:000372	NA	60s ribosom	16.256128	62.303915	0.407514	10.197612	7.4077425	2.889034	0.018531015	0.248488881	up	yes	NA	NA
Pavir.2KG569900.v	GO:000950	04070(Phos	dihydrofolat	21.060234	51.095901	1.128776	8.632636	7.3919772	2.8859603	0.003745618	0.10823485	up	yes	Rhodanese-	STR6
Pavir.5NG354600.v	GO:000016	03040(Splic	wd40 repeat	3.08952	6.554715	0.419184	0.888843	7.3731162	2.8822745	0.001931426	0.071216368	up	yes	LEC14B ho	-;-
Pavir.1KG483200.v	NA	NA	0	137.56378	159.92383	1.816964	38.564137	7.367001	2.8810774	0.010959473	0.189237262	up	yes	NA	NA
Pavir.5NG446800.v	NA	NA	0	3.229507	53.663982	2.059613	5.665981	7.3642867	2.8805458	0.009034844	0.173640002	up	yes	NA	NA
Pavir.1NG118000.v	GO:000472	NA	protein phos	16.230989	45.081211	1.008066	7.317922	7.3639549	2.8804808	0.003552811	0.105270166	up	yes	Probable pr	Os02g0224100
Pavir.4KG192300.v	GO:000473	01200(Carb	pyruvate del	1.859311	9.81768	0.838237	0.747758	7.3625648	2.8802084	0.002595572	0.084995151	up	yes	Pyruvate de	Os06g0246500
Pavir.5KG303200.v	NA	NA	0	1.414021	1.2014	0.138751	0.218577	7.3193844	2.8717223	0.007959665	0.162325709	up	yes	NA	NA
Pavir.9KG434000.v	GO:000573	00250(Alar	aminotransf	98.640518	130.13239	1.808491	29.459948	7.3164159	2.8711371	0.010053525	0.181875162	up	yes	Alanine--gl	At3g08860
Pavir.5KG203600.v	NA	NA	0	122.57155	189.9117	2.745571	39.982185	7.3133549	2.8705334	0.008239552	0.164760344	up	yes	GDSL ester	At1g28610
Pavir.9NG228300.v	GO:000685	NA	mate efflux	2.232972	7.661007	0.143573	1.210346	7.3076595	2.8694094	0.008184291	0.164298747	up	yes	Protein DE	DTX40
Pavir.5NG344300.v	GO:000467	04626(Plan	pto-interacti	7.0107472	49.91134	1.7282728	6.0812959	7.288762	2.8656738	0.003968678	0.111780454	up	yes	Pto-interact	PTI1;PTI1
Pavir.4NG103300.v	GO:000588	NA	sodium/solu	1.077839	1.928669	0.101347	0.312496	7.2648516	2.8609333	0.007845432	0.161235497	up	yes	Urea-protor	DUR3

Pavir.5KG263300.v	GO:000447	01230(Bios	s-adenosylm	191.60834	1977.3887	95.362915	203.28511	7.2627201	2.86051	0.007548575	0.157919056	up	yes	S-adenosylm	SAM2
Pavir.5KG264200.v	GO:000447	01230(Bios	s-adenosylm	170.07364	1696.6067	83.922035	173.21011	7.2596147	2.859893	0.007136852	0.155046644	up	yes	S-adenosylm	SAMS1
Pavir.7KG408800.v	GO:000028	01230(Bios	shikimate ki	30.483362	242.27581	1.1165434	36.490233	7.252926	2.8585631	0.030572182	0.326982236	up	yes	Shikimate k	SK3;SK3
Pavir.1NG465200.v	GO:000419	NA	aspartyl pro	11.742268	9.593019	0.489337	2.453101	7.2508875	2.8581576	0.001470385	0.059726224	up	yes	Aspartyl pro	At5g10770
Pavir.5NG355700.v	GO:000016	NA	wd40 repeat	2.477632	7.918097	0.486853	0.948432	7.242972	2.8565818	0.005080398	0.128459391	up	yes	LEC14B pr	-
Pavir.8KG180100.v	GO:000166	NA	saposin (psa	4.134439	3.524379	1.059358	0.0001	7.2289963	2.8537954	0.047223415	0.40946722	up	yes	NA	NA
Pavir.6KG342500.v	GO:000551	04141(Prot	molecular ch	81.452523	51.10396	11.092294	7.2989082	7.2076029	2.8495195	0.000575659	0.031346695	up	yes	Heat shock	HSP81-1;HSP81-1
Pavir.3NG200700.v	GO:000367	NA	ring finger p	3.3929308	5.1187626	0.0476787	1.1344375	7.2003863	2.8480743	0.016725955	0.237661086	up	yes	Protein LAX	LAX2;NA;LAX2;LAX2;LAX2
Pavir.5KG608300.v	NA	NA	0	30.749887	49.348969	0.105813	11.025165	7.1960304	2.8472013	0.027907732	0.310335091	up	yes	NA	NA
Pavir.5KG253600.v	GO:000557	04144(Endo	zinc finger f	15.351768	59.371799	1.467111	8.949585	7.1734422	2.8426656	0.005716065	0.136102468	up	yes	GDSL ester	At1g09390
Pavir.2KG455000.v	NA	NA	0	23.181807	47.011658	0.272003	9.514261	7.1726519	2.8425066	0.015585043	0.229009317	up	yes	NA	NA
Pavir.5KG388400.v	GO:000550	04626(Plan	ef-hand calc	2.899424	6.96103	0.652189	0.724759	7.161094	2.84018	0.002258973	0.077441975	up	yes	Probable ca	CML14
Pavir.1KG001500.v	GO:000573	04141(Prot	membrane a	13.374326	26.957548	1.5326939	4.1017452	7.1580992	2.8395765	0.000556988	0.03059608	up	yes	Protein disu	PDIL1-4;PDIL1-4
Pavir.5NG243400.v	NA	NA	0	3.723768	3.035836	0.537723	0.407367	7.1523389	2.8384151	0.000854702	0.041383669	up	yes	NA	NA
Pavir.9NG638300.v	GO:000467	00260(Glyc	protein kina	1.656915	1.753007	0.009636	0.467127	7.1522371	2.8383946	0.030960051	0.329256158	up	yes	G-type lecti	At2g19130
Pavir.9KG527500.v	GO:000446	NA	lactoylgluta	1.713666	13.702889	1.118648	1.039305	7.1440643	2.8367451	0.009148803	0.174757488	up	yes	NA	NA
Pavir.8NG346000.v	GO:000573	00130(Ubic	mpbq/msbq	2.415972	4.517557	0.282453	0.688161	7.1434463	2.8366203	0.00524878	0.1307627	up	yes	2-methyl-6-	Os12g0615400
Pavir.5KG765800.v	NA	NA	0	6.643737	9.48775	0.459889	1.801512	7.133404	2.8345907	0.00166711	0.064442719	up	yes	NA	NA
Pavir.7KG410700.v	GO:000028	01230(Bios	shikimate ki	17.03838	135.59096	0.824618	20.679779	7.0975874	2.8273287	0.028269824	0.312498409	up	yes	Shikimate k	SK3
Pavir.5KG536500.v	GO:000983	00500(Starc	cellulose sy	244.64005	581.02777	1.006058	115.57499	7.0823503	2.8242282	0.041904178	0.385680547	up	yes	Cellulose sy	CESA4
Pavir.1KG495000.v	GO:000402	00010(Glyc	aldehyde de	8.0344643	12.729651	1.4865935	1.4463486	7.07962	2.8236719	0.000208955	0.014647483	up	yes	Aldehyde de	ALDH2B4;ALDH2B4
Pavir.9KG372400.v	GO:000367	NA	gb	7.121625	22.179407	1.230552	2.90913	7.0780876	2.8233596	0.001558942	0.061917697	up	yes	Protein CO	CGLD27
Pavir.2KG313600.v	NA	NA	0	14.061126	0.7257209	0.2812581	1.8136239	7.0585582	2.8193735	0.029530941	0.320962114	up	yes	NA	NA
Pavir.2NG638700.v	GO:000460	00940(Phen	peroxidase /	31.424278	3.44143	0.287381	4.655847	7.0532268	2.8182834	0.032024006	0.335163785	up	yes	Peroxidase	PRX112
Pavir.1NG197000.v	NA	NA	protein iq-d	11.399066	34.785942	1.118457	5.431057	7.0516695	2.8179649	0.002444795	0.081870125	up	yes	NA	NA
Pavir.2KG065600.v	GO:000369	NA	aaa domain	10.80366	9.187178	1.057085	1.779582	7.0472981	2.8170702	0.000516951	0.029076929	up	yes	NA	NA
Pavir.8NG053700.v	NA	NA	0	1.369257	1.770155	0.024295	0.421493	7.0423879	2.8160647	0.023957265	0.285730068	up	yes	NA	NA
Pavir.5KG540600.v	GO:000983	00500(Starc	cellulose sy	168.72714	405.24029	0.7891141	80.795283	7.0352599	2.8146037	0.040476998	0.379167847	up	yes	Cellulose sy	CESA4;CESA4
Pavir.2NG283900.v	GO:000367	NA	nucleotide-d	3.060299	13.597959	0.996203	1.375989	7.022306	2.8119449	0.001893319	0.070349639	up	yes	Uncharacter	At4g15970
Pavir.2KG274200.v	GO:000573	NA	copper trans	7.875837	25.774086	0.758419	4.040175	7.0124547	2.8099195	0.003894697	0.110525262	up	yes	NA	NA
Pavir.9KG402500.v	GO:000588	01200(Carb	eama-like tr	1.077617	2.543406	0.387167	0.130444	6.9956454	2.8064572	0.0090284	0.173633972	up	yes	WAT1-rela	At5g45370
Pavir.9NG190600.v	GO:000557	03010(Ribo	leucine rich	9.588388	44.182026	0.100411	7.585886	6.9956201	2.806452	0.032924018	0.340006925	up	yes	Leucine-ricl	LRX6
Pavir.7KG414200.v	GO:000950	00941(Flav	anthocyanid	6.870604	31.922239	1.672435	3.880726	6.9857227	2.8044094	0.00181101	0.068177911	up	yes	Anthocyani	ANR
Pavir.6KG296700.v	GO:000551	04626(Plan	non-specific	7.980733	10.931838	0.108172	2.601182	6.9804725	2.8033247	0.013267825	0.209130801	up	yes	Serine/threo	RIPK
Pavir.5KG745600.v	GO:000013	00790(Fola	exostosin h	245.92426	259.31561	5.608275	66.991768	6.9592227	2.7989262	0.009172407	0.1749902	up	yes	Probable gl	Os01g0926700
Pavir.2KG352700.v	NA	NA	0	23.876642	12.443407	0.850863	4.371222	6.9550858	2.7980683	0.001916392	0.070940503	up	yes	NA	NA
Pavir.1KG071400.v	GO:000375	NA	peptidyl-pro	1.5926451	4.178945	0.181837	0.648028	6.9548543	2.7980203	0.009575571	0.178946539	up	yes	Peptidyl-pro	FKBP17-1;FKBP17-1;FKBP17-1;FKBP17-1
Pavir.5KG673000.v	GO:000573	00965(Beta	4,5-dopa di	42.258179	58.420017	3.019344	11.467484	6.949637	2.7969376	0.001491461	0.060200724	up	yes	Extradiol ri	LIGB
Pavir.9KG538600.v	GO:000467	04626(Plan	non-specific	15.647412	39.7901	1.348829	6.62869	6.9492172	2.7968505	0.002176906	0.07587466	up	yes	Probable se	PBL16
Pavir.5NG572200.v	GO:000579	NA	gdsl/sgnh-li	41.705971	10.183207	1.858513	5.609869	6.9478473	2.7965661	0.002532464	0.083760806	up	yes	Protein ESK	ESK1
Pavir.7NG387800.v	GO:000367	NA	wound-indu	167.30762	541.45111	63.269497	38.784828	6.9449161	2.7959573	0.001493264	0.060200724	up	yes	NA	NA
Pavir.6KG104900.v	GO:000538	NA	zinc/iron tra	5.634762	7.53397	0.232558	1.664497	6.9416712	2.795283	0.005367207	0.132235113	up	yes	Zinc transpo	ZIP4
Pavir.4NG034100.v	NA	NA	0	1.254957	5.003272	0.462672	0.440886	6.9262062	2.7920653	0.010318905	0.184656922	up	yes	NA	NA
Pavir.9KG089500.v	GO:000645	04141(Prot	dnaj homolo	1.666509	12.69427	0.814476	1.268548	6.8941976	2.7853826	0.005980313	0.139941557	up	yes	Chaperone	DJA6
Pavir.9KG573300.v	GO:000950	NA	expansin-lik	10.300973	36.162804	1.943157	4.803413	6.8870221	2.7838803	0.001241534	0.053533583	up	yes	Expansin-li	EXLA1
Pavir.7NG440700.v	GO:000367	NA	protein of u	42.19931	66.383972	0.703843	15.087636	6.8760679	2.7815838	0.014161446	0.21647193	up	yes	NA	NA
Pavir.1KG075700.v	GO:000367	NA	genomic dna	3.194709	12.808742	0.734989	1.595318	6.867529	2.7797911	0.003625134	0.106338496	up	yes	VQ motif-c	VQ22
Pavir.1NG458500.v	GO:000419	04145(Phag	cysteine pro	15.960025	1.59462	0.225468	2.331018	6.8667088	2.7796188	0.01933091	0.254759839	up	yes	Cysteine pro	XCP1
Pavir.1KG484200.v	GO:000419	NA	aspartyl pro	13.474242	17.00807	0.680825	3.774393	6.841935	2.7744044	0.001965224	0.071758382	up	yes	Aspartyl pro	At5g10770
Pavir.3NG027600.v	NA	NA	aba/wds ind	8.560344	61.399464	1.89247	8.366646	6.8192823	2.7696199	0.009274048	0.175807947	up	yes	Abscisic str	ASR1
Pavir.7KG238100.v	NA	NA	0	413.45734	1618.0316	54.964912	243.59891	6.8042034	2.7664263	0.006696011	0.148753231	up	yes	Phenylalani	PAL
Pavir.3KG041700.v	GO:000950	NA	chalcone--fl	46.17379	110.31887	2.188187	20.824707	6.8002165	2.7655807	0.008258168	0.165044759	up	yes	Probable ch	CHI3
Pavir.2KG538200.v	GO:000455	00500(Starc	glucan endo	53.148888	81.80072	0.610382	19.304001	6.7764895	2.7605381	0.019940269	0.258492149	up	yes	Glucan endo	At3g13560
Pavir.9KG564000.v	GO:005121	00941(Flav	oxidoreduct	3.175046	16.549644	1.565849	1.345925	6.7741143	2.7600323	0.002737746	0.087741633	up	yes	Flavanone 3	F3H-2
Pavir.4NG001400.v	GO:000419	NA	aspartyl pro	12.527898	8.95575	0.154607	3.021723	6.76367	2.7578063	0.010813114	0.188267443	up	yes	Aspartyl pro	APCB1
Pavir.1KG345600.v	NA	NA	0	8.737066	6.964334	0.63667	1.692517	6.7411505	2.7529948	0.000687018	0.035710334	up	yes	NA	NA
Pavir.2NG241700.v	GO:000367	04144(Endo	c2 domain (	4.812463	13.602539	0.893958	1.840999	6.7331962	2.7512915	0.001626168	0.063579631	up	yes	NA	NA
Pavir.4KG260100.v	GO:000563	04075(Plan	member of '	6.352292	7.739094	0.031507	2.063782	6.7252708	2.7495924	0.030923151	0.329049999	up	yes	Probable ca	CXE15
Pavir.5NG269000.v	GO:000382	NA	nad depende	18.434855	52.336193	3.906809	6.621181	6.7221804	2.7489293	0.001428728	0.0584767	up	yes	Cinnamoyl-	SNL6
Pavir.1NG419300.v	GO:001584	NA	aquaporin tr	1.800441	9.251705	0.489688	1.154959	6.7200718	2.7484767	0.012409348	0.202368249	up	yes	Aquaporin	NIP2-1
Pavir.5KG468900.v	GO:000941	00196(Phot	light-harves	7.974859	48.059017	1.366453	6.972575	6.7194733	2.7483482	0.006593366	0.147204491	up	yes	Chlorophyll	-
Pavir.5NG606200.v	GO:000097	NA	gata transcr	3.310937	15.193472	0.356201	2.399903	6.7139734	2.7471668	0.011733634	0.196465165	up	yes	GATA trans	GATA23;NA
Pavir.7NG358700.v	GO:000577	00531(Glyc	aquaporin tr	23.872864	40.060204	0.926592	8.597362	6.7128703	2.7469298	0.004840487	0.125342258	up	yes	Aquaporin	PIP2-5
Pavir.1NG127900.v	GO:000573	NA	tetrahydrob	14.004189	26.172327	1.242979	4.744852	6.7096944	2.7462471	0.001325287	0.055795265	up	yes	Berberine b	-
Pavir.8NG345300.v	GO:004316	00563(Glyc	ring finger d	3.077553	13.762088	0.410348	2.099937	6.7082586	2.7459383	0.011832909	0.197160934	up	yes	E3 ubiquitin	ATL55
Pavir.3NG006000.v	GO:000441	00650(Buta	hydroxymet	32.576757	19.964101	1.8644309	5.9730865	6.7037628	2.7449711	0.001376785	0.057240815	up	yes	Hydroxyme	HMGCL;HMGCL
Pavir.5NG640100.v	GO:000550	00860(Porp	adenylate cy	1.121817	8.695575	0.570492	0.895924	6.6948206	2.7430454	0.006066744	0.141348194	up	yes	Chlorophyll	CAO
Pavir.4NG061300.v	GO:000367	03013(RNA	cold shock c	5.3135309	26.469237	4.1887955	0.5629127	6.6887037	2.7417266	0.00607672	0.141348194	up	yes	Glycine-ricl	GRP-2;GRP-2
Pavir.5NG631200.v	GO:000449	00903(Limo	cytochrome	1.457417	7.194613	0.204434	1.0905	6.6814448	2.7401601	0.013941654	0.214437719	up	yes	Cytochrome	CYP94A1
Pavir.4KG158500.v	GO:000588	NA	mlo-like pro	5.847332	31.534082	2.035356	3.56202	6.6783818	2.7394986	0.001943369	0.07143226	up	yes	MLO-like p	MLO1
Pavir.4KG313700.v	GO:000573	00073(Cuti	transferase f	14.728666	52.668407	1.608415	8.502453	6.6658049	2.7367791	0.005867479	0.137872911	up	yes	Acyl transfe	AT7
Pavir.5KG693800.v	GO:000371	00903(Limo	btb and mat	1.755151	9.16373	0.284999	1.354161	6.6612661	2.7357964	0.008968942	0.173170633	up	yes	BTB/POZ a	BT3
Pavir.3KG213300.v	GO:000367	NA	zinc finger c	18.166466	1.511926	0.277816	2.678388	6.6566421	2.7347946	0.023131227	0.280992432	up	yes	Zinc finger	Os05g0576300;Os05g0576300
Pavir.2KG400200.v	NA	NA	camp-respo	4.636529	7.239314	0.561966	1.222429	6.6553891	2.734523	0.005708672	0.136102468	up	yes	NA	NA
Pavir.5NG553400.v	GO:000588	01200(Carb	eama-like tr	60.071613	24.077295	0.816201	11.846642	6.6453409	2.7323432	0.012963095	0.207263269	up	yes	WAT1-rela	At5g07050
Pavir.9KG651800.v	GO:000370	04075(Plan	ap2 domain	23.33773	80.371635	5.153859	10.45724	6.6433097	2.7319022	0.002296286	0.078221538	up	yes	Ethylene-res	RAP2-2
Pavir.5NG559500.v	GO:000467	04626(Plan	protein kina	2.843332	8.250054	0.51359	1.156835	6.641056	2.7314127	0.001377226	0.057240815	up	yes	Probable LI	At1g06840
Pavir.7KG097700.v	GO:000573	NA	copper trans	2.512824	6.585428	0.500142	0.870687	6.6370437	2.7305408	0.007780164	0.160731145	up	yes	NA	NA
Pavir.9KG288300.v	GO:000588	NA	sodium/solu	2.552001	3.447107	0.30278	0.601155	6.6366586	2.7304571	0.00731308	0.156441785	up	yes	Urea-protor	DUR3
Pavir.9KG263500.v	GO:000550	04626(Plan	calcium bin	6.595851	17.981789	0.301424	3.403256	6.634214	2.7299256	0.012271708	0.200996031	up	yes	Probable ca	CML8
Pavir.7KG307100.v	GO:000497	NA	ionotropic g	0.7960421	9.1605933	0.2615221	1.2426771	6.6192266	2.7266627	0.013444246	0.210475267	up	yes	Glutamate r	GLR3.1;GLR3.1;GLR3.1
Pavir.8NG271600.v	GO:000367	NA	targeting pr	107.14092	310.84042	2.54983	60.625729	6.6161874	2.7260001	0.022576517	0.277141434	up	yes	Protein WV	WDL3

Pavir.3KG542500.v	GO:000367	NA	auxin respo	9.713877	8.64695	0.037357	2.743289	6.6030796	2.723139	0.032747414	0.339131364	up	yes	Auxin-respo	SAUR76
Pavir.3KG333100.v	GO:000367	NA	zinc finger c	3.205868	8.013521	0.488056	1.212417	6.5978048	2.7219861	0.005256863	0.1307627	up	yes	Zinc finger	Os05g0525900
Pavir.1KG424000.v	NA	NA	myb-like dn	2.024852	6.859259	0.494681	0.854944	6.5826515	2.7186688	0.006312289	0.144254761	up	yes	Myb-relatec	-
Pavir.9KG559600.v	GO:000367	NA	stress respo	6.768132	27.14142	0.297655	4.857602	6.5776647	2.7175755	0.016197938	0.23345038	up	yes	NA	NA
Pavir.7NG214800.v	GO:000466	01230(Bios	arogenate/p	7.881959	20.281576	0.805234	3.479294	6.573311	2.7166202	0.002572583	0.084566619	up	yes	Arogenate c	ADT6
Pavir.9NG154100.v	GO:000082	04070(Phos	inositol-hex	25.651455	7.7470803	3.0410067	2.0483009	6.562491	2.7142435	0.00070849	0.036465876	up	yes	Inositol hex	VIP2;VIP2
Pavir.9KG214700.v	GO:000563	00500(Star	beta-amylas	8.360798	30.286503	0.780418	5.109066	6.5620861	2.7141545	0.005679805	0.13593549	up	yes	Beta-amylas	BAMY1
Pavir.3KG482600.v	GO:001602	00903(Lim	isoflavone 2	8.408923	5.1784	0.097469	1.973481	6.5609131	2.7138966	0.027231356	0.307256375	up	yes	Cytochrom	CYP81Q32
Pavir.8KG307800.v	NA	NA	0	1.536632	2.506291	0.244905	0.373099	6.5419043	2.7097106	0.004278864	0.117179133	up	yes	NA	NA
Pavir.3NG265300.v	NA	NA	0	1.277596	3.872721	0.114714	0.674326	6.5273205	2.7064909	0.013885947	0.214303781	up	yes	Elongation	HOS3
Pavir.3KG342500.v	GO:000471	04075(Plan	serine-threo	14.98981	16.700478	0.4634677	4.4236171	6.4844974	2.6969948	0.005189769	0.130321762	up	yes	Serine/thre	HT1;HT1;HT1;HT1
Pavir.1KG558400.v	NA	NA	camp-respo	5.637122	38.762127	2.390135	4.461146	6.4804303	2.6960896	0.004068055	0.113642408	up	yes	NA	NA
Pavir.7KG020900.v	GO:000367	NA	thioredoxin	4.303606	12.261446	0.339279	2.218157	6.4772108	2.6953727	0.006485919	0.146275777	up	yes	NA	NA
Pavir.3NG273000.v	GO:000090	00430(Tau	cysteamine	10.946371	31.527191	3.429785	3.141591	6.4634198	2.6922977	0.000592712	0.031905404	up	yes	Plant cystei	PCO1
Pavir.1NG105200.v	NA	NA	0	4.21662	6.482086	0.803284	0.856867	6.4444174	2.6880499	0.00321547	0.097984464	up	yes	Caffeoylshil	CSE
Pavir.1KG131500.v	GO:001602	00380(Tryp	flavonoid 3'	2.007343	19.699234	1.227855	2.149119	6.4278188	2.6843293	0.006377858	0.145321553	up	yes	Trimethyltri	CYP92C6
Pavir.2KG350700.v	GO:000563	NA	heat stress t	11.148069	12.049347	0.389587	3.222112	6.4228542	2.6832145	0.004970209	0.127202873	up	yes	Heat stress	HSFB1
Pavir.1KG234400.v	NA	NA	0	18.328182	5.955827	2.567086	1.215012	6.4207773	2.682748	0.00628799	0.144060155	up	yes	NA	NA
Pavir.9KG136300.v	NA	NA	0	7.89968	73.504089	6.7474	5.936132	6.4180679	2.682139	0.006174197	0.142758828	up	yes	Protein NE	NDX1
Pavir.1NG548300.v	NA	NA	0	6.914987	42.177414	4.312544	3.336893	6.4177796	2.6820743	0.002132514	0.074883129	up	yes	Protein SC	SCAR2
Pavir.3KG035800.v	GO:000014	NA	exocyst com	1.149208	9.01279	0.449377	1.134201	6.4171124	2.6819243	0.010415149	0.185224526	up	yes	Exocyst cor	EXO70A1
Pavir.3KG104300.v	GO:000563	NA	act domain-	4.329614	41.425594	2.822478	4.311093	6.4140678	2.6812396	0.005529482	0.134588386	up	yes	ACT domai	ACR9
Pavir.8NG190700.v	GO:000563	04075(Plan	member of '	4.047782	1.680445	0.017142	0.876323	6.4112495	2.6806055	0.046796457	0.407109922	up	yes	Probable ca	CXE18
Pavir.3KG208200.v	GO:000367	NA	peptidase of	46.211269	54.587509	2.182876	13.546637	6.408258	2.6799322	0.005174112	0.130038071	up	yes	NA	NA
Pavir.6KG052300.v	GO:000367	00190(Oxi	myb transcr	18.889009	17.762455	0.253992	5.466941	6.4065536	2.6795485	0.013347521	0.209368875	up	yes	Transcriptic	MYB86
Pavir.9NG544300.v	GO:000384	01230(Bios	3-deoxy-7-p	170.95711	519.19617	29.114214	78.636322	6.4051029	2.6792218	0.004246708	0.116643333	up	yes	Phospho-2-	DAHPS2
Pavir.7NG401500.v	GO:000573	NA	copper trans	9.15106	6.706323	0.2949	2.18697	6.3892883	2.6756552	0.008651557	0.169658116	up	yes	Heavy meta	HIPP32
Pavir.5KG627400.v	GO:000367	NA	homeobox p	19.504913	94.565834	7.543567	10.341244	6.3780795	2.6731221	0.00385819	0.110003727	up	yes	BEL1-like	BLH9
Pavir.3NG316000.v	GO:000367	NA	dvl family (	15.956783	1.818805	0.130003	2.659621	6.3720372	2.6717547	0.045285044	0.401106775	up	yes	NA	NA
Pavir.6NG264600.v	GO:000563	00360(Phe	caffeoyl-coa	1624.1738	1203.6822	28.264119	415.98182	6.3655191	2.6702782	0.015534692	0.228528838	up	yes	Tricin synth	ROMT-15;ROMT-15;ROMT-15
Pavir.9NG171700.v	GO:000647	NA	n-acetyltran	1.163217	4.895538	0.547489	0.404988	6.3610512	2.6692652	0.0113761	0.193234276	up	yes	NA	NA
Pavir.7NG265900.v	GO:000367	NA	dna topoiso	12.502769	68.771019	4.423934	8.362317	6.3563423	2.6681968	0.002721463	0.087741633	up	yes	NA	NA
Pavir.7KG405200.v	GO:000367	NA	wound-indu	8.335145	29.658813	4.851972	1.128556	6.3529438	2.6674252	0.004116646	0.114404397	up	yes	NA	NA
Pavir.6KG123400.v	GO:000467	NA	msp (major	1.1778052	2.21323	0.3466077	0.1873419	6.3508525	2.6669503	0.009980388	0.181831401	up	yes	Vesicle-ass	PVA13;PVA13
Pavir.1KG304600.v	NA	NA	0	2.057042	7.192677	0.35649	1.100682	6.3477194	2.6662384	0.016468302	0.235719432	up	yes	NA	NA
Pavir.2NG638800.v	GO:000466	00940(Phe	peroxidase	48.972618	7.517066	0.906279	7.992932	6.3477182	2.6662381	0.015109578	0.225193244	up	yes	Peroxidase	PRX112
Pavir.4KG160700.v	GO:000367	00040(Pent	putative tra	1.079252	6.240777	0.313838	0.83937	6.3475357	2.6661966	0.015805881	0.230800811	up	yes	NA	NA
Pavir.9NG196100.v	NA	NA	0	0.5363	22.637924	0.504735	3.156229	6.3300879	2.6622255	0.040451849	0.37902676	up	yes	NA	NA
Pavir.7KG330600.v	NA	NA	0	12.304577	17.408751	1.838552	2.857526	6.3272646	2.6615819	0.0004741	0.027321085	up	yes	NA	NA
Pavir.8KG154800.v	GO:000467	03018(RN	non-specific	6.360004	39.891308	0.149221	7.165743	6.3228352	2.6605716	0.03835667	0.369153475	up	yes	G-type lecti	At4g11900
Pavir.9NG364700.v	GO:000965	NA	putative ser	4.603666	25.759836	0.144224	4.66685	6.3111692	2.6579073	0.033666574	0.343927244	up	yes	Plastidal gl	PLGG1
Pavir.1NG358100.v	GO:000165	04075(Plan	phytosulfok	2.106635	6.51454	0.291519	1.074579	6.3108027	2.6578235	0.012371412	0.202064075	up	yes	Phytosulfok	PSKR
Pavir.2NG329100.v	NA	NA	0	0.51257	8.803717	0.205439	1.271562	6.3075699	2.6570843	0.036195379	0.357604251	up	yes	NA	NA
Pavir.5NG348800.v	GO:000097	NA	transcriptio	18.124252	74.977493	3.108138	11.667359	6.3010906	2.6556015	0.004021858	0.112540498	up	yes	GATA trans	GATA23
Pavir.2KG000500.v	NA	NA	0	16.558655	3.481379	1.531741	1.648767	6.3008909	2.6555558	0.005696104	0.136102468	up	yes	NA	NA
Pavir.6KG401200.v	GO:000367	00190(Oxi	myb-like dn	8.013633	4.378151	0.108099	1.863937	6.2837514	2.6516261	0.026478111	0.303219161	up	yes	Myb-relatec	MYB1
Pavir.5NG551900.v	GO:000455	00500(Star	glucan endo	7.178233	0.336343	0.136422	1.059568	6.2831428	2.6514864	0.047499044	0.410838303	up	yes	Putative glu	-
Pavir.2NG319200.v	GO:000372	NA	60s ribosom	20.481968	46.410458	0.762117	9.884881	6.2827499	2.6513962	0.01471048	0.220835453	up	yes	NA	NA
Pavir.6KG319100.v	NA	NA	0	4.63106	12.264417	1.448759	1.241834	6.2794622	2.650641	0.002797605	0.089227237	up	yes	NA	NA
Pavir.2KG338500.v	GO:000551	00940(Phe	shikimate o	2.040935	1.572756	0.139767	0.436421	6.2717221	2.6488616	0.013806735	0.213594684	up	yes	Hydroxycin	HCT4
Pavir.4KG335900.v	GO:000467	04075(Plan	receptor like	13.153487	22.30471	0.22492	5.429802	6.2705465	2.6485912	0.01734602	0.24174101	up	yes	Receptor lik	LECRKS2
Pavir.2NG491100.v	GO:000455	00230(Puri	nucleoside c	60.596985	50.530422	6.665899	11.085618	6.260164	2.6462004	0.000380349	0.023012673	up	yes	Nucleoside	NDKR
Pavir.6KG190400.v	GO:000551	NA	speckle-typ	18.715725	27.072653	0.703699	6.620941	6.2512803	2.6441517	0.007937832	0.162179542	up	yes	BTB/POZ a	BPM1
Pavir.9KG487100.v	GO:000579	NA	protein eski	93.641289	53.64262	0.830862	22.745672	6.2470552	2.6431763	0.02481728	0.291758346	up	yes	Protein ESI	ESK1
Pavir.2NG256500.v	GO:001526	NA	mitochondri	1.0290511	4.8360277	0.5088921	0.4318218	6.2347109	2.6403227	0.013696072	0.212470074	up	yes	NA	NA
Pavir.4KG373200.v	NA	NA	0	3.776228	34.578144	2.573997	3.579479	6.2329604	2.6399175	0.007976365	0.162515989	up	yes	NA	NA
Pavir.2KG293800.v	NA	NA	f-box-like (	1.590217	10.997089	1.097113	0.924875	6.225213	2.6381232	0.011706881	0.196235312	up	yes	NA	NA
Pavir.5NG405300.v	GO:000557	00906(Car	zinc finger f	40.743382	80.625464	0.9518453	18.587219	6.2115996	2.6349648	0.018995315	0.252016591	up	yes	GDSL ester	At1g28600;At1g28600
Pavir.7NG124200.v	GO:000573	NA	alcohol deh	4.228645	25.832685	2.359137	2.490753	6.198353	2.6318849	0.003087132	0.095166652	up	yes	Chloroplast	CEQORH
Pavir.4NG186300.v	GO:003463	00564(Glyc	phospholipa	2.263319	10.460105	0.678909	1.374053	6.1975935	2.6317081	0.003177694	0.097306858	up	yes	Phospholip	PLD2
Pavir.9KG216600.v	GO:000582	NA	ring finger p	3.099521	24.309723	1.267853	3.160506	6.189481	2.6298184	0.007988902	0.162515989	up	yes	E3 ubiquiti	AIRP2
Pavir.1KG230100.v	NA	NA	duf761-ass	7.1378577	14.121187	0.0961618	3.3448858	6.1780734	2.627157	0.023323214	0.281323632	up	yes	NA	NA
Pavir.7NG350600.v	GO:000842	00500(Star	glycosyl hy	35.869213	126.82273	6.189026	20.154509	6.1757826	2.626622	0.003691131	0.107064137	up	yes	Probable in	BGLU14
Pavir.3NG216100.v	GO:000027	NA	chloroplast	40.061295	157.44527	5.844062	26.171196	6.1691385	2.625069	0.007758813	0.160572041	up	yes	Chloroplast	CSP41B
Pavir.1NG048800.v	NA	NA	0	2.1710769	4.2928421	0.37861	0.6703817	6.1620306	2.6234058	0.007914899	0.162028857	up	yes	NA	NA
Pavir.6KG230800.v	GO:000815	NA	plant family	2.3870307	13.081022	0.6713707	1.843999	6.1494152	2.6204492	0.004617428	0.122509957	up	yes	Protein unc	PATROL1;PATROL1;PATROL1
Pavir.2KG506600.v	NA	NA	0	54.412643	56.478893	1.079253	16.955154	6.1488873	2.6203254	0.015153169	0.225574491	up	yes	NA	NA
Pavir.3NG165300.v	NA	NA	0	1.295401	3.771464	0.284624	0.540594	6.1400321	2.6182462	0.013010787	0.20735682	up	yes	NA	NA
Pavir.6KG177700.v	NA	NA	0	2.813109	9.434235	0.608502	1.389058	6.131152	2.6161582	0.008578652	0.168680678	up	yes	NA	NA
Pavir.7KG099000.v	GO:000561	NA	thioredoxin	20.31743	35.986492	1.058126	8.13349	6.1255738	2.614845	0.005743186	0.136316308	up	yes	NA	NA
Pavir.2KG544400.v	GO:000367	03018(RN	wd40 repea	19.79217	62.132076	2.00518	11.378478	6.1212148	2.613818	0.008000083	0.162529436	up	yes	NA	NA
Pavir.5KG282300.v	NA	NA	npr1 interac	11.370765	19.574078	0.745126	4.310647	6.1206947	2.6136954	0.006216048	0.143461661	up	yes	NA	NA
Pavir.1KG414500.v	GO:000551	00910(Nitr	cytochrome	5.728216	14.681089	0.440574	2.894705	6.1192197	2.6133477	0.012607422	0.204092491	up	yes	Cytochrom	CYTB5-E
Pavir.7KG017400.v	GO:000573	00750(Vita	aldo/keto re	1.460024	4.37181	0.327378	0.627678	6.1062744	2.6102924	0.009658318	0.179649498	up	yes	Probable al	Os04g0338000
Pavir.5KG153100.v	GO:000367	NA	protein of u	210.18396	241.41228	1.81183	72.146568	6.1060846	2.6102476	0.024549378	0.28973757	up	yes	NA	NA
Pavir.1NG159200.v	GO:000579	NA	protein tric	4.075731	4.327094	0.379563	0.996998	6.1042155	2.6098059	0.003705081	0.107250404	up	yes	Protein tric	TBL24
Pavir.3KG387800.v	GO:000098	04626(Plan	wrky dna -b	4.771502	34.435986	2.445193	3.979506	6.1026187	2.6094284	0.004411616	0.118652576	up	yes	Probable W	WRKY46
Pavir.8KG337300.v	NA	NA	0	3.016718	6.11884	0.202941	1.295009	6.0987069	2.6085034	0.017092225	0.240475103	up	yes	NA	NA
Pavir.5KG054400.v	GO:000037	NA	nac domain	26.157356	50.030388	1.279078	11.218348	6.0962749	2.607928	0.010041529	0.181875162	up	yes	NAC doma	NAC075
Pavir.5KG156600.v	GO:000367	NA	protein of u	199.1571	232.28473	1.870827	68.915314	6.0950043	2.6076272	0.023559095	0.282626396	up	yes	NA	NA
Pavir.7KG374200.v	GO:000367	NA	protein of u	59.780369	69.225288	1.329839	19.836411	6.0948754	2.6075967	0.014649521	0.220258684	up	yes	NA	NA
Pavir.5KG774100.v	NA	NA	aba/wds ind	51.219696	54.883068	3.156425	14.255175	6.0937975	2.6073416	0.002403309	0.08062458	up	yes	Abscisic str	ASR3

Pavir.6KG015700.v4	GO:000367	03060(Prot	agamous-lik	17.12512	9.107202	0.639222	3.667987	6.0903295	2.6065203	0.00626418	0.144060155	up	yes	MADS-box	MADS26
Pavir.9NG650200.v4	GO:000002	03010(Ribo	40s ribosom	7.13753	14.625868	1.802527	1.772279	6.0879941	2.605967	0.00699678	0.153378065	up	yes	40S ribosor	RPS17D
Pavir.1NG481900.v4	GO:000367	04075(Plan	erebp-like fa	31.527809	98.043007	4.202272	17.101078	6.0821803	2.6045886	0.006433793	0.145712426	up	yes	Ethylene-res	RAP2-4
Pavir.5NG158800.v4	GO:000535	NA	mfs transpo	1.245372	3.702279	0.405721	0.408578	6.0759635	2.6031132	0.007651737	0.159279073	up	yes	Sugar transp	At1g54730
Pavir.2KG163100.v4	GO:004746	00909(Sesq	(-)-germacre	8.221928	21.85589	0.184048	4.772733	6.0680143	2.6012245	0.022605366	0.277230089	up	yes	(E)-beta-car	TPS23
Pavir.1KG285300.v4	GO:000016	01230(Bios	anthranilate	6.8465305	7.848508	0.4249995	2.0040446	6.0497209	2.5968686	0.005635632	0.135301987	up	yes	Anthranilat	ASB2;ASB2;ASB2
Pavir.7NG315600.v4	GO:000563	NA	platz transcr	4.748689	13.730161	1.011655	2.049302	6.0369518	2.5938203	0.005763033	0.136614798	up	yes	NA	NA
Pavir.7KG395600.v4	GO:000367	NA	mkiaa1688	3.737024	6.409606	0.127463	1.55458	6.032325	2.5927142	0.012010784	0.19862728	up	yes	CRIB doma	RIC7
Pavir.6NG174200.v4	GO:000550	00073(Cuti	caleosin-rela	2.504838	2.861492	0.166531	0.723095	6.0321191	2.5926649	0.009137307	0.174757488	up	yes	Peroxygena	-
Pavir.4KG270600.v4	GO:000550	00053(Asco	inositol oxy	2.4513002	50.08508	3.1199743	5.6023753	6.0231913	2.5905281	0.01510048	0.225146951	up	yes	Probable ino	Os06g0561000;Os06g0561000;Os06g0561000
Pavir.1NG030600.v4	GO:003146	04120(Ubic	cullin	2.295401	7.644498	0.141639	1.513086	6.0069794	2.5866397	0.026619837	0.303797098	up	yes	Cullin-3B C	CUL3B
Pavir.5KG246900.v4	GO:000017	NA	protein of un	1.22954	2.397987	0.36325	0.24202	5.9932377	2.5833356	0.00627737	0.144060155	up	yes	DUF724 do	DUF7
Pavir.4KG335800.v4	GO:001602	00904(Dite	premnaspiro	3.15752	9.973699	1.077752	1.114272	5.990454	2.5826654	0.002635721	0.085741949	up	yes	Ent-cassadi	CYP71Z7
Pavir.7KG327600.v4	GO:000484	NA	ubiquitin th	1.03	2.731956	0.393865	0.234404	5.987811	2.5820287	0.011336668	0.192738746	up	yes	NA	NA
Pavir.5NG294200.v4	GO:000561	04144(Endo	cupin doma	135.22958	134.7816	5.732198	39.365044	5.9873104	2.5819081	0.008485453	0.167537523	up	yes	Germin-like	Os05g0277500
Pavir.9NG046800.v4	GO:000367	NA	protein of un	9.069437	3.126899	0.277903	1.760363	5.9836822	2.5810336	0.009027962	0.173633972	up	yes	NA	NA
Pavir.6NG352900.v4	GO:000367	00190(Oxic	myb domain	2.5876692	22.535323	1.4793514	2.7235432	5.9775451	2.5795531	0.006808812	0.150570354	up	yes	Protein OD	ODO1;ODO1
Pavir.7NG293300.v4	GO:000425	00240(Pyri	rhomboid-re	28.359526	50.981876	1.143519	12.140038	5.9729033	2.5784324	0.012070813	0.199269188	up	yes	RHOMBOI	RBL1
Pavir.5KG323100.v4	GO:000588	NA	mlo-like pro	2.907115	16.051439	0.972098	2.205465	5.9663818	2.5768563	0.00709246	0.154753199	up	yes	MLO-like p	MLO1
Pavir.9NG739900.v4	NA	NA	0	22.992146	142.11787	4.33601	23.34347	5.9650694	2.5765389	0.015600737	0.229110202	up	yes	NA	NA
Pavir.8NG149800.v4	GO:000950	NA	chalcone--fl	9.511774	29.513006	0.530828	6.011508	5.9649611	2.5765127	0.013478447	0.210660638	up	yes	Probable ch	CHI3
Pavir.9KG015400.v4	NA	NA	0	1.970363	6.382577	0.214794	1.185638	5.9645452	2.5764121	0.016778761	0.238167128	up	yes	PGR5-like	PGRL1B
Pavir.9KG650000.v4	GO:000588	04626(Plan	leucine rich	1.504967	7.689474	0.110021	1.431738	5.9636046	2.5761846	0.027383384	0.307954473	up	yes	Putative leu	At2g14440
Pavir.9KG063600.v4	GO:000367	NA	protein of un	7.34941	16.308676	0.714765	3.256878	5.9567504	2.5745255	0.016092078	0.232779784	up	yes	NA	NA
Pavir.5KG331000.v4	NA	NA	duf761-asso	7.791162	14.934368	0.147947	3.668202	5.955095	2.5741245	0.020759109	0.264849578	up	yes	NA	NA
Pavir.5KG375600.v4	GO:000367	00190(Oxic	atp synthase	4.554345	20.590258	1.744819	2.480448	5.9510092	2.5731344	0.003221692	0.098094516	up	yes	GLABRA2	GEM
Pavir.1KG557800.v4	NA	NA	camp-respo	7.412744	38.901253	2.545533	5.238894	5.9495705	2.5727855	0.006829192	0.150702417	up	yes	NA	NA
Pavir.9KG454200.v4	GO:001602	00380(Tryp	flavonoid 3'	4.314861	8.757757	0.247159	1.950555	5.9482799	2.5724725	0.010412034	0.185224526	up	yes	Trimethyltri	CYP92C6
Pavir.3KG303000.v4	GO:000467	04626(Plan	lrr receptor-	8.701373	19.739685	1.11623	3.675689	5.9352126	2.5692997	0.002409869	0.080772523	up	yes	Protein MA	MDIS2
Pavir.7NG149300.v4	NA	NA	0	1.9096887	5.3096777	0.2287713	0.9879323	5.933546	2.5688945	0.015493611	0.228201277	up	yes	Protein PYI	PI21;NA;PI21
Pavir.2NG502300.v4	GO:000981	00942(Anth	glucosyl/glu	3.077199	9.384214	0.678315	1.422568	5.9315121	2.5683999	0.003533595	0.104982088	up	yes	UDP-glycos	UGT71K1
Pavir.5KG074600.v4	GO:001678	NA	alpha/beta h	79.95108	62.234631	2.318146	21.663351	5.9289756	2.5677829	0.010886991	0.188711705	up	yes	NA	NA
Pavir.2NG357400.v4	NA	NA	0	8.540058	3.541446	0.352529	1.692464	5.9078461	2.5626323	0.009048567	0.173814838	up	yes	NA	NA
Pavir.4KG356500.v4	GO:000013	NA	vacuolar-so	59.545624	84.475288	0.59082	23.792669	5.9064932	2.5623018	0.03506238	0.352186433	up	yes	Vacuolar-so	VSR6
Pavir.5KG119200.v4	GO:000452	NA	ribonuclease	3.9379699	12.195094	1.2325379	1.4996609	5.9047912	2.561886	0.001477784	0.059909295	up	yes	Factor of D	FDM5;FDM5
Pavir.5KG402600.v4	GO:000367	NA	protein of un	7.30375	32.632156	2.077767	4.687815	5.9028042	2.5614005	0.003380557	0.101309708	up	yes	NA	NA
Pavir.3KG549800.v4	GO:000367	00230(Puri	transcription	16.527591	5.0288659	1.9512814	1.702727	5.8994	2.5605682	0.001535815	0.061388481	up	yes	Nuclear tran	NFYA10;NFYA10;NFYA10
Pavir.4KG188600.v4	NA	NA	0	1.3014186	1.147473	0.114518	0.301638	5.8845519	2.5569326	0.015294047	0.226415765	up	yes	NA	NA
Pavir.3KG562300.v4	GO:000467	04075(Plan	leucine-rich	3.593646	2.347336	0.260239	0.749473	5.8838382	2.5567576	0.010376616	0.185216433	up	yes	Receptor-lik	RLK7
Pavir.5KG487800.v4	GO:000467	04626(Plan	protein kina	4.3701018	9.7301987	0.354596	2.0433862	5.8800689	2.5558331	0.005530686	0.134588386	up	yes	LEAF RUS	LRK10L-1.2;LRK10L-1.2;LRK10L-1.2;LRK10L-1.2
Pavir.9NG733300.v4	GO:000367	NA	gb	20.959894	12.487882	1.901779	3.790725	5.875758	2.554775	0.001127871	0.049854415	up	yes	NA	NA
Pavir.5NG397400.v4	NA	NA	0	1.515903	8.576015	0.750879	0.967246	5.8737973	2.5542935	0.017564267	0.243072641	up	yes	NA	NA
Pavir.5NG617200.v4	NA	NA	0	18.777054	66.088013	8.357635	6.097873	5.8707772	2.5535515	0.001394978	0.057724136	up	yes	Flowering-p	RAA1
Pavir.9NG006600.v4	GO:000551	NA	uncharacteri	3.5778951	13.489837	1.1416526	1.7673152	5.867281	2.5526921	0.003630513	0.106403492	up	yes	Pirin-like pr	-;-;-
Pavir.9KG319200.v4	GO:000827	NA	ring finger c	6.9552791	3.6818178	0.2700395	1.5429557	5.86714	2.5526574	0.009671065	0.17970867	up	yes	NEP1-intera	NIP2;NA
Pavir.3NG290200.v4	GO:000551	NA	genomic dna	3.770415	1.026318	0.45119	0.366414	5.8668169	2.552578	0.012396667	0.202249228	up	yes	VAN3-bind	VAB;VAB;VAB
Pavir.9NG209700.v4	NA	NA	shn shine ,	2.233752	2.158179	0.261087	0.488371	5.8601429	2.5509359	0.012557968	0.203863881	up	yes	NA	NA
Pavir.2NG002300.v4	GO:000460	00940(Pher	peroxidase 1	19.943405	41.264656	0.358282	10.099608	5.8528117	2.5491299	0.0241172	0.286758091	up	yes	Peroxidase	PER51
Pavir.2NG386900.v4	GO:000551	NA	uncharacteri	7.9524196	30.848003	0.7429561	5.8957913	5.8445397	2.5470894	0.011778114	0.196771493	up	yes	Pirin-like pr	At1g50590;At1g50590
Pavir.1KG111600.v4	GO:000367	NA	protein tag-	1.154264	6.758042	0.415118	0.940723	5.8357182	2.5449102	0.020490119	0.262666409	up	yes	NA	NA
Pavir.5NG266500.v4	GO:000579	NA	beta-1,3-n-a	8.582861	6.797576	0.517978	2.119408	5.8316974	2.5439159	0.004440826	0.119267445	up	yes	Hydroxypro	GALT4
Pavir.2NG578800.v4	GO:000588	NA	cobra-like p	1.234337	3.568963	0.311163	0.513039	5.8278189	2.542956	0.010038992	0.181875162	up	yes	COBRA-lik	BC1L6
Pavir.5KG642600.v4	GO:000695	NA	lurp-one-rel	1.440561	3.688468	0.408143	0.475771	5.8026335	2.5367078	0.00896357	0.173155886	up	yes	Protein LUI	At1g80120
Pavir.4NG280000.v4	GO:000367	NA	protein of un	3.244538	6.917479	0.272069	1.481433	5.7952697	2.5348758	0.017353913	0.24174101	up	yes	NA	NA
Pavir.6KG316900.v4	NA	NA	camp-respo	3.069904	6.904428	0.094124	1.629895	5.7855116	2.5324446	0.028671602	0.31490152	up	yes	NA	NA
Pavir.8KG179200.v4	GO:000563	NA	speckle-type	4.826764	1.709516	0.084001	1.047957	5.7743132	2.5296494	0.035426505	0.3540943	up	yes	BTB/POZ c	At3g56230
Pavir.2KG502900.v4	NA	NA	0	3.70189	9.682156	0.11667	2.202066	5.7721302	2.5291038	0.031705759	0.333684044	up	yes	NA	NA
Pavir.7KG125800.v4	NA	NA	0	6.85534	26.72286	0.816028	5.017119	5.7564467	2.5251786	0.009329607	0.176487135	up	yes	NA	NA
Pavir.4NG273100.v4	GO:000013	NA	vacuolar-so	84.454361	81.807991	0.68675	28.196266	5.7564055	2.5251682	0.037171339	0.363235039	up	yes	Vacuolar-so	VSR6
Pavir.2KG586700.v4	NA	NA	probable zin	1.345472	1.901435	0.114998	0.449327	5.7536118	2.5244679	0.013478513	0.210660638	up	yes	NA	NA
Pavir.9NG839800.v4	NA	NA	0	2.100941	4.013392	0.303727	0.759725	5.7495148	2.5234402	0.009379209	0.176821386	up	yes	NA	NA
Pavir.2KG329800.v4	GO:000480	00500(Starc	trehalose-ph	22.130854	75.425293	4.475045	12.499905	5.7470654	2.5228255	0.005519863	0.13458652	up	yes	Probable tre	TPP7
Pavir.3KG175200.v4	GO:000557	04144(Endo	zinc finger f	13.278944	13.547354	0.4343437	4.2435932	5.734643	2.5197037	0.008933718	0.172943049	up	yes	GDSL ester	At1g09390;At1g09390
Pavir.1KG012500.v4	GO:001014	00561(Glyc	glycerol-3-p	1.554024	2.714444	0.461251	0.283249	5.7333351	2.5193746	0.007372743	0.156860734	up	yes	Glycerol-3-	GPAT4
Pavir.2KG335100.v4	GO:000367	NA	tetraspanin-	11.552192	20.753094	0.1576	5.4876	5.7226114	2.5166736	0.030339088	0.325339676	up	yes	Tetraspanin	TET8
Pavir.1KG189900.v4	GO:000472	NA	protein phos	12.038278	65.515999	3.956063	9.605669	5.7186115	2.5156649	0.007705791	0.160138105	up	yes	Probable pr	Os02g0224100
Pavir.3NG083500.v4	NA	NA	0	4.196158	4.140268	0.214186	1.244337	5.715663	2.5149209	0.0105827	0.186590239	up	yes	NA	NA
Pavir.5NG418200.v4	NA	NA	protein of un	6.339207	18.395342	0.341062	3.987805	5.7138621	2.5144662	0.01777589	0.24402394	up	yes	NA	NA
Pavir.2KG584300.v4	GO:000551	NA	ricin-type be	16.23831	29.096697	0.263186	7.678059	5.7088035	2.5131884	0.027087328	0.306459558	up	yes	Ricin B-like	R40G3
Pavir.3KG165000.v4	GO:000973	04075(Plan	auxin-respo	2.010005	22.549612	1.154514	3.149154	5.7066709	2.5126494	0.014588127	0.219883759	up	yes	Auxin-respo	IAA16
Pavir.5KG280000.v4	GO:000467	00520(Ami	salt stress re	1.526152	18.983336	1.512574	2.085064	5.7008204	2.5111696	0.010888856	0.188711705	up	yes	Cysteine-ric	CRK6;CRK6
Pavir.5NG510300.v4	GO:000436	00480(Glut	glutathione	6.864182	4.762298	0.217971	1.822374	5.6982912	2.5105294	0.020286844	0.261219183	up	yes	Glutathione	GST1
Pavir.1KG284700.v4	GO:001649	NA	cytochrome	0.752241	46.758526	2.670627	5.668131	5.6975831	2.5103501	0.039090829	0.372687063	up	yes	Probable tra	CYB561C
Pavir.5KG595400.v4	GO:000551	04075(Plan	basic region	1.518964	22.987823	3.019189	1.282429	5.6971091	2.51023	0.013658713	0.212161115	up	yes	Transcriptic	TGAL1;TGAL1
Pavir.2NG379900.v4	GO:000370	04626(Plan	wrky transcr	3.789769	5.324873	0.855414	0.75036	5.6761674	2.5049171	0.001612179	0.063164138	up	yes	Probable W	WRKY2
Pavir.9NG606600.v4	GO:000645	04141(Prote	dnaj homolo	5.153377	21.700226	1.326558	3.407156	5.6728402	2.5040712	0.009771073	0.180534287	up	yes	Chaperone	ATJ2
Pavir.9NG735300.v4	GO:000367	NA	bel1-like ho	37.956344	42.537401	0.363817	13.831083	5.6706101	2.503504	0.035983632	0.356637568	up	yes	BEL1-like l	BLH7;BLH7
Pavir.1NG480200.v4	GO:000470	04626(Plan	mitogen-act	1.550992	1.30319	0.057192	0.446269	5.6691223	2.5031254	0.027185032	0.306918028	up	yes	Mitogen-act	MEKK1
Pavir.6NG168000.v4	NA	NA	0	3.635193	9.434669	0.169822	2.142612	5.6519935	2.4987598	0.019808523	0.257977684	up	yes	Phosphopar	Os08g0176100
Pavir.7KG274100.v4	NA	NA	histone h1/h	3.423904	6.974322	0.354649	1.489974	5.6370467	2.4949395	0.008230232	0.164749334	up	yes	NA	NA
Pavir.5NG201300.v4	GO:000472	NA	calcineurin l	12.460012	28.245567	1.3124114	5.9096596	5.6362751	2.494742	0.00520026	0.130321762	up	yes	Calcineurin	CBL4;CBL4
Pavir.4KG404200.v4	GO:000367	NA	crib domain	20.435088	19.203036	0.8075504	6.2262316	5.6353927	2.4945162	0.007760861	0.160572041	up	yes	CRIB doma	RIC10;RIC10

Pavir.2KG102100.v4	GO:000588	01200(Carb	eama-like tr	3.3665622	16.254282	0.9941635	2.492716	5.6270497	2.4923787	0.006079775	0.141348194	up	yes	WAT1-rela	At5g64700;At5g64700;At2g37460;At2g37460;At5g64700
Pavir.9NG388700.v4	GO:000582	NA	ring finger p	10.243272	60.609361	6.3425508	6.2541127	5.6247143	2.4917798	0.003786334	0.108898049	up	yes	E3 ubiquiti	AIRP2;AIRP2
Pavir.8KG163800.v4	GO:000015	NA	ariadne-1 (a	1.9332345	5.8761485	0.5633315	0.8254479	5.623199	2.4913911	0.003210919	0.097984464	up	yes	Probable E3	ARI5;ARI5;ARI5;ARI5;ARI5
Pavir.1NG463200.v4	GO:000864	NA	gb	3.1751006	3.9632108	0.6614821	0.6080016	5.6230039	2.491341	0.004114837	0.114404397	up	yes	Nucleotide-	At5g55950;At5g55950
Pavir.5KG273100.v4	GO:000367	00190(Oxic	myb-like dn	7.8735656	27.188645	1.1666596	5.0753063	5.6171743	2.4898446	0.006835792	0.150728417	up	yes	Myb-relatec	MYBAS2;MYBAS2
Pavir.9KG222000.v4	NA	NA	0	19.415836	90.702377	3.302772	16.312136	5.6140061	2.4890306	0.012994546	0.20735682	up	yes	NA	NA
Pavir.3KG348400.v4	GO:000471	04075(Plan	serine-threo	10.718914	11.658961	0.55045	3.4392756	5.6088758	2.4877116	0.005491327	0.134151906	up	yes	Serine/threc	HT1;HT1
Pavir.8NG093800.v4	GO:000819	00908(Zeat	glucosyl/glu	1.712034	8.820929	1.04468	0.841812	5.5833595	2.4811334	0.005543007	0.134626641	up	yes	UDP-glycos	UGT73E1
Pavir.4NG193100.v4	GO:000573	00073(Cuti	transferase f	6.75562	13.676194	0.398198	3.266523	5.5752714	2.479042	0.01094432	0.189203355	up	yes	Acyl transfe	AT7
Pavir.2KG487300.v4	GO:000367	NA	protein of u	19.804594	42.221149	1.251974	9.883895	5.5699059	2.477653	0.009182691	0.1749902	up	yes	NA	NA
Pavir.1NG497100.v4	GO:000480	00500(Starc	trehalose-ph	2.773837	1.012106	0.257393	0.42265	5.5672112	2.4769548	0.012986929	0.20735682	up	yes	Probable tre	TPP4
Pavir.1NG368700.v4	GO:001250	NA	membrane-a	3.3162394	27.16626	1.3913562	4.0982597	5.5527564	2.4732041	0.011225059	0.191883132	up	yes	Membrane s	MSBP1;MSBP1;MSBP1;MSBP1
Pavir.9NG746800.v4	GO:000588	01200(Carb	eama-like tr	15.476906	8.993664	0.710048	3.697376	5.5521252	2.4730401	0.005839768	0.137393622	up	yes	WAT1-rela	At5g07050
Pavir.6KG326700.v4	GO:000367	NA	tetraspanin-	8.851334	17.222054	0.126335	4.571045	5.5506235	2.4726498	0.040999522	0.380928544	up	yes	Tetraspanin	TET8
Pavir.5KG771500.v4	GO:000460	00564(Glyc	phosphatidy	1.415044	16.074635	2.118556	1.038252	5.540305	2.4699654	0.011282379	0.19242525	up	yes	Phosphatidy	PSD2
Pavir.5NG028100.v4	GO:000098	NA	b-box zinc f	1.112256	4.076058	0.356331	0.580751	5.5366702	2.4690186	0.012316898	0.201385036	up	yes	B-box zinc	BBX22
Pavir.1NG400500.v4	GO:000367	NA	bzip protein	3.479583	1.498456	0.333627	0.565484	5.5366234	2.4690064	0.010711837	0.187634436	up	yes	Basic leucir	BZIP43
Pavir.6NG345900.v4	GO:000573	NA	bcl2-associa	115.07489	101.50698	7.4250753	31.700824	5.5355118	2.4687167	0.006698706	0.148753231	up	yes	BAG family	BAG1;BAG1
Pavir.5KG031400.v4	GO:000557	00906(Carc	zinc finger f	50.170834	35.616894	1.923661	13.615332	5.5208036	2.4648783	0.00996273	0.181831401	up	yes	GDSL ester	At5g45910
Pavir.2KG525800.v4	NA	NA	0	3.128097	18.7444	0.161868	3.802242	5.5176312	2.464049	0.04757558	0.41119236	up	yes	NA	NA
Pavir.3KG575400.v4	NA	NA	0	9.085445	17.866467	0.50562	4.384846	5.5111133	2.4623438	0.011406855	0.193578392	up	yes	NA	NA
Pavir.1NG340000.v4	GO:000104	04075(Plan	transcriptior	2.058428	1.390296	0.184677	0.441272	5.5095926	2.4619457	0.015658118	0.229569567	up	yes	Transcriptic	BHLH128
Pavir.1KG490800.v4	GO:000578	00600(Sphi	lag1 longev	10.152929	20.466337	1.051697	4.516869	5.4985908	2.4590619	0.004995389	0.127349589	up	yes	ASC1-like p	Os02g0728300
Pavir.5KG746600.v4	GO:000573	NA	copper trans	10.514035	25.338819	3.3372482	3.1848545	5.4971311	2.4586789	0.002220659	0.076758795	up	yes	NA	NA
Pavir.1KG378900.v4	GO:000551	00940(Pher	shikimate o-	94.591873	137.8667	6.476288	35.818268	5.4961819	2.4584297	0.009864752	0.181334796	up	yes	Hydroxycin	HCT2
Pavir.7NG355500.v4	GO:000551	00360(Pher	phenylalani	389.78119	1516.3582	90.649681	256.17203	5.4960208	2.4583875	0.010088535	0.182016394	up	yes	Phenylalani	PAL
Pavir.9NG001700.v4	GO:000367	NA	protein tag-	5.010557	30.96286	2.643766	3.908432	5.4902823	2.4568803	0.005514767	0.134549562	up	yes	NA	NA
Pavir.2NG376000.v4	GO:000370	04626(Plan	wrky transc	4.088075	4.342896	0.47764	1.058239	5.4893458	2.4566342	0.002560075	0.084229153	up	yes	Probable W	WRKY2
Pavir.2KG575300.v4	GO:000563	NA	ring finger p	13.433791	41.982876	3.1766344	6.9479537	5.4734738	2.4524567	0.00282978	0.089876029	up	yes	E3 ubiquiti	AIRP2;AIRP2
Pavir.8KG126500.v4	GO:000487	NA	root phototr	14.69516	7.5188383	1.072732	2.989556	5.4683465	2.4511047	0.002588828	0.084951983	up	yes	Root photot	RPT2;RPT2
Pavir.4NG036800.v4	GO:000573	00073(Cuti	n-hydroxyci	1.732608	4.161477	0.592444	0.485578	5.4674997	2.4508812	0.013488628	0.210731073	up	yes	3'-N-debenz	TAX10
Pavir.9NG723800.v4	GO:000367	NA	gb	16.74684	12.253478	1.74988	3.555566	5.4661414	2.4505228	0.001316478	0.055676155	up	yes	NA	NA
Pavir.7KG106100.v4	GO:000367	00240(Pyri	uncharacteri	2.1965732	5.9173526	0.4119386	1.0736656	5.4617011	2.4493504	0.007304046	0.156441785	up	yes	UNC93-like	At1g18000;At1g18000;At1g18000
Pavir.1KG325400.v4	GO:000467	04626(Plan	transmembr	1.623275	8.138113	0.70621	1.082098	5.458449	2.4484911	0.007413039	0.157450594	up	yes	Probable se	At1g01540
Pavir.1KG122000.v4	GO:000003	01212(Fatty	fatty acyl-ac	6.3620279	23.155999	0.686226	4.7221898	5.4577955	2.4483183	0.01205441	0.199099616	up	yes	Palmitoyl-a	FATB;FATB;FATB
Pavir.1KG014300.v4	GO:000484	04141(Prot	ubiquitin-cc	5.823719	9.873451	0.385615	2.490516	5.4577382	2.4483032	0.017169843	0.240987112	up	yes	Ubiquitin-c	UBC28
Pavir.9KG036600.v4	NA	NA	0	5.480893	3.19183	0.247494	1.342687	5.4539219	2.447294	0.022807188	0.27876854	up	yes	NA	NA
Pavir.2NG003000.v4	GO:000551	NA	mads-box p	2.326778	11.638151	1.669614	0.891385	5.4529225	2.4470296	0.01050841	0.185982266	up	yes	MADS-box	MADS15
Pavir.8KG022400.v4	GO:000950	NA	chalcone--fl	36.583126	82.701729	1.455355	20.423256	5.4521219	2.4468178	0.020928854	0.266201702	up	yes	Probable ch	CHI3
Pavir.8NG040600.v4	GO:000695	NA	lurp-one-rel	1.440616	1.20035	0.261009	0.223423	5.4516754	2.4466997	0.014553619	0.219872592	up	yes	Protein LUI	At2g38640
Pavir.5NG163200.v4	NA	NA	0	6.1829701	0.8947988	0.2249677	1.0751403	5.4439854	2.4446632	0.032707601	0.339013157	up	yes	NA	NA
Pavir.5NG214500.v4	GO:000552	NA	universal st	2.533643	24.19817	2.408357	2.505244	5.4403711	2.4437051	0.012256544	0.200835208	up	yes	NA	NA
Pavir.1NG523800.v4	GO:000465	00040(Pent	pectate lyas	54.124393	68.567703	4.488522	18.075447	5.4375228	2.4429495	0.006443131	0.145748209	up	yes	Probable pc	GSVIVT00026920001
Pavir.9NG490600.v4	GO:000563	NA	gb	1.839897	4.550267	0.408243	0.768623	5.4298144	2.4409029	0.014215735	0.216948747	up	yes	Transcriptic	OFP8
Pavir.3KG216500.v4	GO:000367	NA	zinc finger c	25.396628	2.899083	0.5852513	4.6297331	5.4258476	2.4398485	0.025778899	0.297966779	up	yes	Zinc finger	Os05g0576300;Os05g0576300
Pavir.4KG409900.v4	GO:000455	00052(Gala	alpha-gluco	0.868681	5.308999	0.056001	1.082614	5.4256092	2.4397851	0.046139857	0.404713246	up	yes	Probable al	Os06g0675700
Pavir.1NG017800.v4	NA	NA	0	6.860123	78.358307	8.255157	7.478154	5.4164333	2.4373432	0.010334211	0.184810707	up	yes	NA	NA
Pavir.3NG066600.v4	GO:000013	NA	glucuronoxy	96.77314	131.91286	6.705645	35.527958	5.4147878	2.4369048	0.009700401	0.180021127	up	yes	Glucuronox	GXM3
Pavir.5NG044800.v4	GO:000098	NA	b-box zinc f	1.169	4.143335	0.382267	0.599637	5.4102387	2.4356922	0.013229361	0.209027242	up	yes	B-box zinc	BBX22
Pavir.4KG314200.v4	GO:000973	04075(Plan	auxin-respo	3.877817	3.795789	0.555485	0.863806	5.4066474	2.4347343	0.005873878	0.137937014	up	yes	Auxin-respo	IAA23
Pavir.1KG363300.v4	GO:000487	00902(Mon	root phototr	1.5678	2.030015	0.121631	0.543977	5.4053061	2.4343763	0.015655451	0.229569567	up	yes	Coleoptile p	CPT1
Pavir.9KG546800.v4	NA	NA	0	5.072517	5.844104	0.728091	1.291932	5.4042063	2.4340827	0.005587913	0.134904319	up	yes	NA	NA
Pavir.8KG391400.v4	GO:000367	00190(Oxic	transcriptior	1.6103763	7.8000919	0.5226742	1.2190648	5.4029152	2.433738	0.018092885	0.245773676	up	yes	Myb-relatec	MYBAS1;MYBAS1;MYBAS1
Pavir.1KG238100.v4	GO:000472	NA	protein phos	2.89484	5.368364	0.560171	0.970446	5.3986098	2.432588	0.004233019	0.116432812	up	yes	Probable pr	Os05g0457200
Pavir.2KG277300.v4	GO:000484	04141(Prot	ubiquitin-cc	13.19797	12.040297	1.6646266	3.0137739	5.3946357	2.4315255	0.001064531	0.048233626	up	yes	Ubiquitin-c	UBC5A;UBC5A;UBC5A
Pavir.2NG418600.v4	NA	NA	0	1.289435	2.176864	0.250532	0.392486	5.3906718	2.4304651	0.013963165	0.214575875	up	yes	NA	NA
Pavir.3KG305500.v4	GO:000370	NA	no apical m	2.300487	13.49907	0.140266	2.791989	5.3881934	2.4298016	0.036803592	0.360955723	up	yes	NAC domai	NAC073
Pavir.1KG182800.v4	GO:000563	NA	reticulon//su	30.955933	53.201775	1.936876	13.684351	5.3873942	2.4295876	0.011534466	0.194867121	up	yes	Reticulon-li	RTNLB2
Pavir.6NG141400.v4	GO:004748	04626(Plan	mekk and re	1.449944	3.814616	0.394224	0.583766	5.3830407	2.4284213	0.013518169	0.211104813	up	yes	Mitogen-act	MKK3
Pavir.3KG148400.v4	NA	NA	0	1.0027638	1.6227565	0.1494424	0.3389381	5.3759722	2.4265257	0.016749331	0.237839229	up	yes	Coiled-coil	SCD2;SCD2;SCD2
Pavir.4KG105500.v4	GO:000557	00514(Othe	glycosyltran	26.149843	23.871634	0.426747	8.893044	5.3672316	2.4241781	0.025043091	0.293586289	up	yes	Beta-1,2-xy	XAX1
Pavir.6KG070300.v4	NA	NA	0	482.03363	633.44147	21.734776	186.42696	5.3586943	2.4218815	0.017205353	0.241064917	up	yes	Caffeic acid	-
Pavir.7KG242100.v4	GO:000377	04144(Endo	actin-relatec	66.465652	92.362	1.102809	28.537453	5.3585104	2.421832	0.034729051	0.350391522	up	yes	Actin-relate	ARPC2B
Pavir.9KG420200.v4	NA	NA	0	3.279707	7.514328	0.64777	1.367682	5.3556398	2.4210589	0.011691768	0.196113758	up	yes	NA	NA
Pavir.9KG077400.v4	GO:000467	04140(Regu	serine/threo	9.6188272	17.461923	1.4058238	3.6534324	5.3527137	2.4202705	0.002146555	0.075165439	up	yes	CBL-intera	CIPK7;CIPK7;CIPK7;CIPK7;CIPK7;CIPK7;CIPK7
Pavir.6KG254600.v4	GO:000443	00562(Inos	phosphatidy	15.916721	28.427338	2.3191202	5.989133	5.3373504	2.4161237	0.002252202	0.077392148	up	yes	Type IV inc	IP5P9;IP5P9;IP5P9;IP5P9
Pavir.8NG333500.v4	GO:000551	04626(Plan	leucine-rich	1.308227	2.4063948	0.0674264	0.6291981	5.332316	2.4147623	0.027695132	0.309361931	up	yes	Disease resi	RGA5;RGA5;RGA5;RGA5
Pavir.9KG309600.v4	GO:000436	00480(Glut	glutathione	1.170037	3.100246	0.375321	0.426918	5.3229561	2.4122277	0.018892403	0.251024069	up	yes	Probable gl	GSTU6
Pavir.9NG723500.v4	NA	NA	0	1.548985	7.976588	0.953513	0.839374	5.3129801	2.4095213	0.023641666	0.283435963	up	yes	NA	NA
Pavir.4KG402200.v4	GO:000467	00260(Glyc	protein kina	1.003207	1.796486	0.081193	0.446146	5.3090953	2.408466	0.024972648	0.293126303	up	yes	G-type lecti	At2g19130
Pavir.6NG244900.v4	NA	NA	0	8.380654	52.478001	2.225684	9.243676	5.3061945	2.4076776	0.013045502	0.207694349	up	yes	NA	NA
Pavir.1KG386100.v4	GO:000551	00360(Pher	phenylalani	2540.4807	5301.1812	219.36473	1259.6589	5.3019178	2.4065143	0.014301852	0.217732366	up	yes	Phenylalani	PAL
Pavir.7NG190200.v4	GO:000395	00592(alph	12-oxophyto	31.587322	16.068577	1.4577245	7.5369057	5.298261	2.4055189	0.006354801	0.144884055	up	yes	Putative 12-	OPR11;OPR11;OPR11
Pavir.7KG235600.v4	GO:000577	00531(Glyc	aquaporin tr	82.726204	37.446392	0.828636	21.853006	5.2982318	2.405511	0.037074323	0.362853226	up	yes	Aquaporin I	PIP2-5
Pavir.7KG323700.v4	GO:000563	NA	platz transc	24.698318	95.394745	8.734705	13.933976	5.2977526	2.4053805	0.006032524	0.140695229	up	yes	NA	NA
Pavir.7KG358600.v4	GO:000406	NA	asparagine s	16.588015	266.13303	17.141777	36.312695	5.2890063	2.4029967	0.026694869	0.30430913	up	yes	Stem-specif	TSJT1
Pavir.1KG273700.v4	NA	NA	iq calmodul	16.216919	51.86071	2.3511963	10.520876	5.2887856	2.4029365	0.010996572	0.189563798	up	yes	NA	NA
Pavir.6NG334100.v4	NA	NA	0	6.307249	36.774967	4.303417	3.847531	5.2855467	2.4020527	0.005857014	0.137713143	up	yes	NA	NA
Pavir.1NG316400.v4	NA	NA	0	4.072595	2.008751	0.195372	0.955777	5.2828487	2.4013161	0.012600687	0.204071389	up	yes	NA	NA
Pavir.6NG147600.v4	NA	NA	0	5.219808	26.938633	3.277331	2.813865	5.2794954	2.4004	0.007817956	0.160955646	up	yes	Cinnamoyl-	CCR2
Pavir.7KG264900.v4	GO:000666	00514(Othe	kdel lys-asp	3.337115	2.838689	0.076189	1.094423	5.2757054	2.399364	0.049557668	0.420417763	up	yes	NA	NA
Pavir.8NG116700.v4	GO:000013	NA	uncharacteri	13.157887	28.19985	4.73591	3.109487	5.2715926	2.3982389	0.002535203	0.083777632	up	yes	Protein REV	RTE1

Pavir.8KG068000.v4	GO:000370	04141(Prot	transcription	12.851509	39.507748	2.69128	7.249537	5.267098	2.3970083	0.004246882	0.116643333	up	yes	Transcriptic	VIP1
Pavir.7NG158800.v4	NA	NA	0	145.25095	209.42003	14.33424	53.109241	5.2587881	2.3947304	0.007480859	0.157697172	up	yes	NA	NA
Pavir.4KG378500.v4	GO:000550	00260(Glyc	choline dehy	2.1948627	2.6803728	0.1104205	0.8174259	5.2543566	2.3935141	0.026702877	0.30430913	up	yes	Choline mo	At4g29890;At4g29890;At4g29890;At4g29890;At4g29890
Pavir.8KG034800.v4	GO:000551	04075(Plan	btb/poz don	1.209246	1.4468155	0.315072	0.190821	5.2502433	2.3923843	0.010074431	0.182016394	up	yes	BTB/POZ c	NH5.2;NH5.2
Pavir.1KG331900.v4	GO:000467	04075(Plan	serine/threo	9.460741	13.637033	1.455147	2.947116	5.2467956	2.3914366	0.002083848	0.073868681	up	yes	Serine/threc	SAPK6
Pavir.1NG323300.v4	NA	NA	0	5.447392	16.065157	1.465886	2.637607	5.2424968	2.3902541	0.005060038	0.128340138	up	yes	NA	NA
Pavir.2NG349900.v4	GO:000578	00563(Glyc	transmembr	2.444597	3.395666	0.29434	0.820779	5.2373451	2.3888357	0.018818094	0.250638862	up	yes	Transmemb	At1g09580
Pavir.3KG104100.v4	NA	NA	0	26.995642	49.573826	2.229804	12.391448	5.2368612	2.3887024	0.007483905	0.157697172	up	yes	NA	NA
Pavir.7NG273900.v4	NA	NA	0	3.341247	17.671776	1.068604	2.944066	5.2366686	2.3886493	0.018893782	0.251024069	up	yes	NA	NA
Pavir.3KG246800.v4	GO:000521	NA	adenine/gua	2.164444	2.814177	0.327755	0.625057	5.2251871	2.3854827	0.008207724	0.164474037	up	yes	Adenine/gua	AZG1
Pavir.1KG468600.v4	GO:001678	NA	serine hydrc	10.618826	21.617432	1.4490941	4.7204965	5.2250238	2.3854376	0.00410309	0.114255493	up	yes	1-acylglycei	At4g24160;At4g24160
Pavir.6KG379300.v4	GO:000022	00190(Oxic	65-kda micr	18.033836	89.418037	2.900394	17.665051	5.2248747	2.3853964	0.021818398	0.271850514	up	yes	65-kDa mic	MAP65-8
Pavir.1KG454800.v4	GO:000367	NA	zinc-finger c	3.817005	2.193489	0.343516	0.808142	5.2189921	2.3837712	0.019183094	0.253701651	up	yes	FCS-Like Z	FLZ2
Pavir.9NG764300.v4	GO:000521	NA	oligopeptide	83.514191	229.82275	13.766749	46.33952	5.2130493	2.3821275	0.010047688	0.181875162	up	yes	Protein NR	NPF5.2
Pavir.9KG540600.v4	GO:000535	NA	mfs/sugar tr	5.214453	26.732584	3.668436	2.46409	5.2094418	2.3811288	0.004364186	0.118361397	up	yes	NA	NA
Pavir.7KG143400.v4	GO:000953	00053(Asco	l-ascorbate	6.5173463	7.775531	0.7138278	2.0377968	5.1943414	2.3769408	0.002941438	0.092329685	up	yes	Probable L-	APX8;APX8;APX8;APX8;APX8
Pavir.2NG126400.v4	GO:000367	NA	mediator of	16.301856	65.387109	4.6929672	11.036747	5.1932899	2.3766488	0.00993893	0.181831401	up	yes	Mediator of	MED33A;MED33A;MED33A;MED33A
Pavir.2NG597100.v4	GO:000367	NA	wd40 repeat	5.297873	4.452352	0.476303	1.401163	5.1932898	2.3766487	0.011998641	0.1986013	up	yes	NA	NA
Pavir.3KG035700.v4	GO:000487	NA	late embryo	20.208502	37.120399	0.605549	10.439874	5.1902857	2.375814	0.023533169	0.28240554	up	yes	NDR1/HIN	NHL1
Pavir.9NG167500.v4	GO:000484	NA	inhibitor of	1.614715	19.584206	1.887551	2.202371	5.1832091	2.3738456	0.015446617	0.227874188	up	yes	Probable B(	BRG3
Pavir.7KG001200.v4	NA	NA	0	5.293069	6.85178	1.985052	0.358274	5.1827398	2.373715	0.014417833	0.218700543	up	yes	Protein LA	LATE
Pavir.2KG487200.v4	NA	NA	wound-indu	9.292274	71.089142	4.883709	10.665084	5.1696242	2.3700594	0.010996055	0.189563798	up	yes	NA	NA
Pavir.2NG614200.v4	GO:000367	NA	wound-indu	25.808826	86.262344	6.214341	15.464647	5.1695757	2.3700459	0.004502295	0.120338805	up	yes	Wound-indu	WUN1
Pavir.4NG304700.v4	GO:000455	00052(Gala	alpha-gluco	1.673316	11.954233	0.159581	2.476869	5.168901	2.3698576	0.040608562	0.379595456	up	yes	Probable al	Os06g0675700
Pavir.9KG013000.v4	NA	NA	mkiaa1688	31.405647	9.878415	1.047925	6.947099	5.1637196	2.3684107	0.014449256	0.218912048	up	yes	NA	NA
Pavir.5NG457500.v4	GO:000367	NA	protein of u	2.405313	21.583374	2.659295	1.987419	5.1625056	2.3680714	0.019328812	0.254759839	up	yes	NA	NA
Pavir.5NG583900.v4	GO:000552	03040(Splic	heat shock 7	7.103147	20.682619	1.308114	4.075611	5.1610671	2.3676694	0.005064065	0.128340138	up	yes	Heat shock	HSP70
Pavir.8KG006500.v4	GO:000563	NA	speckle-type	9.3430378	3.6949837	0.1016209	2.4247604	5.1607498	2.3675807	0.042364596	0.388140692	up	yes	BTB/POZ c	At3g56230;At3g56230
Pavir.6NG118600.v4	GO:000562	NA	upf0041 bra	6.453099	9.443783	0.223113	2.857662	5.1600269	2.3673786	0.035275642	0.35344312	up	yes	Mitochondr	MPC1
Pavir.2KG202300.v4	NA	NA	0	6.853043	9.674085	1.218754	1.988946	5.1523297	2.3652249	0.005161874	0.129991359	up	yes	NA	NA
Pavir.9NG722300.v4	NA	NA	0	4.079366	26.540068	2.559758	3.383865	5.1516447	2.3650331	0.017597884	0.243207467	up	yes	NA	NA
Pavir.9NG568300.v4	GO:000367	00230(Puri	nuclear tran	3.196798	18.451738	1.244174	2.968858	5.1384694	2.3613387	0.00926993	0.175807947	up	yes	Nuclear tra	NFYC2
Pavir.9NG280100.v4	GO:000367	00561(Glyc	nac domain-	1.365534	15.729018	1.388115	1.939906	5.1365517	2.3608002	0.019172751	0.253654149	up	yes	NAC domai	NAC002
Pavir.7NG191800.v4	GO:000395	00592(alph	12-oxophyt	27.225372	13.644389	1.191129	6.7657431	5.1364104	2.3607605	0.008325836	0.165668823	up	yes	Putative 12-	OPR11;OPR11
Pavir.6KG296500.v4	NA	NA	0	1.229112	4.822345	0.416152	0.763347	5.1305317	2.3591083	0.017351054	0.24174101	up	yes	Zinc finger	ZFP7
Pavir.2KG261000.v4	GO:000406	NA	arylformami	6.3520433	1.9177755	0.222656	1.38962	5.1292823	2.358757	0.035637135	0.355170843	up	yes	Cyclase-like	CYL4;CYL4
Pavir.5NG265100.v4	GO:000573	00073(Cuti	omega-hydr	2.346522	1.756019	0.083098	0.717788	5.1225031	2.3568489	0.033559409	0.343522115	up	yes	Omega-hyd	HHT1
Pavir.4NG024900.v4	GO:000367	03018(RNA	no apical m	13.578835	14.112636	0.369846	5.039079	5.1195886	2.3560279	0.018592311	0.248940219	up	yes	NAC domai	NAC043
Pavir.5KG104200.v4	GO:000940	04141(Prot	small heat-s	15.939679	145.44327	9.877963	21.697327	5.1110519	2.3536203	0.017978883	0.244919876	up	yes	17.9 kDa h	HSP17.9B
Pavir.7KG388400.v4	GO:000969	NA	nucleoporin	18.680861	15.454506	0.45511	6.227511	5.1080806	2.3527813	0.030980597	0.329353436	up	yes	NA	NA
Pavir.7KG266900.v4	GO:000419	NA	legumain (l	5.200638	29.297661	3.298079	3.461758	5.1034217	2.3514649	0.005597742	0.134909534	up	yes	Vacuolar-p	VPE1
Pavir.7NG442800.v4	GO:000367	01200(Carb	tetraspanin-	1.580524	6.908715	0.296097	1.367609	5.1026077	2.3512347	0.029366667	0.320064917	up	yes	Tetraspanin	TOM2AH3
Pavir.7NG087800.v4	GO:000013	NA	uncharacteri	10.906448	25.58873	4.467254	2.686151	5.1017911	2.3510038	0.002157382	0.075474229	up	yes	Protein RE	RTE1
Pavir.5KG370500.v4	GO:000370	00561(Glyc	no apical m	6.150298	7.0246837	0.507436	2.0752471	5.1012771	2.3508585	0.006416571	0.145641094	up	yes	NAC domai	NAC010;NAC010;NAC010;NAC010;NAC010
Pavir.2NG083900.v4	NA	NA	0	27.776224	64.115486	2.948041	15.07427	5.0987751	2.3501507	0.008330196	0.165668823	up	yes	NA	NA
Pavir.7KG387000.v4	GO:000367	NA	protein of u	8.451915	22.056341	1.847345	4.138151	5.0970306	2.349657	0.004761782	0.123730597	up	yes	Uncharacter	At5g50100
Pavir.6KG365200.v4	GO:000563	04141(Prot	dnaj homolo	1.764511	12.535251	1.438851	1.371238	5.0887221	2.3473034	0.013198926	0.208853297	up	yes	NA	NA
Pavir.1KG222800.v4	GO:000419	00904(Dite	aspartyl pro	1.455176	4.109688	0.115091	0.980713	5.0783388	2.3443567	0.036187707	0.357604251	up	yes	Aspartic pro	At1g65240
Pavir.5NG438500.v4	GO:000013	NA	transmembr	2.733414	10.744785	1.159675	1.494709	5.0777126	2.3441787	0.011517606	0.194757431	up	yes	Protein RE	RTE1
Pavir.2NG430500.v4	GO:000576	04144(Endo	ras-related p	2.053524	3.700268	0.136415	1.000821	5.0594529	2.3389814	0.031151625	0.330075502	up	yes	Ras-related	RABA3
Pavir.9KG358200.v4	NA	NA	0	27.05633	22.786684	1.269876	8.583737	5.0583491	2.3386666	0.013791622	0.213511591	up	yes	NA	NA
Pavir.9NG074500.v4	GO:000573	01200(Carb	malate and l	1.580608	20.014482	1.855338	2.41612	5.0556719	2.3379028	0.018938146	0.25134686	up	yes	Malate dehy	Os12g0632700
Pavir.7KG346100.v4	GO:000815	NA	ras suppress	3.268782	11.032441	0.185698	2.645224	5.0517898	2.3367946	0.02919931	0.319204446	up	yes	Plant intrac	IRL1;IRL1
Pavir.6KG173300.v4	GO:000552	00360(Phen	4-coumarate	4.809377	1.031769	0.258955	0.898271	5.0475413	2.3355808	0.026094304	0.300472351	up	yes	Probable 4-	4CL1
Pavir.7KG220400.v4	GO:000098	NA	b-box zinc f	46.962475	110.66365	9.538973	21.689333	5.0475401	2.3355805	0.006092927	0.141489204	up	yes	B-box zinc	BBX24
Pavir.2KG103500.v4	GO:000367	NA	microsphere	2.5160405	1.6891997	0.4066751	0.4274418	5.0415475	2.3338666	0.007148526	0.155165561	up	yes	NA	NA
Pavir.1KG034500.v4	NA	NA	0	1.372969	1.358703	0.329371	0.212679	5.0395203	2.3332864	0.01695708	0.239792389	up	yes	NA	NA
Pavir.3KG053400.v4	GO:000333	NA	amino acid t	1.112654	4.613291	0.183217	0.953104	5.0390207	2.3331434	0.031303905	0.331071534	up	yes	Amino acid	AAP3
Pavir.5NG106200.v4	GO:000455	NA	glycosyl hyc	4.213225	2.109689	0.069526	1.18531	5.0388369	2.3330908	0.047237327	0.40946722	up	yes	Endo-1,4-b	XYN1
Pavir.8NG077700.v4	NA	NA	0	1.696104	4.32356	0.087058	1.110947	5.0247403	2.329049	0.041561786	0.383780045	up	yes	NA	NA
Pavir.3KG323500.v4	GO:000472	NA	calcineurin l	10.06667	19.849923	1.009896	4.9486317	5.0208029	2.3279181	0.007913633	0.162028857	up	yes	Calcineurin	CBL4;CBL4;CBL4;CBL4;CBL4
Pavir.6NG298400.v4	GO:000563	NA	oxysterol-bi	1.1341123	2.3565663	0.4287257	0.2686785	5.0052445	2.3234405	0.013931983	0.214437719	up	yes	Oxysterol-b	ORP4C;ORP4C;ORP4C
Pavir.7KG044400.v4	GO:000573	01200(Carb	malate and l	1.03308	2.651571	0.119799	0.617145	4.9999064	2.3219011	0.037102205	0.362937039	up	yes	Malate dehy	Os12g0632700
Pavir.9NG754600.v4	GO:000950	NA	expansin-lik	4.313088	12.984958	1.358434	2.103186	4.9970956	2.3210898	0.004348428	0.118204918	up	yes	Expansin-li	EXLA1
Pavir.3NG185200.v4	NA	NA	0	1.498259	5.409528	0.324019	1.06034	4.9898812	2.3190055	0.032129488	0.335613359	up	yes	NA	NA
Pavir.4NG109400.v4	GO:000473	01200(Carb	pyruvate del	4.063431	9.81397	1.2977	1.486017	4.9852054	2.3176529	0.005071847	0.128412736	up	yes	Pyruvate de	Os06g0246500
Pavir.3KG029200.v4	GO:000487	NA	late embryo	187.13615	325.31403	6.346688	96.469048	4.984161	2.3173507	0.031818208	0.333940095	up	yes	NDR1/HIN	NHL1
Pavir.4KG402300.v4	GO:000467	00260(Glyc	protein kina	0.998073	2.634712	0.100502	0.628859	4.9807777	2.316371	0.033761767	0.34396173	up	yes	G-type lecti	At2g19130
Pavir.2KG162000.v4	GO:000166	00561(Glyc	no apical m	4.006388	5.160303	1.036447	0.804903	4.9782448	2.3156372	0.00754248	0.157919056	up	yes	NAC domai	NAC067
Pavir.1NG186900.v4	GO:000563	00100(Ster	reticulon//su	18.137495	23.570514	2.0038992	6.3784315	4.9757055	2.3149011	0.005087365	0.128459391	up	yes	Reticulon-li	RTNLB2;RTNLB2
Pavir.7NG349800.v4	GO:000377	04144(Endo	actin-related	20.972256	42.708347	1.575569	11.244615	4.9672144	2.312437	0.016977834	0.239905287	up	yes	Actin-relate	ARPC2B
Pavir.3KG332300.v4	GO:000367	NA	late embryo	1.349241	1.185038	0.316889	0.193457	4.9658056	2.3120278	0.021253055	0.268236832	up	yes	NA	NA
Pavir.1NG172300.v4	GO:000467	04075(Plan	cgmp-deper	11.57164	17.736517	1.0985195	4.8036818	4.9656315	2.3119772	0.009252438	0.175576689	up	yes	Protein pho	At2g20050/At2g20040;At2g20050/At2g20040
Pavir.8NG303500.v4	GO:000367	NA	kelch motif	1.140949	2.200319	0.40825	0.264795	4.9644051	2.3116208	0.023914667	0.285564774	up	yes	Nitrile-spec	NSP2
Pavir.7NG341400.v4	GO:003152	00902(Mon	(+)-neomen	2.057316	9.9188712	0.997552	1.4150385	4.9640364	2.3115137	0.014292768	0.217682272	up	yes	Short-chain	SDR2b;SDR1;SDR1
Pavir.2NG480600.v4	GO:000827	NA	ring finger c	10.08861	14.852317	1.3149	3.7183593	4.9552241	2.3089503	0.003539671	0.105052173	up	yes	NEP1-intera	NIP2;NIP2
Pavir.5KG633100.v4	GO:000367	NA	homeobox p	33.749226	124.76082	15.455266	16.558098	4.9513711	2.3078281	0.007451248	0.157697172	up	yes	BEL1-like l	BLH9
Pavir.1NG524400.v4	NA	NA	0	7.318825	8.424975	0.796145	2.386481	4.9467955	2.3064943	0.011674643	0.196001507	up	yes	NA	NA
Pavir.3KG112100.v4	GO:000557	NA	cupin (cupi	2.6093253	3.2278109	0.5171796	0.6644563	4.9398773	2.3044752	0.009001518	0.173354197	up	yes	Glutelin typ	GLUA2;GLUA2;GLUA2
Pavir.3KG320700.v4	NA	NA	cotton fibre	3.172563	3.510056	0.417413	0.935934	4.9378459	2.3038818	0.012293723	0.201181202	up	yes	Pathogen-as	A70
Pavir.1KG007600.v4	GO:000563	04075(Plan	non-specific	3.049018	5.349426	0.553018	1.148	4.9373046	2.3037237	0.009948719	0.181831401	up	yes	Salt toleran	OsI_16820
Pavir.5NG187500.v4	NA	NA	0	1.703369	3.504364	0.068113	0.986757	4.9368481	2.3035903	0.035757557	0.355562089	up	yes	NA	NA

Pavir.3KG269000.v	NA	NA	0	17.578817	31.746975	1.918425	8.081337	4.9326966	2.3023766	0.009508224	0.1784478	up	yes	NA	NA
Pavir.1NG548400.v	NA	NA	0	1.956292	13.011526	0.860872	2.183579	4.916426	2.2976099	0.027018405	0.306076323	up	yes	NA	NA
Pavir.3KG131900.v	GO:000447	01230(Bios	s-adenosylm	1122.0277	5011.4609	340.19986	907.88428	4.9143231	2.2969927	0.016189314	0.23345038	up	yes	S-adenosylm	SAM1
Pavir.1KG126300.v	GO:000484	NA	f-box domai	3.752091	3.166268	0.323733	1.085688	4.9086533	2.2953273	0.014645857	0.220258684	up	yes	F-box/kelch	At1g55270
Pavir.5KG053500.v	GO:000368	NA	transcriptio	15.68364	34.11578	1.429134	8.723697	4.9049787	2.2942469	0.011356014	0.19298034	up	yes	Transcriptio	KUA1
Pavir.5KG777600.v	GO:001614	00500(Star	x-box transc	2.500146	3.668679	0.943006	0.315808	4.9005056	2.2929306	0.009650993	0.179602151	up	yes	Peptide cha	APG3
Pavir.4NG251200.v	GO:000573	00730(Thia	thiamine thi	3.290622	33.424232	3.757062	3.743468	4.894968	2.2912994	0.013814044	0.213594684	up	yes	Thiamine th	THI1-2
Pavir.7KG260100.v	GO:000484	04141(Prot	rnf5//subfar	28.285723	153.30547	6.755256	30.380657	4.8899078	2.2898073	0.023301153	0.281238111	up	yes	E3 ubiquitin	RMA1H1
Pavir.2NG088200.v	GO:000449	00073(Cuti	abieta-7,13-	54.659935	128.30933	8.919894	28.503708	4.8891408	2.289581	0.010857292	0.188567538	up	yes	Cytochrome	CYP704C1
Pavir.6KG002000.v	GO:000487	NA	late embryo	197.77069	203.40746	2.492338	79.629463	4.8851601	2.2884058	0.048428607	0.414772751	up	yes	NDR1/HIN	NHL1
Pavir.9KG091800.v	GO:000367	03018(RNA	nac domain	18.52986	72.163185	7.851356	10.715646	4.8846359	2.288251	0.007487604	0.157697172	up	yes	NAC domai	NAC087
Pavir.2NG334300.v	GO:000467	04075(Plan	serine/threo	4.054316	4.864858	0.358694	1.467917	4.8829083	2.2877407	0.013348173	0.209368875	up	yes	CBL-intera	CIPK16
Pavir.9NG594300.v	GO:000370	NA	cct motif (co	22.828899	42.193882	1.715047	11.607993	4.8804763	2.287022	0.016109429	0.232779784	up	yes	Zinc finger	COL13
Pavir.8NG345800.v	GO:000573	00130(Ubic	mpbq/msbq	2.4806427	5.8155201	0.5902377	1.1096446	4.8804338	2.2870094	0.01525798	0.226367538	up	yes	2-methyl-6-	Os12g0615400;Os12g0615400;Os12g0615400
Pavir.9KG456800.v	GO:000636	NA	transcriptio	3.050561	11.455006	1.101517	1.87229	4.8777769	2.2862238	0.008854754	0.172158188	up	yes	Probable me	MED26B
Pavir.9KG430200.v	GO:000370	04075(Plan	ap2 domain	1.32511	2.466296	0.093413	0.683999	4.8769584	2.2859817	0.045840063	0.403360351	up	yes	Ethylene-res	RAP2-3
Pavir.5KG054000.v	GO:000387	01200(Carb	phosphofruc	2.783082	7.58454	0.928895	1.201689	4.866094	2.2827642	0.007053398	0.154349059	up	yes	ATP-depen	PFK3
Pavir.5KG696000.v	GO:000551	NA	protein iq-d	94.528148	91.82731	2.7043413	35.5993	4.8652152	2.2825036	0.029698511	0.321850257	up	yes	Protein IQ-I	IQD14;IQD14
Pavir.5KG104700.v	GO:000455	NA	glycosyl hyc	9.625071	4.501206	0.281581	2.622149	4.8648728	2.2824021	0.022635608	0.277230089	up	yes	Endo-1,4-be	XYN1
Pavir.7KG406700.v	GO:000001	NA	endonucleas	7.559867	10.534412	0.851346	2.869996	4.8622994	2.2816387	0.008579266	0.168680678	up	yes	Endonuclea	ENDO1
Pavir.9NG193000.v	GO:000467	04140(Regu	serine/threo	35.432266	108.36691	8.746819	20.844152	4.8595624	2.2808264	0.009852517	0.181198543	up	yes	CBL-intera	CIPK7
Pavir.2NG446500.v	GO:000098	NA	b-box type z	21.850005	65.188284	6.70699	11.219617	4.8552572	2.2795477	0.006495674	0.14632013	up	yes	B-box zinc	BBX19;BBX19
Pavir.2NG492400.v	GO:000827	NA	ring finger c	14.464083	17.151104	1.136869	5.37511	4.8549277	2.2794498	0.006667848	0.148330994	up	yes	NEP1-intera	NIP2
Pavir.5NG352800.v	GO:000472	NA	protein-seri	1.609909	1.840668	0.435836	0.274948	4.854607	2.2793545	0.023318532	0.281323632	up	yes	Dual specifi	DSPTP1B
Pavir.2NG391400.v	GO:000013	NA	cell cycle co	96.173203	167.41657	5.305979	49.020828	4.8519282	2.2785582	0.024594359	0.289920292	up	yes	ALA-intera	ALIS5
Pavir.9NG621700.v	NA	NA	0	1.781173	2.666633	0.2209	0.696093	4.8504252	2.2781112	0.022457345	0.276199619	up	yes	Coiled-coil	SCD2
Pavir.2KG154400.v	GO:000563	04075(Plan	member of '	1.152734	4.743377	0.722331	0.494082	4.8471292	2.2771305	0.018892779	0.251024069	up	yes	Probable ca	CXE15
Pavir.9KG310700.v	NA	NA	0	9.015632	0.96188	0.356517	1.703081	4.8443978	2.2763173	0.048764082	0.416317394	up	yes	NA	NA
Pavir.5KG389800.v	GO:000864	NA	rag1-activat	5.137093	1.9471332	0.1490821	1.3141127	4.8416151	2.2754884	0.041949435	0.385759888	up	yes	Bidirectiona	SWEET6B;SWEET6B
Pavir.5NG556000.v	NA	NA	0	14.177072	96.041855	10.385065	12.384547	4.8406151	2.2751904	0.010853414	0.188567538	up	yes	NA	NA
Pavir.8KG082700.v	GO:000419	NA	aspartyl pro	1.743822	8.3702477	1.2005667	0.8894788	4.8391625	2.2747574	0.01266199	0.204359507	up	yes	Aspartic pro	ASP1;ASP1;ASP1
Pavir.2NG638900.v	GO:000460	00940(Phen	peroxidase (	1127.5752	376.70139	21.559822	289.42847	4.8370843	2.2741377	0.039514458	0.374918366	up	yes	Peroxidase	-
Pavir.5KG421100.v	GO:000370	04626(Plan	wrky transci	2.952502	4.359643	0.071639	1.440831	4.8345719	2.2733882	0.035909583	0.356279575	up	yes	WRKY tran	WRKY71
Pavir.9KG643300.v	GO:000472	NA	protein pho	7.4458352	57.651553	2.9700964	10.543952	4.817016	2.2681397	0.024840199	0.291936444	up	yes	Probable pr	BIPP2C1;BIPP2C1
Pavir.3NG175400.v	GO:000557	NA	cupin (cupin	1.7518075	15.575745	1.3963684	2.201305	4.8163216	2.2679317	0.013781052	0.213435887	up	yes	Glutelin typ	GLUA2;GLUA2;GLUA2;GLUA2;GLUA2;GLUA2
Pavir.9KG590500.v	GO:000588	01200(Carb	eama-like tr	43.568081	29.359735	1.536494	13.621078	4.8113125	2.2664305	0.020561218	0.263211799	up	yes	WAT1-rela	At5g07050
Pavir.5NG096700.v	NA	NA	0	25.26243	28.467356	0.805635	10.365401	4.8097407	2.2659591	0.021715189	0.270884721	up	yes	NA	NA
Pavir.1KG190200.v	NA	NA	domain of u	55.088959	92.917488	5.500287	25.279015	4.8086356	2.2656276	0.011983494	0.198438006	up	yes	NA	NA
Pavir.7NG367400.v	GO:000377	03008(Ribo	kinesin fam	66.500702	59.127156	7.1921462	18.979973	4.8000643	2.2630537	0.007346859	0.156795385	up	yes	Kinesin-like	KIN14H;KIN14H
Pavir.2NG248100.v	GO:000367	00190(Oxic	myb-like dn	23.803898	17.922617	2.917051	5.784065	4.795536	2.2616921	0.002380431	0.080036845	up	yes	Transcriptio	MYB44
Pavir.8KG018200.v	NA	NA	0	3.164777	1.501989	0.175665	0.797634	4.7947917	2.2614682	0.038681939	0.370386463	up	yes	Probable se	PBL26
Pavir.5NG206100.v	GO:000588	NA	hydrophobic	18.332293	32.453751	2.209645	8.389178	4.7916683	2.260528	0.008111888	0.163512853	up	yes	Hydrophobi	LTI6B
Pavir.9NG729100.v	GO:000536	NA	sugar transp	18.938866	73.76004	2.252469	17.098625	4.7903703	2.2601372	0.029193467	0.319204446	up	yes	Sucrose tran	SUT1
Pavir.9NG756300.v	GO:000950	NA	expansin-lik	13.520732	34.052898	3.816308	6.117318	4.7891505	2.2597698	0.003289944	0.099442168	up	yes	Expansin-li	EXLA1
Pavir.1NG288700.v	NA	NA	0	11.506666	25.033958	2.265048	5.371792	4.7847832	2.2584536	0.004260993	0.116860063	up	yes	NA	NA
Pavir.7KG241100.v	GO:000842	00500(Star	glycosyl hyc	1.31458	4.161174	0.198774	0.947188	4.7783033	2.2564984	0.029906534	0.32320922	up	yes	Probable in	BGLU14
Pavir.2KG342900.v	NA	NA	0	5.900263	1.990863	0.520177	1.131382	4.7779861	2.2564027	0.01955071	0.256267806	up	yes	NA	NA
Pavir.8KG077400.v	GO:000367	00190(Oxic	myb transcr	31.813608	60.987778	3.26568	16.168814	4.7750863	2.2555268	0.013633062	0.212114502	up	yes	Transcriptio	MYB4
Pavir.1NG411700.v	NA	NA	0	2.269452	3.468443	0.218973	0.982684	4.7749857	2.2554964	0.044288123	0.397524521	up	yes	NA	NA
Pavir.8NG152700.v	GO:000521	NA	protein nrt1	9.7410795	47.76614	4.2309864	7.8252692	4.7699071	2.2539612	0.012674871	0.204391821	up	yes	Protein NR	NPF4.4;NPF4.4;NPF4.4
Pavir.1NG109400.v	GO:000484	NA	f-box domai	1.694695	1.446143	0.158519	0.500116	4.768708	2.2535984	0.02747566	0.308622709	up	yes	F-box/kelch	At1g55270
Pavir.7NG361900.v	GO:000404	01200(Carb	aminomethy	99.750245	227.39031	12.039122	56.577647	4.7676473	2.2532775	0.016625451	0.236796082	up	yes	Aminometh	GDCST;GDCST
Pavir.8KG182100.v	GO:000467	04075(Plan	serine/threo	19.937027	113.79306	8.115427	19.949781	4.7649776	2.2524694	0.012316186	0.201385036	up	yes	Serine/thre	SAPK4
Pavir.9NG235900.v	NA	NA	0	5.279484	6.775546	0.328489	2.202606	4.7627726	2.2518017	0.029584561	0.321266102	up	yes	NA	NA
Pavir.5KG509000.v	GO:000367	NA	late embryo	6.669975	27.044462	2.597264	4.485145	4.7603064	2.2510544	0.011411815	0.193578392	up	yes	Late embryo	LEA14-A
Pavir.6KG154600.v	GO:000387	00051(Fruc	phosphofruc	62.898769	134.2745	8.8916251	32.560422	4.7566593	2.2499487	0.013072842	0.207949249	up	yes	Pyrophosph	PFP-ALPHA;PFP-ALPHA;PFP-ALPHA
Pavir.4KG170400.v	GO:000419	NA	aspartyl pro	139.12661	145.73195	2.177866	57.729558	4.7549793	2.2494391	0.048674301	0.416023543	up	yes	Aspartyl pro	APCB1;APCB1
Pavir.2NG456400.v	NA	NA	0	4.576612	31.124226	2.29583	5.213514	4.7541886	2.2491992	0.014320248	0.217778448	up	yes	Uncharacter	At1g76070
Pavir.2NG617400.v	GO:000950	04070(Phos	ovarian can	6.9784887	12.649389	0.9222163	3.2102428	4.7496846	2.2478317	0.009726869	0.180068207	up	yes	Rhodanese-	STR6;STR6;STR6
Pavir.2NG582200.v	GO:000370	NA	ccaat-bindir	1.640811	6.790314	0.994176	0.781103	4.7491831	2.2476794	0.019052074	0.252502137	up	yes	Nuclear tra	NFYB10
Pavir.7KG249500.v	GO:000367	NA	caax proteas	7.661412	26.677799	3.000202	4.23288	4.7475213	2.2471745	0.004931855	0.126834076	up	yes	NA	NA
Pavir.3NG158300.v	GO:000695	03013(RNA	protein lurp	1.027465	4.570243	0.328335	0.851321	4.7452037	2.24647	0.034592332	0.349579798	up	yes	Protein LUI	At2g38640
Pavir.1KG030400.v	GO:000007	01200(Carb	cyclin, n-ter	46.14164	38.408058	0.805362	17.019745	4.7432926	2.2458889	0.0397876	0.376274732	up	yes	Cyclin-P4-1	CYCP4-1;CYCP4-1
Pavir.9NG459300.v	GO:000467	04075(Plan	serine/threo	14.928978	12.390673	0.714241	5.058911	4.7321898	2.2425079	0.014457219	0.218944411	up	yes	Serine/thre	SAPK3
Pavir.9NG741400.v	GO:000464	01200(Carb	phosphoseri	47.68222	128.33247	15.15416	22.076317	4.7277045	2.2411399	0.007782656	0.160731145	up	yes	Phosphoser	PSAT1
Pavir.2NG532200.v	GO:000367	NA	protein of u	14.857359	35.757339	1.919673	8.808214	4.7180491	2.2381904	0.013306771	0.209366345	up	yes	NA	NA
Pavir.6KG245700.v	NA	NA	0	6.068981	1.701634	0.650292	0.997184	4.7166787	2.2377713	0.022384388	0.275753638	up	yes	NA	NA
Pavir.2NG032100.v	GO:000333	NA	amino acid	2.427029	22.788332	3.631759	1.71535	4.7156998	2.2374719	0.016023328	0.232455648	up	yes	Amino acid	AAP6
Pavir.5KG228700.v	GO:000078	NA	telomeric re	10.706214	14.17452	2.782297	2.513851	4.6978925	2.2320137	0.009237234	0.175376749	up	yes	NA	NA
Pavir.7KG386500.v	NA	NA	f12f1.11-rel	10.946512	21.806349	3.30665	3.67642	4.690324	2.2296876	0.002248123	0.077392148	up	yes	NA	NA
Pavir.9NG799200.v	GO:000370	04075(Plan	ap2 domain	33.952358	57.807892	5.272126	14.313904	4.6849847	2.2280443	0.004468147	0.119744441	up	yes	Ethylene-res	RAP2-2
Pavir.6NG351100.v	NA	NA	0	119.4428	200.01755	9.807688	58.389011	4.6843961	2.2278631	0.019985836	0.258583965	up	yes	NA	NA
Pavir.7KG134400.v	GO:000382	NA	snf1-related	8.6912348	57.987806	3.7734693	10.463475	4.6835218	2.2275938	0.02206139	0.273820243	up	yes	SNF1-relate	KING1;KING1
Pavir.5KG488900.v	GO:000367	03040(Splic	protein of u	2.577316	11.393252	0.309181	2.676532	4.6791396	2.2262433	0.044564419	0.398414767	up	yes	NA	NA
Pavir.9KG598300.v	GO:000588	NA	sulfate trans	7.543932	16.289501	1.689948	3.407053	4.6759718	2.2252662	0.003994011	0.112241571	up	yes	Sulfate tran	SULTR3;1
Pavir.1KG203300.v	GO:000367	NA	late embryo	44.199982	15.924313	2.770689	10.092395	4.6741742	2.2247115	0.009104697	0.17453612	up	yes	NA	NA
Pavir.5NG555900.v	GO:000372	NA	60s riboson	3.120374	11.654304	1.167861	1.995414	4.6706903	2.2236358	0.007112448	0.154919423	up	yes	Uncharacter	At5g19025
Pavir.5KG127700.v	GO:000557	00514(Othe	glycosyltran	9.362297	2.31305	0.451486	2.052457	4.6627847	2.2211918	0.022128367	0.274218049	up	yes	Beta-1,2-xy	XYXT1
Pavir.3KG029300.v	GO:000014	NA	exocyst con	0.992534	5.570224	0.394456	1.017295	4.6486654	2.2168166	0.03262261	0.338561609	up	yes	Exocyst con	EXO70A1
Pavir.8NG289500.v	NA	NA	0	18.477801	20.030975	7.890451	0.39394	4.6483533	2.2167197	0.032191247	0.335901453	up	yes	NA	NA
Pavir.8NG137300.v	NA	NA	leucine-rich	7.627285	8.292008	0.629525	2.799435	4.642601	2.2149333	0.015780689	0.230621484	up	yes	NA	NA
Pavir.4KG394100.v	GO:000367	NA	brisc and br	7.675415	2.671391	1.076516	1.153672	4.6394322	2.2139482	0.015381319	0.22717071	up	yes	NA	NA

Pavir.4KG001400.v	GO:000367	01200(Cart	gluconokina	3.609325	17.045238	1.425265	3.026697	4.6394293	2.2139474	0.01954546	0.256267806	up	yes	NA	NA
Pavir.7KG213800.v	GO:000104	NA	helix-loop-h	8.6585551	9.386205	2.5959116	1.2953844	4.6372109	2.2132573	0.002617661	0.085302158	up	yes	Transcriptio	BHLH128;BHLH128;BHLH128
Pavir.2KG395300.v	GO:000367	NA	protein nlp1	2.0063423	6.4893948	0.4963998	1.3372525	4.6332324	2.212019	0.012374563	0.202064075	up	yes	Protein NLI	NLP1;NLP1;NLP1;NLP1;NLP1;NLP1;NLP1
Pavir.7KG391800.v	GO:000404	01200(Cart	aminomethy	190.78734	317.46494	16.752234	92.957092	4.6327171	2.2118586	0.019588714	0.256267806	up	yes	Aminometh	GDCST
Pavir.6NG236900.v	GO:000551	04626(Plan	non-specific	4.125905	1.782399	0.139716	1.135909	4.6316935	2.2115398	0.046060582	0.404532086	up	yes	Serine/threo	RIPK
Pavir.1KG108000.v	NA	NA	0	2.524244	6.722494	1.477899	0.521018	4.6258739	2.2097259	0.019599034	0.256267806	up	yes	NA	NA
Pavir.7KG120400.v	NA	NA	0	77.18235	133.24352	13.250966	32.30764	4.6187951	2.2075165	0.00828286	0.165186788	up	yes	NA	NA
Pavir.6KG059700.v	GO:000382	00053(Asco	gdp-d-mann	13.166457	15.488811	1.792133	4.412009	4.6187318	2.2074968	0.003929206	0.111168708	up	yes	GDP-manno	GME-1
Pavir.5KG526500.v	GO:000577	NA	glutamate ca	6.707339	3.3177244	0.2544489	1.9213504	4.6075312	2.2039939	0.023254673	0.281238111	up	yes	Probable gl	LAMP1;LAMP1;LAMP1;LAMP1
Pavir.7NG191300.v	GO:000395	00592(alph	nadh oxidor	19.497608	18.782032	1.970634	6.338991	4.6066628	2.203722	0.005584679	0.134904319	up	yes	Putative 12-	OPR13
Pavir.5NG036700.v	GO:000563	NA	zinc finger c	4.501152	46.209545	2.967041	8.074869	4.5925657	2.1993004	0.024233544	0.287323118	up	yes	Zinc finger	Os01g0192000
Pavir.1NG009300.v	GO:001014	00561(Glyc	glycerol-3-p	5.4244416	2.777236	0.614623	1.1717097	4.5913494	2.1989182	0.009332151	0.176487135	up	yes	Glycerol-3-p	GPAT6;GPAT6
Pavir.5NG508400.v	NA	NA	0	3.117607	12.353095	2.008535	1.362958	4.5886799	2.1980792	0.032274473	0.336473026	up	yes	NA	NA
Pavir.4KG170000.v	GO:000588	NA	osmotic stre	3.640519	8.8955849	0.315085	2.420156	4.5831807	2.1963492	0.021937514	0.272789512	up	yes	Potassium t	HAK25;HAK25
Pavir.5KG111600.v	NA	NA	0	8.391532	88.104752	10.571257	10.499247	4.5796856	2.1952486	0.017984562	0.244919876	up	yes	NA	NA
Pavir.8NG180000.v	GO:000551	04626(Plan	leucine rich	1.476102	1.031082	0.129912	0.417984	4.5760217	2.1940939	0.024256963	0.28738543	up	yes	Receptor ki	XA21
Pavir.7NG416700.v	GO:000098	04075(Plan	auxin respo	1.100225	2.18035	0.292955	0.425439	4.5665401	2.1911015	0.02215802	0.274404512	up	yes	Auxin respo	ARF13
Pavir.3KG125800.v	NA	NA	duf761-asso	1.341046	2.135822	0.434357	0.32833	4.5587089	2.1886253	0.022924013	0.279744358	up	yes	NA	NA
Pavir.5KG375700.v	GO:000367	04141(Prot	gem-like pro	2.249434	12.825063	1.371298	1.943799	4.5472265	2.1849869	0.016568051	0.236606372	up	yes	GEM-like p	FIP1
Pavir.8NG023000.v	GO:000441	00650(Buta	hydroxymet	29.622843	16.132304	0.937641	9.1246716	4.54718	2.1849721	0.024049701	0.286371335	up	yes	Hydroxyme	HMGCL;HMGCL
Pavir.3KG265700.v	GO:000521	NA	protein nrt1.	1.540517	2.116277	0.065716	0.738654	4.5461591	2.1846482	0.046172976	0.404762788	up	yes	Protein NR	NPF2.7
Pavir.7NG357200.v	GO:000367	04075(Plan	b3 dna bind	13.749964	14.657282	1.963347	4.287611	4.5444628	2.1841098	0.003365769	0.10108875	up	yes	Auxin respo	ARF10
Pavir.4NG286200.v	GO:000551	NA	ricin-type be	3.134302	54.59375	6.970655	5.734121	4.5438071	2.1839016	0.027652368	0.309361931	up	yes	Ricin B-like	R40G2
Pavir.7KG182700.v	GO:000467	04712(Circ	cryptochron	8.7538345	37.291141	3.7244177	6.419981	4.5389556	2.1823604	0.012205506	0.200387495	up	yes	Cryptochro	CRY1;CRY1;CRY1
Pavir.6KG340200.v	GO:000563	00360(Pher	caffeoyl-coa	539.45618	803.12213	62.568989	233.32539	4.5373565	2.181852	0.015127865	0.225341714	up	yes	Tricin synth	ROMT-15
Pavir.9NG381900.v	NA	NA	0	5.336514	9.847883	0.968572	2.378609	4.5364732	2.1815711	0.005706377	0.136102468	up	yes	IQ domain-	IQM2;IQM2
Pavir.1KG459200.v	GO:000367	00190(Oxic	myb-like dn	3.603333	11.395781	0.916213	2.391912	4.5340227	2.1807916	0.011298775	0.192482559	up	yes	Myb-relatec	-
Pavir.9NG743800.v	GO:000587	04144(Endo	armadillo re	8.6271249	12.256813	1.0599515	3.5517199	4.5284965	2.1790321	0.00631525	0.144254761	up	yes	Kinesin-like	KINUA;KINUA;KINUA;KINUA;KINUA
Pavir.5KG350300.v	GO:000367	NA	dvl family (	57.784439	9.991859	8.224042	6.749923	4.526276	2.1783246	0.010776143	0.188149509	up	yes	NA	NA
Pavir.2NG403700.v	GO:004316	NA	ring finger c	3.01122	3.620361	1.2234226	0.2431487	4.5218266	2.1769057	0.013542554	0.211239519	up	yes	RING-H2 f	ATL54;ATL54;ATL54
Pavir.1NG189800.v	GO:000562	04120(Ubic	enth/vhs/gat	1.2172152	4.568658	0.2539183	1.0258865	4.5209029	2.1766109	0.028387097	0.313057933	up	yes	TOM1-like	TOL9;TOL9
Pavir.6NG331900.v	GO:000370	01200(Cart	no apical m	11.185232	79.841678	5.2187015	14.916078	4.5208795	2.1766035	0.027519053	0.308757967	up	yes	NAC domai	NAC018;NAC018;NAC018
Pavir.2KG384000.v	GO:000950	NA	major facilit	1.856379	10.856591	1.329674	1.489002	4.510263	2.1732116	0.013631189	0.212114502	up	yes	Protein NU	NFD4
Pavir.7NG444900.v	GO:000598	00500(Starc	alpha-amyla	3.989877	4.580102	0.533279	1.367453	4.5087782	2.1727365	0.013270799	0.209130801	up	yes	Alpha-amyl	AMY1.1
Pavir.5NG620700.v	GO:000480	00564(Glyc	cgi-141-rela	147.32973	279.05601	18.21463	76.395974	4.5067437	2.1720854	0.017771807	0.24402394	up	yes	Phospholipa	Os05g0574000;Os05g0574000
Pavir.4NG286000.v	GO:000013	NA	polysacchar	33.192554	31.682953	3.98273	10.414901	4.5059848	2.1718425	0.004323935	0.117726974	up	yes	Protein IRR	IRX15
Pavir.9KG117600.v	GO:000981	00942(Anth	anthocyanid	7.021282	12.603933	0.905848	3.453593	4.5017733	2.1704934	0.009709493	0.180066525	up	yes	Anthocyanic	RT
Pavir.8KG107200.v	GO:000588	04626(Plan	respiratory b	1.57152	5.449861	0.33741	1.223115	4.499371	2.1697233	0.018669396	0.249720619	up	yes	Respiratory	RBOHC
Pavir.3KG252200.v	NA	NA	0	1.796613	6.936468	1.046169	0.898154	4.4915793	2.1672228	0.024408688	0.288670954	up	yes	NA	NA
Pavir.7KG391600.v	GO:000577	01200(Cart	s -2-hydroxy	4.682933	16.487453	1.546968	3.169165	4.488929	2.1663713	0.01005221	0.181875162	up	yes	Peroxisoma	GLO2
Pavir.7NG314100.v	GO:000367	NA	zinc-finger c	14.118638	37.820782	5.939048	5.639842	4.4856994	2.1653329	0.012230536	0.200571016	up	yes	FCS-Like Z	FLZ2
Pavir.5NG199700.v	GO:000471	04075(Plan	serine-threo	24.176718	25.274832	1.860536	9.163832	4.4856585	2.1653198	0.012913136	0.20681383	up	yes	Serine/threo	HT1
Pavir.5KG433400.v	NA	NA	0	3.565343	3.009334	0.512091	0.953706	4.485394	2.1652347	0.009884863	0.181456243	up	yes	NA	NA
Pavir.2NG280200.v	GO:000418	00940(Pher	serine prote	2.305889	6.861938	0.890926	1.153115	4.4851483	2.1651557	0.015360253	0.227038069	up	yes	Serine carbo	SCPL18
Pavir.5KG230300.v	GO:000097	NA	gata transcr	18.537758	37.71255	1.93488	10.61267	4.4829712	2.1644552	0.013687305	0.212470074	up	yes	GATA trans	GATA23;NA
Pavir.5KG141100.v	NA	NA	0	18.829687	4.3215173	0.3176017	4.8520137	4.4783223	2.1629584	0.046659292	0.406501837	up	yes	NA	NA
Pavir.9KG064200.v	GO:000367	NA	zinc-finger c	27.849056	65.192024	9.753716	11.040002	4.4744802	2.1617201	0.006428833	0.145687913	up	yes	FCS-Like Z	FLZ8
Pavir.7NG389500.v	GO:000449	00904(Dite	protein reve	16.52198	17.378311	2.5943401	4.9820952	4.4744381	2.1617065	0.004038257	0.112893911	up	yes	Protein RE	RVE2;RVE2
Pavir.9NG728200.v	NA	NA	0	1.676723	23.764393	2.0153	3.675663	4.4704413	2.1604173	0.037308958	0.363634109	up	yes	NA	NA
Pavir.3KG519200.v	GO:000467	04626(Plan	tyrosine kin	1.3875027	1.138126	0.2075667	0.3578413	4.4669136	2.1592783	0.025334589	0.295161708	up	yes	Cysteine-ric	CRK19;CRK19;CRK19;CRK19;CRK19
Pavir.7NG339100.v	GO:000467	00500(Starc	legume lecti	1.602602	1.723354	0.261875	0.485078	4.4526978	2.1546797	0.014269111	0.217410101	up	yes	L-type lecti	SIT2
Pavir.9NG215400.v	GO:000582	NA	[protein-pii]	24.413635	72.798744	4.902889	16.939756	4.4505773	2.1539925	0.017024588	0.240294829	up	yes	ACT domai	ACR4
Pavir.3KG298700.v	GO:000467	04626(Plan	lrr receptor-	2.160038	4.640733	0.643341	0.88801	4.4410269	2.1508933	0.012579344	0.203919969	up	yes	Protein MA	MDIS2
Pavir.3KG130700.v	GO:000460	00940(Pher	peroxidase (	139.75024	38.685028	3.799626	36.38702	4.4401633	2.1506127	0.041551006	0.383774816	up	yes	Peroxidase	PER2
Pavir.5NG587000.v	GO:000573	NA	cytochrome	1.106152	3.727791	0.606917	0.481946	4.439441	2.150378	0.030216043	0.324837592	up	yes	NA	NA
Pavir.9KG453800.v	GO:000369	NA	cgi-12 prote	5.02269	27.014778	2.891697	4.327902	4.4375689	2.1497695	0.011680988	0.196020441	up	yes	NA	NA
Pavir.5KG585300.v	GO:000367	NA	ccaat/enhan	2.276971	9.680148	0.960828	1.736054	4.4336827	2.1485055	0.017065406	0.240475103	up	yes	Basic leucir	BZIP24
Pavir.1KG509200.v	GO:001584	NA	aquaporin tr	2.7024011	12.456542	1.3991109	2.0232619	4.4293666	2.1471004	0.013912005	0.21440351	up	yes	Aquaporin	NIP2-1;NIP2-1
Pavir.2NG389300.v	GO:000016	03040(Splic	u2 snrnp au	36.784245	113.57108	14.683646	19.267952	4.4285197	2.1468245	0.009596712	0.178946539	up	yes	Splicing fac	U2AF35A
Pavir.7KG157700.v	GO:000425	04144(Endo	proprotein c	2.513839	3.154149	0.23089	1.049011	4.4284581	2.1468045	0.024552855	0.28973757	up	yes	Subtilisin-li	SBT1.5
Pavir.9KG334700.v	GO:000367	00750(Vita	hd-zip prote	4.520088	7.645121	1.644431	1.104621	4.4252379	2.145755	0.007528226	0.15784489	up	yes	Homeobox-	HAT4
Pavir.1NG007100.v	GO:000557	00860(Porp	zinc finger f	4.431603	1.657306	0.468602	0.907886	4.4235104	2.1451917	0.033716911	0.343943783	up	yes	GDSL ester	At5g45950
Pavir.7NG338100.v	GO:000484	04141(Prot	rnf5//subfar	18.500132	119.2529	11.008389	20.190161	4.4153665	2.1425332	0.021460996	0.26972793	up	yes	E3 ubiquiti	RMA1H1
Pavir.6KG397800.v	GO:000563	NA	heat shock t	3.804983	6.137884	0.512974	1.73912	4.4149432	2.1423949	0.015977525	0.232267769	up	yes	Heat stress	HSFB2B
Pavir.5KG044100.v	GO:000098	NA	b-box zinc f	2.618323	15.658872	1.595922	2.54412	4.4147366	2.1423274	0.018588688	0.248940219	up	yes	B-box zinc	BBX22;BBX22
Pavir.1KG356000.v	GO:000646	04626(Plan	serine/threo	3.974929	11.919247	1.09537	2.507912	4.4110275	2.1411148	0.010906364	0.188905631	up	yes	Serine/threo	NEK6
Pavir.6NG325000.v	GO:000022	00190(Oxic	65-kda micr	10.536544	51.434856	2.223447	11.825942	4.4109676	2.1410952	0.031234426	0.330490308	up	yes	65-kDa mic	MAP65-8
Pavir.9KG200600.v	GO:000573	NA	copper trans	11.53721	22.500925	2.449595	5.283257	4.4017569	2.1380795	0.007519805	0.157756361	up	yes	Heavy meta	HIPP03
Pavir.5KG765000.v	GO:000973	04075(Plan	response reg	1.031316	12.115129	1.455903	1.531582	4.4005058	2.1376694	0.032436216	0.337482178	up	yes	Two-compo	RR4
Pavir.2NG138600.v	GO:000550	NA	ubiquinol-cy	3.824852	4.885724	0.872062	1.107498	4.4002586	2.1375883	0.026646604	0.30386302	up	yes	Putative lac	LAC9
Pavir.1NG273900.v	GO:000573	NA	diphosphoir	1.295333	6.781706	1.035576	0.801852	4.3958397	2.1361388	0.028698377	0.315011423	up	yes	Nudix hydro	NUDT12
Pavir.9KG011100.v	NA	NA	mkiaa1688	14.586365	4.874361	0.64488	3.786901	4.391175	2.134607	0.029575452	0.321260033	up	yes	NA	NA
Pavir.3NG226100.v	NA	NA	0	20.414352	84.645256	11.819669	12.113626	4.3896842	2.1341172	0.00871035	0.170544017	up	yes	NA	NA
Pavir.2KG591200.v	NA	NA	0	4.104821	4.009533	0.905357	0.943434	4.3890056	2.1338941	0.022215292	0.27450782	up	yes	NA	NA
Pavir.4KG303500.v	NA	NA	0	4.393273	4.687053	0.622775	1.451418	4.3777633	2.130194	0.038779975	0.371136017	up	yes	NA	NA
Pavir.2KG198700.v	GO:000333	NA	amino acid t	2.275815	3.177653	0.197203	1.049151	4.375537	2.1294601	0.0413516	0.3826169	up	yes	Amino acid	AAP6
Pavir.4KG395800.v	GO:001678	NA	alpha/beta-h	8.760738	20.010933	1.687164	4.890204	4.3743441	2.1290667	0.009611787	0.17904992	up	yes	Probable ly	BDG3
Pavir.9NG790100.v	GO:000367	NA	stress respo	11.18228	29.554808	1.2371685	8.0763327	4.3739821	2.1289473	0.020728044	0.264543071	up	yes	NA	NA
Pavir.7KG110100.v	GO:000375	03030(DNA	fk506-bindi	14.113475	7.769891	0.892037	4.125866	4.361058	2.1246782	0.012055242	0.199099616	up	yes	70 kDa pep	FKBP70
Pavir.2NG043600.v	GO:000905	NA	glutaredoxir	3.297609	5.932907	0.491776	1.625599	4.3594148	2.1241345	0.021079373	0.267176726	up	yes	Uncharacter	At3g28850
Pavir.9KG607600.v	GO:000367	04712(Circ	dof domain,	8.2158109	41.396542	2.8206535	8.5656543	4.3571941	2.1233994	0.017889994	0.244744653	up	yes	Cyclic dof f	CDF1;CDF1
Pavir.4KG402000.v	NA	NA	wound-indu	22.020285	19.723631	5.146803	4.440199	4.35422	2.1224143	0.011514448	0.194757431	up	yes	NA	NA

Pavir.5NG612000.v	GO:000013	00790(Fola	exostosin h	413.47894	479.07294	20.576345	184.49472	4.3524028	2.1218121	0.034074318	0.345646428	up	yes	Probable gl	Os01g0926700
Pavir.1NG022600.v	GO:000367	00511(Othe	protein of u	5.9817776	26.938622	2.1130364	5.4539969	4.3505028	2.1211821	0.013876027	0.214288516	up	yes	NA	NA
Pavir.5KG682800.v	GO:000467	00260(Glyc	protein kina	5.357937	5.02537	0.535129	1.852361	4.3490473	2.1206994	0.010565748	0.186553968	up	yes	G-type lecti	At2g19130
Pavir.6KG068500.v	GO:000682	NA	mfs transpo	2.017949	10.608742	1.310215	1.593924	4.347826	2.1202942	0.015811388	0.230800811	up	yes	Putative gly	At3g47420
Pavir.1NG240900.v	GO:000367	00520(Ami	mac/perfori	8.1519246	3.7545711	0.4269401	2.3116238	4.3477151	2.1202574	0.020092246	0.259513906	up	yes	MACPF do	At1g14780;At1g14780
Pavir.1KG184900.v	GO:000367	04626(Plan	erebp-like fa	4.055048	3.192788	0.207787	1.460633	4.3441316	2.1190678	0.044611547	0.398414767	up	yes	Dehydration	DREB3
Pavir.1NG503200.v	GO:000550	NA	calcineurin l	1.428145	1.700524	0.34454	0.375696	4.3439498	2.1190074	0.030318033	0.325339676	up	yes	Calcineurin	CBL9
Pavir.4NG243000.v	GO:000333	NA	amino acid t	39.367912	157.41766	17.354277	28.027863	4.3361899	2.1164279	0.015920669	0.231765709	up	yes	Amino acid	AVT6A
Pavir.6NG309900.v	GO:000367	NA	protein of u	22.97788	7.3746116	1.8808789	5.122228	4.3341466	2.1157479	0.01044587	0.185444601	up	yes	NA	NA
Pavir.5NG392400.v	GO:000467	04626(Plan	non-specific	2.618593	6.091195	0.612307	1.398685	4.3310903	2.1147303	0.037744821	0.365544935	up	yes	Probable se	PBL26
Pavir.5KG053700.v	GO:000563	NA	zinc finger c	6.795604	75.212097	8.652612	10.288304	4.3296587	2.1142533	0.022992135	0.280117861	up	yes	Zinc finger	Os01g0192000
Pavir.6NG346300.v	GO:000551	NA	fas-associat	16.23785	92.51268	9.522414	15.595595	4.329584	2.1142284	0.020625822	0.263686294	up	yes	Plant UBX	PUX8
Pavir.3KG300700.v	GO:000370	NA	no apical m	10.840253	22.834312	1.700827	6.086557	4.3242461	2.1124486	0.010856631	0.188567538	up	yes	NAC domai	NAC073
Pavir.9NG673900.v	GO:000382	01200(Carb	enoyl-coa hy	6.623092	11.546126	1.51254	2.693254	4.3200447	2.1110462	0.008946322	0.173089684	up	yes	Probable en	ECHIA
Pavir.2NG455700.v	GO:000367	NA	thioredoxin	1.174412	10.420115	1.973016	0.711764	4.3186134	2.1105682	0.044439977	0.398278861	up	yes	NA	NA
Pavir.2NG578900.v	GO:000382	01200(Carb	6-phosphog	7.0272078	43.764568	3.9495836	7.8682841	4.2978799	2.1036252	0.022231139	0.27450782	up	yes	Probable 6-	OsI_025867;OsI_025867;OsI_025867;OsI_025867
Pavir.3NG294900.v	NA	NA	leucine-rich	0.868455	11.762868	1.091314	1.848236	4.2970261	2.1033385	0.03342406	0.342845262	up	yes	NA	NA
Pavir.8NG245900.v	GO:000166	00010(Glyc	alcohol deh	25.639316	59.096146	14.013435	5.7072171	4.296788	2.1032586	0.009029909	0.173633972	up	yes	Alcohol deh	ADH2;ADH2
Pavir.9NG739600.v	GO:000428	NA	heat shock t	2.221883	3.990098	0.523254	0.928402	4.2792376	2.0973538	0.021181836	0.267787733	up	yes	Heat stress	HSFA2D
Pavir.4NG283300.v	GO:000467	04626(Plan	non-specific	8.769162	2.226668	0.333707	2.237227	4.2769787	2.096592	0.047267785	0.40946722	up	yes	Probable re	At5g57670;At5g57670
Pavir.1NG426000.v	GO:000970	NA	protein of u	10.729108	26.60198	2.627547	6.116696	4.269219	2.0939722	0.008221638	0.164665041	up	yes	Tobamoviru	TOM3
Pavir.8NG311400.v	GO:000842	00500(Star	beta-glucosi	4.225264	11.05742	1.186668	2.393795	4.2683541	2.0936799	0.007426727	0.157460964	up	yes	Beta-glucos	SFR2
Pavir.9NG572000.v	GO:000367	03013(RNA	thaumatin fa	17.884048	24.273426	6.277286	3.606064	4.2655045	2.0927164	0.002984082	0.093123668	up	yes	Thaumatin-	TLP1
Pavir.5KG209100.v	NA	NA	0	16.193178	40.582309	6.5544941	6.7591897	4.2644461	2.0923584	0.005222016	0.130466854	up	yes	NA	NA
Pavir.9NG290400.v	GO:000013	00790(Fola	exostosin h	238.15336	273.75422	9.706444	110.50153	4.2585161	2.0903508	0.04189994	0.385680547	up	yes	Probable gl	Os01g0926700;Os01g0926700
Pavir.4KG180500.v	GO:000588	01200(Carb	eama-like tr	108.57103	59.245728	8.510701	30.902292	4.2579044	2.0901436	0.017446635	0.242474193	up	yes	WAT1-rela	At1g44800
Pavir.5KG650800.v	NA	NA	0	16.542669	7.439745	1.710363	3.923626	4.2567378	2.0897482	0.013298361	0.209349606	up	yes	NA	NA
Pavir.3KG107500.v	GO:000367	00190(Oxic	myb-like dn	12.200513	58.08223	8.870269	7.644461	4.2557609	2.0894171	0.012998625	0.20735682	up	yes	Myb-relatec	MYBAS2
Pavir.5KG731100.v	GO:000104	NA	helix-loop-h	3.563757	7.767884	1.158126	1.506147	4.2531831	2.088543	0.009493002	0.178251151	up	yes	Transcriptic	BHLH128
Pavir.1KG234300.v	NA	NA	0	21.788595	5.959097	4.160231	2.366434	4.251435	2.0879499	0.012166524	0.200067694	up	yes	EPIDERM/	EPFL6
Pavir.9NG733000.v	NA	NA	0	4.270381	21.162359	2.855251	3.132739	4.2472917	2.0865432	0.020994019	0.266759651	up	yes	NA	NA
Pavir.7KG097500.v	NA	NA	0	4.209917	14.758181	1.40929	3.05729	4.2466715	2.0863325	0.01820336	0.246825997	up	yes	NA	NA
Pavir.6KG111200.v	GO:000551	NA	glutaredoxi	12.416269	49.77858	4.987012	9.661931	4.2456885	2.0859985	0.010915129	0.188905631	up	yes	Monothiol g	GRXS5
Pavir.2NG184600.v	NA	NA	0	3.44227	9.594846	0.788059	2.282946	4.2452279	2.085842	0.032150863	0.335743292	up	yes	NA	NA
Pavir.1KG550800.v	NA	NA	0	15.048186	28.933769	4.097823	6.263141	4.2449675	2.0857535	0.019967479	0.258492149	up	yes	NA	NA
Pavir.2NG192500.v	GO:000013	NA	solute carric	1.1201117	8.1974784	0.7622425	1.4331193	4.2442163	2.0854982	0.03518701	0.35293153	up	yes	GDP-fucos	GFT1;GFT1;GFT1;GFT1;GFT1;GFT1;GFT1;GFT1
Pavir.5NG152600.v	GO:000573	00941(Flav	nad depende	142.85556	70.668625	4.241143	46.073303	4.2437948	2.0853549	0.043032927	0.39128833	up	yes	Isoflavone r	IRL;IRL
Pavir.3KG206200.v	GO:000367	NA	desiccation-	13.374969	47.070114	5.376945	8.867834	4.2433149	2.0851917	0.009145607	0.174757488	up	yes	Late embry	LEA14-A
Pavir.3NG021600.v	GO:000487	NA	late embryo	110.46344	80.863655	5.197595	39.895576	4.2429284	2.0850603	0.0313935	0.331486598	up	yes	NDR1/HIN	NHL1
Pavir.7NG323300.v	NA	NA	0	9.121306	10.342023	1.857161	2.730444	4.2425904	2.0849454	0.008648716	0.169658116	up	yes	NA	NA
Pavir.2KG111900.v	GO:000387	00230(Puri	amp deamir	3.212276	10.317479	1.414279	1.782699	4.232045	2.081355	0.008145486	0.164014116	up	yes	Probable Al	AMPD;AMPD
Pavir.6KG334400.v	GO:000573	03040(Spli	oligopeptide	5.914513	27.201401	4.982453	2.845588	4.2306445	2.0808015	0.01467807	0.220599648	up	yes	Oligopeptid	OPT5
Pavir.2NG130000.v	GO:000576	04144(Endo	ras-related p	3.362625	14.92958	1.560827	2.765766	4.2278543	2.0799257	0.022848122	0.279178039	up	yes	Ras-related	RABA2A
Pavir.2NG391600.v	NA	NA	0	3.072231	5.960145	0.559982	1.577439	4.2258292	2.0792344	0.046442524	0.40582121	up	yes	NA	NA
Pavir.3NG165600.v	GO:000467	04140(Regu	serine/threo	7.823478	13.378291	0.327519	4.690549	4.225086	2.0789807	0.042106075	0.386620619	up	yes	CBL-intera	CIPK22
Pavir.4KG246400.v	GO:000467	04075(Plan	serine/threo	5.618466	7.261289	0.317558	2.731567	4.2240823	2.078638	0.033915884	0.344597484	up	yes	CBL-intera	CIPK25
Pavir.6KG113600.v	NA	NA	0	16.358261	17.636761	2.270489	5.779912	4.2227737	2.078191	0.031222542	0.330457627	up	yes	NA	NA
Pavir.6NG096200.v	GO:000455	00052(Gala	galactinol--s	3.6168588	3.7070841	0.6123968	1.1228387	4.22072	2.0774891	0.007467555	0.157697172	up	yes	Probable ga	RFS1;RFS1;RFS1
Pavir.7KG270100.v	NA	NA	senescence r	4.937764	53.766441	6.039427	7.869186	4.2207088	2.0774853	0.025465739	0.295794776	up	yes	NA	NA
Pavir.9NG748000.v	GO:000467	04075(Plan	calmodulin-	8.9921491	3.5221825	0.8126336	2.1529011	4.2199242	2.0772171	0.011150034	0.191122367	up	yes	Calmodulin	CRCK2;CRCK2;CRCK1
Pavir.7NG223400.v	GO:000022	NA	scc1 / rad21	3.859706	2.683787	0.569808	0.981735	4.21741	2.0763573	0.010538724	0.186164311	up	yes	Sister chron	SYN2
Pavir.2NG605400.v	NA	NA	0	2.308756	6.502242	0.637728	1.45232	4.2156917	2.0757694	0.036139646	0.357335506	up	yes	NA	NA
Pavir.4KG380300.v	NA	NA	wound-indu	16.476137	15.493964	3.978548	3.612154	4.211745	2.0744181	0.014813927	0.221981462	up	yes	NA	NA
Pavir.1NG394500.v	GO:000521	NA	major facilit	23.642559	91.320824	10.260936	17.056433	4.2084354	2.073284	0.015919926	0.231765709	up	yes	NA	NA
Pavir.5KG657100.v	GO:000467	04075(Plan	serine/threo	7.6903933	42.847511	2.9934043	9.0215745	4.2062417	2.0725317	0.027697909	0.309361931	up	yes	Serine/threc	SAPK4;SAPK4
Pavir.9NG383100.v	GO:000697	03018(RNA	peptide met	1.034797	2.365334	0.265637	0.544403	4.1974853	2.0695253	0.043766086	0.395199029	up	yes	Peptide met	MSRA4
Pavir.2KG096800.v	GO:000950	NA	protein seed	7.709427	14.074737	2.012466	3.180535	4.1949085	2.0686393	0.005715826	0.136102468	up	yes	Protein SEE	SPD1
Pavir.2KG166200.v	GO:000367	NA	mediator of	23.855266	60.23762	4.179347	15.920486	4.1837604	2.0648002	0.023299777	0.281238111	up	yes	Mediator of	MED33A;MED33A
Pavir.9NG171600.v	GO:000367	NA	zinc-finger c	34.389657	56.004669	9.4557527	12.176436	4.1786954	2.0630526	0.007548066	0.157919056	up	yes	FCS-Like Z	FLZ8;FLZ8;FLZ8
Pavir.4NG216300.v	GO:000387	00051(Fruc	phosphofruc	25.848732	62.594668	1.6787528	19.497989	4.1764404	2.0622738	0.046339084	0.405743446	up	yes	Pyrophosph	PFP-ALPHA;PFP-ALPHA;PFP-ALPHA
Pavir.5NG145500.v	GO:000563	04140(Regu	arm repeat s	1.988883	6.716693	0.775269	1.311874	4.1710491	2.0604103	0.019603194	0.256267806	up	yes	NA	NA
Pavir.9NG224000.v	GO:000576	NA	secretory ca	3.5518168	6.5753787	0.6809893	1.7471517	4.170761	2.0603106	0.018326375	0.247422531	up	yes	Secretory ca	SCAMP4;SCAMP4
Pavir.9NG743400.v	GO:000449	00906(Carc	fad/nad(p)-b	8.0741227	14.152795	1.6333596	3.697006	4.1698674	2.0600015	0.007996115	0.162529436	up	yes	Monooxyge	MO2;MO2
Pavir.9NG292400.v	NA	NA	0	5.098176	14.764538	2.462464	2.309348	4.1625098	2.0574537	0.008350489	0.165831352	up	yes	NA	NA
Pavir.7NG283200.v	NA	NA	0	11.794837	45.566654	1.409228	12.372404	4.1621697	2.0573358	0.037453391	0.364237061	up	yes	NA	NA
Pavir.5KG267600.v	GO:004316	NA	ring finger c	10.067676	12.521316	1.205989	4.221401	4.1620359	2.0572894	0.014485448	0.219044633	up	yes	RING-H2 f	ATL46
Pavir.3KG246100.v	GO:000370	04626(Plan	wrky transc	4.536463	9.270458	0.872585	2.444928	4.1618288	2.0572176	0.014360681	0.218097771	up	yes	WRKY trar	WRKY24
Pavir.1NG356100.v	NA	NA	0	2.114346	4.671762	0.689352	0.941384	4.1613774	2.0570611	0.010527493	0.186164311	up	yes	NA	NA
Pavir.7KG331200.v	NA	NA	0	9.961099	11.905141	0.385462	4.869452	4.1611033	2.0569661	0.037019427	0.36259301	up	yes	NA	NA
Pavir.5KG619900.v	GO:000552	03040(Spli	heat shock 7	25.240233	25.711116	4.5809701	7.6667576	4.1600655	2.0566062	0.007284755	0.156441785	up	yes	Heat shock	HSP70;HSP70;HSP70
Pavir.7KG081000.v	GO:000467	04075(Plan	phototropin	4.385409	31.115215	2.171718	6.36293	4.1595885	2.0564408	0.029016104	0.31784694	up	yes	Phototropin	PHOT2
Pavir.4KG389600.v	GO:000013	NA	polysacchar	46.497746	49.951206	5.244351	17.96862	4.1549594	2.0548344	0.0143244	0.217778448	up	yes	Protein IRR	IRX15
Pavir.6KG113500.v	NA	NA	small subun	162.29791	113.10263	16.166601	50.187485	4.1504685	2.0532742	0.013187707	0.208808142	up	yes	NA	NA
Pavir.3KG020400.v	GO:000441	00650(Buta	hydroxymet	23.657896	6.706103	0.870801	6.449592	4.1478646	2.0523688	0.033473661	0.343010075	up	yes	Hydroxyme	HMGCL
Pavir.1KG017100.v	GO:000382	00360(Pher	amp-binding	2.397678	7.875671	0.807202	1.671548	4.1445684	2.0512219	0.0172252	0.241168064	up	yes	Probable ac	AAE1
Pavir.7NG047700.v	GO:000563	04141(Prot	dnaj homol	5.462073	9.684264	0.896385	2.758408	4.1442394	2.0511073	0.019497359	0.25596585	up	yes	NA	NA
Pavir.7KG174200.v	GO:000029	04626(Plan	ferric-chelat	2.710703	2.616584	0.373201	0.913818	4.139245	2.0493677	0.038107428	0.367696562	up	yes	Ferric reduc	FRO6
Pavir.6NG344900.v	GO:000382	NA	molybdopte	2.687751	10.542544	1.051969	2.145213	4.1381113	2.0489725	0.018386344	0.247552699	up	yes	NA	NA
Pavir.4KG332000.v	GO:000459	NA	rna-directed	1.092045	1.072523	0.308609	0.21476	4.1358353	2.0481787	0.031732976	0.33379146	up	yes	Retrovirus-r	RE2
Pavir.7KG158600.v	NA	NA	t22j18.15	5.735404	5.669918	1.318017	1.439711	4.1357676	2.0481551	0.014022603	0.215049498	up	yes	NA	NA
Pavir.3KG067900.v	GO:000521	NA	oligopeptide	4.104495	6.010138	0.819322	1.626383	4.1356717	2.0481217	0.008183724	0.164298747	up	yes	Protein NR'	NPF4.3
Pavir.5NG085100.v	GO:000481	00970(Ami	arginyl-trna	2.0050739	8.8468286	0.6036176	2.0205356	4.1353922	2.0480241	0.027913692	0.310335091	up	yes	Arginine--tl	EMB1027;EMB1027;EMB1027;EMB1027;EMB1027;EMB1027;EMB1027;EMB1027;EMB1027;EMB1027

Pavir.7KG231100.v4	GO:000588	NA	ubiquitin ca	41.168619	148.21596	13.714673	32.114414	4.13241	2.0469834	0.022030901	0.273574962	up	yes	NA	NA
Pavir.4NG293300.v4	GO:000562	03010(Ribo	ubiquitin b	183.91366	361.31189	84.824033	47.152551	4.1312294	2.0465712	0.011212869	0.19179247	up	yes	Polyubiquit	-;-;-
Pavir.3NG312200.v4	NA	NA	protein of u	5.924507	14.354903	1.592512	3.317968	4.1298223	2.0460797	0.012873435	0.206353906	up	yes	NA	NA
Pavir.7NG013200.v4	GO:000573	NA	alcohol deh	2.630791	6.38836	0.988386	1.195597	4.12968	2.04603	0.019262478	0.254572313	up	yes	Chloroplast	CEQORH
Pavir.7NG131800.v4	GO:000382	NA	snf1-related	12.587855	71.224022	5.359616	14.948924	4.1269277	2.0450682	0.029935541	0.32320922	up	yes	SNF1-relate	KING1
Pavir.2NG202400.v4	GO:000367	NA	remorin, c-t	110.82645	60.479687	8.125688	33.500835	4.1153121	2.0410018	0.02329307	0.281238111	up	yes	Remorin 4.	REM4.1
Pavir.3KG107700.v4	GO:000367	00190(Oxic	myb-like dn	4.702896	9.622496	1.123693	2.357448	4.1151427	2.0409425	0.01615288	0.233131218	up	yes	Myb-relatec	MYBAS2;MYBAS2
Pavir.1KG543400.v4	GO:000467	04626(Plan	abc1 protein	6.598786	11.383062	1.75171	2.618199	4.114925	2.0408662	0.004977376	0.127221065	up	yes	Protein AC	ABC1K3
Pavir.8NG053800.v4	NA	NA	emb	10.735264	4.129402	1.057129	2.562233	4.1069852	2.0380797	0.031777801	0.333937523	up	yes	NA	NA
Pavir.4KG193300.v4	NA	NA	0	38.634912	92.00952	12.767267	19.104725	4.0990357	2.0352845	0.013143885	0.208381427	up	yes	TORTIFOL	TOR1L3;TOR1L3
Pavir.9NG184100.v4	GO:000367	NA	transcription	46.697147	45.940201	10.65142	11.952205	4.0983403	2.0350398	0.007241453	0.156279779	up	yes	Nuclear trai	NFYA10
Pavir.1NG407100.v4	GO:000367	00190(Oxic	myb-like dn	4.383241	13.224944	2.301309	2.005859	4.088112	2.0314347	0.012499434	0.203307889	up	yes	Transcriptic	MYB20
Pavir.5KG650700.v4	GO:000639	NA	tetratricope	28.59687	31.393866	4.546802	10.128944	4.087747	2.0313059	0.008910822	0.172758674	up	yes	NA	NA
Pavir.9NG265200.v4	GO:000563	00500(Star	beta-amylas	5.785932	15.688144	1.158634	4.100005	4.0835806	2.0298347	0.018019646	0.245284518	up	yes	Beta-amylas	BAMY1
Pavir.1KG299400.v4	NA	NA	0	27.24478	46.084957	4.168817	13.813251	4.0779368	2.0278394	0.010779777	0.188149509	up	yes	NA	NA
Pavir.3KG124800.v4	GO:000443	00562(Inos	phosphoino	10.156336	7.2314828	1.1277645	3.1370132	4.0770751	2.0275345	0.008692065	0.170274743	up	yes	Phosphoino	PLC2;PLC2;PLC2
Pavir.7NG147600.v4	GO:000367	NA	myb/sant-lil	1.23984	1.924763	0.203189	0.573772	4.0730526	2.0261104	0.045073471	0.400506709	up	yes	Trihelix tra	ASR3
Pavir.9NG319500.v4	NA	NA	0	47.693939	161.63995	11.435235	39.960266	4.0730003	2.0260919	0.026469251	0.303210111	up	yes	Nodulin-rel	NRP1
Pavir.3KG483700.v4	GO:000588	NA	probable me	1.1147788	3.6085306	0.5244859	0.6354781	4.071945	2.0257181	0.020840967	0.265467377	up	yes	CSC1-like	HYP1;HYP1;HYP1
Pavir.9NG633600.v4	GO:000367	03013(RNA	cct motif fa	1.5391829	21.798769	3.0650927	2.6776644	4.0638933	2.0228625	0.036295768	0.3582193	up	yes	Zinc finger	COL12;NA
Pavir.9NG489300.v4	NA	NA	0	3.765989	5.035141	0.44778	1.719439	4.0610248	2.0218438	0.049664598	0.420944717	up	yes	NA	NA
Pavir.9KG401400.v4	GO:000384	01230(Bios	3-deoxy-7-p	252.12836	844.59415	59.691456	210.44631	4.0598637	2.0214313	0.030882626	0.32878257	up	yes	Phospho-2-	DAHPS2;DAHPS2
Pavir.1NG556900.v4	NA	NA	inosine-5-m	119.26159	319.93219	19.800584	88.382337	4.059733	2.0213849	0.030454702	0.326146251	up	yes	NA	NA
Pavir.7NG432500.v4	GO:004652	00270(Cyst	s-methyl-5-t	21.32766	92.56958	11.387552	16.670551	4.059335	2.0212434	0.018731651	0.250273838	up	yes	Methylthior	MTK1
Pavir.5NG426000.v4	GO:000370	NA	nac domain	109.22218	97.007538	10.209959	40.596889	4.059093	2.0211574	0.021214542	0.268025488	up	yes	NAC domai	NAC073
Pavir.9NG667900.v4	GO:000367	NA	fantastic fou	6.83588	6.109453	1.77505	1.417019	4.0554678	2.0198683	0.006518842	0.14647695	up	yes	Protein FAI	At5g22090
Pavir.1NG344500.v4	GO:000551	00940(Pher	shikimate o-	165.8381	151.66618	7.203749	71.135399	4.0529454	2.0189707	0.044103263	0.396623237	up	yes	Hydroxycin	HCT2
Pavir.5KG405500.v4	GO:000472	NA	protein pho	1.397569	4.865897	0.429965	1.116159	4.0510761	2.0183052	0.038558087	0.37029766	up	yes	Probable pr	PP2C06
Pavir.2NG258600.v4	GO:000535	NA	sugar transp	3.2812011	11.437628	2.1902746	1.4448419	4.0490668	2.0175895	0.0105949	0.186717716	up	yes	Sugar trans	STP14;STP14;STP14;STP14
Pavir.5NG467600.v4	GO:000384	00561(Glyc	lysocardioli	12.060206	16.921209	2.177892	4.983137	4.047102	2.0168892	0.007298132	0.156441785	up	yes	Probable 1-	LPAT5
Pavir.2KG428900.v4	GO:000981	00942(Antl	glucosyl/glu	1.888051	3.847647	0.485994	0.931963	4.0450437	2.0161553	0.032056691	0.33527229	up	yes	UDP-glycos	UGT88B1
Pavir.7KG171400.v4	GO:000395	00592(alph	nadh oxidor	17.673576	12.321421	1.066765	6.350535	4.0439239	2.0157559	0.019912875	0.258492149	up	yes	Putative 12-	OPR13
Pavir.9KG060900.v4	GO:000433	01200(Carb	fructose-bis	172.70212	508.26852	56.562267	111.94209	4.0412642	2.0148067	0.020087946	0.259513906	up	yes	Fructose-bi	ALDP
Pavir.3KG205200.v4	GO:000419	NA	hemoglobin	10.920835	21.515347	0.841897	7.184523	4.0411768	2.0147755	0.031028776	0.329613858	up	yes	Vacuolar-p	-
Pavir.2KG534500.v4	GO:000474	NA	[pyruvate d	3.3926276	16.855604	1.4841637	3.5265799	4.0409634	2.0146993	0.0189367	0.25134686	up	yes	[Pyruvate d	PDK;PDK;PDK;PDK;PDK
Pavir.2KG442900.v4	GO:000455	00230(Puri	nucleoside	11.600658	38.334732	4.321801	8.036067	4.0407771	2.0146328	0.012527125	0.203670128	up	yes	Nucleoside	NDKR
Pavir.2KG207000.v4	GO:000906	00430(Taur	cysteamine	19.846097	75.461795	6.3982256	17.19102	4.0403112	2.0144664	0.023093217	0.280803059	up	yes	Plant cystei	PCO5;PCO5;PCO5
Pavir.5NG053400.v4	NA	NA	late embryo	381.53029	266.43789	19.998127	140.43835	4.0387834	2.0139208	0.036628766	0.359804611	up	yes	NA	NA
Pavir.3KG503800.v4	GO:000456	00531(Glyc	heparanase-	22.855959	29.41869	2.491941	10.451829	4.0385953	2.0138536	0.018783579	0.250445493	up	yes	Heparanase	At5g61250
Pavir.9KG357000.v4	GO:000015	04075(Plan	histidine kir	4.7295923	8.7494742	1.1188937	2.2187768	4.0384653	2.0138071	0.007237496	0.156279779	up	yes	Probable hi	HK5;HK5;HK5;HK5;HK5;HK5;HK5;HK5;HK5;HK5;HK5;HK5;HK5;HK5
Pavir.6NG264400.v4	GO:000367	NA	wd40 repeat	1.5008891	4.1996932	0.4069658	1.0053931	4.0362135	2.0130025	0.030064262	0.324133288	up	yes	NA	NA
Pavir.7NG218800.v4	GO:000098	NA	b-box zinc f	4.974016	39.232731	5.146074	5.818483	4.031786	2.0114191	0.02237807	0.275753638	up	yes	B-box zinc	BBX24
Pavir.5NG241200.v4	GO:000367	NA	copine//sub	14.259869	34.253948	4.108563	7.92543	4.0313981	2.0112803	0.008526252	0.168078108	up	yes	B2 protein	-
Pavir.1NG424700.v4	GO:000223	04626(Plan	non-specific	8.951284	15.050241	2.269213	3.689962	4.027659	2.0099416	0.007961899	0.162325709	up	yes	Serine/threc	NFP
Pavir.6KG172300.v4	NA	NA	0	1.727194	1.14503	0.219003	0.494235	4.0270204	2.0097128	0.041629986	0.384111502	up	yes	NA	NA
Pavir.9NG316600.v4	GO:000950	00860(Porp	chlorophylla	1.047957	5.833548	0.94659	0.762266	4.026966	2.0096933	0.040755581	0.380028688	up	yes	Chlorophyll	CLH2
Pavir.8KG186300.v4	GO:000333	NA	amino acid	1.4655789	9.378451	1.1664684	1.5272544	4.0256667	2.0092277	0.02084286	0.265467377	up	yes	Amino acid	AAP3;AAP3;AAP3
Pavir.4NG234600.v4	NA	NA	0	6.215495	3.681498	0.629782	1.829123	4.0249595	2.0089743	0.03904537	0.372551269	up	yes	NA	NA
Pavir.9NG744300.v4	GO:000432	00360(Pher	4-coumarate	11.441147	6.0741898	0.9123809	3.4437042	4.0208894	2.0075147	0.016534064	0.236300643	up	yes	4-coumarate	4CLL1;4CLL1
Pavir.5KG778200.v4	GO:000815	03013(RNA	protein of u	10.963836	7.028996	2.39313	2.08265	4.0200439	2.0072112	0.005328366	0.13145285	up	yes	NA	NA
Pavir.7NG250900.v4	GO:000367	NA	remorin, c-t	87.697784	49.24197	7.468938	26.617085	4.0174753	2.0062891	0.017678766	0.243312915	up	yes	Remorin 4.	REM4.1
Pavir.5NG106300.v4	NA	NA	0	11.998852	6.465191	1.027517	3.578929	4.0083055	2.0029925	0.038578482	0.37029766	up	yes	Endo-1,4-b	XYN3
Pavir.6KG372800.v4	GO:000588	NA	eukaryotic c	21.766612	19.581862	4.0399478	6.2824444	4.0057065	2.0020567	0.004714891	0.12336531	up	yes	Cytochrome	At3g25290;At5g35735
Pavir.4NG184100.v4	GO:000941	00196(Phot	light-harves	177.51488	486.07346	48.498013	117.17888	4.0053162	2.0019161	0.02186712	0.272237679	up	yes	Chlorophyll	CAB8
Pavir.8KG148400.v4	GO:000521	NA	protein nrt1	20.198103	50.712646	5.788814	11.926878	4.0027084	2.0009765	0.009594712	0.178946539	up	yes	Protein NR	NPF4.4
Pavir.9KG234100.v4	NA	NA	carboxylate	25.921099	85.401215	12.979877	14.876118	3.9963503	1.998683	0.01416933	0.216504373	up	yes	NA	NA
Pavir.6KG112900.v4	NA	NA	0	519.81667	506.34514	60.810333	196.40056	3.9895736	1.9962346	0.020900365	0.266019443	up	yes	NA;Unchar	NA;AtMg00030
Pavir.8NG014500.v4	GO:000367	NA	cotton fibre	16.10881	22.755035	2.203663	7.539508	3.9888292	1.9959653	0.016106977	0.232779784	up	yes	NA	NA
Pavir.9NG009300.v4	GO:000573	NA	nucleotide-c	26.019833	12.125072	1.749977	7.82093	3.9855058	1.9947628	0.018421763	0.247720805	up	yes	Uncharacter	At4g15970
Pavir.5KG720400.v4	GO:000563	00500(Star	sucrose-pho	5.351026	7.242317	0.602786	2.558285	3.9838849	1.994176	0.016124036	0.232831826	up	yes	Sucrose-ph	SPS
Pavir.5KG454200.v4	GO:000552	03050(Prot	bcs1 aaa-typ	3.699657	7.47807	0.76704	2.039169	3.9832126	1.9939325	0.018088339	0.245773676	up	yes	AAA-ATPa	At5g17760
Pavir.2NG374200.v4	GO:000016	03040(Spli	u2 snrnp au	18.36065	44.544581	6.8075819	8.9962144	3.9803873	1.9929088	0.008123479	0.163658692	up	yes	Splicing fac	U2AF35A;U2AF35A;U2AF35A;U2AF35A;U2AF35A;U2AF35A;U2AF35A
Pavir.1NG154500.v4	GO:000519	03010(Ribo	leucine rich	8.641396	2.905785	0.377887	2.523171	3.9803344	1.9928896	0.047286037	0.40946722	up	yes	Leucine-ricl	LRX6
Pavir.8KG090600.v4	GO:000166	00010(Glyc	alcohol deh	68.227295	89.9403	17.616402	22.124607	3.9799592	1.9927536	0.009588036	0.178946539	up	yes	Alcohol del	ADH1
Pavir.9NG111500.v4	NA	00190(Oxic	atp synthase	3.15756	16.093937	2.326272	2.517782	3.9742532	1.9906838	0.026347461	0.302645412	up	yes	ATP syntha	ATPD
Pavir.5KG140700.v4	GO:000563	04140(Regu	arm repeat s	14.778483	30.041126	2.683971	8.593832	3.9741436	1.990644	0.012949397	0.207263269	up	yes	U-box dom	PUB45
Pavir.3KG305000.v4	NA	NA	drought ind	122.88255	705.43451	93.909119	114.64361	3.9717393	1.9897709	0.028588121	0.314260236	up	yes	Protein DEI	DI19-1
Pavir.1NG546800.v4	GO:000367	NA	domain of u	15.708056	49.076294	5.498248	10.817701	3.9706149	1.9893624	0.01156392	0.19504256	up	yes	NA	NA
Pavir.5KG689500.v4	NA	NA	0	4.369934	6.859261	1.114017	1.714652	3.9697805	1.9890592	0.021666666	0.27085151	up	yes	NA	NA
Pavir.5KG184000.v4	GO:000561	04144(Endo	cupin doma	150.18276	57.170044	11.563791	40.758781	3.9629703	1.9865822	0.027267242	0.307384367	up	yes	Germin-like	Os05g0277500
Pavir.2NG464900.v4	GO:000367	NA	myb/sant-lil	1.8641114	4.411549	0.4554354	1.1282018	3.9628144	1.9865254	0.040158771	0.377598979	up	yes	Trihelix tra	ASR3;ASR3
Pavir.8NG050000.v4	GO:000367	NA	homeobox p	3.5844632	11.445849	0.685063	3.1078701	3.9627148	1.9864891	0.02417906	0.286877253	up	yes	BEL1-like	BLH1;BLH1;BLH1;BLH1;BLH1;BLH1;BLH1
Pavir.1KG498900.v4	GO:000367	00190(Oxic	myb-like dn	5.226856	10.943527	2.101585	1.981745	3.960097	1.9855358	0.011099134	0.190684854	up	yes	Transcriptic	MYB20
Pavir.9NG693700.v4	GO:000563	NA	phosphatidy	2.489492	6.760502	0.927435	1.40858	3.9597323	1.9854029	0.011301108	0.192482559	up	yes	NA	NA
Pavir.2KG017300.v4	GO:000467	04075(Plan	serine/threo	3.425543	23.787033	3.05793	3.820834	3.956027	1.9840523	0.022630386	0.277230089	up	yes	Transcriptic	EMB1444
Pavir.6NG368300.v4	GO:000419	04145(Phag	kdel-tailed	47.598198	99.983116	9.13901	28.176994	3.9549067	1.9836437	0.02109911	0.267176726	up	yes	KDEL-taile	CEP1
Pavir.9NG730400.v4	GO:000367	04712(Circ	dof domain,	6.883934	34.395622	2.63457	7.808629	3.9527693	1.9828637	0.024139085	0.286865509	up	yes	Cyclic dof f	CDF1
Pavir.1NG354700.v4	GO:000016	03013(RNA	rna recognit	12.099236	24.669844	3.984815	5.325709	3.9491956	1.9815588	0.006830602	0.150702417	up	yes	Oligouridyl	UBP1B
Pavir.4NG274400.v4	GO:000588	00190(Oxic	cu+-exporti	14.292111	10.262795	0.5645534	5.6543362	3.948439	1.9812824	0.042310173	0.387925849	up	yes	Cation-tran	HMA9;HMA9
Pavir.7KG252000.v4	GO:000842	00500(Star	glycosyl hy	5.6834308	14.865935	0.698351	4.5073589	3.9474666	1.9809271	0.04154312	0.383774816	up	yes	Probable in	BGLU14;BGLU14
Pavir.6KG193300.v4	GO:000372	03015(mRN	cleavage an	8.356134	8.416008	1.52888	2.72079	3.9466928	1.9806442	0.023475007	0.281887642	up	yes	Pre-mRNA	CFIS1
Pavir.8KG273000.v4	NA	NA	0	3.527087	10.802444	1.450887	2.183146	3.9431483	1.979348	0.039037728	0.372551269	up	yes	NA	NA

Pavir.8NG032500.v	GO:000406	NA	aluminum i	103.72234	308.00256	16.675354	87.76387	3.942244	1.9790171	0.03538979	0.354053501	up	yes	Stem-specif	TSJT1
Pavir.2KG361800.v	NA	NA	0	1.758814	9.835804	1.575289	1.365893	3.9421627	1.9789873	0.031752715	0.333905614	up	yes	NA	NA
Pavir.7KG340500.v	NA	NA	0	1.739889	11.649477	2.158412	1.240148	3.9397174	1.9780922	0.037247814	0.363533126	up	yes	NA	NA
Pavir.7KG370400.v	GO:000552	04146(Pero	oxalate--coa	18.877056	8.369192	1.548897	5.369498	3.9382325	1.9775483	0.016087097	0.232779784	up	yes	Oxalate--Co	AAE3
Pavir.1NG459000.v	NA	NA	0	2.490817	4.836395	0.754064	1.106494	3.9381798	1.977529	0.00817267	0.164297344	up	yes	NA	NA
Pavir.9KG245800.v	GO:000557	03010(Ribo	protease inh	911.10181	388.72122	108.22653	222.17896	3.9340237	1.9760057	0.020172355	0.260280188	up	yes	pEARLI1-l	At4g12490
Pavir.1KG453200.v	GO:000367	NA	zinc-finger (	13.313476	8.478082	1.271974	4.273133	3.9298715	1.9744821	0.019100442	0.252941089	up	yes	FCS-Like Z	FLZ2
Pavir.8KG396700.v	NA	NA	0	4.417956	2.064552	0.672359	0.979349	3.92473	1.9725934	0.02226161	0.274511146	up	yes	NA	NA
Pavir.9NG061900.v	GO:000367	NA	remorin, c-t	41.055096	17.514177	1.568394	13.360652	3.9231759	1.972022	0.035980103	0.356637568	up	yes	Remorin 4.	REM4.1
Pavir.4KG376200.v	GO:000367	NA	domain of u	9.479663	5.33235	0.618248	3.167905	3.9121538	1.9679631	0.043748056	0.395155675	up	yes	NA	NA
Pavir.5NG415700.v	GO:000573	01230(Bios	glutamate sy	58.552856	88.824249	9.412362	28.259418	3.9121354	1.9679563	0.021902349	0.272495684	up	yes	Glutamate s	Os01g0681900
Pavir.9NG365100.v	GO:000557	00940(Phen	nad depende	7.380434	20.863855	2.290656	4.929527	3.9118522	1.9678519	0.012219438	0.200489502	up	yes	Cinnamoyl-	SNL6
Pavir.8KG175000.v	NA	NA	0	546.28314	603.08838	95.785568	198.1219	3.9106577	1.9674113	0.015724157	0.230063767	up	yes	NA	NA
Pavir.8NG080900.v	NA	NA	0	5.841687	13.663284	1.880849	3.109755	3.9083388	1.9665555	0.022797509	0.27874091	up	yes	Adenylyltra	MOCS3
Pavir.1KG237800.v	GO:000393	00740(Ribo	monofunctio	8.654208	10.608343	0.651868	4.281711	3.9043767	1.9650923	0.027417445	0.308245262	up	yes	Probable m	RIBA3;RIBA3
Pavir.2NG053100.v	NA	NA	0	1.521679	3.672902	0.673876	0.656727	3.90393	1.9649272	0.031877701	0.334191083	up	yes	NA	NA
Pavir.5KG093400.v	NA	NA	0	21.21575	32.596951	1.453755	12.332812	3.9032706	1.9646835	0.031804738	0.333940095	up	yes	NA	NA
Pavir.9NG691200.v	NA	NA	0	3.092918	17.299229	2.929733	2.296493	3.9018877	1.9641723	0.021821551	0.271850514	up	yes	NA	NA
Pavir.1NG027300.v	GO:000367	04075(Plan	aux/iaa fam	4.7316751	5.4512859	1.1874683	1.4228671	3.9010164	1.96385	0.005833875	0.137393622	up	yes	Auxin respo	ARF5;ARF5;ARF5;ARF5
Pavir.2NG015800.v	GO:000447	01230(Bios	s-adenosylm	758.11591	1905.3378	187.47516	496.23786	3.8955727	1.9618354	0.025848156	0.298500657	up	yes	S-adenosylm	SAM1;SAM1
Pavir.5NG586400.v	GO:000472	NA	protein phos	4.358704	10.539762	1.60952	2.2168	3.8936801	1.9611344	0.009687152	0.179918608	up	yes	Probable pr	Os01g0846300
Pavir.6KG215500.v	GO:000367	NA	camp-respo	7.366692	16.644941	2.933785	3.245172	3.8860334	1.9582983	0.012754949	0.20533064	up	yes	bZIP transc	BZIP44
Pavir.5NG423300.v	NA	NA	0	6.188101	11.345035	2.675473	1.846071	3.8776878	1.9551967	0.012081547	0.199271271	up	yes	NA	NA
Pavir.1NG356200.v	GO:000551	00360(Phen	phenylalani	2311.8904	5149.8911	391.84497	1532.7744	3.8770167	1.9549469	0.032316024	0.336719345	up	yes	Phenylalani	PAL
Pavir.7KG250800.v	GO:000980	00940(Phen	coumaroylq	33.268974	75.059692	7.429392	20.524183	3.8753063	1.9543104	0.021692066	0.270864201	up	yes	Cytochrome	CYP98A1
Pavir.7NG416300.v	GO:000521	NA	oligopeptide	2.3621792	6.5749772	1.3204805	0.9860862	3.8746577	1.9540689	0.018093045	0.245773676	up	yes	Protein NR	NPF5.10;NPF5.10
Pavir.1NG132700.v	NA	NA	0	15.060883	22.432182	4.425028	5.256693	3.872562	1.9532883	0.006420209	0.145641094	up	yes	Polyadenyla	CID11
Pavir.5NG005300.v	GO:000550	NA	drepp plasm	19.192238	68.421088	3.0068156	19.661488	3.8650147	1.9504739	0.045369125	0.401486498	up	yes	Salt stress r	Os01g0233000;Os01g0233000;Os01g0233000;Os01g0233000
Pavir.2NG172100.v	NA	NA	f-box-like (f	2.752159	13.729077	1.749861	2.516652	3.8629288	1.9496951	0.038979804	0.372289827	up	yes	NA	NA
Pavir.1NG356400.v	GO:000551	00360(Phen	phenylalani	338.84979	249.01611	22.011211	130.29889	3.8596646	1.9484755	0.038172942	0.368162761	up	yes	Phenylalani	ZB8
Pavir.1KG029400.v	GO:000573	NA	phosphatidy	14.691685	59.956841	7.617205	11.74389	3.8555942	1.9469532	0.023375984	0.281779229	up	yes	NA	NA
Pavir.5NG023000.v	GO:000367	NA	angiotensin-	5.868735	15.994825	2.35157	3.321719	3.8537716	1.9462711	0.009790291	0.180534287	up	yes	NA	NA
Pavir.7NG437400.v	NA	NA	f-box and le	8.094278	17.983076	2.616551	4.153356	3.8519516	1.9455896	0.010095605	0.182016394	up	yes	NA	NA
Pavir.7NG220100.v	GO:000098	NA	b-box zinc f	2.138024	16.525715	2.095153	2.750892	3.8513342	1.9453583	0.038314208	0.368933808	up	yes	B-box zinc	BBX24
Pavir.4KG275500.v	GO:000414	00561(Glyc	sterol o-acyl	2.515242	5.779329	0.927606	1.227504	3.8487924	1.9444059	0.017450036	0.242474193	up	yes	Diacylglyce	DGAT1-2
Pavir.9KG308400.v	GO:000436	00480(Glut	glutathione	4.825512	10.382245	0.596768	3.355239	3.8481098	1.94415	0.045752254	0.403146483	up	yes	Glutathione	GSTU17
Pavir.4KG373400.v	NA	NA	0	1.447457	5.936616	1.237662	0.681378	3.8477953	1.944032	0.040029822	0.377133998	up	yes	NA	NA
Pavir.8KG167200.v	GO:000166	00010(Glyc	alcohol deh	55.78857	86.295715	18.393812	18.605257	3.8402124	1.9411861	0.011433455	0.193857941	up	yes	Alcohol deh	ADH1
Pavir.1NG251900.v	GO:000367	NA	lysophospha	2.3184972	1.418942	0.2636964	0.7102206	3.8375335	1.9401794	0.038343994	0.369126024	up	yes	Uncharacte	At2g33490;At2g33490
Pavir.4NG272800.v	GO:000579	NA	gdsl/sgnh-li	2.8717513	3.6271726	0.9851899	0.7101374	3.833433	1.938637	0.010678942	0.187407705	up	yes	NA	NA
Pavir.3NG030100.v	GO:000433	01200(Carb	fructose-bis	167.95964	417.9288	45.138315	107.90255	3.8283137	1.936709	0.024785106	0.291562555	up	yes	Fructose-bi	ALDP;ALDP
Pavir.3NG276300.v	GO:000367	00230(Puri	transcriptio	19.827873	18.643698	3.380986	6.668804	3.828097	1.9366274	0.007409554	0.157450594	up	yes	Nuclear tra	NFYA10
Pavir.7NG221500.v	GO:000521	NA	oligopeptide	1.30102	2.074066	0.246471	0.635581	3.8264025	1.9359886	0.039981625	0.377133998	up	yes	Protein NR	NPF4.4
Pavir.3KG272800.v	GO:000476	01200(Carb	alanine-glyo	3.661721	26.676192	1.656371	6.2804539	3.8224244	1.934488	0.045360695	0.401486498	up	yes	Serine--glyo	AGT1;AGT1
Pavir.2KG057100.v	NA	NA	0	2.911722	3.947746	1.17501	0.619528	3.8224145	1.9344842	0.04395892	0.396121577	up	yes	NA	NA
Pavir.7KG413900.v	GO:000950	00941(Flav	anthocyanid	2.772012	13.657264	2.402783	1.897262	3.8207219	1.9338452	0.024956657	0.293030147	up	yes	Anthocyani	ANR
Pavir.2NG472500.v	GO:000563	NA	fbd (fbd)	2.521513	4.871994	1.234919	0.701529	3.8180767	1.9328461	0.029480045	0.320687242	up	yes	F-box/FBD	At5g56420
Pavir.3KG127100.v	GO:000367	NA	homeobox p	2.286299	11.493808	2.272976	1.337851	3.8163299	1.9321859	0.024605873	0.289920292	up	yes	Homeobox	OSH71;OSH71;OSH71
Pavir.2NG238800.v	GO:000950	00860(Porp	very-long-c	12.205291	16.373933	4.4370778	3.0570953	3.8135261	1.9311256	0.005449267	0.133532772	up	yes	Short-chain	TIC32;TIC32
Pavir.8KG062900.v	GO:000573	NA	filament-lik	4.8525002	25.526522	4.0784566	3.8879004	3.8134146	1.9310834	0.01837761	0.247552699	up	yes	Filament-li	FPP3;FPP3;FPP3
Pavir.8NG070500.v	GO:000367	00190(Oxid	myb transcr	29.391602	27.739155	2.641557	12.373047	3.8050126	1.9279012	0.018923898	0.251335315	up	yes	Transcriptio	MYB4
Pavir.9NG840600.v	GO:000535	NA	mfs/sugar tr	47.925213	56.121316	13.103208	14.249144	3.8039334	1.927492	0.010472504	0.185692963	up	yes	NA	NA
Pavir.1KG257500.v	GO:000563	04140(Regu	u-box doma	21.341316	34.701096	4.601015	10.133859	3.8033859	1.9272843	0.012128732	0.199698873	up	yes	U-box doma	PUB9
Pavir.1NG016900.v	GO:000367	03010(Ribo	large subun	103.97361	119.00713	18.808882	39.869518	3.8000481	1.9260177	0.011254184	0.192178627	up	yes	Polyubiquit	-
Pavir.5NG498800.v	GO:000449	00905(Bras	cytochrome	12.421229	51.794788	9.7566348	7.1527388	3.7976579	1.92511	0.019933214	0.258492149	up	yes	Cytochrome	CYP72A15;CYP72A15;CYP72A15;CYP72A15
Pavir.8KG171100.v	NA	NA	0	1.09125	2.740834	0.456354	0.552894	3.7969696	1.9248485	0.049221135	0.418413055	up	yes	NA	NA
Pavir.5NG465100.v	GO:000983	00514(Othe	galactosylga	25.339092	16.841425	1.675057	9.443653	3.7936521	1.9235874	0.025111818	0.294208399	up	yes	Probable be	IRX9L
Pavir.6KG249300.v	NA	NA	0	1.090296	1.263557	0.467189	0.153368	3.7931294	1.9233886	0.048163932	0.413516993	up	yes	NA	NA
Pavir.6KG290400.v	GO:000367	00510(N-G	auxin respo	16.632999	5.065181	0.930236	4.800639	3.7861897	1.9207467	0.040036002	0.377133998	up	yes	Auxin-respo	SAUR76
Pavir.5KG492500.v	NA	NA	f-box domai	4.737092	23.156183	3.303255	4.06904	3.7835267	1.9197316	0.017656702	0.243207467	up	yes	NA	NA
Pavir.9KG394200.v	GO:000578	NA	spx domain-	1.162941	2.698099	0.456092	0.564569	3.7828819	1.9194857	0.04618258	0.404762788	up	yes	SPX domai	SPX5
Pavir.9KG022700.v	GO:000373	NA	30s ribosom	5.987192	46.639252	4.610964	9.305022	3.7817259	1.9190448	0.032679134	0.33881156	up	yes	Ribosome-b	PSRP1
Pavir.5NG531100.v	GO:000384	00561(Glyc	lysocardioli	10.073539	14.015837	1.778953	4.591137	3.7816383	1.9190114	0.01641251	0.235419518	up	yes	Probable 1-	LPAT5
Pavir.1NG088900.v	GO:000557	NA	hypothetical	14.120074	18.574432	2.448562	6.215206	3.7737052	1.9159817	0.016263526	0.234215965	up	yes	NA	NA
Pavir.9NG380100.v	GO:000071	NA	deoxyribodi	5.835111	11.874288	2.486513	2.215032	3.766719	1.9133084	0.010577031	0.186577829	up	yes	Deoxyribod	PHR
Pavir.2KG220400.v	GO:001507	NA	osmotic stre	9.4212155	20.358854	1.9919381	5.9302464	3.7590729	1.9103769	0.021396388	0.26932211	up	yes	Potassium t	HAK18;HAK18
Pavir.9NG163400.v	GO:000446	NA	lactoylgluta	2.226167	7.409884	1.204527	1.358982	3.75893	1.9103221	0.043627747	0.394673885	up	yes	NA	NA
Pavir.9NG505500.v	GO:005121	00941(Flav	oxidoreduct	35.989956	20.448845	1.846889	13.183022	3.7550988	1.9088509	0.034707267	0.350364356	up	yes	2-oxoglutar	2ODD11
Pavir.1NG429800.v	GO:000484	NA	inhibitor of	8.247714	33.88623	5.18145	6.040809	3.7544976	1.9086199	0.016016966	0.232455648	up	yes	NA	NA
Pavir.3KG539300.v	GO:000573	00270(Cyst	homocystein	25.903846	75.324703	8.5799996	18.390032	3.7533724	1.9081874	0.023165601	0.281068051	up	yes	Homocystei	HMT-3;HMT-3;HMT-3
Pavir.5KG423600.v	NA	NA	protein of u	5.700219	13.999915	1.212096	4.039519	3.7512525	1.9073724	0.023461348	0.281861447	up	yes	NA	NA
Pavir.4KG267400.v	GO:000588	02010(ABC	atp-binding	27.085686	44.777664	3.226614	15.95197	3.7470623	1.90576	0.035928538	0.356373533	up	yes	ABC transp	ABCG42
Pavir.3KG172600.v	GO:000097	NA	gata transcr	5.6054527	21.599989	1.7897373	5.4712409	3.7468012	1.9056594	0.029933664	0.32320922	up	yes	GATA trans	GATA23;GATA23
Pavir.3KG216000.v	GO:000965	04144(Endo	protein chup	2.022769	6.294709	0.755142	1.46773	3.741771	1.9037213	0.020921321	0.266196007	up	yes	Protein CH	CHUP1
Pavir.7NG355300.v	GO:000400	00230(Puri	adenosine k	8.90522	29.043642	4.867988	5.277936	3.7403062	1.9031564	0.01389382	0.214303781	up	yes	Adenosine l	ADK2
Pavir.3KG280300.v	GO:000404	03040(Splic	peptidyl-trn	1.8923909	3.6138635	0.7931327	0.6816007	3.7337286	1.9006171	0.016497764	0.236051214	up	yes	Peptidyl-tR	Os01g0693900;Os01g0693900;Os01g0693900;Os01g0693900;Os01g0693900
Pavir.5KG512400.v	NA	NA	0	29.791586	49.059586	4.362099	16.758121	3.7334446	1.9005073	0.025407676	0.295687253	up	yes	NA	NA
Pavir.7KG137400.v	GO:000449	00905(Bras	cytochrome	4.345243	16.213739	2.957149	2.551828	3.7319056	1.8999125	0.01544895	0.227874188	up	yes	Cytochrome	CYP72A15
Pavir.9NG261900.v	NA	NA	0	32.307663	84.286331	14.634504	16.61615	3.7309297	1.8995352	0.014904075	0.222837569	up	yes	NA	NA
Pavir.9KG415100.v	GO:000636	NA	transcriptio	2.4242836	6.9858089	0.8898887	1.6376451	3.7230332	1.8964785	0.02460692	0.289920292	up	yes	Probable m	MED26B;MED26B
Pavir.1KG456900.v	NA	NA	0	13.37196	21.184856	1.571677	7.71625	3.7206167	1.8955418	0.024141504	0.286865509	up	yes	NA	NA
Pavir.1NG004300.v	GO:000563	04075(Plan	non-specific	10.194017	15.088751	1.853634	4.942647	3.7200887	1.895337	0.014206361	0.216893787	up	yes	Salt toleran	OsI_16820

Pavir.9NG114400.v4	GO:000097	04712(Circ	transcriptio	1.6988401	4.0212909	0.5477272	0.9917667	3.7155917	1.893592	0.040199506	0.377792907	up	yes	GATA trans	GATA19;GATA19;GATA19;GATA19;GATA19
Pavir.4NG101300.v4	GO:000437	00500(Starc	glycosyltran	8.471347	16.348989	1.795321	4.900989	3.7065691	1.8900844	0.014588621	0.219883759	up	yes	Soluble star	SSII-3
Pavir.3KG152200.v4	GO:000377	NA	advillin (avi	23.750433	16.379133	3.824761	7.005072	3.7054649	1.8896546	0.010143658	0.182343582	up	yes	Villin-1 OS	VLN1
Pavir.3NG172300.v4	GO:001602	00591(Linc	premnaspiro	2.0044	13.102142	1.32928	2.75134	3.7020213	1.8883132	0.038593821	0.37029766	up	yes	Tryptamine	CYP71P1
Pavir.1NG440400.v4	GO:000552	04144(Endo	adp-ribosyla	1.8095925	2.7783417	0.444716	0.7957207	3.6986444	1.8869966	0.038021429	0.367149613	up	yes	ADP-ribosy	Os01g0813400;Os01g0813400;Os01g0813400
Pavir.5NG356800.v4	GO:000962	04626(Plan	erebp-like fa	114.33077	256.30266	38.704903	61.545446	3.6970787	1.8863858	0.02121491	0.268025488	up	yes	Ethylene-res	EREBP1;EREBP1
Pavir.1KG521300.v4	NA	NA	0	11.861553	39.893578	5.509802	8.513582	3.6906307	1.8838674	0.021291484	0.268614363	up	yes	NA	NA
Pavir.2NG004900.v4	GO:000497	NA	ionotropic g	5.900046	7.1944473	0.9135353	2.6359311	3.6891442	1.8832862	0.013847517	0.214024172	up	yes	Glutamate r	GLR3.4;GLR3.5;GLR3.4
Pavir.9KG392700.v4	GO:000367	00970(Ami	transcriptio	57.721176	93.857399	13.643749	27.448236	3.6887625	1.8831369	0.016605333	0.236699774	up	yes	Nuclear trar	NFYA5
Pavir.4KG205800.v4	GO:000399	00620(Pyru	acylphospha	13.255145	35.71957	9.151678	4.131351	3.6870141	1.8824529	0.026315682	0.302465322	up	yes	NA	NA
Pavir.4KG274700.v4	GO:000367	NA	n-myc dowr	11.624955	31.047009	4.657759	6.918662	3.6861102	1.8820992	0.011831076	0.197160934	up	yes	Protein ND	NDL2
Pavir.2NG397500.v4	GO:000367	04712(Circ	dof domain,	18.472214	10.140713	2.381698	5.383775	3.6846341	1.8815214	0.013059327	0.207826386	up	yes	Dof zinc fin	MNB1A
Pavir.5NG275100.v4	GO:000470	04075(Plan	mitogen-act	71.981548	169.61054	37.265191	28.440486	3.6768831	1.8784833	0.019146441	0.253395296	up	yes	Mitogen-act	MPK10;MPK10;MPK10
Pavir.1NG141600.v4	NA	NA	0	2.3822423	6.3384656	1.0138918	1.358033	3.6766377	1.878387	0.013937167	0.214437719	up	yes	NA	NA
Pavir.2NG041900.v4	GO:000367	NA	putative ribo	2.356226	2.686474	0.575866	0.797087	3.6728861	1.8769142	0.031008951	0.329496409	up	yes	NA	NA
Pavir.2NG043000.v4	NA	NA	0	1.760848	7.317077	0.967904	1.504484	3.6717235	1.8764574	0.04263379	0.389038382	up	yes	NA	NA
Pavir.7NG189600.v4	GO:000395	00592(alph	nadh oxidor	3.517516	3.585929	0.519354	1.415731	3.6708698	1.8761219	0.013371662	0.209600947	up	yes	Retrovirus-r	RE1
Pavir.5KG504700.v4	NA	NA	0	96.324165	180.43071	19.590477	55.852997	3.668374	1.8751407	0.027113492	0.30666322	up	yes	Acyl transfe	AT7
Pavir.1NG558300.v4	GO:000467	04626(Plan	abc1 protein	4.146212	7.4146137	1.3607	1.791117	3.6679876	1.8749888	0.010031401	0.181875162	up	yes	Protein AC	ABC1K3;ABC1K3
Pavir.7NG386400.v4	GO:000001	NA	endonucleas	12.867746	4.504153	0.793477	3.948315	3.6635725	1.8732512	0.040787485	0.380028688	up	yes	Endonuclea	ENDO1
Pavir.2NG322800.v4	NA	NA	0	2.56062	1.96075	0.370357	0.863944	3.6631016	1.8730657	0.04062356	0.379595456	up	yes	NA	NA
Pavir.1KG386300.v4	GO:000551	00360(Pher	phenylalanin	225.20692	223.51479	23.338507	99.290672	3.6591757	1.8715187	0.035271428	0.35344312	up	yes	Phenylalani	ZB8
Pavir.8NG237800.v4	GO:000521	NA	protein nrt1.	6.3131331	13.484661	2.8508003	2.5646533	3.6557962	1.8701856	0.008999243	0.173354197	up	yes	Protein NR	NPF5.10;NPF5.10;NPF5.10
Pavir.2NG504400.v4	GO:000573	NA	filament-lik	15.129168	55.947124	4.6627258	14.800187	3.6518837	1.8686408	0.03923866	0.37326534	up	yes	Filament-lik	FPP3;FPP3;FPP3;FPP;FPP
Pavir.1KG454900.v4	NA	NA	0	2.070642	6.260846	1.234296	1.048318	3.6499767	1.8678873	0.042341078	0.388019839	up	yes	NA	NA
Pavir.7KG261600.v4	NA	NA	0	62.41946	233.21547	34.938374	46.13377	3.6465661	1.8665385	0.019466551	0.255789208	up	yes	Late embryo	At5g17165
Pavir.1KG546200.v4	GO:000467	04626(Plan	protein kina	4.19575	14.880562	2.913618	2.317889	3.6464277	1.8664838	0.016588086	0.23662268	up	yes	Serine/threo	PCRK1
Pavir.7NG183100.v4	GO:000467	04712(Circ	cryptochron	7.2678206	29.380374	4.0148586	6.0363979	3.6461307	1.8663663	0.026053252	0.300253192	up	yes	Cryptochro	CRY1;CRY1;CRY1;CRY1;CRY1
Pavir.2KG065200.v4	GO:000563	NA	glutaredoxin	16.965807	12.904084	2.643012	5.555532	3.6433165	1.8652523	0.012446643	0.202624664	up	yes	Uncharacter	At5g39865
Pavir.9NG640400.v4	NA	NA	0	11.347679	30.616243	3.659602	7.865862	3.6409746	1.8643247	0.024102051	0.286668685	up	yes	NA	NA
Pavir.2NG621800.v4	GO:000563	NA	ring finger p	16.682156	34.135497	3.8067292	10.182644	3.6325897	1.8609984	0.016431616	0.235463425	up	yes	E3 ubiquitin	AIRP2;AIRP2
Pavir.5KG494200.v4	NA	NA	f-box domai	5.648014	27.605255	4.195776	4.959133	3.6322883	1.8608787	0.02065652	0.26398892	up	yes	NA	NA
Pavir.7NG309600.v4	GO:000497	NA	ionotropic g	3.295072	5.253346	0.869883	1.484545	3.6307834	1.8602808	0.034265455	0.347210345	up	yes	Glutamate r	GLR3.1
Pavir.7KG142600.v4	NA	NA	0	2.662656	6.489474	1.426465	1.095481	3.6289952	1.8595702	0.031590148	0.332754923	up	yes	Probable F-	At2g36090
Pavir.5KG760800.v4	GO:000473	01230(Bios	pyrroline-5-	27.147686	37.510826	7.303998	10.531636	3.6252433	1.8580778	0.008044659	0.162733919	up	yes	Pyrroline-5-	-
Pavir.3NG256500.v4	GO:000370	04712(Circ	cct motif (co	9.045647	20.393967	3.097269	5.036227	3.6195523	1.8558113	0.01517186	0.225673911	up	yes	Zinc finger	COL16
Pavir.8KG149000.v4	GO:000467	04075(Plan	non-specific	12.247667	21.126091	2.686568	6.546999	3.614395	1.8537542	0.016402437	0.235404413	up	yes	Receptor pr	CEPR2
Pavir.8KG026700.v4	GO:000582	01200(Carb	dbj	4.531044	14.983403	2.510123	2.88932	3.6141593	1.8536601	0.017270666	0.241363679	up	yes	2-carboxy-I	-
Pavir.5KG758600.v4	NA	NA	0	7.808431	19.652861	4.140426	3.472643	3.6071251	1.8508494	0.014823184	0.221981462	up	yes	NA	NA
Pavir.8KG088600.v4	NA	NA	0	7.034224	16.408379	0.880107	5.619159	3.6069616	1.8507841	0.048734447	0.416253551	up	yes	NA	NA
Pavir.2KG051100.v4	GO:000950	00195(Phot	photosysten	155.50557	470.58633	62.949821	110.76821	3.6040697	1.8496269	0.0264192	0.30297155	up	yes	Photosysten	PSAK
Pavir.6NG098300.v4	GO:000538	NA	zinc/iron tra	2.048736	6.75464	0.725132	1.717639	3.6038483	1.8495383	0.043492254	0.393672478	up	yes	Zinc transpo	ZIP4
Pavir.1NG260700.v4	GO:000575	NA	iron-sulfur c	4.322852	17.43532	1.954406	4.083452	3.6036243	1.8494486	0.038419246	0.369566389	up	yes	Iron-sulfur c	ISU1
Pavir.7NG000200.v4	NA	NA	0	61.532078	63.675999	4.653937	30.106514	3.6020268	1.8488089	0.038037047	0.367206051	up	yes	NA	NA
Pavir.7KG262900.v4	GO:000467	00500(Starc	protein kina	3.616588	2.889549	0.302737	1.504789	3.5994708	1.8477848	0.048219302	0.413734608	up	yes	L-type lectin	SIT2
Pavir.7KG110900.v4	NA	NA	0	206.90105	241.85463	29.821869	95.130188	3.5914229	1.8445555	0.027250927	0.307297815	up	yes	Protein EG(	Ea1
Pavir.2NG410400.v4	GO:000588	NA	predicted m	267.26136	292.67626	35.791606	120.13687	3.5909901	1.8443817	0.03148925	0.331971265	up	yes	Cytochrome	At3g25290;At3g25290
Pavir.7KG432600.v4	GO:000387	01230(Bios	5-methyltetr	2.535645	6.20048	0.9464	1.4879	3.5887627	1.8434865	0.025377392	0.295477148	up	yes	5-methyltetr	Os12g0623900
Pavir.8NG296100.v4	GO:000370	04075(Plan	gras domain	1.897287	3.132154	0.383591	1.018484	3.5871412	1.8428345	0.046483275	0.40582121	up	yes	Scarecrow-l	SCL9
Pavir.3KG551100.v4	GO:000367	00230(Purin	transcriptio	4.8140193	7.6549101	1.5588577	1.9222062	3.5819306	1.8407374	0.01406077	0.215458932	up	yes	Nuclear trar	NFYA10;NFYA10;NFYA10
Pavir.6NG022200.v4	GO:000333	04144(Endo	lysine histid	1.904876	10.644609	1.533952	1.970284	3.5812328	1.8404563	0.034412042	0.348319953	up	yes	Lysine histi	LHT1
Pavir.9NG738300.v4	GO:000372	03013(RNA	rna helicase	1.02208	1.491049	0.444764	0.257529	3.5784623	1.8393398	0.045988768	0.40438474	up	yes	Probable RI	SDE3
Pavir.5NG457800.v4	GO:000367	03040(Splic	protein of ur	3.83606	21.138414	4.518282	2.465048	3.5762987	1.8384672	0.031153162	0.330075502	up	yes	NA	NA
Pavir.5NG385600.v4	GO:000485	00380(Tryp	indole-3-ace	1.992156	11.372187	1.964466	1.779868	3.5692176	1.8356079	0.024048621	0.286371335	up	yes	Indole-3-ac	AO2;AO2
Pavir.9NG154000.v4	GO:000588	00190(Oxid	h+-transpor	6.462621	19.367571	3.764593	3.473874	3.5684617	1.8353023	0.01412031	0.2160189	up	yes	Plasma mer	LHA1
Pavir.5KG097700.v4	GO:000367	04075(Plan	ap2 domain	3.965801	4.601832	1.312255	1.088822	3.5682458	1.835215	0.024244843	0.2873664	up	yes	AP2/ERF a	Os01g0141000
Pavir.9NG797800.v4	GO:000367	00620(Pyru	plant protein	9.69055	21.576862	1.552416	7.221487	3.5636833	1.8333692	0.029842081	0.322663744	up	yes	NA	NA
Pavir.6KG365500.v4	NA	NA	0	9.261553	17.432611	1.709757	5.782761	3.5627761	1.8330018	0.029676636	0.321800941	up	yes	NA	NA
Pavir.5KG557800.v4	GO:000016	01230(Bios	chorismate r	87.864471	115.59578	18.301765	38.892868	3.5573312	1.8307953	0.022108614	0.274171833	up	yes	Chorismate	CM1
Pavir.1NG323900.v4	GO:000646	04626(Plan	serine/threo	59.38901	104.18781	9.8981724	36.152349	3.5521166	1.8286789	0.035461422	0.3540943	up	yes	Serine/threo	NEK6;NEK6
Pavir.9NG437700.v4	GO:000367	NA	dnaj (hsp40	3.716538	16.759106	3.833334	1.935482	3.5493668	1.8275617	0.029196848	0.319204446	up	yes	Transcriptio	DIVARICATA
Pavir.5NG510200.v4	GO:000455	00230(Purin	x8 domain (	24.695761	17.281355	4.728166	7.099135	3.5491712	1.8274822	0.017054124	0.240475103	up	yes	PLASMOD	PDCB2
Pavir.9KG653200.v4	GO:000367	00620(Pyru	plant protein	13.404465	37.195356	2.3959802	11.887351	3.5425784	1.8247998	0.045909463	0.403876435	up	yes	NA	NA
Pavir.2NG601600.v4	NA	NA	0	4.6780254	11.513003	1.9418366	2.6286647	3.542506	1.8247703	0.032396832	0.337280734	up	yes	NA	NA
Pavir.5NG291800.v4	NA	NA	0	23.100409	37.701618	3.966604	13.240008	3.5336432	1.8211564	0.027828588	0.310001237	up	yes	NA	NA
Pavir.9KG645000.v4	GO:000107	00100(Stero	calmodulin-	5.0495219	12.227833	1.4793395	3.4119498	3.5322701	1.8205957	0.01803745	0.245284518	up	yes	Calmodulin	CAMTA3;CAMTA3;CAMTA3;CAMTA3
Pavir.5NG497800.v4	GO:000370	04626(Plan	wrky transcr	9.980268	65.043457	9.718029	11.549543	3.5276112	1.8186916	0.038368742	0.369175097	up	yes	Probable W	WRKY69
Pavir.1NG548000.v4	NA	NA	0	16.326143	6.151007	1.190691	5.183635	3.5262003	1.8181144	0.040157029	0.377598979	up	yes	NA	NA
Pavir.9NG149600.v4	NA	NA	0	10.22209	4.881462	1.208247	3.077937	3.5237759	1.8171222	0.043106069	0.39157121	up	yes	NA	NA
Pavir.7NG431800.v4	GO:000367	NA	f12f1.11-rel	9.8865689	17.590102	3.7007207	4.0984042	3.5230454	1.8168231	0.010111376	0.182213283	up	yes	NA	NA
Pavir.9KG411200.v4	GO:000449	00905(Bras	cytochrome	41.62928	122.28765	13.941237	32.596878	3.5222082	1.8164802	0.034334417	0.347721576	up	yes	Cytochrome	CYP709B2
Pavir.4KG317200.v4	GO:000588	NA	domain of u	4.872002	4.823695	0.7781	1.976537	3.519773	1.8154824	0.020149864	0.260079294	up	yes	MAPK kina	At1g80180
Pavir.1NG084200.v4	NA	NA	0	22.677679	38.864529	3.681115	13.806652	3.5191576	1.8152301	0.023574641	0.282722626	up	yes	NA	NA
Pavir.2NG407300.v4	GO:000557	NA	bactericidal	7.011908	8.043544	1.436565	2.842378	3.5184979	1.8149597	0.018386325	0.247552699	up	yes	NA	NA
Pavir.2KG050900.v4	NA	NA	toll-like rec	15.673885	44.187849	4.6018397	12.413385	3.5181278	1.8148079	0.036916214	0.361871356	up	yes	187-kDa mi	AIR9;AIR9;AIR9;AIR9;AIR9;AIR9;AIR9;AIR9;AIR9;AIR9
Pavir.3KG404200.v4	GO:000027	NA	udp-glucose	13.12652	46.927853	2.729973	14.344059	3.517293	1.8144655	0.045666728	0.403062241	up	yes	Chloroplast	CSP41B
Pavir.3KG329100.v4	NA	NA	0	16.097401	31.442179	5.404606	8.112285	3.5170499	1.8143658	0.01166528	0.195931866	up	yes	NA	NA
Pavir.9NG206600.v4	GO:005121	00904(Dite	oxidoreduct	7.166399	8.128977	1.328	3.025563	3.5133007	1.8128271	0.02163159	0.270740748	up	yes	Probable 2-	At5g05600
Pavir.9NG771600.v4	GO:000573	NA	heavy-metal	34.105587	138.97791	10.834179	38.461983	3.5110947	1.8119209	0.04986907	0.421726439	up	yes	Heavy meta	HIPP39
Pavir.2KG034600.v4	NA	NA	toll-like rec	14.69497	40.662589	4.2327626	11.533723	3.5110906	1.8119192	0.036713742	0.36052721	up	yes	187-kDa mi	AIR9;AIR9;AIR9;AIR9;AIR9;AIR9;AIR9;AIR9;AIR9
Pavir.6NG122000.v4	GO:000563	NA	zinc-finger c	14.868938	9.643835	3.296484	3.69693	3.5051225	1.8094649	0.035213143	0.353099391	up	yes	FCS-Like Z	FLZ6
Pavir.5NG110100.v4	GO:000563	01200(Carb	fructose-1,6	15.166879	28.071251	3.264314	9.082182	3.5020568	1.8082025	0.022939142	0.279744358	up	yes	Fructose-1,(	CFBP

Pavir.1KG416200.v4	GO:000007	01200(Carb	cyclin	17.649878	20.922194	2.227027	8.803679	3.4967909	1.8060315	0.02388769	0.285564774	up	yes	Cyclin-P4-1	CYCP4-1
Pavir.2KG018400.v4	GO:00095(	NA	prestalk a d	18.003025	13.460572	1.818756	7.196001	3.4902324	1.8033231	0.024171399	0.286877253	up	yes	Protein TIC	TIC62
Pavir.4KG250400.v4	GO:00058	NA	ring finger c	8.638166	5.585898	1.422644	2.657912	3.4858152	1.8014961	0.021083863	0.267176726	up	yes	E3 ubiquiti	Os06g0535400
Pavir.8KG218200.v4	GO:00057	NA	polyketide c	5.9512199	12.035958	2.0245885	3.1420415	3.481414	1.7996734	0.012881896	0.206401489	up	yes	NA	NA
Pavir.6NG020500.v4	GO:00095(	00195(Phot	photosysten	5.611708	16.018522	2.609937	3.607606	3.4789032	1.7986325	0.016157179	0.233131218	up	yes	Photosysten	PSBY
Pavir.2KG097400.v4	GO:00058	NA	uncharacteri	6.503391	8.276045	1.215296	3.034032	3.4780643	1.7982846	0.017137401	0.240690464	up	yes	Protein NU	NFD4
Pavir.5KG110000.v4	NA	NA	0	15.413959	30.536651	4.3416291	8.8845737	3.4742103	1.7966851	0.019551485	0.256267806	up	yes	NA	NA
Pavir.6NG305800.v4	GO:00058	NA	eukaryotic c	11.459715	9.387975	2.795878	3.210546	3.4708988	1.7953093	0.008873565	0.172303086	up	yes	Cytochrome	At3g25290
Pavir.4KG352800.v4	GO:000367	NA	c20orf30 pr	18.640829	21.482637	3.447056	8.122299	3.4680815	1.7941378	0.019623216	0.256274967	up	yes	NA	NA
Pavir.4KG157300.v4	NA	NA	0	9.6383846	16.416901	3.2430782	4.2701921	3.4679021	1.7940632	0.011262804	0.192178627	up	yes	NA	NA
Pavir.3KG266700.v4	GO:00057	00941(Flav	(-)-pinoresi	35.907991	103.30245	9.455347	30.714779	3.4655217	1.7930726	0.039156348	0.373071272	up	yes	Isoflavone r	-;-
Pavir.9NG737400.v4	NA	NA	0	24.586981	40.289917	8.541914	10.184216	3.4645118	1.7926521	0.010233809	0.183539342	up	yes	NA	NA
Pavir.2NG478400.v4	NA	NA	0	9.544845	30.541204	4.821579	6.751176	3.4638294	1.7923679	0.020212769	0.260622638	up	yes	Uncharacter	At4g14100
Pavir.9NG179000.v4	GO:000387	01230(Bios	5-methyltetr	607.75208	1365.3339	140.70508	429.43762	3.4606879	1.7910588	0.040921764	0.380527563	up	yes	5-methyltet	Os12g0623900
Pavir.3KG344700.v4	GO:000577	00790(Fola	gamma-glut	2.3255648	2.6678776	0.704837	0.7384868	3.4596826	1.7906397	0.049523392	0.420316782	up	yes	Gamma-glu	GGH2;GGH2
Pavir.2NG088000.v4	NA	NA	0	5.6022616	21.90372	2.590081	5.36527	3.4575447	1.7897479	0.027536804	0.308757967	up	yes	NA	NA
Pavir.7NG438800.v4	NA	NA	domain of u	35.245811	64.179977	9.370694	19.389578	3.4570531	1.7895428	0.023720695	0.284202069	up	yes	NA	NA
Pavir.9NG591400.v4	NA	NA	0	6.291427	16.060926	2.414622	4.056232	3.4543127	1.7883987	0.02516371	0.294261858	up	yes	NA	NA
Pavir.1NG309300.v4	GO:000487	NA	root phototr	12.067952	12.087626	2.915753	4.07865	3.4535582	1.7880835	0.009808556	0.18065533	up	yes	Coleoptile p	CPT1
Pavir.3NG064000.v4	NA	NA	0	5.244493	17.973808	3.80305	2.922435	3.4522865	1.7875522	0.028483523	0.313569126	up	yes	NA	NA
Pavir.5KG745200.v4	GO:00002(	03010(Ribo	ring zinc fin	14.100603	16.253394	3.425133	5.3709941	3.4508366	1.7869461	0.010508619	0.185982266	up	yes	E3 ubiquiti	At3g02290;At3g02290
Pavir.4KG200200.v4	GO:00058	NA	aquaporin t	25.995974	43.48539	3.208835	16.931255	3.4499034	1.7865559	0.037264956	0.363533126	up	yes	Aquaporin	NIP2-2
Pavir.8NG197900.v4	GO:00001(	NA	lupus la pro	18.4383	33.676494	8.6997795	6.4103186	3.4490043	1.7861799	0.01130952	0.192538565	up	yes	La-related p	LARP6B;LARP6B
Pavir.1KG478700.v4	NA	NA	0	6.54777	19.459419	3.998139	3.543954	3.4482721	1.7858736	0.015670151	0.229631275	up	yes	NA	NA
Pavir.2KG056700.v4	GO:000451	NA	hnh endonu	3.4019844	8.6191742	1.076006	2.4108478	3.447566	1.7855782	0.043124629	0.39157121	up	yes	NA	NA
Pavir.2NG070100.v4	GO:00095(	NA	protein seed	6.765936	14.104105	2.681656	3.393135	3.4355159	1.7805268	0.019470288	0.255789208	up	yes	Protein SEI	SPD1
Pavir.9NG016300.v4	NA	NA	0	15.043946	46.299854	4.67523	13.182308	3.4351768	1.7803843	0.028265039	0.312498409	up	yes	PGR5-like	PGRL1B
Pavir.1NG060600.v4	GO:000467	04075(Plan	non-specific	3.10872	3.091192	0.599621	1.208353	3.4292042	1.7778738	0.035590683	0.354896162	up	yes	Cold-respor	CRPK1
Pavir.5NG559700.v4	NA	NA	0	1.875795	12.874355	2.66572	1.636108	3.4288098	1.7777079	0.039240894	0.37326534	up	yes	NA	NA
Pavir.6KG048300.v4	GO:000367	NA	n-myc dowr	15.340894	32.411518	3.9165777	10.017564	3.4270077	1.7769494	0.020476592	0.262666409	up	yes	Protein ND	NDL2;NDL1
Pavir.2KG587300.v4	GO:000367	NA	b3 domain-	27.738855	61.956415	7.5962255	18.597348	3.4243236	1.775819	0.034158719	0.346222163	up	yes	B3 domain-	Os07g0679700;Os07g0679700;Os07g0679700
Pavir.4KG196700.v4	GO:00037(	NA	protein tag-	5.06566	14.359634	2.26303	3.410818	3.4236543	1.775537	0.017504274	0.242738038	up	yes	LIM domai	WLIM1
Pavir.2NG280400.v4	NA	NA	0	5.179572	6.303226	1.273338	2.081858	3.4223926	1.7750053	0.01844417	0.24776647	up	yes	NA	NA
Pavir.9KG604800.v4	GO:000367	NA	gb	26.617517	14.572825	5.023527	7.051237	3.4112751	1.7703111	0.012599925	0.204071389	up	yes	NA	NA
Pavir.5KG634100.v4	NA	NA	0	37.853569	72.486004	10.917867	21.432627	3.4107539	1.7700906	0.026381052	0.302846097	up	yes	NA	NA
Pavir.7KG376800.v4	NA	NA	f-box and le	11.999367	27.646069	3.788119	7.863853	3.4024658	1.7665807	0.01926992	0.25458112	up	yes	NA	NA
Pavir.5KG512600.v4	GO:00058	02010(ABC	abc transpo	4.021368	7.215364	1.36847	1.936375	3.4000784	1.765568	0.014711392	0.220835453	up	yes	ABC transp	ABCG14
Pavir.9NG247900.v4	GO:000563	04626(Plan	interleukin-	37.668068	73.148117	6.643887	25.954506	3.3994371	1.7652959	0.041076849	0.381335448	up	yes	Salt toleran	OsI_16820
Pavir.3KG204100.v4	GO:00041	NA	aspartyl pro	5.185431	35.816116	6.358345	5.712142	3.3968428	1.7641945	0.034695097	0.350364356	up	yes	Aspartic pr	RAP
Pavir.6NG366300.v4	GO:000551	NA	protein prot	23.168217	46.979984	6.931306	13.724036	3.3961288	1.7638912	0.017310002	0.24160056	up	yes	Protein PR	PGR5
Pavir.9KG483000.v4	NA	NA	0	12.241755	28.200542	4.531207	7.381773	3.3948094	1.7633306	0.016376414	0.235402849	up	yes	NA	NA
Pavir.9KG517100.v4	GO:000461	01200(Carb	phosphoeno	8.899019	61.583075	13.109494	7.674692	3.3911404	1.7617705	0.046487569	0.40582121	up	yes	Phosphoen	-;-
Pavir.9NG214700.v4	GO:00002(	03010(Ribo	ring zinc fin	6.390425	5.5432352	1.039852	2.480066	3.3903233	1.7614229	0.022110043	0.274171833	up	yes	E3 ubiquiti	At3g02290;At3g02290;At3g02290
Pavir.4KG274500.v4	GO:00003	03040(Spli	pre-mrna-pr	6.0713556	11.534273	1.9572178	3.2376081	3.3890701	1.7608895	0.017464854	0.242590372	up	yes	Pre-mRNA-	OsI_27673;OsI_27673;OsI_27673
Pavir.8NG275500.v4	GO:000981	03013(RNA	udp-glycosy	5.840309	17.685837	3.34999	3.596329	3.3868508	1.7599444	0.019883842	0.258492149	up	yes	Hydroquino	AS
Pavir.1NG370300.v4	GO:00051	NA	vesicle-asso	11.164967	19.455341	1.932793	7.113636	3.384795	1.7590685	0.029670589	0.321800941	up	yes	Vesicle-ass	PVA12
Pavir.4NG094300.v4	GO:000467	00520(Ami	non-specific	3.3985793	7.4984425	1.013745	2.209559	3.3806994	1.7573217	0.024594419	0.289920292	up	yes	Cysteine-ric	CRK10;CRK10
Pavir.5KG765100.v4	GO:00045	00052(Gala	beta-galacto	8.874178	15.57216	3.455694	3.791169	3.3733683	1.7541898	0.018368164	0.247552699	up	yes	NA	NA
Pavir.7NG238000.v4	GO:000467	04075(Plan	protein kina	1.086122	2.729621	0.533443	0.598022	3.3723915	1.753772	0.04525738	0.401050832	up	yes	Probably in	At5g06940
Pavir.1KG092800.v4	GO:00040(	NA	arylformami	3.184196	5.080983	1.841389	0.609581	3.3722073	1.7536932	0.043118932	0.39157121	up	yes	Cyclase-lik	CYL1
Pavir.1NG461600.v4	NA	NA	0	30.690666	40.192066	5.874683	15.149906	3.3714206	1.7533566	0.023124135	0.280992432	up	yes	NA	NA
Pavir.5KG256700.v4	GO:000552	00561(Glyc	bcs1 aaa-ty	3.0397024	3.6296459	0.5054264	1.47436	3.3687212	1.752201	0.042637099	0.389038382	up	yes	AAA-ATPa	At3g28610;At3g28610
Pavir.6KG133900.v4	NA	NA	0	10.917583	19.800673	3.123031	5.999109	3.3674397	1.7516521	0.022243382	0.27450782	up	yes	NA	NA
Pavir.2KG485400.v4	GO:00053	NA	solute carrie	8.154345	33.165816	6.6249239	5.6462071	3.3672659	1.7515776	0.030413238	0.325931716	up	yes	Equilibrativ	ENT3;ENT3;ENT3;ENT3;ENT3;ENT3;ENT3;ENT3;ENT3;ENT3
Pavir.6KG297500.v4	NA	NA	0	20.799025	79.384712	7.085479	22.672991	3.3665621	1.7512761	0.047781272	0.412140897	up	yes	NA	NA
Pavir.3NG304000.v4	GO:001602	00903(Lim	cytochrome	22.705786	30.157864	6.041859	9.671327	3.3642859	1.7503003	0.012652622	0.204359507	up	yes	Cytochrome	CYP81Q32
Pavir.5KG745500.v4	GO:00001	00790(Fola	beta-1,4-xyl	182.48604	94.872429	25.668877	56.778385	3.3640713	1.7502083	0.032489327	0.337682291	up	yes	Probable gl	Os01g0926600
Pavir.8KG012100.v4	GO:000367	03008(Ribo	5'-3' exoribo	19.87645	55.226102	12.098698	10.240863	3.3618634	1.7492611	0.026959082	0.30605746	up	yes	5'-3' exorib	XRN3;XRN3
Pavir.8NG046300.v4	GO:00037(	04141(Prot	transcriptio	13.633278	32.662128	5.202266	8.586562	3.3574576	1.7473692	0.017961265	0.244868865	up	yes	Transcriptic	VIP1
Pavir.4KG325600.v4	GO:00001	NA	solute carrie	5.449758	21.31365	4.947004	3.034128	3.3533348	1.7455965	0.023059676	0.280667701	up	yes	GDP-mann	GONST3
Pavir.4NG161900.v4	GO:000402	00010(Glyc	aldehyde de	11.184985	45.397316	6.956715	9.927993	3.3510974	1.7446336	0.034888389	0.35134319	up	yes	Aldehyde d	ALDH2B7
Pavir.9NG464700.v4	GO:00037(	00750(Vita	hd-zip prote	4.290034	5.750024	2.164662	0.831746	3.3506979	1.7444616	0.031713883	0.333684044	up	yes	Homeobox-	HOX1
Pavir.4KG115800.v4	GO:00302	NA	peptidase s	4.721016	11.275956	2.530221	2.244495	3.3503505	1.744312	0.017094163	0.240475103	up	yes	Carboxyl-te	CTPA3
Pavir.2KG251400.v4	GO:000367	NA	nucleotide-c	14.69188	14.49652	3.1158056	5.6004653	3.3487257	1.7436122	0.013312118	0.209366345	up	yes	Uncharacter	At4g15970;At4g15970
Pavir.6KG089100.v4	GO:00069	04140(Reg	autophagy-r	57.464798	266.81628	39.301071	57.586842	3.3469715	1.7428563	0.043289148	0.39250673	up	yes	Autophagy-	ATG8C
Pavir.5NG558200.v4	GO:00069	03013(RNA	lurp-one-rel	22.667797	5.518146	2.930418	5.491879	3.3465862	1.7426902	0.037469574	0.364237061	up	yes	Protein LU	At2g38640
Pavir.1NG083600.v4	GO:00098(	00940(Phe	cinnamyl al	159.77209	268.78331	32.772857	95.724492	3.3351303	1.7377431	0.039623536	0.375475482	up	yes	Probable ci	CAD;CAD
Pavir.2NG619000.v4	GO:000374	03013(RNA	elongation f	1.734974	2.538387	0.636101	0.647567	3.3290235	1.7350991	0.048184506	0.413530478	up	yes	Elongation	REFA1
Pavir.6KG383300.v4	NA	NA	0	11.395157	28.069513	6.004084	5.857191	3.3271862	1.7343026	0.017358668	0.24174101	up	yes	NA	NA
Pavir.1NG073000.v4	GO:000573	00040(Pent	ribulokinase	8.465506	15.461264	2.727182	4.471241	3.3238905	1.7328729	0.015560064	0.228731728	up	yes	NA	NA
Pavir.7NG350700.v4	GO:000842	00500(Star	glycosyl hy	12.397119	41.181084	2.855079	13.270651	3.3225288	1.7322817	0.046931691	0.407997326	up	yes	Probable in	BGLU14
Pavir.9KG509600.v4	GO:000367	NA	protein of u	5.834483	20.488247	2.850629	5.078691	3.3196705	1.7310401	0.036762078	0.360736895	up	yes	NA	NA
Pavir.4KG319900.v4	NA	NA	0	4.021186	12.423209	1.640376	3.314523	3.3188154	1.7306684	0.039376715	0.374273034	up	yes	NA	NA
Pavir.5NG478400.v4	GO:00055(	NA	dnaj homol	3.286795	2.927282	0.710195	1.164287	3.3150902	1.7290481	0.032866829	0.339718219	up	yes	NA	NA
Pavir.5KG255600.v4	NA	NA	0	41.335064	57.983765	11.123638	18.859421	3.3124982	1.7279197	0.022637051	0.277230089	up	yes	NA	NA
Pavir.4NG255400.v4	NA	NA	0	3.638456	7.383915	1.145873	2.184317	3.3098325	1.7267582	0.039284331	0.373489416	up	yes	NA	NA
Pavir.3NG178800.v4	GO:000552	00860(Porp	magnesium	24.204836	45.982502	7.369687	13.839116	3.3093493	1.7265476	0.0179735	0.244919876	up	yes	Magnesium	CHLH
Pavir.3KG268700.v4	NA	NA	0	3.7997965	10.79376	0.7902673	3.627605	3.3032997	1.7239079	0.046220783	0.404908717	up	yes	NA	NA
Pavir.5KG626500.v4	GO:000403	00040(Pent	aldo/keto re	3.980957	6.193596	1.606284	1.474727	3.3023423	1.7234897	0.034031452	0.345491424	up	yes	NADPH-de	AKR4C9
Pavir.2KG203900.v4	GO:000557	NA	tetraspanin-	44.261906	116.25839	16.43051	32.204258	3.3005256	1.7226958	0.03475158	0.350435081	up	yes	Tetraspanin	TET4
Pavir.6NG012700.v4	GO:000467	04626(Plan	abc1 family	2.682595	3.51339	0.631536	1.246237	3.2996454	1.722311	0.027866268	0.310144113	up	yes	Serine/threc	CCR4
Pavir.1KG355900.v4	NA	NA	0	4.103892	23.279133	2.758244	5.542509	3.2988604	1.7219677	0.045157286	0.400541715	up	yes	NA	NA

Pavir.9KG581700.v	GO:000521	NA	oligopeptid	15.28847	7.331007	2.062378	4.79796	3.2971374	1.721214	0.023270822	0.281238111	up	yes	Protein NR	NPF5.2
Pavir.9NG544700.v	GO:000419	NA	metacaspas	5.053263	6.933774	0.92637	2.716122	3.290889	1.7184774	0.041300678	0.382442838	up	yes	Metacaspas	AMC1
Pavir.5KG219200.v	GO:000561	04144(End	cupin doma	29.859667	13.432952	3.858042	9.303785	3.289256	1.7177613	0.024195514	0.286962768	up	yes	Germin-like	GER4
Pavir.3KG570000.v	GO:000367	NA	bel1-like ho	31.890261	42.566898	4.009527	18.635778	3.2879733	1.7171986	0.048937098	0.416966544	up	yes	BEL1-like l	BLH7
Pavir.2NG122400.v	GO:000166	00561(Glyc	no apical m	7.092981	7.485113	2.019943	2.41408	3.2877804	1.7171139	0.025612322	0.296558329	up	yes	NAC doma	NAC067
Pavir.9KG597000.v	GO:000563	NA	ovate family	7.489791	7.197607	1.785167	2.688802	3.2828565	1.7149517	0.046657981	0.406501837	up	yes	Transcriptic	OFP11
Pavir.3KG122300.v	NA	NA	0	70.256973	47.267155	10.464635	25.349241	3.281525	1.7143664	0.020591358	0.26342495	up	yes	NA	NA
Pavir.5KG536300.v	GO:000370	04626(Plan	wrky transc	14.050681	49.671383	9.36406	10.068396	3.2791565	1.7133247	0.030115547	0.324406954	up	yes	Probable W	WRKY69
Pavir.4KG390000.v	GO:000367	04144(End	regulator of	4.2828702	12.920471	1.9992928	3.2517092	3.2762017	1.7120242	0.023763255	0.284582003	up	yes	NA	NA
Pavir.2NG604100.v	GO:000646	04140(Reg	ca2+/calmo	23.033538	49.526713	6.4453814	15.729404	3.2721964	1.7102593	0.037804347	0.365805497	up	yes	Serine/thre	NEK4;NEK4
Pavir.1KG507400.v	GO:000551	00350(Tyro	bag family	1.620268	6.451992	1.440988	1.027789	3.2697404	1.7091761	0.029166711	0.319126625	up	yes	BAG family	BAG6
Pavir.1NG299600.v	GO:000467	04626(Plan	non-specific	9.2462461	12.369317	0.9044618	5.7079813	3.2689224	1.7088151	0.045423785	0.401733783	up	yes	Probable se	At1g01540;At1g01540;At1g01540
Pavir.8KG072100.v	GO:000367	NA	homeobox p	6.8806866	13.56331	1.0607771	5.1958096	3.2675957	1.7082295	0.040965677	0.380802418	up	yes	BEL1-like l	BLH1;BLH1;BLH1;BLH1;BLH1;BLH1;BLH1
Pavir.1KG048300.v	GO:000367	00511(Oth	protein of u	7.6662437	18.306629	2.3718193	5.5770557	3.2674904	1.708183	0.025692727	0.297214539	up	yes	NA	NA
Pavir.2KG017700.v	GO:000333	NA	proline tran	12.097408	31.559585	3.6421632	9.7244881	3.266113	1.7075747	0.035125651	0.352598474	up	yes	Probable pr	LOC_Os07g01090;LOC_Os07g01090;LOC_Os07g01090
Pavir.7NG210600.v	NA	NA	0	3.929224	9.976171	2.151825	2.10977	3.2629555	1.7061793	0.045147579	0.400541715	up	yes	NA	NA
Pavir.6NG214800.v	GO:000827	NA	2-methylen	5.738843	9.418085	1.260473	3.407401	3.2470731	1.6991399	0.043081921	0.39156193	up	yes	2-methylen	EO
Pavir.9NG129500.v	GO:000016	01230(Bios	gmp syntha	10.667655	11.284352	2.613504	4.148326	3.2464595	1.6988672	0.031420812	0.331492134	up	yes	Anthranilat	ASB2
Pavir.9NG577000.v	GO:005121	00941(Flav	oxidoreduct	4.6474402	11.810229	2.3566555	2.715113	3.2449568	1.6981993	0.04279847	0.389834405	up	yes	2-oxoglutar	2ODD11;2ODD11
Pavir.4KG108600.v	GO:000563	00100(Ster	reticulon//s	16.34774	34.761921	5.845795	9.907847	3.2443076	1.6979106	0.019584033	0.256267806	up	yes	Reticulon-li	RTNLB2
Pavir.5NG490900.v	GO:000551	NA	iq calmodul	3.777361	2.110378	0.485491	1.331276	3.2407783	1.6963403	0.049751897	0.421209557	up	yes	NA	NA
Pavir.9KG521900.v	NA	NA	0	3.095267	12.333072	1.860132	2.912235	3.2328484	1.6928059	0.042576656	0.389038382	up	yes	Protein EA	ERD7
Pavir.5NG447400.v	GO:000416	00900(Terp	farnesyl dip	70.009956	124.98942	17.13658	43.189713	3.232411	1.6926107	0.038306074	0.368933808	up	yes	Farnesyl py	FPS
Pavir.3KG425800.v	NA	NA	0	11.424055	23.739672	3.710781	7.172406	3.2310138	1.6919869	0.021516494	0.270200282	up	yes	NA	NA
Pavir.5KG540500.v	GO:000370	04626(Plan	wrky transc	6.989878	25.198633	4.929326	5.03973	3.2288424	1.691017	0.025505395	0.296006694	up	yes	Probable W	WRKY69
Pavir.2KG409700.v	GO:000950	00195(Phot	photosysten	328.80331	841.86737	125.14481	237.45242	3.2285704	1.6908955	0.039170845	0.373071272	up	yes	Photosysten	PSAG
Pavir.3KG159500.v	NA	NA	0	88.296471	96.178078	13.915937	43.227921	3.2282481	1.6907514	0.036721519	0.36052721	up	yes	Acyl transfe	AT7
Pavir.9KG328700.v	GO:000557	NA	pollen aller	5.123969	21.099567	3.54783	4.582707	3.2253141	1.6894397	0.033614487	0.343579198	up	yes	Expansin-B	EXPB6
Pavir.5NG117900.v	GO:000367	NA	hras-like su	15.182631	32.82711	4.37382	10.546768	3.2176842	1.6860228	0.026519333	0.303412787	up	yes	NA	NA
Pavir.5KG720800.v	GO:000815	03013(RNA	protein of u	2.9448718	8.656944	1.1884488	2.4190335	3.2160423	1.6852864	0.040792156	0.380028688	up	yes	NA	NA
Pavir.2KG331300.v	GO:000535	NA	sugar transp	3.312342	3.4193308	1.0771212	1.0176602	3.2135443	1.6841654	0.030438935	0.326114088	up	yes	NA	NA
Pavir.9KG024400.v	GO:000371	NA	thyroid rece	28.706562	27.371311	4.939343	12.524049	3.2111673	1.6830978	0.022690607	0.277704943	up	yes	NA	NA
Pavir.1NG376300.v	GO:000007	01200(Carb	cyclin (cycli	57.510197	48.712921	8.850236	24.25646	3.2085086	1.6819028	0.033603886	0.343566433	up	yes	Cyclin-P4-1	CYCP4-1
Pavir.6KG397500.v	GO:000551	NA	fas-associat	18.451305	40.261658	5.92853	12.373733	3.2079619	1.681657	0.034099735	0.34571758	up	yes	Plant UBX	PUX8
Pavir.9NG097900.v	GO:000573	NA	copper trans	161.67976	160.77362	33.495729	67.094241	3.2056216	1.6806041	0.032771202	0.339131364	up	yes	Protein PYI	PI21;PI21
Pavir.5KG685500.v	GO:000456	00520(Ami	beta-hexosa	3.8845196	4.2978572	0.7556775	1.7994736	3.2023064	1.6791113	0.027912452	0.310335091	up	yes	Beta-hexos	HEXO3;HEXO3;HEXO3
Pavir.1KG072600.v	GO:000437	00630(Glyo	glycine clea	207.03684	188.85133	39.327438	84.324623	3.2016302	1.6788067	0.033936994	0.344718755	up	yes	Glycine clea	GDH2
Pavir.7KG410600.v	GO:000467	04626(Plan	chaperone-a	1.270745	5.946419	0.944995	1.311564	3.198305	1.6773075	0.046091658	0.404532086	up	yes	Uncharacte	At1g71810
Pavir.6NG155600.v	GO:000367	04075(Plan	erebp-like f	57.346928	102.07397	15.622996	34.24942	3.1965745	1.6765267	0.034974983	0.351932254	up	yes	Ethylene-re	ERF060
Pavir.9KG497400.v	GO:000367	00195(Phot	photosysten	108.15093	235.11865	34.960667	72.450813	3.195837	1.6761938	0.035004821	0.352052525	up	yes	Photosysten	PSAH
Pavir.3KG407700.v	GO:000456	00052(Gala	beta-galact	123.31638	152.0134	19.451027	67.02877	3.1837468	1.6707256	0.04929738	0.418871651	up	yes	Beta-galact	Os12g0429200
Pavir.7KG407400.v	GO:000367	04075(Plan	camp-respo	1.925929	3.06834	0.571409	0.997622	3.1830276	1.6703997	0.039004341	0.372429507	up	yes	Transcriptic	TGAL6
Pavir.1KG362000.v	GO:000484	NA	protein of u	4.274371	6.0305432	0.860908	2.3771599	3.1824268	1.6701273	0.034041287	0.345497914	up	yes	E3 ubiquiti	RGLG2;RGLG2
Pavir.5NG173300.v	NA	NA	f-box domai	3.404799	7.462197	1.327477	2.090576	3.1792942	1.6687065	0.034556308	0.349403707	up	yes	NA	NA
Pavir.5NG209600.v	GO:000367	NA	sbp domain	1.904724	3.554985	0.756383	0.962364	3.1765635	1.6674669	0.034002684	0.345292665	up	yes	Squamosa p	SPL1
Pavir.9NG171200.v	GO:000367	NA	protein of u	17.152042	27.309429	4.744854	9.260186	3.1746765	1.6666096	0.025306808	0.295142751	up	yes	NA	NA
Pavir.8KG161900.v	GO:000367	NA	plastid-lipi	19.165924	59.020546	12.966862	11.723545	3.166674	1.6629684	0.023419046	0.281789387	up	yes	Probable pl	PAP2
Pavir.5KG436500.v	GO:000370	NA	nac domain	22.005147	9.3783655	1.7184665	8.203302	3.1630967	1.6613376	0.049174464	0.418155773	up	yes	NAC doma	NAC073;NAC073
Pavir.5NG336800.v	GO:000004	NA	solute carri	2.6086642	1.3901163	0.6333763	0.6352529	3.1520484	1.6562897	0.049422874	0.419634379	up	yes	Solute carri	Os06g0560000;Os06g0560000;Os06g0560000;Os06g0560000
Pavir.8KG179300.v	GO:000313	NA	hypoxia ind	5.880884	21.577522	4.843104	3.869855	3.1514444	1.6560132	0.032483328	0.337682291	up	yes	NA	NA
Pavir.4KG382000.v	GO:000455	04075(Plan	xyloglucan	6.298487	16.143932	2.510277	4.612919	3.1506109	1.6556316	0.029398902	0.320150906	up	yes	Probable xy	XTH23
Pavir.5KG397100.v	GO:000367	NA	zinc-finger	5.5958224	7.0758539	1.4820582	2.5404467	3.1501954	1.6554413	0.027310795	0.307615172	up	yes	FCS-Like Z	FLZ10;FLZ10;FLZ10;FLZ10
Pavir.8KG088200.v	GO:000166	04626(Plan	protein t08	8.1315689	14.756281	1.8732958	5.3953256	3.1488571	1.6548283	0.030600051	0.327001057	up	yes	Lipase-like	PAD4;PAD4;PAD4;PAD4
Pavir.2NG636600.v	GO:000367	NA	b3 domain-	24.59977	53.208451	7.4481223	17.268495	3.1480125	1.6544413	0.044495626	0.398414767	up	yes	B3 domain-	Os07g0679700;Os07g0679700
Pavir.5NG481900.v	GO:000981	00942(Anth	glucosyl/glu	9.97791	4.73594	1.377671	3.296511	3.1478984	1.654389	0.045080323	0.400506709	up	yes	UDP-glyco	UGT88F3
Pavir.5KG432800.v	GO:000983	00514(Oth	galactosylg	19.922138	13.165901	2.520539	7.994527	3.1467267	1.6538519	0.031119572	0.330075502	up	yes	Probable be	IRX9L
Pavir.1KG455000.v	GO:000484	NA	inhibitor of	9.818743	31.750181	4.972134	8.250871	3.1436821	1.6524553	0.029314432	0.319970669	up	yes	NA	NA
Pavir.9NG740500.v	GO:000467	04626(Plan	non-specific	26.793274	55.742992	8.321577	17.947075	3.1420061	1.651686	0.038645143	0.370386463	up	yes	Probable se	PBL16
Pavir.9NG074700.v	GO:000447	01230(Bios	s-adenosylm	434.3011	880.14825	133.33564	285.1458	3.140998	1.651223	0.045296775	0.401116129	up	yes	S-adenosyl	SAM1;SAM1;SAM1
Pavir.9NG361100.v	GO:000455	00511(Oth	alpha-mann	14.753794	5.363889	1.40915	5.002991	3.1374362	1.6495861	0.043750768	0.395155675	up	yes	Alpha-mann	-
Pavir.9KG292800.v	GO:000367	NA	pddexk-like	10.069388	43.441265	5.46638	11.591806	3.1369486	1.6493619	0.043290339	0.39250673	up	yes	NA	NA
Pavir.5KG772600.v	GO:000367	NA	protein men	64.035927	70.387001	20.988556	21.890909	3.1349022	1.6484204	0.022926895	0.279744358	up	yes	NA	NA
Pavir.8NG083400.v	GO:000402	00010(Glyc	aldehyde de	20.765999	32.7164	8.030251	9.045819	3.1320087	1.6470882	0.021537254	0.270221052	up	yes	Aldehyde d	ALDH3H1
Pavir.1NG375500.v	GO:000467	04626(Plan	non-specific	4.537951	3.979233	0.87418	1.845545	3.1316343	1.6469157	0.039978594	0.377133998	up	yes	Probable se	PBL19
Pavir.5NG107300.v	GO:000551	NA	cyclomaltod	5.4229157	17.045598	3.3003807	3.8749749	3.1313449	1.6467824	0.026020971	0.300087767	up	yes	NA	NA
Pavir.7NG267200.v	NA	NA	histone h1/	3.090486	6.286517	1.523572	1.473188	3.129047	1.6457233	0.033243638	0.341834488	up	yes	NA	NA
Pavir.7NG253900.v	GO:000563	04712(Circ	b-box zinc f	6.357625	28.590118	4.991158	6.178332	3.1288575	1.645636	0.036023284	0.356842298	up	yes	Zinc finger	COL5
Pavir.7NG400700.v	GO:000467	00520(Ami	protein kina	3.485278	5.52029	0.660655	2.220011	3.1262104	1.6444149	0.039188165	0.373141648	up	yes	Probable LI	At1g56130
Pavir.7KG420900.v	GO:000367	04626(Plan	erebp-like f	25.392635	39.205585	7.063065	13.614338	3.1240974	1.6434394	0.021699126	0.270864201	up	yes	Ethylene-re	ERF008
Pavir.9NG239100.v	NA	NA	protein of u	12.760776	9.840624	2.248606	4.988357	3.1230504	1.6429558	0.032611225	0.338561609	up	yes	NA	NA
Pavir.6KG407300.v	GO:000573	00053(Asc	disulfide ox	12.333402	12.856648	2.761683	5.305291	3.1226145	1.6427545	0.020017365	0.258902742	up	yes	Monodehyd	MDAR4
Pavir.1NG083800.v	GO:000981	00940(Phe	udp-glycosy	3.195244	3.195887	1.697178	1.674256	3.1177033	1.6404837	0.04158278	0.383879555	up	yes	Gallate 1-b	UGT84A13
Pavir.5KG775700.v	GO:000016	02010(ABC	atp-binding	3.27451	5.069748	0.976794	1.699844	3.1174399	1.6403617	0.031326688	0.331185922	up	yes	ABC transp	ABCD1
Pavir.5KG770500.v	GO:000367	00520(Ami	mac/perfori	14.758154	12.158169	2.966804	5.675284	3.1145625	1.6390295	0.02044947	0.262503559	up	yes	MACPF do	At4g24290
Pavir.2NG617900.v	GO:000551	04075(Plan	camp-respo	10.973483	28.87892	6.58712	6.209226	3.114358	1.6389348	0.021970829	0.272986098	up	yes	Transcriptic	TGA2.2
Pavir.7NG123800.v	GO:000367	00531(Glyc	protein of u	5.849621	9.921909	1.433919	3.63488	3.1114925	1.6376068	0.033175673	0.3416967	up	yes	NA	NA
Pavir.5NG540500.v	NA	NA	0	9.1751	5.288973	2.326538	2.326035	3.1088331	1.6363732	0.035696986	0.355331178	up	yes	NA	NA
Pavir.5KG667500.v	GO:000551	03060(Prot	mads box p	15.581908	16.141869	3.765424	6.442113	3.1078777	1.6359298	0.021383245	0.269246869	up	yes	MADS-box	MADS2
Pavir.7KG231300.v	GO:000467	04626(Plan	non-specific	3.0557471	8.9645826	1.898208	1.9709696	3.1066886	1.6353776	0.037276157	0.363533126	up	yes	Calmodulin	CRCK3;CRCK3;CRCK3;CRCK3
Pavir.2KG034800.v	GO:000950	00195(Phot	photosysten	95.588768	307.14957	49.971466	79.858818	3.1020369	1.6332159	0.047373387	0.409940183	up	yes	Photosysten	PSAK
Pavir.2NG380300.v	GO:000950	00195(Phot	photosysten	205.62082	536.7641	80.178993	159.4892	3.097553	1.631129	0.049952808	0.422244516	up	yes	Photosysten	PSAG
Pavir.9NG388400.v	GO:000367	NA	pddexk-like	8.599886	35.486244	5.545574	8.695892	3.0956174	1.6302272	0.046497759	0.40582121	up	yes	NA	NA

Pavir.1KG210500.v	GO:00038	NA	rna polymer	7.519738	12.666872	2.453841	4.069709	3.094421	1.6296695	0.023767555	0.284582003	up	yes	RNA polym	SIGE
Pavir.5KG766600.v	GO:00039	04626(Plan	nad(p)h deh	4.853054	5.939391	1.607301	1.882529	3.0925418	1.6287931	0.04792386	0.412896854	up	yes	Probable N.	Os01g0954000
Pavir.8KG393900.v	NA	NA	metallothio	157.40749	334.23486	54.868614	104.18561	3.0910361	1.6280905	0.044596575	0.398414767	up	yes	Metallothio	ERR26
Pavir.1NG143200.v	GO:000981	01210(2-O	pathogen-in	4.795641	10.545937	1.555095	3.430339	3.0772803	1.6216559	0.03770698	0.365544935	up	yes	UDP-glucos	-
Pavir.1NG034000.v	GO:000467	04075(Plan	mitogen-act	2.8435728	2.5654445	0.6275019	1.1314411	3.075152	1.6206577	0.032911806	0.340004207	up	yes	Mitogen-act	MPK13;MPK13;MPK13;MPK13;MPK13;MPK13;MPK13;MPK13;MPK13;MPK13
Pavir.9NG222600.v	GO:000367	NA	post-illumi	18.891201	39.569859	5.890256	13.125998	3.0742679	1.6202429	0.029606672	0.321404863	up	yes	Protein POS	PIFI
Pavir.5KG754300.v	GO:000382	00240(Pyri	spermatoge	7.67913	9.996744	2.839085	2.915033	3.0718651	1.6191148	0.015912098	0.231765709	up	yes	Probable po	HAK2
Pavir.5KG004400.v	GO:000573	04120(Ubic	ubiquitin-co	29.12104	36.970084	7.1040223	14.420728	3.0704711	1.61846	0.026055501	0.300253192	up	yes	Probable ub	UBC25;UBC25;UBC25
Pavir.9NG325700.v	GO:000573	NA	copper trans	32.435284	50.631413	10.15339	16.916748	3.0685731	1.6175679	0.03324002	0.341834488	up	yes	Heavy meta	HIPP05
Pavir.9KG130400.v	GO:000563	04626(Plan	interleukin-	10.724897	25.131769	2.625335	9.066894	3.0667092	1.6166914	0.042968291	0.390907404	up	yes	Salt toleran	OsI_16820
Pavir.5KG754900.v	GO:000940	01200(Carb	phosphoglu	29.144547	44.626148	10.954189	13.101517	3.066661	1.6166687	0.022725361	0.278039729	up	yes	6-phosphog	G6PGH1
Pavir.9KG392200.v	GO:000538	NA	zinc/iron tra	146.54884	179.43561	39.212139	67.092697	3.0665063	1.6165959	0.035847983	0.355950385	up	yes	Zinc transp	ZIP2
Pavir.6KG113300.v	NA	NA	0	147.49078	113.70611	20.994205	64.21273	3.0654417	1.616095	0.048150272	0.413516993	up	yes	NA	NA
Pavir.4KG053600.v	GO:000573	NA	bag family	45.082047	72.10775	13.965154	24.277615	3.0643648	1.6155881	0.035682338	0.355331178	up	yes	BAG family	BAG1
Pavir.9NG698500.v	GO:000484	NA	26s proteas	7.389074	24.641825	5.312038	5.149252	3.0618498	1.6144035	0.028932202	0.317022348	up	yes	E3 ubiquiti	XB3
Pavir.9KG195700.v	GO:000655	00270(Cyst	1,2-dihydro	4.1990824	10.856439	2.3871538	2.5324761	3.0602955	1.613671	0.039643906	0.375475482	up	yes	1,2-dihydro	ARD4;ARD4;ARD4
Pavir.7KG288200.v	GO:000573	00906(Carc	phytoene de	2.2731883	4.4592479	0.8198367	1.3802111	3.0601318	1.6135938	0.048876064	0.416799896	up	yes	Zeta-carote	ZDS;CPK12;CPK12
Pavir.2NG152300.v	GO:000368	NA	transcriptio	26.728939	44.504887	6.142361	17.137695	3.0598649	1.613468	0.041690112	0.384303867	up	yes	Transcriptic	MYBS3
Pavir.5KG696300.v	NA	NA	rhodanese/c	21.915281	50.190788	7.585172	16.004923	3.0566248	1.6119395	0.044211335	0.397274622	up	yes	NA	NA
Pavir.3NG275700.v	GO:000837	NA	core-2/i-bra	12.609445	17.03731	2.477795	7.243238	3.0497536	1.6086927	0.031949501	0.334642267	up	yes	Glycosyltra	BC10
Pavir.9NG223400.v	GO:000367	04146(Pero	peroxisoma	19.280029	63.85088	11.669268	15.612254	3.0471507	1.6074609	0.045136514	0.400541715	up	yes	NA	NA
Pavir.8KG073100.v	NA	NA	0	17.84174	45.240959	6.929951	13.806117	3.0421727	1.605102	0.035709965	0.355331178	up	yes	NA	NA
Pavir.7NG380200.v	GO:000367	04626(Plan	erebp-like f	34.210209	79.095337	10.528356	26.768175	3.0379647	1.6031051	0.035568959	0.354773688	up	yes	Ethylene-re	ERF008
Pavir.9NG431900.v	GO:000681	04146(Pero	protein of u	4.973226	8.782141	1.004729	3.524914	3.0367442	1.6025254	0.047818075	0.412174478	up	yes	NAD(P)H-c	ndhS
Pavir.2KG515800.v	GO:000377	04626(Plan	formin-relat	16.71697	12.94631	1.960162	7.824467	3.0316203	1.6000891	0.041440587	0.383131682	up	yes	Formin-like	FH13
Pavir.7NG380500.v	GO:000367	04626(Plan	floral home	11.056784	10.123292	2.2047349	4.7837221	3.0307228	1.5996619	0.024940653	0.292933778	up	yes	APETALA	AP2-5;AP2-5
Pavir.7KG137100.v	GO:000449	00905(Bras	cytochrome	4.315599	6.802637	1.727447	1.941993	3.0299544	1.5992961	0.021471701	0.26972793	up	yes	Cytochrome	CYP72A14
Pavir.9NG460900.v	GO:000697	03018(RNA	peptide met	10.766022	17.446844	4.183806	5.153649	3.0214728	1.5952519	0.024261746	0.28738543	up	yes	Peptide met	MSRA4
Pavir.1KG453400.v	GO:000484	NA	inhibitor of	8.741855	24.270201	4.106441	6.821313	3.020937	1.5949961	0.03130698	0.331071534	up	yes	NA	NA
Pavir.5KG750200.v	GO:000582	00960(Trop	tropinone re	9.877516	7.351116	2.958089	2.745464	3.0206841	1.5948753	0.032203572	0.335901453	up	yes	Tropinone r	At5g06060
Pavir.9KG320100.v	GO:000557	04075(Plan	xyloglucan:	37.574009	16.103876	11.514643	6.268236	3.0185149	1.5938389	0.037443011	0.364237061	up	yes	Xyloglucan	XTH31
Pavir.2KG495000.v	GO:000370	04075(Plan	scarecrow-li	9.342007	20.454277	6.102854	3.777667	3.0156592	1.5924734	0.023247874	0.281238111	up	yes	Scarecrow-l	SCL23
Pavir.6NG202400.v	GO:000367	NA	expressed p	11.276608	25.361731	4.113594	8.044605	3.0134676	1.5914246	0.030598911	0.327001057	up	yes	NA	NA
Pavir.4NG187100.v	GO:000953	00860(Porp	heme oxyge	5.264381	18.920813	3.819731	4.213793	3.0105336	1.5900192	0.040623675	0.379595456	up	yes	Heme oxyge	HO1
Pavir.4NG032000.v	GO:000367	NA	myeloid leu	7.813264	22.649799	3.222138	6.896889	3.0104735	1.5899904	0.039454501	0.374504823	up	yes	NA	NA
Pavir.3KG358800.v	GO:000592	NA	tubby-relate	42.160194	65.162849	14.776375	20.879736	3.0099481	1.5897386	0.033255547	0.341863386	up	yes	Tubby-like	TULP9
Pavir.5KG514600.v	GO:000557	00040(Pent	pectinestera	26.07262	84.776772	15.807056	21.036755	3.0086299	1.5891067	0.04566441	0.403062241	up	yes	Probable pe	PME53
Pavir.8NG211300.v	NA	NA	0	10.234064	6.377556	2.631669	2.890313	3.0082713	1.5889347	0.04815098	0.413516993	up	yes	NA	NA
Pavir.6KG170600.v	GO:000534	NA	purine nucle	12.0517	37.588474	7.648671	8.864534	3.0060896	1.587888	0.030459298	0.326146251	up	yes	Probable pu	PUP4
Pavir.9KG110400.v	GO:000582	NA	[protein-pii	3.6901506	5.7684779	1.031533	2.1152397	3.0058189	1.5877581	0.044090771	0.396605823	up	yes	ACT domai	ACR4;ACR4
Pavir.7KG268400.v	GO:000042	04144(Endo	guanyl-nucl	10.728441	23.914627	3.1111667	8.4297267	3.0017665	1.5858118	0.037748233	0.365544935	up	yes	SH3 domai	SH3P2;SH3P2
Pavir.9NG453100.v	GO:000563	NA	platz transc	5.843935	26.801727	4.549657	6.35172	2.9946365	1.5823809	0.043431122	0.393213865	up	yes	NA	NA
Pavir.5NG624700.v	GO:000399	NA	inactive pur	7.875472	16.451677	3.056867	5.067738	2.9942562	1.5821977	0.032443127	0.337482178	up	yes	Probable in	PAP16
Pavir.8NG117000.v	GO:001678	NA	alpha/beta h	12.875936	12.394037	2.95868	5.497576	2.9883169	1.5793332	0.039805622	0.376276654	up	yes	NA	NA
Pavir.7KG413400.v	GO:000377	03008(Ribo	kinesin fam	37.652205	39.942297	8.6723082	17.306164	2.9868771	1.5786379	0.040025287	0.377133998	up	yes	Kinesin-like	KIN14H;KIN14H
Pavir.2KG249100.v	NA	NA	0	8.462905	14.553961	2.542688	5.167829	2.9851262	1.5777919	0.028186729	0.311980065	up	yes	NA	NA
Pavir.1KG093700.v	GO:000981	00940(Pher	udp-glycosy	3.793217	10.838423	2.885702	2.019827	2.9826834	1.5766109	0.044263211	0.397490825	up	yes	Gallate 1-b	UGT84A13
Pavir.9KG514500.v	GO:000367	NA	fantastic fo	6.596275	3.478188	2.146179	1.233121	2.9812278	1.5759066	0.033040095	0.340400939	up	yes	Protein FAI	At5g22090
Pavir.5KG744000.v	GO:000552	04626(Plan	leucine-rich	3.742267	3.525014	0.842721	1.59714	2.9785635	1.5746167	0.035846763	0.355950385	up	yes	Disease resi	RPP13
Pavir.3KG227800.v	GO:000484	NA	phytochrom	4.208036	19.169903	4.543738	3.319135	2.9732057	1.5720193	0.044409414	0.398226279	up	yes	Phytochrom	EID1
Pavir.5KG172400.v	GO:000367	04712(Circ	dof domain,	3.163823	7.37781	1.545474	2.000836	2.9725639	1.5717078	0.038880392	0.371529239	up	yes	Cyclic dof f	CDF2
Pavir.3KG481600.v	NA	NA	protein of u	7.4612416	8.226103	2.640302	2.63715	2.9725225	1.5716877	0.028157348	0.311805491	up	yes	NA	NA
Pavir.1NG048700.v	NA	NA	0	5.4296185	10.786351	1.586156	3.8695349	2.9723036	1.5715815	0.035810778	0.355769003	up	yes	NA	NA
Pavir.5KG109100.v	GO:000367	NA	epididymal	23.632818	16.86405	3.95523	9.669578	2.9722891	1.5715744	0.030889268	0.32878257	up	yes	NA	NA
Pavir.2KG570300.v	GO:000551	04075(Plan	camp-respo	12.493983	28.626343	5.690734	8.15309	2.9703011	1.5706092	0.03850993	0.370154411	up	yes	Transcriptic	TGA2.2
Pavir.3KG362200.v	GO:000573	NA	rubber elon	32.01526	36.652446	5.4111	17.714243	2.9693703	1.570157	0.037760432	0.365568847	up	yes	REF/SRPP-	OsI_017815;OsI_017815
Pavir.9KG554200.v	GO:000027	00500(Starc	galacturono	34.672527	43.692169	9.294834	17.104601	2.9684232	1.5696968	0.038590786	0.37029766	up	yes	Probable ga	GATL7
Pavir.9NG098500.v	GO:000685	NA	oligopeptid	14.755434	19.936279	5.096571	6.595116	2.9672119	1.569108	0.026796905	0.304882556	up	yes	Oligopeptid	OPT3
Pavir.1KG400500.v	GO:004316	04141(Prot	glucose-ind	9.239856	23.774507	4.723994	6.4071866	2.9659354	1.5684872	0.029515139	0.320883193	up	yes	E3 ubiquiti	PRT1;PRT1;PRT1;PRT1;PRT1;PRT1
Pavir.2KG204300.v	GO:000983	00514(Othe	beta-1,4-xyl	211.0311	198.37892	44.425156	93.888702	2.9600072	1.5656007	0.048171929	0.413516993	up	yes	Probable be	GT43A
Pavir.2NG024600.v	GO:000451	NA	hnh endonu	6.343818	11.867546	3.154432	3.002761	2.957738	1.5644943	0.034737935	0.350391522	up	yes	NA	NA
Pavir.9NG296100.v	GO:000573	NA	membrane a	4.2972718	11.511345	2.647352	2.7012537	2.9556519	1.5634764	0.029827654	0.322600583	up	yes	NA	NA
Pavir.9KG150600.v	GO:000588	NA	lim domain	9.241265	25.262688	3.381116	8.298009	2.9543269	1.5628295	0.042631093	0.389038382	up	yes	Protein DA	DAR1
Pavir.7KG295000.v	GO:000367	NA	uncharacteri	5.162183	9.148166	2.020858	2.823434	2.9540641	1.5627011	0.043887018	0.395740974	up	yes	NA	NA
Pavir.9NG128600.v	GO:000437	01200(Carb	triokinase /	21.767962	41.224777	9.413473	11.92282	2.952375	1.561876	0.036220989	0.357725139	up	yes	Putative 3,4	DHBK
Pavir.2NG450100.v	GO:000467	04626(Plan	protein kina	7.621688	6.078187	1.158402	3.483619	2.9512738	1.5613378	0.048729752	0.416253551	up	yes	Probable se	PBL19
Pavir.3KG464000.v	NA	NA	small vcp/p	8.7684137	28.472861	4.3616026	8.2706539	2.9481095	1.5597901	0.042071656	0.386588004	up	yes	NA	NA
Pavir.2KG018100.v	GO:000551	00750(Vita	pyridoxal 5'	16.743856	31.300394	6.806017	9.496481	2.9470484	1.5592707	0.025346963	0.295214331	up	yes	Probable py	PDX11
Pavir.8KG173200.v	NA	NA	0	28.244202	63.052494	9.550591	21.447924	2.9451958	1.5583635	0.03889311	0.37155627	up	yes	NA	NA
Pavir.1KG533800.v	GO:000367	NA	f23a5.30 pr	31.596689	54.800304	8.257447	21.080288	2.9449101	1.5582236	0.042027457	0.386370849	up	yes	Protein HE	HDR1
Pavir.1KG401600.v	GO:000016	03013(RNA	rna recognit	12.265133	18.168152	5.4653431	4.8721488	2.9439718	1.5577638	0.022861535	0.279203442	up	yes	Oligouridyl	UBP1B;UBP1B
Pavir.9NG431400.v	GO:000576	00130(Ubic	methyltrans	7.097481	9.179901	1.84814	3.681707	2.9435502	1.5575572	0.049069072	0.417687411	up	yes	NA	NA
Pavir.7NG442700.v	GO:000582	NA	ring finger p	6.7713379	12.801026	3.1900742	3.4618309	2.9423697	1.5569785	0.031156116	0.330075502	up	yes	E3 ubiquiti	AIRP2;AIRP2;AIRP2;AIRP2;AIRP2
Pavir.9KG463500.v	GO:000372	03040(Splic	pre-mrna sp	13.528336	29.661139	6.206577	8.484592	2.9398256	1.5557306	0.032598025	0.338561609	up	yes	Protein high	HCF107
Pavir.8NG137400.v	GO:000551	04626(Plan	leucine-rich	3.201943	6.157037	0.826539	2.361102	2.9360207	1.5538621	0.04917974	0.418155773	up	yes	Disease resi	RGA5
Pavir.5KG272900.v	GO:000367	00190(Oxic	myb-like dn	11.458057	21.089422	5.770506	5.317262	2.9354401	1.5535768	0.030879015	0.32878257	up	yes	Myb-relatec	MYBAS2
Pavir.5KG604700.v	GO:000563	NA	bag family	56.524788	35.496567	11.936212	19.489943	2.9281773	1.5500029	0.040583133	0.379595456	up	yes	BAG family	BAG4
Pavir.9KG398000.v	GO:000582	NA	protein wea	4.958092	13.464616	1.721375	4.57745	2.9247849	1.5483305	0.047060362	0.408683509	up	yes	Protein WE	WEL2
Pavir.7KG277700.v	GO:000367	04626(Plan	duo pollen	10.162048	13.820641	3.509289	4.695977	2.9228411	1.5473714	0.025461669	0.295794776	up	yes	Probable tra	MYB58
Pavir.9NG612300.v	NA	NA	0	7.33431	7.13623	2.585771	2.365561	2.922555	1.5472302	0.04958847	0.420489188	up	yes	NA	NA
Pavir.5KG053000.v	GO:000367	NA	n-myc dowr	58.543804	40.082443	13.085529	20.668339	2.9219243	1.5469188	0.03830903	0.368933808	up	yes	Protein ND	NDL2
Pavir.1NG564100.v	GO:000552	03018(RNA	abc transpo	19.568405	29.094139	5.934969	10.752132	2.9161772	1.5440784	0.027735347	0.309489406	up	yes	ABC transp	ABCI19

Pavir.2KG351200.v4	NA	NA	0	5.035521	6.093894	1.574422	2.244314	2.9144238	1.5432107	0.045089383	0.400506709	up	yes	NA	NA
Pavir.7KG248400.v4	GO:000819	00906(Caro	glucosyl/glu	6.9221816	7.6324464	1.308038	3.689236	2.9125135	1.5422647	0.046155384	0.404713246	up	yes	UDP-glycos	UGT92A1;UGT92A1
Pavir.2KG415700.v4	GO:000531	NA	anion transp	3.493865	7.147634	2.041346	1.614425	2.9108768	1.5414538	0.046474575	0.40582121	up	yes	Probable an	PHT4;5
Pavir.4NG170600.v4	GO:000557	00906(Caro	zinc finger f	2.8243204	8.3456499	1.8452821	1.9962792	2.9076642	1.5398607	0.045695308	0.403094714	up	yes	GDSL ester	At5g45910;At1g28600
Pavir.2NG534100.v4	GO:000300	00600(Sphi	sphingosine	7.9439722	20.776294	3.964962	5.9390646	2.8998575	1.535982	0.033885102	0.344471032	up	yes	Sphingosine	SPHK1;SPHK1
Pavir.2KG347700.v4	GO:000962	04626(Plan	erebp-like fa	111.58918	213.91905	47.19471	65.166992	2.8969678	1.5345436	0.047950463	0.413031352	up	yes	Ethylene-res	EREBP1
Pavir.5KG005800.v4	GO:000815	NA	heme-bindin	117.36929	99.416031	24.730886	50.160698	2.8946553	1.5333916	0.04554689	0.402456212	up	yes	NA	NA
Pavir.1KG067500.v4	GO:000467	04075(Plan	non-specific	10.377362	10.461159	2.316264	4.890209	2.8916394	1.5318876	0.02995555	0.323332338	up	yes	Cold-respon	CRPK1
Pavir.5NG418000.v4	GO:000563	04144(Endo	amsh-like u	6.2384077	12.05737	1.6389114	4.6902852	2.8906952	1.5314165	0.048728831	0.416253551	up	yes	AMSH-like	AMSH2;AMSH2;AMSH2;AMSH2;AMSH2
Pavir.1NG503800.v4	NA	NA	0	18.360016	45.818119	9.155324	13.049236	2.8903133	1.5312259	0.032986522	0.340214817	up	yes	NA	NA
Pavir.9KG287800.v4	GO:000681	04146(Pero	protein of u	7.9422353	14.407427	2.2171883	5.5184009	2.8891997	1.5306699	0.039046839	0.372551269	up	yes	NAD(P)H-q	ndhS;ndhS;ndhS;ndhS
Pavir.5NG491100.v4	GO:000577	NA	n-acetylated	6.157698	14.33241	3.6092318	3.4901654	2.8861758	1.5291592	0.028540457	0.314011887	up	yes	Probable gl	LAMP1;LAMP1;LAMP1
Pavir.3KG329500.v4	GO:000550	NA	dnaj homolo	23.729094	45.991234	8.671947	15.548378	2.8785876	1.5253611	0.037909097	0.366347356	up	yes	NA	NA
Pavir.9NG775900.v4	GO:000006	03060(Prot	mads-box p	4.7950985	6.1236419	1.286698	2.5082891	2.8771483	1.5246396	0.03370346	0.343927244	up	yes	MADS-box	MADS50;MADS50
Pavir.5KG771600.v4	GO:000588	NA	cyanobacter	21.3384	19.093423	6.8219322	7.2903059	2.8650185	1.5185445	0.025650877	0.296913253	up	yes	NAD(P)H-q	ndhO;ndhO
Pavir.9KG639800.v4	GO:000588	NA	nitrate, from	11.003926	32.29562	8.2430599	6.897172	2.8598998	1.5159646	0.034451558	0.348626018	up	yes	CASP-like	Sb01g044070;Sb01g044070
Pavir.9KG612800.v4	GO:000367	00970(Ami	transcriptio	54.978374	108.74071	25.384407	31.894854	2.8582611	1.5151377	0.043989441	0.396121577	up	yes	Nuclear tra	NFYA3
Pavir.9KG395300.v4	GO:000367	NA	zinc-finger d	13.262133	26.909346	8.903002	5.157249	2.8570954	1.5145492	0.028692554	0.315011423	up	yes	FCS-Like Z	FLZ13
Pavir.2NG461100.v4	GO:000367	NA	protein nlp1	3.855858	6.53743	1.887136	1.752476	2.8556033	1.5137956	0.037540545	0.364567305	up	yes	Protein NLI	NLP1
Pavir.1NG286000.v4	GO:000588	03040(Splic	protein of u	10.730313	12.645445	2.848134	5.340391	2.854697	1.5133376	0.029741427	0.322131631	up	yes	Protein PSK	PSI1
Pavir.4NG196700.v4	NA	NA	0	19.263212	33.288409	8.2248451	10.21392	2.8500618	1.5109932	0.030152928	0.324425589	up	yes	NA	NA
Pavir.9NG684200.v4	GO:000027	00500(Starc	galacturono	32.467033	34.712719	7.50204	16.132547	2.8424339	1.5071268	0.041180035	0.381756095	up	yes	Probable ga	GATL7
Pavir.5KG418900.v4	GO:000098	NA	transcriptio	1.658802	4.198831	1.299717	0.761898	2.8412837	1.5065429	0.047749731	0.412130382	up	yes	Transcriptio	DPB
Pavir.5KG755300.v4	GO:000578	NA	vesicle-asso	23.464422	29.98859	7.784883	11.047871	2.8383003	1.5050273	0.033782911	0.344083852	up	yes	Vesicle-asso	PVA22
Pavir.9NG124000.v4	GO:001602	NA	atp-depende	13.270855	19.454088	4.255029	7.284724	2.8358443	1.5037783	0.030340676	0.325339676	up	yes	Sulfite expo	At2g36630
Pavir.9NG659200.v4	GO:000367	00052(Gala	serine/threo	4.94936	6.35102	1.741301	2.246667	2.8336185	1.5026455	0.040273491	0.378298971	up	yes	Zinc finger	ENY
Pavir.5KG778300.v4	GO:000551	00910(Nitro	cytochrome	213.5202	144.07123	51.392796	74.908966	2.8312466	1.5014374	0.047610474	0.411328658	up	yes	Cytochrome	Os05g0108800
Pavir.3KG187600.v4	GO:000973	04075(Plan	auxin-respo	44.955013	38.024792	10.809906	18.499313	2.8311844	1.5014057	0.034516173	0.349185829	up	yes	Auxin-respo	IAA15
Pavir.7NG258100.v4	GO:000524	NA	potassium c	6.337502	11.060658	1.680535	4.477632	2.8252173	1.4983618	0.049018464	0.41735118	up	yes	BTB/POZ d	At2g24240
Pavir.2KG365400.v4	GO:004665	NA	plastocyanin	25.270721	14.408271	6.261922	7.784726	2.8248015	1.4981495	0.031916905	0.334508754	up	yes	Blue copper	BCP
Pavir.9NG521800.v4	GO:000815	NA	zinc-finger d	25.137236	48.787292	17.605927	8.568795	2.8242718	1.4978789	0.042532731	0.389038382	up	yes	FCS-Like Z	FLZ14
Pavir.9KG502000.v4	GO:000016	03040(Splic	transformer-	10.135264	10.931937	3.892601	3.586161	2.8169369	1.4941273	0.0326262	0.338561609	up	yes	Serine/argin	SR45A
Pavir.1KG467600.v4	GO:000367	03013(RNA	dna2/nam7	1.528924	4.222142	1.129039	0.913952	2.8150227	1.4931465	0.048090998	0.413516993	up	yes	Regulator o	UPF1
Pavir.2NG149000.v4	GO:000973	NA	lob domain-	11.234373	13.887255	5.799175	3.131252	2.8130377	1.4921289	0.038200678	0.368312571	up	yes	LOB domai	LBD40
Pavir.9NG491600.v4	NA	NA	0	11.78931	10.432344	1.959496	5.950738	2.8092284	1.4901739	0.049789747	0.421245255	up	yes	RING-H2 f	ATL80
Pavir.5KG772800.v4	GO:000550	00860(Porp	chlorophylli	19.794639	27.682103	7.2259587	9.6820052	2.8079515	1.489518	0.040707522	0.380000927	up	yes	Chlorophyll	CAO;CAO
Pavir.6NG123700.v4	GO:000573	03013(RNA	copper trans	153.64752	98.846596	32.63163	57.544437	2.8000125	1.4854333	0.049723605	0.421159828	up	yes	Protein SOI	NAKR2
Pavir.4NG291800.v4	GO:000842	00500(Starc	beta-glucosi	7.009772	17.285919	3.8157956	4.8739203	2.7959132	1.4833196	0.037738771	0.365544935	up	yes	Beta-glucos	BGLU25;BGLU25
Pavir.4KG065100.v4	GO:000563	04075(Plan	transcriptio	6.8688516	6.8797087	2.0752775	2.8477223	2.7927201	1.481671	0.039460905	0.374504823	up	yes	Transcriptio	SPT;SPT;SPT
Pavir.1NG341000.v4	GO:000098	NA	b-box zinc f	34.448948	83.294121	20.024315	22.399437	2.7754044	1.472698	0.049703923	0.421146189	up	yes	B-box zinc	BBX24
Pavir.9NG291000.v4	GO:000467	03008(Ribo	serine/threo	9.283809	24.054415	4.80132	7.223975	2.7723415	1.471105	0.041354356	0.3826169	up	yes	Serine/threo	STN7
Pavir.6KG226700.v4	GO:000013	00500(Starc	galacturono	21.802853	15.144913	6.427112	6.915762	2.769101	1.4694177	0.031104682	0.330075502	up	yes	Probable ga	GAUT4
Pavir.7NG380100.v4	GO:004316	03013(RNA	ring finger d	11.115757	14.151558	3.699164	5.441004	2.7644257	1.4669798	0.030325642	0.325339676	up	yes	Probable E3	HIP1
Pavir.5NG204500.v4	NA	NA	0	17.959137	17.659792	5.392618	7.50572	2.7615131	1.465459	0.030569868	0.326982236	up	yes	Germin-like	GER4
Pavir.9NG124100.v4	NA	NA	0	7.136189	10.495817	4.077311	2.309445	2.7607139	1.4650414	0.033338215	0.342343779	up	yes	NA	NA
Pavir.6NG207900.v4	GO:004316	04141(Prot	ring/u-box d	18.586063	27.933706	9.382848	7.487771	2.7574429	1.463331	0.02858417	0.314260236	up	yes	Probable E3	XERICO
Pavir.5KG775300.v4	GO:003514	NA	growth inhil	4.726999	9.339189	3.070773	2.078992	2.7314233	1.4496529	0.037531327	0.364567305	up	yes	NA	NA
Pavir.3KG105700.v4	GO:000367	00190(Oxid	myb-like dn	22.068214	39.245068	11.684521	10.793284	2.7277255	1.4476985	0.038213926	0.368345773	up	yes	Myb-related	MYBAS2
Pavir.3KG554800.v4	GO:000090	00430(Taur	cysteamine d	69.959869	80.485718	33.534271	21.622498	2.7275997	1.4476319	0.044015606	0.396121577	up	yes	Plant cystei	PCO2
Pavir.5KG768900.v4	GO:000395	00760(Nico	nad(+) kinas	7.091372	4.151955	1.904829	2.24047	2.7123078	1.4395209	0.04670861	0.406640471	up	yes	Probable N	Os01g0957000
Pavir.2NG342800.v4	GO:000521	NA	xanthine-ura	13.078233	9.278734	3.153496	5.11184	2.7049072	1.4355791	0.03735356	0.363911658	up	yes	Nucleobase	NAT6
Pavir.5KG505700.v4	GO:000389	NA	rna polymer	8.833452	13.693217	3.17771	5.165881	2.6998769	1.4328937	0.044516582	0.398414767	up	yes	RNA polym	SIGE
Pavir.9KG486500.v4	GO:000563	NA	phosphatidy	2.4981964	5.0119956	1.5427308	1.2487896	2.690359	1.4277987	0.046394508	0.40582121	up	yes	NA	NA
Pavir.6KG376600.v4	GO:000551	NA	uncharacteri	7.490901	4.544971	2.418496	2.060536	2.6871592	1.4260818	0.049098722	0.417845141	up	yes	NA	NA
Pavir.4NG060200.v4	GO:000367	NA	late embryo	14.001669	26.317601	5.5088123	9.5188477	2.6830039	1.4238491	0.042837436	0.390061015	up	yes	NA	NA
Pavir.9NG749700.v4	GO:000821	00860(Porp	pheophorbid	6.076939	18.634144	5.726314	3.487015	2.6821014	1.4233638	0.047816701	0.412174478	up	yes	Pheophorbi	PAO
Pavir.5KG613000.v4	GO:000382	00940(Pher	nad depende	11.352589	14.197638	3.119624	6.408895	2.6814479	1.4230122	0.046075931	0.404532086	up	yes	Cinnamoyl-	CCR1
Pavir.2NG300900.v4	NA	NA	0	44.85667	22.932514	10.930031	14.359981	2.6804726	1.4224874	0.039453422	0.374504823	up	yes	NA	NA
Pavir.2NG328600.v4	GO:000521	NA	xanthine-ura	15.240455	10.554711	3.712912	5.925374	2.6763229	1.4202522	0.037980939	0.366866669	up	yes	Nucleobase	NAT6
Pavir.5KG771700.v4	GO:007076	NA	presenilin e	16.782557	16.180712	7.215001	5.114341	2.6735627	1.4187635	0.039964653	0.377133998	up	yes	Probable ga	At5g09310
Pavir.9NG750400.v4	NA	NA	0	5.386958	8.681481	2.293356	2.975659	2.6700321	1.4168571	0.040150416	0.377598979	up	yes	NA	NA
Pavir.1KG092100.v4	GO:000471	04075(Plan	protein kina	9.672908	14.865829	3.642061	5.553056	2.6686704	1.4161212	0.040797054	0.380028688	up	yes	PTI1-like ty	At3g15890
Pavir.7KG170600.v4	GO:000436	04120(Ubic	anaphase-pr	7.871181	15.018124	3.637516	5.033761	2.6396695	1.4003573	0.047266238	0.40946722	up	yes	NA	NA
Pavir.2NG369600.v4	GO:000550	NA	dnaj homolo	9.766005	17.75997	4.053632	6.43565	2.6242001	1.3918777	0.046468084	0.40582121	up	yes	NA	NA
Pavir.3KG336600.v4	GO:000467	04075(Plan	leucine rich	3.047569	7.2282232	1.7226354	2.1969888	2.6216269	1.3904624	0.048501822	0.41512827	up	yes	Receptor-lik	HSL1;HSL1
Pavir.8NG308000.v4	GO:005113	03040(Splic	zinc finger d	6.199088	10.878425	2.681397	3.832728	2.6216127	1.3904546	0.043973088	0.396121577	up	yes	Hsp70-Hsp	HOP
Pavir.5KG775000.v4	GO:000368	04120(Ubic	regulator of	5.221261	4.901886	2.052149	1.819909	2.61441	1.3864854	0.03864889	0.370386463	up	yes	PH, RCC1	PRAF1
Pavir.1NG308000.v4	GO:000467	04075(Plan	serine/threo	14.989515	25.101288	7.064455	8.330834	2.6040955	1.3807824	0.0404302	0.379012946	up	yes	Serine/threo	SAPK6
Pavir.7KG286700.v4	NA	NA	0	19.797827	22.415262	7.592031	8.637076	2.6010728	1.3791068	0.041227596	0.3820085	up	yes	NA	NA
Pavir.5NG076400.v4	GO:000573	00561(Glyc	gdp-fucose p	11.807465	12.982688	3.5610464	5.9927638	2.5947923	1.3756191	0.042538491	0.389038382	up	yes	Rhamnogal	RRT3;RRT4
Pavir.9KG347200.v4	GO:000386	01200(Carb	3-hydroxyis	14.976193	18.955063	4.887268	8.253478	2.5821408	1.3685677	0.045413243	0.401733783	up	yes	3-hydroxyis	CHY1
Pavir.2KG222500.v4	GO:000563	NA	thioesterase	11.840878	19.847496	5.036602	7.243083	2.5805527	1.3676801	0.044419299	0.398226279	up	yes	NA	NA
Pavir.9NG822500.v4	GO:000367	NA	uncharacteri	34.833004	41.719929	11.69708	17.991243	2.5785536	1.3665621	0.046606721	0.406300312	up	yes	NA	NA
Pavir.5KG257700.v4	GO:000563	NA	lob domain-	6.304639	13.964581	4.877051	3.056274	2.5549464	1.353293	0.04885966	0.416754602	up	yes	LOB domai	LBD41
Pavir.2NG196300.v4	GO:000367	03013(RNA	myb-like dn	25.53196	26.290979	9.732785	10.600039	2.548733	1.3497802	0.038670366	0.370386463	up	yes	Protein PHI	PHL2
Pavir.4KG069300.v4	GO:000367	NA	bsd domain	13.352291	15.688782	5.235217	6.167398	2.5468783	1.34873	0.042105503	0.386620619	up	yes	NA	NA
Pavir.5KG227400.v4	GO:000367	NA	ring finger d	9.026544	13.133924	3.953038	4.748871	2.5466214	1.3485845	0.04710193	0.408815155	up	yes	E3 ubiquiti	RFI2
Pavir.1NG232600.v4	GO:000973	NA	lob domain-	11.187187	15.862862	7.295919	3.369024	2.536352	1.342755	0.04555522	0.402456212	up	yes	LOB domai	LBD40
Pavir.6KG405500.v4	GO:000557	NA	zinc finger d	7.469567	10.424699	2.863888	4.195142	2.5349469	1.3419555	0.047284024	0.40946722	up	yes	Probable pr	PAT20;PAT20
Pavir.9NG575900.v4	GO:000573	03010(Ribo	calcium-bin	14.47513	20.277955	5.5959785	8.1607767	2.526256	1.3370009	0.046143701	0.404713246	up	yes	Putative MC	At4g17270;At4g17270;At4g17270;At4g17270;At4g17270
Pavir.9NG663100.v4	GO:000016	03040(Splic	transformer-	15.377213	16.792456	6.429265	6.487637	2.4905096	1.316441	0.044183161	0.397151649	up	yes	Serine/argin	SR45A
Pavir.8NG253700.v4	GO:000016	03013(RNA	rna recognit	24.009147	23.935752	7.9211565	11.375976	2.4845608	1.3129909	0.047214849	0.40946722	up	yes	Polyadenyla	CID8;CID8

Pavir.1NG536700.v	GO:000367	NA	zinc-finger	29.449191	26.644252	11.210012	11.452591	2.4751544	1.3075185	0.043780359	0.395232922	up	yes	FCS-Like Z	FLZ2;NA
Pavir.2KG191400.v	GO:000367	03013(RNA	myb-like dn	18.501226	23.509214	9.083364	7.894489	2.4744259	1.3070939	0.044018619	0.396121577	up	yes	Protein PHI	PHL2
Pavir.9NG819600.v	NA	NA	0	21.660624	33.324402	9.995922	12.294126	2.4667971	1.3026391	0.049775419	0.421245255	up	yes	NA	NA
Pavir.5KG769700.v	GO:000469	03022(Basa	cell divisior	18.631584	20.898676	7.53514	8.595761	2.4505922	1.2931304	0.04698147	0.408335597	up	yes	Cyclin-depe	CDKC-2
Pavir.1NG444600.v	GO:000727	00906(Carc	abscisic aci	12.726833	12.526329	5.901562	4.496067	2.4287424	1.2802095	0.046710677	0.406640471	up	yes	Abscisic aci	CYP707A5
Pavir.5KG045100.v	NA	NA	0	0.0001	0.0001	51.933453	1.83809	3.719E-06	-18.03648	4.8431E-11	4.66589E-08	down	yes	NA	NA
Pavir.9NG063000.v	GO:000367	NA	f17a17.37 p	0.0001	0.0001	46.765671	1.919289	4.108E-06	-17.89312	1.62771E-11	1.88843E-08	down	yes	NA	NA
Pavir.7KG099500.v	NA	NA	leucine-rich	0.0001	0.0001	24.969515	9.928687	5.731E-06	-17.41279	5.75701E-19	7.21027E-15	down	yes	NA	NA
Pavir.4KG134300.v	GO:000367	NA	protein of ui	0.0001	0.0001	14.572288	15.376374	6.678E-06	-17.19213	9.87797E-17	6.18575E-13	down	yes	Protein DM	DMP5
Pavir.1KG147700.v	GO:000455	04075(Plan	xyloglucan-	0.0001	0.0001	20.43708	7.910214	7.055E-06	-17.11285	2.68568E-16	1.26136E-12	down	yes	Xyloglucan	XTH14
Pavir.3KG066800.v	GO:000969	NA	nucleoporin	0.0001	0.0001	11.386144	14.587339	7.7E-06	-16.98668	3.84381E-13	9.02646E-10	down	yes	NA	NA
Pavir.9NG275700.v	GO:000557	00860(Porp	zinc finger f	0.0001	0.0001	21.825466	2.630569	8.178E-06	-16.89983	2.59346E-12	4.64019E-09	down	yes	GDSL ester	At1g71691
Pavir.3KG065700.v	NA	NA	0	0.0001	0.0001	10.573527	11.593955	9.022E-06	-16.75809	9.21097E-09	3.78213E-06	down	yes	NA	NA
Pavir.6NG292900.v	NA	NA	0	0.0001	0.0001	10.286592	10.950538	9.417E-06	-16.69623	6.92453E-12	1.0407E-08	down	yes	NA	NA
Pavir.9KG439800.v	GO:001602	00909(Sesq	premnaspir	0.0001	0.0001	16.622819	3.408138	9.985E-06	-16.61187	8.02827E-14	2.32036E-10	down	yes	Premnaspir	CYP71D55;CYP71D55
Pavir.7KG068000.v	GO:000716	NA	ankyrin repe	0.0001	0.0001	13.715606	3.460068	1.164E-05	-16.39001	3.84555E-14	1.20407E-10	down	yes	Protein AC	ACD6;ACD6
Pavir.8KG361700.v	GO:000716	NA	domain of u	0.0001	0.0001	7.972825	7.98943	1.253E-05	-16.2843	4.82967E-18	3.6293E-14	down	yes	NA	NA
Pavir.5KG716200.v	NA	NA	0	0.0001	0.0001	11.02653	4.7756716	1.266E-05	-16.26977	3.81992E-10	2.70803E-07	down	yes	NA	NA
Pavir.6NG111500.v	NA	NA	0	0.0001	0.0001	10.126599	4.541412	1.364E-05	-16.16231	4.44333E-12	6.95622E-09	down	yes	NA	NA
Pavir.2KG376700.v	GO:000455	00500(Starc	endoglucan	0.0001	0.0001	12.464006	2.092446	1.374E-05	-16.1513	8.8108E-12	1.2261E-08	down	yes	Endoglucan	GLU12
Pavir.7NG111100.v	GO:000588	NA	plant protei	0.0001	0.0001	7.9333451	6.0495218	1.43E-05	-16.0933	7.4083E-16	3.0928E-12	down	yes	NA	NA
Pavir.3NG119500.v	NA	NA	0	0.0001	0.0001	10.835597	2.033285	1.554E-05	-15.97353	1.01251E-07	2.69809E-05	down	yes	NA	NA
Pavir.2KG033400.v	GO:000467	04626(Plan	leucine-rich	0.0001	0.0001	8.011566	4.631966	1.582E-05	-15.94804	2.21346E-18	2.07916E-14	down	yes	LRR recept	GHR1
Pavir.6NG156500.v	NA	NA	0	0.0001	0.0001	9.388081	3.071083	1.605E-05	-15.92685	8.08279E-11	7.23082E-08	down	yes	Auxin resp	ARF2
Pavir.9NG359600.v	GO:000573	00780(Biot	8-amino-7-	0.0001	0.0001	4.7686767	7.328027	1.653E-05	-15.88425	8.08634E-13	1.5991E-09	down	yes	8-amino-7-	BIOF;BIOF
Pavir.2KG078000.v	GO:000460	00564(Glyc	phosphatidy	0.0001	0.0001	6.651996	3.560989	1.958E-05	-15.64005	1.71114E-11	1.88843E-08	down	yes	Lecithin-ch	Os03g0232800
Pavir.1KG175300.v	GO:000367	04141(Prot	x-box trans	0.0001	0.0001	5.624258	3.853827	2.11E-05	-15.53231	2.14637E-09	1.20366E-06	down	yes	Basic leucir	BZIP19
Pavir.5KG611000.v	GO:000557	00561(Glyc	zinc finger f	0.0001	0.0001	5.046268	4.348038	2.129E-05	-15.5195	2.83948E-11	2.88346E-08	down	yes	GDSL ester	At1g58430
Pavir.3KG389000.v	NA	NA	0	0.0001	0.0001	7.660412	1.082999	2.287E-05	-15.41591	1.7947E-08	6.24372E-06	down	yes	NA	NA
Pavir.3NG076300.v	NA	NA	far1 dna-bir	0.0001	0.0001	4.1055271	4.5238959	2.318E-05	-15.39698	4.25968E-13	9.41464E-10	down	yes	NA	NA
Pavir.8NG329900.v	GO:000716	NA	domain of u	0.0001	0.0001	4.413712	4.180814	2.327E-05	-15.39113	8.68778E-11	7.5913E-08	down	yes	NA	NA
Pavir.2KG358000.v	GO:000367	03013(RNA	genomic dn	0.0001	0.0001	6.086873	2.243496	2.401E-05	-15.34609	7.61553E-10	4.8498E-07	down	yes	NAC doma	NAC086
Pavir.7NG297400.v	GO:000425	04144(Endo	subtilisin se	0.0001	0.0001	6.643887	1.431295	2.477E-05	-15.30121	7.7535E-11	7.10542E-08	down	yes	Subtilisin-li	SBT1.3
Pavir.1KG300700.v	NA	NA	0	0.0001	0.0001	3.778568	3.90135	2.604E-05	-15.2288	2.79484E-09	1.4543E-06	down	yes	Pollen-spec	PEX2
Pavir.5KG724300.v	GO:000557	NA	epidermal p	0.0001	0.0001	4.6647504	2.5055934	2.789E-05	-15.12975	1.72113E-08	6.04375E-06	down	yes	EPIDERMA	EPFL9;EPFL9
Pavir.5NG492700.v	GO:000097	NA	transcriptio	0.0001	0.0001	5.656456	1.478037	2.803E-05	-15.12252	7.76964E-10	4.86548E-07	down	yes	GATA trans	GATA9
Pavir.5NG615500.v	GO:000557	00960(Trop	nad(p)-bind	0.0001	0.0001	3.987602	2.768852	2.96E-05	-15.04398	3.01127E-09	1.50857E-06	down	yes	Tropinone r	At1g07440
Pavir.5KG564800.v	GO:000450	00350(Tyro	tyrosinase//	0.0001	0.0001	4.98335	1.357103	3.154E-05	-14.9523	3.18855E-10	2.30391E-07	down	yes	Polyphenol	-
Pavir.3KG439600.v	NA	NA	0	0.0001	0.0001	2.544397	3.718097	3.194E-05	-14.93445	9.26936E-09	3.78213E-06	down	yes	NA	NA
Pavir.5KG170700.v	GO:000449	00906(Carc	cytochrome	0.0001	0.0001	2.56288	3.252748	3.439E-05	-14.82765	2.04622E-10	1.67136E-07	down	yes	Taxadiene 5	-
Pavir.2KG256900.v	NA	NA	0	0.0001	0.0001	3.572376	2.20474	3.462E-05	-14.81806	3.17729E-07	6.86094E-05	down	yes	NA	NA
Pavir.8NG044100.v	GO:000367	NA	protein of ui	0.0001	0.0001	3.614067	1.688763	3.772E-05	-14.69447	1.94827E-09	1.10912E-06	down	yes	Protein DM	DMP9
Pavir.7KG421500.v	GO:000194	NA	wuschel-rel	0.0001	0.0001	4.1696925	1.0374834	3.841E-05	-14.66821	5.25064E-08	1.56424E-05	down	yes	WUSCHEL	WOX4;WOX4
Pavir.2KG051200.v	GO:000455	00500(Starc	carbohydrat	0.0001	0.0001	3.836478	1.310172	3.886E-05	-14.65135	2.23293E-07	5.16019E-05	down	yes	Glucan end	At5g56590
Pavir.3NG058100.v	NA	NA	bomb/kira p	0.0001	0.0001	2.116962	2.913373	3.976E-05	-14.61837	4.68689E-10	3.20183E-07	down	yes	NA	NA
Pavir.5NG158300.v	GO:000557	00561(Glyc	zinc finger f	0.0001	0.0001	2.388159	2.585227	4.021E-05	-14.60194	2.0386E-07	4.81737E-05	down	yes	GDSL ester	At1g33811
Pavir.2NG508600.v	NA	NA	0	0.0001	0.0001	1.876827	3.040225	4.067E-05	-14.58551	6.17183E-07	0.000117118	down	yes	NA	NA
Pavir.7NG166400.v	NA	NA	0	0.0001	0.0001	3.28428	1.590491	4.103E-05	-14.57305	6.56613E-11	6.16773E-08	down	yes	NA	NA
Pavir.6NG159400.v	GO:000419	NA	aspartyl pro	0.0001	0.0001	1.960033	2.407217	4.58E-05	-14.41444	2.50504E-09	1.3446E-06	down	yes	Aspartyl pr	At5g10770
Pavir.9NG776700.v	GO:000563	NA	max dimeriz	0.0001	0.0001	1.726902	2.497384	4.735E-05	-14.36642	5.87296E-09	2.62696E-06	down	yes	Transcriptio	BHLH70
Pavir.5NG493500.v	GO:000367	01200(Carb	glyceraldeh	0.0001	0.0001	2.367647	1.767038	4.837E-05	-14.33549	2.81738E-08	9.20498E-06	down	yes	Glyceraldeh	GAPC3
Pavir.2NG439100.v	GO:000981	01210(2-Ox	pathogen-in	0.0001	0.0001	1.682189	2.383465	4.919E-05	-14.3112	1.03718E-08	4.05936E-06	down	yes	UDP-glucos	-
Pavir.9NG817300.v	GO:000557	NA	outer arm d	0.0001	0.0001	2.502351	1.2727059	5.298E-05	-14.20421	6.7112E-09	2.93209E-06	down	yes	NA	NA
Pavir.2NG374600.v	GO:000842	00500(Starc	glycosyl hyc	0.0001	0.0001	2.278506	1.375218	5.474E-05	-14.15708	3.13171E-07	6.8016E-05	down	yes	Beta-glucos	BGLU29
Pavir.4KG150900.v	GO:000716	NA	ankyrin repe	0.0001	0.0001	1.377668	2.161512	5.651E-05	-14.11113	4.20974E-09	2.02786E-06	down	yes	NA	NA
Pavir.8KG083400.v	GO:000367	NA	protein of ui	0.0001	0.0001	2.089465	1.42348	5.693E-05	-14.10039	4.19415E-08	1.30237E-05	down	yes	Protein DM	DMP10;DMP4
Pavir.2NG279100.v	GO:000716	NA	domain of u	0.0001	0.0001	1.5719468	1.9112295	5.742E-05	-14.08812	6.45663E-10	4.18267E-07	down	yes	NA	NA
Pavir.1NG058900.v	GO:004665	NA	plastocyanir	0.0001	0.0001	2.460663	0.998071	5.782E-05	-14.07796	5.75815E-07	0.000110383	down	yes	Early nodul	At4g27520
Pavir.8NG330200.v	GO:000716	NA	domain of u	0.0001	0.0001	1.709142	1.514574	6.204E-05	-13.97644	5.82097E-08	1.68239E-05	down	yes	NA	NA
Pavir.6KG365300.v	NA	NA	0	0.0001	0.0001	1.501966	1.662682	6.32E-05	-13.94976	1.37883E-07	3.52427E-05	down	yes	NA	NA
Pavir.3NG005400.v	NA	NA	0	0.0001	0.0001	2.026808	1.079235	6.439E-05	-13.92279	5.85256E-07	0.000111623	down	yes	Inner memb	ALB3
Pavir.2KG358100.v	GO:000367	03013(RNA	no apical m	0.0001	0.0001	1.286558	1.816783	6.445E-05	-13.92153	3.59743E-07	7.63652E-05	down	yes	NAC doma	NAC086
Pavir.7KG096400.v	GO:000367	04144(Endo	protein of ui	0.0001	0.0001	1.71162	1.224653	6.811E-05	-13.8417	3.23014E-09	1.59692E-06	down	yes	NA	NA
Pavir.1KG148100.v	NA	NA	0	0.0001	0.0001	1.426975	1.322847	7.273E-05	-13.74705	3.05958E-07	6.72267E-05	down	yes	NA	NA
Pavir.4KG128000.v	GO:000716	NA	domain of u	0.0001	0.0001	1.421493	1.314776	7.309E-05	-13.73992	3.54627E-07	7.57067E-05	down	yes	NA	NA
Pavir.4NG235500.v	GO:000716	NA	domain of u	0.0001	0.0001	1.058001	1.556353	7.65E-05	-13.67417	2.4052E-07	5.444E-05	down	yes	NA	NA
Pavir.8NG164700.v	GO:000467	04626(Plan	protein kina	0.0001	0.0001	1.328115	1.191857	7.937E-05	-13.62112	3.13839E-08	1.00785E-05	down	yes	Putative dis	RGA3
Pavir.5KG301800.v	GO:000014	NA	lyst-interact	0.0001	0.0001	1.19183	1.277924	8.098E-05	-13.59208	5.30555E-09	2.46105E-06	down	yes	Putative cal	CALS6
Pavir.2KG309000.v	NA	NA	0	0.0001	0.0001	1.299667	0.995162	8.715E-05	-13.4861	1.45633E-07	3.64792E-05	down	yes	NA	NA
Pavir.3NG318300.v	NA	NA	0	0.0001	0.050538	149.72215	3.074901	0.0003314	-11.55911	1.39409E-08	5.08544E-06	down	yes	NA	NA
Pavir.7NG021800.v	NA	NA	0	0.0001	0.126852	208.31232	11.703882	0.000577	-10.75911	7.46605E-09	3.15193E-06	down	yes	NA	NA
Pavir.1KG388800.v	GO:000557	NA	gamma-thio	0.10304	0.0001	140.3022	6.39361	0.0007031	-10.47401	6.28516E-09	2.77826E-06	down	yes	Defensin-lil	CAL1
Pavir.5NG565100.v	GO:000521	NA	non-specific	0.07823	0.13502	254.5697	14.263372	0.0007932	-10.29995	2.09612E-10	1.67569E-07	down	yes	Non-specifi	-
Pavir.9KG259300.v	NA	04141(Prot	small heat-s	0.0001	0.064879	35.038303	29.397444	0.0010084	-9.953672	5.00803E-15	1.88167E-11	down	yes	NA	NA
Pavir.2KG387500.v	GO:000577	00531(Glyc	aquaporin t	0.037139	0.0001	18.129419	11.994572	0.0012362	-9.659883	1.66777E-13	4.47595E-10	down	yes	Probable aq	PIP2-7
Pavir.9KG578500.v	GO:000436	00480(Glut	glutathione	0.087661	0.125441	97.111076	40.45034	0.0015491	-9.334316	6.73275E-15	2.29972E-11	down	yes	Glutathione	GSTF11
Pavir.8KG095700.v	GO:000551	00500(Starc	beta-glucosi	0.029654	0.299096	90.559883	113.10537	0.0016142	-9.274993	1.22659E-11	1.58919E-08	down	yes	Avenacosid	P60B
Pavir.9NG275400.v	GO:000557	00860(Porp	zinc finger f	0.053377	0.0001	29.504078	3.623707	0.0016143	-9.274907	2.82553E-09	1.4543E-06	down	yes	GDSL ester	At1g71691
Pavir.7NG369600.v	GO:000455	04075(Plan	xyloglucan	0.0001	0.040931	22.158064	1.806927	0.0017121	-9.189998	6.56502E-08	1.86869E-05	down	yes	Xyloglucan	XTH9
Pavir.1NG358200.v	GO:000557	NA	gamma-thio	0.4750133	0.102133	327.66812	8.8876015	0.0017149	-9.187693	1.483E-07	3.69011E-05	down	yes	Defensin-lil	CAL1;CAL1
Pavir.4NG281100.v	GO:000557	NA	thaumatin f	0.147863	0.042914	73.43541	11.675236	0.0022415	-8.801309	1.72939E-11	1.88843E-08	down	yes	Thaumatin-	At1g18250
Pavir.7NG345300.v	GO:000557	00561(Glyc	zinc finger f	0.046358	0.0001	17.465345	2.653696	0.0023092	-8.758419	2.18309E-08	7.2639E-06	down	yes	GDSL ester	LTL1

Pavir.3KG065600.v4	GO:000969	04626(Plan	kelch repeat	0.0001	0.139428	31.457907	28.90469	0.0023115	-8.756956	1.90489E-10	1.5905E-07	down	yes	Protein GO	GOS9
Pavir.1NG393700.v4	GO:000662	NA	predicted m	0.0001	0.0577022	12.066183	10.775501	0.0025306	-8.62633	4.9977E-10	3.35319E-07	down	yes	NA	NA
Pavir.9KG578600.v4	GO:000436	00480(Glut	glutathione	0.886646	0.200809	239.11311	183.54497	0.0025729	-8.602392	4.22032E-12	6.89436E-09	down	yes	Glutathione	GST3
Pavir.8NG085000.v4	NA	NA	0	0.04347	0.0001	3.344739	12.305346	0.002784	-8.48862	3.87184E-08	1.21231E-05	down	yes	23 kDa jasr	-
Pavir.2NG025000.v4	GO:000716	NA	domain of u	0.014027	0.0001	1.838922	2.966759	0.0029396	-8.410142	7.04441E-09	3.00772E-06	down	yes	NA	NA
Pavir.7KG417600.v4	GO:000367	NA	hydrophobic	0.063579	0.0001	8.782001	12.799502	0.0029506	-8.404762	5.52026E-09	2.49895E-06	down	yes	Putative lip	AIR1B
Pavir.5KG250900.v4	GO:000557	NA	thaumatin fa	0.348033	0.178159	145.65617	26.113754	0.0030634	-8.350672	7.66847E-12	1.10818E-08	down	yes	Thaumatin-	At1g18250
Pavir.2KG212500.v4	GO:000561	04144(Endo	cupin (cupir	0.0001	0.044179	2.147584	11.371149	0.0032754	-8.254122	4.41359E-07	8.96387E-05	down	yes	Putative ger	Os03g0651800
Pavir.2NG203100.v4	GO:000367	NA	f17a17.37 p	0.385334	0.0001	104.47031	8.302808	0.0034178	-8.192724	1.24061E-05	0.001635555	down	yes	NA	NA
Pavir.8NG025100.v4	GO:000716	NA	domain of u	0.0323498	0.0001	2.6195996	6.3421825	0.0036209	-8.109434	1.06345E-08	4.07723E-06	down	yes	NA	NA
Pavir.3KG024500.v4	GO:000467	04075(Plan	protein kina	0.047487	0.0001	9.355347	2.540251	0.0040004	-7.965645	4.71907E-08	1.44154E-05	down	yes	Leucine-ricl	PXC1
Pavir.1NG509300.v4	GO:000950	01212(Fatty	3-oxoacyl- (	0.018315	0.0001	2.710919	1.845265	0.0040418	-7.950801	2.76627E-07	6.15013E-05	down	yes	NADPH-de	At3g03980
Pavir.9NG767700.v4	GO:000436	00480(Glut	glutathione	1.409534	0.185011	229.79871	133.31307	0.0043913	-7.831125	1.68E-09	9.86292E-07	down	yes	Glutathione	GSTF11
Pavir.6NG348800.v4	NA	NA	0	0.142514	0.0001	19.784125	11.790091	0.0045168	-7.790487	1.31286E-08	4.88398E-06	down	yes	NA	NA
Pavir.9KG621100.v4	NA	NA	0	0.024961	0.0001	2.003235	3.366741	0.0046669	-7.743328	1.99479E-07	4.74369E-05	down	yes	NA	NA
Pavir.8KG183800.v4	NA	NA	0	0.04136	0.0001	6.556473	2.31464	0.0046736	-7.741251	4.70998E-07	9.31411E-05	down	yes	Protein GL2	GIR1
Pavir.9NG428300.v4	GO:000573	00592(alph	transferase f	0.048576	0.0001	6.605308	2.924511	0.0051078	-7.613094	1.51257E-07	3.73895E-05	down	yes	Acyl transfe	AT1
Pavir.2NG327900.v4	GO:000551	04075(Plan	cyclin-d1-1	0.271803	0.132861	62.2291	14.363138	0.0052834	-7.56433	2.47305E-10	1.89633E-07	down	yes	Cyclin-D2-	CYCD2-1;CYCD2-1
Pavir.3NG088900.v4	GO:000557	00561(Glyc	zinc finger f	0.0001	0.139653	22.248203	3.528688	0.0054216	-7.527055	1.72211E-06	0.000288861	down	yes	GDSL ester	LTL1
Pavir.5NG162400.v4	NA	NA	0	0.0001	0.291172	44.502823	5.877803	0.0057814	-7.434358	5.10411E-06	0.000758011	down	yes	MFS18 pro	MFS18
Pavir.1NG509000.v4	NA	NA	0	0.336387	0.086885	51.155979	21.900732	0.0057937	-7.431288	1.76508E-11	1.88843E-08	down	yes	Protein CDI	CDI
Pavir.1NG093300.v4	GO:000367	NA	protein of u	0.028726	0.0001	1.779474	2.866213	0.0062049	-7.332377	1.20104E-06	0.000209058	down	yes	NA	NA
Pavir.1KG542400.v4	GO:000557	00561(Glyc	zinc finger f	2.525006	0.200063	166.228	267.94122	0.0062765	-7.315821	1.02765E-07	2.71915E-05	down	yes	GDSL ester	At5g33370
Pavir.9KG238900.v4	NA	NA	protein of u	0.05212	0.020073	7.912594	3.30906	0.0064334	-7.280211	1.06346E-09	6.55038E-07	down	yes	NA	NA
Pavir.9KG480500.v4	GO:000016	03040(Splic	predicted rn	0.269505	0.0001	35.140083	6.65273	0.006451	-7.276264	4.72305E-06	0.000712687	down	yes	Probable RI	ARP1
Pavir.8NG138700.v4	GO:000418	00460(Cyar	hydroxymar	0.0001	0.0506265	3.406684	4.3959732	0.0065012	-7.265083	4.68665E-07	9.31411E-05	down	yes	P-(S)-hydro	-;-;-
Pavir.8KG254600.v4	GO:000563	00040(Pent	helix-loop-h	0.08446	0.024339	10.630689	6.079177	0.0065111	-7.262891	2.59448E-10	1.94965E-07	down	yes	Transcriptic	SCRM
Pavir.2NG339200.v4	GO:000371	04075(Plan	jasmonate z	0.1600806	0.0001	16.324117	6.9385618	0.0068857	-7.182174	9.08694E-07	0.000162583	down	yes	Protein TIF	TIFY10C;TIFY10C
Pavir.2NG374800.v4	GO:000842	00500(Starc	glycosyl hyd	0.057394	0.106304	13.805787	9.21498	0.0071109	-7.135755	1.29043E-11	1.59116E-08	down	yes	Beta-glucos	BGLU30
Pavir.7NG098300.v4	GO:000367	04144(Endo	domain of u	0.064723	0.0001	4.940765	4.0179	0.0072358	-7.110634	4.83501E-07	9.5113E-05	down	yes	NA	NA
Pavir.7KG088700.v4	GO:000367	NA	protein of u	0.064427	0.0001	5.656944	3.226457	0.0072638	-7.105065	4.30527E-06	0.000660252	down	yes	NA	NA
Pavir.9KG381600.v4	GO:000449	00903(Lim	cytochrome	0.0001	0.076097	7.61561	2.266989	0.0077102	-7.019012	4.11734E-06	0.00063915	down	yes	Cytochrome	CYP94A2
Pavir.6NG088100.v4	GO:190170	00350(Tyrc	polyphenol	0.080007	0.023056	11.201378	2.140085	0.007725	-7.016247	7.36607E-08	2.03504E-05	down	yes	Polyphenol	-
Pavir.9NG695000.v4	GO:000079	04070(Phos	histone h2a	0.506029	0.755051	159.68309	2.301065	0.0077852	-7.005049	1.50657E-05	0.001912377	down	yes	Histone H2	-
Pavir.4KG308800.v4	GO:000588	00561(Glyc	diacylglycer	0.074099	0.0001	8.174517	1.258259	0.0078661	-6.990139	1.99468E-05	0.002441246	down	yes	Lipid phosp	LPP2
Pavir.2NG539300.v4	NA	NA	plant protei	0.0206683	0.0001	1.0175459	1.6088497	0.0079075	-6.982556	4.69234E-06	0.000710909	down	yes	NA	NA
Pavir.1NG509200.v4	GO:000551	00940(Phen	shikimate o-	0.12585	0.0001	11.008813	3.727469	0.0085469	-6.870378	3.5522E-06	0.000558438	down	yes	Putrescine h	PHT1
Pavir.8KG184000.v4	GO:000367	00970(Ami	transcriptio	0.113772	0.067292	13.956752	7.210851	0.0085538	-6.869214	2.66417E-10	1.96276E-07	down	yes	Transcriptic	OFP8
Pavir.2NG070200.v4	GO:000551	00730(Thia	1-deoxy-d-x	0.0309488	0.0001	2.152543	1.4540812	0.0086088	-6.859969	2.69641E-06	0.000436691	down	yes	Probable 1-	Os07g0190000;Os07g0190000
Pavir.9NG676600.v4	GO:000369	04145(Phag	lon-like atp-	0.070547	0.050574	6.048962	7.719901	0.0087967	-6.828817	1.11216E-11	1.4924E-08	down	yes	Lon proteas	Os07g0689300
Pavir.6NG197900.v4	GO:000588	00190(Oxic	h+-transpor	0.070211	0.016186	4.9009801	4.7530548	0.0089493	-6.804007	2.29941E-09	1.25718E-06	down	yes	Plasma mer	Os04g0656100;Os04g0656100
Pavir.7KG181400.v4	NA	NA	0	1.08535	0.256816	121.08711	18.144554	0.0096398	-6.69678	1.64264E-07	4.03392E-05	down	yes	NA	NA
Pavir.7KG125900.v4	NA	NA	hsp20/alpha	1.191013	0.135675	54.760391	82.842377	0.0096414	-6.696537	1.08951E-08	4.09363E-06	down	yes	NA	NA
Pavir.1NG477400.v4	GO:000557	04626(Plan	lysm domai	0.078358	0.0001	6.7274052	1.374367	0.0096841	-6.690173	2.40068E-05	0.00285446	down	yes	LysM doma	LYP6;LYP6
Pavir.4KG016400.v4	GO:000419	NA	aspartyl pro	0.148318	0.0001	3.672228	11.54342	0.0097543	-6.679746	9.45705E-06	0.001316036	down	yes	Aspartyl pr	At5g10770
Pavir.9NG032200.v4	GO:000521	NA	xanthine-ura	0.0577	0.024312	7.123811	1.238651	0.0098072	-6.671949	1.08858E-06	0.000192024	down	yes	Nucleobase	NAT6
Pavir.2KG351900.v4	GO:000588	04626(Plan	leucine-rich	0.05585	0.0001	2.9108884	2.6051673	0.0101431	-6.623355	5.26175E-06	0.000778346	down	yes	Leucine-ricl	At2g14510;At2g14510
Pavir.6NG171100.v4	GO:000981	00906(Carc	glucosyl/glu	0.052794	0.029996	5.30144	2.7677953	0.01026	-6.606832	1.29536E-07	3.3566E-05	down	yes	UDP-glycos	UGT73E1;UGT73E1
Pavir.7KG245800.v4	GO:000557	00561(Glyc	zinc finger f	0.161809	0.0001	14.509668	1.161877	0.0103314	-6.59682	0.000117539	0.009559058	down	yes	GDSL ester	LTL1
Pavir.9NG359200.v4	GO:000377	04626(Plan	formin-relat	0.0001	0.06681	4.312538	2.075661	0.010474	-6.577044	1.25855E-05	0.001647645	down	yes	Formin-like	FH4
Pavir.2KG293100.v4	GO:000003	00062(Fatty	3-ketoacyl-c	0.050782	0.0001	2.766787	2.000703	0.0106727	-6.549931	1.44885E-05	0.001845338	down	yes	3-ketoacyl-(	KCS17
Pavir.5NG495700.v4	GO:001629	00561(Glyc	zinc finger f	0.057095	0.032906	2.362611	5.90875	0.010881	-6.52204	3.69378E-07	7.79699E-05	down	yes	GDSL ester	At5g45670
Pavir.3KG102600.v4	GO:000961	NA	potato inhib	0.0001	0.0580036	3.2147534	2.0584803	0.0110186	-6.503918	2.3548E-05	0.002817741	down	yes	Subtilisin-c	-;-
Pavir.9NG767800.v4	GO:000436	00480(Glut	glutathione	2.686483	0.17306	184.00153	71.00956	0.0112134	-6.478632	2.04766E-06	0.000338928	down	yes	Glutathione	GSTF11
Pavir.7KG247900.v4	GO:000588	NA	plant protei	0.0766671	0.0001	3.6722807	3.1696506	0.0112201	-6.477772	1.03315E-05	0.001405436	down	yes	NA	NA
Pavir.7KG126700.v4	GO:000028	00900(Terp	farnesyl dip	1.552471	0.233534	124.09786	33.874607	0.0113058	-6.466793	4.20886E-07	8.66429E-05	down	yes	Acyclic sesc	TPS1
Pavir.1NG510900.v4	GO:000551	NA	dipeptidyl p	0.018029	0.053423	4.012284	2.274075	0.0113662	-6.459106	3.12614E-07	6.8016E-05	down	yes	Nudix hydro	NUDT3
Pavir.7KG372400.v4	GO:000455	00500(Starc	glucosidase-	0.184169	0.0001	7.617219	8.452671	0.0114667	-6.446403	9.09385E-06	0.001270198	down	yes	Glucan endo	GLC1
Pavir.2NG347700.v4	GO:000578	NA	receptor exp	0.064907	0.0001	4.050728	1.613313	0.0114771	-6.445093	3.86379E-05	0.004124271	down	yes	Protein HV	HVA22
Pavir.8NG053900.v4	NA	NA	0	0.0001	0.056835	3.578012	1.38035	0.0114826	-6.444404	4.77818E-05	0.004959411	down	yes	NA	NA
Pavir.2NG222400.v4	GO:000561	04144(Endo	cupin doma	0.145813	0.0001	1.121595	11.276213	0.0117693	-6.408833	0.000189622	0.013590638	down	yes	Germin-like	GER8
Pavir.8KG053100.v4	GO:000367	NA	protein of u	0.0001	0.062397	4.077946	1.195481	0.0118513	-6.39881	5.81776E-05	0.005739215	down	yes	Protein DM	DMP9
Pavir.1KG250100.v4	GO:004746	00909(Sesq	beta-sesquip	0.315211	0.0001	16.294775	10.100474	0.0119457	-6.387359	1.43231E-05	0.001836998	down	yes	Alpha-terpi	TPS26
Pavir.1KG496500.v4	NA	NA	0	0.1046449	0.6135462	50.823525	8.9055022	0.0120242	-6.377921	6.98174E-07	0.000128591	down	yes	NA	NA
Pavir.9NG640900.v4	NA	NA	0	0.206954	0.028165	6.907912	12.295169	0.0122438	-6.351803	9.33044E-08	2.54038E-05	down	yes	NA	NA
Pavir.4NG005500.v4	GO:000550	00053(Asco	multi-coppe	0.228461	0.0001	14.963578	3.644126	0.0122831	-6.347177	6.05454E-05	0.00590876	down	yes	Monocoppe	SKU5
Pavir.4KG306400.v4	GO:000588	NA	mlo protein	0.034907	0.0001	1.134314	1.680985	0.0124346	-6.329501	3.86102E-05	0.004124271	down	yes	MLO-like p	MLO13
Pavir.8KG344300.v4	NA	NA	rrna n-glyco	0.677042	0.077058	23.332815	36.297787	0.0126462	-6.305153	7.26706E-08	2.02256E-05	down	yes	NA	NA
Pavir.3KG067600.v4	GO:000468	04626(Plan	serine/threo	0.063921	0.0001	3.855044	1.124991	0.0128555	-6.281467	6.16649E-05	0.006002423	down	yes	Calcium-de	CPK29
Pavir.7KG263600.v4	GO:003152	00902(Mon	short chain	0.050687	0.115366	9.400305	3.469446	0.0129026	-6.276197	3.75139E-08	1.1945E-05	down	yes	(-)-isopiper	-
Pavir.4NG289500.v4	GO:000563	04140(Regu	beta catenin	0.040876	0.0001	1.324688	1.848164	0.0129146	-6.274857	2.92684E-05	0.003292523	down	yes	NA	NA
Pavir.5KG603800.v4	GO:000521	NA	non-specific	1.729011	0.094022	133.97688	6.179142	0.0130072	-6.264549	0.000117059	0.009559058	down	yes	Non-specifi	-
Pavir.9KG254700.v4	GO:000982	00073(Cuti	transferase f	0.087771	0.0001	4.179804	2.563459	0.0130309	-6.261916	2.61173E-05	0.003019405	down	yes	Acyl transfe	AT1
Pavir.9KG618200.v4	GO:000455	00500(Starc	glucan endo	0.157275	0.072464	14.735152	2.815446	0.0130901	-6.255381	1.41704E-07	3.59747E-05	down	yes	Glucan endo	At2g01630
Pavir.7NG154900.v4	GO:000484	NA	inhibitor of	0.0001	0.043951	2.213279	1.143679	0.0131223	-6.251836	7.92447E-05	0.007297699	down	yes	Probable BC	BRG3
Pavir.7NG245900.v4	NA	NA	extensin, pr	0.344712	0.299077	41.923206	6.07363	0.0134132	-6.220208	9.87633E-07	0.000175869	down	yes	NA	NA
Pavir.9NG688300.v4	NA	NA	0	0.782199	0.0001	43.983238	13.738568	0.0135529	-6.205253	5.59951E-05	0.005580645	down	yes	NA	NA
Pavir.3NG013100.v4	GO:000467	04075(Plan	protein kina	0.100196	0.119507	11.590833	4.31008	0.013817	-6.177411	2.87424E-09	1.45938E-06	down	yes	Leucine-ricl	PXC1
Pavir.6NG288400.v4	GO:000367	04144(Endo	protein of u	0.056509	0.0001	1.182844	2.814664	0.0141611	-6.141926	5.91606E-05	0.005803764	down	yes	NA	NA
Pavir.8KG282400.v4	GO:000367	NA	plant protei	0.102953	0.0001	3.999778	3.232402	0.0142492	-6.132972	2.82907E-05	0.003221112	down	yes	UPF0481 p	At3g47200
Pavir.5NG266200.v4	GO:000552	03050(Prot	mitochondri	0.069977	0.0001	3.333815	1.545825	0.0143611	-6.12169	5.48908E-05	0.005499769	down	yes	AAA-ATPa	At4g25835
Pavir.1KG497800.v4	GO:004665	NA	plastocyanir	0.047081	0.0001	1.793773	1.485757	0.0143865	-6.119139	5.22197E-05	0.005317207	down	yes	Chemocyan	-

Pavir.1KG408500.v	GO:000694	NA	pollen aller	0.2828191	0.0001	13.712758	5.6326073	0.0146246	-6.095455	9.23747E-05	0.008185832	down	yes	Expansin-B	EXPB16;EXPB16
Pavir.3KG029100.v	GO:000487	NA	late embryo	0.097426	0.0001	2.354879	4.294247	0.0146675	-6.091234	6.53659E-05	0.006265293	down	yes	NA	NA
Pavir.9KG077900.v	GO:000551	NA	iq calmodul	0.133286	0.0001	4.413546	4.48836	0.014984	-6.060435	4.42016E-05	0.004639066	down	yes	Protein IQ-l	IQD31
Pavir.4KG391700.v	GO:000563	NA	beta catenin	0.160909	0.0001	4.133442	6.485658	0.0151622	-6.043376	5.11337E-05	0.005235004	down	yes	NA	NA
Pavir.2KG142000.v	GO:000551	NA	ribosome in	0.333959	0.0001	15.298331	6.607539	0.0152497	-6.035071	0.000106808	0.008976715	down	yes	Ricin OS=F	-
Pavir.5KG332900.v	GO:00071€	NA	ankyrin repe	0.06126	0.0001	1.058396	2.952481	0.0152984	-6.030475	0.000101661	0.008665437	down	yes	Protein AC	ACD6
Pavir.1NG058800.v	GO:004665	NA	plastocyanin	0.138207	0.0001	3.347489	5.430324	0.0157564	-5.987915	5.15199E-05	0.00526021	down	yes	Early nodul	At4g27520
Pavir.2KG103300.v	GO:000551	00730(Thia	1-deoxy-d-x	0.113732	0.0001	4.774876	2.330526	0.0160205	-5.963938	7.33387E-05	0.006899584	down	yes	Probable 1-	Os07g0190000
Pavir.1KG400200.v	GO:000578	00073(Cuti	protein cer1	0.054042	0.0001	2.270216	1.036466	0.0163735	-5.932492	9.3142E-05	0.008215084	down	yes	Very-long-c	GL1-4
Pavir.2NG260900.v	NA	NA	0	0.093682	0.0001	3.185692	2.513271	0.016456	-5.925245	8.23094E-05	0.007506338	down	yes	NA	NA
Pavir.9KG578700.v	GO:000436	00480(Glut	glutathione	2.74548	0.259078	79.598801	101.01536	0.0166352	-5.909614	2.38248E-06	0.000389204	down	yes	Glutathione	GSTF10
Pavir.7NG155700.v	NA	NA	0	0.131288	0.0001	3.918902	3.97099	0.0166527	-5.9081	6.28251E-05	0.006068197	down	yes	NA	NA
Pavir.7KG312500.v	GO:000013	00500(Star	cellulose sy	0.335511	0.0001	10.307652	9.839244	0.0166582	-5.907624	0.000132136	0.010452082	down	yes	Cellulose sy	CSLH1
Pavir.4KG391100.v	GO:000663	01212(Fatty	acyl- acyl-ca	0.273899	0.0001	10.073493	6.308116	0.016726	-5.901763	8.43737E-05	0.007657425	down	yes	Acyl-[acyl-c	Os03g0423300
Pavir.6KG291400.v	GO:000563	00966(Gluc	sulfotransfe	0.0001	0.075375	2.809351	1.646728	0.0169375	-5.883632	0.000110932	0.009221422	down	yes	Cytosolic su	SOT5
Pavir.3KG400200.v	NA	NA	0	0.109804	0.0001	3.5505069	2.811044	0.0172763	-5.855063	8.89773E-05	0.007955435	down	yes	NA	NA
Pavir.7KG259600.v	GO:000694	NA	expansin-b1	0.2046036	0.0512922	13.255698	1.0666376	0.0178669	-5.806566	2.90572E-05	0.003278573	down	yes	Expansin-B	EXPB17;EXPB17;EXPB17
Pavir.3KG104900.v	GO:00007¢	04070(Phos	histone h2a	1.438966	1.330436	150.44153	3.390109	0.0180028	-5.795634	5.11052E-05	0.005235004	down	yes	Probable hi	OsI_017523
Pavir.9KG271500.v	GO:00094(	04141(Prot	small heat-s	1.441774	0.520572	74.79705	33.713818	0.0180843	-5.789116	6.86722E-09	2.96577E-06	down	yes	26.7 kDa h	HSP26.7
Pavir.1NG202600.v	GO:000455	04075(Plan	xyloglucan-	0.1229816	0.0565022	5.0782618	4.6991292	0.018357	-5.767525	1.06004E-08	4.07723E-06	down	yes	Xyloglucan	XTH14;XTH14
Pavir.6KG263400.v	GO:000074	03410(Base	swi/snf-rela	0.202923	0.620914	43.349964	1.347528	0.0184314	-5.761691	8.07498E-05	0.007415139	down	yes	High mobil	HMGB7
Pavir.7NG264700.v	GO:000367	NA	plant protei	0.134434	0.0001	2.162255	5.071689	0.0185976	-5.74874	0.000148317	0.011467723	down	yes	NA	NA
Pavir.3NG176700.v	GO:000551	NA	iq calmodul	0.104489	0.0001	2.9946741	2.5596463	0.0188302	-5.730807	0.000143921	0.011242309	down	yes	NA	NA
Pavir.3NG197700.v	GO:00055(	NA	nitric oxide	1.442624	0.217382	31.805094	56.22084	0.0188581	-5.728668	6.48533E-07	0.00012063	down	yes	Fruit protei	pKIWI502
Pavir.5KG301000.v	GO:000014	00500(Star	lyst-interact	0.272317	0.071989	12.079456	5.915626	0.0191333	-5.707768	2.70543E-08	8.91677E-06	down	yes	Callose syn	CALS7
Pavir.9KG271400.v	NA	04141(Prot	small heat-s	3.994314	0.544356	109.36082	124.57139	0.0194016	-5.687677	1.07592E-06	0.000190686	down	yes	Small heat s	HSP22
Pavir.7NG104400.v	NA	04626(Plan	leucine-rich	0.283105	0.0001	6.238095	8.316889	0.0194576	-5.683523	0.000248311	0.016720058	down	yes	NA	NA
Pavir.9KG336600.v	GO:000557	03010(Ribo	hydrophobic	0.346052	0.057515	14.257888	6.415439	0.0195211	-5.678819	6.1751E-06	0.000895818	down	yes	Lipid transf	EARLI1
Pavir.6KG182900.v	NA	NA	bhlh transcr	0.2130135	0.0001	9.5640872	1.2816384	0.0196495	-5.669361	0.000670253	0.034976986	down	yes	Transcriptic	IBL1;IBL1
Pavir.3KG224900.v	NA	NA	0	0.480926	0.0001	4.939131	19.51009	0.0196745	-5.66753	0.000350246	0.02171584	down	yes	NA	NA
Pavir.9KG495800.v	GO:00007¢	04070(Phos	histone h2a	3.740343	4.230018	389.0993	7.540222	0.0200947	-5.63704	0.000158901	0.012072757	down	yes	Histone H2	-
Pavir.8KG302900.v	GO:000367	NA	duf761-asso	0.48398	0.0001	18.846561	4.769561	0.0204979	-5.608383	0.000684586	0.035675381	down	yes	NA	NA
Pavir.2NG544900.v	GO:000485	NA	leucine-rich	10.096333	0.154141	384.25751	113.82041	0.0205801	-5.602609	0.000376005	0.022823311	down	yes	Polygalactu	FOR1
Pavir.7NG444200.v	GO:000455	00500(Star	glucosidase	0.068221	0.0001	2.186188	1.097457	0.0208065	-5.586825	0.000266002	0.01772071	down	yes	Glucan end	OLE9
Pavir.9KG077300.v	GO:000551	NA	iq calmodul	0.051293	0.02452	1.825245	1.748015	0.0212168	-5.558652	1.32279E-05	0.001719769	down	yes	Protein IQ-l	IQD31
Pavir.1NG130300.v	GO:000578	NA	protein of u	0.4207335	0.0001	7.7370202	11.984527	0.0213388	-5.550379	0.00027869	0.018274388	down	yes	NA	NA
Pavir.1NG266100.v	GO:000367	04144(End	protein of u	0.476614	0.0001	19.356773	2.885528	0.0214328	-5.544038	0.001279028	0.054501785	down	yes	NA	NA
Pavir.6KG144200.v	GO:000436	00480(Glut	glutathione	0.273249	0.092042	12.421873	4.509069	0.0215754	-5.534472	9.5998E-08	2.59492E-05	down	yes	Glutathione	GSTF13
Pavir.2NG259200.v	GO:000573	NA	amine oxida	0.141117	0.043575	5.780437	2.760272	0.0216249	-5.531163	1.3647E-06	0.000232017	down	yes	NA	NA
Pavir.3KG035500.v	GO:000487	NA	late embryo	0.178868	0.0001	2.879016	5.396737	0.0216256	-5.531117	0.000287341	0.018776134	down	yes	NA	NA
Pavir.3KG445300.v	GO:00007¢	04070(Phos	histone h2a	1.006993	1.199846	99.920822	1.65994	0.021725	-5.524502	0.000176398	0.013004172	down	yes	Probable hi	OsI_012923
Pavir.6KG251700.v	GO:000521	NA	xanthine-ura	0.107217	0.0001	3.8069186	1.0665331	0.0220207	-5.504993	0.000525555	0.029277606	down	yes	Nucleobase	NAT6;NAT6
Pavir.2KG498400.v	GO:000485	NA	leucine-rich	7.730229	0.308084	285.2833	78.739792	0.0220819	-5.500993	0.00012582	0.010101337	down	yes	Polygalactu	FOR1
Pavir.3NG318400.v	NA	NA	thaumatin fa	0.692182	0.421836	42.749344	7.595634	0.0221277	-5.498003	4.56855E-07	9.17936E-05	down	yes	NA	NA
Pavir.7NG322300.v	GO:000557	NA	notum-relat	1.063833	0.024305	32.840202	16.028395	0.0222666	-5.488974	7.09652E-05	0.006699435	down	yes	Pectin acety	PAE7
Pavir.6NG045000.v	NA	NA	0	0.111818	0.0001	3.16052	1.856347	0.0223083	-5.486273	0.000254254	0.017028678	down	yes	NA	NA
Pavir.6KG178600.v	GO:000484	NA	calcium-acti	0.13041	0.0001	1.122104	4.596188	0.0228232	-5.453352	0.000669052	0.034976986	down	yes	NA	NA
Pavir.6KG271100.v	GO:00009(	00190(Oxid	myb-like dn	0.388132	0.064059	11.189509	8.457385	0.0230159	-5.441225	1.27178E-06	0.000219196	down	yes	Transcriptic	MYB106
Pavir.4KG396200.v	GO:00001€	03040(Splic	predicted rn	0.055845	0.20738	7.973574	3.368454	0.0232079	-5.429238	6.26168E-06	0.000904885	down	yes	Probable RI	ARP1
Pavir.9NG670800.v	GO:000573	NA	rho/rac/cdc	0.0954	0.031515	4.099209	1.346132	0.0233071	-5.423088	6.69864E-06	0.000956989	down	yes	Rho GTPas	REN1
Pavir.3KG383800.v	NA	NA	0	0.0747581	0.0821264	4.784684	1.8758193	0.0235545	-5.407856	4.15086E-07	8.61659E-05	down	yes	NA	NA
Pavir.6NG102600.v	GO:000455	00500(Star	glucan endo	0.067705	0.0001	1.850985	1.023632	0.0235875	-5.405834	0.000371019	0.02259369	down	yes	Glucan end	At5g58090
Pavir.8NG109100.v	GO:000367	04144(End	protein of u	0.200773	0.070948	3.320867	8.195704	0.0235939	-5.405441	7.25753E-07	0.000133018	down	yes	NA	NA
Pavir.2KG139500.v	GO:000551	NA	ricin-type b	0.206231	0.022671	7.181326	2.477132	0.0236996	-5.398991	6.94371E-05	0.006621724	down	yes	Agglutinin	-
Pavir.4KG280300.v	GO:00055(	00053(Asco	l-ascorbate	0.21897	0.115833	12.34629	1.607464	0.0239938	-5.381197	5.02954E-06	0.000749901	down	yes	L-ascorbate	-
Pavir.9KG642000.v	GO:000573	00330(Argi	polyamine c	0.8678966	0.200036	11.036612	33.366447	0.0240509	-5.377767	6.40102E-07	0.000119655	down	yes	Polyamine c	PAO6;PAO6
Pavir.8KG062400.v	GO:000404	00970(Ami	amidase//su	0.2631289	0.02114	10.108535	1.6938282	0.0240858	-5.375676	9.49077E-05	0.008312274	down	yes	Fatty acid a	FAAH;FAAH;FAAH
Pavir.9NG420300.v	GO:000367	04141(Prot	small heat-s	4.164549	0.570563	91.958443	100.97588	0.0245426	-5.348567	8.70198E-07	0.000157192	down	yes	23.5 kDa h	HSP23.5
Pavir.5NG210200.v	GO:000467	04626(Plan	protein kina	0.070713	0.1637571	4.4679609	5.0800696	0.0245569	-5.347727	4.37814E-06	0.000668698	down	yes	Serine/threc	PBL27;PBL27
Pavir.6NG043200.v	GO:00055(	00350(Tyro	catechol oxi	0.205678	0.0001	6.962487	1.37945	0.0246679	-5.341222	0.00102752	0.047024362	down	yes	Polyphenol	PPO
Pavir.8KG300800.v	NA	NA	0	0.263681	0.0001	8.591384	1.968702	0.0249791	-5.323137	0.000961235	0.045258758	down	yes	NA	NA
Pavir.7KG282700.v	NA	NA	0	0.173964	0.0001	3.282164	3.568805	0.0254072	-5.298618	0.000396575	0.023764777	down	yes	14 kDa prol	-
Pavir.1NG080900.v	GO:000367	NA	platz transc	0.147969	0.0001	3.4803361	2.33727	0.0254519	-5.296084	0.000495835	0.02831307	down	yes	NA	NA
Pavir.5NG164200.v	GO:000367	NA	protein of u	0.128673	0.0001	2.0298	2.982634	0.0256907	-5.282609	0.000602606	0.032345315	down	yes	NA	NA
Pavir.6KG133300.v	GO:000078	NA	histone h4 (	0.345315	1.784391	81.377243	1.455171	0.025711	-5.281469	0.000585861	0.031718374	down	yes	Histone H4	-
Pavir.3KG106700.v	GO:00007¢	04070(Phos	histone h2a	5.956444	0.855502	241.16296	23.076567	0.0257794	-5.277636	0.000101708	0.008665437	down	yes	Probable hi	OsI_017523
Pavir.6KG044100.v	NA	04626(Plan	ph domain l	0.103674	0.0001	2.213155	1.769301	0.0260578	-5.262141	0.000507159	0.028741313	down	yes	NA	NA
Pavir.5NG306900.v	NA	NA	0	0.2219216	0.0001	2.7984622	5.6394685	0.0263123	-5.248118	0.000694376	0.03598591	down	yes	Hydroxypro	HPAT1;HPAT1
Pavir.4KG403000.v	GO:000382	00941(Flav	nad depend	0.106992	0.0001	2.040099	1.978687	0.0266478	-5.229837	0.000493054	0.028197146	down	yes	Putative ant	-;-
Pavir.9KG321900.v	GO:000487	NA	gb	0.080463	0.046374	3.570765	1.185107	0.0266696	-5.228662	0.000106819	0.008976715	down	yes	NA	NA
Pavir.7KG414700.v	GO:000455	04075(Plan	xyloglucan	2.688932	0.185732	95.682907	11.912556	0.0267173	-5.22608	0.000146264	0.011377995	down	yes	Xyloglucan	XTH9
Pavir.5KG708500.v	GO:000367	NA	plant protei	0.260496	0.0001	4.908494	4.770698	0.0269233	-5.215	0.000636117	0.033719184	down	yes	NA	NA
Pavir.9KG520200.v	GO:000455	04075(Plan	xyloglucan	0.424393	0.108766	14.431889	5.329918	0.0269793	-5.212005	4.16545E-06	0.000641429	down	yes	Probable xy	XTH28
Pavir.8KG361400.v	GO:00071€	NA	domain of u	0.130271	0.0001	1.85214	2.961111	0.0270859	-5.206317	0.000621628	0.033129711	down	yes	NA	NA
Pavir.2NG415600.v	GO:000367	03013(RNA	genomic dn	0.12271	0.0001	3.111979	1.343847	0.0275617	-5.181193	0.000967877	0.045432108	down	yes	NAC doma	NAC086
Pavir.9NG437600.v	GO:000367	NA	solute carri	0.0001	0.440563	6.948562	8.518128	0.0284911	-5.133345	0.000899526	0.043109551	down	yes	NA	NA
Pavir.9NG420200.v	NA	NA	0	6.694215	0.816966	155.77327	106.70103	0.0286168	-5.126993	3.63667E-06	0.000569336	down	yes	NA	NA
Pavir.5KG038600.v	NA	NA	0	0.178089	0.066904	4.552462	3.967225	0.0287561	-5.119988	8.55445E-05	0.007728982	down	yes	NA	NA
Pavir.4NG149200.v	GO:000367	NA	3'-5' exorib	0.0236291	0.1182859	2.7062191	2.224514	0.0287817	-5.118704	9.64549E-05	0.008350459	down	yes	Uncharacter	At3g15140;At3g15140;At3g15140;At3g15140
Pavir.2NG180100.v	GO:000573	00910(Nitr	cytochrome	0.1563884	0.0001	4.0342184	1.4005359	0.028794	-5.118087	0.001188655	0.051811312	down	yes	Cytochrome	CB5LP;CB5LP;CB5LP;CB5LP;CB5LP
Pavir.9KG604500.v	GO:000588	NA	bifunctional	0.842582	0.0001	27.936411	1.258322	0.0288642	-5.114576	0.005831466	0.137393622	down	yes	Protein YL	YLS3
Pavir.5NG246000.v	GO:000557	NA	domain of u	0.2216074	0.0001	4.2052764	3.4479454	0.0289692	-5.109339	0.000830513	0.040643524	down	yes	NA	NA

Pavir.6NG327000.v	GO:000367	04144(Endo	protein of u	0.114371	0.0001	1.228544	2.715933	0.0290206	-5.10678	0.000965184	0.04538781	down	yes	NA	NA
Pavir.9NG354600.v	NA	NA	0	0.520032	0.187524	23.184278	1.124969	0.0291065	-5.102517	0.000166062	0.012469856	down	yes	Kinesin-like	KIN5A
Pavir.5KG658100.v	GO:000078	00053(Asco	histone h3 (	1.463048	4.501643	193.32347	7.944858	0.0296355	-5.076529	0.000170564	0.012706237	down	yes	Histone H3	HTR2
Pavir.8KG212300.v	NA	NA	small heat-s	12.537425	0.504311	200.24332	238.30568	0.0297384	-5.07153	0.000155873	0.011903654	down	yes	NA	NA
Pavir.5KG385400.v	GO:000419	NA	aspartyl pro	0.440432	0.0001	11.563101	3.042771	0.0301613	-5.051158	0.001895881	0.070349639	down	yes	Aspartyl pr	APF2
Pavir.3NG195100.v	NA	NA	protein of u	0.274497	0.0001	4.076218	5.007171	0.0302307	-5.047843	0.000932272	0.044339587	down	yes	NA	NA
Pavir.1NG462000.v	GO:000509	04120(Ubic	immunoglol	0.84413	0.0001	18.653494	9.253289	0.0302518	-5.046836	0.0013632	0.056978853	down	yes	Rho GDP-d	GDI1
Pavir.9NG682000.v	NA	NA	0	0.304245	0.0001	6.907706	3.059003	0.0305362	-5.033338	0.001108421	0.049402971	down	yes	NA	NA
Pavir.9KG639700.v	GO:000551	04075(Plan	f-box protei	0.696911	0.0001	15.966658	6.844543	0.0305556	-5.032417	0.00132609	0.055795265	down	yes	F-box prote	SNE
Pavir.5KG160800.v	NA	NA	0	0.330553	3.354987	106.98822	13.130316	0.0306825	-5.026439	0.000138641	0.010897847	down	yes	NA	NA
Pavir.6NG326100.v	GO:000367	04144(Endo	protein of u	0.184703	0.0001	1.9042244	4.0877673	0.0308417	-5.018976	0.001296442	0.055120254	down	yes	NA	NA
Pavir.1KG075900.v	NA	NA	0	0.221267	0.0001	4.690302	2.463973	0.0309419	-5.014293	0.001213679	0.052657697	down	yes	NA	NA
Pavir.7NG165100.v	GO:000551	NA	chromosom	0.241312	0.0001	6.561845	1.230985	0.0309787	-5.012578	0.002484796	0.082547508	down	yes	NA	NA
Pavir.2KG311200.v	GO:000551	04075(Plan	cyclin-d1-1	1.036146	0.199466	31.446867	8.354419	0.0310445	-5.009517	6.42528E-06	0.000924969	down	yes	Cyclin-D2-	CYCD2-1
Pavir.3KG129500.v	NA	NA	0	0.488872	0.0001	11.926579	3.76852	0.0311544	-5.004418	0.001629041	0.063625719	down	yes	NA	NA
Pavir.6KG382600.v	GO:000864	NA	rag1-activat	0.401382	0.0001	9.782767	3.009552	0.0313846	-4.993799	0.001867632	0.069615606	down	yes	Bidirection	SWEET11
Pavir.6KG088800.v	NA	NA	0	0.09945	0.0001	1.17454	1.97525	0.0316053	-4.983691	0.00124696	0.053563962	down	yes	NA	NA
Pavir.3KG246500.v	NA	NA	0	0.6141	0.039725	12.760637	7.780271	0.0318304	-4.973452	8.86909E-05	0.007953182	down	yes	NA	NA
Pavir.1NG532800.v	GO:000097	00130(Ubic	homeobox-l	0.2772203	0.0716662	7.1105295	3.8326822	0.0318815	-4.971135	2.06581E-06	0.000340433	down	yes	Homeobox-	ROC8;ROC8
Pavir.1KG053600.v	GO:000367	NA	arabidopsis	0.171467	0.0001	2.68046	2.681146	0.0319992	-4.965821	0.001142726	0.050334863	down	yes	NA	NA
Pavir.5NG630500.v	GO:000551	NA	calmodulin-	0.138432	0.0001	3.196711	1.108217	0.0321799	-4.957698	0.001549559	0.061824615	down	yes	Calmodulin	PICBP
Pavir.9NG113400.v	GO:000716	NA	ankyrin repe	0.094018	0.0001	1.166886	1.757759	0.032181	-4.957647	0.00133071	0.055927022	down	yes	Protein AC	ACD6
Pavir.1NG527600.v	GO:000155	04075(Plan	lrr receptor-	1.1672397	0.3044131	38.993638	6.7079204	0.0322014	-4.956734	2.50048E-05	0.002935949	down	yes	LRR recept	ER1;ER1;ER1;ER1;ER1;ER1
Pavir.2NG539200.v	NA	NA	plant protei	0.178676	0.0001	1.812057	3.729272	0.0322623	-4.954007	0.001414394	0.058270877	down	yes	NA	NA
Pavir.5KG322800.v	GO:000367	00561(Glyc	no apical m	0.049687	0.057172	2.173997	1.06242	0.0330177	-4.920617	8.78626E-05	0.007897755	down	yes	NAC doma	NAC035
Pavir.2KG391200.v	NA	NA	0	0.338069	0.050265	6.3257482	5.4156725	0.0330739	-4.918165	2.90135E-06	0.000465865	down	yes	NA	NA
Pavir.7NG123300.v	NA	NA	0	0.411248	0.0001	7.224612	5.209666	0.0330818	-4.91782	0.001467683	0.059680995	down	yes	NA	NA
Pavir.9KG633700.v	GO:000551	04075(Plan	cyclin-d5-1	0.43857	0.0001	10.296729	2.912477	0.0332094	-4.912264	0.002454454	0.081953792	down	yes	Cyclin-D5-	CYCD5-3
Pavir.7KG285200.v	GO:000819	00908(Zeat	cis-zeatin o	0.052492	0.105565	2.645338	2.097875	0.0333228	-4.907348	5.40986E-05	0.005464105	down	yes	Putative cis	SB20O07.14
Pavir.3NG176100.v	NA	NA	myosin-13-r	0.118147	0.118533	5.952858	1.107343	0.0335231	-4.8987	0.000252444	0.016967948	down	yes	NA	NA
Pavir.5NG248200.v	GO:000460	00940(Phen	peroxidase (	0.394435	0.0001	10.092006	1.61561	0.033699	-4.89115	0.003786759	0.108898049	down	yes	Peroxidase	PER72
Pavir.2NG520400.v	GO:000487	NA	late embryo	0.482917	0.175126	6.981978	12.27637	0.0341692	-4.871158	3.41234E-05	0.003727083	down	yes	NDR1/HIN	NHL2
Pavir.2NG214000.v	GO:000815	NA	bax inhibito	0.15312	0.0001	1.988293	2.467713	0.0343851	-4.862075	0.001339396	0.056229203	down	yes	Protein LIF	LFG4
Pavir.2NG172700.v	GO:000003	00062(Fatty	3-ketoacyl-c	0.088448	0.0001	1.492835	1.072896	0.0345118	-4.856766	0.001383675	0.057382809	down	yes	3-ketoacyl-	KCS17
Pavir.5KG211900.v	GO:000487	NA	late embryo	1.587069	0.058082	18.481628	29.14238	0.0345446	-4.855397	0.000176558	0.013004172	down	yes	NDR1/HIN	NHL2
Pavir.6KG202800.v	GO:000467	04626(Plan	protein kina	0.114474	0.079172	2.032825	3.53967	0.0347503	-4.84683	2.41842E-05	0.002866477	down	yes	Receptor-lil	ALE2
Pavir.8KG170400.v	NA	NA	0	0.1833942	0.0001	2.009948	3.2548861	0.0348528	-4.842582	0.001793875	0.067752155	down	yes	NA	NA
Pavir.9KG329100.v	GO:000557	NA	pollen allerg	0.205305	0.0001	1.958619	3.843656	0.0354008	-4.820075	0.001811846	0.068177911	down	yes	Expansin-B	EXPB2
Pavir.1NG133800.v	GO:000367	04141(Prot	x-box transc	2.000448	0.0001	33.842499	22.596737	0.0354461	-4.818231	0.003434837	0.102426308	down	yes	Basic leucir	BZIP19
Pavir.9KG529800.v	GO:004703	00040(Pent	aldo/keto re	0.1120957	0.0001	1.81883	1.3341603	0.0355839	-4.812632	0.001665416	0.064442719	down	yes	Deoxymugi	DMAS1;DMAS1;DMAS1
Pavir.2KG578500.v	GO:000419	NA	aspartyl pro	3.087008	0.734726	81.787247	25.489841	0.0356249	-4.810971	1.60147E-05	0.002025989	down	yes	Aspartyl pr	AP25
Pavir.7KG219800.v	GO:000419	NA	aspartyl pro	0.157271	0.0001	2.628876	1.742521	0.0360002	-4.795853	0.001715875	0.065853481	down	yes	Aspartyl pr	At5g10770
Pavir.6NG371500.v	GO:000552	NA	leucine-rich	0.127158	0.072637	3.251883	2.236728	0.0364017	-4.779849	0.000206092	0.014528109	down	yes	Putative dis	At3g14460
Pavir.1NG392900.v	NA	NA	late embryo	0.767134	0.029483	7.045969	14.800336	0.0364646	-4.777359	0.000446758	0.02586446	down	yes	NA	NA
Pavir.1KG470100.v	GO:000367	04712(Circ	dof domain,	0.076688	0.282958	8.413622	1.436086	0.0365134	-4.775431	0.000278338	0.018274388	down	yes	Dof zinc fin	DOF5.6
Pavir.9NG483000.v	GO:000097	NA	gata transcr	2.698754	0.0001	53.996132	19.490519	0.0367258	-4.767063	0.007064527	0.154502603	down	yes	GATA trans	GATA4
Pavir.6NG300200.v	GO:000373	NA	mitochondri	0.54102	0.213968	15.307825	5.038835	0.0371062	-4.752194	5.7532E-06	0.000837849	down	yes	Probable m	At3g20240
Pavir.8KG127400.v	NA	NA	0	0.060962	0.105405	2.188617	2.285456	0.0371847	-4.749148	0.000246683	0.016678944	down	yes	NA	NA
Pavir.9KG004200.v	NA	NA	0	0.446729	0.0001	8.579445	3.424977	0.037222	-4.747699	0.00236214	0.079712811	down	yes	Protein GL	GIR1
Pavir.8KG288600.v	GO:000815	04144(Endo	protein of u	0.218345	0.325547	3.528782	10.979396	0.0374886	-4.737402	2.84115E-06	0.000458156	down	yes	NA	NA
Pavir.2NG495200.v	GO:000508	NA	prone (plant	0.256968	0.0001	2.04931	4.805424	0.0375023	-4.736879	0.002806662	0.089258594	down	yes	Rop guanin	ROPGEF3
Pavir.2KG013500.v	NA	NA	leucine-rich	0.407606	0.289171	10.256438	8.278708	0.0375922	-4.733423	5.13022E-07	9.93597E-05	down	yes	NA	NA
Pavir.3NG154800.v	GO:000521	NA	patellin-6	0.614934	0.327009	19.999216	4.993986	0.037688	-4.729752	5.29918E-06	0.000780808	down	yes	Patellin-6 C	PATL6
Pavir.7NG066500.v	GO:000550	00350(Tyro	catechol oxi	3.143764	0.092937	77.434402	8.208397	0.037793	-4.725736	0.001364216	0.056978853	down	yes	Polyphenol	-
Pavir.8KG360900.v	GO:000685	NA	leucine-rich	0.086124	0.0001	0.99966	1.276924	0.0378743	-4.722637	0.002141437	0.07505616	down	yes	NA	NA
Pavir.3NG019100.v	GO:000452	03030(DNA	ribonucleas	0.257569	0.212602	10.620882	1.73335	0.0380575	-4.715676	0.000106393	0.008976715	down	yes	Ribonuclea	At2g25100
Pavir.9KG152400.v	GO:000550	00053(Asco	multi-coppe	0.226501	0.0001	3.651281	2.300495	0.0380728	-4.715094	0.002043249	0.073184918	down	yes	Monocoppe	SKS2
Pavir.6KG141000.v	NA	NA	0	1.775527	0.0001	37.475719	9.11745	0.0381092	-4.713718	0.004689922	0.123006906	down	yes	NA	NA
Pavir.4KG010800.v	GO:000550	00053(Asco	multi-coppe	0.8512035	0.1800875	19.792738	7.2284838	0.038166	-4.711569	9.06533E-06	0.001270198	down	yes	Monocoppe	SKU5;SKU5
Pavir.2NG169300.v	GO:000460	00564(Glyc	phosphatida	0.03775	0.039811	1.019885	0.999644	0.0384055	-4.702544	0.000417126	0.0245738	down	yes	Phosphatid	CDS1
Pavir.8NG237900.v	GO:000367	00563(Glyc	extensin, pr	0.0001	0.64145	12.881681	3.780128	0.0385042	-4.69884	0.003908683	0.110754875	down	yes	Gibberellin	GASA3
Pavir.6NG319300.v	GO:000382	00500(Star	udp-glucurc	0.304027	0.043568	5.825817	3.174774	0.0386191	-4.694541	7.5775E-05	0.007082321	down	yes	UDP-glucu	GAE6
Pavir.7NG272100.v	NA	NA	cotton fibre	0.129636	0.0001	1.439515	1.912161	0.0387078	-4.691232	0.002310622	0.07849639	down	yes	NA	NA
Pavir.1KG213700.v	GO:004746	00909(Sesq	alpha-humu	1.9721478	0.0721085	24.906878	27.807866	0.0387796	-4.688559	0.000206887	0.014556885	down	yes	Alpha-terpi	TPS26;TPS26
Pavir.5NG041600.v	GO:000367	04141(Prot	x-box transc	0.9494634	0.0001	16.741857	7.7263191	0.0388081	-4.687498	0.003949141	0.111480907	down	yes	Basic leucir	BZIP02;BZIP02;BZIP02;BZIP19
Pavir.1KG451400.v	GO:000078	NA	histone h4 (	1.312498	5.004972	157.97742	4.197553	0.0389547	-4.68206	0.000926323	0.04416845	down	yes	Histone H4	-
Pavir.7KG255400.v	GO:000418	00460(Cyar	serine carbo	0.130497	0.187439	6.890141	1.265729	0.0389825	-4.68103	0.000129358	0.010275581	down	yes	Serine carb	SCPL35
Pavir.7NG328300.v	GO:000456	00520(Ami	chitinase//su	0.099173	0.0001	1.413236	1.123478	0.0391345	-4.675416	0.002302727	0.078298966	down	yes	Nod factor l	NFH1
Pavir.1NG374700.v	GO:000078	NA	histone h3 (	0.730805	1.802134	63.04953	1.514394	0.0392315	-4.671844	0.001168407	0.051225832	down	yes	Histone H4	-
Pavir.5KG626900.v	NA	NA	bowman-bi	1.699353	0.530561	43.476719	12.822084	0.0396086	-4.658044	7.3849E-06	0.001047067	down	yes	NA	NA
Pavir.3NG218500.v	NA	NA	probable lip	3.332075	0.120769	50.471813	36.683495	0.0396171	-4.657731	0.000223602	0.015387183	down	yes	Non-specif	LTP110-A
Pavir.5NG406400.v	GO:000897	00564(Glyc	phospholipa	0.158247	0.0001	2.265177	1.720125	0.0397327	-4.653528	0.002491187	0.08257308	down	yes	Phospholip	OsI_03088
Pavir.7NG155600.v	NA	NA	0	0.234165	0.023675	3.570272	2.836967	0.040242	-4.635155	0.000101113	0.008665437	down	yes	NA	NA
Pavir.4NG074300.v	GO:000016	03040(Splic	predicted rn	0.138727	0.046846	3.051516	1.50788	0.0407012	-4.618784	0.000112628	0.009334008	down	yes	Probable R	ARP1
Pavir.7KG113900.v	GO:000367	00230(Puri	dna primase	0.366629	0.253927	13.857224	1.24481	0.0410909	-4.605038	0.000179301	0.013117311	down	yes	Probable D	At1g67320
Pavir.1NG029300.v	GO:000009	NA	extensin-lik	1.833274	0.0001	31.36342	13.252557	0.0410923	-4.604987	0.006772176	0.150030056	down	yes	Leucine-ric	LRX1
Pavir.1NG479800.v	GO:000557	04626(Plan	lysm domai	0.536423	0.0001	10.28026	2.738646	0.0412111	-4.600824	0.004982247	0.127225339	down	yes	LysM doma	LYP6
Pavir.5KG724000.v	NA	NA	0	0.5513887	0.0001	11.37282	2.0045685	0.0412254	-4.600321	0.006284559	0.144060155	down	yes	NA	NA
Pavir.1NG506100.v	GO:000034	03030(DNA	dna replicat	0.448876	0.391221	19.209162	1.148666	0.0412665	-4.598884	0.000189652	0.013590638	down	yes	DNA replic	MCM5
Pavir.3KG245400.v	GO:000521	NA	patellin-6	0.34675	0.717311	21.776495	3.86617	0.0414957	-4.590893	2.68847E-05	0.003089113	down	yes	Patellin-6 C	PATL6
Pavir.6NG111800.v	NA	NA	0	0.5575614	0.0708103	7.6046641	7.5379998	0.0414968	-4.590857	2.57751E-05	0.002989041	down	yes	NA	NA
Pavir.1KG175400.v	GO:000367	04141(Prot	x-box transc	1.842064	0.0001	22.979214	21.297716	0.0416055	-4.587082	0.005593935	0.134904319	down	yes	Basic leucir	BZIP19

Pavir.4KG252200.v4	GO:000455	00500(Starc	glycosyl hyc	0.13131	0.0001	1.172208	1.966503	0.0418675	-4.578025	0.003372462	0.101208889	down	yes	Glucan endo	At1g32860
Pavir.8NG106200.v4	GO:000578	NA	protein of u	0.485614	0.0001	4.707384	6.893785	0.0418677	-4.578019	0.003430546	0.102379593	down	yes	NA	NA
Pavir.4NG118300.v4	GO:000003	00062(Fatty	3-ketoacyl-c	0.638153	0.024744	7.278253	8.351147	0.0424135	-4.559334	0.000400418	0.023837006	down	yes	3-ketoacyl-C	CUT1
Pavir.2NG489400.v4	GO:000981	00908(Zeat	glucosyl/glu	0.076507	0.058271	1.966764	1.203628	0.0425115	-4.556004	0.000198036	0.014082726	down	yes	Myricetin 3	UGT709G2
Pavir.9KG562000.v4	GO:000367	00630(Glyc	f12p19.8 pr	0.248234	0.0001	1.694883	4.139575	0.0425633	-4.554245	0.004228357	0.116389794	down	yes	NAC domai	NAC086
Pavir.9NG561600.v4	GO:000535	NA	sugar transp	0.154111	0.2093927	6.6953223	1.7598501	0.0429919	-4.539792	7.34526E-05	0.006899584	down	yes	Sugar transp	At5g18840;At5g18840
Pavir.1NG442300.v4	GO:000563	NA	wrc (wrc) //	0.404849	0.059527	8.269322	2.528919	0.0430048	-4.539359	0.000267017	0.017756891	down	yes	Growth-reg	GRF4
Pavir.2KG090300.v4	NA	04626(Plan	leucine-rich	0.459853	0.0001	6.341828	4.318899	0.0431446	-4.534676	0.004684281	0.123006906	down	yes	NA	NA
Pavir.1KG219600.v4	GO:000367	NA	protein of u	0.123844	0.0001	1.44402	1.422246	0.0432423	-4.531412	0.002966884	0.092741035	down	yes	Protein DM	DMP6
Pavir.2NG532900.v4	GO:000970	NA	transporter l	0.246543	0.0001	3.059127	2.622044	0.0434141	-4.525692	0.003344763	0.10061873	down	yes	Organic cat	44106
Pavir.9KG245700.v4	NA	NA	phosphatidy	0.481941	0.029404	7.9064821	3.7088433	0.0440233	-4.505589	0.000307132	0.019760059	down	yes	NA	NA
Pavir.1KG081400.v4	NA	NA	0	0.787594	0.0001	10.344644	7.493123	0.0441588	-4.501156	0.004153789	0.114926602	down	yes	Transcriptic	IBL1
Pavir.1KG095300.v4	GO:000367	NA	protein of u	0.131077	0.0001	1.02275	1.941447	0.0442538	-4.498055	0.00341751	0.102152823	down	yes	NA	NA
Pavir.4KG277800.v4	GO:000550	00053(Asco	ascorbate ox	0.735797	0.074108	15.466456	2.679762	0.0446322	-4.485772	0.000515656	0.029047602	down	yes	L-ascorbate	-
Pavir.6KG253700.v4	NA	NA	0	0.1720207	0.0213013	2.1485372	2.1553944	0.0449175	-4.476577	0.000418571	0.0245738	down	yes	NA	NA
Pavir.6KG298200.v4	GO:000561	NA	cupin doma	95.211411	1.04286	1015.4248	1125.5403	0.0449584	-4.475267	0.003196948	0.097737123	down	yes	Germin-like	GER5
Pavir.1NG510800.v4	GO:000094	NA	spindle and	0.25638	0.0001	4.401973	1.251871	0.0453638	-4.462314	0.005592791	0.134904319	down	yes	NA	NA
Pavir.5NG058000.v4	NA	NA	0	0.0832644	0.2014193	3.4459158	2.7925319	0.0456337	-4.453755	9.57452E-05	0.008335023	down	yes	NA	NA
Pavir.9NG420100.v4	GO:000940	04141(Prot	small heat-s	2.2530646	0.3488622	38.021717	18.847061	0.0457532	-4.449985	2.64912E-05	0.003053229	down	yes	Small heat s	HSP21;HSP21
Pavir.2KG380400.v4	GO:000079	04070(Phos	histone h2a	1.36681	2.317333	78.479126	1.978623	0.0457898	-4.44883	0.001607026	0.063114666	down	yes	Probable hi	OsI_002060
Pavir.1NG559000.v4	GO:000557	00561(Glyc	zinc finger f	24.662941	0.298476	207.08682	336.41705	0.0459268	-4.444519	0.003007786	0.093552614	down	yes	GDSL ester	At5g33370
Pavir.3KG410300.v4	GO:000561	04144(Endo	germin-like	1.491433	0.0001	16.040873	16.424189	0.0459427	-4.44402	0.004766774	0.123774715	down	yes	Germin-like	Os05g0277500
Pavir.5KG603200.v4	GO:000467	04075(Plan	leucine rich	1.085516	0.150427	17.713894	9.148665	0.0460099	-4.441913	3.28565E-05	0.003609705	down	yes	Probable LI	IRK
Pavir.9KG049900.v4	GO:000367	NA	homeobox p	0.021849	0.099189	1.524958	1.1039613	0.046041	-4.440938	0.000498333	0.028412543	down	yes	BEL1-like l	BLH11;BLH11;BLH11
Pavir.1NG306900.v4	NA	NA	0	0.2416036	0.0001	1.7910559	3.4363914	0.0462374	-4.434796	0.005582002	0.134904319	down	yes	NA	NA
Pavir.7NG049000.v4	NA	NA	0	1.020998	0.523544	20.270771	13.07905	0.0463134	-4.432428	4.28263E-05	0.004507314	down	yes	NA	NA
Pavir.4NG321700.v4	GO:000551	03008(Ribo	cyclin-a2-1-	0.174151	0.174898	5.88384	1.646215	0.0463541	-4.431159	7.68582E-05	0.007148004	down	yes	Cyclin-A2-1	CYCA2-1
Pavir.5NG503800.v4	GO:000941	00904(Dite	gibberellin 2	0.617361	0.0001	7.715393	5.505002	0.0467052	-4.420273	0.005059238	0.128340138	down	yes	Gibberellin	GA2OX3
Pavir.1NG562500.v4	GO:000557	00062(Fatty	3-ketoacyl-c	0.412799	0.019006	2.909374	6.320307	0.0467844	-4.417829	0.001378728	0.057240815	down	yes	3-ketoacyl-C	KCS12
Pavir.2KG241500.v4	GO:000573	NA	cyclopropan	0.811617	0.0001	13.754059	3.579947	0.046828	-4.416484	0.007304305	0.156441785	down	yes	NA	NA
Pavir.3KG115000.v4	NA	NA	0	0.084108	0.084123	1.994291	1.576835	0.0471087	-4.407864	0.00017881	0.013117311	down	yes	NA	NA
Pavir.9KG142800.v4	GO:000550	00053(Asco	multi-coppe	2.617012	0.0001	39.99152	15.549355	0.0471205	-4.407502	0.009980961	0.181831401	down	yes	Monocoppe	SKU5
Pavir.9NG718800.v4	GO:001678	NA	alpha/beta-h	0.092224	0.122484	2.816404	1.724619	0.0472819	-4.402569	0.00021957	0.015165284	down	yes	NA	NA
Pavir.2KG265600.v4	GO:000367	NA	domain of u	0.113424	0.0559	2.246799	1.33292	0.0473009	-4.401988	0.000128125	0.010220858	down	yes	NA	NA
Pavir.8NG245500.v4	GO:000969	00970(Ami	nucleoporin	0.781721	0.0001	8.438878	7.992939	0.0475797	-4.39351	0.00471421	0.12336531	down	yes	Dirigent pro	DIR1
Pavir.1NG404400.v4	NA	NA	0	0.096044	1.19311	24.660187	2.427467	0.0475919	-4.393139	0.001311901	0.055571673	down	yes	NA	NA
Pavir.2KG521900.v4	NA	NA	extensin, pr	0.475905	0.0001	7.164515	2.804787	0.0477471	-4.388444	0.006437867	0.145716862	down	yes	NA	NA
Pavir.6KG017200.v4	GO:000367	03018(RNA	no apical m	0.197286	0.0001	1.64534	2.484117	0.0477995	-4.386861	0.004897772	0.126476958	down	yes	NAC domai	NAC035
Pavir.7NG155800.v4	NA	NA	adenylate cy	0.13285	0.020622	1.838892	1.367257	0.047868	-4.384794	0.000696079	0.036024497	down	yes	NA	NA
Pavir.8NG131400.v4	GO:000367	NA	plant protei	0.145343	0.0001	1.633439	1.362436	0.0485478	-4.364452	0.004691371	0.123006906	down	yes	UPF0481 p	At3g47200
Pavir.2KG385600.v4	NA	NA	0	0.26702	0.033983	4.944364	1.238287	0.0486851	-4.360376	0.001296844	0.055120254	down	yes	NA	NA
Pavir.4KG105200.v4	GO:000557	NA	f12f1.23 pro	0.848471	0.0001	13.29898	4.125484	0.0487	-4.359935	0.007611165	0.158610259	down	yes	NA	NA
Pavir.9KG649300.v4	GO:000419	NA	aspartyl pro	5.288866	0.609215	85.957115	34.858543	0.0488188	-4.356418	0.000198274	0.014082726	down	yes	Aspartyl pro	AP25
Pavir.1NG300000.v4	NA	NA	leucine-rich	0.189832	0.072583	1.55599	3.810872	0.0488954	-4.354157	0.000212303	0.014826876	down	yes	NA	NA
Pavir.9KG645400.v4	GO:000557	NA	outer arm dy	0.2443212	0.1552597	6.4419959	1.7276366	0.0489105	-4.353712	7.75525E-05	0.007194767	down	yes	NA	NA
Pavir.2KG453600.v4	GO:000455	04075(Plan	xyloglucan e	0.458565	0.0001	7.9716023	1.368827	0.0491053	-4.347976	0.010448626	0.185444601	down	yes	Probable xy	XTH8;XTH8
Pavir.8KG212200.v4	NA	NA	0	4.422975	0.940619	64.59385	44.127937	0.0493332	-4.341297	1.08364E-05	0.001454124	down	yes	NA	NA
Pavir.8NG084900.v4	NA	NA	0	0.467917	0.0001	3.291406	6.178524	0.0494214	-4.338721	0.006253592	0.144060155	down	yes	NA	NA
Pavir.6KG416100.v4	GO:000557	NA	domain of u	0.265232	0.0001	2.9836097	2.383605	0.0494357	-4.338303	0.005463599	0.133648303	down	yes	NA	NA
Pavir.1KG471500.v4	NA	NA	0	0.141158	0.0001	1.572504	1.281173	0.0495003	-4.336418	0.004631298	0.122629853	down	yes	NA	NA
Pavir.4KG351400.v4	NA	NA	0	0.138458	0.0001	1.611647	1.174934	0.0497233	-4.329934	0.004995962	0.127349589	down	yes	NA	NA
Pavir.2KG002600.v4	GO:000550	00053(Asco	multi-coppe	1.646422	0.203624	26.080956	11.124823	0.0497247	-4.329894	8.55736E-05	0.007728982	down	yes	L-ascorbate	-
Pavir.6NG285700.v4	GO:000469	04075(Plan	cycline//sub	0.0001	0.325441	5.0391013	1.483482	0.0499098	-4.324532	0.009209505	0.175061118	down	yes	Cyclin-D4-2	CYCD4-2;CYCD4-2;CYCD4-2
Pavir.8KG099900.v4	NA	NA	extensin, pr	4.207244	0.48444	65.702316	28.247131	0.0499384	-4.323707	0.000100345	0.008653603	down	yes	NA	NA
Pavir.9NG778500.v4	NA	NA	0	0.421598	0.0001	5.097412	3.317635	0.0501124	-4.318689	0.00650798	0.14647695	down	yes	NA	NA
Pavir.5KG353600.v4	GO:000588	01200(Carb	eama-like tr	0.0320339	0.1939998	2.5825946	1.8848457	0.0505958	-4.304839	0.000994469	0.046014367	down	yes	WAT1-rela	At5g64700;At5g64700;At5g64700;At5g64700
Pavir.4KG322200.v4	NA	NA	0	0.438256	0.320114	5.095907	9.803838	0.0508982	-4.296242	0.000137844	0.010857874	down	yes	NA	NA
Pavir.2NG453800.v4	GO:000368	NA	minichromo	0.236	0.0001	3.368413	1.238718	0.0512466	-4.286399	0.007870711	0.161510774	down	yes	NA	NA
Pavir.2KG385100.v4	GO:000367	NA	protein of u	0.8753654	0.2049418	12.807655	8.0525245	0.051788	-4.271238	4.53765E-05	0.004749112	down	yes	Protein AL7	APSR1;APSR1
Pavir.9NG638600.v4	GO:000455	00500(Starc	glucan endo	0.861483	0.024588	14.526954	2.519482	0.0519798	-4.265904	0.002946636	0.092338585	down	yes	Glucan endo	At2g01630
Pavir.2NG347900.v4	GO:000578	NA	receptor exp	0.8476705	0.0001	9.9584006	6.2561956	0.0522844	-4.257475	0.007431759	0.157460964	down	yes	Protein HV.	HVA22;HVA22
Pavir.5NG331100.v4	GO:000557	NA	expansin-a1	1.10274	0.103389	11.444789	11.590434	0.0523602	-4.255385	0.000180043	0.013135416	down	yes	Expansin-A	EXPA7
Pavir.5KG538900.v4	GO:001629	00561(Glyc	zinc finger f	1.097978	0.0001	9.364477	11.458531	0.0527339	-4.245126	0.007962268	0.162325709	down	yes	GDSL ester	At5g45670
Pavir.1KG238300.v4	GO:000508	NA	prone (plant	0.438183	0.040995	6.555266	2.505174	0.0528868	-4.240948	0.000639797	0.033810263	down	yes	Rop guanin	ROPGEF7
Pavir.9NG255800.v4	GO:000561	NA	microtubule	1.212841	0.080171	21.360342	3.058795	0.0529508	-4.239205	0.00187157	0.069693271	down	yes	Microtubule	EB1C
Pavir.4KG081300.v4	GO:000382	00500(Starc	nad depende	0.204777	0.0001	2.580454	1.251909	0.0534597	-4.225404	0.007593421	0.158328296	down	yes	UDP-glucu	GAE1
Pavir.7KG010800.v4	NA	04626(Plan	adenylate cy	0.436267	0.01398	4.290724	4.126017	0.0534942	-4.224473	0.002021338	0.072677271	down	yes	NA	NA
Pavir.1KG556900.v4	GO:000014	00500(Starc	lyst-interact	0.686024	0.566168	10.747511	12.620575	0.0535856	-4.222012	1.21929E-06	0.000211117	down	yes	Callose syn	CALS1
Pavir.9KG149900.v4	GO:000588	02010(ABC	atp-binding	0.331946	0.390768	8.4207652	5.0203563	0.0537689	-4.217085	3.92624E-06	0.000612118	down	yes	ABC transp	ABCG11;ABCG11
Pavir.3KG311800.v4	GO:000403	00010(Glyc	apospory-as	0.242721	0.103011	4.355641	2.068365	0.0538188	-4.215747	0.000234223	0.016000805	down	yes	Putative glu	-
Pavir.9NG028500.v4	GO:000521	NA	xanthine-ura	0.262352	0.157282	6.434831	1.341344	0.0539641	-4.211857	0.000325684	0.020600876	down	yes	Nucleobase	NAT6;NAT6
Pavir.2KG553400.v4	GO:000002	03010(Ribo	ribosomal p	0.666428	0.164454	12.770233	2.578239	0.0541345	-4.207308	0.000540549	0.029780115	down	yes	40S riboson	GSVIVT00034021001
Pavir.4KG370300.v4	GO:000521	NA	cellular reti	1.500038	0.027893	23.294001	4.776599	0.0544317	-4.199409	0.003638338	0.106425992	down	yes	Patellin-4 C	PATL4
Pavir.9NG092900.v4	NA	NA	0	0.625463	0.06441	6.336109	6.208452	0.0549938	-4.184587	0.000486789	0.02792387	down	yes	NA	NA
Pavir.7KG416700.v4	GO:005121	00270(Cyst	oxidoreduct	0.091564	0.105545	1.538714	2.011324	0.0555231	-4.170769	0.000525357	0.029277606	down	yes	Gibberellin	20ox2
Pavir.5NG502600.v4	GO:000367	04141(Prot	x-box transc	1.656812	0.0001	16.471466	13.292645	0.0556681	-4.167005	0.009910716	0.181735162	down	yes	Basic leucir	BZIP06
Pavir.6KG087500.v4	GO:000550	00350(Tyro	polyphenol	0.446786	0.507576	13.056113	3.979263	0.0560224	-4.157853	2.78576E-05	0.003191137	down	yes	Aureusidin	AS1
Pavir.5KG724200.v4	NA	NA	probable lip	3.047017	0.189025	47.731277	9.783182	0.0562648	-4.151622	0.00308754	0.095166652	down	yes	NA	NA
Pavir.9NG374500.v4	GO:000367	NA	pathogenesi	1.811596	0.0001	24.501675	7.537465	0.0565463	-4.144423	0.01212744	0.199698873	down	yes	Thaumatin-	TLP1
Pavir.2NG564300.v4	GO:000410	00511(Othe	alpha-l-fuco	0.245909	0.299562	7.974116	1.664447	0.0565926	-4.143244	0.000413626	0.024435795	down	yes	GDSL ester	At3g26430
Pavir.2KG519800.v4	GO:000588	NA	zinc finger c	0.2455151	0.1107532	4.5720019	1.7151188	0.0566664	-4.141364	9.01003E-05	0.008022128	down	yes	Probable pr	PAT22;PAT22;PAT22;PAT22;PAT22
Pavir.2NG347800.v4	GO:000557	04626(Plan	lysm domai	2.785794	0.0001	33.256756	15.755021	0.0568413	-4.136916	0.015979492	0.232267769	down	yes	LysM doma	LYP4

Pavir.9NG354500.v	GO:003024	00500(Starc	glucan endo	3.112209	0.0001	35.645214	18.911983	0.0570467	-4.131712	0.016310779	0.234716551	down	yes	Glucan endo	At1g32860
Pavir.7NG124900.v	NA	NA	adenylate cy	0.275796	0.006641	2.689382	2.223706	0.0574867	-4.120629	0.003805211	0.109139838	down	yes	NA	NA
Pavir.4KG288700.v	GO:000097	NA	transcription	0.140101	0.201591	1.31778	4.61584	0.0575858	-4.118144	0.000434321	0.025261227	down	yes	Protein CY	CGA1
Pavir.9NG199200.v	GO:000487	NA	late embryo	0.349109	0.0001	3.032315	3.031385	0.0575901	-4.118036	0.00742036	0.157460964	down	yes	NA	NA
Pavir.3KG400000.v	NA	NA	calcium-acti	2.3786111	0.0001	7.6052729	33.48382	0.0578915	-4.110504	0.018513005	0.248424689	down	yes	NA	NA
Pavir.7KG104900.v	NA	NA	0	0.400101	0.0001	4.697106	2.178426	0.0582066	-4.102675	0.008857845	0.172158188	down	yes	NA	NA
Pavir.1NG493700.v	GO:000367	NA	(phi-1) prot	0.836067	0.26245	12.832867	6.009225	0.0583012	-4.10033	8.34178E-05	0.007588997	down	yes	Protein PH	PHI-1
Pavir.5NG154500.v	GO:000024	00100(Ster	cholestenol	1.138332	0.280986	20.218285	4.087779	0.0583936	-4.098047	0.000527102	0.029277606	down	yes	Probable 3-	Os01g0103600
Pavir.9NG530300.v	GO:000367	NA	domain of u	0.2015004	0.0001	2.152114	1.2981282	0.0584308	-4.097127	0.009515858	0.178501907	down	yes	Protein LO	LNG1;LNG1;LNG2
Pavir.5NG559800.v	NA	NA	0	0.301959	0.0001	2.914265	2.247631	0.0585171	-4.094999	0.00662425	0.147553864	down	yes	NA	NA
Pavir.9KG132400.v	GO:000447	01200(Carb	malate dehy	0.1709452	0.9777392	2.540124	16.954054	0.0589245	-4.084989	0.00059151	0.031905404	down	yes	NAD-deper	-;-
Pavir.1NG331200.v	NA	NA	0	0.2431844	0.0001	2.9104632	1.2069132	0.0590872	-4.08101	0.011401979	0.193578392	down	yes	Uncharacter	At3g27390;At3g27390;At3g27390;At3g27390
Pavir.5NG062700.v	NA	NA	0	0.250779	0.0001	2.903736	1.336037	0.0591727	-4.078923	0.009982164	0.181831401	down	yes	Leucine-ricl	LRX2
Pavir.1KG176200.v	GO:000524	04626(Plan	cyclic nucle	1.008854	0.417595	7.199998	16.834743	0.0593495	-4.074621	1.90705E-05	0.002364802	down	yes	Potassium c	Os02g0245800;Os02g0245800
Pavir.5KG118800.v	NA	NA	0	0.498156	0.0001	3.577185	4.799258	0.059483	-4.071379	0.00919722	0.175059342	down	yes	NA	NA
Pavir.9KG123300.v	GO:00046	00940(Pher	peroxidase (	0.2299842	0.3977253	8.5491362	1.9850055	0.0595881	-4.068832	0.000381779	0.023062011	down	yes	Peroxidase	PER56;PER56
Pavir.2NG567700.v	GO:00046	00940(Pher	peroxidase /	0.916485	0.0001	4.288714	11.069287	0.0596813	-4.066578	0.011832457	0.197160934	down	yes	Peroxidase	PER70
Pavir.1KG249200.v	NA	NA	0	0.335904	0.0001	1.029832	4.588952	0.0598001	-4.063708	0.013556049	0.211344984	down	yes	NA	NA
Pavir.5NG478500.v	NA	NA	0	0.460255	0.0001	4.028114	3.667391	0.0598213	-4.063197	0.008861521	0.172158188	down	yes	NA	NA
Pavir.9NG186800.v	GO:000467	00010(Glyc	ribosomal p	0.290449	0.0001	2.710189	2.139639	0.0599091	-4.06108	0.010037188	0.181875162	down	yes	Protein kina	PID
Pavir.5KG655000.v	GO:000456	00520(Ami	chitinase (e	0.309467	0.0001	1.57902	3.554687	0.0603009	-4.051677	0.010778575	0.188149509	down	yes	Acidic endo	-
Pavir.7KG117200.v	NA	NA	0	0.403437	0.0001	4.003635	2.683048	0.0603494	-4.050518	0.010417427	0.185224526	down	yes	NA	NA
Pavir.9NG005700.v	GO:000557	03015(mRN	zinc finger f	2.966272	1.32524	51.642113	19.466003	0.0603519	-4.050456	3.95459E-05	0.004209226	down	yes	GDSL ester	At3g48460
Pavir.4NG340700.v	GO:00082	00100(Ster	delta24-ster	2.412072	1.529635	55.540855	9.266118	0.0608223	-4.039257	7.80475E-05	0.007222851	down	yes	Delta(24)-s	DIM
Pavir.2NG375600.v	GO:000434	00520(Ami	glucosamine	0.389597	0.128309	6.762113	1.736202	0.0609422	-4.036415	0.001838544	0.068873001	down	yes	Glucosamin	GNA1
Pavir.7NG177100.v	GO:000333	NA	transmembr	0.574715	0.0001	6.193053	3.22578	0.0610283	-4.034379	0.011940579	0.197989133	down	yes	Amino acid	AVT1I
Pavir.7NG209200.v	NA	NA	0	19.934097	19.713443	481.3266	165.32076	0.0613125	-4.027676	5.70632E-05	0.00567205	down	yes	NA	NA
Pavir.2KG199300.v	GO:000578	NA	protein of u	0.721941	0.0001	2.451518	9.318352	0.0613466	-4.026874	0.014204268	0.216893787	down	yes	Protein DM	DMP8
Pavir.3KG357500.v	GO:000367	NA	tcp family t	0.2726712	0.7043871	7.5988511	8.3267835	0.0613513	-4.026762	3.85537E-05	0.004124271	down	yes	Transcriptic	TCP5;TCP5
Pavir.7NG059300.v	NA	04626(Plan	leucine-rich	0.159583	0.0001	1.502869	1.097134	0.0614165	-4.025231	0.009409846	0.177199055	down	yes	NA	NA
Pavir.9NG719900.v	GO:000367	NA	yggt family	0.467172	1.283376	15.041784	13.280889	0.0618073	-4.016079	3.26221E-05	0.00359446	down	yes	YlmG hom	YLMG2
Pavir.1NG074700.v	NA	NA	0	0.269074	0.0001	2.282288	2.072615	0.0618094	-4.01603	0.010531306	0.186164311	down	yes	NA	NA
Pavir.1NG389800.v	GO:000557	03010(Ribo	protease inh	27.984444	0.081572	264.52457	189.03357	0.0618796	-4.014391	0.014055974	0.215458932	down	yes	pEARLI1-l	At4g12500
Pavir.9NG778800.v	GO:000367	03013(RNA	f12p19.8 pr	0.326447	0.0001	1.285653	3.983497	0.0619734	-4.012208	0.013720289	0.212670137	down	yes	NAC domai	NAC086
Pavir.3KG084500.v	NA	NA	0	0.317486	0.0001	3.783353	1.334073	0.0620597	-4.010199	0.01271165	0.204721316	down	yes	NA	NA
Pavir.7KG296700.v	NA	NA	0	0.5640368	0.0001	5.6166428	3.4691505	0.06209	-4.009495	0.011805057	0.196988904	down	yes	NA	NA
Pavir.7KG139300.v	GO:19017	00350(Tyrc	tyrosinase	3.372962	0.0001	47.934719	6.25442	0.0622461	-4.005873	0.026456166	0.303152643	down	yes	(+)-larreatri	-
Pavir.9KG094000.v	GO:000016	NA	rna recognit	0.731864	1.336061	29.148993	3.960204	0.0624577	-4.000976	0.000161452	0.012181225	down	yes	Zinc finger	Os07g0682400
Pavir.2KG080500.v	GO:000551	NA	growth-regu	0.1218385	0.2998458	5.4754153	1.2738758	0.0624783	-4.000501	0.000605644	0.032462015	down	yes	Growth-reg	GRF6;GRF6
Pavir.4KG343600.v	GO:000367	NA	protein of u	0.276548	0.180934	5.717363	1.592798	0.0625817	-3.998116	0.000523987	0.029277606	down	yes	NA	NA
Pavir.6NG328700.v	NA	NA	non-specific	0.692592	0.882527	20.550875	4.556413	0.0627355	-3.994573	0.000117045	0.009559058	down	yes	NA	NA
Pavir.7NG022600.v	GO:000449	00380(Tryp	indole-3-py	0.157302	0.054609	2.135561	1.239351	0.0627901	-3.99332	0.000936248	0.044472357	down	yes	Probable in	YUC5
Pavir.3KG337800.v	GO:000557	00040(Pent	pectinestera	0.425134	0.026469	4.241727	2.945734	0.0628321	-3.992355	0.002204339	0.076617601	down	yes	Probable pe	PME53
Pavir.9NG446100.v	NA	NA	0	0.349448	0.090428	4.347055	2.649652	0.062869	-3.991507	0.000271491	0.017990688	down	yes	NA	NA
Pavir.5NG286400.v	GO:000563	04141(Prot	dnaj homolo	0.263886	0.178681	4.470963	2.521233	0.0632944	-3.981778	0.000356234	0.021895072	down	yes	Chaperone	ATJ6
Pavir.4NG334900.v	GO:00082	00100(Ster	delta24-ster	2.139739	1.32732	46.905411	7.812608	0.0633623	-3.980232	9.45276E-05	0.00829833	down	yes	Delta(24)-s	DIM
Pavir.5NG218400.v	NA	NA	0	0.91013	0.477496	16.291819	5.493356	0.0636959	-3.972656	0.000191288	0.013664027	down	yes	NA	NA
Pavir.1NG394100.v	NA	NA	0	0.948212	0.0001	7.651127	7.215703	0.0637871	-3.970591	0.012080787	0.199271271	down	yes	NA	NA
Pavir.7NG161000.v	NA	04626(Plan	adenylate cy	0.36916	0.015105	3.133125	2.882766	0.063875	-3.968605	0.002374742	0.079982686	down	yes	NA	NA
Pavir.9NG830000.v	GO:000904	00500(Starc	periplasmic	0.587421	0.0001	3.435126	5.704262	0.0642845	-3.959385	0.013349661	0.209368875	down	yes	Probable be	BXL7
Pavir.2KG577100.v	GO:000563	04120(Ubic	seven in abs	0.194287	0.200279	4.564048	1.565397	0.0643722	-3.957418	0.000752096	0.038084222	down	yes	E3 ubiquiti	DIS1
Pavir.2NG008500.v	GO:00055	00053(Asco	multi-coppe	1.036389	1.288226	26.530151	9.570414	0.0643928	-3.956958	1.20511E-05	0.001594357	down	yes	L-ascorbate	-
Pavir.7KG282200.v	GO:000557	00040(Pent	pectinestera	4.44663	0.968376	71.140938	12.472946	0.064762	-3.948708	0.000518865	0.029097471	down	yes	Probable pe	PME68
Pavir.6KG408000.v	GO:001034	00073(Cuti	hexadecanal	1.261927	0.223069	13.991452	8.918459	0.0648189	-3.947441	0.000112997	0.009334008	down	yes	Fatty acyl-C	FAR1
Pavir.8KG293000.v	NA	NA	hsp20/alpha	0.312809	0.0001	1.504917	3.322247	0.0648225	-3.947361	0.010811952	0.188267443	down	yes	NA	NA
Pavir.2NG354400.v	GO:000456	00052(Gala	beta-galacto	0.279011	0.214396	5.773655	1.836923	0.0648317	-3.947156	0.000951184	0.044898039	down	yes	Beta-galact	Os01g0875500
Pavir.9KG523900.v	GO:003024	00500(Starc	glucan endo	0.219941	0.166776	4.713732	1.243634	0.0649141	-3.945325	0.000769969	0.038728309	down	yes	Glucan endo	At1g32860
Pavir.6NG244500.v	GO:000578	NA	hva22-like p	0.3525654	0.154609	4.476098	3.335907	0.0649224	-3.945139	0.000444484	0.025772521	down	yes	Protein HV.	HVA22;HVA22
Pavir.9NG654200.v	GO:000551	04075(Plan	cyclin-d5-1	1.257277	0.039233	11.948688	7.852017	0.065478	-3.932847	0.002324054	0.078597368	down	yes	Cyclin-D5-	CYCD5-3
Pavir.3KG036700.v	NA	NA	0	2.691316	0.801008	51.94363	1.376528	0.0654973	-3.932422	0.004503137	0.120338805	down	yes	NA	NA
Pavir.9KG434700.v	GO:000467	04075(Plan	inflorescenc	0.577759	0.057499	6.995676	2.702386	0.0655036	-3.932282	0.000832926	0.040643524	down	yes	Probable LI	IRK
Pavir.3NG151800.v	NA	NA	protein of u	0.365299	0.026325	4.608979	1.369082	0.0655102	-3.932137	0.002991496	0.093277573	down	yes	NA	NA
Pavir.2NG397200.v	GO:000561	03015(mRN	fasciclin do	1.512489	0.021514	15.055062	8.350655	0.0655397	-3.931488	0.005612001	0.13507056	down	yes	Fasciclin-lil	FLA1
Pavir.9KG621400.v	NA	NA	0	0.765971	0.0001	6.931777	4.653601	0.066124	-3.918683	0.013747396	0.213002441	down	yes	Zinc finger	ZFP8
Pavir.6KG201700.v	GO:000563	00966(Gluc	sulfotransfe	0.310388	0.047885	3.836441	1.565415	0.0663241	-3.914324	0.003339849	0.100551399	down	yes	Cytosolic su	SOT5
Pavir.6KG197100.v	NA	NA	0	0.643013	0.0001	7.536248	2.158512	0.0663361	-3.914061	0.018762816	0.250354018	down	yes	NA	NA
Pavir.2NG567400.v	GO:00041	00511(Othe	alpha-l-fuco	0.190702	0.240469	5.145517	1.340129	0.0664808	-3.910918	0.000884705	0.042507709	down	yes	GDSL ester	At3g26430
Pavir.7KG200600.v	GO:000449	00905(Bras	cytochrome	0.6696234	0.0001	6.4057724	3.6395856	0.0666699	-3.90682	0.015804576	0.230800811	down	yes	Cytochrome	CYP724B1;CYP724B1;CYP724B1
Pavir.9NG421300.v	NA	NA	pollen prote	0.845091	0.0001	1.30464	11.366283	0.0667032	-3.9061	0.025052294	0.293602568	down	yes	NA	NA
Pavir.1KG510700.v	GO:000551	NA	sterol regula	1.246618	0.0001	13.119776	5.532312	0.0668407	-3.90313	0.01660609	0.236699774	down	yes	Transcriptic	ILI5
Pavir.2KG541300.v	GO:000373	03010(Ribo	small subun	8.408111	3.430441	147.58113	27.507557	0.0676146	-3.886521	0.000331624	0.020906239	down	yes	40S ribosor	RPS9C
Pavir.5KG623900.v	GO:000419	NA	aspartyl pro	0.507719	0.302567	8.715796	3.212327	0.0679307	-3.879792	0.000139825	0.010945123	down	yes	Aspartic pr	PCS1
Pavir.5KG540800.v	GO:000367	NA	duf814-rela	0.298673	0.321081	4.179466	4.939145	0.0679658	-3.879046	0.00027412	0.018132929	down	yes	NA	NA
Pavir.1KG359300.v	GO:00055	00260(Glyc	primary-am	0.1922482	0.0001	1.4449461	1.3727073	0.0682654	-3.872702	0.015338408	0.226804411	down	yes	Primary am	At1g31672;At1g31672;At1g31672
Pavir.1NG246100.v	NA	NA	0	0.327155	0.0001	3.53566	1.238828	0.0685424	-3.866859	0.017659216	0.243207467	down	yes	NA	NA
Pavir.1KG553100.v	GO:00082	00100(Ster	delta(24)-st	2.3929998	0.3701769	26.265443	14.033551	0.0685669	-3.866344	0.000173969	0.01286717	down	yes	Delta(24)-s	DIM;DIM;DIM;DIM
Pavir.6KG351800.v	NA	NA	0	60.737464	2.614045	507.17899	413.91222	0.0687788	-3.861893	0.002740251	0.087741633	down	yes	NA	NA
Pavir.6KG004400.v	GO:000467	04075(Plan	protein kina	1.823787	0.594359	28.85272	6.230956	0.0689251	-3.858827	0.000170778	0.012706237	down	yes	Proline-rich	PERK9
Pavir.2NG580200.v	GO:000563	00760(Nicc	haloacid del	1.0410107	0.1933305	7.8264928	10.031098	0.0691214	-3.854724	0.000243061	0.016484685	down	yes	NA	NA
Pavir.5KG578800.v	GO:000562	00510(N-G	nuclear tran	1.575762	0.0001	13.178654	9.475957	0.0695603	-3.845592	0.016529591	0.236300643	down	yes	Nuclear tra	NTF2B
Pavir.5KG262900.v	GO:00046	00940(Pher	peroxidase (	0.220609	0.339308	5.500224	2.536182	0.0696726	-3.843266	0.000659548	0.034643898	down	yes	Cationic pe	Sb03g046810
Pavir.6KG059000.v	NA	04626(Plan	leucine-rich	0.230345	0.064511	2.359467	1.833259	0.0703256	-3.829806	0.000518104	0.029097471	down	yes	NA	NA

Pavir.9KG261200.v4	GO:003024	00500(Starc	glucan endo	1.7321957	0.0889592	17.877759	7.9641647	0.0704729	-3.826788	0.001962684	0.071735327	down	yes	Glucan endo	At1g32860;At1g32860
Pavir.5NG219600.v4	GO:000557	00906(Carc	zinc finger f	1.37597	0.392379	11.225197	13.847105	0.07053	-3.82562	7.08441E-05	0.006699435	down	yes	GDSL ester	At5g45910
Pavir.9KG435000.v4	GO:00037(	04141(Prot	bzip transcr	1.231705	0.247022	16.831812	4.067636	0.0707544	-3.821037	0.000725833	0.037104393	down	yes	Transcriptic	RF2b
Pavir.5NG535500.v4	GO:004665	NA	plastocyanin	9.176412	0.103548	39.231071	91.909164	0.0707636	-3.820848	0.007786637	0.160731145	down	yes	Early nodul	ENODL1
Pavir.6NG139800.v4	GO:000817	00402(Benz	o-methyltran	0.179991	0.0001	1.082081	1.455981	0.0709561	-3.816929	0.015248133	0.226367538	down	yes	Acetylserotc	ASMT3
Pavir.6KG132400.v4	GO:000455	00500(Starc	glucan endo	0.607556	0.0001	3.088441	5.431389	0.0713225	-3.809498	0.017654031	0.243207467	down	yes	Glucan endo	At1g64760
Pavir.7KG372800.v4	GO:000588	NA	mlo-like prc	0.119113	0.048117	1.1865623	1.154682	0.0714278	-3.80737	0.001123672	0.049787424	down	yes	MLO-like p	MLO14;MLO14
Pavir.3KG132200.v4	NA	NA	0	0.486224	0.153638	7.168025	1.786796	0.0714545	-3.806832	0.001364004	0.056978853	down	yes	Nuclear env	NEAP2
Pavir.8NG018400.v4	GO:000367	03018(RNA	no apical m	0.195298	0.0001	1.341632	1.381855	0.0717455	-3.800967	0.014088439	0.215619109	down	yes	NAC domai	NAC45
Pavir.4NG209500.v4	GO:000436	00480(Glut	glutathione	1.754817	0.0001	11.191832	13.252569	0.0717922	-3.800029	0.016666427	0.237199872	down	yes	Probable gl	GSTU1
Pavir.5KG450700.v4	GO:000573	NA	cystathionin	0.408117	0.0001	1.716656	3.96323	0.0718706	-3.798454	0.017142301	0.240690464	down	yes	CBS domai	CBSX3
Pavir.3KG321200.v4	GO:000367	03030(DNA	flap endonu	0.082564	0.673289	9.375458	1.135554	0.0719106	-3.797652	0.0055254	0.134588386	down	yes	Flap endonu	FEN1
Pavir.2NG452800.v4	GO:000367	NA	protein of u	0.9200791	0.0001	9.8067709	2.9515301	0.072124	-3.793378	0.023191335	0.281068051	down	yes	Protein AL7	APSR1;APSR1
Pavir.2NG181700.v4	NA	NA	0	0.62885	0.358499	5.502891	8.174254	0.0721897	-3.792063	0.000113033	0.009334008	down	yes	NA	NA
Pavir.4NG340200.v4	GO:000557	00500(Starc	glycosyltran	0.945145	0.0001	2.519042	10.565596	0.0722408	-3.791042	0.023914569	0.285564774	down	yes	Sucrose syn	SUS7
Pavir.9NG248900.v4	GO:00055(	00053(Asco	multi-coppe	0.9523285	0.0836129	10.007006	4.3253917	0.0722797	-3.790266	0.000997288	0.046014367	down	yes	Monocoppe	SKU5;SKU5;SKU5;SKU5
Pavir.3NG318700.v4	NA	NA	0	0.965002	0.31866	12.878084	4.866345	0.0723417	-3.789029	0.000871081	0.041906675	down	yes	Thaumatin-	-
Pavir.2NG167800.v4	GO:000904	00500(Starc	periplasmic	0.434573	0.0001	2.287927	3.712352	0.0724421	-3.787027	0.01793603	0.244825273	down	yes	Probable be	BXL7
Pavir.9KG433800.v4	NA	NA	0	2.650752	0.459372	26.68762	15.741517	0.0733016	-3.770011	0.000236638	0.016107256	down	yes	NA	NA
Pavir.7NG248700.v4	GO:000467	04075(Plan	protein kina	1.514301	0.680522	18.618126	11.308069	0.0733412	-3.769232	2.07492E-05	0.002531205	down	yes	Probable in	RLK902
Pavir.4KG082200.v4	GO:000497	NA	glutamate re	0.0978488	0.0804728	1.3914769	1.0370274	0.0734286	-3.767514	0.001501184	0.060454431	down	yes	Glutamate r	GLR2.9;GLR2.9;GLR2.9;GLR2.8
Pavir.2NG493300.v4	GO:000404	00330(Argi	amidase//su	6.616126	1.0340768	77.412054	26.733195	0.0734571	-3.766955	0.000746313	0.037944792	down	yes	Probable an	At4g34880;At4g34880
Pavir.5NG493200.v4	GO:000457	00510(N-G	dolichyl-dip	1.088027	0.58564	16.273901	6.224019	0.0743921	-3.748707	3.69265E-05	0.003977267	down	yes	Dolichyl-dip	STT3A
Pavir.7NG390600.v4	GO:00046(	00940(Pher	peroxidase (	1.590879	0.055639	16.675982	5.422393	0.0745086	-3.74645	0.004387906	0.118484849	down	yes	Peroxidase	PER16;PER16
Pavir.9NG284400.v4	NA	NA	0	0.308605	0.077623	3.267974	1.87744	0.0750626	-3.735762	0.00293485	0.09219992	down	yes	NA	NA
Pavir.1NG259100.v4	GO:000403	00010(Glyc	apospory-as	0.324434	0.185601	5.534601	1.26007	0.075064	-3.735736	0.001515735	0.060844773	down	yes	Putative glu	-
Pavir.8KG044200.v4	GO:000367	04144(Endo	protein of u	1.320626	0.0001	3.677663	13.844434	0.0753749	-3.729772	0.02805654	0.311277434	down	yes	NA	NA
Pavir.7NG155100.v4	GO:000716	NA	ankyrin repe	0.178691	0.0001	1.28326	1.084914	0.0754974	-3.727429	0.016011597	0.232455648	down	yes	NA	NA
Pavir.3KG315300.v4	GO:000842	00500(Starc	glycosyl hyc	1.1749845	0.7500303	14.835044	10.620317	0.0756232	-3.725028	1.73228E-05	0.002169561	down	yes	Beta-glucos	BGLU20;BGLU20;BGLU20;BGLU20
Pavir.3NG176200.v4	NA	NA	0	1.0941883	0.0186777	2.6380794	12.033094	0.0758539	-3.720633	0.009174691	0.1749902	down	yes	NA	NA
Pavir.9NG517700.v4	GO:000449	00905(Bras	cytochrome	4.858522	5.898855	104.64967	36.896049	0.0759993	-3.71787	0.00011513	0.009465628	down	yes	Cytochrome	CYP724B1
Pavir.9NG076300.v4	NA	NA	domain of u	1.042576	0.0001	9.761998	3.911036	0.0762578	-3.712971	0.023230069	0.281238111	down	yes	NA	NA
Pavir.7KG296400.v4	NA	NA	0	0.2694299	0.0618846	2.8311317	1.4979853	0.0765317	-3.707799	0.003365664	0.10108875	down	yes	NA	NA
Pavir.7KG315800.v4	GO:000577	04120(Ubic	f12p19.9 pr	0.649614	0.161308	6.606553	3.984394	0.0765675	-3.707125	0.000180573	0.013136497	down	yes	PH, RCC1	PRAF1
Pavir.6NG277500.v4	GO:000468	04626(Plan	serine/threo	0.108649	0.054987	1.036416	1.095835	0.0767433	-3.703815	0.002066869	0.073749738	down	yes	Serine/threc	PEPKR2
Pavir.7KG276800.v4	NA	NA	cotton fibre	0.263296	0.0001	1.357912	2.067486	0.076895	-3.700966	0.019867485	0.258476809	down	yes	NA	NA
Pavir.2NG599600.v4	GO:000373	03010(Ribo	small subun	5.128102	3.582003	95.442299	17.419127	0.0771752	-3.695719	0.000214035	0.014892482	down	yes	40S ribosor	RPS9C
Pavir.1NG330400.v4	GO:000371	00903(Lim	btb/poz and	0.1605742	0.0947786	1.9628955	1.3423327	0.0772573	-3.694186	0.000850684	0.041242269	down	yes	BTB/POZ a	BT1;BT1;NA;BT1
Pavir.7NG155200.v4	GO:000716	NA	ankyrin repe	0.163803	0.0001	1.103234	1.012159	0.0774811	-3.690012	0.018744876	0.250284723	down	yes	NA	NA
Pavir.7KG374900.v4	GO:000561	NA	disease resis	0.507014	0.0001	2.190898	4.332859	0.0777334	-3.685321	0.021355976	0.269063303	down	yes	NA	NA
Pavir.1KG476500.v4	GO:000387	00051(Fruc	phosphofruc	7.7656865	0.1521236	72.098929	29.743451	0.0777457	-3.685093	0.008779812	0.17119038	down	yes	Pyrophosph	PFP-ALPHA;PFP-ALPHA
Pavir.5KG563500.v4	GO:000425	04144(Endo	serine prote	0.3229949	0.0373591	3.4430026	1.1757479	0.0780198	-3.680016	0.002592113	0.084985585	down	yes	Subtilisin-li	SBT2.2;SBT2.3;SBT2.2
Pavir.8KG175900.v4	GO:00009(	03010(Ribo	extensin-lik	0.623407	0.122579	8.547944	1.00275	0.078108	-3.678385	0.00366846	0.106931773	down	yes	Leucine-ricl	LRX1
Pavir.7KG026700.v4	NA	NA	0	0.194055	0.183165	3.087986	1.729397	0.0783039	-3.674772	0.002278477	0.077786442	down	yes	NA	NA
Pavir.9KG508800.v4	NA	04144(Endo	uncharacteri	0.421377	0.0001	1.111673	4.26422	0.0784013	-3.672979	0.025851573	0.298500657	down	yes	NA	NA
Pavir.8KG332800.v4	GO:000418	00460(Cyar	serine prote	1.767561	0.0001	10.369087	12.147174	0.078506	-3.671054	0.026197989	0.301481486	down	yes	Serine carbo	SCPL45
Pavir.6NG296500.v4	GO:000016	NA	extracellular	3.22972	0.412317	32.01823	14.338045	0.0785662	-3.669947	0.000636279	0.033719184	down	yes	DNA damag	DRT100
Pavir.7KG293800.v4	GO:000425	04144(Endo	subtilisin se	0.220533	0.962726	12.958457	2.096406	0.0785965	-3.669392	0.001660629	0.064442719	down	yes	Subtilisin-li	SBT1.3
Pavir.9KG124100.v4	GO:000388	00053(Asco	d-arabinono	0.206446	0.037616	1.643651	1.46066	0.0786203	-3.668954	0.003139206	0.096442669	down	yes	L-gulonolac	GULLO2
Pavir.7KG328200.v4	NA	NA	s100 calciu	0.353592	0.055983	1.163359	4.027525	0.0789027	-3.663781	0.005339963	0.131652506	down	yes	NA	NA
Pavir.2NG502400.v4	GO:000563	NA	protein of u	0.622897	0.929368	7.702006	11.963182	0.0789347	-3.663197	0.000433521	0.025253752	down	yes	NA	NA
Pavir.7NG114200.v4	GO:000969	NA	nucleoporin	8.018913	0.247593	54.695923	49.962357	0.0789857	-3.662265	0.004629249	0.122629853	down	yes	NA	NA
Pavir.4NG340900.v4	NA	NA	extensin, pr	5.849774	0.254303	45.625652	31.130598	0.0795255	-3.652439	0.002841486	0.090108622	down	yes	NA	NA
Pavir.2NG175100.v4	GO:000003	00062(Fatty	3-ketoacyl-c	0.232215	0.0001	1.621677	1.294636	0.0796605	-3.649991	0.01992271	0.258492149	down	yes	3-ketoacyl-(	KCS17
Pavir.2KG091800.v4	GO:000573	00073(Cuti	transferase f	1.259377	0.200966	11.063783	7.268255	0.0796607	-3.649988	0.000996445	0.046014367	down	yes	Acyl transfe	AT15
Pavir.1NG441400.v4	GO:000487	04120(Ubic	cell division	1.780779	2.492326	49.983234	3.046684	0.0805791	-3.63345	0.002062045	0.073647555	down	yes	Cell divisio	CDC20-1
Pavir.7KG406500.v4	NA	NA	0	0.558669	0.054675	3.839236	3.765444	0.0806535	-3.632119	0.003022769	0.093940865	down	yes	NA	NA
Pavir.8NG240000.v4	GO:000626	03030(DNA	dna replicat	0.669505	1.13011	20.199976	2.01512	0.0810087	-3.62578	0.000982695	0.045866809	down	yes	DNA replic	OsI_36121
Pavir.1KG018800.v4	GO:000497	NA	ionotropic g	0.275029	0.049668	2.8020663	1.200436	0.0811235	-3.623736	0.002449385	0.081878002	down	yes	Glutamate r	GLR3.1;GLR3.1
Pavir.9KG424100.v4	GO:000467	04075(Plan	inflorescenc	1.784709	0.029546	15.671177	6.683799	0.0811567	-3.623147	0.008047258	0.162733919	down	yes	Probable in	At5g10020;At5g10020
Pavir.7NG299800.v4	GO:000588	03015(mRN	fasciclin-lik	0.703608	1.970269	23.93779	8.994017	0.0811944	-3.622477	0.000148333	0.011467723	down	yes	Fasciclin-lil	FLA10
Pavir.8NG020600.v4	GO:000557	NA	protease inh	63.938984	1.180421	438.72714	362.99945	0.081224	-3.621951	0.009449701	0.177692771	down	yes	Non-specifi	LTP110-A
Pavir.6NG331200.v4	GO:000864	NA	rag1-activat	2.853867	0.03416	14.360443	21.160248	0.0813055	-3.620504	0.009278662	0.175807947	down	yes	Bidirectiona	SWEET11
Pavir.2KG133600.v4	GO:000487	NA	late embryo	0.061605	0.16231	1.348761	1.398093	0.0815169	-3.616757	0.003760376	0.108516589	down	yes	NA	NA
Pavir.4KG349900.v4	GO:000367	NA	protein of u	0.228955	0.138687	3.253199	1.251787	0.0816078	-3.615149	0.002190651	0.07621234	down	yes	NA	NA
Pavir.2NG274100.v4	GO:000573	NA	cyclopropan	0.9444709	0.5637524	13.889322	4.5541194	0.0817756	-3.612186	8.59135E-05	0.007741071	down	yes	NA	NA
Pavir.9KG405900.v4	GO:000373	03010(Ribo	small subun	0.790899	0.0001	8.183018	1.447112	0.0821379	-3.605807	0.035705541	0.355331178	down	yes	40S ribosor	RPS9C
Pavir.2NG415800.v4	GO:000367	03013(RNA	no apical m	0.287672	0.034082	1.900839	2.011497	0.0822409	-3.604	0.006018123	0.140446545	down	yes	NAC domai	NAC086
Pavir.9NG357500.v4	GO:000367	NA	phosphate-i	0.523686	0.0001	1.491947	4.869022	0.0823437	-3.602197	0.028773386	0.315650346	down	yes	Protein PH(	PHI-1
Pavir.5NG313000.v4	GO:000367	NA	protein of u	0.774047	0.0001	6.595404	2.785337	0.0825251	-3.599022	0.028598163	0.314278675	down	yes	NA	NA
Pavir.2NG519300.v4	GO:000455	04075(Plan	xyloglucan (	1.552966	0.285441	18.022282	4.114195	0.0830488	-3.589898	0.001666639	0.064442719	down	yes	Probable xy	XTH8
Pavir.1KG483500.v4	GO:000509	04120(Ubic	immunoglol	2.707709	0.414448	26.823101	10.742638	0.0831118	-3.588802	0.000733222	0.037380378	down	yes	Rho GDP-d	GDI1
Pavir.1KG464400.v4	GO:000487	04120(Ubic	cell division	0.9893866	2.6319576	42.115573	1.2595672	0.0834889	-3.582271	0.006614422	0.147491786	down	yes	Cell divisio	CDC20-1;CDC20-1
Pavir.9NG185800.v4	GO:000557	NA	pollen allerg	0.4682395	0.0475861	4.7584472	1.3884665	0.0839162	-3.574907	0.005059661	0.128340138	down	yes	Expansin-B	EXPB12;EXPB12;EXPB12;EXPB12;EXPB12;EXPB12;EXPB12;EXPB12;EXPB12;EXPB12;EXPB12;EXPB12;EXPB12
Pavir.5NG489500.v4	NA	NA	0	0.181138	0.149116	2.671293	1.259683	0.0840132	-3.57324	0.003808418	0.109148517	down	yes	NA	NA
Pavir.5KG536700.v4	GO:000367	NA	duf814-rela	0.579115	0.460563	6.640738	5.728683	0.0840523	-3.572569	0.000452619	0.026163463	down	yes	NA	NA
Pavir.9NG590600.v4	NA	NA	0	4.029408	0.244131	31.224493	19.592999	0.0840958	-3.571822	0.002317084	0.078562572	down	yes	NA	NA
Pavir.3KG094000.v4	GO:000377	NA	formin-relat	0.557386	0.278207	8.147134	1.788474	0.0841008	-3.571736	0.000991668	0.046014367	down	yes	Formin-like	FH14
Pavir.5KG645200.v4	GO:000485	NA	plant inverta	0.965781	0.086186	7.037946	5.400133	0.0845763	-3.563602	0.00657138	0.147055664	down	yes	Pectinestera	PMEI28
Pavir.9KG243800.v4	GO:000367	NA	pathogenesi	3.829217	0.0001	34.471352	10.799875	0.0845861	-3.563435	0.039399093	0.374391025	down	yes	Thaumatin-	TLP1
Pavir.5KG206200.v4	GO:000904	00500(Starc	periplasmic	2.658098	0.0001	21.163815	10.259322	0.0845937	-3.563307	0.038851984	0.371444599	down	yes	Beta-xylosi	Xyl1
Pavir.1NG245800.v4	GO:004746	00909(Sesq	beta-sesquip	1.440786	0.103989	12.604797	5.640332	0.0846678	-3.562043	0.003231256	0.09822653	down	yes	Alpha-terpi	TPS26

Pavir.4NG334300.v4	GO:000557	00500(Starc	glycosyltran	1.202003	0.0001	3.552576	10.602239	0.0849254	-3.55766	0.034927573	0.351549346	down	yes	Sucrose syn	SUS7
Pavir.3NG076900.v4	GO:000573	NA	ras suppress	2.091531	0.130698	11.44532	14.537791	0.0855259	-3.547495	0.002096468	0.073961786	down	yes	NA	NA
Pavir.9NG037100.v4	GO:000573	03013(RNA	chloroplast-	5.152466	0.355843	40.222393	23.964149	0.0858172	-3.542589	0.001951883	0.071479622	down	yes	Protein SOI	NAKR2
Pavir.4KG213300.v4	GO:000469	04075(Plan	cycline//sub	0.215514	0.0001	1.279052	1.224632	0.0861187	-3.53753	0.021710635	0.270884721	down	yes	Cyclin-D3-	CYCD3-1
Pavir.4KG059800.v4	GO:000969	NA	nucleoporin	0.416243	0.0001	1.694235	3.12484	0.0863948	-3.532912	0.027004408	0.306076323	down	yes	NA	NA
Pavir.3NG054000.v4	GO:000484	NA	von willebra	2.024467	1.469886	26.109766	14.327173	0.0864149	-3.532577	3.20904E-05	0.003546271	down	yes	NA	NA
Pavir.4KG204300.v4	GO:000367	00563(Glyc	extensin, pr	16.94812	0.413951	148.45207	51.511482	0.0868262	-3.525726	0.010288071	0.184424471	down	yes	Protein GAS	GAST1
Pavir.7NG308400.v4	GO:000472	00010(Glyc	protein phos	0.350491	0.088882	3.803716	1.249294	0.0869527	-3.523625	0.004004327	0.112447366	down	yes	Probable pr	Os04g0584300
Pavir.9NG627500.v4	NA	NA	0	0.85625	0.0001	7.23103	2.614056	0.0869825	-3.523131	0.033296289	0.34213558	down	yes	NA	NA
Pavir.5KG002800.v4	GO:000367	NA	awpm-19-li	0.494686	0.0001	1.520217	4.161646	0.0870816	-3.521487	0.030512551	0.326623382	down	yes	NA	NA
Pavir.8NG311500.v4	GO:000573	NA	protein of u	2.278366	1.205266	7.327444	32.658897	0.0871205	-3.520843	0.000511006	0.028828877	down	yes	NA	NA
Pavir.5KG334900.v4	GO:000551	NA	vacuolar cat	0.393207	0.017272	1.724741	2.984387	0.0871667	-3.52008	0.008472561	0.167370951	down	yes	Vacuolar ca	CAX1a
Pavir.8KG018900.v4	NA	NA	mannan end	0.713854	0.041895	2.06709	6.599375	0.0872038	-3.519465	0.009556578	0.178730357	down	yes	NA	NA
Pavir.9KG433500.v4	NA	NA	0	3.010539	0.26805	26.632633	10.829774	0.0875168	-3.514297	0.00279408	0.089194546	down	yes	Transcriptic	RF2b
Pavir.5KG202800.v4	GO:000552	03008(Ribo	mitochondri	0.6198116	0.0001	5.9276659	1.1492505	0.0875963	-3.512986	0.041385274	0.382804081	down	yes	AAA-ATPa	At5g17760;At5g17760
Pavir.6NG109400.v4	GO:000467	04075(Plan	non-specific	0.264487	0.0001	1.999257	1.019357	0.0876518	-3.512072	0.025183406	0.294312939	down	yes	Receptor-lil	HSL1
Pavir.7KG320500.v4	GO:004316	NA	e3 ubiquitin	0.274459	0.036402	1.432755	2.109907	0.0877479	-3.510492	0.007133897	0.155046644	down	yes	E3 ubiquiti	ATL4
Pavir.9NG730500.v4	GO:000013	NA	glucomanna	0.533482	0.053633	4.515017	2.165421	0.0878857	-3.508228	0.004888281	0.12631869	down	yes	Probable gl	CSLA2
Pavir.1NG462300.v4	NA	NA	0	0.222317	0.0001	1.479538	1.047904	0.0880008	-3.506339	0.023187493	0.281068051	down	yes	NA	NA
Pavir.2KG514600.v4	GO:000370	04075(Plan	protein shor	0.404653	0.283838	5.898868	1.923002	0.0880213	-3.506004	0.000418578	0.0245738	down	yes	Protein SHO	SHR1
Pavir.9NG463100.v4	GO:000090	NA	protein bran	0.774516	0.084667	5.794528	3.961539	0.0880665	-3.505262	0.005582389	0.134904319	down	yes	NA	NA
Pavir.7KG305100.v4	GO:000577	04120(Ubic	f12p19.9 pr	0.502332	0.038763	3.86216	2.272766	0.0881991	-3.503092	0.003885484	0.110347156	down	yes	PH, RCC1	PRAF1
Pavir.3KG069200.v4	NA	NA	rho gtpase a	0.262025	0.185438	2.856147	2.208112	0.0883571	-3.500511	0.002029799	0.07291171	down	yes	NA	NA
Pavir.2NG151200.v4	NA	NA	0	0.407738	0.802302	12.242041	1.413743	0.0886101	-3.496385	0.007323673	0.156441785	down	yes	NA	NA
Pavir.7KG290300.v4	GO:000563	NA	protein of u	0.442394	0.128423	2.780017	3.631995	0.0890231	-3.489677	0.000947402	0.044775752	down	yes	Protein ROI	REL2
Pavir.1NG526000.v4	GO:000155	04075(Plan	lrr receptor-	2.964154	0.47533	32.87327	5.707557	0.0891501	-3.48762	0.002642261	0.085806122	down	yes	LRR recept	ER1;ER1
Pavir.1NG367900.v4	GO:000694	NA	pollen allerg	1.1769	0.0001	8.984579	4.214513	0.0891728	-3.487252	0.037134936	0.362973709	down	yes	Expansin-B	EXPB16
Pavir.5NG019300.v4	GO:000367	04626(Plan	shn shine ,	0.481416	0.004524	4.00186	1.446799	0.0891852	-3.487051	0.022144188	0.274323631	down	yes	Ethylene-res	ERF3
Pavir.1KG200600.v4	NA	NA	protein of u	7.446146	0.810548	68.306366	24.203447	0.0892521	-3.48597	0.001936757	0.071273036	down	yes	NA	NA
Pavir.5KG166200.v4	GO:000049	03008(Ribo	h/aca ribonu	0.875089	0.902375	18.068644	1.843598	0.0892649	-3.485763	0.001215795	0.052688678	down	yes	H/ACA ribo	CBF5;CBF5
Pavir.4NG333500.v4	GO:000014	00500(Starc	lyst-interact	1.294875	0.0001	10.624114	3.882643	0.089267	-3.485729	0.048026271	0.413305336	down	yes	Callose syn	CALS3
Pavir.5KG354300.v4	GO:000557	NA	domain of u	0.762357	0.0001	4.442164	4.090606	0.0893563	-3.484286	0.030621917	0.327141682	down	yes	NA	NA
Pavir.1NG262200.v4	GO:000436	00480(Glut	glutathione	0.454086	1.724212	21.847677	2.478501	0.0895454	-3.481236	0.003869553	0.110144475	down	yes	Glutathione	-
Pavir.2KG571500.v4	GO:001602	00909(Sesq	premnaspiro	0.508758	0.226737	4.562934	3.6471209	0.0895847	-3.480604	0.000687158	0.035710334	down	yes	Premnaspiro	CYP71D55;CYP71D55;CYP71D55
Pavir.6NG138800.v4	GO:000367	NA	arabinanase	0.5724513	0.12021	5.011233	2.711007	0.0896969	-3.478797	0.002723976	0.087741633	down	yes	NA	NA
Pavir.9NG681600.v4	GO:000550	01230(Bios	ll-diaminop	0.56827	0.0001	4.212229	2.101016	0.0900282	-3.473479	0.03277173	0.339131364	down	yes	Probable LI	AGD2;AGD2
Pavir.7NG329400.v4	GO:003152	00902(Mon	(+)-neoment	1.086797	2.733034	29.677137	12.728152	0.0900791	-3.472664	0.000304069	0.019596514	down	yes	(+)-neomen	SDR1
Pavir.5NG245700.v4	NA	NA	0	0.273446	0.0001	1.676442	1.353931	0.0902681	-3.46964	0.027951099	0.31062013	down	yes	NA	NA
Pavir.3NG309200.v4	GO:000367	NA	b3 dna bind	0.202761	0.0001	1.132332	1.112799	0.090356	-3.468236	0.026750494	0.304574637	down	yes	B3 domain-	Os12g0591400
Pavir.8KG100000.v4	GO:000820	00100(Stero	delta24-ster	18.538374	4.125769	194.03539	56.446018	0.0904823	-3.46622	0.001665154	0.064442719	down	yes	Delta(24)-st	DIM
Pavir.5KG357000.v4	GO:000557	NA	domain of u	0.991031	0.044645	6.596796	4.840496	0.0905526	-3.465101	0.005834356	0.137393622	down	yes	NA	NA
Pavir.2KG489800.v4	NA	NA	0	0.742097	0.455975	6.845631	6.376882	0.0906085	-3.46421	0.002279373	0.077786442	down	yes	NA	NA
Pavir.9NG609000.v4	GO:000438	00230(Puri	guanylate ki	0.590439	0.34519	5.697293	4.589752	0.0909522	-3.458748	0.000772239	0.038790561	down	yes	Guanylate k	V2
Pavir.1KG481600.v4	GO:000509	04120(Ubic	immunoglol	0.319359	0.052597	2.973964	1.112564	0.0910201	-3.457672	0.008085306	0.16315209	down	yes	Rho GDP-d	GDI1
Pavir.7KG191500.v4	GO:000467	04075(Plan	protein kina	0.686005	0.253222	5.781206	4.520339	0.0911734	-3.455243	0.000205936	0.014528109	down	yes	Probable in	RLK902
Pavir.6NG153400.v4	GO:000367	NA	(phi-1) prot	5.961644	0.540571	45.397064	25.81267	0.0913108	-3.453071	0.001696807	0.065321851	down	yes	Protein PHO	PHI-1
Pavir.8NG103500.v4	GO:000563	00040(Pent	helix-loop-h	0.389446	0.0001	2.952151	1.305331	0.0914968	-3.450135	0.035377484	0.354053501	down	yes	Transcriptic	SCRM
Pavir.5NG272800.v4	GO:000455	00500(Starc	carbohydrat	1.926068	0.0001	8.558174	12.43113	0.091769	-3.445849	0.035781664	0.35559835	down	yes	Glucan endo	At4g34480
Pavir.2KG253500.v4	GO:000557	00511(Othe	zinc finger f	3.640152	0.109594	23.066118	17.775356	0.0918122	-3.44517	0.006331808	0.14453524	down	yes	GDSL ester	At2g27360
Pavir.7NG091000.v4	GO:000557	00500(Starc	glycosyltran	4.2817704	0.0318786	10.97745	35.931527	0.0919579	-3.442883	0.02265018	0.27730049	down	yes	Sucrose syn	SUS7;SUS7
Pavir.6KG355200.v4	GO:000467	04075(Plan	leucine-rich	1.466528	0.441112	15.907547	4.796937	0.0921366	-3.440082	0.00057009	0.031088546	down	yes	Receptor pr	ZAR1
Pavir.5NG592900.v4	GO:000367	NA	awpm-19-li	2.855741	0.0001	12.286715	18.64814	0.0923179	-3.437246	0.033593203	0.343566433	down	yes	Membrane p	PM19L
Pavir.9NG104000.v4	GO:000815	NA	bax inhibito	0.377228	0.0001	1.003789	3.074682	0.092517	-3.434137	0.035472776	0.3540943	down	yes	Protein LIF	LFG4
Pavir.5KG051000.v4	GO:000367	04141(Prot	x-box transc	5.111308	0.0001	28.732634	26.406826	0.0926996	-3.431292	0.044217988	0.397274622	down	yes	Basic leucir	BZIP06
Pavir.5KG448800.v4	GO:000455	00500(Starc	carbohydrat	1.308501	0.030233	3.170772	11.259966	0.0927696	-3.430204	0.014228303	0.217052385	down	yes	Glucan endo	At4g34480;At4g34480
Pavir.7KG208400.v4	GO:000422	04144(Endo	mitochondri	0.587292	0.640619	11.077462	2.154494	0.0927989	-3.429748	0.00157425	0.062296446	down	yes	NA	NA
Pavir.3NG297600.v4	GO:000551	NA	speckle-type	1.2699936	0.0001	2.3454216	11.321139	0.0929344	-3.427643	0.042505709	0.388959333	down	yes	BTB/POZ a	BPM2;BPM2;BPM2
Pavir.5KG674200.v4	GO:000663	01212(Fatty	acyl-[acyl-ca	0.573161	0.0001	4.122679	2.032646	0.0931325	-3.424571	0.035961771	0.35660903	down	yes	Stearoyl-[ac	OsI 04666
Pavir.1KG531000.v4	NA	NA	0	4.151159	0.0001	19.644592	24.869835	0.0932565	-3.422652	0.035397733	0.354053501	down	yes	NA	NA
Pavir.9KG048100.v4	GO:000097	04712(Circ	transcriptio	0.588699	0.420117	5.642186	5.16249	0.0933685	-3.420921	0.000810842	0.04019688	down	yes	GATA trans	GATA18
Pavir.7KG256200.v4	GO:000695	NA	leucine rich	0.226405	0.036146	1.493573	1.315112	0.0934783	-3.419225	0.004952842	0.127009363	down	yes	Receptor-lil	RLP35
Pavir.2KG488200.v4	GO:000970	NA	transporter l	5.986901	0.18219	35.722378	29.772762	0.0941916	-3.408258	0.007233654	0.156279779	down	yes	Organic cat	44106
Pavir.1KG017900.v4	GO:000837	00603(Glyc	cmp-n-acety	0.589666	0.404081	8.481183	2.066043	0.0942188	-3.407841	0.001607731	0.063114666	down	yes	Sialyltransfe	STLP4
Pavir.1KG239000.v4	NA	NA	0	0.832073	0.38538	5.506327	7.407829	0.0942727	-3.407015	0.000185274	0.013387082	down	yes	NA	NA
Pavir.2KG035000.v4	GO:000455	00500(Starc	carbohydrat	0.032217	0.48781	4.100105	1.392546	0.0946769	-3.400844	0.016578713	0.23662268	down	yes	Glucan endo	At5g56590
Pavir.5KG192000.v4	NA	NA	0	1.952308	0.353934	13.816794	10.539988	0.0946858	-3.400708	0.001795575	0.067752155	down	yes	NA	NA
Pavir.2NG003700.v4	GO:000377	03008(Ribo	kinesin fam	1.36822	0.229856	14.799186	2.059793	0.0947908	-3.399109	0.004159913	0.114964127	down	yes	Kinesin-like	KIN14N
Pavir.3NG106400.v4	GO:000953	01200(Carb	ribulose-bis	5.47986	1.641807	8.595643	66.459373	0.094886	-3.397662	0.002090022	0.073943863	down	yes	Ribulose bi	RBCS-A
Pavir.9KG509100.v4	GO:000573	NA	rho/rac/cdc	0.503824	0.2841398	6.5796533	1.7245448	0.0948874	-3.39764	0.000831532	0.040643524	down	yes	Rho GTPas	REN1;REN1;REN1;REN1
Pavir.7KG370300.v4	GO:000550	NA	c2 calcium/l	2.547504	0.213585	18.738142	10.340813	0.0949515	-3.396666	0.002273125	0.077785179	down	yes	FT-interacti	FTIP3
Pavir.1NG355800.v4	GO:000469	03022(Basa	cyclin-deper	1.165324	0.24114	13.253541	1.516307	0.0952254	-3.39251	0.007357739	0.156828349	down	yes	Cyclin-depe	CDKB1-1
Pavir.1KG066200.v4	GO:004665	NA	plastocyanin	1.791614	0.0001	6.814263	11.937134	0.095551	-3.387586	0.037681757	0.365544935	down	yes	Early nodul	At5g25090
Pavir.8NG056900.v4	GO:000333	04075(Plan	auxin transp	0.099431	0.509527	1.700542	4.668351	0.0956144	-3.386628	0.006267281	0.144060155	down	yes	Putative au	Os11g0169200
Pavir.3NG166800.v4	GO:000573	NA	sec14 relate	0.421748	0.408944	2.385767	6.266022	0.0960139	-3.380613	0.001242416	0.053533583	down	yes	NA	NA
Pavir.1NG020000.v4	GO:000552	NA	arabinose-5	11.809457	0.234318	76.207169	49.073864	0.0961341	-3.378808	0.013274877	0.209130801	down	yes	Probable ar	SETH3
Pavir.2NG313400.v4	NA	NA	0	2.025975	0.03272	12.652911	8.755227	0.0961641	-3.378357	0.01329991	0.209349606	down	yes	NA	NA
Pavir.2KG014700.v4	GO:004665	NA	plastocyanin	0.55016	0.0001	1.016204	4.704108	0.0961941	-3.377908	0.043041404	0.39128833	down	yes	NA	NA
Pavir.9NG708600.v4	GO:000550	01230(Bios	ll-diaminop	0.250358	0.257634	3.100113	2.1727091	0.0963416	-3.375698	0.001460975	0.059472612	down	yes	Probable LI	AGD2;AGD2
Pavir.9KG562200.v4	NA	NA	0	1.759892	0.685374	18.932465	6.444139	0.0963591	-3.375436	0.000423078	0.024799226	down	yes	NA	NA
Pavir.9KG615500.v4	GO:001678	NA	alpha/beta-h	0.814789	0.467106	10.961987	2.326907	0.0964636	-3.373871	0.001978774	0.071903748	down	yes	NA	NA
Pavir.2NG613700.v4	GO:000483	04120(Ubic	ubiquitin-ac	0.617242	0.303231	4.594117	4.923205	0.0967155	-3.370108	0.000213909	0.014892482	down	yes	Ubiquitin-a	UBA3
Pavir.1NG003500.v4	GO:000551	NA	bag family r	14.930034	14.613317	234.79453	70.668961	0.0967165	-3.370095	0.000670134	0.034976986	down	yes	BAG family	BAG7

Pavir.5KG062900.v4	GO:000519	03010(Ribo	leucine rich	1.078282	0.074274	9.478866	2.421914	0.0968471	-3.368147	0.008040813	0.162733919	down	yes	Leucine-ricl	LRX2
Pavir.7KG110600.v4	GO:000455	NA	glycosyl hyc	0.9237064	0.7529141	8.1653252	9.1398587	0.0968855	-3.367576	9.16505E-05	0.008140857	down	yes	Endo-1,4-be	At4g33820;At4g33820
Pavir.5KG336700.v4	GO:000467	04075(Plan	lrr receptor-	5.267628	1.015301	38.218437	26.348801	0.0973083	-3.361293	0.001057318	0.048037018	down	yes	LRR recept	SIK1
Pavir.3KG494500.v4	GO:000367	NA	thaumatin fa	0.103233	1.595179	13.265148	4.187659	0.0973145	-3.361201	0.008928388	0.172943049	down	yes	NA	NA
Pavir.1NG453500.v4	NA	NA	0	0.192001	0.063948	1.143249	1.469045	0.0979786	-3.351389	0.005236513	0.130731907	down	yes	NA	NA
Pavir.6KG099000.v4	GO:000552	04626(Plan	leucine-rich	0.22383	0.045369	1.088966	1.657958	0.0980002	-3.351072	0.004743572	0.123521137	down	yes	Putative dis	RPP13L3
Pavir.3KG017900.v4	GO:000588	NA	leucine rich	0.678548	0.452639	7.61007	3.919095	0.0981153	-3.349379	0.000754439	0.038100161	down	yes	Receptor-lil	RLP44
Pavir.5KG186900.v4	GO:000574	NA	mitochondri	0.7280109	0.853171	14.921719	1.1683906	0.0982704	-3.347099	0.004839144	0.125342258	down	yes	Mitochondr	TOM40-1;TOM40-1
Pavir.9NG542400.v4	GO:000577	NA	ca2+:h+ ant	0.2047262	0.072267	1.758208	1.0541345	0.098492	-3.34385	0.005292249	0.131031695	down	yes	Vacuolar ca	CAX2;CAX2
Pavir.2KG562100.v4	GO:000377	NA	fused ig-ph	0.6608002	0.4984372	9.9296772	1.8149778	0.0987034	-3.340756	0.001934381	0.071255382	down	yes	Stomatal clo	SCAB1;SCAB1;SCAB1
Pavir.1NG422800.v4	GO:000563	NA	transcriptio	0.371397	0.0001	2.314849	1.438693	0.0989724	-3.33683	0.036627579	0.359804611	down	yes	Transcriptic	OFP5
Pavir.5KG039700.v4	GO:000941	00904(Dite	oxidoreduct	0.336266	0.037822	1.700499	2.076848	0.0990346	-3.335924	0.010053887	0.181875162	down	yes	Gibberellin	GA2OX3
Pavir.5KG441200.v4	GO:000557	00511(Othe	zinc finger f	1.23697	0.148799	6.187304	7.804621	0.0990406	-3.335836	0.003137968	0.096442669	down	yes	GDSL ester	At1g28600;At1g28600
Pavir.4KG202700.v4	GO:000437	00500(Starc	glycosyltran	1.223952	0.086412	7.831544	5.381332	0.0991733	-3.333905	0.004452772	0.119502871	down	yes	Soluble star	SSII-3
Pavir.3NG151700.v4	NA	NA	0	0.707887	0.056774	5.6756	2.018008	0.0993891	-3.330768	0.015331133	0.22678609	down	yes	NA	NA
Pavir.3KG355600.v4	GO:000072	NA	protein brea	1.590849	0.132818	12.715077	4.610834	0.0994849	-3.329378	0.004101444	0.114255493	down	yes	Protein BRI	BRCA1
Pavir.6KG362500.v4	GO:000485	NA	plant inverta	0.963372	0.286933	8.562436	3.999251	0.0995332	-3.328678	0.001968955	0.071764192	down	yes	Pectinestera	PMEI28
Pavir.7NG184100.v4	GO:000981	00908(Zeat	glucosyl/glu	1.41636	0.0001	7.25194	6.922094	0.0999334	-3.322889	0.040689108	0.379923424	down	yes	7-deoxyloga	UGT85A24
Pavir.4KG314400.v4	GO:000374	NA	eukaryotic t	1.359577	0.375686	10.866484	6.484507	0.1000094	-3.321792	0.000373163	0.022687448	down	yes	Protein argo	PHN1
Pavir.3NG049600.v4	GO:003421	01230(Bios	solute carrie	1.7669251	0.7211695	17.63094	7.2474673	0.1000102	-3.321781	0.000293241	0.018963739	down	yes	Triose phos	TPT;TPT
Pavir.6KG335300.v4	GO:000367	03013(RNA	thaumatin fa	0.653087	0.0001	1.999521	4.529992	0.1000361	-3.321407	0.041911311	0.385680547	down	yes	Pathogenesi	-
Pavir.1KG115700.v4	GO:000550	00053(Asco	ascorbate ox	0.80103	0.229309	8.805636	1.469031	0.1002796	-3.317901	0.0095514	0.178730357	down	yes	L-ascorbate	AAO
Pavir.1KG076000.v4	GO:000815	NA	haloacid del	0.5595352	0.0248306	2.858528	2.9667259	0.1003159	-3.317377	0.01281122	0.205647752	down	yes	NA	NA
Pavir.3KG414200.v4	GO:001616	00591(Linc	lipoxygenas	1.499927	0.449411	6.763779	12.645015	0.1004358	-3.315654	0.000361343	0.022111985	down	yes	Probable lir	LOX1.5
Pavir.3KG528600.v4	NA	NA	0	0.834188	0.944686	15.159328	2.53813	0.1005158	-3.314506	0.002013944	0.07262055	down	yes	F-box/LRR	FBL17
Pavir.2KG071300.v4	GO:000550	NA	c2 calcium/l	0.305642	0.185931	3.778407	1.109631	0.1005665	-3.313778	0.00324061	0.098431225	down	yes	Protein QU	QKY
Pavir.1KG515900.v4	NA	NA	0	5.623877	0.171663	36.674622	20.827724	0.1007879	-3.310606	0.009053874	0.173827902	down	yes	Protein CDI	CDI
Pavir.5KG436700.v4	GO:000367	04075(Plan	auxin respo	0.6233626	0.3220289	7.058957	2.3086303	0.1009216	-3.308694	0.000814383	0.04026158	down	yes	Auxin respo	ARF2;ARF2;ARF2
Pavir.5KG263200.v4	GO:000460	00940(Phen	peroxidase (	4.511341	0.351816	38.496784	9.680861	0.1009422	-3.308399	0.005618803	0.13507056	down	yes	Peroxidase	PER1
Pavir.9KG318200.v4	GO:000436	00480(Glut	glutathione	1.448363	0.0001	3.284585	10.991035	0.1014641	-3.300959	0.046742978	0.406827407	down	yes	Glutathione	GSTF12
Pavir.5NG325000.v4	GO:000367	NA	solute carrie	0.468558	0.91092	6.060161	7.469906	0.1019565	-3.293975	0.000292046	0.018948513	down	yes	NA	NA
Pavir.1NG132900.v4	GO:000524	04626(Plan	cyclic nucle	2.5409768	0.0277325	9.6817388	15.509435	0.1019686	-3.293803	0.018388725	0.247552699	down	yes	Potassium c	Os02g0245800;Os02g0245800;Os02g0245800;Os02g0245800
Pavir.5NG066000.v4	NA	NA	0	0.293958	0.245454	3.8955297	1.3876709	0.1020995	-3.291953	0.004217324	0.116171203	down	yes	NA	NA
Pavir.5NG014100.v4	GO:000367	NA	nuclear tran	0.231774	0.065148	1.273428	1.628574	0.1023163	-3.288893	0.006522767	0.14647695	down	yes	NA	NA
Pavir.1NG431100.v4	GO:003059	NA	pectinestera	0.553284	0.0001	2.787977	2.619515	0.1023365	-3.288607	0.040010983	0.377133998	down	yes	NA	NA
Pavir.1NG509800.v4	NA	NA	0	0.869066	0.343717	6.778357	5.039503	0.1026229	-3.284575	0.000867844	0.041858165	down	yes	NA	NA
Pavir.9KG381800.v4	GO:000449	00905(Bras	cytochrome	6.897015	27.797941	246.42168	91.621124	0.1026348	-3.284408	0.002036422	0.073009987	down	yes	Cytochrome	CYP724B1
Pavir.9NG847900.v4	GO:000455	04075(Plan	xyloglucan	1.473216	0.288617	11.687132	5.474477	0.1026613	-3.284036	0.00207924	0.073868681	down	yes	Probable xy	XTH28
Pavir.9KG073400.v4	GO:000467	00010(Glyc	ribosomal p	0.275782	0.0001	1.639212	1.039338	0.1029968	-3.279329	0.043350767	0.392959798	down	yes	Protein kina	PID
Pavir.9NG815300.v4	NA	NA	0	5.114255	3.291303	66.4869	15.067063	0.1030674	-3.278339	0.001387776	0.057489408	down	yes	NA	NA
Pavir.9NG685300.v4	GO:000579	NA	protein pmr	0.599495	0.0001	2.349503	3.46501	0.1031204	-3.277598	0.04337933	0.393008497	down	yes	Protein PM	PMR5
Pavir.2NG509800.v4	GO:000367	NA	ankyrin repe	0.172528	0.242946	3.021789	0.996121	0.1034055	-3.273615	0.005083845	0.128459391	down	yes	NA	NA
Pavir.4KG391200.v4	NA	NA	0	0.802096	0.183634	6.726438	2.754952	0.1039647	-3.265834	0.004965472	0.127176329	down	yes	NA	NA
Pavir.6NG310200.v4	GO:000467	00500(Starc	legume lecti	0.93342	0.0001	3.206877	5.770488	0.103986	-3.265539	0.046602612	0.406300312	down	yes	Probable L-	LECRKS5
Pavir.7NG179500.v4	GO:000403	00010(Glyc	aldose-1-ep	0.162978	0.252078	2.622046	1.355285	0.1043554	-3.260423	0.004718175	0.123365338	down	yes	NA	NA
Pavir.1KG228900.v4	GO:000456	00052(Gala	galactose bi	0.5978707	0.0096332	1.7662622	4.0338131	0.1047407	-3.255106	0.020785112	0.265001358	down	yes	Beta-galact	Os05g0428100;Os05g0428100
Pavir.3NG119000.v4	NA	NA	cysteine-ricl	0.384826	0.0001	1.890585	1.778062	0.1049232	-3.252595	0.037576153	0.364818808	down	yes	NA	NA
Pavir.8NG120600.v4	GO:000455	04075(Plan	xyloglucan	1.37921	0.033468	8.72375	4.73087	0.1049958	-3.251597	0.017612952	0.243207467	down	yes	Probable xy	XTH
Pavir.3KG143200.v4	GO:000028	NA	sucrose-pho	0.33336	0.14026	2.374727	2.133891	0.1050477	-3.250883	0.00447487	0.119839116	down	yes	Sucrose-pho	SPP2
Pavir.7KG106500.v4	NA	NA	0	20.587711	0.811745	67.457886	136.24333	0.1050532	-3.250809	0.010320697	0.184656922	down	yes	NA	NA
Pavir.6KG414000.v4	GO:000685	NA	multidrug re	0.534346	0.127734	4.225135	2.069833	0.1051761	-3.249122	0.003788086	0.108898049	down	yes	Protein DET	DTX35
Pavir.6KG391900.v4	GO:000372	03010(Ribo	large subun	16.200482	11.291635	208.65527	52.581233	0.1052384	-3.248267	0.001118055	0.049655658	down	yes	60S ribosor	RPL38A;RPL38A;RPL38A
Pavir.9NG021900.v4	GO:000465	00040(Pent	pectate lyas	7.431936	0.114301	43.740807	27.858122	0.1053959	-3.246109	0.019708164	0.25684872	down	yes	Probable po	GSVIVT00026920001
Pavir.5KG540700.v4	GO:000588	NA	leucine rich	1.24771	0.061464	3.253539	9.162985	0.105438	-3.245533	0.014601176	0.219883759	down	yes	Receptor-lil	RLP51
Pavir.2NG343600.v4	GO:000455	00500(Starc	alpha-amyla	0.156913	0.344717	2.977327	1.780192	0.1054394	-3.245514	0.004652257	0.122881305	down	yes	Alpha-amyl	AMY1.7
Pavir.7KG427900.v4	GO:000460	00940(Phen	peroxidase (	2.021838	0.061101	17.255835	2.493567	0.1054685	-3.245116	0.022204398	0.27450782	down	yes	Peroxidase	PER16
Pavir.8NG018900.v4	GO:000013	00500(Starc	alpha-1,4-g	1.2671046	0.5978992	12.375514	5.291482	0.1055643	-3.243806	0.000312797	0.020021682	down	yes	Probable ga	GAUT7;GAUT7;GAUT7;GAUT7
Pavir.9NG599600.v4	GO:000016	NA	rna recognit	1.796129	1.086406	22.896519	4.246648	0.1061974	-3.235179	0.000982517	0.045866809	down	yes	Zinc finger	Os07g0682400
Pavir.7KG265800.v4	NA	NA	0	1.857967	0.18056	12.903717	6.278283	0.1062729	-3.234154	0.004406095	0.118588963	down	yes	Probable as	At4g16563
Pavir.5NG172200.v4	GO:000400	03018(RNA	zinc finger c	0.6102789	0.0882117	5.080429	1.47801	0.1065026	-3.23104	0.006491931	0.14632013	down	yes	Zinc finger	Os01g0256800;Os01g0256800;Os01g0256800;Os01g0256800
Pavir.3KG494100.v4	GO:000557	NA	thaumatin fa	2.603518	0.361764	22.166105	5.667373	0.1065365	-3.23058	0.005131693	0.129317978	down	yes	Thaumatin-	-
Pavir.5KG379500.v4	NA	NA	0	0.361941	0.0001	1.275265	2.118134	0.1066898	-3.228506	0.041349491	0.3826169	down	yes	NA	NA
Pavir.1NG035900.v4	GO:000557	00561(Glyc	zinc finger f	1.377987	0.0001	5.651547	7.261806	0.106718	-3.228125	0.046474787	0.40582121	down	yes	GDSL ester	EXL3
Pavir.1KG489900.v4	GO:000563	NA	sterol regula	0.586841	0.028804	3.474934	2.278718	0.1070007	-3.224307	0.016197118	0.23345038	down	yes	Transcriptic	BIM1
Pavir.1NG485100.v4	GO:000097	00130(Ubic	homeobox-l	0.603462	0.178247	4.615371	2.677746	0.1071845	-3.221832	0.001591167	0.062799285	down	yes	Homeobox-	ROC8
Pavir.6KG194200.v4	NA	NA	0	0.6699532	1.7729459	15.341033	7.1684959	0.1085273	-3.20387	0.002072675	0.073816683	down	yes	NA	NA
Pavir.9NG450300.v4	GO:000552	00940(Phen	rac-like gtp-	1.240163	0.276092	11.594634	2.250805	0.109513	-3.190826	0.007138921	0.155046644	down	yes	Rac-like GT	RAC2
Pavir.2NG052700.v4	GO:000218	03010(Ribo	large subun	21.70809	18.413408	304.99783	60.964265	0.1096329	-3.189247	0.002004086	0.072473063	down	yes	60S acidic r	RPP2A;RPP2A
Pavir.2NG038900.v4	GO:000455	00500(Starc	carbohydrat	0.064568	1.005231	6.537473	3.217213	0.1096703	-3.188756	0.015305522	0.226496414	down	yes	Glucan endo	At5g56590
Pavir.9NG206800.v4	GO:005121	00941(Flav	oxidoreduct	0.758054	0.0001	3.565163	3.334263	0.1098865	-3.185914	0.048853971	0.416754602	down	yes	Probable 2-	At5g05600
Pavir.2NG245100.v4	GO:000367	NA	awpm-19-li	4.698784	1.08869	19.749683	32.697838	0.1103479	-3.179869	0.000897169	0.043051485	down	yes	Membrane p	PM19L
Pavir.9KG123000.v4	NA	NA	ppr repeat (	0.183957	0.115365	1.589502	1.119529	0.1104904	-3.178007	0.005725654	0.136158237	down	yes	NA	NA
Pavir.1NG186100.v4	NA	NA	protein of u	14.002682	0.181003	96.382278	31.853264	0.1106065	-3.176492	0.02396945	0.285730068	down	yes	NA	NA
Pavir.2NG650800.v4	GO:000575	NA	10 kda heat	1.474147	1.881475	27.588097	2.733484	0.1106678	-3.175693	0.007822426	0.16095948	down	yes	10 kDa cha	CPN10
Pavir.3NG223500.v4	GO:000535	NA	predicted tr	8.1374693	0.435075	32.565673	44.751315	0.1108753	-3.17299	0.00773402	0.160458503	down	yes	Polyol trans	PLT5;PLT5
Pavir.4KG017000.v4	GO:000373	03010(Ribo	small subun	6.164408	4.556386	81.653061	14.715212	0.1112482	-3.168146	0.001177497	0.051444317	down	yes	40S ribosor	RPS20
Pavir.4KG310900.v4	GO:000550	NA	anthranilate	9.801501	0.30894	54.618752	36.237675	0.1112793	-3.167743	0.014632724	0.220258684	down	yes	FT-interacti	FTIP7
Pavir.3KG339500.v4	GO:000551	NA	iq calmodul	0.611121	0.18431	4.668857	2.478143	0.1112958	-3.167529	0.004323113	0.117726974	down	yes	NA	NA
Pavir.3KG471000.v4	GO:000013	NA	cell cycle co	0.4158064	0.0959862	2.561346	2.0302566	0.1114627	-3.165367	0.004161265	0.114964127	down	yes	ALA-intera	ALIS5;ALIS5;ALIS5;ALIS5
Pavir.5KG611700.v4	GO:000382	NA	interferon, g	0.2508561	0.0001	1.1734845	1.0772411	0.1115001	-3.164883	0.047026054	0.408628572	down	yes	Gamma-inte	GILT;GILT
Pavir.6NG038800.v4	GO:000467	NA	leucine-rich	0.572992	0.181409	5.264841	1.477373	0.1118922	-3.159819	0.01280011	0.205647752	down	yes	NA	NA
Pavir.9NG516600.v4	GO:000981	00908(Zeat	glucosyl/glu	1.563766	0.09583	10.111176	4.704061	0.1120195	-3.158178	0.009453806	0.177692771	down	yes	UDP-glycos	UGT73C5

Pavir.7KG297500.v	GO:000981	00908(Zeat	glucosyl/glu	2.373811	0.094113	12.554592	9.475867	0.1120233	-3.15813	0.009998801	0.181831401	down	yes	7-deoxylog	UGT85A24
Pavir.8KG236700.v	NA	NA	0	0.410272	0.221535	4.098375	1.539893	0.1120569	-3.157696	0.006203553	0.143261282	down	yes	NA	NA
Pavir.9NG082400.v	NA	NA	0	23.918728	15.342262	284.49521	65.203186	0.112271	-3.154943	0.000857991	0.041489451	down	yes	NA	NA
Pavir.9KG408500.v	GO:000373	03010(Ribo	small subun	4.686264	3.126464	57.61961	11.788139	0.1125628	-3.151198	0.001076416	0.04849422	down	yes	40S ribosor	RPS9C
Pavir.1KG031700.v	NA	NA	0	4.894248	0.415843	11.825133	35.349266	0.112563	-3.151196	0.005905942	0.138603358	down	yes	NA	NA
Pavir.4NG021400.v	GO:000467	04075(Plan	lrr receptor-	5.658279	0.337737	28.515207	24.724209	0.1126236	-3.150419	0.008066856	0.162969566	down	yes	LRR recept	SIK1
Pavir.2KG311100.v	NA	NA	0	0.123311	0.36951	3.209099	1.155509	0.112913	-3.146716	0.013951606	0.214485965	down	yes	Protein PLA	PRIN2
Pavir.9KG571600.v	NA	NA	kinase-relat	3.209538	0.44929	14.449271	17.921385	0.1130292	-3.145233	0.001763354	0.067192175	down	yes	NA	NA
Pavir.1NG451300.v	GO:000367	04712(Circ	dof domain,	0.073744	0.764546	6.066882	1.344107	0.1131145	-3.144145	0.019074046	0.252704212	down	yes	Dof zinc fin	DOF5.6
Pavir.1KG062000.v	GO:000552	NA	arabinose-5	8.455545	0.12908	39.713894	36.144028	0.1131672	-3.143473	0.019683837	0.256703501	down	yes	Probable ar	SETH3
Pavir.3NG130400.v	GO:000467	04626(Plan	interleukin-	1.3005128	0.9475059	16.462648	3.3345817	0.1135522	-3.138573	0.001623113	0.063526276	down	yes	Proline-rich	PERK10;At1g30570
Pavir.7KG306900.v	GO:000588	NA	protein s-ac	1.6484336	0.4241449	13.351014	4.883282	0.1136638	-3.137156	0.001594167	0.062851646	down	yes	Probable pr	PAT19;PAT19;PAT19;PAT19
Pavir.5NG294800.v	GO:000419	NA	aspartyl pro	2.922552	1.475133	29.198799	9.470063	0.1137268	-3.136356	0.001425038	0.05846665	down	yes	Aspartyl pro	APF2
Pavir.9KG429700.v	NA	NA	0	2.358502	0.121539	14.756608	7.043802	0.1137612	-3.135919	0.021030195	0.266948492	down	yes	NA	NA
Pavir.9KG161500.v	GO:000460	00940(Pher	peroxidase	3.086915	0.436218	20.36216	10.59825	0.1137948	-3.135494	0.002799854	0.089227237	down	yes	Peroxidase	PER19
Pavir.7NG186600.v	GO:000431	01212(Fatty	3-oxoacyl- (	0.360553	0.08495	2.010556	1.901479	0.1138801	-3.134412	0.009210972	0.175061118	down	yes	3-oxoacyl-[a	KAS12
Pavir.3KG096600.v	GO:000588	01200(Carb	eama-like tr	0.02734	0.419972	1.16338	2.755626	0.1141391	-3.131134	0.023918035	0.285564774	down	yes	WAT1-relat	At5g64700
Pavir.2NG360900.v	GO:000367	NA	protein of u	2.5601218	0.2188041	11.985171	12.324556	0.1143133	-3.128934	0.003937822	0.111328658	down	yes	NA	NA
Pavir.5KG620200.v	GO:000218	03010(Ribo	large subun	16.99362	10.475059	209.78153	30.146029	0.1144874	-3.126739	0.003561871	0.105338145	down	yes	60S ribosor	RPL36A
Pavir.9KG385600.v	GO:000367	NA	domain of u	0.289564	0.479971	4.2486192	2.459343	0.1147196	-3.123816	0.001207836	0.052464745	down	yes	Protein LO	LNG2;LNG2
Pavir.7NG334200.v	GO:000367	04141(Prot	gram domai	0.995006	0.171378	5.982668	4.174181	0.1148372	-3.122338	0.00700276	0.153419648	down	yes	Putative GE	At5g23370
Pavir.7NG152100.v	NA	04626(Plan	adenylate cy	2.167161	0.427385	13.057272	9.498866	0.1150262	-3.119966	0.001129165	0.049854415	down	yes	NA	NA
Pavir.9KG084000.v	GO:000367	04141(Prot	small heat-s	2.330471	0.165666	5.819458	15.857424	0.115152	-3.118388	0.00941339	0.177199055	down	yes	22.0 kDa h	HSP22.0;HSP22.0
Pavir.5KG504600.v	GO:000367	NA	alba (alba)	0.629872	0.493286	4.806214	4.887796	0.115861	-3.109533	0.003249945	0.098555441	down	yes	Uncharacte	At2g34160
Pavir.9NG383200.v	GO:000551	04626(Plan	disease resi	0.325924	0.109464	2.110669	1.641682	0.1160307	-3.107421	0.004190915	0.115698191	down	yes	Disease resi	RGA4
Pavir.9KG603700.v	GO:000456	00052(Gala	beta-galacto	4.082696	0.532986	29.129969	10.352037	0.116906	-3.09658	0.004984316	0.127225339	down	yes	Beta-galact	Os03g0165400
Pavir.6KG403300.v	NA	NA	arabidopsis	1.519455	1.647917	13.044941	13.852287	0.1177583	-3.086099	0.000657744	0.03461265	down	yes	NA	NA
Pavir.9NG572400.v	GO:000455	NA	glycosyl hyd	0.621053	0.118597	2.328455	3.949654	0.1178141	-3.085415	0.009890676	0.181456243	down	yes	Endo-1,4-b	XYN4
Pavir.1KG428600.v	GO:000372	03010(Ribo	small subun	1.843684	0.755284	18.787607	3.232374	0.1180277	-3.082802	0.008188629	0.164298747	down	yes	40S ribosor	RPS26
Pavir.5NG449000.v	GO:000367	NA	awpm-19-li	7.622931	0.198305	21.928114	44.17482	0.118319	-3.079246	0.01591211	0.231765709	down	yes	Membrane	PM19L
Pavir.7KG016600.v	GO:000573	00750(Vita	aldo/keto re	0.253316	0.302477	1.033278	3.6493	0.1186938	-3.074683	0.009211291	0.175061118	down	yes	Probable al	Os04g0338000
Pavir.4KG143400.v	NA	NA	0	0.225902	0.248919	2.778925	1.208616	0.1190761	-3.070044	0.008019785	0.162733919	down	yes	NA	NA
Pavir.3NG326200.v	GO:000370	NA	tesmin/tso1	1.661972	0.983845	16.893583	5.284554	0.1192984	-3.067353	0.000968567	0.045432108	down	yes	CRC domai	TSO1
Pavir.5KG148600.v	NA	NA	oxidoreduct	1.397613	0.028571	3.66413	8.289785	0.1193069	-3.067251	0.026732209	0.304458713	down	yes	NA	NA
Pavir.2NG242400.v	GO:000375	03040(Splic	peptidyl-pro	1.439532	1.885085	25.270473	2.5839046	0.1193571	-3.066644	0.008419627	0.16685161	down	yes	Peptidyl-pro	CYP19-3;CYP19-3
Pavir.8NG111800.v	GO:000152	03008(Ribo	h/aca ribonu	1.979964	2.35686	32.020443	4.236931	0.1196122	-3.063564	0.004588745	0.122035291	down	yes	Putative H/	At3g03920
Pavir.9NG652100.v	GO:000487	00902(Mon	btb/poz don	0.904216	0.093818	6.219773	2.115832	0.1197314	-3.062126	0.010872576	0.188690661	down	yes	BTB/POZ d	NPY2
Pavir.5KG104600.v	GO:000550	04626(Plan	ef-hand calc	0.614078	0.160584	4.918711	1.550907	0.1197384	-3.062042	0.008576814	0.168680678	down	yes	Probable ca	CML16
Pavir.1NG501600.v	GO:000373	03010(Ribo	small subun	15.354761	5.990308	143.34212	34.905254	0.1197497	-3.061906	0.00166514	0.064442719	down	yes	40S ribosor	RPS30A
Pavir.1KG134300.v	GO:000367	NA	uncharacteri	0.7046521	0.481028	7.8150455	2.0848243	0.1197672	-3.061695	0.005009584	0.127610234	down	yes	Kinetochore	SPC25;SPC25
Pavir.9NG386900.v	NA	NA	0	5.385214	1.173688	38.506134	16.183315	0.1199299	-3.059736	0.002791533	0.089189012	down	yes	NA	NA
Pavir.8NG080100.v	GO:000582	01200(Carb	phosphoglyc	2.881843	0.2634472	15.740566	10.463481	0.1200307	-3.058525	0.006388173	0.145417214	down	yes	Phosphogly	At3g50520;At3g50520
Pavir.9KG273500.v	NA	NA	0	0.538578	0.148112	3.43273	2.276548	0.1202762	-3.055577	0.008304552	0.165443765	down	yes	NA	NA
Pavir.8NG292100.v	GO:000904	00500(Starc	beta-d-xylos	3.620781	1.8724123	17.772112	27.822171	0.1204799	-3.053136	0.000834729	0.040678681	down	yes	Beta-D-xylo	BXL1;BXL1
Pavir.8KG073400.v	GO:000487	NA	late embryo	1.385681	0.523582	7.314572	8.530919	0.1204925	-3.052985	0.001310775	0.055571673	down	yes	NDR1/HIN	NHL1
Pavir.7NG299600.v	GO:000402	00010(Glyc	aldehyde de	1.516002	0.491279	12.041705	4.583898	0.1207343	-3.050092	0.00221032	0.076683618	down	yes	Aldehyde de	ALDH3F1
Pavir.5KG316600.v	GO:000474	00230(Puri	ribonucleos	0.375386	0.817484	8.438823	1.430719	0.1208638	-3.048546	0.004810745	0.12474404	down	yes	Ribonucleo	RNR1
Pavir.3KG326000.v	GO:000573	03013(RNA	copper trans	1.574927	0.249437	11.158897	3.934629	0.1208706	-3.048464	0.01821774	0.246847474	down	yes	Protein SO	NAKR1
Pavir.7KG337900.v	GO:000980	00940(Pher	cinnamyl-al	1.85576	0.538716	13.286971	6.512618	0.1209356	-3.047689	0.002049778	0.07334886	down	yes	Cinnamyl a	CAD7;CAD5
Pavir.2NG393000.v	GO:000039	NA	nucleic acid	0.360843	0.392378	4.860272	1.356048	0.1211683	-3.044916	0.005370633	0.132235113	down	yes	RNA-bindi	RBP1
Pavir.9NG671900.v	GO:000456	00052(Gala	beta-galacto	2.293932	1.900888	22.58136	11.89507	0.1216721	-3.03893	0.000290314	0.018905043	down	yes	Beta-galact	Os03g0255100
Pavir.9NG553000.v	GO:000373	03010(Ribo	small subun	2.0806854	2.3716351	31.010207	5.5538128	0.1217678	-3.037795	0.002864262	0.090664626	down	yes	40S ribosor	RPS9C;RPS9C;RPS9C
Pavir.9NG657600.v	GO:000367	NA	domain of u	0.406952	0.142887	3.053253	1.454684	0.1219713	-3.035386	0.008043301	0.162733919	down	yes	NA	NA
Pavir.1NG060500.v	GO:000367	04075(Plan	aux/iaa fam	1.0751713	0.2474769	7.7927831	3.0488981	0.1219966	-3.035087	0.002301707	0.078298966	down	yes	Auxin respo	ARF6;ARF6;ARF6
Pavir.6KG205700.v	GO:000456	00520(Ami	beta-hexosa	13.149629	0.806457	63.974411	50.330479	0.1220953	-3.033921	0.010759975	0.188149509	down	yes	Beta-hexos	HEXO3
Pavir.6KG341400.v	GO:000372	03010(Ribo	large subun	5.158295	4.009272	61.914841	12.987885	0.122393	-3.030407	0.001425371	0.05846665	down	yes	60S ribosor	RPL38A
Pavir.3NG101300.v	GO:000451	NA	nyn domain	0.1957346	2.5265998	8.4975059	13.743022	0.1224042	-3.030275	0.007318184	0.156441785	down	yes	NA	NA
Pavir.3KG102500.v	GO:000961	NA	potato inhib	0.977172	7.934243	33.220272	39.582748	0.1224045	-3.030272	0.00399179	0.112241571	down	yes	Subtilisin-c	-
Pavir.3NG229000.v	NA	NA	adenine nuc	2.737712	0.439972	19.920237	6.022354	0.1224891	-3.029275	0.006481376	0.146261094	down	yes	NA	NA
Pavir.2NG490900.v	GO:000373	03010(Ribo	small subun	11.885171	13.725943	170.18071	38.496632	0.1227307	-3.026432	0.002471888	0.082191385	down	yes	40S ribosor	RPS15AA
Pavir.6NG196100.v	GO:000046	03010(Ribo	large subun	8.24129	5.167999	85.438011	23.727715	0.1228342	-3.025215	0.001843347	0.068984137	down	yes	60S ribosor	RPL7C
Pavir.9NG045000.v	GO:000425	04144(Endo	subtilisin-lil	4.290648	0.111406	22.798021	12.953396	0.1231295	-3.021752	0.01816181	0.246440481	down	yes	Subtilisin-li	SBT1.7
Pavir.7NG248300.v	GO:000016	01230(Bios	anthranilate	2.721967	4.119289	40.428276	14.958309	0.1235183	-3.017203	0.000623009	0.033133136	down	yes	Anthranilat	ASB1
Pavir.4NG275300.v	NA	NA	0	2.439456	0.142621	16.566658	4.322554	0.1236082	-3.016154	0.017508935	0.242738038	down	yes	NA	NA
Pavir.8KG004000.v	GO:000551	NA	protease inh	0.6526431	2.5088688	5.639416	19.876682	0.1239026	-3.012721	0.003563707	0.105338145	down	yes	Non-specifi	LTP2;LTP2
Pavir.3NG014400.v	NA	NA	phytosulfok	1.494533	3.92576	32.414394	11.300026	0.1239932	-3.011667	0.002318843	0.078562572	down	yes	Phytosulfok	PSK2
Pavir.5KG675900.v	GO:000367	NA	protein of u	0.4735	0.27991	1.602412	4.47066	0.1240575	-3.010919	0.012551121	0.203863881	down	yes	Protein DM	DMP7
Pavir.3KG339800.v	GO:000557	00040(Pent	pectinestera	0.9773376	0.4029568	7.3786379	3.7369009	0.124177	-3.00953	0.002000762	0.072473063	down	yes	Probable pe	PME53;PME53
Pavir.9NG816700.v	GO:000402	01200(Carb	s-(hydroxym	25.043801	6.2409648	143.02613	107.90554	0.1246744	-3.003762	0.0028419	0.090108622	down	yes	Alcohol deh	At1g22440;At1g22440
Pavir.9KG194000.v	NA	NA	0	1.101484	0.308258	6.237373	5.058099	0.1248059	-3.002241	0.015896998	0.231765709	down	yes	NA	NA
Pavir.5KG327000.v	GO:000372	NA	double-stra	0.379966	0.168692	3.029333	1.36447	0.1248709	-3.001491	0.007922865	0.162028857	down	yes	Double-stra	DRB5;DRB5
Pavir.9NG667600.v	GO:000551	04070(Phos	calmodulin-	46.223301	7.029852	248.44923	177.53188	0.1250128	-2.999852	0.005327134	0.13145285	down	yes	Calmodulin	CAM3
Pavir.2KG120800.v	GO:000576	00100(Ster	sterol 24-c-	20.795708	7.698705	109.57138	117.71969	0.1253653	-2.99579	0.001764931	0.067192175	down	yes	Cycloarten	Smt1-1
Pavir.9KG563700.v	GO:000563	NA	max dimeriz	0.209507	0.194274	1.030576	2.186433	0.1255144	-2.994075	0.009555288	0.178730357	down	yes	Transcriptio	BHLH70
Pavir.8NG261800.v	GO:000551	03008(Ribo	cyclin-a3-2-	1.45663	0.3170433	11.448854	2.6699344	0.125625	-2.992804	0.010609367	0.186793906	down	yes	Cyclin-A3-2	CYCA3-2;CYCA3-2
Pavir.5KG756200.v	NA	NA	classical ara	2.844467	0.599074	20.069736	7.324026	0.1257053	-2.991883	0.00474629	0.123521137	down	yes	NA	NA
Pavir.3NG108900.v	GO:000467	04626(Plan	non-specific	4.425806	0.756249	32.84288	8.374321	0.1257255	-2.99165	0.005202742	0.130321762	down	yes	Receptor-lil	ANX1
Pavir.9KG448900.v	GO:000551	NA	calmodulin	2.8341148	0.7823064	20.865356	7.8419991	0.1259754	-2.988786	0.001825832	0.068533462	down	yes	NA	NA
Pavir.1NG453300.v	GO:000078	01212(Fatty	histone h2b	13.091314	15.056739	211.02341	11.60406	0.1264357	-2.983525	0.013813141	0.213594684	down	yes	Histone H2	-
Pavir.6KG212000.v	NA	NA	0	0.194873	0.069214	1.053775	1.033052	0.1265495	-2.982226	0.00936104	0.176596389	down	yes	NA	NA
Pavir.9NG005800.v	NA	NA	0	0.281034	0.116956	1.747087	1.386976	0.1269885	-2.97723	0.013710215	0.212601703	down	yes	NA	NA
Pavir.9NG151100.v	GO:000221	00591(Linc	lipoxygenas	0.5741121	0.6330967	6.0500463	3.4252931	0.1274053	-2.972502	0.001005316	0.04629014	down	yes	Putative lin	Os03g0700400;Os03g0700400

Pavir.9NG125600.v	GO:000521	03015(mRN	retinaldehyc	4.6463896	1.1861292	32.471862	13.183389	0.1277513	-2.96859	0.002034739	0.073009987	down	yes	NA	NA
Pavir.3KG025900.v	NA	NA	phytosulfok	2.0709839	2.3492145	28.420476	6.0936494	0.1280693	-2.965004	0.003213279	0.097984464	down	yes	Phytosulfok	PSK2;PSK2
Pavir.5KG139200.v	GO:000367	00130(Ubic	homeobox-l	1.143182	0.123629	5.961706	3.927629	0.1280987	-2.964672	0.007249353	0.156360474	down	yes	Homeobox-	ROC5
Pavir.7NG079500.v	GO:000367	NA	sap30//subf	3.985498	0.2905603	18.01416	15.35024	0.1281623	-2.963956	0.008847905	0.172158188	down	yes	NA	NA
Pavir.5KG007600.v	GO:000577	NA	reticulon-lik	25.589127	4.847286	164.57907	72.696381	0.1282746	-2.962693	0.005393544	0.132538663	down	yes	Reticulon-li	RTNLB1
Pavir.1NG492300.v	GO:000563	NA	transcriptio	1.324518	1.389721	14.466765	6.658959	0.1284803	-2.960381	0.001116977	0.049655658	down	yes	Transcriptic	BHLH96
Pavir.9NG684600.v	GO:000436	00480(Glut	glutathione	6.683201	0.097897	9.141776	43.550743	0.1286919	-2.958007	0.036041192	0.356925592	down	yes	Glutathione	GSTF13
Pavir.9KG059900.v	NA	NA	actinidain /	0.928284	0.456748	7.222586	3.534713	0.1287528	-2.957325	0.006794329	0.150432125	down	yes	NA	NA
Pavir.6NG311500.v	GO:000008	03022(Basa	cyclin-deper	6.295763	0.130604	34.177341	15.695021	0.1288563	-2.956165	0.025002629	0.293294965	down	yes	Cyclin-depe	CDKB2-1
Pavir.5NG518700.v	GO:000685	NA	mate efflux	1.243887	0.630008	12.710082	1.828906	0.1288876	-2.955815	0.007090445	0.154753199	down	yes	Protein DE	DTX19
Pavir.8NG066000.v	GO:000446	01212(Fatty	long-chain a	1.283151	0.677333	10.930968	4.266139	0.1290038	-2.954515	0.001246928	0.053563962	down	yes	Long chain	LACS9
Pavir.2KG051900.v	NA	NA	0	4.205173	3.836481	49.729435	12.556528	0.1291086	-2.953343	0.002782214	0.088966929	down	yes	NA	NA
Pavir.2KG514000.v	NA	NA	effector of t	0.697678	0.098807	4.726372	1.43878	0.1291915	-2.952417	0.020485072	0.262666409	down	yes	Protein EFF	ET
Pavir.9NG779000.v	GO:000078	NA	histone h4 (	2.427189	1.123494	25.453598	2.026398	0.1292097	-2.952213	0.010657461	0.187205603	down	yes	Histone H4	-
Pavir.9KG355800.v	NA	NA	0	1.7789998	0.277907	10.678902	5.195683	0.1295723	-2.948171	0.010978102	0.189471856	down	yes	NA	NA
Pavir.7NG022900.v	GO:000981	NA	sterol 3-beta	0.9362784	0.8586172	4.4917216	9.329528	0.1298649	-2.944916	0.002647809	0.085912042	down	yes	Sterol 3-bet	UGT80A2;UGT80A2;UGT80A2;UGT80A2
Pavir.9KG077500.v	NA	NA	actinidain /	0.438173	0.511763	4.796931	2.486673	0.1304211	-2.93875	0.008775059	0.171186547	down	yes	NA	NA
Pavir.4NG035600.v	GO:000367	NA	phosphate-i	0.599881	0.137822	4.5674	1.082218	0.1305757	-2.937041	0.017247485	0.241265726	down	yes	Protein EX	EXL3
Pavir.7NG372700.v	GO:000001	NA	aspergillus	1.74743	0.5182722	12.105246	5.246364	0.1305759	-2.937039	0.003051108	0.094586855	down	yes	Endonuclea	ENDO2;ENDO2;ENDO2
Pavir.5NG529300.v	NA	NA	0	0.445579	0.289595	3.913557	1.711073	0.1307062	-2.935601	0.010121074	0.182213283	down	yes	NA	NA
Pavir.5NG556500.v	GO:000002	03010(Ribc	large subun	26.293771	16.440054	256.09625	70.621457	0.1307974	-2.934594	0.003155001	0.096836422	down	yes	60S ribosor	RPL24;RPL24
Pavir.6NG346700.v	GO:000563	NA	hras-like su	0.675664	0.323392	4.057631	3.567357	0.1310239	-2.932098	0.004657487	0.122881305	down	yes	NA	NA
Pavir.2NG334000.v	NA	NA	ggl domain	1.492602	0.266328	11.261043	2.15363	0.1311199	-2.931042	0.015004457	0.224070926	down	yes	NA	NA
Pavir.8NG069300.v	GO:000969	00970(Ami	nucleoporin	3.188194	2.079939	37.473927	2.654017	0.1312834	-2.929244	0.011042889	0.190066184	down	yes	Dirigent prc	DIR21
Pavir.9KG607500.v	GO:000013	NA	beta-manna	0.2966377	0.2462307	3.1122168	1.0208687	0.131347	-2.928545	0.007075692	0.154566855	down	yes	Probable gl	CSLA4;CSLA4
Pavir.5NG149100.v	GO:000588	NA	leucine-rich	2.080711	0.28918	11.448691	6.591283	0.1313689	-2.928305	0.005321103	0.131446292	down	yes	Piriformosp	PII-2
Pavir.9NG062800.v	GO:000373	03010(Ribc	small subun	4.937038	3.485479	51.185707	12.879609	0.1314677	-2.92722	0.001571054	0.06226711	down	yes	40S ribosor	RPS2D
Pavir.7KG028200.v	GO:000551	00053(Ascc	l-ascorbate	1.650417	0.87843	3.819964	15.413283	0.1314831	-2.927051	0.003747732	0.10823485	down	yes	Probable L-	APX3
Pavir.9KG600500.v	GO:000079	04070(Phos	histone h2a	15.719922	0.787974	112.33255	13.206428	0.1314962	-2.926907	0.028400369	0.313112402	down	yes	Probable hi	Os03g0162200
Pavir.2NG037500.v	GO:000467	04626(Plan	leucine-rich	5.057941	0.394626	27.618288	13.819887	0.1315832	-2.925953	0.012174442	0.200100754	down	yes	LRR recept	GHR1;GHR1;GHR1
Pavir.6KG055800.v	GO:000373	03010(Ribc	predicted m	1.221636	0.42027	9.997037	2.479756	0.1315968	-2.925804	0.016634523	0.236835519	down	yes	NA	NA
Pavir.1NG156400.v	GO:000950	01212(Fatty	3-oxoacyl- (	2.217414	0.142326	12.839649	5.020614	0.1321224	-2.920054	0.021179864	0.267787733	down	yes	NADPH-de	At3g03980
Pavir.1NG553300.v	GO:000049	03008(Ribc	rrna 2'-o-me	5.9170566	7.3848552	89.158847	11.420897	0.1322524	-2.918634	0.006548421	0.146723658	down	yes	rRNA 2'-O-	FIB1;FIB1
Pavir.4KG309800.v	GO:000563	04075(Plan	transcriptio	7.7029698	0.3443437	37.736921	23.106649	0.1322623	-2.918526	0.015281223	0.226404334	down	yes	Transcriptic	BHLH48;BHLH48
Pavir.7NG171200.v	NA	NA	leucine-rich	0.340445	0.101502	2.206136	1.129281	0.1325013	-2.915922	0.01854621	0.248603904	down	yes	NA	NA
Pavir.2NG161000.v	GO:000046	03010(Ribc	small subun	6.384767	5.124001	72.923782	13.920983	0.1325211	-2.915705	0.00258144	0.084783622	down	yes	40S ribosor	RPS24A
Pavir.5KG146500.v	GO:000097	NA	achaete-scut	0.541936	0.324558	5.106843	1.422898	0.1326996	-2.913764	0.013261778	0.209130801	down	yes	Transcriptic	BHLH162
Pavir.5KG151600.v	GO:000024	00100(Stero	cholestenol	4.965682	2.499585	42.98584	13.195146	0.1328789	-2.911816	0.00157511	0.062296446	down	yes	Probable 3-	Os01g0103600
Pavir.1NG010000.v	GO:000456	00052(Gala	beta-galacto	2.90394	0.459963	8.01763	17.177256	0.1335153	-2.904923	0.004268092	0.116969369	down	yes	Beta-galact	OsI_006270
Pavir.2NG573700.v	GO:000588	NA	zinc finger c	1.541975	0.114267	8.021918	4.37897	0.1335583	-2.904458	0.013351285	0.209368875	down	yes	Probable pr	PAT22
Pavir.1NG514000.v	GO:000557	NA	integral mer	0.8203051	2.29441	12.95922	10.340928	0.1336779	-2.903167	0.000937986	0.044498669	down	yes	NA	NA
Pavir.3NG273900.v	NA	NA	downstream	0.419153	0.947798	8.903219	1.31804	0.1337361	-2.902539	0.010003074	0.181831401	down	yes	NA	NA
Pavir.8KG273800.v	NA	NA	0	2.679565	0.643699	15.053321	9.654328	0.1345034	-2.894285	0.005703533	0.136102468	down	yes	NA	NA
Pavir.5NG503400.v	GO:000557	NA	protein of u	34.16003	1.738727	164.18263	102.12989	0.1347994	-2.891114	0.019561678	0.256267806	down	yes	NA	NA
Pavir.5KG741100.v	GO:000367	NA	f1c9.28 pro	0.61619	0.033447	1.95282	2.864345	0.1348588	-2.890479	0.032210708	0.335901453	down	yes	NA	NA
Pavir.7NG089900.v	GO:000550	NA	plant phosp	1.481229	1.137925	13.158924	6.261534	0.1348657	-2.890404	0.001512813	0.060792428	down	yes	FT-interacti	FTIP3
Pavir.9KG570600.v	GO:000078	NA	histone h4 (	15.273749	12.687016	191.17334	16.116539	0.1348873	-2.890174	0.008738412	0.170737565	down	yes	Histone H4	-
Pavir.3NG019000.v	GO:000551	03030(DNA	replication f	0.774745	0.784025	8.947015	2.601386	0.1349771	-2.889213	0.00735919	0.156828349	down	yes	Replication	RPA2A
Pavir.8KG002900.v	GO:000013	00500(Starc	alpha-1,4-g	3.9797909	0.6381903	21.675454	12.480314	0.1352036	-2.886795	0.003742764	0.10823485	down	yes	Probable ga	GAUT7;GAUT7;GAUT7;GAUT7;GAUT7
Pavir.9NG027500.v	GO:000635	NA	rho-interacti	0.872436	0.195251	5.47374	2.39538	0.1356806	-2.881714	0.008620311	0.169310473	down	yes	NA	NA
Pavir.2KG491900.v	NA	NA	0	0.132841	0.384897	2.642093	1.152754	0.1364319	-2.873748	0.021409475	0.269385754	down	yes	NA	NA
Pavir.9KG466900.v	GO:000563	NA	t14p4.7 pro	5.858429	0.17613	26.972775	17.208334	0.1365869	-2.872109	0.023281235	0.281238111	down	yes	NA	NA
Pavir.9NG741700.v	NA	NA	small heat-s	2.97616	0.206958	5.828129	17.47448	0.1365992	-2.871979	0.020340494	0.261527703	down	yes	NA	NA
Pavir.8KG045500.v	GO:000367	04075(Plan	b3 dna bind	3.5202459	0.5376963	16.466554	13.226251	0.1366642	-2.871293	0.004289692	0.117219331	down	yes	B3 domain-	Os11g0156000;Os11g0156000
Pavir.8NG098100.v	GO:000078	NA	histone h4 (	4.008902	6.615111	74.816925	2.434379	0.1375254	-2.86223	0.025201459	0.294432348	down	yes	Histone H4	-
Pavir.2NG401300.v	GO:000372	03010(Ribc	large subun	15.840387	17.679808	199.05827	44.508186	0.1376224	-2.861213	0.002190495	0.07621234	down	yes	60S ribosor	RPL37C
Pavir.3NG262100.v	GO:000399	03015(mRN	acid phosph	3.082136	0.461041	16.467096	9.277496	0.137628	-2.861154	0.005252564	0.1307627	down	yes	Purple acid	PAP2
Pavir.2NG424200.v	GO:000372	NA	double-strar	0.696914	0.25536	5.014418	1.900717	0.1377087	-2.860309	0.010152577	0.182343582	down	yes	Double-stra	DRB5
Pavir.5NG160300.v	GO:000002	03010(Ribc	small subun	10.663975	9.865591	120.29341	28.289841	0.1381688	-2.855496	0.003548863	0.105241853	down	yes	40S ribosor	RPS5
Pavir.5NG017300.v	GO:000577	03013(RNA	reticulon-lik	8.5272389	2.4386804	52.409994	26.914276	0.1382417	-2.854736	0.003461642	0.103062034	down	yes	Reticulon-li	RTNLB1;RTNLB1
Pavir.4KG090100.v	GO:000456	00531(Glyc	heparanase-	19.679363	0.359393	73.213638	71.572182	0.1384028	-2.853055	0.036364653	0.358334415	down	yes	Heparanase	At5g34940
Pavir.5KG613700.v	GO:000367	NA	domain of u	1.228474	0.412064	5.718353	6.121183	0.1385644	-2.851372	0.010575595	0.186577829	down	yes	NA	NA
Pavir.1KG192800.v	GO:000097	NA	transcriptio	0.784748	0.9675132	4.092834	8.5264321	0.138856	-2.848338	0.002956881	0.092582416	down	yes	Protein CY	CGA1;CGA1;CGA1
Pavir.9NG735000.v	GO:000456	00052(Gala	beta-galacto	8.5810775	1.1046745	51.662029	18.059615	0.1389203	-2.847671	0.011565614	0.19504256	down	yes	Beta-galact	Os03g0165400;Os03g0165400
Pavir.5NG078300.v	NA	NA	0	1.328502	0.045968	5.329337	4.564313	0.1389245	-2.847627	0.035380308	0.354053501	down	yes	NA	NA
Pavir.5NG564300.v	GO:000467	04075(Plan	leucine rich	3.034759	1.667002	20.517267	13.320246	0.1389511	-2.84735	0.001016016	0.046701331	down	yes	Probable LI	IRK
Pavir.1NG041000.v	NA	NA	0	4.267547	1.249498	10.546269	29.134855	0.1390345	-2.846485	0.002913355	0.09167795	down	yes	NA	NA
Pavir.6NG105500.v	GO:000561	01212(Fatty	3-hydroxyac	1.256143	0.164334	8.112594	2.083675	0.1393134	-2.843594	0.028315899	0.312780873	down	yes	NA	NA
Pavir.7KG176900.v	GO:001616	00591(Linc	lipoxygenas	0.847497	0.06944	3.40688	3.174237	0.1393285	-2.843438	0.03098673	0.329353436	down	yes	Putative lip	Os04g0447100
Pavir.8NG017900.v	GO:000046	03010(Ribc	small subun	30.236511	26.072372	323.04739	79.589119	0.1398504	-2.838044	0.004741834	0.123521137	down	yes	40S ribosor	RPS16A
Pavir.1KG469800.v	GO:000573	00053(Ascc	disulfide ox	0.8126697	0.951444	2.4488641	10.162852	0.139879	-2.837749	0.004204662	0.115907386	down	yes	Monodehyd	MDAR1;MDAR1
Pavir.5NG379700.v	NA	NA	small heat-s	3.175571	0.083812	5.023608	18.236013	0.1401305	-2.835157	0.039583545	0.375463894	down	yes	NA	NA
Pavir.9KG002500.v	GO:000508	NA	prone (plant	0.569499	0.671946	5.760077	3.087886	0.1403086	-2.833325	0.002917102	0.091719073	down	yes	Rop guanin	ROPGEF7
Pavir.9NG516700.v	GO:000981	00906(Carc	flavonol 3-c	3.980144	0.668323	24.759989	8.366144	0.1403263	-2.833143	0.006548734	0.146723658	down	yes	UDP-glucos	GT7
Pavir.5NG622500.v	NA	NA	classical ara	21.643873	1.602944	113.67204	51.751163	0.1405294	-2.831057	0.016394719	0.235402849	down	yes	NA	NA
Pavir.2NG460100.v	GO:000576	00561(Glyc	gdp-fucose	0.447368	0.224878	3.522236	1.260798	0.140548	-2.830865	0.011632846	0.195737083	down	yes	O-fucosyltra	OFUT16
Pavir.2NG350700.v	GO:000969	00270(Cyst	1-aminocycl	3.701655	0.977804	24.364826	8.858798	0.1408473	-2.827796	0.003943311	0.111400005	down	yes	1-aminocyc	ACO1
Pavir.9NG758100.v	NA	NA	kinase-relat	6.133236	0.338734	19.659143	26.276903	0.1408909	-2.82735	0.01822303	0.246847474	down	yes	NA	NA
Pavir.2NG401400.v	NA	NA	0	5.378725	4.416981	57.262119	12.204647	0.1410128	-2.826102	0.010382447	0.185224526	down	yes	NA	NA
Pavir.7KG192500.v	GO:000016	01230(Bios	anthranilate	2.729499	2.445337	28.854046	7.79506	0.1411995	-2.824193	0.002671381	0.086378469	down	yes	Anthranilat	ASB1
Pavir.6NG206700.v	GO:000373	03010(Ribc	large subun	1.1684558	0.6432296	9.9447493	2.8406678	0.1416994	-2.819095	0.011883715	0.197394707	down	yes	50S ribosor	RPL27;RPL27
Pavir.7NG020400.v	GO:000399	NA	inactive pur	0.3641488	0.3000747	2.8192591	1.8639071	0.1418321	-2.817744	0.005682398	0.13593549	down	yes	Probable in	PAP27;PAP27

Pavir.5NG061100.v4	NA	NA	cramped pro	0.392736	0.321647	3.778398	1.256179	0.1418953	-2.817101	0.010075686	0.182016394	down	yes	TSL-kinase	TKI1
Pavir.9KG317200.v4	GO:000465	00330(Argi	proline dehy	2.826317	1.109204	7.172356	20.462973	0.1424091	-2.811887	0.011109441	0.190687546	down	yes	Proline deh	POX2
Pavir.2KG547500.v4	GO:000636	03010(Ribo	small subun	22.601568	16.902079	216.17883	60.704224	0.1426727	-2.809219	0.00457473	0.121991708	down	yes	40S ribosor	rps6
Pavir.3KG000800.v4	GO:000072	NA	cell division	0.728696	0.06305	4.289353	1.244447	0.1430746	-2.805161	0.025445703	0.295794776	down	yes	NA	NA
Pavir.2KG355800.v4	GO:000367	NA	protein of u	4.4187516	0.0480673	17.967034	13.24233	0.1431243	-2.804659	0.042660582	0.389038382	down	yes	NA	NA
Pavir.5NG476400.v4	GO:000425	04144(Endo	subtilisin-lil	1.647658	0.053961	5.576689	6.304537	0.1432191	-2.803704	0.025467652	0.295794776	down	yes	Subtilisin-li	SBT5.6
Pavir.7KG242000.v4	GO:000072	NA	breast cance	1.007301	1.216074	13.063162	2.45824	0.1432458	-2.803436	0.014600227	0.219883759	down	yes	BRCA1-ass	BARD1
Pavir.3KG259400.v4	GO:000557	00040(Pent	pectinestera	0.716384	0.397847	5.699777	2.076051	0.1432942	-2.802948	0.015418328	0.22762784	down	yes	Probable pe	PME53
Pavir.5KG218900.v4	GO:000003	00062(Fatty	very-long-cl	1.210662	0.149978	5.391875	4.098692	0.1433676	-2.802209	0.010410193	0.185224526	down	yes	3-ketoacyl-C	CUT1
Pavir.7NG275300.v4	GO:000557	00040(Pent	pectinestera	0.747028	2.052351	13.780943	5.738767	0.1434129	-2.801753	0.004138736	0.114658043	down	yes	Probable pe	PME68
Pavir.1KG555800.v4	GO:000557	00500(Starc	sucrose synt	2.097406	0.29078	4.732833	11.913852	0.1434632	-2.801248	0.008324769	0.165668823	down	yes	Sucrose syn	SUS6
Pavir.2NG576200.v4	NA	NA	0	10.402447	2.307314	55.966347	32.606152	0.1434956	-2.800922	0.005939074	0.139206999	down	yes	Protein LOI	LNG2
Pavir.3NG273200.v4	NA	NA	0	0.316357	1.509074	11.126148	1.569161	0.1437878	-2.797986	0.026860696	0.30527433	down	yes	NA	NA
Pavir.3KG127700.v4	GO:000588	NA	plant protein	0.256746	0.69603	1.835396	4.783559	0.1439466	-2.796394	0.010832389	0.188515672	down	yes	UPF0481 p	At3g47200
Pavir.2NG457600.v4	GO:000436	00480(Glut	glutathione	0.662917	1.154773	7.278385	5.320207	0.1442772	-2.793084	0.001702871	0.065488211	down	yes	Glutathione	GSTF12
Pavir.6KG417600.v4	GO:000573	00330(Argi	polyamine c	61.199701	42.786555	451.81327	268.92253	0.1442779	-2.793078	0.002882536	0.090860354	down	yes	Polyamine c	MPAO1;MPAO1;MPAO1
Pavir.7NG362600.v4	GO:000090	NA	f11f12.2 pro	0.7744111	0.149039	4.6376936	1.7419053	0.1447505	-2.78836	0.011014686	0.189754601	down	yes	NA	NA
Pavir.8KG107800.v4	GO:000573	NA	protein t28c	2.07726	0.03077	6.545464	8.008512	0.1448422	-2.787446	0.044382849	0.398226279	down	yes	NA	NA
Pavir.2KG264900.v4	GO:000370	00230(Purin	dna polymer	0.186298	1.1540613	8.4255987	0.8268201	0.1448658	-2.787211	0.045537289	0.402456212	down	yes	Nuclear tran	NFYB3;NA
Pavir.5NG635800.v4	NA	NA	expressed p	4.138422	0.149246	7.003832	22.575718	0.1449538	-2.786335	0.029322176	0.319970669	down	yes	NA	NA
Pavir.9KG592800.v4	GO:000573	NA	copper trans	3.313074	1.340453	18.198996	13.898523	0.1449809	-2.786065	0.002677165	0.08649108	down	yes	Heavy meta	HIPP05
Pavir.3KG290100.v4	GO:000072	NA	histone chap	0.407693	0.792392	7.176628	1.040277	0.1460507	-2.775458	0.025324228	0.295161708	down	yes	Histone cha	ASF1B
Pavir.9KG632000.v4	GO:000487	00902(Mon	btb/poz don	1.6961706	0.176677	9.6881994	3.134824	0.1460535	-2.775431	0.013921721	0.214437719	down	yes	BTB/POZ c	NPY2;NPY2;NPY2;NPY2;NPY2;NPY2
Pavir.2NG443900.v4	NA	NA	0	0.861051	0.746398	7.67577	3.329624	0.1460601	-2.775366	0.011090821	0.190629196	down	yes	NA	NA
Pavir.9KG586700.v4	GO:000588	NA	aquaporin ti	281.90009	18.024895	1094.1899	958.12695	0.1461397	-2.77458	0.021361505	0.269063303	down	yes	Probable aq	TIP1-1
Pavir.1NG176800.v4	GO:000563	NA	transcription	0.5532957	0.0287077	2.0031838	1.9774102	0.1462102	-2.773884	0.040359306	0.378880534	down	yes	Transcriptic	BHLH144;BHLH144
Pavir.6KG409000.v4	GO:000587	04144(Endo	125 kda kin	1.6990907	1.2477214	18.89055	1.258075	0.1462538	-2.773454	0.015459274	0.227874188	down	yes	Kinesin-like	KIN5C;KIN5C
Pavir.8NG299300.v4	GO:000551	04144(Endo	ap-2 comple	0.758422	1.026881	7.89956	4.288681	0.1464775	-2.771249	0.008959888	0.173155886	down	yes	AP-2 compl	ALPHAC-AD
Pavir.9NG213500.v4	GO:000449	00905(Bras	brassinoster	3.734901	0.196928	14.519241	12.296865	0.1466219	-2.769827	0.017960127	0.244868865	down	yes	Cytochrome	CYP85A1
Pavir.9NG083100.v4	GO:000552	NA	uncharacteri	0.605758	0.436678	4.451847	2.647944	0.1468263	-2.767818	0.006939349	0.152564173	down	yes	Obg-like AT	YCHF1
Pavir.9NG351000.v4	NA	NA	arginine and	0.4021679	1.4408192	7.4803177	5.0616284	0.1469459	-2.766643	0.007423907	0.157460964	down	yes	NA	NA
Pavir.9NG298700.v4	GO:000557	00062(Fatty	3-ketoacyl-c	6.993424	0.07186	20.411085	27.606671	0.147139	-2.764749	0.044991277	0.400202948	down	yes	3-ketoacyl-C	KCS12;KCS12
Pavir.6KG354800.v4	GO:000557	NA	dna-damage	10.341984	1.842809	54.953918	27.793661	0.1472526	-2.763635	0.007919136	0.162028857	down	yes	DNA dama	DRT100
Pavir.1KG421500.v4	GO:000588	NA	aquaporin ti	5.40762	0.801087	12.306269	29.777514	0.1475321	-2.7609	0.007513353	0.157709059	down	yes	Aquaporin	TIP2-1
Pavir.2NG127800.v4	GO:000077	NA	shugoshin c	0.48376	0.578225	5.712019	1.483071	0.1475986	-2.760249	0.016700031	0.237588142	down	yes	Shugoshin-	SGO1
Pavir.1NG547400.v4	GO:000820	00100(Stero	delta(24)-ste	5.449642	2.276281	36.077427	16.254118	0.1476341	-2.759902	0.003583934	0.105668933	down	yes	Delta(24)-st	DIM
Pavir.5NG645900.v4	GO:000013	00500(Starc	x-box transc	2.548024	0.621555	13.707332	7.750996	0.1477086	-2.759175	0.003763983	0.108537319	down	yes	Cellulose sy	CSLG2
Pavir.2NG412200.v4	GO:002262	03010(Ribo	large subuni	6.797293	5.683071	71.130524	13.328481	0.1477683	-2.758591	0.004382019	0.118484849	down	yes	60S ribosor	RPL32A
Pavir.5NG581900.v4	GO:000218	03010(Ribo	60s ribosom	12.489248	6.410719	100.74644	27.144163	0.1477823	-2.758455	0.003625465	0.106338496	down	yes	60S ribosor	RPL36
Pavir.5NG129000.v4	GO:000573	NA	copper trans	0.376747	0.952561	6.937574	2.046719	0.1479591	-2.75673	0.012280232	0.201047995	down	yes	Heavy meta	HIPP37
Pavir.4NG181200.v4	GO:000563	04075(Plan	transcription	4.7431775	0.1424047	18.766421	14.240603	0.1480164	-2.756171	0.027500864	0.308757967	down	yes	Transcriptic	BHLH48;BHLH48
Pavir.9NG033800.v4	GO:000557	NA	expansin-a1	5.729247	2.455349	15.959155	39.319496	0.1480607	-2.755739	0.002175152	0.07587466	down	yes	Expansin-A	EXPA7
Pavir.1NG363800.v4	GO:000373	03010(Ribo	small subun	10.426832	3.4811694	68.25278	25.288878	0.1486824	-2.749694	0.00360474	0.105978786	down	yes	40S ribosor	RPS11;RPS11
Pavir.9NG517000.v4	GO:005121	00904(Dite	gibberellin 2	0.650645	0.184214	3.486378	2.121821	0.148864	-2.747933	0.014853923	0.222176139	down	yes	NA	NA
Pavir.2KG285100.v4	GO:000578	NA	delta(8)-fatt	28.245293	4.37675	136.85782	82.257011	0.148881	-2.747768	0.01073057	0.187853135	down	yes	Delta(8)-fat	SLD2
Pavir.1NG395700.v4	GO:000557	00561(Glyc	zinc finger f	2.182365	0.571678	15.092754	3.403722	0.1488956	-2.747627	0.010921617	0.1889309	down	yes	GDSL ester	At5g55050
Pavir.9NG747200.v4	GO:000017	03018(RNA	exosome co	0.480325	0.690189	6.735062	1.124445	0.1489297	-2.747297	0.021466964	0.26972793	down	yes	Exosome cc	RRP41
Pavir.4KG134100.v4	GO:000370	04712(Circ	b-box zinc f	0.502771	0.514139	3.062273	3.759699	0.1490639	-2.745997	0.006262362	0.144060155	down	yes	Zinc finger	COL9
Pavir.4KG374600.v4	GO:000563	03008(Ribo	cyclin-a2-1-	1.943697	0.395067	14.676926	0.946666	0.1496944	-2.739908	0.033749594	0.343943783	down	yes	Cyclin-A2-	CYCA2-1
Pavir.7NG326700.v4	GO:000538	00190(Oxid	ca2+-transp	0.887303	1.291704	9.094433	5.45943	0.1497202	-2.739659	0.000997851	0.046014367	down	yes	Calcium-tra	ACA5
Pavir.4KG376500.v4	GO:000425	04144(Endo	subtilisin se	4.52284	1.919913	35.719307	7.296326	0.149777	-2.739112	0.005812407	0.137393622	down	yes	Subtilisin-li	SBT2.6
Pavir.9NG322900.v4	NA	NA	0	2.164162	0.1849996	11.090738	4.5773932	0.1499325	-2.737615	0.023159246	0.281068051	down	yes	F-box prote	At4g35930;At4g35930
Pavir.9NG048000.v4	GO:000573	NA	starch phos	1.2544782	0.8940202	9.7640108	4.5604915	0.1499876	-2.737084	0.001603624	0.063092092	down	yes	Alpha-1,4 g	-;-;-
Pavir.1NG123000.v4	GO:000046	03010(Ribo	small subun	14.305001	14.653669	158.35548	34.583939	0.150092	-2.736081	0.005814565	0.137393622	down	yes	40S ribosor	RPS24A
Pavir.5NG459100.v4	GO:000551	03008(Ribo	guanine nuc	13.666229	12.529892	132.35606	42.123024	0.150139	-2.735629	0.004018421	0.112540498	down	yes	Guanine nu	RACK1A
Pavir.6NG333300.v4	GO:000563	04075(Plan	sterol regula	0.501253	0.078056	2.472641	1.3785927	0.1504217	-2.732916	0.02980714	0.322471544	down	yes	Transcriptic	BHLH137;BHLH137
Pavir.8NG015100.v4	NA	NA	0	0.732514	0.444701	5.33894	2.481476	0.150531	-2.731868	0.018874165	0.251024069	down	yes	PLASTID T	PTAC6
Pavir.5NG204600.v4	GO:000375	03040(Splic	peptidyl-pro	0.6923637	0.8788388	7.4733581	2.9626336	0.1505561	-2.731627	0.010318562	0.184656922	down	yes	Peptidyl-pro	CYP20-3;-
Pavir.2NG391200.v4	GO:000013	NA	cell cycle co	0.2643741	1.0240462	2.9498116	5.6038784	0.1506274	-2.730944	0.012955072	0.207263269	down	yes	ALA-intera	ALIS5;ALIS5;ALIS5;ALIS5
Pavir.1KG301100.v4	GO:000367	NA	gb	1.583065	0.248379	4.565447	7.555037	0.1511032	-2.726394	0.018411982	0.247720805	down	yes	NA	NA
Pavir.5KG577800.v4	GO:000573	NA	ppr repeat fa	0.4525635	1.0370293	8.3998702	1.4451161	0.1513047	-2.724471	0.018268166	0.247080567	down	yes	Pentatricop	At1g55890;At1g55890
Pavir.5NG195300.v4	GO:000904	00500(Starc	periplasmic	2.493553	0.1099203	12.316534	4.8628783	0.1515461	-2.722171	0.027332269	0.30767167	down	yes	Beta-xylosi	Xyl1;Xyl1
Pavir.9NG560600.v4	GO:000377	NA	fused ig-ph	7.439786	0.972363	28.260811	27.169111	0.1517619	-2.720119	0.007534268	0.157883459	down	yes	Stomatal cl	SCAB1
Pavir.9NG681500.v4	GO:000013	NA	xyloglucan	0.594379	1.572751	7.318967	6.953225	0.1518428	-2.719349	0.00474276	0.123521137	down	yes	Probable xy	XXT1
Pavir.1KG547900.v4	GO:000049	03008(Ribo	rrna 2'-o-me	3.807063	4.417145	48.181465	5.946025	0.1519414	-2.718413	0.00826964	0.165110981	down	yes	rRNA 2'-O-	FIB1
Pavir.6KG086700.v4	NA	NA	0	0.8083602	0.311633	5.6787043	1.6825154	0.1521478	-2.716455	0.00836342	0.165961883	down	yes	NA	NA
Pavir.2KG529600.v4	NA	NA	0	0.461768	0.57114	5.384554	1.368906	0.152945	-2.708915	0.01554018	0.228528838	down	yes	NA	NA
Pavir.2NG620500.v4	GO:000563	04120(Ubic	seven in abs	0.911704	0.774012	8.759913	2.260218	0.152967	-2.708708	0.012698339	0.204594639	down	yes	E3 ubiquitin	DIS1
Pavir.9NG767000.v4	GO:000573	00100(Stero	24-methyler	37.789989	32.044079	308.7724	145.5793	0.1537005	-2.701807	0.003885238	0.110347156	down	yes	24-methyler	Smt2-1
Pavir.1NG001500.v4	GO:000218	03010(Ribo	60s/50s ribo	12.041651	17.004501	151.67497	37.2943	0.1537083	-2.701733	0.005999705	0.140190869	down	yes	60S ribosor	RPL9
Pavir.6KG224100.v4	GO:000521	NA	xanthine-ura	1.82404	0.840248	13.893373	3.429144	0.1538049	-2.700827	0.005793976	0.13717521	down	yes	Nucleobase	NAT2
Pavir.5KG113300.v4	GO:000467	04075(Plan	protein kina	0.868977	0.157755	5.373355	1.300801	0.153837	-2.700526	0.023141489	0.281026239	down	yes	Probable le	At1g68400
Pavir.3KG464800.v4	GO:000373	03010(Ribo	small subun	14.535747	12.800305	143.38474	34.310043	0.1538371	-2.700524	0.005683726	0.13593549	down	yes	40S ribosor	RPS29
Pavir.4NG094600.v4	NA	NA	0	0.600808	0.09674	3.219572	1.312102	0.1539272	-2.69968	0.032723318	0.339082526	down	yes	Uncharacter	At4g28100
Pavir.3KG357300.v4	GO:000455	00500(Starc	gb	10.241353	1.533984	64.527054	11.954303	0.1539635	-2.69934	0.016086494	0.232779784	down	yes	Glucan endo	At2g01630
Pavir.9NG086900.v4	GO:000582	NA	nucleoside d	1.429641	0.710788	8.874538	5.023786	0.1540063	-2.698939	0.006593702	0.147204491	down	yes	Nudix hydro	NUDT9
Pavir.5KG072200.v4	GO:000367	NA	domain of u	5.499668	0.700665	25.058144	15.14665	0.1542187	-2.69695	0.008675991	0.170048525	down	yes	NA	NA
Pavir.6NG129700.v4	GO:000455	04075(Plan	xyloglucan::	3.590587	0.403624	13.697139	12.183456	0.1543323	-2.695888	0.010879783	0.188711705	down	yes	Xyloglucan	XTH8
Pavir.4NG015700.v4	GO:000467	04075(Plan	lrr receptor-	5.5688784	1.2078115	21.032152	22.799399	0.1546076	-2.693317	0.006347853	0.144813539	down	yes	LRR recept	SIK1;SIK1
Pavir.9KG180500.v4	GO:000573	00380(Tryp	subgroup i a	0.308219	0.076309	1.45975	1.026876	0.1546385	-2.693029	0.028320351	0.312780873	down	yes	NA	NA
Pavir.5NG245600.v4	GO:000551	NA	vacuolar cat	7.651432	0.777601	24.543972	29.918674	0.1547672	-2.691828	0.014471425	0.219044633	down	yes	Vacuolar ca	CAX1a
Pavir.5NG489400.v4	GO:000641	04075(Plan	auxin-respo	0.742995	0.163435	3.70705	2.144346	0.1549083	-2.690513	0.021018453	0.266940036	down	yes	Auxin-respo	IAA6

Pavir.5NG388700.v	GO:000014	00500(Star	lyst-interact	1.058111	0.43309	3.493659	6.131765	0.1549231	-2.690375	0.002251456	0.077392148	down	yes	Callose syn	CALS7
Pavir.5KG137100.v	GO:000455	00500(Star	glucan endo	1.825124	0.859959	13.236142	4.089407	0.1549782	-2.689862	0.004295493	0.117292557	down	yes	Probable gl	A6
Pavir.3NG043800.v	GO:000425	NA	rhomboid-re	2.322966	1.848488	17.686399	9.229945	0.1549785	-2.68986	0.003534515	0.104982088	down	yes	Rhomboid-l	RBL11
Pavir.6KG299300.v	GO:000367	00052(Gala	serine/threo	1.354745	0.525378	4.156797	7.923078	0.1556409	-2.683707	0.009187685	0.1749902	down	yes	Protein inde	IDD14;IDD14
Pavir.7NG075100.v	NA	NA	0	12.856625	0.198936	52.483223	31.346121	0.1557397	-2.682791	0.045584151	0.402522519	down	yes	NA	NA
Pavir.5NG533300.v	GO:000104	NA	transcriptio	0.413984	0.3619976	2.9787201	2.0007955	0.1558347	-2.681911	0.010346788	0.184947598	down	yes	Transcriptic	BHLH123;BHLH123
Pavir.5KG391300.v	GO:000531	NA	amino-acid	1.908954	0.329534	6.623034	7.740478	0.1558455	-2.681812	0.007476458	0.157697172	down	yes	Amino-acid	BAT1
Pavir.9NG469900.v	GO:000557	NA	pollen allerg	1.776415	0.562391	11.271921	3.719758	0.1560069	-2.680318	0.009098246	0.174501469	down	yes	Expansin-B	EXPB3
Pavir.6KG188200.v	GO:000046	03010(Ribo	large subuni	14.24096	9.390891	112.70667	38.72781	0.1560533	-2.679889	0.004614187	0.122509957	down	yes	60S ribosor	RPL7C
Pavir.3NG116700.v	NA	NA	cysteine-ricl	3.060098	0.282326	6.668215	14.743238	0.1561045	-2.679416	0.032018365	0.335163785	down	yes	NA	NA
Pavir.3NG138800.v	GO:000218	03010(Ribo	large subuni	19.517818	10.80156	152.64681	41.546627	0.1561298	-2.679182	0.006072999	0.141348194	down	yes	60S ribosor	RPL29B
Pavir.2NG594500.v	GO:000636	03010(Ribo	small subun	37.274963	36.908665	377.11227	97.907043	0.1561697	-2.678813	0.00717327	0.15551388	down	yes	40S ribosor	rps6
Pavir.9KG277500.v	GO:000166	NA	extra-large g	0.6021492	0.5487636	5.8974575	1.4695674	0.1562249	-2.678304	0.016923866	0.239450344	down	yes	Extra-large	XLG3;XLG3;XLG3;XLG3
Pavir.9NG011100.v	GO:000374	03013(RNA	elongation f	8.808428	7.11529	82.680443	19.192337	0.1563098	-2.677519	0.006602652	0.14731678	down	yes	Elongation	TUFA
Pavir.2NG005100.v	NA	NA	0	0.802379	0.983823	8.921119	2.472809	0.1567679	-2.673298	0.026432812	0.302977439	down	yes	NA	NA
Pavir.3NG122500.v	NA	NA	0	16.392145	1.683184	38.896847	76.378372	0.1568015	-2.672989	0.012117935	0.199696132	down	yes	NA	NA
Pavir.8NG096500.v	GO:000373	03010(Ribo	small subun	14.850955	13.671606	141.12868	40.454945	0.1570767	-2.670459	0.005739707	0.136316308	down	yes	40S ribosor	RPS23
Pavir.9NG592900.v	GO:000015	03008(Ribo	nucleolar pr	6.46485	6.544539	70.94548	11.628508	0.1575483	-2.666134	0.009974002	0.181831401	down	yes	Probable nu	NOP5-1
Pavir.2KG548400.v	GO:000636	03010(Ribo	small subun	10.577746	8.420321	95.290352	25.290773	0.1575542	-2.66608	0.005675395	0.13593549	down	yes	40S ribosor	rps6
Pavir.4KG404900.v	GO:000497	NA	ionotropic g	0.394539	0.325925	3.257792	1.314286	0.1575791	-2.665852	0.007806253	0.160955646	down	yes	Glutamate r	GLR3.4
Pavir.2NG423900.v	GO:000367	NA	pollen ole e	1.87774	0.258355	10.688051	2.860078	0.1576672	-2.665046	0.029371725	0.320064917	down	yes	NA	NA
Pavir.6KG109000.v	GO:000561	01212(Fatty	3r -hydroxy	6.316217	3.494703	50.587978	11.540485	0.1579135	-2.662794	0.005102093	0.128744745	down	yes	NA	NA
Pavir.4KG016800.v	GO:000367	NA	phosphate-i	16.65414	3.987414	99.281059	31.272566	0.1581079	-2.661019	0.011118554	0.190756823	down	yes	Protein EX(	EXL3
Pavir.9NG762500.v	NA	NA	0	3.512105	0.154227	13.166305	10.013322	0.1581704	-2.660448	0.028120302	0.311670826	down	yes	NA	NA
Pavir.5KG091000.v	GO:000425	00240(Pyri	protein rhor	0.481662	0.190789	2.326642	1.924518	0.1581806	-2.660356	0.013648837	0.212161115	down	yes	RHOMBOI	RBL1
Pavir.3NG063300.v	GO:000573	NA	3-deoxy-8-p	14.214359	4.4620283	67.657813	50.390588	0.1582096	-2.660091	0.004919589	0.126799488	down	yes	2-dehydro-3	KDSA;KDSA
Pavir.1NG000500.v	GO:000373	03010(Ribo	ribosomal s	1.533464	0.631367	10.607573	3.072599	0.1582459	-2.65976	0.008885833	0.17245218	down	yes	NA	NA
Pavir.8NG250200.v	GO:000467	04075(Plan	protein kina	0.500867	0.3431953	3.937245	1.3964231	0.1582517	-2.659707	0.009521131	0.178511713	down	yes	Probable in	At3g03770;At3g03770;At3g03770
Pavir.9KG509000.v	NA	NA	0	1.1870842	0.3770597	8.1086387	1.7718275	0.1583067	-2.659206	0.025520861	0.296006694	down	yes	NA	NA
Pavir.5KG021600.v	NA	NA	0	0.333197	0.150855	1.753373	1.293758	0.158855	-2.654218	0.018319527	0.247418968	down	yes	NA	NA
Pavir.1NG059300.v	NA	NA	0	0.303294	0.174379	1.683953	1.322466	0.1588844	-2.653951	0.01992992	0.258492149	down	yes	NA	NA
Pavir.7KG382200.v	NA	NA	0	3.4398207	1.2466655	19.060987	10.422648	0.1589521	-2.653336	0.004093865	0.114193614	down	yes	NA	NA
Pavir.9NG448900.v	GO:000373	03010(Ribo	large subuni	10.956462	9.310453	97.718399	29.779617	0.1589587	-2.653276	0.005244988	0.1307627	down	yes	60S ribosor	RPL27C
Pavir.5KG549600.v	GO:000557	NA	protein of u	45.813179	11.865236	223.46635	139.3346	0.1589809	-2.653075	0.008235835	0.164760344	down	yes	NA	NA
Pavir.5KG120000.v	GO:000573	NA	copper trans	1.183766	0.745418	6.940375	5.189638	0.1590422	-2.652518	0.004663956	0.122881305	down	yes	Heavy meta	HIPP37
Pavir.3NG224000.v	GO:000685	NA	multidrug re	3.183157	0.535813	10.81583	12.567462	0.1590439	-2.652503	0.007461142	0.157697172	down	yes	Protein DE	DTX30
Pavir.8KG022000.v	GO:000551	NA	protease inh	19.210203	9.346194	108.40714	71.052406	0.1591244	-2.651773	0.003586322	0.105668933	down	yes	Non-specifi	LTP2
Pavir.2NG393400.v	GO:000573	NA	2-keto-3-de	1.395933	1.394654	13.152632	4.360751	0.1593403	-2.649817	0.008984434	0.173297114	down	yes	NA	NA
Pavir.6KG271700.v	GO:000573	NA	lipoate-prot	0.193454	0.610119	3.840994	1.201453	0.1593617	-2.649623	0.030765623	0.328231795	down	yes	NA	NA
Pavir.9NG798300.v	GO:000002	03010(Ribo	small subun	24.58757	18.470636	199.36092	70.755074	0.1594064	-2.649219	0.005804531	0.137338562	down	yes	40S ribosor	-
Pavir.5KG573800.v	NA	NA	0	0.962297	0.138531	4.235622	2.664004	0.1595489	-2.647929	0.040534969	0.379521655	down	yes	NA	NA
Pavir.6NG367000.v	GO:000552	04626(Plan	leucine-rich	0.453585	0.166158	2.060883	1.823051	0.1595658	-2.647777	0.015057142	0.224669567	down	yes	Putative dis	RGA4
Pavir.7KG366700.v	GO:000419	NA	aspartyl pro	9.72086	0.691533	49.068203	16.125	0.1597159	-2.64642	0.023105007	0.280855519	down	yes	Aspartic pro	nep1
Pavir.2KG353600.v	GO:000563	00943(Isofl	member of '	9.442209	0.691984	44.490337	18.939314	0.1597706	-2.645926	0.020198997	0.260534467	down	yes	Tuliposide	TCEA1
Pavir.1NG533800.v	GO:000455	00500(Star	o-glycosyl h	18.158781	1.508186	80.775879	42.154175	0.159985	-2.643991	0.022569401	0.277141434	down	yes	Glucan endo	At2g01630
Pavir.5NG559900.v	GO:000467	04075(Plan	protein kina	7.527056	1.533221	34.98143	21.553991	0.1602584	-2.641528	0.007178434	0.15551388	down	yes	Probable in	At5g58300
Pavir.4KG222200.v	NA	NA	0	1.5027742	0.2299895	6.4577822	4.3480879	0.1603539	-2.640668	0.021574832	0.270230878	down	yes	Uncharacter	At4g28100;At4g28100
Pavir.5KG701800.v	NA	NA	0	1.178288	1.419725	11.810468	4.386819	0.160398	-2.640272	0.00386017	0.110003727	down	yes	Protein PLA	PTAC10;PTAC10
Pavir.2NG169500.v	GO:000521	NA	xanthine-ura	1.687436	0.443561	9.931955	3.348404	0.1604623	-2.639694	0.010077229	0.182016394	down	yes	Nucleobase	NAT2
Pavir.2NG186000.v	GO:000013	00500(Star	glycosyl tra	0.796024	0.662411	6.940319	2.140203	0.1606114	-2.638354	0.01740592	0.24225232	down	yes	Probable ga	GAUT7
Pavir.1NG276800.v	GO:003421	NA	solute carrie	1.103087	1.463116	9.657991	6.306048	0.160749	-2.637119	0.003676877	0.107064137	down	yes	UDP-N-ace	UGNT1
Pavir.7NG268000.v	GO:000573	01210(2-O	protein root	0.25557	0.44208	2.937774	1.397311	0.1609311	-2.635485	0.013987153	0.214856628	down	yes	Protein root	RUS1
Pavir.1NG155500.v	GO:000002	03010(Ribo	60s ribosom	7.970098	9.000797	71.783401	33.519024	0.1611634	-2.633404	0.003203858	0.097868743	down	yes	60S ribosor	RPL3
Pavir.9KG654600.v	GO:000002	03010(Ribo	large subuni	21.913206	16.892174	193.745	46.808983	0.1613167	-2.632032	0.007739385	0.160481195	down	yes	60S ribosor	RPL6
Pavir.8NG020700.v	GO:000551	NA	protease inh	176.51057	21.679876	535.68665	692.6568	0.1613477	-2.631755	0.017131031	0.240690464	down	yes	Non-specifi	LTP2-A
Pavir.3KG473700.v	NA	NA	0	1.647478	0.238062	8.873643	2.809867	0.1613847	-2.631424	0.027587598	0.308994921	down	yes	NA	NA
Pavir.5NG198900.v	GO:000382	00500(Star	bcdna.gh08	4.8857197	1.7552872	16.184136	24.943798	0.1614719	-2.630645	0.004920386	0.126799488	down	yes	Alpha,alpha	TPS1;TPS1;TPS1
Pavir.9NG083300.v	GO:000578	NA	fatty acid hy	15.637514	0.949331	59.637314	42.979431	0.1616388	-2.629155	0.022865062	0.279203442	down	yes	Dihydrocera	FAH1
Pavir.9NG338400.v	GO:000373	03010(Ribo	small subun	12.594063	9.006482	107.44369	26.043631	0.1618172	-2.627563	0.007749485	0.160513448	down	yes	40S ribosor	RPS4
Pavir.9KG378000.v	GO:000373	03010(Ribo	large subuni	23.284723	21.090595	200.60496	73.466236	0.1619116	-2.626721	0.005984809	0.139941557	down	yes	60S ribosor	RPL4A;RPL4A
Pavir.6KG217800.v	GO:000419	NA	aspartyl pro	3.004417	0.512143	13.50031	8.21194	0.161962	-2.626272	0.008565956	0.168680678	down	yes	Aspartyl pro	At5g10770
Pavir.5KG415800.v	GO:000535	NA	sugar (and c	0.286382	0.26805	2.131533	1.290655	0.162011	-2.625837	0.024327467	0.287982328	down	yes	Sugar trans	MST8
Pavir.5KG549400.v	NA	NA	0	17.658609	1.60083	61.080517	57.786423	0.1620252	-2.62571	0.018232534	0.246847474	down	yes	NA	NA
Pavir.3KG323400.v	GO:000573	03013(RNA	copper trans	0.4578211	0.7905658	6.6275922	1.07604	0.1620517	-2.625474	0.030857108	0.32878257	down	yes	Protein SOI	NAKR2;NAKR2
Pavir.1NG246200.v	GO:000467	04626(Plan	non-specific	2.4766731	0.0696186	8.4636976	7.2420995	0.1621243	-2.624828	0.036548595	0.359769549	down	yes	Protein STF	SRF8;SRF8
Pavir.6NG165600.v	GO:000521	NA	xanthine-ura	0.7229171	0.0768788	3.4407912	1.490646	0.1621831	-2.624304	0.033735078	0.343943783	down	yes	Nucleobase	NAT2;NAT2;NAT2
Pavir.2KG442700.v	GO:000373	03010(Ribo	small subun	14.537703	11.542589	121.56134	39.078449	0.1623526	-2.622797	0.005284383	0.130969727	down	yes	40S ribosor	RPS15AA
Pavir.9KG645900.v	GO:000402	01200(Carb	alcohol deh	4.066623	7.100439	47.363277	21.358673	0.1624963	-2.621521	0.002741561	0.087741633	down	yes	Alcohol deh	At1g22440
Pavir.9NG478700.v	GO:000465	00330(Argi	proline deh	2.44016	1.543777	6.337991	18.177164	0.1625091	-2.621407	0.003913535	0.110808767	down	yes	Proline deh	POX2
Pavir.8KG240800.v	GO:000446	01212(Fatty	long chain a	6.610471	0.286985	21.633396	20.778095	0.1626318	-2.620319	0.027638678	0.309344068	down	yes	Long chain	LACS2
Pavir.5NG576700.v	GO:000373	03010(Ribo	small subun	32.873425	28.244799	296.53903	79.206619	0.1626585	-2.620082	0.008150566	0.164028512	down	yes	40S ribosor	RPS23
Pavir.7KG392800.v	GO:000090	NA	f11f12.2 pro	4.271863	0.392035	23.478519	5.193913	0.1626614	-2.620056	0.025333144	0.295161708	down	yes	NA	NA
Pavir.3KG264000.v	NA	NA	0	32.834724	30.194529	306.5343	80.934944	0.162669	-2.619988	0.007221136	0.156110334	down	yes	60S ribosor	RPL27C
Pavir.5KG610200.v	GO:000367	NA	domain of u	1.471538	0.51519	6.632634	5.576786	0.1627209	-2.619528	0.008761094	0.171039596	down	yes	NA	NA
Pavir.7NG352000.v	GO:000373	03010(Ribo	large subuni	14.066128	12.776214	131.36824	33.503204	0.1628077	-2.618759	0.006756903	0.149868424	down	yes	60S ribosor	RPL14A
Pavir.3KG154600.v	GO:000467	04075(Plan	protein kina	0.376895	0.097351	1.389012	1.523892	0.1628087	-2.618751	0.018387717	0.247552699	down	yes	LRR recept	RGI3
Pavir.5KG670700.v	GO:000456	00052(Gala	beta-galacto	8.3319472	0.6014431	34.905339	19.960837	0.1628215	-2.618637	0.025153757	0.294261858	down	yes	Beta-galact	Os01g0875500;Os01g0875500;Os01g0875500
Pavir.2NG051100.v	GO:000218	03010(Ribo	large subuni	28.610926	21.082338	234.51885	70.598984	0.1628658	-2.618244	0.007583838	0.158292027	down	yes	60S ribosor	SB62
Pavir.9NG643100.v	GO:000551	03030(DNA	replication p	6.98909	1.172491	35.637932	14.435933	0.1629908	-2.617137	0.01121465	0.19179247	down	yes	Replication	RPA1B;RPA1B;RPA1B
Pavir.9NG673000.v	GO:000016	04626(Plan	leucine-rich	0.370594	0.030233	1.386012	1.07229	0.1630503	-2.616611	0.040602245	0.379595456	down	yes	Disease resi	RPS5
Pavir.5NG053700.v	GO:000467	04626(Plan	non-specific	2.98008	0.12103	13.337626	5.680914	0.1630572	-2.61655	0.032972293	0.340201195	down	yes	Receptor-lil	ALE2
Pavir.2KG162300.v	GO:000016	03008(Ribo	u3 small nu	1.908755	1.325107	16.165522	3.665017	0.1630748	-2.616394	0.008737669	0.170737565	down	yes	Protein SLC	SWA1

Pavir.8KG013700.v	NA	NA	0	0.636879	1.008381	7.611849	2.448963	0.1635315	-2.612359	0.029916595	0.32320922	down	yes	NA	NA
Pavir.9KG156900.v	GO:000557	00600(Sphi	pectin acety	3.7290575	0.268267	11.211724	13.230923	0.1635389	-2.612294	0.019730937	0.257056341	down	yes	Pectin acety	PAE9;PAE9;PAE9;PAE9;PAE9
Pavir.9NG178800.v	GO:000367	04141(Prot	small heat-s	5.363808	1.395679	10.250191	31.03042	0.1637448	-2.610479	0.00727382	0.156441785	down	yes	22.0 kDa h	HSP22.0
Pavir.8KG286100.v	GO:000446	01212(Fatty	long chain a	1.1169197	0.1441771	4.3331025	3.3684434	0.1637459	-2.610469	0.031441963	0.331565785	down	yes	Long chain	LACS2;LACS2;LACS2
Pavir.4KG084900.v	GO:000372	03010(Ribo	large subun	9.095531	5.285916	68.132141	19.68449	0.1637668	-2.610285	0.017285673	0.241440373	down	yes	60S ribosor	RPL39
Pavir.7KG098900.v	GO:000016	00230(Puri	dna polymer	0.684528	0.30811	3.837694	2.215323	0.1639906	-2.608315	0.020422329	0.262334411	down	yes	DNA polym	POL2A
Pavir.1KG540700.v	GO:000373	03010(Ribo	large subun	27.918406	25.548706	259.02609	66.941772	0.1640257	-2.608006	0.008370313	0.165961883	down	yes	60S ribosor	RPL23A
Pavir.1NG458100.v	GO:000387	00051(Fruc	phosphofruc	19.693129	1.280663	78.331665	49.479351	0.1641	-2.607353	0.028437918	0.31334249	down	yes	Pyrophosph	PFP-ALPHA
Pavir.6NG078500.v	NA	NA	0	0.568422	0.496918	4.991345	1.496087	0.164216	-2.606334	0.019328946	0.254759839	down	yes	NA	NA
Pavir.9NG719600.v	GO:001678	NA	alpha/beta-l	0.883319	0.434988	5.185369	2.83255	0.1644201	-2.604541	0.012849094	0.206139626	down	yes	NA	NA
Pavir.9KG629000.v	GO:000588	NA	zinc finger c	1.4670643	0.9891576	10.438798	4.4974384	0.1644472	-2.604304	0.003984003	0.112128062	down	yes	Probable pr	PAT22;PAT22;PAT22
Pavir.9KG549000.v	GO:000373	03410(Base	adp,atp carr	2.011432	4.567779	34.167522	5.797254	0.1646252	-2.602743	0.010408942	0.185224526	down	yes	ADP,ATP c	AAC3
Pavir.5KG209600.v	GO:000425	04144(Endo	subtilisin-lil	2.70831	0.549594	13.484177	6.305622	0.1646254	-2.602741	0.007987164	0.162515989	down	yes	CO(2)-resp	CRSP
Pavir.1KG523700.v	GO:000373	03010(Ribo	small subun	14.610953	8.425899	109.2042	30.726227	0.1646308	-2.602694	0.004370138	0.118361397	down	yes	40S ribosor	RPS30A
Pavir.9NG052200.v	GO:000425	04144(Endo	subtilisin-lil	4.336164	0.201518	15.933982	11.582509	0.1649077	-2.600269	0.027184628	0.306918028	down	yes	Subtilisin-li	SBT1.7
Pavir.1KG144200.v	GO:000002	03010(Ribo	60s ribosom	15.641998	10.249434	109.287	47.45919	0.1651806	-2.597884	0.002449389	0.081878002	down	yes	60S ribosor	RPL3
Pavir.7NG403300.v	GO:000373	03010(Ribo	small subun	26.682945	18.919125	205.68135	70.354454	0.1652035	-2.597684	0.006812594	0.150570354	down	yes	40S ribosor	RPS11
Pavir.5NG639200.v	GO:000563	NA	protein of u	0.52133	0.160168	1.367466	2.751247	0.1654638	-2.595412	0.0182807	0.247161118	down	yes	Protein RO	REL2
Pavir.5KG500500.v	GO:000551	03008(Ribo	guanine nuc	7.458999	6.878195	65.781807	20.8473	0.1655009	-2.595089	0.004130597	0.11462254	down	yes	Guanine nu	RACK1A
Pavir.2NG469700.v	NA	NA	0	13.207181	12.530131	133.98816	21.476751	0.1655506	-2.594656	0.012116673	0.199696132	down	yes	NA	NA
Pavir.9NG168200.v	GO:000455	00500(Starc	glucan endo	14.522098	0.457047	51.253571	39.087105	0.1658073	-2.59242	0.042648605	0.389038382	down	yes	Glucan end	At4g34480
Pavir.6NG029900.v	GO:000484	NA	copine//sub	0.687803	0.073327	2.488448	2.099273	0.1659059	-2.591563	0.036593879	0.359804611	down	yes	E3 ubiquiti	RGLG4
Pavir.5NG074500.v	GO:000367	NA	domain of u	1.243873	0.172538	4.761564	3.774858	0.1659256	-2.591392	0.023717599	0.284202069	down	yes	NA	NA
Pavir.5KG144300.v	GO:000588	NA	leucine-rich	7.44061	0.524404	33.159473	14.826635	0.1659858	-2.590868	0.022957986	0.279883323	down	yes	Piriformosp	PII-2
Pavir.9KG579400.v	GO:000573	00100(Stero	24-methyler	60.674599	14.709867	329.06412	124.94373	0.1660422	-2.590378	0.013909252	0.21440351	down	yes	24-methyler	Smt2-1
Pavir.9NG764100.v	GO:000373	03010(Ribo	large subun	15.422287	6.761303	105.69688	27.871962	0.1660836	-2.590019	0.010398926	0.185224526	down	yes	60S ribosor	RPL23A
Pavir.7KG382500.v	GO:000165	04075(Plan	protein kina	0.977353	0.494619	4.642223	4.211435	0.1662558	-2.588523	0.00326693	0.098935401	down	yes	Phytosulfok	PSKR
Pavir.5KG097900.v	GO:000218	03010(Ribo	large subun	7.586274	5.24193	59.876274	17.184015	0.1664697	-2.586668	0.007507621	0.157709059	down	yes	60S ribosor	RPL26A
Pavir.2KG305000.v	GO:000563	NA	armadillo/b	1.733369	0.333804	7.553374	4.857895	0.1665561	-2.58592	0.017608463	0.243207467	down	yes	NA	NA
Pavir.3KG194800.v	GO:000370	NA	no apical m	0.4262357	0.3909928	2.8837368	2.022133	0.1665818	-2.585698	0.01408767	0.215619109	down	yes	NAC domai	NAC075;NAC075;NAC075;NAC075;NAC075;NAC075
Pavir.5NG253100.v	GO:000402	00940(Pher	cinnamyl-al	3.303279	3.150025	23.658039	15.080896	0.1665844	-2.585674	0.001785154	0.067614521	down	yes	Cinnamoyl-	CCR1
Pavir.2NG089700.v	GO:000372	03010(Ribo	small subun	37.537537	33.48447	334.13742	92.198105	0.1665871	-2.585651	0.008822371	0.171930996	down	yes	40S ribosor	RPS26
Pavir.1KG281100.v	GO:000218	03010(Ribo	large subun	13.205949	5.391346	84.938698	26.39052	0.1670477	-2.581668	0.005569278	0.134904319	down	yes	60S ribosor	RPL35AA
Pavir.2KG182500.v	NA	NA	myosin-13-i	2.727927	2.536889	6.275324	25.219669	0.1671636	-2.580667	0.004931268	0.126834076	down	yes	NA	NA
Pavir.4NG056900.v	GO:000552	02010(ABC	abc transpo	2.608682	1.571576	11.230618	13.775902	0.1671667	-2.58064	0.002727238	0.087741633	down	yes	ABC transp	ABCC10
Pavir.4NG099200.v	GO:000017	00600(Sphi	sphinganine	0.444639	0.819048	4.410945	3.147695	0.1671844	-2.580488	0.012806158	0.205647752	down	yes	Sphinganin	SBH1
Pavir.5NG447900.v	NA	NA	dehydrin (de	1.436972	0.343941	6.651236	4.000684	0.1671917	-2.580425	0.02158366	0.270230878	down	yes	NA	NA
Pavir.8NG019700.v	GO:000370	04075(Plan	protein scar	0.849448	0.935222	6.928441	3.740576	0.167276	-2.579698	0.003836748	0.109792942	down	yes	Protein SC	SCR
Pavir.2NG484200.v	GO:000508	NA	prone (plant	0.436645	0.236267	1.3782308	2.6418351	0.1673883	-2.578729	0.017661433	0.243207467	down	yes	Rop guanin	ROPGEF3;ROPGEF3
Pavir.5KG154600.v	GO:000002	03010(Ribo	small subun	46.988319	45.178421	425.26682	124.97279	0.1675029	-2.577742	0.008467199	0.167353015	down	yes	40S ribosor	RPS5A
Pavir.1KG042300.v	GO:000090	03010(Ribo	extensin-lik	4.947749	0.244454	22.757341	8.235655	0.1675283	-2.577524	0.033376604	0.342545523	down	yes	Leucine-ricl	LRX1
Pavir.2NG384600.v	NA	NA	0	0.513548	0.060792	2.291662	1.136139	0.1675535	-2.577306	0.037376177	0.363911658	down	yes	IRK-interac	IRKI
Pavir.4NG002900.v	NA	NA	0	1.2318756	0.3296741	6.7899973	2.5187557	0.1677507	-2.575609	0.01163982	0.195766764	down	yes	BAG family	BAG7;BAG7
Pavir.2NG488900.v	GO:000373	03010(Ribo	small subun	16.274754	12.136984	130.47415	38.890602	0.1677547	-2.575575	0.004141031	0.114658043	down	yes	40S ribosor	RPS15AA
Pavir.4KG050400.v	GO:000437	00500(Starc	starch synth	3.260397	3.64611	27.43541	13.724004	0.167799	-2.575194	0.002003643	0.072473063	down	yes	Soluble star	SS1
Pavir.2NG306700.v	GO:000557	00073(Cuti	fatty acid or	1.338599	0.814477	10.033675	2.792455	0.1678664	-2.574615	0.008457238	0.167244116	down	yes	Protein HO	HTH
Pavir.9NG436900.v	GO:000028	00190(Oxic	fl1o4.12	33.297035	1.940237	108.06722	101.73282	0.1679565	-2.573841	0.031401477	0.331486598	down	yes	Soluble ino	IPP
Pavir.1NG056300.v	GO:000013	NA	beta-manna	22.056993	1.075117	111.05366	26.659616	0.167973	-2.573699	0.048469934	0.415032094	down	yes	Glucomann	CSLA1
Pavir.8KG285200.v	GO:000152	03008(Ribo	nucleolar pr	4.345493	2.551151	37.295586	3.725075	0.1681261	-2.572384	0.025566936	0.296124071	down	yes	Putative H/	At3g03920
Pavir.9KG630500.v	GO:000373	NA	large subun	1.0475164	1.793519	13.941962	2.9089469	0.1685983	-2.568338	0.023438526	0.281789387	down	yes	NA	NA
Pavir.1KG227100.v	GO:000883	01230(Bios	4-hydroxy-t	1.5014793	1.7978545	16.335978	3.2279287	0.1686439	-2.567948	0.011545899	0.194885026	down	yes	Probable 4-	DAPB1;DAPB1
Pavir.5NG636300.v	GO:000467	04075(Plan	protein kina	7.335737	1.698252	35.367882	18.172384	0.1687326	-2.567189	0.010804902	0.188267443	down	yes	Probably in	At3g28040
Pavir.6NG243500.v	GO:000367	00052(Gala	serine/threo	0.794136	0.233758	2.710913	3.374116	0.1689218	-2.565573	0.015482877	0.228132609	down	yes	Protein inde	IDD14
Pavir.1NG028200.v	GO:001068	00100(Stero	cycloartenol	1.6200606	1.4936678	14.382906	4.0422178	0.1689936	-2.564959	0.004516371	0.120606674	down	yes	Cycloartenc	Os02g0139700;Os02g0139700;Os02g0139700
Pavir.9KG324700.v	GO:000508	NA	rop guanine	1.277122	0.080803	4.025132	4.0003264	0.1692022	-2.56318	0.033835671	0.344372661	down	yes	Rop guanin	ROPGEF3;ROPGEF3
Pavir.4NG293600.v	GO:000002	03010(Ribo	small subun	11.332209	10.123041	98.687892	27.903239	0.1694846	-2.560774	0.007254464	0.156380932	down	yes	40S ribosor	-;-
Pavir.1KG072000.v	GO:000373	03010(Ribo	large subun	19.290583	14.989029	162.84692	39.142933	0.1697096	-2.55886	0.008731942	0.170737565	down	yes	60S ribosor	RPL27AC
Pavir.3KG435300.v	GO:000535	NA	predicted tr	19.653761	0.672305	68.929276	50.601501	0.1700488	-2.555979	0.044897296	0.39955616	down	yes	Putative pol	PLT1
Pavir.3KG001200.v	GO:000046	03010(Ribo	small subun	25.975311	17.501009	196.15637	59.142078	0.1702961	-2.553883	0.008032243	0.162733919	down	yes	40S ribosor	RPS16A
Pavir.3KG381000.v	GO:000218	03010(Ribo	60s ribosom	22.516811	8.453071	138.84602	42.844177	0.1704543	-2.552543	0.007555626	0.157976512	down	yes	60S ribosor	RPL29A
Pavir.7NG165200.v	NA	NA	arabidopsis	1.202039	0.785994	7.15007	4.492298	0.1707585	-2.549971	0.012984451	0.20735682	down	yes	NA	NA
Pavir.1KG344200.v	GO:000373	03010(Ribo	small subun	16.747238	15.7925	147.3477	43.128559	0.1708336	-2.549337	0.007301161	0.156441785	down	yes	40S ribosor	RPS12
Pavir.5NG570700.v	NA	NA	integral mer	1.254932	0.105746	4.873333	3.080851	0.1710644	-2.547388	0.039165581	0.373071272	down	yes	NA	NA
Pavir.9KG102400.v	GO:000373	03010(Ribo	large subun	0.848918	0.729675	7.886842	1.337577	0.171132	-2.546819	0.029338372	0.319979875	down	yes	NA	NA
Pavir.5KG189200.v	GO:000367	NA	igr protein r	2.23585	2.793646	23.969971	5.414002	0.1711646	-2.546544	0.01634323	0.235093483	down	yes	NA	NA
Pavir.3KG493500.v	GO:000218	03010(Ribo	large subun	46.407078	40.035835	375.7912	128.9131	0.1712744	-2.545619	0.008190231	0.164298747	down	yes	60S ribosor	RPL8
Pavir.1NG324900.v	GO:000002	03010(Ribo	ribosomal p	27.875519	19.938179	214.17915	64.96151	0.1712889	-2.545496	0.008508617	0.167906653	down	yes	60S ribosor	RPL6C
Pavir.5KG198200.v	GO:000218	03010(Ribo	large subun	6.2820921	4.8850976	51.82691	13.352926	0.1713289	-2.54516	0.005616882	0.13507056	down	yes	60S ribosor	RPL29A;RPL29A;RPL29A
Pavir.9KG230000.v	GO:000002	03010(Ribo	large subun	103.73521	84.01741	858.76721	236.87573	0.171363	-2.544873	0.011261763	0.192178627	down	yes	60S ribosor	RPL5A
Pavir.5KG364800.v	GO:000551	NA	speckle-type	2.7226	4.671035	32.930533	10.185592	0.1714819	-2.543872	0.005037341	0.128230354	down	yes	BTB/POZ a	BPM1;BPM1
Pavir.1NG469800.v	NA	NA	0	2.813403	0.760521	14.050455	6.761584	0.1717239	-2.541837	0.006397539	0.145417262	down	yes	NA	NA
Pavir.5KG639200.v	GO:000373	03010(Ribo	small subun	11.976798	12.062396	106.79253	33.178375	0.1717442	-2.541667	0.006984886	0.153206733	down	yes	40S ribosor	RPS23
Pavir.7KG117700.v	GO:000950	NA	2-pyrone-4,	0.708016	0.3642373	3.5356805	2.6964458	0.1720526	-2.539079	0.009784951	0.180534287	down	yes	NA	NA
Pavir.6NG324100.v	GO:000373	03010(Ribo	small subun	19.580305	16.84478	171.3246	40.374237	0.1720609	-2.539009	0.009973418	0.181831401	down	yes	40S ribosor	RPS25B
Pavir.5NG495200.v	GO:000367	NA	phd zn-fing	3.0029856	3.3071369	20.227743	16.43733	0.1721017	-2.538666	0.00386168	0.110003727	down	yes	Homeobox	HAZ1;HAZ1;HAZ1
Pavir.7KG345300.v	NA	NA	0	0.777042	0.17836	4.034224	1.516503	0.172122	-2.538497	0.040614829	0.379595456	down	yes	60S ribosor	RPL11
Pavir.1KG481100.v	GO:000372	03010(Ribo	large subun	23.051605	15.940253	180.98714	45.226673	0.1723673	-2.536442	0.009182792	0.1749902	down	yes	60S ribosor	RPL39
Pavir.7KG349600.v	GO:000367	NA	kelch motif	11.633694	2.116925	42.033791	37.733776	0.1723836	-2.536306	0.012673177	0.204391821	down	yes	F-box/kelch	At5g60570
Pavir.9NG431700.v	GO:000166	NA	extra-large g	0.626807	0.259578	4.035781	1.102109	0.1725193	-2.535171	0.025499863	0.296006694	down	yes	Extra-large	XLG3
Pavir.9KG557000.v	GO:000484	NA	ring/u-box c	1.2803939	0.2787926	7.0675663	1.9688885	0.1725441	-2.534963	0.021234613	0.268184238	down	yes	NA	NA
Pavir.5KG053900.v	GO:000078	04626(Plan	aurora kinas	1.959395	2.663383	24.070074	2.704307	0.1726568	-2.534021	0.020042908	0.259100884	down	yes	Serine/threc	AUR1
Pavir.7KG338700.v	GO:000373	03010(Ribo	small subun	21.652685	15.889791	165.43213	51.984932	0.1726749	-2.533869	0.008434988	0.166891938	down	yes	40S ribosor	RPS11

Pavir.3KG330900.v	GO:000002	03010(Ribo	large subun	0.437003	1.1607	7.997612	1.25469	0.1726817	-2.533813	0.040443135	0.37902676	down	yes	50S ribosor	rpl20
Pavir.5NG619500.v	GO:00037	NA	nuclear tran	0.53288	0.374003	3.692044	1.556456	0.172789	-2.532917	0.02086636	0.265676635	down	yes	Nuclear tra	NFYB5
Pavir.7NG327900.v	GO:00004	03010(Ribo	large subun	21.35878	12.856997	159.31244	38.515751	0.172957	-2.531514	0.010494863	0.185953556	down	yes	60S ribosor	RPL35B
Pavir.3KG101000.v	GO:00003	03040(Splic	small nucle	1.593215	1.355177	14.545363	2.49703	0.1730034	-2.531128	0.026273734	0.302168052	down	yes	NA	NA
Pavir.4NG036100.v	GO:00037	03010(Ribo	small subun	5.50138	6.855017	60.497509	10.909801	0.1730411	-2.530814	0.009333296	0.176487135	down	yes	40S ribosor	RPS20
Pavir.4NG135100.v	GO:00021	03010(Ribo	large subun	3.8635685	3.0639582	30.950655	9.0539897	0.1731681	-2.529755	0.007261494	0.156441785	down	yes	60S ribosor	RPL31;RPL31
Pavir.5NG328500.v	GO:000484	NA	inhibitor of	1.511507	0.416353	8.860841	2.271198	0.1731812	-2.529646	0.037593848	0.364896315	down	yes	Probable B	BRG3
Pavir.1KG228800.v	GO:000695	NA	universal st	0.515486	1.272164	7.808662	2.495809	0.1734829	-2.527134	0.015252597	0.226367538	down	yes	NA	NA
Pavir.3NG057000.v	GO:000467	04075(Plan	non-specific	1.3957023	0.1476801	3.6057689	5.2901885	0.1734926	-2.527054	0.017200194	0.241064917	down	yes	Receptor pr	ZAR1;ZAR1
Pavir.6NG150400.v	GO:00431	03013(RNA	ring finger c	0.389714	0.388775	3.332906	1.152148	0.1735741	-2.526377	0.025309396	0.295142751	down	yes	E3 ubiquiti	EL5.1
Pavir.2KG083900.v	GO:00037	03010(Ribo	60s riboson	18.522755	14.221747	152.04707	36.58746	0.173587	-2.526269	0.010082425	0.182016394	down	yes	60S ribosor	RPL4A
Pavir.9KG444200.v	GO:000467	03022(Basa	cell division	1.62285	0.65435	10.282683	2.83426	0.1736075	-2.526099	0.016114237	0.232779784	down	yes	Probable se	At1g54610
Pavir.3KG006000.v	GO:00004	03010(Ribo	small subun	17.497091	11.418119	128.36531	38.031166	0.173773	-2.524724	0.007563935	0.157976512	down	yes	40S ribosor	RPS16A
Pavir.8NG124400.v	GO:00044	01212(Fatty	long chain a	9.660406	0.605535	28.644054	30.379499	0.1739296	-2.523425	0.03203367	0.335171564	down	yes	Long chain	LACS2
Pavir.9KG400400.v	GO:003463	00564(Glyc	phospholipa	3.006689	1.268209	5.811719	18.740194	0.1741167	-2.521874	0.006297152	0.144182148	down	yes	Phospholip	PLD1
Pavir.5KG549200.v	NA	NA	trypsin-like	0.326966	0.137395	1.650241	1.015737	0.1741804	-2.521346	0.032209656	0.335901453	down	yes	NA	NA
Pavir.4KG267700.v	GO:00004	03010(Ribo	small subun	9.76772	7.280441	80.894531	16.921633	0.1742878	-2.520457	0.007748777	0.160513448	down	yes	40S ribosor	RPS24A
Pavir.8NG132100.v	GO:000573	00591(Lino	protein auxi	1.5514849	1.0774728	10.796067	4.2844834	0.1743277	-2.520126	0.009128639	0.174757488	down	yes	Protein AU	AXR4;AXR4;AXR4
Pavir.4KG361300.v	NA	NA	0	0.890403	0.518246	6.128421	1.944666	0.174487	-2.518808	0.016879982	0.238971959	down	yes	NA	NA
Pavir.2KG364600.v	GO:000372	03010(Ribo	large subun	17.456642	14.900613	151.593	33.825539	0.1745093	-2.518624	0.010627014	0.18693296	down	yes	60S ribosor	RPL37C
Pavir.2KG539400.v	GO:000017	03018(RNA	exosome co	1.419356	1.680129	15.063051	2.693274	0.1745567	-2.518233	0.018443828	0.24776647	down	yes	Exosome co	RRP41L
Pavir.5KG733800.v	GO:000667	NA	ceramide gl	5.320831	1.398151	19.73535	18.739981	0.1746309	-2.517619	0.00539075	0.132538663	down	yes	NA	NA
Pavir.7KG302200.v	GO:00036	03030(DNA	replication f	0.722555	1.193765	9.024044	1.939287	0.1747936	-2.516276	0.023826711	0.285108608	down	yes	Replication	RFC4
Pavir.5KG093500.v	GO:000367	NA	complement	2.668178	3.494514	28.268888	6.974695	0.17486	-2.515728	0.008601558	0.169030519	down	yes	NA	NA
Pavir.9NG501800.v	GO:000077	03013(RNA	cell cycle ar	0.612404	0.873225	7.432035	1.047314	0.1752055	-2.51288	0.032388568	0.337280734	down	yes	Mitotic che	BUB3.1
Pavir.9NG798400.v	GO:000002	03010(Ribo	small subun	17.901731	13.695434	133.85215	46.459552	0.1752364	-2.512626	0.008347658	0.165831352	down	yes	40S ribosor	-;-
Pavir.3NG005100.v	GO:000521	03040(Splic	permease of	0.723776	1.283275	7.240785	4.206983	0.1753225	-2.511917	0.006010661	0.14035958	down	yes	Protein ZIN	ZIFL1
Pavir.2NG619400.v	GO:00041	NA	aspartyl pro	17.954882	1.442119	70.982437	39.52047	0.1755339	-2.510179	0.029573594	0.321260033	down	yes	Aspartyl pr	AP25
Pavir.5NG424000.v	GO:00046	00940(Pher	peroxidase	4.319475	0.581361	19.86541	8.050587	0.1755565	-2.509992	0.019639541	0.256399048	down	yes	Peroxidase	PER19
Pavir.5NG195700.v	NA	NA	0	0.788462	0.355339	1.740146	4.770208	0.1756895	-2.5089	0.037035209	0.362659348	down	yes	Polygalactu	At1g48100
Pavir.5KG361100.v	GO:000551	NA	vacuolar cat	34.903767	2.7272208	103.07139	110.58766	0.1761263	-2.505317	0.030244225	0.324954613	down	yes	Vacuolar ca	CAX1a;CAX1a
Pavir.9KG625200.v	GO:00045	00500(Starc	glycosyl hyc	1.551246	0.434957	5.44183	5.834503	0.1761391	-2.505213	0.011509197	0.194757431	down	yes	Beta-glucos	BGLU6
Pavir.9NG523200.v	GO:000484	NA	f-box domai	1.28471	0.22561	6.422861	2.144748	0.1762826	-2.504038	0.033537113	0.343486404	down	yes	F-box only	FBX13;FBX13
Pavir.3KG467200.v	GO:000573	NA	adenine nuc	2.580082	0.161567	9.597493	5.9491	0.1763505	-2.503483	0.041141925	0.381591101	down	yes	NA	NA
Pavir.3NG319100.v	GO:00021	03010(Ribo	large subun	62.009411	52.108421	493.01697	153.98706	0.1763789	-2.50325	0.010307706	0.184656922	down	yes	60S ribosor	RPL8
Pavir.2NG486400.v	GO:00021	03010(Ribo	large subun	8.275923	11.086935	86.928757	22.823036	0.1764241	-2.502881	0.006667266	0.148330994	down	yes	60S ribosor	SB62
Pavir.5KG526200.v	GO:00045	00500(Starc	glucan endo	4.4026101	0.2420441	17.585422	8.686348	0.1767926	-2.49987	0.033541416	0.343486404	down	yes	Glucan end	At5g56590;At5g56590;At5g56590;At5g56590
Pavir.1KG241200.v	GO:00004	03010(Ribo	large subun	18.856159	17.727163	161.63548	45.223938	0.1768511	-2.499393	0.008855188	0.172158188	down	yes	60S ribosor	RPL35B
Pavir.2NG630700.v	GO:00021	03010(Ribo	large subun	18.543438	15.343235	152.70969	38.898457	0.176854	-2.499369	0.009791876	0.180534287	down	yes	60S ribosor	RPL22B
Pavir.2NG381300.v	GO:000013	00500(Starc	glycosyl tra	0.252689	0.206759	1.438976	1.158854	0.1768584	-2.499334	0.021866813	0.272237679	down	yes	Probable ga	GAUT4;GAUT4
Pavir.8KG177400.v	GO:000467	04075(Plan	leucine-rich	2.204158	0.215292	12.18038	1.48595	0.1770373	-2.497875	0.0493642	0.41934458	down	yes	Probable le	IMK3
Pavir.8NG132000.v	GO:000563	NA	pentatricope	1.122161	1.130657	7.109046	5.601583	0.1772389	-2.496233	0.006124215	0.141865055	down	yes	Uncharacte	At3g49140;At3g49140
Pavir.3NG001200.v	NA	NA	0	0.606059	0.173067	2.225406	2.164976	0.177462	-2.494418	0.023444325	0.281789387	down	yes	NA	NA
Pavir.5KG549100.v	NA	NA	trypsin-like	1.692419	0.25156	8.454957	2.478602	0.1777993	-2.491679	0.044560042	0.398414767	down	yes	NA	NA
Pavir.9NG137000.v	GO:00045	00500(Starc	carbohydrat	16.216372	2.06158	59.304192	43.486317	0.1778175	-2.491531	0.019454595	0.255789208	down	yes	Glucan end	At4g34480
Pavir.6NG087700.v	GO:000437	00500(Starc	glycosyl tra	3.1195561	0.9530494	13.45864	9.4412352	0.177844	-2.491316	0.005222424	0.130466854	down	yes	Soluble sta	SSIIIA;SSIIIA;SSIIIA;SSIIIA;SSIIIA;SSIIIA;SSIIIA
Pavir.2NG390400.v	GO:000367	NA	sbp domain	2.799127	0.478684	13.74037	4.67819	0.1779624	-2.490356	0.019936643	0.258492149	down	yes	Squamosa p	SPL17
Pavir.2KG464200.v	GO:00045	00500(Starc	glucan endo	5.045002	2.260624	16.046558	24.992008	0.1780185	-2.489901	0.003695797	0.107064137	down	yes	Glucan end	At1g32860
Pavir.9KG496900.v	NA	NA	0	0.781967	0.310134	4.10542	2.027898	0.1780604	-2.489561	0.031407987	0.331486598	down	yes	NA	NA
Pavir.6NG274600.v	GO:000367	NA	remorin, c-t	4.570505	0.747314	21.908001	7.957225	0.1780606	-2.48956	0.015734663	0.230127863	down	yes	NA	NA
Pavir.9NG657900.v	NA	NA	0	1.9529104	1.0594223	12.239867	4.6762401	0.1780748	-2.489445	0.011329589	0.192705582	down	yes	NA	NA
Pavir.9NG256200.v	GO:00055	03018(RNA	chaperonin	3.7476533	5.5196522	46.344679	5.6809313	0.1781297	-2.489	0.022257575	0.274511146	down	yes	Chaperonin	CPN60II;CPN60II;CPN60II
Pavir.7NG237300.v	GO:000002	03010(Ribo	large subun	31.224121	30.404488	275.97031	69.829208	0.1782206	-2.488264	0.011960658	0.198234581	down	yes	60S ribosor	RPL6
Pavir.2NG210700.v	GO:000842	00500(Starc	periplasmic	1.4472032	1.1297143	8.5103284	5.9310379	0.17844	-2.486489	0.002381529	0.080036845	down	yes	Beta-xylosi	Xyl2;Xyl2
Pavir.9KG380700.v	GO:000981	00906(Caro	flavonol 3-c	0.531726	1.51991	8.543391	2.93762	0.1786982	-2.484403	0.01681435	0.238475181	down	yes	UDP-gluco	GT7
Pavir.7NG404700.v	GO:000557	03010(Ribo	bifunctional	1.894382	2.199777	1.60191	21.29562	0.1788035	-2.483553	0.036077948	0.357007308	down	yes	36.4 kDa p	TPRP-F1
Pavir.2KG059900.v	GO:00021	03010(Ribo	large subun	6.316689	4.380939	46.048565	13.755543	0.1788778	-2.482954	0.009318169	0.176467517	down	yes	60S acidic	RPP2A
Pavir.1KG139700.v	NA	NA	0	1.298799	0.113404	5.879416	2.015285	0.1788799	-2.482937	0.03549142	0.3540943	down	yes	NA	NA
Pavir.4NG246300.v	GO:000467	04626(Plan	leucine-rich	1.423334	0.244832	7.034443	2.291042	0.1788825	-2.482916	0.027148156	0.306772055	down	yes	Receptor pr	At4g34220
Pavir.1NG560900.v	GO:00037	03010(Ribo	large subun	24.625549	14.419359	173.91982	44.25346	0.1789628	-2.482268	0.013346748	0.209368875	down	yes	60S ribosor	RPL23A
Pavir.7KG350700.v	NA	NA	0	38.837578	2.695392	137.96101	93.977646	0.1790688	-2.481414	0.036348009	0.358264361	down	yes	NA	NA
Pavir.5KG427000.v	NA	NA	e3 ubiquitin	0.71262	0.488421	5.235998	1.467214	0.179174	-2.480567	0.010442116	0.185444601	down	yes	NA	NA
Pavir.9KG629700.v	GO:000002	03010(Ribo	small subun	23.696789	19.334734	184.06433	56.079113	0.1791909	-2.480431	0.010640653	0.187067868	down	yes	40S ribosor	RPS17
Pavir.1NG095100.v	GO:00045	00500(Starc	glycosyl hyc	1.090755	0.077618	2.051134	4.461163	0.1794103	-2.478666	0.046662295	0.406501837	down	yes	Glucan end	At2g27500
Pavir.5KG439900.v	GO:000002	03010(Ribo	large subun	26.896437	21.352884	210.02178	58.906986	0.179413	-2.478644	0.011326075	0.192705582	down	yes	60S ribosor	RPL24;RPL24
Pavir.5NG088500.v	GO:00164	00860(Porp	nad(p)-bind	35.955376	5.694618	119.68733	112.41242	0.1794487	-2.478357	0.018734335	0.250273838	down	yes	Short-chain	TIC32B
Pavir.3NG178300.v	GO:001602	00100(Ster	cycloeucale	4.017717	3.249118	27.738817	12.743295	0.1795073	-2.477886	0.003688422	0.107064137	down	yes	Cycloeucal	CPI1
Pavir.8KG350700.v	GO:000015	03440(Hom	dna repair p	1.271452	1.3859476	11.71901	3.083038	0.1795292	-2.47771	0.015246782	0.226367538	down	yes	DNA repair	RAD51A;RAD51A
Pavir.8KG395600.v	GO:00055	NA	synaptotagn	0.575825	0.65729	4.379924	2.487517	0.1795596	-2.477465	0.011107354	0.190687546	down	yes	FT-interact	FTIP3
Pavir.4KG199800.v	NA	NA	0	0.668335	1.32444	5.42906	5.666349	0.1796036	-2.477112	0.019590106	0.256267806	down	yes	NA	NA
Pavir.5KG114100.v	NA	NA	phosphatidy	0.557887	0.541515	4.827171	1.291915	0.1796677	-2.476597	0.014487156	0.219044633	down	yes	NA	NA
Pavir.7KG305000.v	GO:00007	NA	histone h4 (	3.893462	17.376022	111.66167	6.503217	0.1799983	-2.473944	0.048123684	0.413516993	down	yes	Histone H4	-
Pavir.6NG296900.v	GO:00021	NA	trna (cytidin	0.704992	0.800826	5.633535	2.731521	0.1800129	-2.473828	0.019615847	0.256267806	down	yes	NA	NA
Pavir.3KG262000.v	GO:000573	NA	ras suppress	1.358084	2.54239	12.349401	9.312334	0.1800629	-2.473427	0.003639779	0.106425992	down	yes	NA	NA
Pavir.5NG214900.v	GO:00021	03010(Ribo	large subun	7.5234602	3.669545	44.897152	17.123241	0.180473	-2.470145	0.006821301	0.150674161	down	yes	60S ribosor	RPL29A;RPL29A
Pavir.9NG678400.v	GO:000563	NA	t14p4.7 pro	5.883151	0.260524	21.012177	13.013404	0.1805605	-2.469446	0.037896418	0.366319044	down	yes	NA	NA
Pavir.4NG041600.v	GO:000582	NA	protein argi	0.549373	1.083365	8.191645	0.850014	0.1805795	-2.469294	0.047802649	0.412174478	down	yes	Protein argi	PRMT10
Pavir.9NG588800.v	GO:000372	03010(Ribo	large subun	17.96982	11.832228	130.72437	34.310555	0.1805803	-2.469288	0.011631084	0.195737083	down	yes	60S ribosor	RPL18B
Pavir.7KG054700.v	GO:00044	00380(Tryp	indole-3-py	0.908011	0.164341	2.375739	3.560262	0.1806523	-2.468713	0.030934426	0.329076785	down	yes	Probable in	YUC5
Pavir.3NG305900.v	GO:000551	04075(Plan	cycline//sub	0.534059	2.095366	12.607671	1.942301	0.1807168	-2.468197	0.031426157	0.331492134	down	yes	Cyclin-D5-	CYCD5-2
Pavir.5NG323700.v	NA	NA	0	1.278223	0.293308	4.913919	3.781498	0.180731	-2.468084	0.022299478	0.274887887	down	yes	NA	NA
Pavir.1NG030900.v	GO:000367	04712(Circ	tcp family t	1.692435	0.706495	8.287416	4.973057	0.1809083	-2.466669	0.032782187	0.339131364	down	yes	Transcriptio	TCP7

Pavir.1NG064500.v	GO:000373	03010(Ribo	large subun	16.117409	10.010271	112.70645	31.697463	0.1809347	-2.466459	0.010860432	0.188567538	down	yes	60S ribosor	RPL27AB;RPL27AB
Pavir.3KG361800.v	GO:000373	03010(Ribo	30s/40s ribo	60.277477	52.774681	469.03409	155.08232	0.1811735	-2.464827	0.011193439	0.191717364	down	yes	40S ribosor	RPS4
Pavir.2NG410700.v	GO:002262	03010(Ribo	large subun	11.606929	9.3943785	91.201856	24.706675	0.1811886	-2.464436	0.006477247	0.14625576	down	yes	60S ribosor	RPL32A;RPL32B
Pavir.2NG489500.v	GO:000681	NA	ferredoxin/a	2.953279	1.576742	12.055452	12.919173	0.1813849	-2.462873	0.006131802	0.141953284	down	yes	Photosynth	PNSB3
Pavir.4KG264200.v	GO:000002	03010(Ribo	large subun	9.32178	5.859578	63.263622	20.402464	0.1814518	-2.462342	0.005825937	0.137393622	down	yes	60S ribosor	RPL11
Pavir.2NG203000.v	GO:000373	03010(Ribo	small subun	10.502265	10.72397	91.864761	25.080229	0.1815062	-2.46191	0.009646861	0.179602151	down	yes	40S ribosor	RPS2D
Pavir.2KG386600.v	GO:000367	NA	histone-bin	1.201525	1.072381	11.154282	1.368897	0.1815758	-2.461356	0.033338724	0.342343779	down	yes	WD-40 rep	MSI2
Pavir.9KG655000.v	GO:000563	NA	protein of u	2.421937	0.324409	8.440578	6.678343	0.1816496	-2.46077	0.018737399	0.250273838	down	yes	Protein ROI	REL2
Pavir.1KG380300.v	GO:000373	03010(Ribo	small subun	6.404399	3.759045	42.250183	13.689935	0.1816844	-2.460494	0.007198838	0.155807577	down	yes	40S ribosor	RPS11
Pavir.5NG426500.v	GO:000014	00500(Star	callose synt	1.046052	0.680952	6.5017828	3.000676	0.1817429	-2.460029	0.004587913	0.122035291	down	yes	Callose syn	CALS12;CALS12
Pavir.9NG711700.v	GO:000636	03010(Ribo	small subun	51.854584	40.792747	399.08502	110.38043	0.181852	-2.459163	0.013132777	0.20837747	down	yes	40S ribosor	RPS7
Pavir.1KG066400.v	GO:000002	03010(Ribo	small subun	7.051869	2.880452	42.689713	11.92431	0.1818639	-2.459069	0.011055934	0.190127899	down	yes	40S ribosor	RPS14B
Pavir.1NG560600.v	GO:000372	03010(Ribo	large subun	29.454807	27.299402	238.35957	73.619812	0.1819165	-2.458651	0.008892132	0.172485318	down	yes	60S ribosor	RPL37C
Pavir.9NG031700.v	GO:000373	03010(Ribo	small subun	6.9556256	7.1624461	58.366093	19.209129	0.181992	-2.458053	0.005295915	0.131031695	down	yes	40S ribosor	RPS23;RPS23
Pavir.6KG031400.v	NA	NA	0	31.066343	22.275738	225.03908	67.713188	0.1822089	-2.456334	0.006284031	0.144060155	down	yes	NA	NA
Pavir.2KG587400.v	GO:000370	04141(Prot	bzip transcr	4.749912	1.046941	21.484522	10.310239	0.182321	-2.455447	0.011872987	0.197303733	down	yes	Transcriptic	RF2b
Pavir.8NG050800.v	NA	NA	0	2.145469	0.330547	6.888694	6.690826	0.1823346	-2.45534	0.029390074	0.320150906	down	yes	NA	NA
Pavir.2NG044000.v	GO:000072	NA	cst complex	0.519187	0.199416	2.191366	1.749228	0.1823591	-2.455146	0.03369773	0.343927244	down	yes	CST compl	TEN1
Pavir.9NG744600.v	GO:000573	NA	copper trans	2.802898	1.436178	11.816794	11.405469	0.1825436	-2.453687	0.004593583	0.122061307	down	yes	Heavy meta	HIPP05
Pavir.8KG164200.v	GO:000467	04626(Plan	non-specific	1.871542	0.169439	5.758104	5.40489	0.1828346	-2.451389	0.033476644	0.343010075	down	yes	G-type lecti	At1g11410
Pavir.7NG257100.v	GO:000002	03010(Ribo	large subun	24.534182	18.452752	182.9532	52.134514	0.1828469	-2.451229	0.012207888	0.200387495	down	yes	60S ribosor	RPL23A;RPL23A
Pavir.9KG617500.v	GO:000003	00062(Fatty	3-ketoacyl-c	5.603397	6.145738	40.066517	24.161091	0.1829297	-2.450639	0.005262108	0.1307627	down	yes	3-ketoacyl-C	FDH
Pavir.7KG133500.v	GO:000373	03010(Ribo	large subun	32.004745	23.012188	226.75194	73.633003	0.1831548	-2.448865	0.010094124	0.182016394	down	yes	60S ribosor	Os01g0679700
Pavir.4KG067500.v	GO:000373	03010(Ribo	small subun	15.409999	13.991946	128.08986	32.440018	0.1831556	-2.448858	0.007810311	0.160955646	down	yes	40S ribosor	RPS30A
Pavir.5KG270900.v	GO:000374	03013(RNA	translation i	4.49608	2.967489	29.909971	10.781921	0.1834166	-2.446804	0.005575142	0.134904319	down	yes	Eukaryotic	TIF3F1
Pavir.3KG401100.v	NA	NA	phosphatidy	1.785361	0.297954	7.23228	4.122684	0.1834717	-2.44637	0.017905635	0.244744653	down	yes	NA	NA
Pavir.8KG188800.v	GO:004665	NA	plastocyanin	4.090479	1.088878	20.190235	8.014791	0.1836324	-2.445107	0.016530698	0.236300643	down	yes	NA	NA
Pavir.7NG438700.v	GO:000415	00240(Pyri	dihydroorot	0.494678	0.582245	4.757824	1.105525	0.1836703	-2.44481	0.027448546	0.308502611	down	yes	Dihydrooro	PYRD
Pavir.6KG409600.v	GO:000373	03010(Ribo	small subun	21.87097	20.725784	180.13268	51.712624	0.1837292	-2.444347	0.01125204	0.192178627	down	yes	40S ribosor	RPS25B
Pavir.5NG345900.v	GO:000373	03010(Ribo	small subun	15.393259	9.861265	99.879295	37.538166	0.1837796	-2.443952	0.00531046	0.131269684	down	yes	Ubiquitin-4	UBF9
Pavir.9KG060700.v	GO:000534	NA	sodium-bile	0.978146	0.266486	5.064718	1.70646	0.1838132	-2.443688	0.027251386	0.307297815	down	yes	Probable so	BASS1
Pavir.9KG206400.v	GO:000372	03010(Ribo	large subun	16.466764	12.004911	120.13228	34.745171	0.1838336	-2.443528	0.010690334	0.187520043	down	yes	60S ribosor	RPL18B
Pavir.4KG059900.v	GO:000474	00230(Puri	ribonucleos	0.841817	1.634601	12.27757	1.183181	0.1839732	-2.442432	0.029329012	0.319970669	down	yes	Ribonucleo	RNR1
Pavir.3NG053900.v	GO:000367	NA	wd40-like b	1.908613	1.317502	11.227663	6.304587	0.1840103	-2.442141	0.00413644	0.114658043	down	yes	NA	NA
Pavir.5KG474400.v	GO:000455	00500(Star	4-alpha-d-((	0.518894	0.399144	3.356856	1.631761	0.1840266	-2.442014	0.012544503	0.203863881	down	yes	Alpha-amyl	AMY3
Pavir.5KG455700.v	GO:000578	00240(Pyri	auxin efflux	1.471398	0.20232	5.672947	3.421497	0.1840374	-2.441929	0.027468985	0.308622709	down	yes	Auxin efflu	PIN3A
Pavir.6NG016300.v	GO:000218	03010(Ribo	large subun	19.60915	28.276699	198.3251	61.750267	0.184123	-2.441258	0.01078631	0.188149509	down	yes	60S acidic	RPP1A
Pavir.7KG152000.v	NA	04626(Plan	leucine-rich	0.314376	0.157689	1.302473	1.261378	0.1841234	-2.441255	0.018227472	0.246847474	down	yes	NA	NA
Pavir.4NG010200.v	GO:000027	00500(Star	x-box transc	2.778629	0.420931	9.433698	7.933458	0.1842305	-2.440416	0.013541201	0.211239519	down	yes	Cellulose sy	CSLD2
Pavir.4NG190000.v	GO:000563	00062(Fatty	very-long-ch	10.32836	2.8537493	43.802022	27.695111	0.1843726	-2.439304	0.011769343	0.196771493	down	yes	3-ketoacyl-C	KCS11;KCS11
Pavir.2KG149900.v	GO:000218	03010(Ribo	large subun	14.941661	11.097111	106.82786	34.363266	0.1844222	-2.438916	0.009547389	0.178730357	down	yes	60S acidic	RPP2A
Pavir.5KG326600.v	GO:000367	NA	pollen ole e	2.428869	0.455748	11.749113	3.889495	0.1844548	-2.43866	0.030767636	0.328231795	down	yes	NA	NA
Pavir.6NG190000.v	GO:000431	01212(Fatty	enoyl- (fabi	2.994785	1.501246	19.41885	4.953669	0.1844713	-2.438531	0.01184767	0.19717125	down	yes	Enoyl-[acyl-	Os08g0327400
Pavir.1KG436500.v	NA	NA	0	2.172358	0.708049	7.719461	7.867965	0.1847904	-2.436038	0.020046477	0.259100884	down	yes	NA	NA
Pavir.7NG118900.v	GO:000373	03010(Ribo	large subun	27.39522	23.866381	210.04837	66.903984	0.1850918	-2.433687	0.010149106	0.182343582	down	yes	60S ribosor	Os01g0679700
Pavir.1NG434400.v	GO:000563	NA	max dimeriz	1.3262254	0.5105643	5.6519593	4.2484985	0.1855257	-2.430309	0.007905249	0.162028857	down	yes	Transcriptic	BHLH96;BHLH94
Pavir.4NG210700.v	GO:000333	NA	transmembr	0.757999	1.043248	5.849395	3.849908	0.1857089	-2.428885	0.010139728	0.182343582	down	yes	Amino acid	AVT1C
Pavir.3KG032700.v	GO:000551	03030(DNA	replication f	2.8153264	1.3084902	16.939505	5.2652647	0.1857176	-2.428818	0.012859964	0.206225958	down	yes	Replication	RPA2A;RPA2A
Pavir.3KG042200.v	GO:000551	NA	protease inh	9.489028	3.042741	42.182495	25.290117	0.1857312	-2.428712	0.006921767	0.152266706	down	yes	Non-specifi	LTP2
Pavir.2KG359100.v	GO:000367	04712(Circ	f25a4.19 pr	0.876773	0.184874	3.49909	2.215421	0.1857809	-2.428326	0.02650141	0.303393505	down	yes	Transcriptic	DIVARICATA
Pavir.2NG093200.v	GO:000373	03010(Ribo	small subun	26.07023	22.04924	197.66754	61.327991	0.1857927	-2.428235	0.011056595	0.190127899	down	yes	40S ribosor	RPS2C
Pavir.7KG128700.v	GO:000004	03010(Ribo	large subun	26.550882	14.579819	178.45075	42.907692	0.1858104	-2.428097	0.015062482	0.224669567	down	yes	60S ribosor	RPL35B
Pavir.1NG486700.v	GO:000367	NA	protein of u	0.951606	1.308895	10.686256	1.458544	0.1861291	-2.425624	0.036238341	0.357746504	down	yes	Probable tra	At4g00390
Pavir.9NG619900.v	GO:000072	NA	gins comple	0.586967	0.936092	7.164844	1.014897	0.1861989	-2.425083	0.046211836	0.404908717	down	yes	NA	NA
Pavir.4KG404500.v	GO:000002	03010(Ribo	small subun	33.450523	23.389225	236.00577	69.248451	0.1862046	-2.425039	0.012963263	0.207263269	down	yes	40S ribosor	-
Pavir.7KG337300.v	GO:000004	03010(Ribo	large subun	23.656888	18.552859	170.17168	56.395356	0.1863014	-2.42429	0.011039834	0.190066184	down	yes	60S ribosor	RPL7D;RPL7D
Pavir.6KG236000.v	GO:000010	NA	thioredoxin-	1.410768	3.909463	22.20853	6.339835	0.1863585	-2.423847	0.01889053	0.251024069	down	yes	Thioredoxin	OsI_29059
Pavir.6KG068800.v	GO:000372	03010(Ribo	large subun	32.081497	19.748798	218.46011	59.552513	0.1864314	-2.423283	0.013013264	0.20735682	down	yes	60S ribosor	RPL34
Pavir.4KG315100.v	GO:000563	00062(Fatty	very-long-ch	20.22179	5.145451	72.947586	63.084766	0.1864795	-2.422911	0.01448465	0.219044633	down	yes	3-ketoacyl-C	KCS11
Pavir.7NG317700.v	GO:000002	03010(Ribo	large subun	25.479527	22.028141	189.369	65.304176	0.1865437	-2.422415	0.010119643	0.182213283	down	yes	60S ribosor	RPL12A
Pavir.5NG443500.v	GO:000373	03010(Ribo	large subun	22.800907	14.80352	155.4395	46.057281	0.1866254	-2.421782	0.011731746	0.196465165	down	yes	60S ribosor	RPL28A
Pavir.5KG704300.v	GO:000667	NA	ceramide glu	1.7138145	0.4659697	5.9465635	5.7247585	0.1867641	-2.420711	0.011477541	0.194517652	down	yes	NA	NA
Pavir.1KG308100.v	GO:000484	NA	inhibitor of	0.618162	0.111567	2.403643	1.499928	0.1869388	-2.419362	0.04001806	0.377133998	down	yes	Probable B(	BRG3
Pavir.1KG294000.v	GO:000002	03010(Ribo	large subun	23.027782	16.858227	156.40262	56.932417	0.1869642	-2.419166	0.011776771	0.196771493	down	yes	60S ribosor	RPL3;RPL3
Pavir.2NG474500.v	NA	NA	rna-directed	0.952216	0.251243	3.886888	2.549261	0.1869843	-2.419011	0.032627993	0.338561609	down	yes	NA	NA
Pavir.1KG097300.v	GO:000467	04626(Plan	non-specific	3.387453	0.383075	10.604317	9.544901	0.1871302	-2.417885	0.019581193	0.256267806	down	yes	Protein STF	SRF8
Pavir.3KG445100.v	NA	NA	mitotic chec	2.691665	1.953881	7.85564	16.950781	0.1872719	-2.416794	0.004399073	0.118484849	down	yes	NA	NA
Pavir.2KG590800.v	GO:000218	03010(Ribo	large subun	14.072617	10.620466	101.4577	30.033176	0.1877931	-2.412784	0.007138293	0.155046644	down	yes	60S ribosor	RPL22B
Pavir.2NG530700.v	NA	NA	0	3.84684	0.786182	15.280664	9.326456	0.1882797	-2.40905	0.011806856	0.196988904	down	yes	Uncharacter	At4g28100
Pavir.5KG641300.v	GO:000002	03010(Ribo	small subun	9.936199	5.681611	65.402679	17.437466	0.1885295	-2.407138	0.008954326	0.173155379	down	yes	40S ribosor	RPS15
Pavir.4KG244500.v	GO:000449	00903(Lim	cytochrome	1.069924	0.252777	3.74699	3.260577	0.1887532	-2.405427	0.027155773	0.306772055	down	yes	Cytochrom	CYP94B1
Pavir.1NG145000.v	GO:000465	00040(Pent	glycoside hy	3.276585	1.406736	16.786585	7.985636	0.1890554	-2.403119	0.007474076	0.157697172	down	yes	Probable pc	GSVIVT00026920001
Pavir.8KG385200.v	GO:001014	00561(Glyc	ancient ubic	1.072001	0.67425	3.967883	5.267036	0.1890922	-2.402838	0.009594386	0.178946539	down	yes	Probable gl	GPAT3
Pavir.9NG813000.v	GO:000419	NA	aspartyl pro	33.845806	3.27969	120.27323	75.971512	0.1891796	-2.402172	0.033838764	0.344372661	down	yes	Aspartyl pr	AP25
Pavir.9NG047600.v	GO:004665	NA	plastocyanin	20.184496	2.196397	83.867249	34.399269	0.1892412	-2.401702	0.027537007	0.308757967	down	yes	NA	NA
Pavir.5NG460800.v	GO:000367	NA	protein of u	0.572604	0.248001	2.237735	2.092841	0.189491	-2.399799	0.033860375	0.344406025	down	yes	NA	NA
Pavir.1KG010000.v	GO:000372	03010(Ribo	large subun	10.909253	6.867582	74.398796	19.381996	0.1895573	-2.399294	0.00893414	0.172943049	down	yes	60S ribosor	RPL37A
Pavir.1KG133200.v	GO:000372	NA	rna binding	0.62768	1.157206	7.9629869	1.430941	0.1900042	-2.395897	0.020231538	0.260721745	down	yes	Pumilio hor	APUM1;APUM1
Pavir.5NG606500.v	GO:000372	03010(Ribo	large subun	22.699934	19.961227	176.06754	48.283485	0.1901536	-2.394763	0.014586056	0.219883759	down	yes	60S ribosor	RPL18C
Pavir.4KG144100.v	GO:000218	03010(Ribo	large subun	26.904865	16.425537	177.32443	50.504238	0.1901885	-2.394498	0.013393971	0.209775598	down	yes	60S ribosor	RPL31
Pavir.1NG086600.v	GO:001602	02010(ABC	abc transpo	0.425149	0.956134	2.154342	5.107843	0.1902021	-2.394395	0.01149982	0.194719577	down	yes	Putative mu	Os02g0190300;Os02g0190300
Pavir.7KG229100.v	GO:000002	03010(Ribo	large subun	37.611207	33.81028	290.12898	85.333225	0.1902228	-2.394238	0.013694157	0.212470074	down	yes	60S ribosor	RPL23A;RPL23A;RPL23A

Pavir.7NG229100.v4	GO:000422	04144(Endo	mitochondri	1.1500593	1.1324259	9.184443	2.8126262	0.1902536	-2.394005	0.015691988	0.229861626	down	yes	NA	NA
Pavir.1KG205100.v4	GO:000367	NA	probable zi	0.620914	0.153979	2.666281	1.40174	0.190484	-2.392258	0.032737225	0.339131364	down	yes	NA	NA
Pavir.8NG005900.v4	GO:000663	01212(Fatty	omega-3 fat	3.462396	0.228335	11.557333	7.812967	0.1905356	-2.391868	0.037792729	0.365787274	down	yes	Fatty acid d	DES3
Pavir.7KG249800.v4	GO:000373	03010(Ribo	large subun	15.437673	10.849601	104.19366	33.738079	0.1905818	-2.391518	0.007728248	0.160427326	down	yes	60S ribosor	RPL14A
Pavir.5NG343600.v4	GO:000218	03010(Ribo	large subun	15.475224	20.143671	147.40465	39.371033	0.1907041	-2.390592	0.013649069	0.212161115	down	yes	60S ribosor	RPL26A
Pavir.9KG652600.v4	GO:000002	03010(Ribo	mitochondri	11.976152	12.10472	85.33741	40.801193	0.190908	-2.38905	0.004367365	0.118361397	down	yes	40S ribosor	-
Pavir.4KG009200.v4	NA	NA	0	35.259499	19.694727	240.25722	47.535671	0.1909506	-2.388729	0.016023744	0.232455648	down	yes	NA	NA
Pavir.3KG150900.v4	GO:000373	03010(Ribo	30s/40s ribo	40.412041	36.465281	302.7334	99.78582	0.1909904	-2.388428	0.013393739	0.209775598	down	yes	40S ribosor	RPS4;RPS4
Pavir.1KG491000.v4	GO:000046	03008(Ribo	ribosomal p	5.669942	7.685592	63.533852	6.346291	0.1911206	-2.387445	0.029279566	0.319819571	down	yes	H/ACA ribo	At5g08180
Pavir.9NG524900.v4	GO:000002	03010(Ribo	small subun	11.426295	8.293585	80.376175	22.736719	0.1912455	-2.386502	0.014996634	0.224070926	down	yes	40S ribosor	RPS19A
Pavir.3KG426200.v4	NA	NA	0	0.196783	0.290143	1.114378	1.431687	0.1912465	-2.386495	0.04036924	0.378880534	down	yes	NA	NA
Pavir.2KG061200.v4	GO:000218	03010(Ribo	large subun	22.631174	21.515709	180.62801	49.930401	0.1914781	-2.384749	0.012812328	0.205647752	down	yes	60S acidic r	RPP2A
Pavir.8NG170100.v4	GO:000367	NA	f12a21.28	3.5877468	0.9708214	15.730442	8.0736673	0.1915034	-2.384558	0.011709461	0.196235312	down	yes	NA	NA
Pavir.9KG473500.v4	GO:000636	03010(Ribo	small subun	45.723061	39.981831	340.94028	106.21668	0.1916662	-2.383332	0.014401881	0.2186351	down	yes	40S ribosor	RPS7
Pavir.2NG374300.v4	GO:000967	NA	auxin efflux	0.654327	0.407829	3.099415	2.441586	0.1916903	-2.383151	0.027691915	0.309361931	down	yes	Protein PIN	PILS1
Pavir.2NG060000.v4	GO:000373	03010(Ribo	large subun	14.67261	15.501439	123.26888	34.065952	0.1917824	-2.382458	0.01500255	0.224070926	down	yes	60S ribosor	RPL4A
Pavir.2KG086000.v4	GO:000373	03010(Ribo	large subun	7.092207	7.069499	55.886005	17.937138	0.1918329	-2.382078	0.009716225	0.180068207	down	yes	60S ribosor	RPL4A
Pavir.6KG374700.v4	GO:002262	03010(Ribo	large subun	14.55459	10.061328	101.6226	26.543985	0.1920619	-2.380357	0.009362574	0.176596389	down	yes	60S ribosor	RPL32A
Pavir.7KG158800.v4	NA	04626(Plan	protein pho	1.147783	0.472975	4.893061	3.542882	0.1921253	-2.379881	0.00820112	0.1644294	down	yes	NA	NA
Pavir.3NG035200.v4	GO:000573	00940(Pher	transferase f	2.202054	1.524393	13.732106	5.663637	0.1921271	-2.379867	0.007848714	0.161235497	down	yes	Uncharacter	At3g50280
Pavir.9NG471700.v4	GO:000367	NA	protein of u	0.641361	0.687679	4.789335	2.127181	0.1921545	-2.379661	0.01858196	0.248940219	down	yes	NA	NA
Pavir.2KG286800.v4	GO:000578	NA	delta(8)-fatt	42.122829	5.082654	137.59486	107.96497	0.1922362	-2.379048	0.030140391	0.324425589	down	yes	Delta(8)-fat	SLD2
Pavir.8NG332500.v4	GO:000373	03010(Ribo	small subun	16.484135	17.251745	129.28589	46.079506	0.1923748	-2.378008	0.010497099	0.185953556	down	yes	40S ribosor	RPS29
Pavir.7KG128100.v4	GO:000046	03010(Ribo	large subun	7.868171	5.21112	52.541138	15.364451	0.1926099	-2.376246	0.009781412	0.180534287	down	yes	60S ribosor	RPL35B
Pavir.9NG243000.v4	GO:000576	NA	methyltrans	3.574592	2.086775	21.053614	8.335601	0.1926342	-2.376064	0.00669079	0.148753231	down	yes	Probable m	At1g33170
Pavir.6NG046400.v4	NA	NA	ppr repeat (	2.524489	1.823983	18.48942	4.080834	0.1926638	-2.375842	0.013250726	0.209130801	down	yes	Pentatricop	At3g02650
Pavir.3KG296000.v4	GO:000373	03010(Ribo	large subun	2.003014	3.253105	23.109249	4.167779	0.192694	-2.375617	0.02217354	0.274506234	down	yes	50S ribosor	RPL21M
Pavir.2KG318600.v4	GO:000372	03010(Ribo	large subun	56.9207	43.49736	401.68912	119.40984	0.1927044	-2.375539	0.015604104	0.229110202	down	yes	60S ribosor	RPL13B
Pavir.2NG040500.v4	GO:000367	NA	transcriptio	4.378213	1.032065	16.95454	11.115522	0.1927419	-2.375258	0.014316258	0.217778448	down	yes	Transcriptio	PCF6
Pavir.6KG019400.v4	GO:000218	03010(Ribo	60s acidic r	37.978836	34.256735	289.912	84.521242	0.1929198	-2.373927	0.01464544	0.220258684	down	yes	60S acidic r	RPP1A;RPP1A
Pavir.1KG401400.v4	GO:000373	03010(Ribo	large subun	62.489445	53.306244	475.86505	123.93566	0.1930569	-2.372902	0.017093618	0.240475103	down	yes	60S ribosor	RPL14A
Pavir.8KG013600.v4	GO:000367	04146(Pero	peroxisoma	0.697542	2.141853	12.970138	1.732086	0.1931269	-2.372379	0.047348305	0.40989681	down	yes	NA	NA
Pavir.9KG109000.v4	GO:000449	00905(Bras	brassinoster	2.429504	0.337317	9.867743	4.436908	0.1934211	-2.370183	0.032614321	0.338561609	down	yes	Cytochrome	CYP85A1
Pavir.6KG394400.v4	GO:000047	03010(Ribo	ribosomal p	26.036786	22.487523	190.46342	60.370983	0.1934516	-2.369956	0.014576863	0.219883759	down	yes	60S ribosor	RPL10A;RPL10A
Pavir.6NG059300.v4	GO:000372	03010(Ribo	large subun	10.782001	8.671141	80.040062	20.236391	0.1939951	-2.365908	0.009735123	0.180097383	down	yes	60S ribosor	RPL34
Pavir.9NG664300.v4	GO:000016	01230(Bios	anthranilate	0.405331	1.056605	4.623245	2.902448	0.1942593	-2.363944	0.019493681	0.25596585	down	yes	Anthranilat	OsASA2
Pavir.7NG435700.v4	GO:000563	04075(Plan	response re	2.448616	0.198937	5.59068	8.031605	0.1943545	-2.363237	0.043248342	0.392368384	down	yes	Two-compo	RR6
Pavir.7NG046500.v4	GO:000373	03010(Ribo	small subun	10.477	10.802698	83.846001	25.474669	0.1946539	-2.361017	0.007859026	0.16135912	down	yes	40S ribosor	RPS12;RPS12
Pavir.5KG607200.v4	GO:000373	03010(Ribo	30s ribosom	8.088535	8.084349	65.852936	17.231934	0.194655	-2.361009	0.009433129	0.17748171	down	yes	40S ribosor	RPS23
Pavir.7NG446300.v4	GO:000550	NA	c2 calcium/l	6.766144	1.762827	25.878025	17.878349	0.1949195	-2.35905	0.014818136	0.221981462	down	yes	FT-interacti	FTIP3
Pavir.2NG410800.v4	GO:002262	03010(Ribo	60s ribosom	5.263358	7.429586	52.489357	12.552876	0.1951493	-2.35735	0.013010132	0.20735682	down	yes	60S ribosor	RPL32A
Pavir.2NG550600.v4	GO:000373	03010(Ribo	ribosomal p	3.925759	3.753172	31.909584	7.433283	0.1951798	-2.357125	0.016825019	0.238475181	down	yes	40S ribosor	Os08g0117200
Pavir.3KG256400.v4	GO:000375	03030(DNA	fk506-bindi	0.975147	1.543907	11.549372	1.34791	0.1953167	-2.356113	0.036576092	0.359804611	down	yes	Peptidyl-pro	FKBP53
Pavir.2KG488900.v4	NA	NA	0	3.245497	1.949023	20.460638	6.130883	0.195345	-2.355904	0.021105063	0.267176726	down	yes	NA	NA
Pavir.3KG297000.v4	NA	NA	0	1.38108	0.142495	4.895821	2.892231	0.1956298	-2.353802	0.045838055	0.403360351	down	yes	NA	NA
Pavir.1KG524700.v4	GO:000563	03030(DNA	proliferating	5.249136	9.438113	66.995422	8.066404	0.1956687	-2.353515	0.025276898	0.295038486	down	yes	Proliferating	PCNA
Pavir.4NG105500.v4	GO:000579	NA	gdsl/sgnh-li	1.755333	0.176286	6.257266	3.611486	0.1957308	-2.353057	0.046817953	0.407196976	down	yes	Protein AL	AXY4
Pavir.7KG207900.v4	NA	NA	bowman-bi	2.844687	9.208049	41.132671	20.443905	0.1957357	-2.353021	0.010644641	0.187067868	down	yes	NA	NA
Pavir.8NG092700.v4	GO:000372	03010(Ribo	60s ribosom	18.118547	17.147375	138.0262	42.091095	0.1957942	-2.35259	0.008067586	0.162969566	down	yes	60S ribosor	RPL18C
Pavir.7NG397600.v4	GO:000467	04626(Plan	protein tyro	1.196245	0.197962	4.05704	3.053661	0.1960717	-2.350547	0.028409935	0.313125986	down	yes	Probable re	At1g30570
Pavir.2NG386200.v4	GO:000005	NA	nucleolar pr	9.7025081	0.8281595	30.337911	23.363419	0.196097	-2.350361	0.037697321	0.365544935	down	yes	NA	NA
Pavir.7NG091800.v4	GO:000472	NA	calcineurin-	0.737443	0.408509	3.868124	1.974753	0.196128	-2.350132	0.025035233	0.293585776	down	yes	NA	NA
Pavir.2KG326100.v4	GO:000372	03010(Ribo	large subun	69.664116	52.00103	481.15491	139.16765	0.1961321	-2.350103	0.017101362	0.240475103	down	yes	60S ribosor	RPL13B
Pavir.2KG057400.v4	GO:000218	03010(Ribo	ribosomal p	70.556152	54.197224	486.12058	149.86757	0.1961568	-2.349921	0.016106562	0.232779784	down	yes	60S ribosor	SB62
Pavir.9KG566500.v4	GO:004665	NA	plastocyani	13.446632	1.803886	44.663746	33.037121	0.1962722	-2.349072	0.01967672	0.256703501	down	yes	NA	NA
Pavir.9NG308400.v4	GO:000373	03010(Ribo	large subun	27.326069	21.063765	186.65601	59.807842	0.1963364	-2.3486	0.013144119	0.208381427	down	yes	60S ribosor	RPL23A
Pavir.3KG175800.v4	GO:000373	03010(Ribo	small subun	46.121601	25.578272	275.39008	89.792236	0.1963399	-2.348574	0.017741991	0.243825837	down	yes	Ubiquitin-4	MUB1
Pavir.9KG445800.v4	GO:000015	03008(Ribo	nucleolar pr	6.1342352	6.5374155	55.433663	9.0482226	0.1965149	-2.347289	0.022217575	0.27450782	down	yes	Probable nu	NOP5-1;NOP5-1
Pavir.9KG478600.v4	GO:000443	00562(Inos	phosphatidy	1.657602	1.135241	9.90987	4.301185	0.1965261	-2.347207	0.010538329	0.186164311	down	yes	Phosphoino	PLC6
Pavir.7KG319900.v4	GO:000002	03010(Ribo	large subun	28.720842	24.29631	205.66516	64.080376	0.1965451	-2.347068	0.014018154	0.215049498	down	yes	60S ribosor	RPL12B
Pavir.9NG413300.v4	GO:000046	03010(Ribo	40s ribosom	1.062591	2.216919	12.678681	4.006396	0.1965535	-2.347006	0.033040807	0.340400939	down	yes	40S ribosor	RPS24A
Pavir.5KG371400.v4	GO:000374	03013(RNA	translation i	3.356052	2.912325	22.274612	9.583313	0.1967604	-2.345488	0.010998565	0.189563798	down	yes	Eukaryotic t	TIF3F1
Pavir.3KG291700.v4	GO:000218	03010(Ribo	60s ribosom	7.740347	7.674571	61.627361	16.705332	0.1967878	-2.345287	0.010958438	0.189237262	down	yes	60S ribosor	RPL35AA
Pavir.7KG073000.v4	GO:000582	NA	predicted pl	2.2747204	1.539311	14.241732	5.1353673	0.1968319	-2.344964	0.012565389	0.203863881	down	yes	Phosphogly	At5g64460;At5g64460
Pavir.4NG061700.v4	NA	NA	0	8.889236	1.013514	24.225119	25.962681	0.1973139	-2.341436	0.022627468	0.277230089	down	yes	NA	NA
Pavir.2NG438000.v4	GO:000377	NA	formin-relat	0.733173	0.185191	3.261838	1.39123	0.1973674	-2.341044	0.037420732	0.364237061	down	yes	Formin-like	FH15
Pavir.3KG219000.v4	GO:000471	NA	serine-threo	0.223842	0.267877	1.435634	1.054389	0.1974757	-2.340253	0.042420847	0.38846661	down	yes	NA	NA
Pavir.9KG034700.v4	GO:000842	00500(Starc	glycosyl hyc	5.377791	0.837329	22.281235	9.141402	0.1977912	-2.33795	0.02276321	0.278412144	down	yes	Beta-glucos	BGLU8
Pavir.5KG258400.v4	GO:000456	00052(Gala	beta-galactc	1.248582	0.1928	4.048308	3.238437	0.1978088	-2.337822	0.035125196	0.352598474	down	yes	Beta-galact	Os01g0580200
Pavir.1KG415800.v4	NA	NA	0	3.845309	3.124409	30.774929	4.450204	0.1978621	-2.337433	0.020345607	0.261527703	down	yes	NA	NA
Pavir.9NG657300.v4	GO:000582	03010(Ribo	small subun	14.051779	13.598452	104.03854	35.675293	0.1979062	-2.337111	0.012235083	0.200571016	down	yes	40S ribosor	RPS3A
Pavir.2KG403000.v4	GO:000561	03015(mRN	fasciclin do	3.147899	0.715019	13.395494	6.118137	0.19796	-2.336719	0.019949337	0.258492149	down	yes	Fasciclin-lil	FLA1
Pavir.1NG116900.v4	GO:000097	NA	transcriptio	2.230583	1.746673	10.061652	10.004533	0.1982069	-2.334921	0.006586253	0.147204491	down	yes	Protein CY	CGA1
Pavir.3KG358300.v4	GO:000552	00940(Pher	rac-like gtp-	2.264124	0.291045	8.88192	4.007713	0.1982344	-2.334721	0.040181298	0.377716268	down	yes	Rac-like GT	RAC2
Pavir.9KG595500.v4	GO:000588	02010(ABC	atp-binding	0.818271	0.086776	1.799498	2.7660354	0.1982347	-2.334719	0.047961824	0.413034522	down	yes	ABC transp	ABCG22;ABCG22
Pavir.1KG541000.v4	GO:000372	03010(Ribo	large subun	50.904232	44.52832	368.22809	112.85213	0.1983714	-2.333724	0.015829733	0.230978856	down	yes	60S ribosor	RPL37C
Pavir.6KG207000.v4	NA	NA	0	0.575849	0.387694	3.082039	1.774674	0.1983941	-2.333559	0.027041363	0.306076323	down	yes	NA	NA
Pavir.7NG280600.v4	GO:000573	NA	leucine rich	1.989169	0.357723	7.93923	3.88492	0.1984829	-2.332913	0.028081919	0.311429145	down	yes	NA	NA
Pavir.1NG175400.v4	NA	NA	0	1.667036	0.353591	6.428327	3.742573	0.1986675	-2.331572	0.045737165	0.403146483	down	yes	NA	NA
Pavir.5KG361400.v4	GO:000521	NA	protein nrt1	12.938755	4.047782	25.859741	59.582123	0.1988081	-2.330551	0.017273759	0.241363679	down	yes	Protein NR	NPF6.2
Pavir.6KG351600.v4	NA	NA	0	3.341352	0.404748	4.468848	14.364789	0.1989048	-2.32985	0.040147418	0.377598979	down	yes	NA	NA
Pavir.5KG670100.v4	GO:000695	NA	universal st	13.942588	5.6709144	73.279114	25.290307	0.1989816	-2.329293	0.017269759	0.241363679	down	yes	NA	NA

Pavir.1NG376200.v4	GO:000077	00310(Lysi	histone-lysi	0.546869	0.226881	2.256229	1.629892	0.199106	-2.328391	0.020518621	0.262852422	down	yes	Histone-lysi	SUVH5
Pavir.7NG152000.v4	NA	NA	0	10.200059	0.87273	38.284485	17.327751	0.1991071	-2.328384	0.046563116	0.406108626	down	yes	NA	NA
Pavir.6KG013200.v4	GO:000373	03010(Ribo	small subun	27.952932	16.832423	170.05246	54.858317	0.199125	-2.328254	0.017207034	0.241064917	down	yes	40S ribosor	RPS13;RPS13
Pavir.5KG269800.v4	GO:000367	NA	expressed p	0.113528	0.454777	1.227693	1.626275	0.199128	-2.328232	0.040910333	0.380527563	down	yes	NA	NA
Pavir.2NG599500.v4	GO:000367	03040(Splic	myb-related	4.947516	0.626008	16.1523	11.824135	0.1992221	-2.32755	0.022424421	0.276065782	down	yes	Transcriptic	MYB124
Pavir.2KG354700.v4	GO:000563	03013(RNA	apoptosis-p	11.975743	2.168477	50.165829	20.798162	0.1993155	-2.326875	0.026588657	0.303559891	down	yes	Polyadenyla	RBP47
Pavir.2NG124000.v4	GO:000582	03010(Ribo	small subun	47.708534	40.287926	334.11688	106.67139	0.1996343	-2.324568	0.016585875	0.23662268	down	yes	40S ribosor	RPS3A
Pavir.3NG236500.v4	NA	NA	0	1.855464	0.757936	6.775749	6.313197	0.1996647	-2.324349	0.030599417	0.327001057	down	yes	NA	NA
Pavir.7NG279700.v4	GO:000573	04075(Plan	leucine rich	2.893111	0.652995	11.440626	6.271562	0.2002071	-2.320435	0.017554957	0.243072641	down	yes	NA	NA
Pavir.9NG654000.v4	GO:000377	04626(Plan	formin-relat	1.017746	0.438338	4.629074	2.636861	0.2003987	-2.319055	0.015906345	0.231765709	down	yes	Formin-like	FH8;FH8
Pavir.9KG212600.v4	GO:000550	03018(RNA	chaperonin	4.708429	3.282634	34.863625	4.951833	0.2007025	-2.316869	0.021798527	0.271743878	down	yes	Chaperonin	CPN60II
Pavir.2NG423500.v4	GO:000047	03010(Ribo	large subun	27.420281	22.097994	204.40435	42.295396	0.2007228	-2.316723	0.024504003	0.289342841	down	yes	60S ribosor	RPL7A-2;RPL7A-2;RPL7A-2
Pavir.4KG177700.v4	GO:000579	NA	protein trich	1.854494	0.58758	7.559984	4.586852	0.2010461	-2.314402	0.012809765	0.205647752	down	yes	Protein trich	TBR
Pavir.2NG179900.v4	GO:000578	00520(Ami	delta8-fatty-	43.565815	4.40079	129.74901	108.60967	0.2012371	-2.313032	0.039229337	0.37326534	down	yes	Delta(8)-fat	SLD2
Pavir.3KG385200.v4	GO:000416	00900(Terp	farnesyl dip	4.7358201	3.8625375	28.001842	14.717899	0.2012736	-2.31277	0.00549902	0.134252553	down	yes	Farnesyl py	FPS;FPS
Pavir.1KG227700.v4	GO:000550	NA	synaptotagn	0.859751	0.813952	5.543689	2.770459	0.2013078	-2.312525	0.009916679	0.18175579	down	yes	FT-interacti	FTIP3
Pavir.6KG324400.v4	GO:000372	03010(Ribo	large subun	19.151638	15.847166	135.43976	38.413212	0.2013127	-2.31249	0.015377799	0.22717071	down	yes	60S ribosor	RPL30
Pavir.6KG269100.v4	GO:000474	01200(Carb	dihydrolipoa	3.638072	3.800445	27.002535	9.916004	0.2014846	-2.311259	0.008097149	0.163303361	down	yes	Dihydrolipc	LTA2
Pavir.9NG022100.v4	GO:000582	NA	guanine dea	5.16711	2.840413	19.45833	20.270967	0.2015521	-2.310775	0.00730836	0.156441785	down	yes	Guanosine (	GSDA
Pavir.8NG298900.v4	NA	NA	protein of u	0.772033	0.131314	1.943813	2.532392	0.2018109	-2.308924	0.044856549	0.39955616	down	yes	NA	NA
Pavir.2NG087200.v4	GO:000367	NA	domain of u	10.030629	2.186481	26.738689	33.692272	0.2021664	-2.306385	0.013102675	0.208163554	down	yes	NA	NA
Pavir.3NG261900.v4	GO:000588	NA	characteri	0.436942	0.63188	3.432534	1.849649	0.2023448	-2.305113	0.025156337	0.294261858	down	yes	NA	NA
Pavir.9NG273100.v4	GO:000373	03010(Ribo	small subun	11.838941	15.242241	100.06392	33.757408	0.2023682	-2.304945	0.0095789	0.178946539	down	yes	40S ribosor	RPS12
Pavir.3KG296300.v4	GO:000047	03013(RNA	ribosome bi	2.775131	1.653363	18.573835	3.303437	0.2024244	-2.304545	0.026083758	0.300442986	down	yes	NA	NA
Pavir.1NG523400.v4	GO:000575	03060(Prot	20 kda chap	1.3965817	3.6290409	18.229906	6.5946917	0.2024453	-2.304396	0.014328063	0.217778448	down	yes	20 kDa cha	CPN20;CPN20
Pavir.1NG249400.v4	GO:000573	00940(Pher	copper trans	1.302861	1.060079	10.323585	1.339773	0.2025952	-2.303328	0.044714498	0.39887413	down	yes	Heavy meta	HIPP07
Pavir.4KG150000.v4	GO:000384	00561(Glyc	1-acylglycer	3.3751871	1.0413175	14.790679	7.0085464	0.2025992	-2.3033	0.015169434	0.225673911	down	yes	Probable 1-	LPAT5;LPAT5
Pavir.9NG445600.v4	GO:000372	03010(Ribo	large subun	31.101171	26.343304	219.39023	64.06797	0.2026559	-2.302896	0.01004453	0.181875162	down	yes	60S ribosor	RPL30
Pavir.8NG001000.v4	GO:000367	NA	protein of u	3.952344	1.831405	15.084705	13.422153	0.2028897	-2.301232	0.006312268	0.144254761	down	yes	NA	NA
Pavir.5KG696400.v4	GO:000002	03010(Ribo	large subun	75.795532	78.366104	571.56409	188.10117	0.2029336	-2.30092	0.018597802	0.248940219	down	yes	60S ribosor	RPL5A
Pavir.1KG535500.v4	NA	NA	0	5.4199825	4.2943029	34.816311	13.01672	0.2030874	-2.299827	0.008158649	0.164103267	down	yes	NA	NA
Pavir.5KG223100.v4	GO:000478	00730(Thia	thiamin pyro	3.8205182	2.6919884	18.630029	13.429112	0.2031404	-2.299451	0.005938808	0.139206999	down	yes	Thiamine p	TPK2;TPK2;TPK2
Pavir.7NG111300.v4	GO:000046	03010(Ribo	small subun	51.747231	48.290691	362.41821	129.96764	0.2031698	-2.299242	0.016442559	0.235530414	down	yes	40S ribosor	RPS8
Pavir.3NG186000.v4	GO:000563	NA	kelch repeat	2.5632991	1.8580737	13.868508	7.8897192	0.2032046	-2.298995	0.006100348	0.141574035	down	yes	Epithiospec	ESP;ESP
Pavir.1NG242000.v4	GO:000002	03010(Ribo	small subun	26.451393	26.397888	196.90328	63.069023	0.2032881	-2.298402	0.015270377	0.226403915	down	yes	40S ribosor	RPS27
Pavir.6NG308200.v4	GO:002262	03010(Ribo	large subun	10.227168	8.953251	77.056519	16.870718	0.2042051	-2.291909	0.014691525	0.220713578	down	yes	60S ribosor	RPL32A
Pavir.7NG258900.v4	GO:000373	03010(Ribo	small subun	13.153012	17.10173	119.57359	28.406334	0.2044517	-2.290168	0.018648713	0.249532796	down	yes	40S ribosor	RPS11
Pavir.7NG364900.v4	GO:000367	00130(Ubic	homeobox-l	0.997744	0.80782	5.94902	2.880291	0.2044966	-2.289851	0.011126802	0.190811192	down	yes	Homeobox-	ROC2
Pavir.9KG174000.v4	GO:000373	03010(Ribo	small subun	19.006034	15.823825	132.63281	37.677048	0.2045088	-2.289765	0.017008202	0.240244054	down	yes	40S ribosor	RPS28;RPS28
Pavir.1NG250400.v4	GO:000002	03010(Ribo	60s ribosom	41.537712	29.627466	266.91724	80.669304	0.2047409	-2.288129	0.018763392	0.250354018	down	yes	60S ribosor	RPL3
Pavir.4KG272000.v4	GO:000551	NA	t-complex p	2.565687	2.331677	18.586176	5.326973	0.204798	-2.287727	0.012432352	0.202479745	down	yes	T-complex	-
Pavir.1NG220100.v4	GO:000218	03010(Ribo	large subun	25.717548	27.916264	198.90465	62.953186	0.2048203	-2.287569	0.017942127	0.244825273	down	yes	60S acidic	RP-P0
Pavir.6KG356600.v4	GO:000372	NA	60s ribosom	1.420162	0.310294	6.847853	1.599304	0.2048566	-2.287314	0.047758116	0.412130382	down	yes	NA	NA
Pavir.9NG461300.v4	NA	NA	0	0.599891	0.611063	2.749077	3.159659	0.204943	-2.286705	0.048609417	0.415847365	down	yes	NA	NA
Pavir.1NG338000.v4	NA	NA	0	2.751139	3.043144	21.752512	6.516195	0.2049716	-2.286504	0.016062497	0.232658514	down	yes	NA	NA
Pavir.9NG322300.v4	GO:000437	00500(Starc	glycosyltran	0.3717394	2.4955182	8.6731589	5.3135086	0.2049993	-2.286309	0.026356472	0.302656394	down	yes	Soluble star	SSII-1;SSII-1
Pavir.1KG252000.v4	NA	NA	0	4.250813	6.548251	40.312946	12.343732	0.2050844	-2.28571	0.011591338	0.195331535	down	yes	NA	NA
Pavir.3KG239200.v4	GO:000435	01230(Bios	glutamine s	3.301797	1.550554	11.461988	12.195118	0.2051118	-2.285518	0.013331761	0.209368875	down	yes	Glutamine s	GLN2
Pavir.2NG395400.v4	GO:000467	00010(Glyc	ribosomal p	7.194701	1.228997	24.884766	16.18042	0.2051299	-2.28539	0.019040519	0.252438046	down	yes	Protein kina	Os06g0291600
Pavir.9NG518300.v4	GO:000002	03010(Ribo	small subun	8.866182	6.67439	57.045757	18.645739	0.2053146	-2.284092	0.009822118	0.180816484	down	yes	40S ribosor	RPS19A
Pavir.1NG354500.v4	GO:000373	03010(Ribo	large subun	18.114063	17.72455	136.43658	38.104733	0.2053303	-2.283982	0.020514872	0.262852422	down	yes	60S ribosor	RPL14A
Pavir.1NG362500.v4	NA	NA	0	1.535647	0.807577	7.8849	3.521719	0.2054267	-2.283304	0.018416355	0.247720805	down	yes	NA	NA
Pavir.5KG326400.v4	GO:000047	03010(Ribo	large subun	30.488617	29.332777	221.23015	69.94001	0.2054517	-2.283129	0.018816218	0.250638862	down	yes	60S ribosor	RPL7A-2
Pavir.7KG395300.v4	GO:000367	00130(Ubic	homeobox-l	0.50304	0.538886	2.946275	2.12453	0.2054755	-2.282962	0.022578179	0.277141434	down	yes	Homeobox-	ROC2
Pavir.5KG117100.v4	GO:000372	NA	60s ribosom	1.332013	0.555862	5.465483	3.715456	0.2056298	-2.281878	0.017790441	0.244134493	down	yes	NA	NA
Pavir.2KG121500.v4	GO:000373	03010(Ribo	small subun	28.413158	26.267742	200.72965	65.128967	0.2056766	-2.28155	0.017559815	0.243072641	down	yes	40S ribosor	RPS2C
Pavir.1KG463600.v4	GO:000002	03010(Ribo	large subun	17.119093	12.312527	110.6098	32.460232	0.2057148	-2.281283	0.016040061	0.23247951	down	yes	60S ribosor	RPL12
Pavir.3NG003000.v4	GO:006148	03010(Ribo	60s ribosom	9.562012	8.72832	72.156029	16.754429	0.2057163	-2.281272	0.01441396	0.218700543	down	yes	60S ribosor	RPL17
Pavir.6NG040100.v4	GO:000373	03010(Ribo	small subun	28.005314	23.322557	189.30264	60.051422	0.2058433	-2.280381	0.016396062	0.235402849	down	yes	40S ribosor	RPS13
Pavir.1KG491500.v4	GO:000467	04075(Plan	protein kina	3.953615	0.991617	13.143408	10.868796	0.2059466	-2.279658	0.020272439	0.261123193	down	yes	Probable re	At2g42960
Pavir.9NG533400.v4	GO:000576	NA	methyltrans	3.58694	2.414268	22.076641	7.033668	0.206154	-2.278205	0.010785635	0.188149509	down	yes	Probable m	At1g33170
Pavir.6NG175500.v4	GO:000373	03010(Ribo	small subun	11.178102	8.192007	74.567101	19.330742	0.2062892	-2.27726	0.01357418	0.211539891	down	yes	40S ribosor	RPS28
Pavir.1NG500300.v4	GO:000563	03030(DNA	proliferating	4.108691	11.264627	68.955727	5.558736	0.2063132	-2.277092	0.048001096	0.413183315	down	yes	Proliferating	PCNA
Pavir.5NG576200.v4	NA	NA	0	3.7054429	0.5463135	10.775911	9.8323372	0.2063133	-2.277091	0.02097637	0.266715793	down	yes	Interactor o	ICR2;ICR2;ICR2
Pavir.9NG169300.v4	GO:000455	00500(Starc	glucan endo	2.42186	0.258348	6.585244	6.402597	0.2063629	-2.276745	0.037121896	0.362973709	down	yes	Glucan endo	At4g34480;At4g34480
Pavir.9NG434200.v4	GO:000467	04626(Plan	protein kina	1.546306	0.183492	4.416972	3.949391	0.2067563	-2.273997	0.041652406	0.384144786	down	yes	Probable se	At1g01540
Pavir.4NG204300.v4	NA	NA	0	13.29908	10.206304	87.896111	25.703327	0.2069146	-2.272893	0.012155793	0.200056765	down	yes	60S ribosor	RPL27C
Pavir.2KG578900.v4	GO:000218	03010(Ribo	large subun	42.234409	41.233582	297.49435	105.31123	0.2072166	-2.270789	0.017892223	0.244744653	down	yes	60S acidic	RP-P0
Pavir.2KG306200.v4	GO:000576	NA	methyltrans	15.024373	6.569486	66.794891	37.243885	0.2075559	-2.268428	0.015757746	0.2303758	down	yes	Probable m	At1g26850
Pavir.3NG006100.v4	GO:006148	03010(Ribo	large subun	16.188528	16.487179	120.68626	36.74028	0.2075616	-2.268388	0.017685275	0.243313374	down	yes	60S ribosor	RPL17
Pavir.6NG351000.v4	GO:000218	03010(Ribo	large subun	31.047234	20.336391	193.43579	53.850021	0.2077904	-2.266799	0.019827032	0.258039853	down	yes	60S ribosor	RPL31
Pavir.9KG363500.v4	GO:000002	03010(Ribo	small subun	25.807894	22.538937	162.61202	70.049034	0.2077994	-2.266736	0.015197677	0.225968469	down	yes	40S ribosor	GSVIVT00034021001
Pavir.5NG256200.v4	NA	NA	rapsyn-relat	3.371409	2.8952032	17.821955	12.31874	0.207912	-2.265955	0.005720772	0.136128289	down	yes	Protein FLU	FLU;FLU;FLU;FLU;FLU
Pavir.9NG587300.v4	GO:000218	03010(Ribo	large subun	41.011135	31.442097	252.98967	94.626503	0.2084288	-2.262373	0.017330007	0.24174101	down	yes	60S ribosor	RPL22B
Pavir.3KG081600.v4	GO:000467	04626(Plan	non-specific	11.7525	4.294885	48.93639	28.040651	0.2084698	-2.26209	0.017207466	0.241064917	down	yes	Proline-rich	PERK1;PERK1
Pavir.6KG284200.v4	NA	NA	0	2.675078	0.733218	11.483439	4.861509	0.2085229	-2.261722	0.016617766	0.236776381	down	yes	NA	NA
Pavir.6NG145100.v4	GO:000955	NA	burp domai	1.957145	2.046794	11.745732	7.449482	0.2085905	-2.261255	0.006811114	0.150570354	down	yes	BURP dom	BURP9
Pavir.1NG520500.v4	GO:000218	03010(Ribo	large subun	22.368645	23.11907	168.46738	49.556023	0.2086368	-2.260934	0.017696406	0.243377397	down	yes	60S ribosor	RPL35AA
Pavir.2NG052500.v4	GO:000582	03010(Ribo	small subun	40.209339	26.56971	235.6852	83.882706	0.2089667	-2.258655	0.018379826	0.247552699	down	yes	40S ribosor	RPS18A
Pavir.1NG413400.v4	GO:000521	NA	nucleobase-	12.026927	3.601183	45.479782	29.269419	0.2090739	-2.257915	0.018036121	0.245284518	down	yes	Nucleobase	NAT6
Pavir.4NG153400.v4	GO:000864	NA	gb	0.678825	0.472024	4.101054	1.402857	0.2090966	-2.257759	0.03960025	0.375463894	down	yes	Nucleotide-	At5g55950
Pavir.2KG388900.v4	GO:000419	03013(RNA	ribonucleas	1.820374	2.689109	18.243181	3.322004	0.2091094	-2.25767	0.029801267	0.322471544	down	yes	NA	NA

Pavir.2NG481400.v4	GO:000404	00330(Argi	amidase//su	4.268718	1.096209	16.062056	9.59006	0.2091417	-2.257447	0.014596983	0.219883759	down	yes	Probable an	At4g34880
Pavir.3KG309100.v4	GO:000367	NA	endosulfine/	8.9242208	1.5517158	31.40222	18.684273	0.2091569	-2.257342	0.023484164	0.281907504	down	yes	NA	NA
Pavir.6KG144800.v4	GO:000373	03010(Ribo	small subun	14.848841	12.258089	100.843	28.656542	0.2093207	-2.256213	0.013121839	0.208366623	down	yes	40S ribosor	RPS28
Pavir.1KG233400.v4	GO:000372	03010(Ribo	small subun	52.402935	47.320114	356.48993	119.88976	0.2093352	-2.256113	0.019614578	0.256267806	down	yes	40S ribosor	RPS26
Pavir.7KG054200.v4	GO:000981	NA	sterol 3-beta	5.3945848	3.3340196	13.97632	27.695027	0.209463	-2.255233	0.007926145	0.162028857	down	yes	Sterol 3-bet	UGT80A2;UGT80A2
Pavir.1NG309000.v4	GO:000563	03013(RNA	apoptosis-p	6.869647	6.132508	45.753117	16.311666	0.2094933	-2.255024	0.009728754	0.180068207	down	yes	Polyadenyla	RBP47
Pavir.5KG429900.v4	GO:000388	00240(Pyri	ctp synthase	2.3999711	9.2487363	40.675302	14.902135	0.2095942	-2.254329	0.02696442	0.30605746	down	yes	NA	NA
Pavir.8KG263500.v4	GO:000552	04626(Plan	leucine-rich	1.133333	0.582245	2.885691	5.296277	0.2096779	-2.253753	0.042579398	0.389038382	down	yes	Putative dis	At1g50180
Pavir.3NG084200.v4	GO:000563	NA	protein of ur	1.232198	1.152073	8.604897	2.75078	0.2099629	-2.251794	0.017408279	0.24225232	down	yes	Protein ROI	REL2
Pavir.1NG110700.v4	GO:000373	04146(Pero	mitochondri	0.9422875	0.4317081	3.5074433	3.0326622	0.2100877	-2.250936	0.015457368	0.227874188	down	yes	Calcium-de	APC2;APC2;APC2;APC2;APC2;APC2
Pavir.2NG290600.v4	GO:000407	01210(2-O	aspartate ki	0.779025	0.7358379	5.6336248	1.5756039	0.2101283	-2.250658	0.031809759	0.333940095	down	yes	Aspartokina	AK1;AK1;AK1;AK1;AK1
Pavir.1KG482900.v4	GO:000372	03010(Ribo	large subun	5.268921	4.118327	34.384407	10.250089	0.2103137	-2.249385	0.012659753	0.204359507	down	yes	60S ribosor	RPL39
Pavir.8KG055800.v4	GO:000333	04075(Plan	auxin transp	1.30946	2.49404	5.721778	12.357247	0.2103819	-2.248917	0.01241603	0.202389361	down	yes	Putative au	Os11g0169200
Pavir.6NG334900.v4	GO:000482	00970(Ami	glycyl-trna s	1.532525	3.565896	21.700342	2.504796	0.2106338	-2.247191	0.038632658	0.370386463	down	yes	Glycine--tR	At1g29880
Pavir.9NG786900.v4	NA	NA	0	1.472581	2.155279	12.32399	4.893526	0.2107075	-2.246686	0.017839898	0.244539759	down	yes	PLASTID T	PTAC6
Pavir.3KG294600.v4	GO:000047	03013(RNA	ribosome bi	2.1841717	2.064134	16.716487	3.4243595	0.2109298	-2.245165	0.019962237	0.258492149	down	yes	NA	NA
Pavir.1KG265700.v4	GO:000046	03010(Ribo	small subun	53.306456	40.349012	325.59593	117.90739	0.211172	-2.24351	0.020125962	0.259860063	down	yes	40S ribosor	RPS8;RPS8;RPS8;RPS8
Pavir.1KG004700.v4	GO:000218	03010(Ribo	large subun	14.685041	18.087263	122.2917	32.791435	0.2113209	-2.242493	0.021128538	0.267383818	down	yes	60S ribosor	RPL9
Pavir.6KG065200.v4	GO:000006	NA	diazepam-b	62.696091	13.936735	232.60556	129.55495	0.2115991	-2.240595	0.028046693	0.311277434	down	yes	Acyl-CoA-b	ACBP3
Pavir.1NG215300.v4	GO:000046	03010(Ribo	large subun	7.989815	6.191122	52.803307	14.163239	0.2117615	-2.239488	0.021549575	0.270230878	down	yes	60S ribosor	RPL35B
Pavir.7NG411100.v4	GO:000046	03010(Ribo	large subun	13.294575	12.851896	91.751985	31.672614	0.2118416	-2.238942	0.016422286	0.235419518	down	yes	60S ribosor	RPL7C;RPL7C
Pavir.1NG450900.v4	GO:000573	00053(Asco	disulfide ox	1.514367	1.122989	2.746178	9.698181	0.2119318	-2.238328	0.020337282	0.261527703	down	yes	Monodehyd	MDAR1
Pavir.4KG116200.v4	GO:000022	00190(Oxic	protein regu	4.737304	4.631896	33.671078	10.525272	0.2119904	-2.237929	0.017919194	0.24480284	down	yes	65-kDa mic	MAP65-1
Pavir.2NG444000.v4	GO:000398	00650(Buta	hydroxymet	11.99378	6.998891	64.64875	24.920288	0.212045	-2.237557	0.017075412	0.240475103	down	yes	Hydroxyme	HMGS
Pavir.6KG323100.v4	GO:000016	03040(Splic	rna recognit	13.345575	12.37498	102.87832	18.358372	0.2121516	-2.236833	0.021964003	0.272986098	down	yes	Glycine-ricl	RBG2;RBG2
Pavir.4NG229900.v4	GO:000471	04075(Plan	protein kina	2.211333	3.365465	16.996752	9.289511	0.2121564	-2.2368	0.009702254	0.180021127	down	yes	Serine/threo	STY13
Pavir.3KG204500.v4	GO:000577	NA	hgg motif-co	0.704761	0.989084	5.162089	2.814	0.2123654	-2.23538	0.035404757	0.354053501	down	yes	1,4-dihydro	DHNAT1
Pavir.7NG273600.v4	GO:000551	01200(Carb	malate dehy	1.372948	0.407557	2.741528	5.635659	0.2125421	-2.234179	0.032090434	0.335485217	down	yes	Malate dehy	Os10g0478200
Pavir.4KG411500.v4	NA	NA	0	4.404777	0.85395	10.116966	14.62096	0.2125775	-2.233939	0.021562148	0.270230878	down	yes	NA	NA
Pavir.5NG125700.v4	GO:000372	NA	60s ribosom	0.9325377	0.1862687	3.620077	1.6409621	0.2126588	-2.233387	0.048939267	0.416966544	down	yes	NA	NA
Pavir.5KG045400.v4	GO:000471	04075(Plan	serine-threo	2.3128042	0.8162791	6.5442877	8.1655805	0.21272	-2.232972	0.01154412	0.194885026	down	yes	Serine/threo	HT1;HT1
Pavir.9KG440500.v4	GO:000044	03010(Ribo	small subun	34.183422	25.518623	206.53267	74.087418	0.2127504	-2.232766	0.017425548	0.242313137	down	yes	40S ribosor	RPS21
Pavir.2NG315600.v4	GO:000576	NA	methyltrans	7.088853	5.04109	42.723255	14.290734	0.2127538	-2.232743	0.017664631	0.243207467	down	yes	Probable m	At1g26850
Pavir.4KG094300.v4	GO:000218	03010(Ribo	large subun	30.13851	22.995523	190.70967	58.919762	0.2128516	-2.23208	0.019690189	0.256703501	down	yes	60S ribosor	RPL35AA
Pavir.5NG406500.v4	NA	NA	0	1.2447585	0.2066812	4.3149729	2.4889472	0.213324	-2.228882	0.042661412	0.389038382	down	yes	Protein CHU	CHUP1;CHUP1
Pavir.5KG689800.v4	GO:000582	03010(Ribo	small subun	25.42378	20.187012	156.02075	57.787292	0.2133259	-2.228869	0.017182151	0.241064917	down	yes	40S ribosor	RPS18A
Pavir.9KG575300.v4	GO:000373	03010(Ribo	large subun	41.753357	32.698227	261.30197	87.363907	0.2135328	-2.227471	0.019943033	0.258492149	down	yes	60S ribosor	RPL21E
Pavir.9NG131700.v4	GO:000467	04075(Plan	inactive rec	1.693677	1.150911	11.644986	1.665741	0.2137064	-2.226298	0.033563189	0.343522115	down	yes	Probable in	RLK902
Pavir.5KG619200.v4	GO:000373	03010(Ribo	large subun	2.187819	2.721632	17.439291	5.525446	0.2137822	-2.225787	0.028793526	0.315732906	down	yes	NA	NA
Pavir.6NG179000.v4	NA	NA	0	4.330344	2.57622	26.211113	6.088579	0.2138275	-2.22548	0.035411946	0.354053501	down	yes	NA	NA
Pavir.2KG394600.v4	GO:000576	00561(Glyc	gdp-fucose	1.347311	0.291677	5.1841	2.476312	0.2139556	-2.224617	0.045772839	0.403146483	down	yes	O-fucosyltra	OFUT6
Pavir.5KG280600.v4	GO:000002	03010(Ribo	small subun	38.424732	31.947647	238.07529	90.283913	0.2143152	-2.222194	0.017923845	0.24480284	down	yes	40S ribosor	RPS17
Pavir.7NG253600.v4	GO:000016	NA	lupus la pro	0.876831	2.016701	11.401404	2.091221	0.2144529	-2.221268	0.043154257	0.391745566	down	yes	La protein 1	LA1
Pavir.1NG133600.v4	GO:000014	00500(Starc	callose synt	0.3274078	0.1259317	1.1080224	1.0036677	0.2146809	-2.219734	0.03485079	0.351058637	down	yes	Putative cal	CALS8;CALS8;CALS8;CALS8
Pavir.4KG120300.v4	GO:000367	NA	complement	2.88134	3.115092	23.084192	4.838459	0.2147515	-2.21926	0.022067115	0.273820243	down	yes	Uncharacter	At2g39795
Pavir.8NG153800.v4	GO:000373	03010(Ribo	large subun	4.433537	4.063433	29.986629	9.575134	0.2147773	-2.219086	0.013201676	0.208853297	down	yes	60S ribosor	RPL18A;RPL18A;RPL18A
Pavir.5KG035100.v4	GO:000002	03010(Ribo	large subun	24.362888	18.639893	155.18906	44.483898	0.2153661	-2.215137	0.023179177	0.281068051	down	yes	60S ribosor	RPL11
Pavir.2NG455400.v4	GO:000419	03013(RNA	ribonuclease	0.77006	0.85463	6.048764	1.492151	0.21545	-2.214575	0.049650984	0.420924282	down	yes	NA	NA
Pavir.6KG034800.v4	GO:000367	NA	protein of ur	8.007545	8.10379	41.379379	33.348106	0.2156012	-2.213563	0.00644975	0.145810141	down	yes	NA	NA
Pavir.2NG636700.v4	GO:000370	04141(Prot	bzip transcr	4.128313	0.65388	14.986298	7.170577	0.2158334	-2.21201	0.034789227	0.350720584	down	yes	Transcriptic	RF2b
Pavir.4NG074800.v4	GO:000460	NA	annexin//sul	1.072874	0.872691	5.051122	3.956387	0.2159937	-2.210939	0.027783769	0.309768342	down	yes	Annexin D1	ANN1
Pavir.6NG329500.v4	GO:000047	03010(Ribo	large subun	28.503336	29.207838	203.19151	63.890087	0.2160807	-2.210358	0.022232363	0.27450782	down	yes	60S ribosor	RPL7A-2
Pavir.1NG381900.v4	GO:000588	NA	aquaporin ti	10.016808	1.959886	15.133255	40.261829	0.216205	-2.209528	0.025745994	0.297739072	down	yes	Aquaporin	TIP2-1
Pavir.2NG463000.v4	GO:000433	00520(Ami	l-arabinokir	1.159837	0.974241	5.900671	3.969225	0.2162209	-2.209422	0.009224361	0.17522089	down	yes	L-arabinoki	ARA1
Pavir.2KG550900.v4	GO:000551	NA	protein of ur	2.580165	0.716942	11.755823	3.491067	0.2162478	-2.209242	0.04147824	0.383291177	down	yes	NA	NA
Pavir.6NG066000.v4	GO:000006	NA	diazepam-b	27.051683	4.6317398	98.526322	47.894945	0.2163854	-2.208325	0.035135783	0.352605972	down	yes	Acyl-CoA-b	ACBP3;ACBP3
Pavir.9NG761500.v4	GO:000373	03010(Ribo	large subun	44.015648	30.616846	258.16531	86.37542	0.2166144	-2.206799	0.021329921	0.268845733	down	yes	60S ribosor	RPL21E
Pavir.4KG228400.v4	GO:000373	03010(Ribo	small subun	38.125946	28.843302	241.42204	67.472099	0.2168032	-2.205542	0.0229386	0.279744358	down	yes	40S ribosor	RPS30A
Pavir.3NG125400.v4	NA	NA	0	0.560091	0.628222	4.006732	1.473115	0.2168515	-2.205221	0.038806281	0.371293201	down	yes	NA	NA
Pavir.9KG017200.v4	GO:000374	03013(RNA	elongation f	2.291733	2.032082	15.13002	4.808089	0.2168618	-2.205152	0.016717798	0.237660924	down	yes	Elongation	TUFA
Pavir.5KG510900.v4	GO:000218	03010(Ribo	large subun	56.149983	52.724031	399.58014	102.08824	0.2170239	-2.204074	0.027037988	0.306076323	down	yes	60S ribosor	RPL36C;RPL36C
Pavir.2KG104700.v4	GO:000467	NA	thaumatin fa	0.729201	2.318088	5.06802	8.970766	0.2170621	-2.20382	0.040751545	0.380028688	down	yes	Thaumatin-	RASTL-3
Pavir.5NG234700.v4	GO:000367	03420(Nucl	swi/snf-rela	0.7636602	0.7057283	5.3726386	1.3949477	0.2171215	-2.203425	0.024370649	0.288402642	down	yes	Probable ch	Os01g0367900;Os01g0367900
Pavir.9KG419100.v4	GO:000367	NA	transcriptio	8.084666	0.704212	28.416903	12.048969	0.2171924	-2.202955	0.04943182	0.419634379	down	yes	Transcriptic	BHLH148
Pavir.9NG348900.v4	GO:000563	NA	protein of ur	0.561251	0.629674	4.024042	1.455858	0.217326	-2.202067	0.03866192	0.370386463	down	yes	Protein ROI	REL2
Pavir.9NG046200.v4	GO:001602	00903(Lim	isoflavone 2	1.285489	0.485886	4.836416	3.293503	0.2178835	-2.198371	0.027025531	0.306076323	down	yes	Cytochrome	CYP81Q32
Pavir.8KG200100.v4	GO:000465	NA	burp domai	4.983373	2.014797	17.802618	14.296075	0.2180204	-2.197465	0.01077886	0.188149509	down	yes	BURP dom	BURP12
Pavir.5KG006600.v4	GO:000367	04075(Plan	auxin respo	0.745171	0.885843	5.775564	1.703777	0.2180692	-2.197142	0.027981057	0.310769211	down	yes	Auxin respo	ARF1
Pavir.3KG302000.v4	NA	NA	0	0.950575	0.841755	5.586688	2.631778	0.2180857	-2.197033	0.023942222	0.285708255	down	yes	NA	NA
Pavir.2KG254500.v4	GO:000218	03010(Ribo	large subun	30.69519	28.165054	209.15945	60.601414	0.2181942	-2.196316	0.022032785	0.273574962	down	yes	60S ribosor	RPL35AA
Pavir.2NG585500.v4	GO:000373	03010(Ribo	large subun	38.577976	36.772087	281.96048	63.353054	0.2182077	-2.196226	0.028463212	0.313437359	down	yes	60S ribosor	RPL27AC
Pavir.5NG488200.v4	GO:000573	NA	had superfa	0.848971	0.678013	5.601269	1.387292	0.2184976	-2.19431	0.042767161	0.389643678	down	yes	Endoribonu	YBEY
Pavir.9KG471300.v4	GO:000550	01230(Bios	ll-diaminop	0.5428602	0.6968017	4.0632497	1.6067996	0.2186334	-2.193415	0.036613175	0.359804611	down	yes	Probable LI	AGD2;AGD2;AGD2
Pavir.3KG284600.v4	GO:000373	03010(Ribo	large subun	45.213497	32.25482	270.8116	83.204246	0.2188273	-2.192136	0.025548924	0.296006694	down	yes	60S ribosor	RPL28A;RPL28A
Pavir.4NG293800.v4	GO:001602	00903(Lim	cytochrome	2.893962	0.335704	8.107389	6.648542	0.2188724	-2.191838	0.040925981	0.380527563	down	yes	Cytochrome	CYP77A3
Pavir.4KG263400.v4	GO:000002	03010(Ribo	large subun	13.493876	10.884769	86.290482	25.005089	0.2190442	-2.190706	0.01748247	0.242738038	down	yes	60S ribosor	RPL11
Pavir.6KG373900.v4	GO:000382	00500(Starc	udp-glucuro	0.426126	0.503964	2.86274	1.382841	0.2190725	-2.19052	0.032932829	0.340006925	down	yes	UDP-glucur	GAE6
Pavir.9NG076200.v4	GO:000367	NA	protein of ur	5.146023	0.878012	14.630894	12.848917	0.2192168	-2.18957	0.023223945	0.281238111	down	yes	NA	NA
Pavir.3NG225900.v4	GO:000373	03010(Ribo	small subun	57.150215	46.444454	378.67563	93.838173	0.2192416	-2.189407	0.027917191	0.310335091	down	yes	40S ribosor	RPS29
Pavir.4NG004100.v4	NA	NA	0	32.9851	22.787928	200.49028	53.817577	0.219313	-2.188937	0.020359224	0.261613244	down	yes	NA	NA
Pavir.9KG336300.v4	GO:000367	NA	bzip-like pr	1.950989	1.064934	9.1745	4.573151	0.2193773	-2.188514	0.016714161	0.237660924	down	yes	Protein ROI	REL2
Pavir.9KG337600.v4	GO:002262	03010(Ribo	large subun	38.491211	28.634098	231.92865	73.86808	0.2195096	-2.187644	0.023249162	0.281238111	down	yes	60S ribosor	RPL44

Pavir.5KG696900.v	GO:000372	03010(Ribc	large subun	54.298279	40.103745	335.44278	94.283905	0.2196792	-2.18653	0.027672967	0.309361931	down	yes	NA	NA
Pavir.2NG085200.v	GO:000372	NA	protein rrp5	1.078724	0.786273	6.973503	1.512665	0.219769	-2.18594	0.022525485	0.276856408	down	yes	rRNA biog	RRP5
Pavir.1NG475700.v	GO:004543	00941(Flav	flavonol syr	0.455742	0.89321	4.030971	2.106545	0.2197879	-2.185816	0.044026394	0.396121577	down	yes	Flavonol sy	FLS
Pavir.6KG242200.v	GO:000573	NA	rrna-process	2.8522758	3.8168714	24.067729	6.270021	0.21983	-2.18554	0.020699716	0.264299902	down	yes	Probable rR	EBP2;EBP2
Pavir.6KG355700.v	NA	NA	0	1.492865	0.576191	4.110244	5.281174	0.2203135	-2.18237	0.033300237	0.34213558	down	yes	NA	NA
Pavir.2KG115000.v	GO:000373	NA	small subun	0.955905	1.744906	10.11145	2.144391	0.2203693	-2.182005	0.040039058	0.377133998	down	yes	NA	NA
Pavir.3NG318500.v	GO:000373	03010(Ribc	large subun	0.872621	0.937549	6.264777	1.932842	0.2208166	-2.17908	0.048324192	0.414226418	down	yes	50S ribosor	RPL27
Pavir.9NG584300.v	GO:000044	03010(Ribc	small subun	28.622616	27.430916	184.17944	69.59053	0.2208832	-2.178644	0.018686736	0.249863605	down	yes	40S ribosor	RPS21
Pavir.9NG190200.v	GO:000551	NA	family of un	0.603867	0.412279	1.188578	3.410661	0.2209379	-2.178287	0.041028602	0.381104491	down	yes	AUGMIN s	44051
Pavir.4NG069500.v	GO:000431	01212(Fatty	3-oxoacyl- (	4.836444	1.944226	17.896006	12.772975	0.2210921	-2.17728	0.011912842	0.197698252	down	yes	3-oxoacyl-[	KAS12
Pavir.5KG627900.v	GO:000508	NA	rop guanine	1.422767	0.59939	6.569437	2.571152	0.2212283	-2.176392	0.036109827	0.357134649	down	yes	Rop guanin	ROPGEF7
Pavir.4KG215700.v	GO:000373	03010(Ribc	small subun	9.569373	5.403465	52.245388	15.389066	0.2213789	-2.175411	0.017123309	0.240690464	down	yes	40S ribosor	RPS15AA
Pavir.6KG260200.v	GO:000165	04075(Plan	protein kina	0.41475	0.223759	1.51202	1.369813	0.2215635	-2.174208	0.036593351	0.359804611	down	yes	Leucine-ricl	PEPR1
Pavir.2NG059800.v	GO:000002	03010(Ribc	small subun	50.152584	47.32074	336.71826	103.12587	0.2216088	-2.173913	0.025528339	0.296006694	down	yes	40S ribosor	RPS15
Pavir.2NG618800.v	GO:000218	03010(Ribc	60s acidic r	12.591913	14.765435	93.816093	29.612997	0.2216442	-2.173682	0.01565983	0.229569567	down	yes	60S acidic r	RP-P0
Pavir.3KG172300.v	GO:000372	03010(Ribc	large subun	78.715017	52.616769	447.44306	144.24242	0.2219622	-2.171614	0.026578021	0.303559891	down	yes	60S ribosor	RPL18B;RPL18B
Pavir.3KG289000.v	GO:000551	NA	guanine nuc	67.082245	61.489529	417.24561	161.80441	0.2220392	-2.171114	0.022975925	0.280011167	down	yes	Guanine nu	RACK1A
Pavir.9NG212000.v	GO:000367	NA	protein of u	0.3791361	0.366206	2.3115191	1.044294	0.2221048	-2.170688	0.0491236	0.417944951	down	yes	NA	NA
Pavir.9KG220300.v	GO:000435	NA	type-1 gluta	4.7598327	1.3596503	8.9294653	18.575939	0.2224829	-2.168233	0.018529273	0.248488881	down	yes	NA	NA
Pavir.3KG252500.v	GO:000442	00900(Terp	hydroxymet	3.303157	1.045829	10.802571	8.718424	0.2227851	-2.166276	0.019303574	0.254756997	down	yes	3-hydroxy-3	HMGR
Pavir.9NG594600.v	GO:000015	03008(Ribc	protein nol-	3.459964	4.98506	34.464821	3.424994	0.2228837	-2.165637	0.044747992	0.398922365	down	yes	Probable nu	NOP5-2
Pavir.7NG112300.v	NA	NA	0	4.622543	5.288729	36.667912	7.773773	0.2230175	-2.164771	0.028356629	0.312948164	down	yes	NA	NA
Pavir.7NG392300.v	GO:000467	00520(Ami	interleukin-	0.882167	0.431457	3.344601	2.54182	0.2231617	-2.163838	0.033009998	0.340311982	down	yes	Probable LI	At1g56140
Pavir.9KG404300.v	NA	NA	0	38.277267	10.200873	131.93543	85.205643	0.2232564	-2.163226	0.026293092	0.302298145	down	yes	NA	NA
Pavir.5NG286900.v	GO:000373	03010(Ribc	small subun	48.032734	29.682713	251.82509	96.232155	0.2232835	-2.163051	0.023431267	0.281789387	down	yes	40S ribosor	RPS25B
Pavir.2KG569100.v	GO:000367	NA	centromere	0.839868	0.521696	5.042626	1.053613	0.2233449	-2.162655	0.044404885	0.398226279	down	yes	NA	NA
Pavir.1NG520200.v	GO:000575	03060(Prot	20 kda chap	6.8319782	4.53042	37.303485	13.499653	0.2236554	-2.16065	0.015522809	0.228452218	down	yes	20 kDa cha	CPN20;CPN20
Pavir.9KG069500.v	GO:000455	00500(Starc	glucan endo	2.940171	0.51672	7.561354	7.890287	0.2237232	-2.160213	0.04400138	0.396121577	down	yes	Glucan end	At4g34480
Pavir.2KG203200.v	GO:000587	NA	kinesin mot	3.067286	0.726635	7.627876	9.31151	0.2239704	-2.15862	0.025165969	0.294261858	down	yes	Kinesin-lik	KIN5A
Pavir.2NG378600.v	GO:004278	03013(RNA	ribonucleas	0.52662	0.537787	3.32914	1.419853	0.2241332	-2.157572	0.049710786	0.421146189	down	yes	tRNase Z T	TRZ2
Pavir.6KG398000.v	GO:000563	NA	hras-like su	10.649663	4.284798	44.094448	22.449503	0.22443	-2.155662	0.015288006	0.226415552	down	yes	NA	NA
Pavir.8KG282300.v	GO:000367	NA	plant protei	2.127456	0.743827	7.382115	5.4033	0.2245749	-2.154732	0.021669274	0.27085151	down	yes	UPF0481 p	At3g47200
Pavir.9NG652400.v	GO:000373	03010(Ribc	small subun	20.908051	18.613729	141.39703	34.587425	0.2245754	-2.154728	0.019302747	0.254756997	down	yes	40S ribosor	RPS29
Pavir.1KG267000.v	GO:000557	04141(Prot	translocon-a	17.003414	15.392384	111.7343	32.095025	0.2252378	-2.150479	0.025941993	0.299360724	down	yes	Translocon-	At2g21160;At2g21160
Pavir.5NG582700.v	GO:000480	01200(Carb	triosephospl	21.797125	4.410155	67.907875	48.386715	0.2253525	-2.149744	0.03240738	0.337297085	down	yes	Triosephos	-
Pavir.8NG262100.v	GO:000573	04626(Plan	disease resi	0.545393	0.246516	1.675447	1.8379452	0.2253973	-2.149458	0.027866993	0.310144113	down	yes	Retrovirus-	RE2;RE2
Pavir.6NG137700.v	GO:000474	01200(Carb	dihydrolipo	6.25518	4.278198	32.309269	14.379632	0.2256078	-2.148111	0.012004687	0.19861387	down	yes	Dihydrolipo	LTA2
Pavir.9NG196800.v	NA	NA	0	4.16989	2.680659	24.236376	6.119476	0.2256747	-2.147683	0.044975185	0.400154543	down	yes	NA	NA
Pavir.5NG486200.v	GO:000573	NA	had superfa	0.848026	0.717669	5.577706	1.356292	0.2257997	-2.146884	0.045218723	0.400802804	down	yes	Endoribonu	YBEY
Pavir.3KG363500.v	GO:000372	03010(Ribc	large subun	27.019525	21.185146	162.42392	51.0088	0.2258542	-2.146537	0.025918866	0.299185735	down	yes	60S ribosor	RPL38A;RPL38A
Pavir.1KG186700.v	GO:000046	03010(Ribc	small subun	12.205219	14.48106	93.240082	24.89588	0.2258946	-2.146278	0.018504808	0.248403407	down	yes	40S ribosor	RPS24A
Pavir.6NG317600.v	GO:002262	03010(Ribc	large subun	6.083159	6.620086	43.496758	12.731623	0.2259223	-2.146101	0.031762836	0.333918593	down	yes	60S ribosor	RPL32A
Pavir.2NG598400.v	GO:000467	04626(Plan	protein kina	5.47945	2.650925	21.781874	14.199369	0.2259615	-2.145851	0.016856963	0.238736021	down	yes	Probable in	At5g10020
Pavir.9KG443000.v	GO:000551	NA	calmodulin	5.630047	0.921788	20.358829	8.60795	0.2261845	-2.144428	0.038452931	0.369795742	down	yes	NA	NA
Pavir.7KG152300.v	GO:000467	04075(Plan	serine/threo	1.526946	0.758238	7.730587	2.366757	0.2263154	-2.143594	0.045035912	0.400410395	down	yes	Serine/threc	SAPK7
Pavir.5KG124200.v	NA	NA	0	1.4729137	0.9059809	7.5803616	2.9166439	0.226626	-2.141615	0.024451062	0.288990169	down	yes	NA	NA
Pavir.6NG330000.v	GO:000370	NA	zinc finger	3.075223	3.211891	15.770839	11.957135	0.2267426	-2.140872	0.009998327	0.181831401	down	yes	Zinc finger	COL15
Pavir.5NG007500.v	GO:000367	NA	protein abil	0.50236	1.035826	4.760001	2.021884	0.226808	-2.140456	0.048670106	0.416023543	down	yes	Probable pr	Os01g0236400
Pavir.4KG227600.v	NA	NA	0	2.410753	3.365564	21.420076	4.044987	0.226833	-2.140297	0.049895968	0.421858955	down	yes	NA	NA
Pavir.9NG585200.v	NA	NA	protein of u	0.704467	0.822464	4.765653	1.964211	0.2268888	-2.139942	0.040385823	0.378880534	down	yes	NA	NA
Pavir.3NG023200.v	GO:000372	03010(Ribc	large subun	64.738113	48.997807	377.00726	124.1906	0.2269282	-2.139692	0.027596522	0.308994921	down	yes	60S ribosor	RPL13AD
Pavir.4KG132300.v	GO:000467	00010(Glyc	ribosomal p	0.9863815	0.2772246	3.5396619	2.0254771	0.2270574	-2.138871	0.040892962	0.380508779	down	yes	Protein kina	OsI_021818;OsI_021818;OsI_021818;OsI_021818;OsI_021818
Pavir.3NG220700.v	GO:000367	NA	dna binding	1.0553663	1.6477717	8.7443218	3.1548463	0.2271703	-2.138154	0.023431334	0.281789387	down	yes	NA	NA
Pavir.2NG482000.v	GO:000218	03010(Ribc	large subun	23.918495	21.011513	153.12013	44.497173	0.2273587	-2.136958	0.026527886	0.303412787	down	yes	60S ribosor	SB62
Pavir.9KG646900.v	NA	NA	0	12.99703	2.653549	44.521183	24.260946	0.2275384	-2.135818	0.027153892	0.306772055	down	yes	NA	NA
Pavir.9NG054700.v	GO:004665	NA	plastocyanii	5.923093	1.650866	23.2335	9.994776	0.2279372	-2.133292	0.046463305	0.40582121	down	yes	NA	NA
Pavir.1NG461900.v	GO:000373	03410(Base	adp,atp carr	130.18907	31.597393	414.49505	294.56159	0.2281714	-2.13181	0.036337722	0.358264361	down	yes	ADP,ATP c	ANT2;ANT2;ANT2
Pavir.2NG182100.v	GO:000372	03010(Ribc	large subun	19.930489	21.771809	140.93622	41.793015	0.2282191	-2.131509	0.025444897	0.295794776	down	yes	60S ribosor	RPL30
Pavir.6NG370600.v	GO:000047	03010(Ribc	large subun	30.594118	24.691977	175.30031	66.929626	0.2282381	-2.131389	0.024180669	0.286877253	down	yes	60S ribosor	RPL10A
Pavir.2KG059800.v	GO:000582	03010(Ribc	small subun	31.081646	27.945473	189.97028	68.450996	0.2284143	-2.130275	0.023399475	0.281789387	down	yes	40S ribosor	RPS18A
Pavir.9NG283200.v	GO:000372	03010(Ribc	small subun	1.961805	3.110075	17.167667	5.036922	0.2284158	-2.130265	0.042058979	0.386566047	down	yes	40S ribosor	RPS26
Pavir.2NG575200.v	GO:000467	04075(Plan	protein kina	0.527004	0.185076	1.695081	1.421664	0.2284691	-2.129929	0.042844112	0.390061015	down	yes	Probable LI	At1g74360
Pavir.2KG036700.v	GO:000367	NA	transcriptio	3.024906	0.745755	10.634742	5.861291	0.2285799	-2.12923	0.039690317	0.375732999	down	yes	Transcriptic	PCF6
Pavir.1KG032100.v	GO:000573	NA	ppr repeat (	0.51736	0.534817	3.353725	1.242699	0.2289121	-2.127134	0.03212651	0.335613359	down	yes	Pentatricop	At1g71210
Pavir.1NG398800.v	GO:000046	03010(Ribc	40s ribosom	26.471617	21.366673	164.57877	44.36816	0.2289495	-2.126899	0.020567691	0.263211799	down	yes	40S ribosor	RPS8
Pavir.7NG326500.v	GO:000046	03010(Ribc	large subun	14.076775	13.265341	104.41341	14.989919	0.2289896	-2.126646	0.044568988	0.398414767	down	yes	60S ribosor	RPL7D;RPL7D;RPL7D
Pavir.3KG286800.v	GO:000416	00900(Terp	farnesyl dip	5.436965	2.533027	21.633484	13.164947	0.2290331	-2.126372	0.012812961	0.205647752	down	yes	Farnesyl py	FPS
Pavir.7NG405900.v	GO:000002	03010(Ribc	large subun	19.259136	16.73509	115.06364	41.919857	0.2292867	-2.124775	0.022423095	0.276065782	down	yes	60S ribosor	RPL11
Pavir.9NG750800.v	GO:000588	NA	aquaporin ti	448.97366	167.15509	1412.5385	1272.8788	0.229435	-2.123842	0.02774227	0.309489406	down	yes	Probable aq	TIP1-1
Pavir.3NG255100.v	GO:000373	03010(Ribc	large subun	41.106384	41.202953	283.82584	74.868149	0.2294695	-2.123626	0.031590153	0.332754923	down	yes	60S ribosor	RPL27AB
Pavir.9KG527000.v	NA	NA	0	1.498854	3.0475972	16.12696	3.657801	0.2297956	-2.121577	0.045153519	0.400541715	down	yes	NA	NA
Pavir.9KG247800.v	GO:000372	NA	methyltrans	0.875537	1.172931	7.356203	1.544398	0.2301494	-2.119357	0.02846222	0.313437359	down	yes	NA	NA
Pavir.5KG718400.v	GO:000016	03040(Splic	rna recognit	3.620687	6.120127	36.981262	5.313535	0.2303076	-2.118366	0.043674418	0.394750756	down	yes	Glycine-ricl	RBG4
Pavir.3KG375600.v	GO:000372	03010(Ribc	large subun	38.334114	42.941998	268.47504	83.993301	0.2305912	-2.11659	0.020553827	0.263211799	down	yes	60S ribosor	RPL30
Pavir.2KG118300.v	GO:000367	NA	domain of u	16.00712	2.309982	46.780048	32.646988	0.2306155	-2.116439	0.043165287	0.391751047	down	yes	NA	NA
Pavir.9KG438700.v	GO:000218	03010(Ribc	large subun	21.891588	15.065737	120.11382	40.07933	0.2307048	-2.11588	0.024913913	0.292711207	down	yes	60S ribosor	RPL22B
Pavir.8KG308500.v	GO:000373	03010(Ribc	small subun	38.014179	33.280247	231.60075	77.333542	0.2307754	-2.115439	0.027517034	0.308757967	down	yes	40S ribosor	RPS15AA
Pavir.4NG203800.v	GO:000588	02010(ABC	abc transpo	2.11048	0.332856	6.018332	4.568103	0.2307988	-2.115293	0.046037813	0.40453198	down	yes	ABC transp	ABCG8
Pavir.4NG314100.v	GO:000218	03010(Ribc	large subun	17.969067	19.548313	128.29524	34.254116	0.2308061	-2.115247	0.021539724	0.270221052	down	yes	60S acidic r	RPP3A
Pavir.4NG004200.v	GO:000218	03010(Ribc	large subun	24.241632	17.675808	131.20792	50.16708	0.2311093	-2.113353	0.021309408	0.268677308	down	yes	60S ribosor	RPL9
Pavir.1NG362800.v	GO:000002	03010(Ribc	large subun	22.208418	17.893677	131.28702	42.071613	0.2313245	-2.11201	0.017546628	0.243072641	down	yes	60S ribosor	RPL23A
Pavir.5KG302100.v	GO:000014	00500(Starc	lyst-interact	0.525244	0.799183	2.425111	3.298992	0.2313772	-2.111681	0.029281115	0.319819571	down	yes	Callose syn	CALS7

Pavir.1NG168500.v	NA	NA	0	1.28983	1.555323	9.237567	3.039835	0.231739	-2.109427	0.026582943	0.303559891	down	yes	Probable ca	At4g34215
Pavir.3KG544100.v	GO:000565	NA	nucleolar ar	2.734586	3.554266	23.409798	3.696719	0.2320052	-2.107771	0.03633544	0.358264361	down	yes	NA	NA
Pavir.1KG367900.v	GO:00063(	NA	cgr1 family	1.377388	2.083478	11.751535	3.162732	0.2320507	-2.107488	0.046283321	0.405362057	down	yes	NA	NA
Pavir.5NG041400.v	GO:000543	NA	ef-hand calc	11.282471	8.924995	56.279774	30.765987	0.2321476	-2.106886	0.019002842	0.252027449	down	yes	Sodium/calc	NCL1
Pavir.7KG351300.v	GO:000367	NA	remorin, c-te	1.338515	1.14641	7.228234	3.472961	0.23221	-2.106498	0.025168577	0.294261858	down	yes	NA	NA
Pavir.4NG332600.v	GO:000484	00970(Ami	f-box domai	0.2928786	0.6799553	1.9643499	2.2211934	0.2324271	-2.10515	0.041603209	0.383973809	down	yes	Putative F-l	At1g12855;At1g12855;At2g02030;At1g12855;At1g12855;At1g12855;At1g12855;At1g12855
Pavir.8KG246000.v	GO:000025	00100(Ster	4-alpha-met	5.699271	8.969566	36.813126	26.256586	0.2325813	-2.104193	0.01091188	0.188905631	down	yes	Very-long-c	GL1-11
Pavir.2NG319300.v	GO:000372	03010(Ribo	large subun	34.290226	24.852221	206.30658	47.979412	0.2325824	-2.104186	0.034394243	0.3482336	down	yes	60S ribosor	RPL34
Pavir.3KG353100.v	GO:000002	03010(Ribo	small subun	11.82358	6.655549	60.794785	18.631971	0.2326562	-2.103728	0.020613623	0.26362003	down	yes	40S ribosor	RPS15
Pavir.6KG358700.v	GO:000478	04146(Pero	superoxide	2.0172052	2.7277845	16.248914	4.1325009	0.2328096	-2.102777	0.027741193	0.309489406	down	yes	Superoxide	Os06g0143000;Os06g0143000;Os06g0143000;Os06g0143000;Os06g0143000
Pavir.3KG430300.v	GO:000373	03010(Ribo	small subun	70.945183	58.97002	405.82126	151.99806	0.2328984	-2.102228	0.028043917	0.311277434	down	yes	40S ribosor	RPS9C
Pavir.7KG156600.v	GO:000002	03010(Ribo	small subun	44.205761	35.899578	259.00983	84.767319	0.2330153	-2.101503	0.030557943	0.326982236	down	yes	40S ribosor	RPS10-2
Pavir.2KG205000.v	NA	NA	arabidopsis	2.050572	1.771654	10.590486	5.786602	0.2333886	-2.099194	0.019465711	0.255789208	down	yes	NA	NA
Pavir.1KG397300.v	GO:00040(	03040(Splic	dead-box at	1.064826	1.791841	9.55117	2.675378	0.2336446	-2.097612	0.032489017	0.337682291	down	yes	DEAD-box	Os02g0636300
Pavir.1NG468500.v	GO:000563	NA	transcriptior	2.60011	0.924984	9.656206	5.430641	0.2336535	-2.097558	0.03212871	0.335613359	down	yes	Transcriptic	BHLH96
Pavir.1NG002300.v	GO:000367	NA	dna-binding	1.873116	1.119624	9.100264	3.702473	0.2337578	-2.096913	0.022630325	0.277230089	down	yes	Trihelix trai	GT-2
Pavir.7NG212600.v	GO:000002	03010(Ribo	small subun	28.896078	14.20876	136.86198	47.415237	0.233913	-2.095956	0.021750237	0.271231881	down	yes	40S ribosor	-
Pavir.6NG316000.v	GO:002262	03010(Ribo	large subun	30.783102	29.35358	209.52058	47.470562	0.2340029	-2.095401	0.035486734	0.3540943	down	yes	60S ribosor	RPL32A
Pavir.2KG343300.v	GO:000588	NA	eama-like tr	9.57825	1.277396	26.556925	19.822672	0.2340608	-2.095045	0.037982363	0.366866669	down	yes	WAT1-rela	At4g30420
Pavir.2KG574900.v	GO:000374	NA	elongation f	44.564324	40.278213	269.99225	92.333999	0.2341606	-2.09443	0.030338201	0.325339676	down	yes	Elongation	Os07g0662500;Os07g0662500
Pavir.3KG003000.v	GO:00037(	04075(Plan	protein scar	3.58473	0.990004	11.420025	8.105807	0.2342914	-2.093624	0.020677934	0.264172729	down	yes	Protein SCA	SCR
Pavir.3KG306700.v	GO:000373	03010(Ribo	large subun	30.138168	34.271446	214.70171	60.200901	0.2342998	-2.093573	0.032840776	0.339554498	down	yes	60S ribosor	RPL18A
Pavir.9NG056400.v	GO:000372	03010(Ribo	large subun	12.50822	6.01399	57.897148	21.135986	0.2343601	-2.093201	0.022440068	0.276105064	down	yes	60S ribosor	RPL13AD
Pavir.8KG064800.v	GO:000563	NA	fha domain	1.5893909	1.698474	7.2756902	6.7520986	0.2343823	-2.093065	0.00979725	0.180535591	down	yes	FHA domai	PS1;PS1;PS1;PS1
Pavir.6KG380700.v	NA	NA	non-specific	3.555403	2.780319	18.413776	8.595283	0.2345777	-2.091862	0.016459206	0.235679016	down	yes	NA	NA
Pavir.1KG357100.v	GO:000002	03010(Ribo	large subun	20.980215	18.792904	130.5426	39.006626	0.2345815	-2.091839	0.028333648	0.312835784	down	yes	60S ribosor	RPL6C
Pavir.8NG306500.v	GO:000455	00500(Starc	beta-1, 3-gl	5.107591	7.464344	32.311714	21.279728	0.2345885	-2.091796	0.011530128	0.194867121	down	yes	Glucan endo	GLC1
Pavir.9NG145100.v	GO:000842	00500(Starc	glycosyl hyc	4.8916941	0.6584373	16.855591	6.7930596	0.2346913	-2.091164	0.047037169	0.408630645	down	yes	Beta-glucos	BGLU8;BGLU8;BGLU8
Pavir.3KG522100.v	GO:000372	03010(Ribo	large subun	12.251334	5.926368	57.166336	20.246702	0.2348145	-2.090407	0.023197297	0.281068051	down	yes	60S ribosor	RPL13AD
Pavir.7NG374400.v	GO:000092	NA	microtubule	2.275302	1.944581	13.745503	4.22295	0.2348495	-2.090191	0.036226656	0.357725139	down	yes	Microtubule	EB1A
Pavir.6KG368800.v	GO:000563	NA	creg1 protei	2.087647	2.512065	15.442945	4.135015	0.2349434	-2.089615	0.030804762	0.328534577	down	yes	NA	NA
Pavir.1KG219800.v	GO:000002	03010(Ribo	small subun	16.527548	17.140787	110.22974	32.910103	0.2352129	-2.087961	0.023002368	0.280151687	down	yes	40S ribosor	RPS27
Pavir.1KG517400.v	GO:00041(	00900(Terp	diphosphom	12.110315	8.408118	45.566204	41.646557	0.2352687	-2.087619	0.017016859	0.240275998	down	yes	Diphosphor	MVD2
Pavir.3KG067200.v	GO:000573	NA	2-alkenal re	8.009922	3.051373	26.037823	20.957737	0.2353689	-2.087004	0.017760572	0.24399195	down	yes	2-alkenal re	DBR
Pavir.6KG209900.v	GO:000014	00500(Starc	lyst-interact	3.724903	1.999118	17.818686	6.483191	0.2355382	-2.085967	0.028134631	0.311737691	down	yes	Callose syn	CALS3
Pavir.5NG423700.v	GO:000373	03010(Ribo	large subun	104.28201	70.34462	543.43567	196.79985	0.2359069	-2.083711	0.031845251	0.334037299	down	yes	60S ribosor	RPL18A
Pavir.7KG088200.v	GO:00001(	03040(Splic	rna recognit	5.826288	4.513425	35.983158	7.837174	0.235957	-2.083404	0.048804281	0.416471324	down	yes	Glycine-ricl	RBG2
Pavir.2NG053900.v	GO:000218	03010(Ribo	large subun	21.138041	24.729588	145.97325	48.329826	0.2360623	-2.082761	0.026972039	0.30605746	down	yes	60S acidic r	RPP2A
Pavir.5KG459600.v	GO:000465	00040(Pent	pectate lyas	16.85437	5.816957	39.187111	56.786125	0.2362255	-2.081763	0.026647557	0.30386302	down	yes	Probable pc	GSVIVT00026920001
Pavir.5KG421800.v	GO:000467	04626(Plan	non-specific	4.3585108	1.5681211	10.099574	14.989161	0.2362268	-2.081755	0.017945035	0.244825273	down	yes	Probable re	At2g42960;At2g42960
Pavir.6KG362900.v	GO:000442	00900(Terp	hydroxymet	16.793207	6.501007	67.799576	30.670158	0.2365622	-2.079709	0.031188964	0.330195252	down	yes	3-hydroxy-3	HMGR
Pavir.5NG582200.v	GO:000588	NA	fasciclin do	4.3777421	1.4514986	13.262219	11.375774	0.2365956	-2.079505	0.017856893	0.244599717	down	yes	Fasciclin-lil	FLA4;FLA4
Pavir.3KG326100.v	GO:000472	NA	calcineurin	1.1456636	1.6946329	7.7647492	4.237317	0.2366506	-2.079169	0.021415784	0.269385754	down	yes	Calcineurin	CBL4;CBL4;CBL4;CBL4;CBL4
Pavir.1KG284600.v	GO:000467	04626(Plan	receptor pro	11.990668	2.447974	35.243191	25.748884	0.2367298	-2.078687	0.038738267	0.370831314	down	yes	Receptor pr	TMK1
Pavir.9KG194800.v	GO:000373	03010(Ribo	large subun	24.095358	17.283964	131.66429	42.922806	0.2370125	-2.076965	0.028238516	0.312336112	down	yes	60S ribosor	RPL23A
Pavir.4KG192700.v	GO:000367	NA	e2f transcrip	3.024154	1.691681	14.379085	5.51271	0.2370744	-2.076588	0.027312278	0.307615172	down	yes	E2F transcr	E2FE
Pavir.9NG741200.v	NA	NA	ndh-depend	1.057596	0.98321	4.45277	4.154889	0.2370919	-2.076482	0.029423516	0.320224347	down	yes	Photosynthe	PNSB2
Pavir.8NG181800.v	GO:000028	01200(Carb	pyruvate kir	3.810435	2.161834	11.744139	13.433748	0.2372029	-2.075806	0.014789384	0.221740438	down	yes	Pyruvate kir	Os12g0145700
Pavir.3KG121400.v	NA	NA	0	0.594014	0.617786	3.147292	1.960818	0.2372306	-2.075638	0.038673893	0.370386463	down	yes	Interactor o	ICR2
Pavir.8NG327700.v	NA	NA	0	2.59093	0.701861	5.280824	8.595523	0.2372952	-2.075245	0.045186763	0.400660246	down	yes	NA	NA
Pavir.8KG366600.v	GO:000373	03010(Ribo	small subun	13.195457	10.623944	75.462311	24.914371	0.2373001	-2.075215	0.027594827	0.308994921	down	yes	40S ribosor	RPS29
Pavir.6KG280500.v	GO:000551	00940(Pher	shikimate o-	6.541501	3.203553	29.6001	11.436207	0.237474	-2.074159	0.019575898	0.256267806	down	yes	Putrescine h	PHT1
Pavir.2KG359500.v	GO:000967	01200(Carb	uncharacteri	1.6637151	0.3421897	3.8743953	4.5592885	0.2378094	-2.07191	0.046424539	0.40582121	down	yes	Protein PIN	PILS7;PILS7;PILS7
Pavir.2KG251300.v	GO:000548	03013(RNA	nuclear porc	1.790733	1.426773	11.023952	2.500119	0.2379096	-2.071515	0.044879669	0.39955616	down	yes	Nuclear por	NUP54
Pavir.2NG557600.v	GO:000367	NA	domain of u	11.694868	1.693269	25.576509	30.677322	0.2379951	-2.070996	0.036612461	0.359804611	down	yes	NA	NA
Pavir.6NG356600.v	GO:000367	NA	arabidopsis	1.819084	2.200084	8.571036	8.314171	0.2380289	-2.070791	0.028189719	0.311980065	down	yes	NA	NA
Pavir.3KG290800.v	GO:000865	00564(Glyc	phosphatidy	3.581568	0.751092	7.962376	10.233069	0.2381178	-2.070252	0.037288647	0.363533126	down	yes	CDP-diacyl	PSS2
Pavir.1KG282800.v	GO:000373	03010(Ribo	large subun	19.658403	13.311207	103.52065	34.869396	0.2382369	-2.069531	0.028290783	0.312638119	down	yes	60S ribosor	RPL19A
Pavir.2NG101200.v	GO:000333	NA	solute carric	1.430152	1.252434	6.615936	4.62483	0.238648	-2.067044	0.024729603	0.29109191	down	yes	Amino acid	ANT1
Pavir.7KG431800.v	NA	NA	0	2.899488	1.215809	13.280362	3.951433	0.23882	-2.066005	0.040611162	0.379595456	down	yes	NA	NA
Pavir.9KG452600.v	GO:000425	03013(RNA	placental pr	2.813594	0.655192	10.970808	3.549265	0.2388959	-2.065546	0.049786716	0.421245255	down	yes	Rhomboid-l	RBL19
Pavir.6NG034100.v	GO:000367	NA	protein of ui	4.44296	3.684309	18.148157	15.866473	0.2389345	-2.065313	0.015986066	0.232267769	down	yes	NA	NA
Pavir.2KG553600.v	GO:000431	01212(Fatty	beta-ketoac	4.312771	5.482343	27.565992	13.407556	0.2390595	-2.064559	0.014255115	0.217284967	down	yes	3-oxoacyl-[	KAS2
Pavir.6NG306500.v	GO:002262	03010(Ribo	large subun	16.041178	15.400104	96.510353	34.849201	0.2393528	-2.062789	0.019428647	0.255778752	down	yes	60S ribosor	RPL32A
Pavir.2KG524600.v	GO:000582	03010(Ribo	small subun	38.877243	33.409351	233.77481	67.841934	0.2396637	-2.060917	0.03536722	0.354053501	down	yes	40S ribosor	RPS3A
Pavir.1NG398500.v	GO:000557	00500(Starc	zinc finger f	1.5916185	1.3037586	8.8420287	3.2246242	0.2399487	-2.059202	0.029768585	0.322332868	down	yes	GDSL ester	At5g55050;At5g55050
Pavir.6NG347900.v	GO:000047	03010(Ribo	large subun	29.399935	23.927053	169.73856	52.458408	0.2399987	-2.058901	0.033898263	0.344511615	down	yes	60S ribosor	RPL10A
Pavir.1KG002200.v	GO:000333	NA	transmembr	0.804401	0.807387	4.415087	2.298159	0.2400907	-2.058349	0.037371477	0.363911658	down	yes	Amino acid	AVT1C
Pavir.9NG781600.v	GO:000398	00650(Buta	hydroxymet	1.0993169	1.735359	7.8410752	3.9608848	0.2401869	-2.057771	0.027599259	0.308994921	down	yes	Hydroxyme	HMGS;HMGS
Pavir.5NG568600.v	GO:000367	NA	dolichyl-p-r	5.641665	1.391148	13.307995	15.956988	0.240315	-2.057002	0.037134617	0.362973709	down	yes	NA	NA
Pavir.4NG179700.v	GO:00055(	NA	anthranilate	6.388665	1.646863	19.362198	14.068353	0.2403648	-2.056702	0.024153812	0.286868275	down	yes	FT-interacti	FTIP7
Pavir.5NG446700.v	NA	NA	0	18.387039	17.521833	79.657173	69.664062	0.2404807	-2.056007	0.019328567	0.254759839	down	yes	NA	NA
Pavir.4KG093300.v	GO:000373	03010(Ribo	large subun	42.442723	30.009516	220.93407	80.314876	0.2405062	-2.055854	0.032875082	0.339718219	down	yes	60S ribosor	RPL19C;RPL19A
Pavir.1NG344800.v	GO:000002	03010(Ribo	large subun	15.558374	14.038296	88.237658	34.822243	0.2405062	-2.055854	0.026728359	0.304458713	down	yes	60S ribosor	RPL23A;RPL23A;RPL23A
Pavir.8NG275400.v	GO:000373	03010(Ribo	small subun	31.012686	23.933731	169.21101	59.213299	0.2405454	-2.055619	0.028797718	0.315732906	down	yes	40S ribosor	RPS15AA
Pavir.9NG853000.v	GO:00045(	00100(Ster	squalene mc	5.8230145	2.1082212	20.838945	12.128707	0.2405763	-2.055434	0.020234333	0.260721745	down	yes	Squalene m	SQE1;SQE1
Pavir.4NG153800.v	GO:00037(	04712(Circ	b-box zinc f	1.7278544	0.7090033	6.3284175	3.790288	0.240827	-2.053931	0.031365626	0.331318717	down	yes	Zinc finger	COL9;COL9;COL9;COL9;COL9
Pavir.9NG240500.v	GO:000551	00910(Nitro	cytochrome	22.295782	20.103968	120.02743	55.859277	0.2410628	-2.052519	0.023297969	0.281238111	down	yes	Cytochrome	CYB5;CYB5
Pavir.3KG182200.v	NA	NA	0	11.657978	2.48015	32.007965	26.590971	0.2412694	-2.051283	0.029779537	0.322358557	down	yes	NA	NA
Pavir.7KG271500.v	GO:000435	01200(Carb	glutamate d	1.22461	0.763883	5.084577	3.156773	0.2412824	-2.051205	0.025249485	0.294810101	down	yes	Glutamate c	GDH2
Pavir.7NG320500.v	GO:000563	NA	wrc (wrc) //	3.054197	2.757793	19.106373	4.979859	0.2412993	-2.051105	0.031866458	0.334166461	down	yes	Growth-reg	GRF3
Pavir.6KG029600.v	GO:000484	NA	copine//sub	3.522212	1.387634	9.468017	10.853993	0.2416024	-2.049293	0.024595996	0.289920292	down	yes	E3 ubiquitii	RGLG4

Pavir.2NG155800.v	GO:003421	00500(Star	solute carrie	23.439104	9.88009	91.719574	46.01099	0.2419158	-2.047423	0.030726045	0.327994416	down	yes		Phosphoen	PPT1
Pavir.7NG321300.v	NA	NA	s100 calciu	0.523439	0.568806	1.920306	2.594329	0.2419343	-2.047313	0.042276139	0.387803069	down	yes		NA	NA
Pavir.5NG136300.v	GO:000367	00130(Ubic	homeobox-l	2.2163791	0.373642	5.9858056	4.7145038	0.242051	-2.046617	0.041752969	0.384788893	down	yes		Homeobox-	ROC5;ROC5
Pavir.5KG254600.v	GO:000418	00460(Cyar	serine prote	1.436162	0.86182	5.431048	4.053279	0.2422926	-2.045178	0.030152385	0.324425589	down	yes		Serine carb	SCP26
Pavir.1NG435700.v	GO:001602	02010(ABC	lp14331p-r	0.564652	0.9874383	4.0713793	2.3269264	0.2425783	-2.043477	0.024480081	0.289241611	down	yes		ABC transp	ABCB2;ABCB2
Pavir.1KG219700.v	GO:000002	03010(Ribc	small subun	24.618914	22.162216	148.59154	44.255615	0.2425814	-2.043459	0.035900808	0.356279575	down	yes		40S ribosor	RPS27
Pavir.5KG634800.v	GO:00055(	00053(Asc	multi-coppe	6.4006483	10.072853	28.365748	39.350267	0.2432733	-2.03935	0.022246728	0.27450782	down	yes		Laccase-7 (	LAC7;LAC7;LAC7;LAC7;LAC7
Pavir.9NG469200.v	GO:000367	NA	bzip-like pr	2.836688	1.598585	11.273955	6.954791	0.243312	-2.039121	0.018033136	0.245284518	down	yes		Protein RO	REL2
Pavir.7KG175400.v	GO:000431	01212(Fatty	3-oxoacyl- (	1.654315	1.567067	7.611243	5.61837	0.2434978	-2.038019	0.026004335	0.299987994	down	yes		3-oxoacyl-[	KAS12
Pavir.6KG105800.v	GO:000573	00940(Pher	protein ecer	0.708695	0.398347	3.261093	1.28133	0.2437118	-2.036752	0.049806476	0.421291926	down	yes		Protein ECI	CER26
Pavir.9KG383000.v	GO:000002	03010(Ribc	small subun	47.194622	41.390335	277.44534	85.868294	0.243825	-2.036082	0.036059893	0.356986264	down	yes		40S ribosor	RPS19A
Pavir.1KG515400.v	GO:000575	03060(Prot	20 kda chap	6.498807	3.905875	29.449791	13.192778	0.2439975	-2.035061	0.025537031	0.296006694	down	yes		20 kDa cha	CPN20
Pavir.5NG042100.v	GO:000002	03010(Ribc	large subun	5.391978	4.838876	33.37508	8.545666	0.2440523	-2.034738	0.030864376	0.32878257	down	yes		60S ribosor	RPL11
Pavir.3KG147200.v	GO:000367	NA	ppr repeat (	2.216732	2.2368907	15.048804	3.1826955	0.2442818	-2.033382	0.039074979	0.372687063	down	yes		Pentatricop	At4g35850;At4g35850;At4g35850
Pavir.3KG329700.v	GO:000368	03030(DN/	replication f	1.6657417	1.8732335	10.535793	3.9480095	0.2443402	-2.033037	0.021102689	0.267176726	down	yes		Protein STI	At4g18820;At4g18820;At4g18820
Pavir.5KG186600.v	GO:000372	03010(Ribc	large subun	35.56459	30.534185	199.41872	70.854362	0.2445629	-2.031722	0.03016045	0.324425589	down	yes		60S ribosor	RPL30
Pavir.5NG473300.v	GO:000046	03010(Ribc	small subun	67.348618	52.578312	368.42386	121.89879	0.2445878	-2.031576	0.036395704	0.358546357	down	yes		40S ribosor	RPS24A
Pavir.4KG073400.v	GO:000372	03010(Ribc	large subun	37.390282	23.807699	188.31711	61.814865	0.2446628	-2.031134	0.023078829	0.280718955	down	yes		60S ribosor	RPL39
Pavir.7NG234500.v	GO:000367	03030(DN/	ribonucleas	2.339837	2.196925	13.000829	5.52467	0.2448928	-2.029778	0.027695752	0.309361931	down	yes		NA	NA
Pavir.6NG316900.v	GO:000392	04144(Endo	ras-related p	9.676751	3.489157	28.196163	25.560478	0.2449169	-2.029636	0.01910539	0.252941089	down	yes		Ras-related	RABA5D
Pavir.1NG315000.v	GO:000373	03010(Ribc	small subun	31.802332	34.001022	201.21379	66.999039	0.2453401	-2.027145	0.033014057	0.340311982	down	yes		40S ribosor	RPS12
Pavir.9KG205500.v	GO:000437	00500(Star	glycosyltran	0.9724463	0.4925481	4.0016988	1.9623186	0.2456389	-2.025389	0.036585382	0.359804611	down	yes		Soluble sta	SSII-1;SSII-1;SSII-1;SSII-1;SSII-1
Pavir.5NG033600.v	GO:000002	03010(Ribc	large subun	4.094759	3.822798	24.610605	7.621068	0.2456452	-2.025352	0.030409028	0.325931716	down	yes		60S ribosor	RPL11
Pavir.2KG590400.v	GO:000372	03010(Ribc	large subun	8.744503	13.257273	70.993607	18.532457	0.2457583	-2.024688	0.029306516	0.319970669	down	yes		60S ribosor	RPL18C
Pavir.3KG443200.v	GO:000685	NA	multidrug re	2.4638126	1.9256058	10.369698	7.4717494	0.2460237	-2.023131	0.021583489	0.270230878	down	yes		Protein DE	DTX33;DTX33;DTX33;DTX33
Pavir.9NG669000.v	GO:000573	00073(Cuti	ifa-binding	4.6322829	1.4694081	10.526171	14.260174	0.2461715	-2.022265	0.022241812	0.27450782	down	yes		Myosin-bin	MYOB3;MYOB3
Pavir.3KG288400.v	GO:000372	03018(RN/	crs1 / yhby	1.558684	1.125895	6.749843	4.152262	0.2462441	-2.021839	0.019202339	0.253866814	down	yes		CRM-doma	CFM3
Pavir.5KG260800.v	GO:000373	03010(Ribc	small subun	34.562714	28.939878	190.83392	67.021065	0.2462725	-2.021673	0.031956364	0.334642267	down	yes		Ubiquitin-4	MUB1
Pavir.3NG234100.v	GO:000373	03010(Ribc	small subun	33.701955	32.088603	200.15015	66.913055	0.2463483	-2.021229	0.035177682	0.35293153	down	yes		40S ribosor	RPS9C;RPS9C
Pavir.2NG180200.v	GO:000973	04626(Plan	wrky dna -b	0.6752	0.846403	3.03512	3.136756	0.2465382	-2.020117	0.046091106	0.404532086	down	yes		Probable W	WRKY30
Pavir.1NG326400.v	GO:00055(	00260(Glyc	primary-am	2.1985646	1.5967398	12.059618	3.3007727	0.2470838	-2.016927	0.030697896	0.32786016	down	yes		Primary am	At1g31672;At1g31672
Pavir.5NG270100.v	GO:000465	00040(Pent	polygalactu	13.407964	5.350712	41.598885	34.316612	0.2470994	-2.016836	0.027837654	0.310001237	down	yes		Polygalactu	At1g48100
Pavir.2KG224400.v	GO:000013	NA	sec14 relate	1.045527	1.269024	5.728333	3.634406	0.2472087	-2.016198	0.033209917	0.341834488	down	yes		Phosphatid	SFH11
Pavir.4KG074300.v	GO:000372	03010(Ribc	large subun	16.559282	17.156662	107.74314	28.523815	0.2474257	-2.014933	0.027333671	0.30767167	down	yes		60S ribosor	RPL39
Pavir.9KG190600.v	GO:000576	NA	methyltrans	5.4591641	1.215759	17.63699	9.2680242	0.2480922	-2.011052	0.034291184	0.347377367	down	yes		Probable m	At1g04430;At1g04430
Pavir.4NG322300.v	GO:000557	04141(Prot	translocon-a	10.450268	10.313025	60.642914	23.032536	0.2481408	-2.010769	0.021297266	0.268614363	down	yes		Translocon-	At2g21160
Pavir.1KG397100.v	GO:000367	04712(Circ	tcp family t	7.67432	3.347139	27.697811	16.707348	0.2482022	-2.010412	0.026625605	0.303797098	down	yes		Transcriptic	TCP7
Pavir.3NG141600.v	GO:000372	03010(Ribc	large subun	45.297981	47.626415	283.39389	90.807816	0.248327	-2.009687	0.03916348	0.373071272	down	yes		60S ribosor	RPL13AD
Pavir.5NG641500.v	GO:000002	03010(Ribc	small subun	41.092148	28.842785	209.36154	72.167015	0.2484115	-2.009196	0.037709997	0.365544935	down	yes		40S ribosor	RPS10-2
Pavir.4NG325500.v	GO:000557	04141(Prot	translocon-a	13.625951	12.652921	73.098022	32.574497	0.2486822	-2.007625	0.017897834	0.244744653	down	yes		Translocon-	At2g21160
Pavir.1NG272600.v	GO:000373	03010(Ribc	small subun	7.121986	7.881715	44.506535	15.819397	0.2487106	-2.00746	0.026933193	0.305913203	down	yes		40S ribosor	RPS12
Pavir.5NG414200.v	GO:000373	03010(Ribc	large subun	11.826254	8.819565	61.703754	21.267643	0.2488306	-2.006764	0.024157007	0.286868275	down	yes		60S ribosor	Os01g0679700
Pavir.9NG305900.v	GO:000576	NA	methyltrans	13.490701	5.144949	48.59956	26.291634	0.2488363	-2.006731	0.034649679	0.350065174	down	yes		Probable m	At1g04430
Pavir.7KG255700.v	GO:000367	04712(Circ	tcp family t	9.541732	3.303218	34.252605	17.275419	0.2492809	-2.004156	0.026334593	0.302590112	down	yes		Transcriptic	TCP21
Pavir.5KG544700.v	GO:000367	NA	tcp family t	0.994877	1.707243	6.543703	4.29287	0.2493519	-2.003745	0.047357465	0.40989681	down	yes		Transcriptic	PCF7
Pavir.9KG636400.v	GO:000551	03018(RN/	tetratricope	1.2141446	0.370829	4.1043194	2.2493491	0.249458	-2.003131	0.047255423	0.40946722	down	yes		Protein NP(	NPGR1;NPGR1;NPGR1;NPGR1
Pavir.6NG046700.v	GO:000368	03010(Ribc	40s riboson	3.203382	3.248713	17.158419	8.698198	0.2495336	-2.002694	0.035784137	0.35559835	down	yes		40S ribosor	RPS3A
Pavir.2KG310700.v	GO:00047(	04626(Plan	map kinase	6.3432605	1.5804766	22.45863	9.2591916	0.2498197	-2.001041	0.037184462	0.363268797	down	yes		Mitogen-act	NPK1;NPK1
Pavir.6NG024900.v	GO:000663	01230(Bios	chloroplast	1.9108037	1.328324	9.4536348	3.5110723	0.249842	-2.000912	0.041083914	0.381335448	down	yes		NA	NA
Pavir.8NG130300.v	GO:000104	04712(Circ	flavin-bindi	0.607026	0.8268577	3.5264675	2.206112	0.2501289	-1.999256	0.042478422	0.388804321	down	yes		Adagio-like	Os11g0547000;Os11g0547000
Pavir.3NG315000.v	GO:000367	NA	f7a19.6 pro	0.744269	0.431169	2.5816093	2.116452	0.2501964	-1.998867	0.04768113	0.411844391	down	yes		NA	NA
Pavir.9NG609100.v	GO:000467	04075(Plan	inflorescenc	6.144844	4.771388	26.863703	16.760426	0.2502338	-1.998651	0.025221963	0.294580291	down	yes		Probable in	At5g10020
Pavir.4KG009100.v	GO:000218	03010(Ribc	large subun	72.058289	60.002083	386.12534	141.29405	0.2503897	-1.997753	0.037621892	0.36507421	down	yes		60S ribosor	RPL9
Pavir.5NG004600.v	GO:000573	03013(RN/	zinc phosph	0.8941965	2.1074519	7.7492367	4.2325732	0.2505171	-1.997019	0.026922987	0.305889745	down	yes		tRNase Z T	TRZ3;TRZ3;TRZ4
Pavir.9KG192900.v	GO:000373	03010(Ribc	large subun	26.774826	15.602763	119.12847	49.834141	0.2508105	-1.995331	0.021657584	0.27085151	down	yes		60S ribosor	RPL23A
Pavir.3KG337600.v	GO:000551	NA	iq calmodul	1.2580928	1.2661741	6.9243656	3.1346772	0.250945	-1.994557	0.032061143	0.33527229	down	yes		NA	NA
Pavir.1NG288600.v	GO:000443	NA	phosphatidy	5.0611843	1.1686217	15.577485	9.2175864	0.2512518	-1.992794	0.034088108	0.345692976	down	yes		Phosphoino	SAC7;SAC7;SAC7
Pavir.6NG066400.v	GO:000368	03010(Ribc	small subun	22.905633	21.298903	131.19802	44.58886	0.2514666	-1.991561	0.036105797	0.357134649	down	yes		40S ribosor	RPS3C;RPS3C
Pavir.5KG542300.v	GO:000392	03008(Ribc	elongation f	5.286075	4.918626	28.603832	11.954696	0.2516043	-1.990771	0.021456324	0.26972793	down	yes		Putative elo	SVR3
Pavir.5KG361300.v	NA	NA	protein of u	0.8087182	0.3626762	3.0692702	1.5829904	0.2517904	-1.989705	0.044620588	0.398414767	down	yes		NA	NA
Pavir.5KG635900.v	GO:000573	NA	starch phos	13.41456	11.503412	55.891979	43.020195	0.2519202	-1.988961	0.026526164	0.303412787	down	yes		Alpha-gluc	-
Pavir.9NG500000.v	GO:000573	03060(Prot	yidc/oxa1 fa	3.268975	2.06669	15.111752	6.066278	0.2519434	-1.988828	0.028655878	0.314820844	down	yes		Mitochondr	OXA1
Pavir.9NG465900.v	GO:000025	00100(Ster	4,4-dimethy	14.774322	6.800503	59.196556	26.39637	0.2520632	-1.988143	0.033858	0.344406025	down	yes		Very-long-c	GL1-10
Pavir.1KG480800.v	GO:000218	03010(Ribc	large subun	55.152054	35.264938	268.81924	89.835281	0.2521005	-1.987929	0.040495201	0.379243812	down	yes		60S ribosor	RPL31
Pavir.7KG230400.v	GO:000373	03010(Ribc	small subun	18.946972	26.131773	143.14026	35.66116	0.2521163	-1.987839	0.043654192	0.394673885	down	yes		40S ribosor	RPS11
Pavir.5NG613400.v	GO:000563	00040(Pent	helix-loop-h	8.273249	2.516969	26.691431	16.079275	0.2522806	-1.986899	0.027579453	0.308994921	down	yes		Transcriptic	SCRM
Pavir.4KG217200.v	GO:00055(	NA	annexin//sul	13.900939	8.402775	54.956478	33.196644	0.2530111	-1.982728	0.021697182	0.270864201	down	yes		Annexin D2	ANN2
Pavir.8NG051400.v	GO:000487	NA	late embryo	4.2545339	2.24191	14.551277	11.114679	0.2531152	-1.982134	0.0204397	0.262467822	down	yes		NDR1/HIN	NHL1;NHL1
Pavir.1NG443800.v	GO:000508	NA	rop guanine	3.858132	0.7651049	11.702272	6.5536675	0.2532456	-1.981391	0.044745881	0.398922365	down	yes		Rop guanin	ROPGEF14;ROPGEF14
Pavir.6NG215500.v	GO:00001(	NA	thioredoxin-	1.470815	3.211262	13.002822	5.4795	0.2533273	-1.980925	0.048331744	0.414226418	down	yes		Thioredoxi	OsI_29059
Pavir.1KG006900.v	GO:000551	NA	bag family r	2.554109	6.749075	28.082102	8.606989	0.2535681	-1.979555	0.04607104	0.404532086	down	yes		BAG family	BAG7
Pavir.3KG055800.v	GO:000028	00562(Inos	3'(2'), 5'-bis	2.00297	1.019394	5.792606	6.119904	0.2537134	-1.978728	0.04001556	0.377133998	down	yes		3'(2'),5'-bis	Os12g0183300
Pavir.6KG273300.v	GO:000372	03010(Ribc	large subun	36.055965	27.021404	190.47043	57.939625	0.2539244	-1.977529	0.040614791	0.379595456	down	yes		60S ribosor	RPL34
Pavir.9NG037800.v	GO:000373	03010(Ribc	small subun	25.285671	20.160416	133.34747	45.524357	0.2540707	-1.976698	0.035666448	0.355310568	down	yes		40S ribosor	RPS23
Pavir.2NG587400.v	GO:000431	01212(Fatty	beta-ketoac	1.7661795	2.9777213	13.146868	5.521386	0.2541159	-1.976441	0.027280573	0.307442404	down	yes		3-oxoacyl-[	KAS2;KAS2
Pavir.1KG262700.v	GO:000557	00514(Othe	genomic dn	4.3483609	1.0317825	12.605139	8.5368165	0.2544771	-1.974392	0.036995565	0.362460073	down	yes		Alpha-1,3-a	XAT2;XAT2;XAT2;XAT2;XAT2;XAT2
Pavir.1NG385500.v	GO:000416	00900(Terp	(2e,6e)-farn	17.170149	9.039755	75.067841	27.726681	0.2549737	-1.971579	0.028834183	0.31604048	down	yes		Farnesyl py	FPS
Pavir.2NG054000.v	GO:000218	03010(Ribc	large subun	8.2212616	5.3032197	41.41601	11.578975	0.255203	-1.970283	0.033604094	0.343566433	down	yes		60S acidic r	RPP2A;RPP2A
Pavir.2KG307000.v	GO:000372	03010(Ribc	large subun	25.044031	21.183771	140.81477	40.295685	0.2552465	-1.970037	0.039807833	0.376276654	down	yes		60S ribosor	RPL34
Pavir.9NG440300.v	GO:000218	03010(Ribc	large subun	98.894204	81.868917	555.0492	152.76894	0.2553807	-1.969278	0.047998235	0.413183315	down	yes		60S ribosor	RPL36C;RPL36C
Pavir.8KG333500.v	GO:000904	00500(Star	beta-d-xylos	4.025128	3.295747	11.063398	17.599934	0.2554091	-1.969118	0.017496301	0.242738038	down	yes		Probable be	BXL2
Pavir.4NG182000.v	GO:000382	NA	hypothetical	1.901152	1.006504	6.62961	4.75423	0.2554196	-1.969059	0.025683458	0.297198817	down	yes		NA	NA

Pavir.6NG315100.v	GO:000367	NA	sbp domain	1.3603342	1.0479038	7.3447458	2.0813914	0.2554851	-1.968689	0.034706921	0.350364356	down	yes	Squamosa p	SPL14;SPL14;SPL14;SPL14;SPL14
Pavir.2KG063900.v	NA	NA	0	1.7330818	1.8649825	9.9345693	4.1451452	0.2555495	-1.968325	0.040066772	0.377300462	down	yes	PLASTID T	PTAC6;PTAC6
Pavir.3NG257000.v	GO:000435	00650(Buta	group ii pyr	4.042251	0.99764	12.907657	6.809554	0.2556087	-1.967991	0.048344143	0.414238198	down	yes	Glutamate c	GAD
Pavir.8NG284000.v	GO:000373	03010(Ribc	large subun	30.400518	22.321665	141.32614	64.890221	0.2556644	-1.967677	0.033750851	0.343943783	down	yes	60S ribosor	RPL19A
Pavir.5KG466200.v	GO:000392	04144(Endc	ras-related p	3.8244373	2.1763016	14.964306	8.5022881	0.2557141	-1.967396	0.029972616	0.323423634	down	yes	Ras-related	-;-;-
Pavir.1NG181000.v	GO:000578	04144(Endc	extended sy	12.212243	2.781036	27.208357	31.31325	0.2562007	-1.964653	0.04465705	0.398557495	down	yes	Synaptotagr	SYT2;SYT2
Pavir.9NG664000.v	NA	NA	0	2.456021	2.480782	13.219522	6.033615	0.2564155	-1.963445	0.024302738	0.287780269	down	yes	NA	NA
Pavir.7NG021000.v	GO:000002	03010(Ribc	small subun	32.54319	24.513905	154.54248	67.804008	0.2566134	-1.962331	0.032285784	0.336497573	down	yes	40S ribosor	RPS27
Pavir.2NG373500.v	GO:000382	00940(Pher	nad depende	1.889001	2.483177	11.848261	5.173199	0.2568627	-1.960931	0.04942354	0.419634379	down	yes	Cinnamoyl-	CCR1
Pavir.9KG017000.v	GO:000370	NA	transcriptio	13.236588	10.329508	68.437798	23.270103	0.2569691	-1.960333	0.026543471	0.303412787	down	yes	Basic transc	BTF3
Pavir.3KG414900.v	NA	NA	0	1.926021	2.025778	11.881097	3.485348	0.2571707	-1.959202	0.048950422	0.416966544	down	yes	NA	NA
Pavir.1NG552500.v	GO:000573	03010(Ribc	39s riboson	2.6392046	3.737249	13.604833	11.183701	0.257234	-1.958847	0.028146553	0.311777839	down	yes	50S ribosor	RPL9;RPL9
Pavir.8NG298500.v	GO:000025	00100(Stero	4-alpha-met	15.736848	22.564367	84.504616	64.218803	0.2575332	-1.95717	0.02871929	0.315148911	down	yes	Very-long-c	GL1-11
Pavir.9KG547100.v	GO:000016	01230(Bios	anthranilate	3.466108	3.615609	20.341434	7.108327	0.2579883	-1.954622	0.029078507	0.318289052	down	yes	Anthranilat	PAT1
Pavir.1NG008300.v	GO:000372	03010(Ribc	large subun	14.603942	11.974899	78.098648	24.829353	0.2582275	-1.953285	0.028360485	0.312948164	down	yes	60S ribosor	RPL37A
Pavir.6NG305700.v	GO:000444	00562(Inos	inositol 5-pl	0.384563	0.473423	2.237872	1.0841288	0.2582739	-1.953026	0.047402631	0.410098789	down	yes	Type I inosi	IP5P13;IP5P12;IP5P12
Pavir.1NG384100.v	GO:000373	03010(Ribc	large subun	31.167393	23.403407	163.58144	47.523369	0.258501	-1.951758	0.045011558	0.400288581	down	yes	60S ribosor	RPL28A;RPL28A
Pavir.9NG600400.v	GO:000425	04144(Endc	proprotein c	8.915032	1.809058	23.068848	18.3335	0.2590213	-1.948857	0.03885366	0.371444599	down	yes	Subtilisin-li	SBT1.8
Pavir.5KG681600.v	GO:000588	NA	mlo-like pro	1.111044	1.295741	6.836459	2.453787	0.2590658	-1.94861	0.048321712	0.414226418	down	yes	MLO-like p	MLO1
Pavir.6NG154500.v	GO:000550	NA	annexin//sul	9.4051985	5.9221514	34.499806	24.621759	0.2592514	-1.947576	0.01880955	0.250638862	down	yes	Annexin D7	ANNAT7;ANNAT7
Pavir.9KG578200.v	GO:000010	01230(Bios	atp phospho	2.194005	1.248393	8.023714	5.254172	0.259258	-1.94754	0.037094816	0.362937039	down	yes	ATP phospl	Os03g0134300
Pavir.1KG149800.v	GO:000465	00040(Pent	glycoside hy	5.308729	1.605209	15.319277	11.342424	0.259321	-1.947189	0.029614448	0.321404863	down	yes	Probable po	GSVIVT00026920001
Pavir.1NG349200.v	GO:000577	00531(Glyc	aquaporin tr	11.328338	6.741882	24.270512	45.349243	0.2595559	-1.945883	0.024054186	0.286371335	down	yes	Aquaporin I	PIP2-4
Pavir.1NG497900.v	GO:000551	NA	atp binding	1.1378837	1.1261	6.2469726	2.4738693	0.2596061	-1.945604	0.048082804	0.413516993	down	yes	SAP-like pr	BP-73;BP-73
Pavir.5NG479000.v	GO:000368	03030(DNA	protein stich	8.2543758	3.005146	25.039298	18.317922	0.259692	-1.945127	0.028209011	0.31210164	down	yes	Protein STI	At4g18820;At4g18820;At4g18820
Pavir.5KG697100.v	GO:000842	00500(Stare	beta-glucosi	14.491534	6.509459	39.364574	41.485962	0.2597508	-1.9448	0.034727594	0.350391522	down	yes	Beta-glucos	BGLU4
Pavir.7NG248800.v	GO:000573	NA	prohibitin 1	2.344471	3.598968	18.560291	4.29272	0.2600725	-1.943014	0.041887982	0.385680547	down	yes	Prohibitin-3	PHB3
Pavir.2NG092600.v	GO:000570	00100(Stero	sterol 24-c-r	37.036767	13.520424	90.341546	103.99606	0.2601514	-1.942577	0.04257415	0.389038382	down	yes	Cycloartenc	Smt1-1;Smt1-1
Pavir.9KG568400.v	GO:000577	04146(Pero	peroxisoma	1.2710364	1.9620355	7.6554905	4.7718736	0.2601575	-1.942543	0.038483946	0.36999931	down	yes	Peroxisoma	PEX11-1;PEX11-1
Pavir.3NG034800.v	GO:000485	NA	plant inverta	5.7823	1.644025	18.33724	10.203965	0.2601966	-1.942326	0.042590369	0.389038382	down	yes	Pectinestera	PMEI28
Pavir.4KG351200.v	GO:000367	NA	tetraspanin	19.653795	4.596669	59.67852	33.521652	0.2601976	-1.94232	0.043397979	0.393008497	down	yes	Tetraspanin	TET8
Pavir.9NG287900.v	GO:000373	03010(Ribc	large subun	50.728809	46.803123	277.83502	96.731377	0.2603862	-1.941275	0.043397599	0.393008497	down	yes	60S ribosor	RPL18A
Pavir.2NG588300.v	GO:000550	01230(Bios	ll-diaminop	1.643707	2.531277	11.761024	4.272077	0.2603978	-1.941211	0.038825694	0.371384368	down	yes	Probable LI	AGD2
Pavir.6KG066700.v	GO:000636	03008(Ribc	u3 small nu	3.3979155	3.521512	21.254805	5.3099133	0.2604743	-1.940787	0.039437711	0.374504823	down	yes	NA	NA
Pavir.7KG188100.v	GO:000372	03010(Ribc	large subun	14.55223	11.348816	76.17234	23.009655	0.2611467	-1.937068	0.031157684	0.330075502	down	yes	60S ribosor	RPL34
Pavir.9KG554600.v	GO:000367	03060(Prot	cobra-like p	1.898002	0.746786	5.973929	4.152826	0.2611684	-1.936948	0.040395912	0.378880576	down	yes	COBRA-lik	COBL7
Pavir.2KG056800.v	GO:000372	04141(Prot	yth yt521-b	3.38355	5.713763	27.845539	6.987194	0.2611714	-1.936931	0.038861649	0.371444599	down	yes	YTH domai	ECT4
Pavir.5KG435900.v	GO:000014	00500(Stare	callose synt	2.37717	1.953425	11.349666	5.222332	0.26132	-1.93611	0.022224336	0.27450782	down	yes	Callose syn	CALS12
Pavir.3NG058400.v	NA	NA	procyclic ac	108.2911	66.10659	323.60037	343.71326	0.2613429	-1.935984	0.03617377	0.357578813	down	yes	NA	NA
Pavir.9KG561300.v	GO:000370	NA	transcriptio	5.3849669	1.22631	9.1445346	16.149787	0.261374	-1.935813	0.03945489	0.374504823	down	yes	Trihelix tra	DF1;DF1
Pavir.9KG434100.v	GO:000433	01200(Carb	fumarate hy	15.870675	7.7655951	55.0534	35.330883	0.2615086	-1.93507	0.035224192	0.353115946	down	yes	Fumarate hy	FUM1;FUM1
Pavir.1NG245900.v	GO:000034	03030(DNA	dna replicat	1.3632402	0.9750714	5.8688758	3.0656454	0.2617165	-1.933923	0.045572463	0.402513901	down	yes	DNA replic	MCM5;MCM5
Pavir.4KG090300.v	GO:000367	NA	uncharacteri	3.225901	3.55182	19.0445	6.840149	0.2618433	-1.933225	0.027833529	0.310001237	down	yes	NA	NA
Pavir.9NG482100.v	GO:002262	03010(Ribc	large subun	28.647978	25.626582	151.54317	55.560613	0.2620646	-1.932006	0.039653121	0.375475482	down	yes	60S ribosor	RPL44;RPL44
Pavir.1NG011700.v	GO:000367	NA	remorin, c-te	7.4419024	1.3612171	18.924386	14.651575	0.2621852	-1.931342	0.044897014	0.39955616	down	yes	NA	NA
Pavir.6NG307600.v	GO:000392	04144(Endc	ras-related p	8.572779	2.671126	23.989588	18.843504	0.2625051	-1.929583	0.031119702	0.330075502	down	yes	Ras-related	RABA5D
Pavir.3NG053600.v	GO:000969	04626(Plan	nucleoporin	1.337666	0.804377	4.019305	4.127773	0.2629216	-1.927295	0.049548576	0.420417763	down	yes	Horcolin OS	-
Pavir.9NG542000.v	NA	NA	0	0.770043	0.909504	3.213744	3.171033	0.2630549	-1.926564	0.042669661	0.389038382	down	yes	NA	NA
Pavir.1KG466200.v	GO:000508	NA	rop guanine	2.948397	0.834253	9.315991	5.06173	0.2630911	-1.926366	0.044600034	0.398414767	down	yes	Rop guanin	ROPGEF14
Pavir.4KG173400.v	GO:000017	00600(Sphi	sphinganine	1.833107	1.961416	9.522557	4.898756	0.2631191	-1.926212	0.037862815	0.366194433	down	yes	Sphinganine	SBH1
Pavir.7NG374100.v	GO:000815	NA	predicted m	38.631657	16.934273	104.87756	105.1243	0.2645973	-1.91813	0.038534097	0.370223692	down	yes	Uncharacter	At4g09580
Pavir.2KG542900.v	GO:000467	04626(Plan	protein kina	5.636179	1.14523	15.029077	10.587738	0.2647249	-1.917434	0.047065008	0.408683509	down	yes	Probable in	At5g10020
Pavir.2KG405600.v	GO:000467	00010(Glyc	ribosomal p	4.182305	1.279783	14.23455	6.385136	0.2648966	-1.916498	0.039946051	0.377133998	down	yes	Protein kina	Os06g0291600
Pavir.1NG014900.v	GO:000002	03010(Ribc	small subun	31.673094	31.538513	175.98091	62.629227	0.2649158	-1.916394	0.042683797	0.389072854	down	yes	40S ribosor	RPS15
Pavir.4NG030800.v	GO:000478	04146(Pero	superoxide	1.387825	1.303696	7.150545	2.997611	0.2652227	-1.914724	0.048046562	0.413385271	down	yes	Superoxide	Os06g0115400
Pavir.4KG151400.v	GO:000467	00520(Ami	s-locus glyc	1.191979	0.911339	5.4680269	2.45246	0.2655541	-1.912922	0.041950733	0.385759888	down	yes	Receptor-lil	SD18;SD18
Pavir.9NG594700.v	GO:000467	03022(Basa	cell division	1.5966556	1.5197684	9.0463944	2.6807051	0.2657455	-1.911883	0.04866739	0.416023543	down	yes	Probable se	At1g54610;At1g54610
Pavir.5NG288000.v	GO:000002	03010(Ribc	small subun	29.733755	31.426355	173.06046	56.986627	0.2658591	-1.911266	0.048154233	0.413516993	down	yes	40S ribosor	RPS5;RPS5
Pavir.7KG422100.v	GO:000467	04075(Plan	protein kina	4.385214	2.010204	17.303686	6.727107	0.2661343	-1.909774	0.033804257	0.344207948	down	yes	Probable in	At5g67200
Pavir.8KG002600.v	GO:000370	04075(Plan	protein scar	2.351803	1.050273	7.98674	4.779953	0.2664806	-1.907898	0.031180536	0.330195252	down	yes	Protein SCA	SCR
Pavir.9KG515900.v	GO:000573	NA	zein-binding	5.156655	2.4599475	14.358298	14.207464	0.266634	-1.907068	0.027969468	0.310732356	down	yes	Myosin-bin	MYOB2;MYOB2;MYOB2
Pavir.8NG340800.v	GO:000373	03010(Ribc	60s riboson	11.257497	11.88007	65.855698	20.758621	0.2671333	-1.904368	0.03928092	0.373489416	down	yes	60S ribosor	RPL19C
Pavir.9NG286400.v	GO:000374	03013(RNA	translation i	5.916619	3.200496	24.200014	9.923692	0.2671783	-1.904125	0.030826377	0.328578568	down	yes	Eukaryotic	TIF3D1
Pavir.5KG112900.v	GO:000373	03010(Ribc	s25 riboson	26.178204	13.191479	93.037575	54.117702	0.2675384	-1.902182	0.027198685	0.306979929	down	yes	40S ribosor	RPS25D
Pavir.3KG042300.v	GO:000557	NA	probable lip	20.09137	6.934861	52.198532	48.815926	0.2675481	-1.90213	0.040680415	0.379923424	down	yes	Non-specifi	LTP2-A
Pavir.7KG345200.v	GO:000002	03010(Ribc	large subun	15.301277	14.360983	80.563225	30.263844	0.2676445	-1.90161	0.040835944	0.380255	down	yes	60S ribosor	RPL11
Pavir.3NG103000.v	NA	NA	glyoxalase-l	5.207054	7.172886	36.62693	9.610499	0.2677472	-1.901057	0.04428648	0.397524521	down	yes	NA	NA
Pavir.3KG317500.v	GO:000416	00900(Terp	farnesyl dip	19.735168	10.792463	76.121506	37.842457	0.2678709	-1.90039	0.039756531	0.376075563	down	yes	Farnesyl py	FPS
Pavir.3NG032800.v	GO:000520	04145(Phag	actin//subfa	38.69474	24.389748	77.417213	157.74049	0.2682646	-1.898271	0.035602803	0.354922824	down	yes	Actin-7 OS	ACT7
Pavir.7NG074800.v	GO:000372	NA	rna-directed	1.356416	0.982787	5.237721	3.481885	0.2682693	-1.898246	0.026839749	0.305128561	down	yes	Probable RI	RDR3
Pavir.5NG645700.v	GO:000382	00520(Ami	udp-apiose/	25.745504	24.128191	83.271505	102.53482	0.2684176	-1.897449	0.035488354	0.3540943	down	yes	UDP-D-api	Os01g0969100;Os01g0969100
Pavir.6KG378600.v	GO:006148	03010(Ribc	large subun	20.954637	17.812535	106.31884	37.785985	0.2690206	-1.894211	0.043857053	0.395735127	down	yes	60S ribosor	RPL17;RPL17;RPL17
Pavir.3KG144200.v	GO:000367	NA	ppr repeat (	4.834844	4.4843834	26.886858	7.7331965	0.2691858	-1.893326	0.037059214	0.362799852	down	yes	Pentatricop	At4g35850;At4g35850
Pavir.8NG267300.v	GO:000372	03010(Ribc	large subun	3.408861	3.366946	19.80818	5.359462	0.2692269	-1.893105	0.044235333	0.397335447	down	yes	NA	NA
Pavir.3KG136400.v	GO:000467	04626(Plan	leucine rich	5.152967	1.395181	12.349145	11.959454	0.2693758	-1.892308	0.03606632	0.356986264	down	yes	Probable LI	IRK
Pavir.2NG622200.v	GO:000374	NA	elongation f	57.712364	46.456585	274.91345	111.36948	0.2696701	-1.890733	0.047094888	0.408815155	down	yes	Elongation	Os07g0662500
Pavir.5KG319800.v	GO:000218	NA	translation i	4.915587	5.51327	28.190155	10.427635	0.2700532	-1.888685	0.030727926	0.327994416	down	yes	NA	NA
Pavir.9KG532600.v	GO:000373	03010(Ribc	large subun	71.903442	65.724701	370.86026	138.65253	0.2701172	-1.888343	0.043912725	0.395857199	down	yes	Ubiquitin-6	Ub-CEP52-2
Pavir.1KG378300.v	GO:000367	NA	ppr repeat (	1.090244	1.247997	6.030004	2.624534	0.2701751	-1.888033	0.049248245	0.418548818	down	yes	Protein NU	NFD5
Pavir.2KG233000.v	GO:002262	03010(Ribc	large subun	27.986406	26.568825	150.95111	50.801563	0.2704065	-1.886798	0.045191976	0.400660246	down	yes	60S ribosor	RPL44
Pavir.9KG429800.v	GO:000013	00500(Stare	galacturono	1.724601	1.117451	6.498007	4.003245	0.2706393	-1.885556	0.037433084	0.364237061	down	yes	Probable ga	GAUT7
Pavir.1KG043800.v	GO:000577	00100(Stero	cycloartenol	5.178676	6.337397	29.057022	13.473081	0.2707746	-1.884836	0.037244219	0.363533126	down	yes	Cycloartenc	Os02g0139700

Pavir.3KG286100.v	GO:000465	00040(Pent	polygalactu	4.193175	1.406885	12.25092	8.39566	0.2712343	-1.882389	0.040998994	0.380928544	down	yes	Probable pc	At1g80170
Pavir.6NG016000.v	GO:000401	00270(Cyst	adenosylhoi	22.009203	8.181364	64.312454	46.901386	0.2714641	-1.881167	0.045370656	0.401486498	down	yes	Adenosylho	SAHH
Pavir.1NG296200.v	NA	NA	dextransucr	9.292321	1.891899	17.760191	23.418783	0.2716003	-1.880443	0.048503263	0.41512827	down	yes	NA	NA
Pavir.2NG272500.v	GO:002262	03010(Ribo	large subun	49.202415	36.145958	222.00734	92.156685	0.2716682	-1.880082	0.045080179	0.400506709	down	yes	60S ribosor	RPL44
Pavir.3NG202800.v	GO:000373	03010(Ribo	small subun	12.064857	14.148873	69.422864	26.705433	0.2726953	-1.874639	0.039084202	0.372687063	down	yes	40S ribosor	RPS9C;RPS9C
Pavir.3KG301100.v	GO:000072	NA	e3 sumo-pro	2.768813	3.740641	14.471951	9.39804	0.2727045	-1.87459	0.039652494	0.375475482	down	yes	E3 SUMO-	MMS21
Pavir.7NG202100.v	GO:000002	03010(Ribo	small subun	39.737637	35.150723	198.58377	75.145966	0.273585	-1.869939	0.049132698	0.417944951	down	yes	40S ribosor	RPS10-2
Pavir.5NG596200.v	GO:000367	NA	awpm-19-li	1.3968349	2.0081329	5.9068198	6.5373702	0.2736191	-1.869759	0.047235226	0.40946722	down	yes	Membrane p	PM19L;PM19L
Pavir.1NG534100.v	GO:000367	NA	protein of u	4.702885	3.220571	22.29567	6.642767	0.2738039	-1.868785	0.045279699	0.401106775	down	yes	Probable tra	At4g00390
Pavir.2NG478900.v	GO:000573	NA	rrna-proces	3.670356	3.559384	19.617208	6.77542	0.2739303	-1.868119	0.040088298	0.37740857	down	yes	Probable rR	EBP2
Pavir.8NG337600.v	GO:000373	03010(Ribo	60s ribosom	13.04568	12.960312	71.568207	23.238873	0.2743043	-1.866151	0.041779686	0.384940688	down	yes	60S ribosor	RPL19C
Pavir.5NG058200.v	GO:001678	NA	alpha/beta-l	4.6715316	6.0986655	23.587991	15.623324	0.2746706	-1.864225	0.028531176	0.314001722	down	yes	NA	NA
Pavir.9NG199300.v	GO:000487	NA	late embryo	2.722031	1.481773	6.564082	8.733792	0.2747966	-1.863564	0.039962774	0.377133998	down	yes	NA	NA
Pavir.3KG077200.v	GO:003421	01230(Bios	solute carric	8.55687	6.5813613	36.099325	18.93743	0.2750568	-1.862199	0.026819508	0.304990732	down	yes	Triose phos	TPT;TPT
Pavir.5KG451800.v	GO:000465	00040(Pent	polygalactu	1.960537	2.561554	8.174347	8.259806	0.2751642	-1.861635	0.030261377	0.324966107	down	yes	Polygalactu	At1g48100
Pavir.7NG267100.v	GO:000435	01200(Carb	glutamate d	5.14282	1.871591	14.591704	10.881586	0.2753634	-1.860591	0.037864035	0.366194433	down	yes	Glutamate c	GDH2
Pavir.9KG374100.v	GO:000582	NA	3-oxo-5-bet	17.074564	7.544866	42.44949	46.936214	0.2754292	-1.860247	0.044863636	0.39955616	down	yes	3-oxo-Delta	-
Pavir.5KG178600.v	GO:000367	NA	lysosomal a	3.421535	4.294711	13.385925	14.607757	0.2756424	-1.85913	0.022523627	0.276856408	down	yes	NA	NA
Pavir.5KG665000.v	GO:003059	00040(Pent	pectinestera	7.127552	3.326826	24.04952	13.812174	0.2761202	-1.856632	0.031335426	0.331185922	down	yes	Probable pe	PME51
Pavir.5KG481200.v	NA	NA	0	9.4964428	8.2258858	23.271566	40.866229	0.2763165	-1.855607	0.024990168	0.293240345	down	yes	NA	NA
Pavir.9NG394600.v	GO:000002	03010(Ribo	small subun	20.590643	16.926863	100.25424	35.475582	0.2764131	-1.855102	0.042207948	0.387366693	down	yes	40S ribosor	RPS15
Pavir.2NG049000.v	GO:000016	03013(RNA	translation i	16.255978	12.100664	70.567619	31.908815	0.2767138	-1.853534	0.042991821	0.391026793	down	yes	Eukaryotic t	TIF3I1
Pavir.2NG373400.v	GO:000442	00900(Terp	hydroxymet	9.1245536	2.8363739	26.698802	16.43441	0.277302	-1.85047	0.046797108	0.407109922	down	yes	3-hydroxy-3	HMGR;HMGR
Pavir.6KG357300.v	GO:000373	04146(Pero	mitochondri	2.2214	1.865036	11.027001	3.681842	0.2778217	-1.847769	0.047583754	0.41119236	down	yes	Probable m	At3g20240
Pavir.2KG541700.v	GO:000367	03040(Splic	myb-related	3.0007903	2.226636	11.080688	7.7235882	0.2779914	-1.846888	0.026421411	0.30297155	down	yes	Transcriptic	MYB88;MYB88
Pavir.7NG205500.v	GO:000002	03010(Ribo	small subun	28.272732	24.621044	140.83695	49.145081	0.2784146	-1.844693	0.048986602	0.417174432	down	yes	40S ribosor	RPS27
Pavir.9KG285200.v	GO:000550	NA	annexin//sul	7.054556	4.248097	22.293095	18.302353	0.2784217	-1.844657	0.031062823	0.329882262	down	yes	Annexin D7	ANNAT7
Pavir.3NG191100.v	GO:000374	03013(RNA	elongation f	26.395405	23.591602	96.75563	82.57373	0.2787441	-1.842987	0.033408061	0.342774737	down	yes	Elongation	TEF1
Pavir.3NG033800.v	GO:000398	01200(Carb	acetyl-coa c	15.230905	12.050208	64.349686	33.427685	0.2790125	-1.841598	0.042962748	0.390907404	down	yes	Acetyl-CoA	AAT1
Pavir.1KG136200.v	GO:000038	03040(Splic	small nuclea	7.9821261	4.7736338	34.846944	10.8405	0.2791962	-1.840649	0.047518151	0.410907277	down	yes	NA	NA
Pavir.7NG435300.v	GO:000165	04075(Plan	protein kina	1.652756	0.778383	4.383839	4.309375	0.2796594	-1.838257	0.037288922	0.363533126	down	yes	Phytosulfok	PSKR
Pavir.7NG177500.v	GO:000372	03010(Ribo	large subun	10.23251	9.010949	52.998367	15.689603	0.2801576	-1.835689	0.046029208	0.40453198	down	yes	60S ribosor	RPL34
Pavir.1NG282800.v	GO:000002	03010(Ribo	small subun	13.428637	13.476315	76.929817	19.085087	0.2802164	-1.835387	0.047755496	0.412130382	down	yes	40S ribosor	-
Pavir.2NG085500.v	GO:000373	NA	small subun	3.024737	2.699118	15.00009	5.402671	0.2805432	-1.833705	0.043643927	0.394673885	down	yes	NA	NA
Pavir.1NG261400.v	GO:000535	NA	mfs transpo	1.190508	2.543791	6.091103	7.201831	0.2809236	-1.83175	0.043889299	0.395740974	down	yes	Sugar trans	At1g19450
Pavir.5NG272700.v	GO:000467	04626(Plan	protein kina	13.987672	5.116509	43.326038	24.612104	0.2811996	-1.830333	0.039708952	0.375814719	down	yes	Probable re	At2g42960
Pavir.7KG137900.v	GO:000815	00520(Ami	nuclear inhi	3.380692	3.771126	16.223564	9.191808	0.2813973	-1.829319	0.040640466	0.379657935	down	yes	NA	NA
Pavir.9NG367200.v	GO:000373	00270(Cyst	ribosomal p	6.651033	5.409845	30.754255	12.035307	0.281865	-1.826924	0.041050979	0.381218103	down	yes	NA	NA
Pavir.7KG201200.v	GO:000024	03040(Splic	small nuclea	7.206686	7.840424	39.708042	13.448454	0.2830719	-1.82076	0.044768247	0.398974701	down	yes	Small nucle	SMD1B
Pavir.1KG443600.v	GO:000373	03010(Ribo	small subun	10.656355	6.968475	44.374416	17.793003	0.2835059	-1.818549	0.045764072	0.403146483	down	yes	40S ribosor	RPS29
Pavir.3KG157100.v	NA	NA	0	13.521921	6.112913	31.67128	37.58527	0.2835087	-1.818535	0.04614553	0.404713246	down	yes	NA	NA
Pavir.5NG505800.v	GO:000372	NA	protein of u	9.736058	5.039165	30.409557	21.664062	0.2837372	-1.817373	0.046905924	0.407867688	down	yes	Endoribonu	DCL3B
Pavir.9NG758700.v	GO:000401	00230(Puri	adenylate ki	7.020618	4.050974	27.21244	11.762516	0.2840694	-1.815685	0.038100189	0.367696562	down	yes	Probable ad	Os03g0130400
Pavir.4NG217400.v	GO:000022	00190(Oxic	protein regu	5.055499	5.474796	27.730055	9.334147	0.2843184	-1.815481	0.040721088	0.380028688	down	yes	65-kDa mic	MAP65-1
Pavir.1KG172100.v	GO:000697	00130(Ubic	tocopherol c	1.5597428	1.3940377	6.7835177	3.6008262	0.2844456	-1.813776	0.04656131	0.406108626	down	yes	Probable to	VTE1;VTE1
Pavir.2NG404300.v	GO:000442	00900(Terp	hydroxymet	9.258731	2.696659	19.32131	22.700686	0.2845031	-1.813483	0.042905459	0.390524907	down	yes	3-hydroxy-3	HMGR
Pavir.1NG533300.v	NA	NA	0	4.9319784	2.1094136	14.231697	10.48299	0.2849072	-1.811436	0.034823168	0.350924653	down	yes	NA	NA
Pavir.9NG270200.v	GO:000667	NA	ceramide glu	5.332251	2.091757	12.552582	13.493606	0.2850324	-1.810802	0.03998733	0.377133998	down	yes	NA	NA
Pavir.8NG117900.v	GO:000384	00250(Alar	l-glutamate	4.946315	3.10554	18.99135	9.187499	0.2857411	-1.80722	0.036833564	0.361155398	down	yes	Delta-1-pyr	ALDH12A1
Pavir.8KG299500.v	GO:000372	03010(Ribo	large subun	4.000668	4.468334	21.912291	7.627087	0.2867021	-1.802376	0.046694592	0.406640471	down	yes	NA	NA
Pavir.2NG214600.v	GO:000367	03013(RNA	alba dna/rna	3.866021	3.783955	19.08535	7.524936	0.2874819	-1.798457	0.042288954	0.387825942	down	yes	NA	NA
Pavir.4KG309400.v	GO:000382	NA	hypothetical	2.430713	3.120996	12.042747	7.219033	0.2882241	-1.794737	0.031637534	0.333160613	down	yes	NA	NA
Pavir.9NG015700.v	GO:000003	NA	acyl carrier	10.683095	6.31895	41.834431	17.110607	0.2884389	-1.793662	0.042441977	0.3885654	down	yes	Acyl carrier	ACL1.3;ACL1.1
Pavir.3NG243800.v	GO:000588	NA	boron trans	7.264557	2.439568	21.303299	12.300391	0.2887815	-1.79195	0.045511206	0.402351186	down	yes	Probable bo	BOR2
Pavir.3NG262400.v	GO:001678	00590(Arac	alpha/beta h	20.55154	19.001427	81.173111	55.41124	0.2895864	-1.787934	0.045757763	0.403146483	down	yes	NA	NA
Pavir.8NG271800.v	GO:001988	NA	trna-2-meth	4.089163	3.195009	15.072507	10.058022	0.2898535	-1.786604	0.030135134	0.324425589	down	yes	CDK5RAP	Os11g0592800;Os11g0592800
Pavir.9KG220200.v	NA	NA	0	8.002679	2.475173	21.792557	14.141128	0.2915886	-1.777994	0.048409188	0.414700961	down	yes	NA	NA
Pavir.9NG500400.v	GO:000455	00500(Stan	alpha,alpha	4.925033	5.859097	20.43161	16.532627	0.291745	-1.77722	0.029362058	0.320064917	down	yes	Probable tre	Os10g0521000
Pavir.2NG601900.v	GO:000016	03013(RNA	nucleolin (n	10.231055	11.863091	47.654138	27.994589	0.2920624	-1.775652	0.043826192	0.395551651	down	yes	29 kDa ribo	-;-
Pavir.7NG232000.v	GO:000428	NA	plant specif	6.075383	4.29332	24.906597	10.551963	0.2924175	-1.773899	0.041113213	0.381513149	down	yes	Eukaryotic t	EIF4B
Pavir.9KG132000.v	NA	NA	protein of u	6.073766	2.384724	15.103677	13.667422	0.2939926	-1.766148	0.042200554	0.387366693	down	yes	NA	NA
Pavir.9NG113700.v	GO:000367	03013(RNA	alba dna/rna	4.8696742	5.667381	21.237056	14.570932	0.2942655	-1.76481	0.031360099	0.331318717	down	yes	NA	NA
Pavir.3KG054200.v	GO:000474	01200(Carb	dihydrolipo	10.675833	5.9887429	35.203835	21.344226	0.2946976	-1.762693	0.042730487	0.389403975	down	yes	Dihydrolipo	EMB3003;EMB3003
Pavir.7KG282100.v	GO:000662	NA	thioesterase	10.145056	5.828214	24.823556	29.171267	0.2958297	-1.757161	0.037459494	0.364237061	down	yes	Acyl-acyl ca	ALT3;ALT3
Pavir.7NG416200.v	GO:000013	NA	e3 ubiquitin	4.8301465	2.8696535	7.3757349	18.557317	0.2969107	-1.751899	0.044668395	0.398557495	down	yes	Receptor ho	Os03g0167500;Os03g0167500;Os03g0167500;Os03g0167500;Os03g0167500;Os03g0167500;Os03g0167500;Os03g0167500;Os03g0167500;Os03g016750
Pavir.8KG357200.v	GO:000372	NA	programmed	17.496397	15.695543	77.120918	34.573032	0.2971686	-1.750646	0.040893579	0.380508779	down	yes	NA	NA
Pavir.9NG796900.v	GO:000003	00062(Fatty	3-ketoacyl-c	4.109607	6.8854828	19.550168	17.415079	0.297444	-1.74931	0.034905797	0.351424308	down	yes	3-ketoacyl-C	FDH;FDH
Pavir.8NG087700.v	GO:000524	NA	chloride cha	4.656482	4.929053	18.453611	13.511099	0.2998787	-1.737549	0.036407332	0.35856689	down	yes	Chloride ch	CLC-A
Pavir.6KG348000.v	GO:000047	03010(Ribo	ribosomal p	17.13545	15.592203	79.692329	29.11507	0.3007852	-1.733195	0.04830223	0.414226418	down	yes	60S ribosor	RPL7A-2
Pavir.6NG216700.v	NA	00940(Pher	transferase f	6.191333	3.462527	18.990957	12.968624	0.3020647	-1.727071	0.048305653	0.414226418	down	yes	Putrescine I	PHT1
Pavir.3KG487700.v	GO:000382	NA	molybdopte	2.215983	1.896926	8.470916	5.140579	0.3021644	-1.726594	0.044394898	0.398226279	down	yes	Molybdenu	FLACCA
Pavir.9NG145500.v	GO:000579	NA	solute carric	5.152053	2.268323	15.036993	9.514328	0.3022394	-1.726236	0.047911983	0.412889206	down	yes	Probable su	At3g17430
Pavir.5NG308300.v	GO:000563	NA	inactive tpr	5.7383441	3.8200098	18.529367	13.027807	0.30289	-1.723134	0.04400454	0.396121577	down	yes	TPR repeat-	TTL1;TTL1
Pavir.9NG764000.v	GO:000367	NA	domain of u	2.726568	1.611169	7.398633	6.78819	0.3057582	-1.709537	0.043105794	0.39157121	down	yes	NA	NA
Pavir.3KG099500.v	GO:000550	NA	nitrogen me	11.233457	5.916673	33.777142	21.796783	0.3086003	-1.696189	0.045798387	0.403216066	down	yes	Uncharacter	At2g37660
Pavir.1NG053600.v	GO:000497	NA	ionotropic g	3.4774699	2.11284	9.8237513	8.2733788	0.3089059	-1.694761	0.040867523	0.380454766	down	yes	Glutamate r	GLR3.1;GLR3.1;GLR3.1;GLR3.1
Pavir.1KG101800.v	GO:001616	00591(Linc	lipoxygenas	2.502285	4.514069	12.932435	9.697968	0.3100411	-1.689469	0.044567255	0.398414767	down	yes	Lipoxygena	LOX2.3
Pavir.2NG317200.v	GO:000028	00562(Inos	chaperonin/	0.9187655	1.284603	3.765851	3.3353946	0.3102792	-1.688361	0.044124334	0.396717782	down	yes	Putative 1-p	FAB1C;FAB1C;FAB1C
Pavir.2NG638200.v	GO:000016	03013(RNA	nucleolin (n	6.288573	8.508588	29.849716	17.826826	0.3103657	-1.687959	0.045430677	0.401733783	down	yes	29 kDa ribo	-
Pavir.5NG002000.v	GO:000374	NA	translation i	7.247593	5.995677	27.825201	14.530244	0.3126698	-1.677288	0.046373065	0.405769721	down	yes	NA	NA
Pavir.4KG016100.v	GO:000398	00650(Buta	hydroxymet	3.8512108	5.9097474	19.633073	11.53929	0.3131286	-1.675173	0.048166512	0.413516993	down	yes	Hydroxyme	HMGS;HMGS
Pavir.5KG010800.v	GO:000374	NA	translation i	5.089982	4.72797	20.393784	10.681121	0.3159447	-1.662256	0.048749382	0.416286485	down	yes	NA	NA

Pavir.8NG200300.v4	GO:000551	00940(Pher	shikimate o-	6.725273	4.471611	19.551043	15.189902	0.3222965	-1.63354	0.048514871	0.415133057	down	yes	Putrescine h	PHT1

Table S3. List of primer pairs used in this study.

Primer	5'-3' Sequence
**Primer amplid for subcellular localization construct**	
WOX3a-1300cGFP-F	CGACTCTAGAAAGCTTATGCCGCAGACGCCGTCGA
WOX3a-1300cGFP-R	CGGGCCCCTGCAGAAGCTTGTTGGTGGATGTGGAGC
**Primer amplid for PvWOX3a-OE construct**	
WOX3a-F	TCCTTCACCCGGGATCCTCCATCTGTCCCAACTACAA
WOX3a-R	ACCCTTTATCGGGATCCGACACAATAAAACCTATGACCACT
**Primer amplid for genomic PCR to detect positive transgenic plants**	
hph3	AAGGAATCGGTCAATACACTACATGG
hph4	AAGACCAATGCGGAGCATATACG
ZmUbiF	TGTCGATGCTCACCCTGTTG
PvWOX3a-R	GACACAATAAAACCTATGACCACT
**Primer amplid for qRT-PCR**	
PvUBQ2-F	TTCGTGGTGGCCAGTAAG
PvUBQ2-R	AGAGACCAGAAGACCCAGGTACAG
WOX3a-cds-QF	GGTGGCTATGGAGGAACAG
WOX3a-cds-QR	GCAGGAGGAGGATTTGGA
WOX3a-3‘UTR-QF	CATTTAGTCCCAGTAATAAGAGTG
WOX3a-3‘UTR-QR	TACAACGAGATCCTCAAATAGAA
CPS-0400QF	CTGATGGGACGGACTTGC
CPS-0400-QR	CATACGCCGAGATGCTGA
KO1-2900-QF	ATGCTTGTTTGGTGGCTGAA
KO1-2900-QR	CACAACCCCAATAATCCTGTCAT
KO2-QF	GGCGTTGAACATCTCGTGC
KO2-QR	GGTGTCAGCCTTGTCCTCGT
KAO-5300-F	GAGACACTGCGCTTCGTCAA
KAO-5300-R	CGAGGAGGAAATGGTGGAGG
GA20ox2-6900-QF	TTCTTCATTGCCAGTTGCC
GA20ox2-6900-QR	TGCCTCCCTATCGAGTGTTCT
GA3ox2-5800-QF	GAGACCAAGACAAAGACGAAACG
GA3ox2-5800-QR	GACAAACTCGGCTGGGAGC
PvGA2ox3-QF	CGGGATGATGGCGAGGTAGA
PvGA2ox3-QR	GCAGCAGATGCCGAAACA
PvGA2ox7-QF	GCCTGACGGGGTTCGGCGAGCACAC
PvGA2ox7-QR	TGGAGCGGAGCACGGAGATG
PvCKX4b-FUQF	CTAGAGTTCTTGGACAGGGTG
PvCKX4b-FUQR	TCTTTCAGGATCTTGCCGAAG

**Primer amplid for yeast one-hybrid construct**	
WOX3-AD-F	GCATCGATACGGGATCATGCCGCAGACGCCGTCGA
WOX3-AD-R	CTCGAGCTCGATGGATTAGTTGGTGGATGTGGAGC
PvGA2ox3-proF-pHIS	TAGGGCGAATTCGAGCTCCTCGGACGGAATAAAGAAACG
PvGA2ox3-proR-pHIS	GGATCCACGCGTGAGCTCTTGACGACCTTGAAGAACCC
PvGA2ox7-proF-pHIS	TAGGGCGAATTCGAGCTCAGCCGCAATGTGATG
PvGA2ox7-proR-pHIS	GGATCCACGCGTGAGCTCCTCCTCGTCCTTCTCG
PvCKX4b-proF-pHIS	TAGGGCGAATTCGAGCTCGGCTGGTTATGGTTAGTGG
PvCKX4b-proR-pHIS	GGATCCACGCGTGAGCTCAGAGGGCTTCTTGTAGTTCC
**Primer amplid for luciferase assay system construct**	
PvGA2ox3-proF-pgreen	ATTCCTGCAGCCCGGGGCTCGGACGGAATAAAGAAACG
PvGA2ox3-proR-pgreen	CTAGAACTAGTGGATCTTGACGACCTTGAAGAACCC
PvGA2ox7-proF-pgreen	ATTCCTGCAGCCCGGGGAGCCGCAATGTGATG
PvGA2ox7-proR-pgreen	CTAGAACTAGTGGATCCTCCTCGTCCTTCTCG
PvCKX4b-proF-pgreen	ATTCCTGCAGCCCGGGGGGCTGGTTATGGTTAGTGG
PvCKX4b-proR-pgreen	CTAGAACTAGTGGATCAGAGGGCTTCTTGTAGTTCC
**Primer amplid for qRT-PCR of matrue miR156**	
SL156RT	GTCGTATCCAGTGCAGGGTCCGAGGTATTCGCACTGGATACGACGTGCTC
SL168RT	GTCGTATCCAGTGCAGGGTCCGAGGTATTCGCACTGGATACGACTTCCCG
miR156RT-F	CGGCGGTGACAGAAGAGAGT
miR168RT-F	TGCTCGCTTGGTGCAGAT
miRNART-R	GTGCAGGGTCCGAGGT
